# Global burden and trends of smoking-related low back pain in women of childbearing age and projections to 2032: A secondary dataset analysis of Global Burden of Disease 2021

**DOI:** 10.18332/tpc/218955

**Published:** 2026-10-08

**Authors:** Qiliang Xu, Degen Tian, Chengtian Xiao, Xuecheng Huang, Haoxiong Chen, Guosheng Tang

**Affiliations:** 1 Shenzhen Hospital (Futian) of Guangzhou University of Chinese MedicineShenzhenChina; 2 Sichuan Provincial Orthopaedic HospitalChengduChina

**Keywords:** smoking-related low back pain, women of childbearing age, global burden of disease, DALYs, epidemiology

## Abstract

**Introduction:**

This study used Global Burden of Disease (GBD) data to examine trends in smoking-related low-back pain (LBP) among women of childbearing age (WCBA) and project future trends.

**Methods:**

This study is a secondary analysis of the GBD 2021 data. This study analyzes the temporal trends in the disability-adjusted life years (DALYs) caused by smoking-related LBP between 1990 and 2021 across 204 countries and territories. We evaluated the changing trends in the burden of smoking-related LBP in WCBA using the estimated annual percentage change (EAPC) and percentage change and Bayesian age-period-cohort modeling to forecast the burden to 2032.

**Results:**

Globally, smoking-related low-back pain DALYs among women of childbearing age declined by 3% from 1990 to 2021, with the age-standardized rate falling from 84.58 to 56.54 per 100000 (EAPC= ‐1.39%). Regional disparities were pronounced: high-SDI regions accounted for 40.8% of the 2021 burden but saw a 22% decline, whereas low-SDI regions experienced a 54% increase. The largest national increases occurred in Qatar (733.3%) and the UAE (715.4%). Eastern Europe showed the greatest regional EAPC increase (1.85%), while Tropical Latin America declined most sharply (‐2.68%). The age group of 45–49 years exhibited the highest 2021 DALY rate (140.2 per 100000), increasing 22% since 1990, contrasting with a 20% decline among those aged 30–34 years. Frontier analysis identified Greece, Chile, and Slovenia as having the greatest improvement potential. BAPC modeling projects a 17.24% decline in the global age-standardized DALY rate by 2032.

**Conclusions:**

The burden of smoking-related LBP in WCBA is shifting from high-income to low- and middle-income regions, with middle-SDI countries and older reproductive-age women facing escalating risks.

## Introduction

Low-back pain (LBP) is a leading cause of disability worldwide, imposing a significant burden on individuals, healthcare systems, and societies^1-4^. It is a primary contributor to years lived with disability, affecting millions of people across diverse demographic groups^5^. Among the modifiable risk factors for LBP, smoking has been identified as a key contributor, negatively impacting the musculoskeletal system^6,7^. Smoking impairs spinal health by reducing blood flow, impairing tissue repair, and promoting inflammation, which collectively exacerbate LBP^8^. Consequently, curbing smoking prevalence represents a pivotal strategy for mitigating the global burden of LBP.

However, the epidemiological profile of smoking-related LBP is far from homogeneous across the globe. While high-income nations have often experienced parallel declines in smoking rates and associated musculoskeletal morbidity, this trajectory is not universally observed^9^. In numerous low- and middle-income countries (LMICs), smoking prevalence remains high or is even escalating within specific subpopulations^10,11^. This divergence, compounded by disparities in healthcare access and public health capacity, suggests a dynamic and shifting burden. Smoking-related LBP may be increasingly concentrated in already resource-constrained settings, threatening to overwhelm fragile health systems^6^.

Within the broader population, women of childbearing age (WCBA) constitute a particularly vulnerable demographic whose specific risk for smoking-related LBP has been largely underappreciated in epidemiological research^9^. This group exists at a critical intersection of biological, physiological, and social risk factors. Biologically, hormonal fluctuations across the menstrual cycle and during pregnancy can modulate pain perception and increase ligamentous laxity, potentially heightening spinal vulnerability^12-14^. Physiologically, the biomechanical demands of pregnancy and childrearing impose unique stresses on the musculoskeletal system^15-17^. When these inherent susceptibilities are compounded by the direct pathological assaults of smoking, including microvascular damage and impaired tissue healing, the likelihood of developing chronic and debilitating LBP is likely amplified^6,7^. Furthermore, given the well-documented risks of smoking during pregnancy for fetal development, tobacco cessation in this group carries a dual imperative for both maternal and child health^18^.

Despite this clear convergence of risks, detailed epidemiological evidence specifically quantifying the burden of smoking-related LBP among WCBA remains absent. Existing investigations often address LBP and smoking as separate entities or analyze mixed-gender cohorts, thereby obscuring the distinct experience of this population^9,19^. Critically, the variation of this burden across different sociodemographic contexts – from high-income regions with advanced healthcare to low-income areas with profound resource limitations – has not been systematically quantified.

To address this critical research gap, the present study harnesses the latest data from the GBD 2021 study to deliver a comprehensive analysis of the global burden of smoking-related LBP among women of childbearing age. Trends in disability-adjusted life years (DALYs) from 1990 to 2021 were systematically analyzed across 204 countries and territories, examining variations by age, geographical region, and sociodemographic index (SDI). Furthermore, A Bayesian age-period-cohort (BAPC) model was employed to project the future trajectory of this burden to the year 2032.

## Methods

### Data source

This study is a secondary analysis of the Global Burden of Disease (GBD) 2021 data. Data on smoking-related LBP were obtained from the GBD 2021 Results Tool, accessible via the Global Health Data Exchange (GHDx) platform^20^. The GBD 2021 study systematically compiled information from 100983 diverse sources, including vital registration systems, cause-of-death analyses, population censuses, household surveys, disease registries, and records of health service contacts, to provide a comprehensive overview of the incidence, prevalence, and DALYs associated with 371 diseases and injuries^21^.

To address variations arising from differences in data sources, definitions, and measurement meta-regression, a Bayesian meta-methods, the study applied systematic adjustments to epidemiological datasets. Advanced statistical modeling techniques, such as MR-BRT analysis (a meta-regression with Bayesian priors, regularization, and trimming analysis), were assessed by DisMod-MR 2.1 (disease model-Bayesian regression modeling tool)^22^, were employed to ensure consistency in estimates across geographical regions, age groups, and sexes. Through standardized and calibrated analytical procedures, the study minimized the influence of data heterogeneity on the results, thereby establishing a robust and reliable basis for global health research.

### Definitions

LBP is clinically defined as pain localized between the lower margin of the 12th rib and the gluteal fold, which may present with or without radiation to one or both lower limbs and must persist for at least 1 day. According to the International Classification of Diseases, Ninth Revision (ICD-9), this condition is coded as 724, corresponding specifically to LBP^23^. Additionally, in line with the 2021 Global Health Guidelines, smoking is defined as current use of any tobacco product or a history of former smoking with abstinence for at least 6 months^24^.

In this study, DALYs for smoking-related LBP from the GBD 2021 data were selected as the primary metric for analysis. DALYs combine years of life lost due to premature mortality and years lived with disability, offering a comprehensive measure of disease burden that encompasses both fatal and non-fatal health outcomes. This indicator enables direct comparison of disease impact across different health conditions and populations, and serves as a key reference for assessing health disparities^21^.

Furthermore, the SDI was employed as a composite indicator reflecting regional development levels. The SDI is derived from the geometric mean of three normalized components: the total fertility rate among individuals under 25 years, average years of education in those aged ≥15 years, and lag-distributed income per capita. Calculated at the national level, it provides a reliable representation of socioeconomic development across countries and regions^21^. Based on SDI values, the 2021 GBD study categorized 204 countries and territories into five development tiers: low, low-middle, middle, high-middle, and high SDI^21^.

### Statistical analysis

The estimated annual percentage change (EAPC) is a widely used metric for assessing trends in key health indicators, such as prevalence and incidence-over defined time periods^25^. In this study, the EAPC was employed to evaluate trends in DALYs attributable to smoking-related LBP from 1990 to 2021. The calculation was based on a linear regression model in which the calendar year served as the independent variable and the natural logarithm of the DALY rate as the dependent variable. The model is expressed as: y=α+βx+ε, where x denotes the year, y represents the natural logarithm of the rate, α is the intercept, β is the slope, and ε is the random error term^21^. The EAPC was derived as: EAPC=100×[exp(β)−1], with the 95% confidence interval (CI) obtained from the regression fit. Trend interpretation was based on the CI: an upward trend was concluded if the lower 95% CI limit was above zero, a downward trend if the upper limit was below zero, and no significant trend if the CI included zero^25^. A frontier analysis was conducted to compare observed DALYs with ideal values predicted by SDI levels across 204 countries and territories, highlighting regions with the largest observed-to-ideal disparities^26^.

Furthermore, a Bayesian age-period-cohort (BAPC) model was applied to project global DALYs trends from 2022 to 2032^26^. This model assumes smooth effects across adjacent age, period, and cohort groups. It employs a second-order random walk prior and uses integrated nested Laplace approximation (INLA) for posterior estimation, which avoids convergence issues common in Markov chain Monte Carlo approaches. The BAPC framework has been extensively used in chronic disease epidemiology for burden forecasting^26^. All analyses were performed in R (version 4.4.3), utilizing the *BAPC* and *INLA* packages^27^. Statistical significance was defined as p<0.05.

## Results

### Global level

Globally, a modest decline in the number of DALYs attributable to smoking-related low-back pain among WCBA was observed between 1990 and 2021. Specifically, the total DALYs fell from 1131.08×10^3^ in 1990 to 1101.9×10^3^ in 2021, corresponding to a decrease of 3%. Similarly, the global DALY rate per 100000 population dropped significantly from 84.58 (95% UI: 47.14–133.89) in 1990 to 56.54 (95% UI: 31.09–88.22) in 2021. This trend was further quantified by an estimated annual percentage change (EAPC) of ‐1.39% (95% CI: ‐1.45 – ‐1.33) (Table 1).

**Table 1:** DALYs of low-back pain attributable to smoking and rates among WCBA in 1990 and 2021 and and the trends from 1990 to 2021

Location	DALYs			DALY rates		
	1990 Numbers in thousands (95% UI)	2021 Numbers in thousands (95% UI)	Percentage change	1990 Numbers per 100000 (95% UI)	2021 Numbers per 100000(95% UI)	EAPC (95% CI)
**Global**	1131.08 (630.42–1790.61)	1101.9 (605.93–1719.27)	−0.03	84.58 (47.14–133.89)	56.54 (31.09–88.22)	−1.39 (-1.45 − ‐1.33)
**DI regions**						
Low	27.75 (14.88–45.64)	42.67 (22.73–70.7)	0.54	24.84 (13.32–40.87)	15.56 (8.29–25.77)	−1.72 (‐1.87 – ‐1.58)
Low-middle	79.3 (41.36–127.23)	103.87 (53.76–178.86)	0.31	29.06 (15.16–46.62)	20.52 (10.62–35.33)	−1.22 (-1.27 – -1.17)
Middle	154.53 (83.4–250.37)	164.57 (86.26–269.3)	0.06	34.57 (18.66–56)	26.61 (13.95–43.54)	−1.1 (-1.2 – -0.99)
High-middle	290.37 (163.14–455.46)	339.64 (188.9–537.11)	0.17	104.54 (58.74–163.98)	111.31 (61.91–176.03)	0.33 (0.28–0.37)
High	576.72 (330.17–902.38)	449.12 (252.3–692.61)	−0.22	254.39 (145.64–398.04)	184.7 (103.76–284.84)	−1.22 (-1.31 – -1.13)
**Regions**						
Andean Latin America	1.81 (0.95–3.11)	3.43 (1.8–5.65)	0.9	19.04 (10–32.77)	19.68 (10.29–32.39)	−0.04 (-0.12–0.03)
Australasia	19.27 (11.17–30.45)	20.78 (11.16–33.07)	0.08	359.06 (208.11–567.35)	287.89 (154.65–458.11)	−0.89 (-1.07 – -0.72)
Caribbean	6.38 (3.46–10.48)	6.12 (3.27–10.02)	−0.04	68.45 (37.17–112.45)	50.87 (27.17–83.27)	−1.19 (-1.47 – -0.91)
Central Asia	5.25 (2.82–8.87)	9.87 (5.01–16.26)	0.88	31.27 (16.8–52.9)	40.66 ( 20.64–67)	0.96 (0.77–1.16)
Central Europe	116.43 (67.95–180.06)	94.41 (53.77–146.56)	−0.19	379.12 (221.25–586.28)	366.6 (208.81–569.1)	−0.18 (-0.27 – -0.09)
Central Latin America	30.29 (16.24–48.49)	34.3 (18.23–56.53)	0.13	72.27 (38.74–115.68)	50.29 (26.73–82.89)	−1.47 (-1.61 – -1.32)
Central Sub-Saharan Africa	1.34 (0.68–2.28)	3.31 (1.67–5.71)	1.47	10.81 (5.52–18.43)	10.12 (5.13–17.5)	−0.23 (-0.33 – -0.13)
East Asia	59.37 (29.9–100.95)	57.04 (27.1–102.54)	−0.04	17.81 (8.97–30.28)	17.24 (8.19–30.99)	0.34 (0.2–0.49)
Eastern Europe	79.61 (43.35–127.78)	109.47 (60.45–175.59)	0.38	143.97 (78.4–231.09)	226.92 (125.3–363.97)	1.85 (1.69–2.01)
Eastern Sub-Saharan Africa	6.32 (3.45–10.45)	13.96 (7.52–22.27)	1.21	14.65 (8–24.21)	13.04 (7.02–20.8)	−0.4 (-0.42 – -0.37)
High-income Asia Pacific	71.2 (38.67–116.1)	52.26 (26.55–86.8)	−0.27	155.65 (84.54–253.83)	137.39 (69.8–228.18)	−0.37 (-0.57 – -0.18)
High-income North America	245.03 (140.02–384.38)	179.26 (102.62–275.91)	−0.27	329.49 (188.28–516.87)	213.35 (122.13–328.38)	−1.57 (-1.63 – -1.5)
North Africa and Middle East	30.27 (16.99–48.92)	72.57 (39.05–121.05)	1.4	38.75 (21.74–62.62)	45.54 (24.51–75.97)	0.52 (0.47–0.57)
Oceania	1.02 (0.55–1.67)	2.68 (1.48–4.36)	1.63	65.9 (35.11–107.69)	77.25 (42.66–125.6)	0.45 (0.4–0.51)
South Asia	64.27 (32.75–105.63)	79.24 (39.02–144)	0.23	25.21 (12.85–41.44)	16.04 (7.9–29.14)	−1.54 (-1.64 – -1.45)
Southeast Asia	24.88 (13.03–42.1)	35.98 (18.9–59.77)	0.45	20.7 (10.84–35.03)	19.64 (10.31–32.63)	−0.25 (-0.4 – -0.1)
Southern Latin America	35.99 (20.37–54.3)	45.28 (25.47–71.28)	0.26	290.41 (164.4–438.19)	259.78 (146.14–408.98)	−0.6 (-0.73 – -0.48)
Southern Sub-Saharan Africa	6.35 (3.62–10.55)	6.71 (3.56–11.31)	0.06	47.77 (27.24–79.39)	30.92 (16.4–52.08)	−1.6 (-1.74 – -1.46)
Tropical Latin America	77.37 (42.6–120.86)	61.34 (32.71–99.68)	−0.21	193.95 (106.88–302.98)	101.2 (53.9–164.46)	−2.68 (-2.9 – -2.46)
Western Europe	244.84 (142.6–376.89)	206.28 (118.01–320.4)	−0.16	256.25 (149.25–394.45)	221.38 (126.65–343.86)	−0.51 (-0.65 – -0.36)
Western Sub-Saharan Africa	3.79 (2.02–6.32)	7.61 (3.87–12.32)	1.01	8.7 (4.63–14.49)	6.35 (3.23–10.27)	−1.1 (‐1.31 – -0.9)

CI: confidence interval; DALYs: disability-adjusted life years; EAPC: estimated annual percentage change; UI: uncertainty interval; WCBA: women of childbearing age.

### SDI region level

In 2021, the high SDI region recorded the highest absolute number of DALYs attributable to smoking-related low-back pain among WCBA, reaching 449.12×10^3^ (95% UI: 252.3–692.61). This figure represented approximately two-fifths of the global total DALY burden for this condition. Geographically, the high SDI regions also exhibited the highest DALY rates, and DALY cases increased progressively with decreasing SDI levels. The low SDI region experienced the most substantial relative increase, with a percentage change of approximately 54% over the studied period. Conversely, the high SDI region demonstrated a notable negative growth in DALYs, declining by about 22%. Trend analysis from 1990 to 2021 revealed that the high-middle SDI region underwent a rapid rise in DALY rates, with an EAPC of 0.33% (95% CI: 0.28–0.37), as supported by data in Table 1. In contrast, by 2021, other SDI regions aligned with the global pattern of negative DALY rates of EAPC. Most strikingly, the low SDI region registered the most significant decline, with an EAPC of ‐1.72% (95% CI: -1.87 – ‐1.58) (Table 1 and Figure 1).

**Figure 1:**
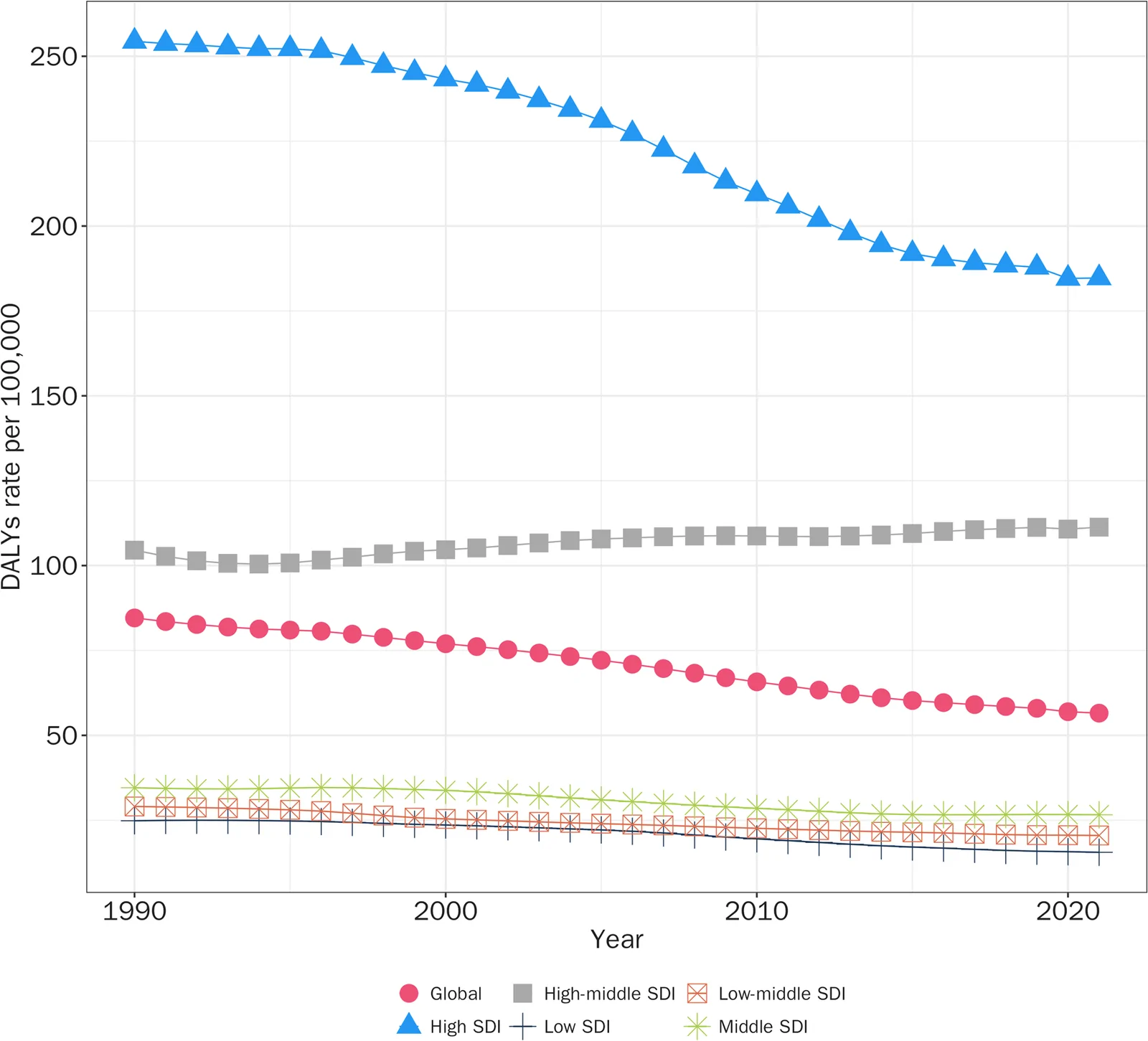
emporal trends of smoking-related low-back pain burden in WCBA for global and 5 SDI regions; the rates of DALYs from 1990 to 2021

### GBD regional level

Over the observed period, the absolute number of DALYs attributable to smoking-related LBP among WCBA increased in most regions. A declining trend was noted only in the Caribbean, Central Europe, East Asia, high-income Asia Pacific, high-income North America, Tropical Latin America, and Western Europe, all classified as high or high-middle SDI regions. Between 1990 and 2021, age-standardized DALY rates decreased across most regions. The most pronounced reduction occurred in Tropical Latin America, with an EAPC of -2.68% (95% CI: -2.90 – -2.46). In contrast, Central Asia, Eastern Europe, North Africa, and the Middle East, and Oceania experienced increases in DALY rates, with Eastern Europe showing the largest rise, with EAPC of 1.85% (95% CI: 1.69–2.01). Notably, trends in DALY numbers and rates diverged in ten regions. In several high-SDI areas – including the Caribbean, Central Europe, high-income Asia Pacific, high-income North America, Tropical Latin America, and Western Europe – both the number and rate of DALYs exhibited significant declines (Table 1 and Figure 2).

**Figure 2:**
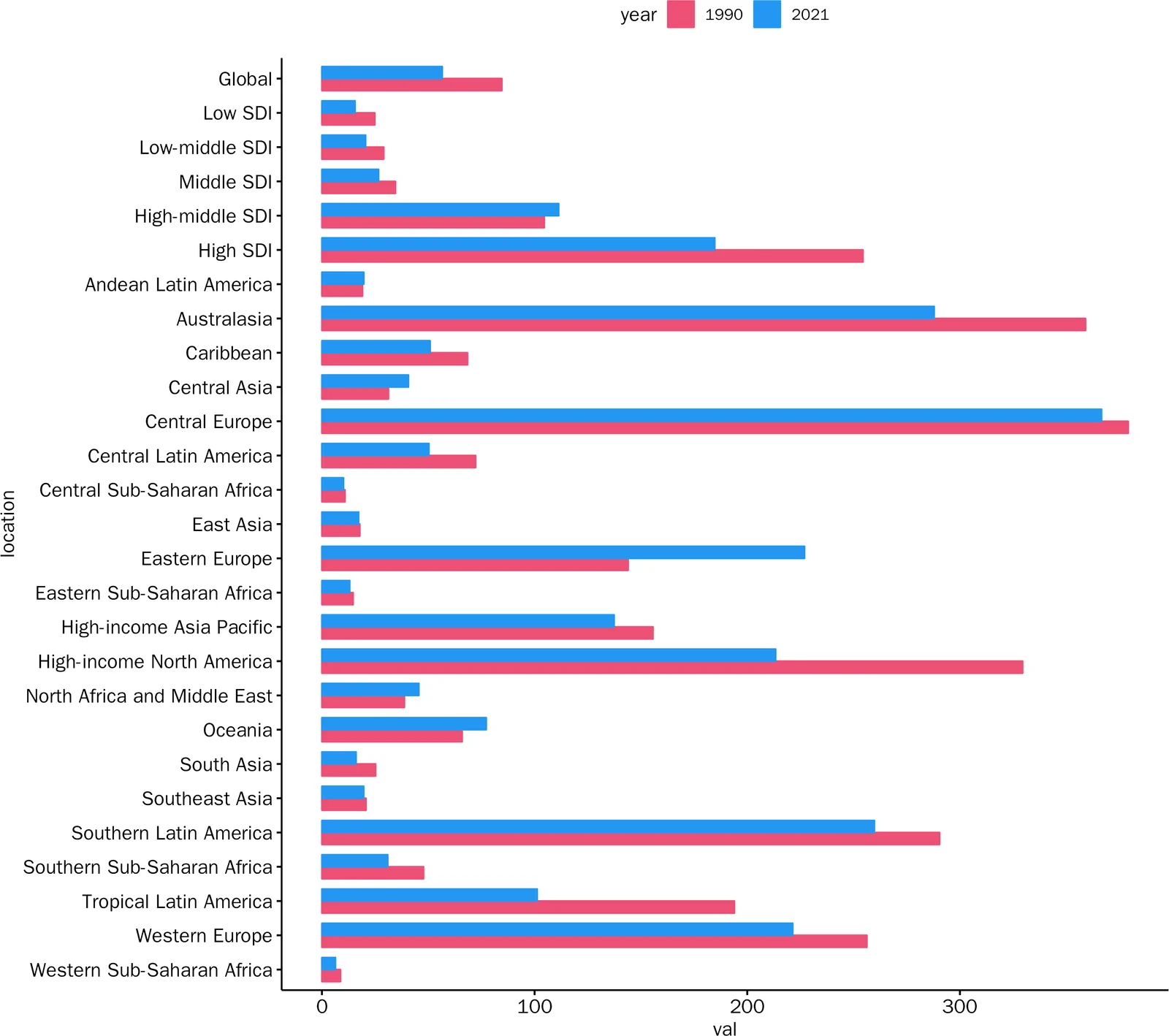
Temporal trends of smoking-related low-back pain burden in WCBA for different regions; DALY rate per 100000 population in 1990 and 2021

### Countries level

The global DALYs attributable to smoking-related low-back pain exhibited considerable variation across countries between 1990 and 2021. In 1990, the highest DALYs were recorded in the US (221387.25; 95% UI: 125265.53–347172.68). By 2021, this pattern showed no obvious change, with the highest number observed in the US (161678.03; 95% UI: 93035.05–251773.57) in 2021. Approximately 76% of countries exhibited an increasing trend in DALYs of smoking-related LBP among WCBA from 1990 to 2021. The highest percentage changes were observed in Qatar and the United Arab Emirates, both classified as high SDI regions, ranging from 71.5–73.3%. This contrasts with the notably lower percentage changes observed in other high SDI regions. Additionally, only 23.53% of countries exhibited a decrease in the DALYs, such as Hungary and Bulgaria (middle-SDI), Denmark, Germany, and Finland (high-SDI), with percentage changes ranging from -1% to -50%. Over the past 32 years, the DALY rates in different countries have shown different trends; the most significant increases in DALY rates were observed in Mongolia and Albania, with EAPC of 3.47% (95% CI: 3.3–3.63) and 3.28% (95% CI: 2.95–3.61). Among the countries where DALY rates have declined, Nepal has shown the most significant decreasing trend, with EAPC of -3.16% (95% CI: -3.55 – -2.77) (Figure 3; and Supplementary file Table S1).

**Figure 3:**
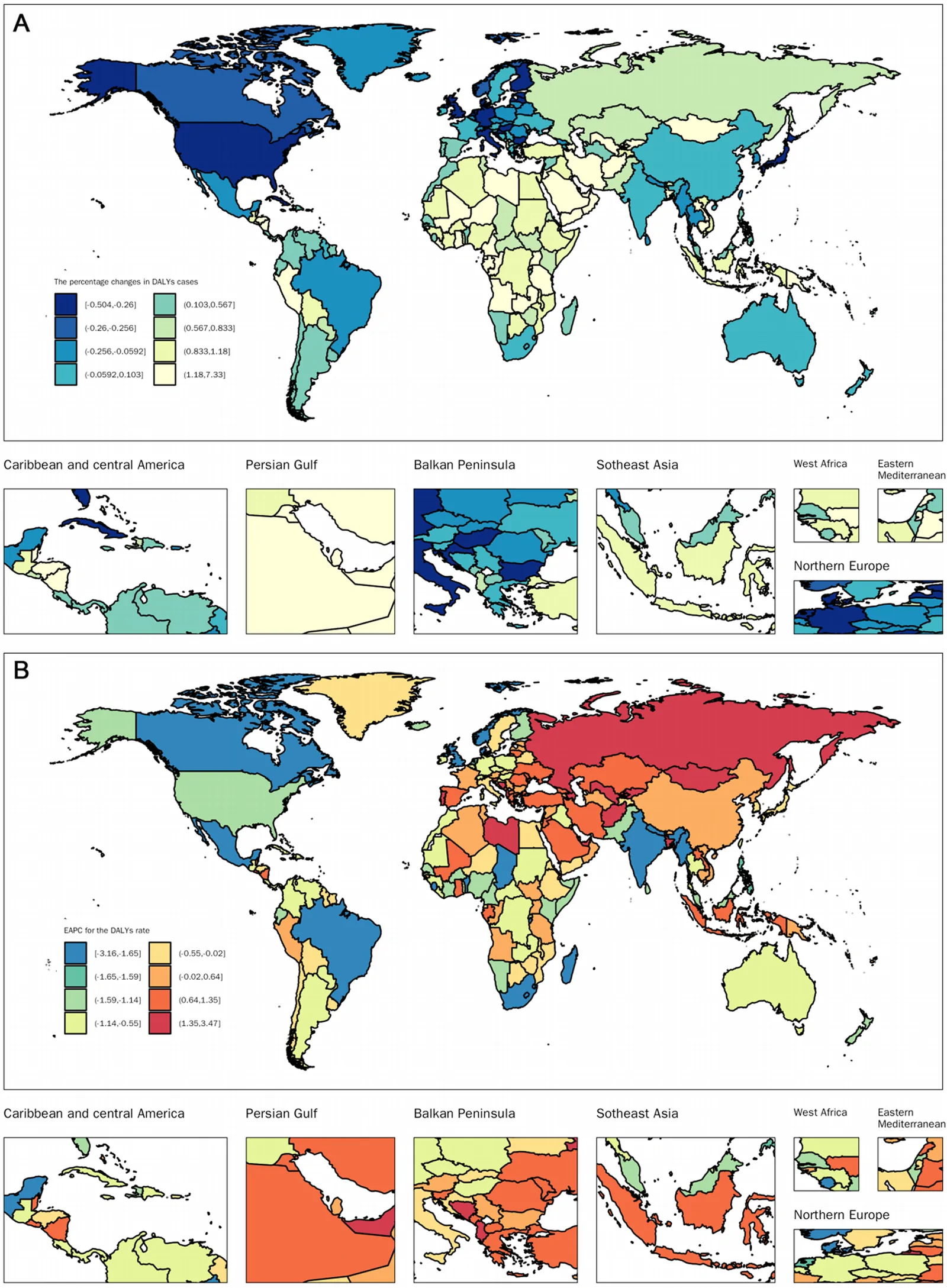
Temporal trend of smoking-related low-back pain burden in WCBA globally: A) Percentage change in DALYs across 204 countries in 1990 and 2021; B) EAPC in DALY rates across 204 countries from 1990 to 2021

### Age trends

The detailed trends of smoking-related LBP cases over the past 32 years in both the global and the five SDI regions are illustrated in Supplementary file Figure 1. Globally, the percentage change in smoking-related LBP DALYs among WCBA generally decreased across age groups, with the most pronounced decline observed in the 30–34 years age group by -20%. In contrast, the age group of 45–49 years showed an increase of 22%. Across SDI regions, the percentage change in LBP cases among WCBA varied by age. The low SDI region consistently showed the highest percentage changes across all age groups, remaining stable at approximately 56%. From low-middle to high SDI regions, the percentage change generally increased with age. For example, in the high-middle SDI region, the percentage change rose from -0.11% in the 30–34 age group to 0.5% in the age group of 45–49 years. Notably, in middle SDI regions, the 30–34 years age group experienced a sharp increase of 59%, the highest across all age groups and SDI regions (Tales 2; and Supplementary file Figure 2).

Globally, DALY rates declined across all four age groups. Declines were also observed in low SDI regions, while low-middle SDI regions exhibited a similar pattern, falling rates accompanied by modest increases in numbers. Middle SDI regions experienced steeper declines, with the 35–39 years age group showing an EAPC of -2.27% (95% CI: -2.47 – -2.06). High-middle SDI regions also showed reductions, though to a less extent than low-SDI regions. A consistent age gradient was observed across all regions, with higher DALY rates in older age groups. Although rates have declined globally, the magnitude of decline varied, and case numbers increased in less developed regions (Table 2; and Supplementary file Figure 2).

**Table 2:** DALYs of low-back pain attributable to smoking and rates among WCBA in 1990 and 2021 and and the trends by age from 1990 to 2021

Location	Age (years)	DALYs		DALY rates			
		1990 Numbers in thousands (95% UI)	2021 Numbers in thousands (95% UI)	Percentage change	1990 Numbers per 100000 (95% UI)	2021 Numbers per 100000 (95% UI)	EAPC (95% CI)
**Global**	30–34	272.27 (143.82–450.92)	218.81 (111.59–375.99)	−0.2	143.22 (75.65–237.19)	73.2 (37.33–125.78)	-2.01 (-2.11 – -1.9)
	35–39	291.87 (157.19–468.12)	258.88 (136.79–424.98)	−0.11	168.27 (90.62–269.88)	93.19 (49.24–152.98)	-2.04 (-2.14 – -1.93)
	40–44	295.45 (158.67–478.49)	293.83 (154.38–472.72)	−0.01	210.7 (113.15–341.23)	118.44 (62.23–190.54)	-1.92 (-2.03 – -1.82)
	45–49	271.5 (140.62–447.91)	330.38 (168.86–554.63)	0.22	238.57 (123.56–393.6)	140.2 (71.66–235.37)	-1.75 (-1.89 – -1.61)
**SDI regions**							
Low	30–34	5.51 (2.7–10.22)	8.78 (4.26–15.4)	0.59	36.24 (17.76–67.22)	23.65 (11.47–41.48)	-1.61 (-1.71 – -1.51)
	35–39	6.83 (3.45–11.74)	10.2 (5.15–18.14)	0.49	53.05 (26.74–91.1)	32.02 (16.16–56.91)	-1.88 (-2.06 – -1.7)
	40–44	7.4 (3.81–12.47)	11.52 (5.67–19.6)	0.56	74.67 (38.41–125.8)	44.1 (21.72–75.06)	-1.87 (-2.08 – -1.66)
	45–49	7.99 (3.93–13.55)	12.17 (5.8–21.67)	0.52	96.3 (47.37–163.2)	58.6 (27.95–104.36)	-1.9 (-2.15 – -1.66)
Low-middle	30–34	16.77 (7.98–29.75)	19.74 (9.19–34.96)	0.18	44.77 (21.3–79.45)	26.71 (12.44–47.31)	-1.7 (-1.79 – 1.62)
	35–39	19.14 (9.44–32.89)	23.47 (11.61–41.45)	0.23	59.76 (29.47–102.66)	35.25 (17.44–62.25)	-1.81 (-1.9 – -1.72)
	40–44	21.26 (10.66–37.42)	27.85 (13.39–49.85)	0.31	81.94 (41.09–144.22)	48.28 (23.21–86.42)	-1.94 (-2.01 – -1.86)
	45–49	22.13 (10.45–40.04)	32.82 (15.25–60.19)	0.48	101.95 (48.14–184.49)	66.38 (30.85–121.75)	-1.41 (-1.45 – -1.38)
Middle	30–34	37.09 (18.86–63.46)	32.13 (15.44–57.94)	−0.13	61.77 (31.42–105.69)	32.54 (15.63–58.67)	-2.05 (-2.18 – -1.92)
	35–39	41.41 (21.43–67.73)	37.18 (18.91–63.66)	−0.1	74.7 (38.66–122.18)	40.57 (20.64–69.46)	-2.27 (-2.47 – -2.06)
	40–44	38.45 (19.94–65.84)	43.52 (21.73–74.88)	0.13	91 (47.19–155.81)	53.17 (26.55–91.48)	-2.06 (-2.25 – -1.86)
	45–49	37.58 (18.14–63.57)	51.73 (24.54–90.5)	0.38	110.89 (53.53–187.57)	63.78 (30.26–111.58)	-1.9 (-2.02 – -1.79)
High-middle	30–34	69.94 (37.31–113.76)	62.01 (31.51–105.33)	−0.11	167.53 (89.37–272.49)	120.43 (61.2–204.56)	-0.51 (-0.78 – -0.23)
	35–39	75.3 (40.4–120.77)	78.7 (42.78–127.49)	0.05	190.65 (102.29–305.76)	158.82 (86.33–257.29)	-0.32 (-0.44 – -0.21)
	40–44	74.92 (40–119.47)	93.49 (49.02–152.21)	0.25	243.7 (130.1–388.59)	205.39 (107.68–334.38)	-0.34 (-0.47 – -0.22)
	45–49	70.21 (34.99–116.75)	105.44 (53.68–177.41)	0.5	286.36 (142.7–476.21)	218.63 (111.31–367.86)	-0.8 (-0.94 – -0.66)
High	30–34	142.43 (76.53–233.06)	95.79 (49.67–161.22)	−0.33	401.39 (215.66–656.79)	255.89 (132.68–430.68)	-1.56 (-1.61 – -1.51)
	35–39	148.53 (81.59–236.88)	108.86 (58.86–172.52)	−0.27	444.19 (243.98–708.4)	286.99 (155.16–454.8)	-1.61 (-1.68 -1.53)
	40–44	152.78 (83.29–244.58)	116.9 (63.99–184.16)	−0.23	489.33 (266.74–783.34)	318.42 (174.31–501.64)	-1.43 (-1.5 – -1.36)
	45–49	132.97 (69.75–218.5)	127.57 (67.51–207.72)	−0.04	526.28 (276.04–864.77)	355.3 (188.01–578.52)	-1.15 (-1.27 – -1.04)

CI: confidence interval; DALYs: disability-adjusted life years; EAPC: estimated annual percentage change; UI: uncertainty interval; WCBA: women of childbearing age.

In 2021, the global DALYs in thousands for smoking-related LBP among WCBA aged 45–49 years were 330.38 (95% UI: 168.86–554.63), the highest among all age groups. The corresponding rate was 140.2 per 100000 (95% UI: 71.66–235.37), also the highest across age groups. This age group also exhibited the smallest decline in DALY rates from 1990 to 2021, with an EAPC of -1.75% (95% CI: -1.89 – -1.61), indicating a persistently high burden in this demographic (Table 2; and Supplementary file Figure 2).

Between 1990 and 2021, the proportion of smoking-related LBP cases among WCBA – measured by DALY rates – decreased in the 30–34 and 35–39 years age groups globally, but increased among those aged 40–44 and 45–49 yeras. Similar patterns were observed in low-middle to high-middle SDI regions. In low SDI regions, proportions declined in the 35–39 and 45–49 age groups but rose in the 30–34 and 40–44 age groups. In high SDI regions, only the 45–49 years age group showed an increasing trend. Among the 21 GBD regions, East Asia experienced the most notable increase in the proportion of smoking-related LBP cases in the 45–49 years age group, rising from 29.2% in 1990 to 39.5% in 2021. Conversely, Eastern Europe saw the largest decline in the 30–34 years age group, from 28–18.8% over the same period (Supplementary file Figure 3).

### The association between smoking-related LBP burden and SDI

As socio-economic development progresses, the burden of smoking-related LBP generally demonstrates a non-linear trajectory. Analysis across 21 regions reveals that when the SDI falls below 0.5, the burden increases gradually. Between SDI values of 0.5 and 0.6, it remains relatively stable, then rises more rapidly to peak around SDI 0.75. Beyond this threshold, a gradual decline is observed. Regions with lower SDI levels – including South Asia, Southeast Asia, and certain parts of Sub-Saharan Africa – exhibited comparatively lower DALY rates in earlier decades, though these have risen over time. In middle-SDI regions, a U-shaped pattern emerged between 1990 and 2021, characterized by an initial rise in DALYs followed by a decline as healthcare systems improved. This trend was particularly evident in Latin America and parts of the Middle East. In contrast, high-SDI regions such as North America, Europe, and high-income Asia Pacific initially recorded elevated DALY rates around 1990. These regions experienced a stabilization or reduction in burden over time. For instance, Western Europe and North America reported DALY rates of 100–200 per 100000 population in 1990, which decreased by 2021 following sustained tobacco control efforts (Supplementary file Figure S4).

### Frontier analysis

This study utilizes global data from 1990 to 2021, focusing on ASDR and the SDI to assess the potential for health improvements across countries and regions at various development levels. Supplementary file Figure S6A illustrates the unrealized health gains over this period. The results indicate that, as sociodemographic indicators improve, the effective difference grows significantly. This suggests that countries with higher SDI have a higher potential to mitigate health burdens. A total of 15 countries were identified as having the highest potential for improvement: Greece, Chile, Slovenia, Slovakia, Poland, Romania, Central Europe, Hungary, Czechia, North Macedonia, Croatia, Bosnia and Herzegovina, Serbia, Bulgaria, and Montenegro. These countries have higher ASDR values compared to similar countries, highlighting considerable room for health improvements. In low-SDI countries (SDI<0.5) such as Somalia, Niger, Ethiopia, Eritrea, and Gambia, significant progress has been made in mitigating disease burdens. However, some high SDI countries (SDI>0.85), including Germany, Switzerland, Monaco, Denmark, and Andorra, continue to show high effective differences, particularly in managing smoking-related LBP. This necessitates further improvements in these countries (Supplementary file Figure S5, and Tables S2 and S3).

### Global prediction

This study is grounded in comprehensive data sourced from the GBD study and employs the Bayesian age-period-cohort (BAPC) model to conduct a predictive analysis. The findings project a promising and significant downward trend in the age-standardized death rate (ASDR) attributable to smoking-related LBP over the period 2022 to 2032. This decline is anticipated to be particularly notable among WCBA. Specifically, the model forecasts that by the year 2032, the global ASDR for smoking-related LBP among the WBCA population will fall to approximately 86.13 per 100000 individuals, as visually detailed in Supplementary file Figure S6 and Table S4. This projected figure represents a substantial decrease of 17.24% compared to the baseline rate recorded in 2021. Collectively, these statistical insights underscore a positive and encouraging trajectory, indicating that the global health burden imposed by smoking-related LBP is on course to be significantly alleviated within the next decade.

## Discussion

This analysis evaluated the global, regional, and national patterns of smoking-related low-back pain (LBP) among women of childbearing age from 1990 to 2021. The age-standardized rate of DALYs attributable to smoking fell by about one-third during the study period (from 84.58 to 56.54 per 100000), and the estimated annual percentage change (EAPC) was ‐1.39%. Absolute DALYs decreased only modestly, emphasizing that reductions in age-standardized rates were offset by population growth and ageing. The Bayesian age-period-cohort model predicted a further 17.24% decline by 2032, suggesting that continued tobacco control and healthcare improvements will further ease the burden. The observed decline in smoking-related LBP among women aligns with broader reductions in global LBP burden and with evidence supporting a causal link between smoking and musculoskeletal pain^6,7^. The GBD 2021 study estimated that low-back pain affected 619 million people in 2020, with an age-standardized years-lived-with-disability (YLD) rate of 832 per 100000 and a 10.5% decrease since 1990^4^. The findings mirror these trends but highlight the specific contribution of smoking. In this cohort, the majority of regions exhibited negative EAPC values, indicating a consistent decline in age-standardized DALY rates. Prospective evidence from a UK Biobank cohort of 438510 participants demonstrated that heavy smokers (>30 cigarettes per day or >30 pack-years) had hazard ratios of 1.45 for developing back pain; risk reduction was evident among former smokers and those who cut consumption^3^. Mendelian randomization analyses further indicate that genetic liability to smoking initiation causally increases the risk of LBP, with depression, sedentary behavior, and insomnia mediating up to two-thirds of the effect^28^. Together these external studies provide mechanistic and causal support for the results and show that reductions in smoking prevalence are likely to drive decreases in LBP.

Despite overall declines, socio-economic disparities were evident. High sociodemographic index (SDI) regions accounted for roughly 40% of global DALYs in 2021 yet experienced a 22% decrease in age-standardized rates, whereas low-SDI regions saw a 54% increase and high-middle SDI regions displayed a small positive EAPC (0.33%). This inverted U-shaped relationship between SDI and burden suggests that countries at intermediate stages of development bear the greatest risk. High-SDI countries such as Denmark, Germany and Finland exhibited some of the largest reductions in DALY rates. By contrast, Qatar and the United Arab Emirates, despite high economic wealth, showed rapid increases in smoking-related LBP (over 700% increase in DALYs). These contrasting trajectories illustrate that wealth alone does not guarantee improvement: cultural norms, gender roles, targeted cessation programs and regulatory frameworks, all influence smoking prevalence among women.

Geographical variation was further reflected at the level of Global Burden of Disease (GBD) regions. Tropical Latin America exhibited the steepest decline (EAPC= ‐2.68%), whereas Eastern Europe and Central Asia saw increases (EAPC=1.85%). The US maintained the highest absolute DALY numbers across the study period, reflecting its large population and persistent smoking among women of reproductive age. Country-specific differences likely stem from the timing of smoking epidemics, enforcement of smoke-free laws, female empowerment, and co-occurring risk factors such as obesity and occupational exposures. For example, high-income nations that implemented taxes, packaging warnings, and bans on indoor smoking decades ago, have seen pronounced declines, whereas rapidly urbanizing regions in the Middle East and parts of Asia continue to experience rising smoking prevalence among women^29,30^.

The age-pattern analysis revealed that the burden of smoking-related LBP peaks in midlife. DALYs were highest in the age group of 45–49 years, with a rate of 140.2 per 100000, and this cohort even showed a 22% increase since 1990. Younger age groups experienced greater relative declines: the 30–34 years cohort declined by about 20%, and the 35–39 years cohort had the steepest negative EAPC (‐2.27%). These observations are consistent with the general epidemiology of LBP, which tends to peak in the fourth and fifth decades of life^3,4,9^. Biological mechanisms provide plausible explanations for this age-specific risk. Nicotine causes vasoconstriction and reduces blood flow to intervertebral discs, promoting disc degeneration, while carbon monoxide and oxidative stress impair vertebral bone metabolism; chronic smoking upregulates inflammatory cytokines and accelerates systemic catabolism^6-8^. Pregnancy-related biomechanical strain, hormonal changes and weight gain may further exacerbate spinal vulnerability among women of childbearing age, particularly when combined with prolonged smoking^12-17^.

The future trajectory of smoking-related low-back pain is also delineated in the present findings. Frontier analyses identified countries such as Greece, Chile, Slovenia, and Poland as having the largest potential for improvement. Conversely, Germany, Switzerland and Monaco still exhibited high effective differences despite high development indices, indicating untapped opportunities for interventions. Bayesian age-period-cohort model predicted that the age-standardized DALY rate will decline by 17.24% by 2032, falling to around 86 per 100000. This prediction is concordant with independent projections that the age-standardized smoking-related LBP rate among women will decline to approximately 47 per 100000 by 2036, whereas men will remain higher at 122 per 100000^9^. Together, these forecasts suggest a sustained downward trend but also warn that gender disparities will persist unless cessation efforts specifically target men.

### Limitations

Several limitations inherent in this analysis should be considered. First, the estimates rely on secondary data from the GBD 2021 study; while the GBD uses comprehensive modeling, country-level data are uneven and some regions lack robust surveillance, potentially affecting accuracy^4^. Second, this study analyzed aggregated data rather than individual-level records, which precludes adjustment for confounders such as obesity, psychosocial stress, occupational ergonomics and co-morbidities. Third, the restriction of the sample to women of childbearing age limits the extrapolation of these results to men and to women over 50 years; sex-specific biological differences and health behaviors could modify the association. Fourth, the study classification of smoking did not distinguish between cigarettes, cigars, e-cigarettes or smokeless tobacco, each of which may carry different risks and patterns of use. Fifth, the analysis assumed a constant effect of smoking over time; yet the chemical composition of cigarettes and the rising popularity of vaping may alter risk. Finally, the forecasting model relies on an extrapolation of past trends and does not incorporate potential future demographic changes, emerging nicotine products, or societal shifts.

Future research should address these limitations by integrating individual-level cohort data, detailed smoking histories and co-exposures. Longitudinal studies capturing smoking intensity, duration, and cessation alongside body mass index, occupation, physical activity, and reproductive factors will clarify causal pathways. Genetic and epigenetic studies can elucidate susceptibility, complementing Mendelian randomization evidence that smoking initiation has a causal effect on LBP risk^28^. Randomized controlled trials of gender-sensitive cessation interventions among women of reproductive age are needed, particularly in low and high-middle SDI regions where rates remain high. Finally, researchers should monitor the health effects of emerging nicotine products such as electronic cigarettes and heated tobacco devices, and examine interactions between smoking, obesity, depression, and occupational exposures, all of which contribute substantially to LBP^4,28^. By closing data gaps and implementing targeted interventions, future work can accelerate the decline in smoking-related low-back pain and ensure that all regions and populations benefit.

## Conclusions

While the global burden of smoking-related LBP among women of childbearing age has declined in age-standardized terms, the absolute number of affected women remains substantial, and profound inequities persist. The shifting burden towards low-SDI regions and older reproductive ages.
