# Supplementary material for: Global burden and trends of smoking-related low back pain in women of childbearing age and projections to 2032: A secondary dataset analysis of Global Burden of Disease 2021
Source: Tob Prev Cessat. 2026 Oct 8;12:10.18332/tpc/218955. doi: 10.18332/tpc/218955 (PMC13660435; doi:10.18332/tpc/218955)
Supplement: Supplementary file 1 [file TPC-12-59-s001.pdf]

TOBACCO PREVENTION AND CESSATION

Supplementary file

© 2026 6 Xu Q. et al.

DOI:
10.18332/tpc/218955

The content has been provided by the author(s) and has not been reviewed, verified, or endorsed by European Publishing. It may not have undergone peer review. The views, opinions, and recommendations expressed are solely those of the author(s) and do not necessarily reflect the position of European Publishing. European Publishing accepts no responsibility or liability for any consequences arising from the use of, or reliance on, this content.

Supplementary file figure captions

Fig. S1 Temporal trend of Smoking-Related Low Back Pain burden in WCBA by age pattern in different regions. DALYs cases of 4 age groups (15-49 years, 5-year intervals) from 1990 to 2021 globally and in 5 territories (low to high SDI).

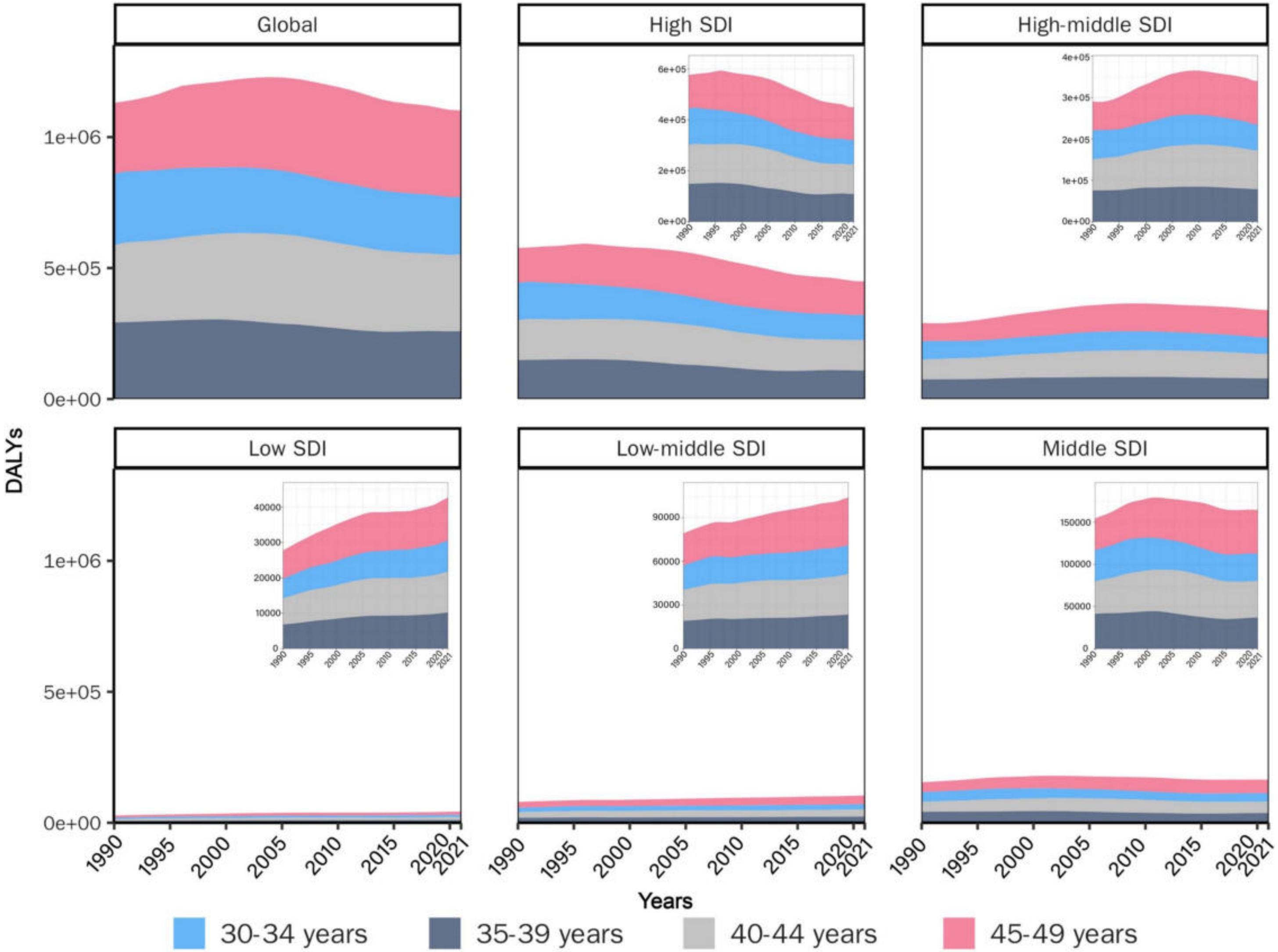

Fig. S2 Temporal trend of Smoking-Related Low Back Pain burden in WCBA by age pattern in different regions. Percentage change in DALYs cases of 7 age groups globally and in 5 territories in 1990 and 2021. Temporal trend of Smoking-Related Low Back Pain burden in WCBA by age pattern in different regions. EAPC of DALYs rates of 7 age groups globally and in 5 territories from 1990 to 2021. WCBA=Women of Childbearing Age, EAPC=Estimated Annual Percentage Change, SDI=Socio-Demographic Index, GBD=Global Burden of Disease.

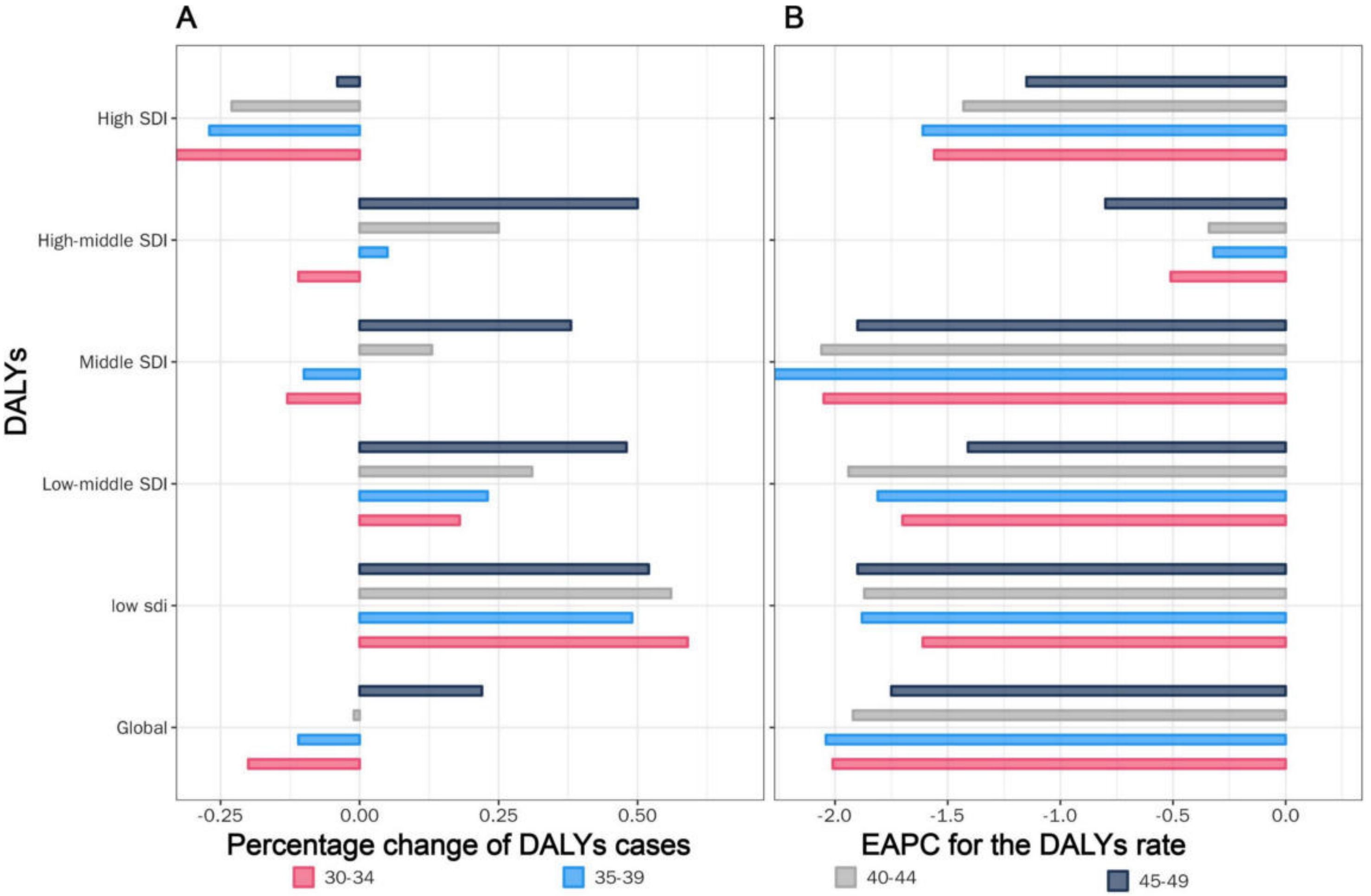

Fig. S3 Temporal trend of Smoking-Related Low Back Pain burden in WCBA by age pattern in different regions. The distribution of DALYs cases across 4 age groups as percentages globally, in 5 territories, and 21 GBD regions in 1990 and 2021.

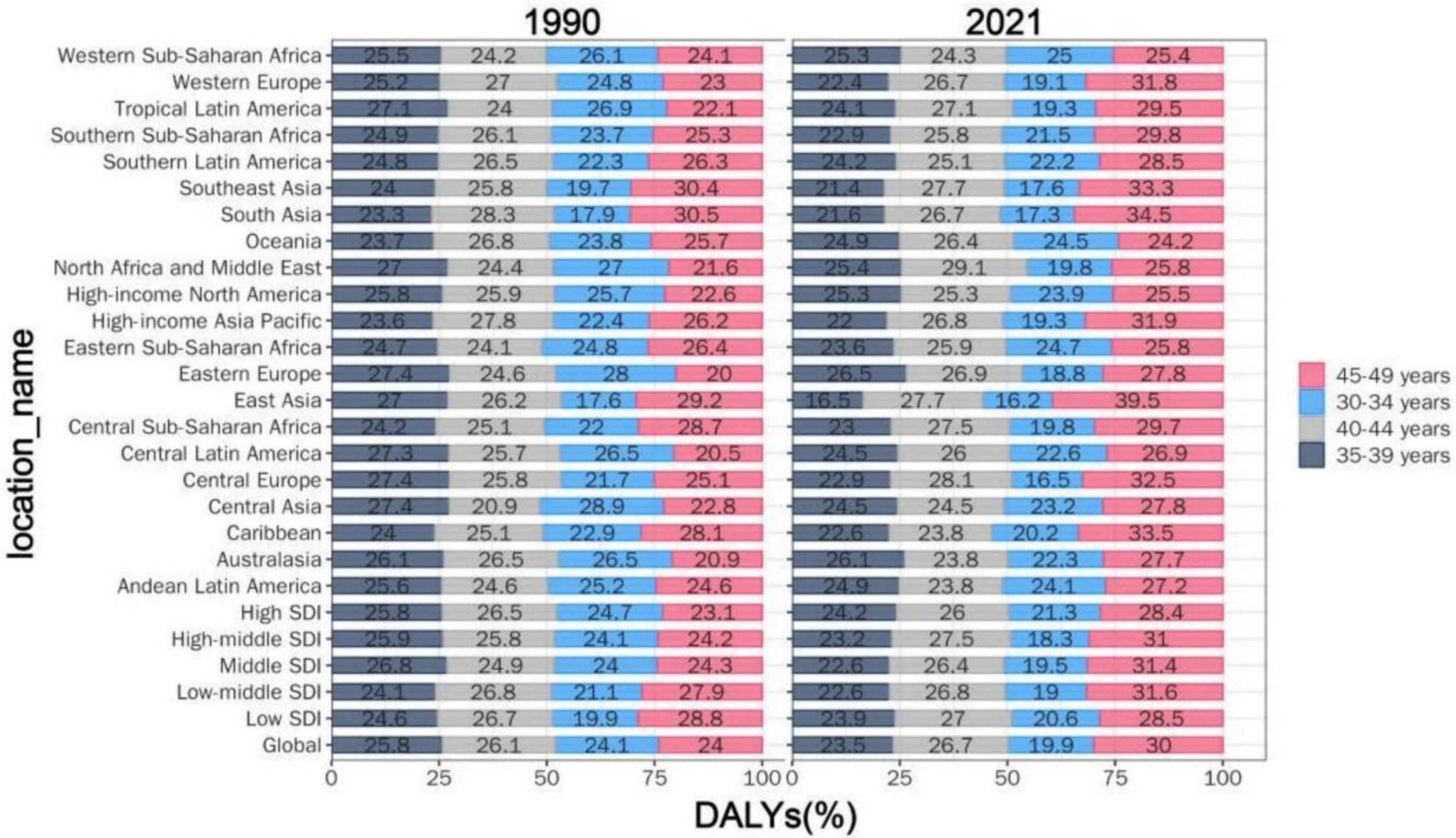

Fig. S4 The associations between the SDI and DALYs rates per 100,000 population of Smoking-Related Low Back Pain in WCBA across 21 GBD regions. WCBA=Women of Childbearing Age, SDI=Socio-Demographic Index, GBD=Global Burden of Disease

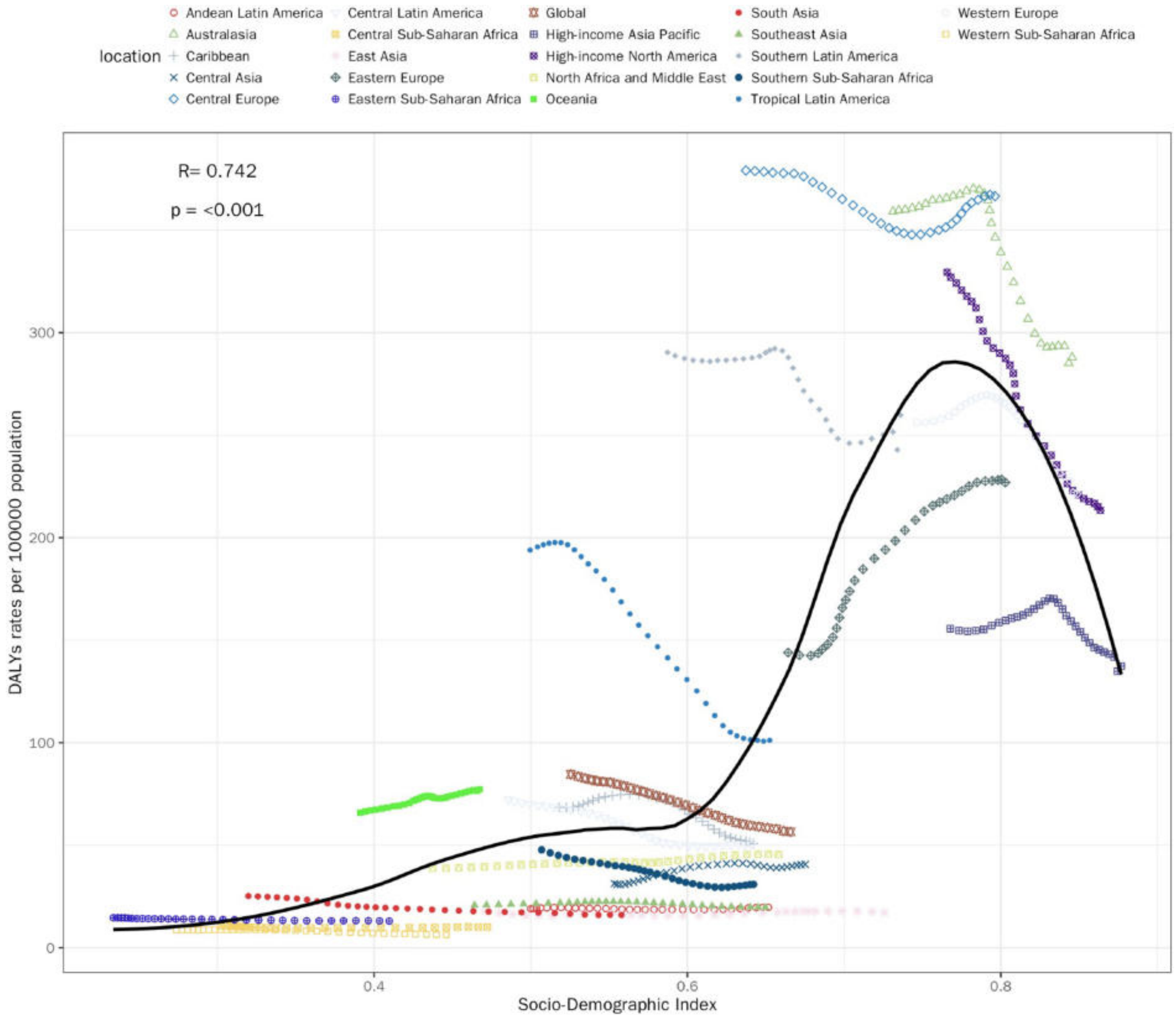

Fig. S5 The frontier analysis results. (A) The frontier analysis based on ASDR and SDI from 1990 to 2021 is illustrated. The color gradient ranges from light blue (representing 1990) to dark blue (representing 2021). The boundaries are outlined with solid black lines. (B) The frontier analysis for 2021 based on ASDR and SDI is depicted. The boundaries are represented by solid black lines, and countries and regions are marked with dots. The top 15 countries and regions with the largest effective differences are highlighted in black. Border countries with low SDI and low effective variance are marked in green, while those with high SDI and relatively high effective variance are marked in yellow. Yellow dots indicate a decrease, and blue dots indicate an increase. ASDR, age-standardized DALYs rate; SDI, sociodemographic index.

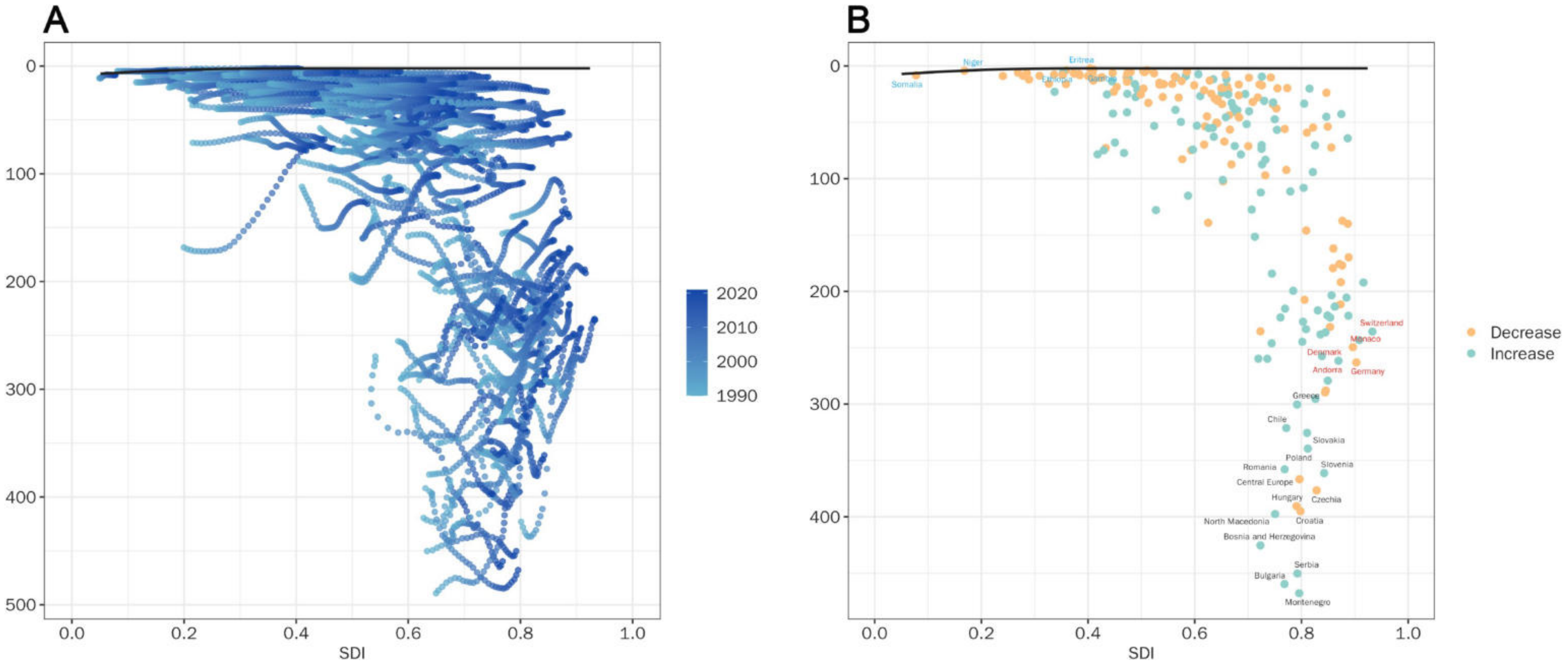

Fig. S6 Analyzing the observed and predicted trends of ASDR of LBP attributable to smoking globally using the BAPC model. ASDR, age-standardized DALYs rate; LBP, low back pain; BAPC, Bayesian age-period-cohort.

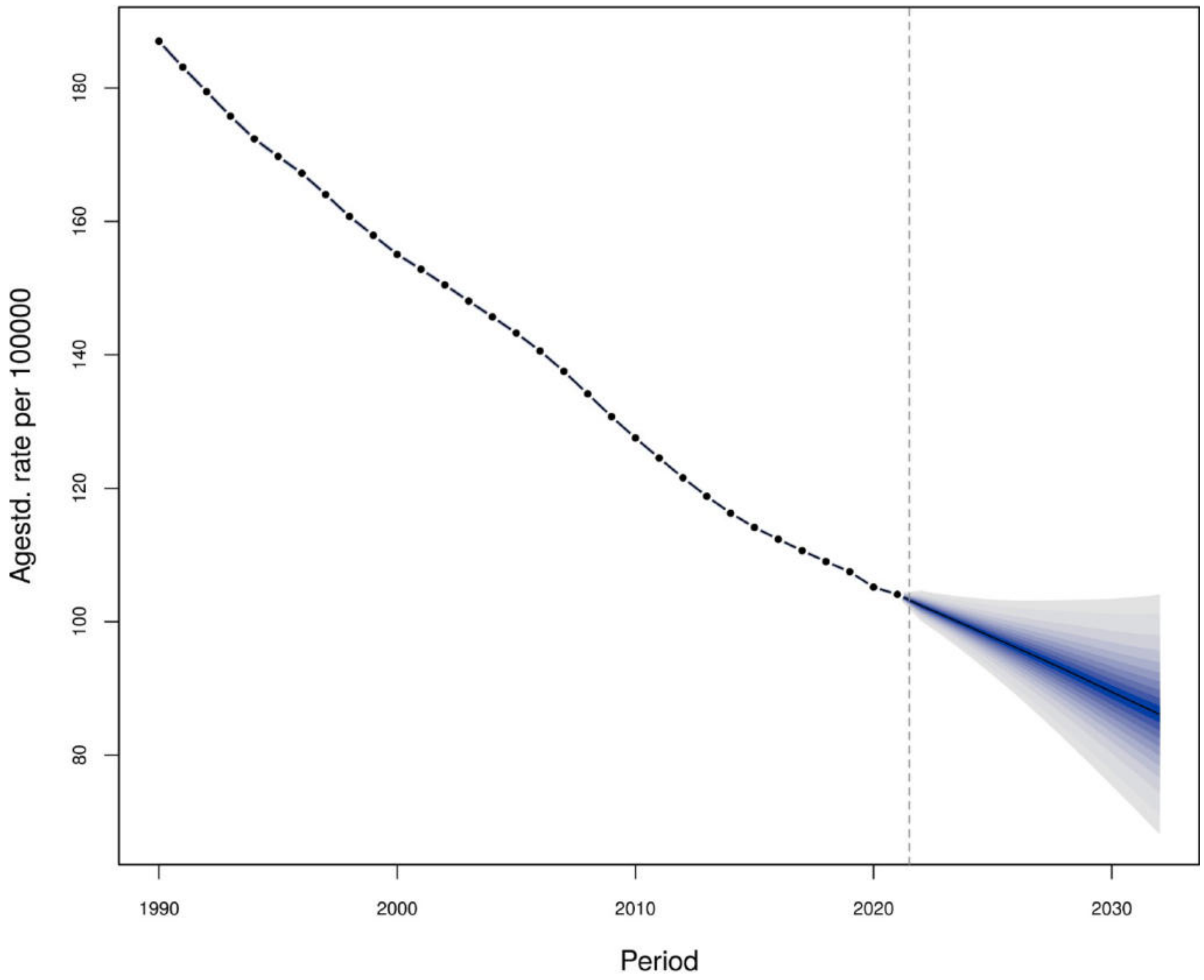

Table S1 The DALYs of Smoking-Related Low Back Pain cases and rates among WCBA in 1990 and 2021 across 204 countries, and the trends from 1990 to 2021.

Location	DALYs cases			DALYs rates		
	1990_(95% UI)	2021_(95% UI)	ercentage change(100%	1990_per 100 000(95% UI)	2021_per 100 000(95% UI)	EAPC(95% CI)
Afghanistan	330.16 (158.9 ~ 579.17)	1652.45 (784.96 ~ 2931.79)	4	15.01 (7.22 ~ 26.32)	22.97 (10.91 ~ 40.75)	2.73 (2.25 ~ 3.22)
Albania	469.74 (224.95 ~ 858.52)	782.53 (395.24 ~ 1322.53)	0.67	56.25 (26.94 ~ 102.8)	127.51 (64.4 ~ 215.5)	3.28 (2.95 ~ 3.61)
Algeria	638.16 (307.04 ~ 1184.22)	1326.47 (573.75 ~ 2473.74)	1.08	10.98 (5.28 ~ 20.38)	11.82 (5.11 ~ 22.04)	0.06 (-0.09 ~ 0.2)
American Samoa	10.77 (5.74 ~ 17.88)	13.18 (6.87 ~ 21.78)	0.22	89.57 (47.78 ~ 148.78)	112.31 (58.56 ~ 185.63)	0.64 (0.41 ~ 0.87)
Andorra	35.89 (19.7 ~ 57.77)	52.29 (28.21 ~ 85.67)	0.46	240.49 (131.99 ~ 387.1)	261.6 (141.12 ~ 428.61)	0.39 (0.27 ~ 0.52)
Angola	433.92 (202.07 ~ 759.3)	1430.21 (670.28 ~ 2518.75)	2.3	18.85 (8.78 ~ 32.98)	18.63 (8.73 ~ 32.82)	0.07 (-0.09 ~ 0.23)
Antigua and Barbuda	6.2 (2.99 ~ 10.89)	11.44 (5.76 ~ 19.85)	0.85	38.15 (18.38 ~ 67.01)	47.36 (23.82 ~ 82.16)	0.55 (0.44 ~ 0.66)
Argentina	23252.86 (12851.62 ~ 35495.9)	27986.41 (15552.1 ~ 45091.39)	0.2	289.69 (160.11 ~ 442.22)	235.45 (130.84 ~ 379.36)	-0.91 (-1.02 ~ -0.8)
Armenia	270.26 (130.38 ~ 475.99)	296.48 (143.87 ~ 512.6)	0.1	31.2 (15.05 ~ 54.96)	40.24 (19.53 ~ 69.57)	0.42 (0.22 ~ 0.61)
Australia	15916.59 (9203.26 ~ 25183.75)	17447.56 (9259.79 ~ 28029.92)	0.1	356.59 (206.19 ~ 564.21)	289.62 (153.71 ~ 465.28)	-0.82 (-0.99 ~ -0.66)
Austria	4210.01 (2393.87 ~ 6325.95)	4407.19 (2451.64 ~ 6964.72)	0.05	212.75 (120.97 ~ 319.67)	223.2 (124.16 ~ 352.73)	0.16 (-0.03 ~ 0.34)
Azerbaijan	272.34 (132.35 ~ 494.75)	685.97 (328.85 ~ 1192.43)	1.52	14.52 (7.06 ~ 26.38)	25.03 (12 ~ 43.5)	1.9 (1.45 ~ 2.36)
Bahamas	21.56 (10.64 ~ 37.58)	36.14 (17.49 ~ 65.58)	0.68	29.73 (14.67 ~ 51.81)	33.52 (16.22 ~ 60.83)	0.35 (0.17 ~ 0.54)
Bahrain	45.01 (21.93 ~ 80.34)	123.16 (52.09 ~ 226.44)	1.74	39.04 (19.02 ~ 69.68)	37.78 (15.98 ~ 69.45)	-0.12 (-0.23 ~ -0.02)
Bangladesh	3518.81 (1714.97 ~ 6251.1)	9633.14 (4873.17 ~ 16453.79)	1.74	14.29 (6.97 ~ 25.39)	20.94 (10.59 ~ 35.77)	1.78 (1.36 ~ 2.2)
Barbados	24.6 (11.98 ~ 41.62)	24.44 (12.33 ~ 43.09)	-0.01	35.91 (17.48 ~ 60.75)	34.33 (17.33 ~ 60.53)	-0.48 (-0.75 ~ -0.21)
Belarus	4866.48 (2603.14 ~ 7977.21)	4230.34 (2282.07 ~ 7032.21)	-0.13	192.07 (102.74 ~ 314.84)	199.48 (107.61 ~ 331.6)	-0.06 (-0.12 ~ 0)
Belgium	6585.43 (3725.34 ~ 10275.85)	5736.36 (3233.57 ~ 9270.88)	-0.13	270.51 (153.03 ~ 422.1)	231.53 (130.51 ~ 374.19)	-0.75 (-0.9 ~ -0.6)
Belize	9.6 (4.69 ~ 16.94)	32.37 (16.4 ~ 55.83)	2.37	22.81 (11.15 ~ 40.25)	26.8 (13.57 ~ 46.22)	0.68 (0.59 ~ 0.77)
Benin	145.31 (69.2 ~ 270.44)	220.67 (106.08 ~ 393.94)	0.52	13.27 (6.32 ~ 24.69)	6.8 (3.27 ~ 12.14)	-2.63 (-3.11 ~ -2.14)
Bermuda	12.92 (6.69 ~ 22.78)	12.7 (6.5 ~ 21.53)	-0.02	75.51 (39.1 ~ 133.19)	94.22 (48.2 ~ 159.67)	0.58 (0.44 ~ 0.71)
Bhutan	52.09 (25.19 ~ 96.89)	85.38 (39.29 ~ 153.6)	0.64	36.39 (17.59 ~ 67.68)	41.24 (18.98 ~ 74.19)	0.11 (-0.04 ~ 0.25)
Bolivia (Plurinational State of)	287.93 (141.24 ~ 492.07)	538.02 (276.34 ~ 884.07)	0.87	18.82 (9.23 ~ 32.16)	17.26 (8.86 ~ 28.35)	-0.07 (-0.28 ~ 0.14)
Bosnia and Herzegovina	3138.84 (1768.95 ~ 4884.14)	3111.58 (1792.02 ~ 4859.82)	-0.01	269.6 (151.94 ~ 419.51)	425.31 (244.94 ~ 664.27)	1.45 (1.32 ~ 1.58)
Botswana	99.85 (47.35 ~ 171.46)	199.4 (98.27 ~ 349.28)	1	31.01 (14.71 ~ 53.26)	29.31 (14.44 ~ 51.34)	-0.33 (-0.44 ~ -0.22)
Brazil	76635.78 (42190.15 ~ 119724.5)	60144.99 (32051.07 ~ 97676.84)	-0.22	196.75 (108.32 ~ 307.37)	102.43 (54.59 ~ 166.36)	-2.69 (-2.92 ~ -2.46)
Brunei Darussalam	48.68 (22.85 ~ 86.2)	73.99 (35.56 ~ 132.36)	0.52	72.06 (33.82 ~ 127.59)	59.22 (28.46 ~ 105.94)	-0.65 (-0.78 ~ -0.51)
Bulgaria	9322.41 (5365.71 ~ 14165.72)	6546.75 (3827.03 ~ 10074.33)	-0.3	450.17 (259.11 ~ 684.05)	459.59 (268.66 ~ 707.24)	0.12 (0.02 ~ 0.23)
Burkina Faso	183.38 (91.52 ~ 316.54)	353.71 (178.07 ~ 655.62)	0.93	8.76 (4.37 ~ 15.12)	6.44 (3.24 ~ 11.94)	-1.36 (-1.63 ~ -1.1)
Burundi	292.19 (141.23 ~ 508.81)	377.99 (182.78 ~ 665.06)	0.29	23.2 (11.21 ~ 40.4)	12.09 (5.84 ~ 21.26)	-2.04 (-2.3 ~ -1.77)
Cabo Verde	14.39 (7.07 ~ 25.87)	22.55 (10.74 ~ 38.44)	0.57	18.15 (8.92 ~ 32.61)	15 (7.14 ~ 25.58)	-1.19 (-1.39 ~ -0.99)
Cambodia	492.44 (242.73 ~ 880.65)	1122.24 (534.7 ~ 2117.69)	1.28	19.66 (9.69 ~ 35.16)	24.84 (11.83 ~ 46.87)	0.31 (0.04 ~ 0.58)

Table S1 The DALYs of Smoking-Related Low Back Pain cases and rates among WCBA in 1990 and 2021 across 204 countries, and the trends from 1990 to 2021.

Location	DALYs cases			DALYs rates		
	1990_(95% UI)	2021_(95% UI)	ercentage change(100%	1990_per 100 000(95% UI)	2021_per 100 000(95% UI)	EAPC(95% CI)
Cameroon	183.94 (90.47 ~ 327.1)	422.09 (203.33 ~ 760.78)	1.29	7.73 (3.8 ~ 13.75)	5.39 (2.6 ~ 9.71)	-1.28 (-1.42 ~ -1.14)
Canada	23596.57 (13496.28 ~ 36682.6)	17539.44 (9803.04 ~ 28514.64)	-0.26	320.77 (183.47 ~ 498.67)	211.47 (118.2 ~ 343.8)	-1.65 (-1.79 ~ -1.51)
Central African Republic	72.3 (32.62 ~ 130.71)	121.89 (59.08 ~ 220.15)	0.69	11.28 (5.09 ~ 20.39)	8.81 (4.27 ~ 15.91)	-0.92 (-1.16 ~ -0.68)
Chad	202.19 (95.21 ~ 389.1)	348.84 (170.65 ~ 625.36)	0.73	15.21 (7.16 ~ 29.26)	9.02 (4.41 ~ 16.17)	-1.8 (-2.02 ~ -1.57)
Chile	10818.02 (6292.17 ~ 17140.84)	15129.03 (8622.21 ~ 23631.47)	0.4	299.29 (174.08 ~ 474.22)	321.2 (183.06 ~ 501.71)	-0.02 (-0.19 ~ 0.15)
China	55251.42 (27873.33 ~ 93861.72)	52787.97 (24755.34 ~ 95991.71)	-0.04	17.15 (8.65 ~ 29.13)	16.56 (7.77 ~ 30.12)	0.39 (0.22 ~ 0.56)
Colombia	6895.72 (3508.15 ~ 11585.79)	8795.97 (4623.38 ~ 15032.75)	0.28	79.4 (40.39 ~ 133.4)	67.14 (35.29 ~ 114.75)	-0.67 (-0.83 ~ -0.51)
Comoros	11.46 (5.44 ~ 20.25)	22.39 (10.63 ~ 41.2)	0.95	10.88 (5.17 ~ 19.22)	11.47 (5.45 ~ 21.11)	0.16 (0.11 ~ 0.22)
Congo	43.69 (21.97 ~ 75.53)	131.56 (59.47 ~ 246.39)	2.01	7.75 (3.9 ~ 13.4)	9.18 (4.15 ~ 17.2)	0.64 (0.52 ~ 0.76)
Cook Islands	5 (2.63 ~ 8.09)	4.8 (2.6 ~ 7.75)	-0.04	108.91 (57.29 ~ 176.01)	111.36 (60.45 ~ 180.01)	0.02 (-0.27 ~ 0.3)
Costa Rica	646.17 (342.02 ~ 1060.63)	921.56 (476.99 ~ 1596.27)	0.43	83.09 (43.98 ~ 136.39)	71.19 (36.85 ~ 123.31)	-0.76 (-0.94 ~ -0.57)
Côte d'Ivoire	343.84 (159.46 ~ 616.7)	667.6 (320.6 ~ 1227.59)	0.94	12.57 (5.83 ~ 22.54)	10.03 (4.81 ~ 18.44)	-1.36 (-1.67 ~ -1.06)
Croatia	4976.58 (2875.69 ~ 7668.78)	3543.83 (2039.13 ~ 5662.51)	-0.29	414.47 (239.5 ~ 638.69)	395.05 (227.32 ~ 631.24)	-0.02 (-0.09 ~ 0.06)
Cuba	3211.64 (1726.97 ~ 5425.38)	2175.42 (1183.42 ~ 3513.12)	-0.32	104.81 (56.36 ~ 177.06)	87.41 (47.55 ~ 141.15)	-0.73 (-1.12 ~ -0.34)
Cyprus	408.89 (221.46 ~ 680.68)	850.7 (476.92 ~ 1367.88)	1.08	207 (112.12 ~ 344.6)	238.21 (133.55 ~ 383.03)	0.31 (0.15 ~ 0.47)
Czechia	10999.48 (6040.24 ~ 17084.11)	8637.4 (4706.18 ~ 13805.6)	-0.21	427.24 (234.61 ~ 663.57)	376.54 (205.16 ~ 601.84)	-0.55 (-0.72 ~ -0.37)
Democratic People's Republic of Korea	1368.5 (645.86 ~ 2573.31)	1851.67 (809.43 ~ 3406.6)	0.35	24.13 (11.39 ~ 45.38)	28.1 (12.28 ~ 51.69)	0.56 (0.45 ~ 0.67)
Democratic Republic of the Congo	757.13 (372.83 ~ 1357.38)	1535.88 (735.16 ~ 2794.39)	1.03	8.87 (4.37 ~ 15.9)	7.21 (3.45 ~ 13.11)	-0.77 (-0.84 ~ -0.7)
Denmark	5522.23 (3292.2 ~ 8235.18)	3151.24 (1683.76 ~ 5199.29)	-0.43	422.94 (252.14 ~ 630.72)	249.49 (133.31 ~ 411.64)	-2.26 (-2.47 ~ -2.06)
Djibouti	15.78 (7.4 ~ 30.08)	52.01 (24.52 ~ 94.83)	2.3	16.05 (7.53 ~ 30.6)	16.19 (7.63 ~ 29.52)	0.16 (-0.03 ~ 0.36)
Dominica	5.41 (2.61 ~ 9.4)	5.34 (2.51 ~ 9.28)	-0.01	31.76 (15.31 ~ 55.19)	32.47 (15.27 ~ 56.45)	-0.04 (-0.11 ~ 0.03)
Dominican Republic	1092.3 (585.35 ~ 1832.32)	1555.19 (810.55 ~ 2653.45)	0.42	57.86 (31 ~ 97.06)	53.69 (27.98 ~ 91.6)	-0.65 (-0.99 ~ -0.31)
Ecuador	656.38 (348.88 ~ 1141.86)	872.38 (448.79 ~ 1517.42)	0.33	26.03 (13.83 ~ 45.27)	18.5 (9.52 ~ 32.18)	-1.14 (-1.26 ~ -1.02)
Egypt	961.73 (442.47 ~ 1771.39)	1914.08 (904.95 ~ 3492.78)	0.99	7.33 (3.37 ~ 13.51)	7.38 (3.49 ~ 13.46)	-0.17 (-0.29 ~ -0.04)
El Salvador	387.89 (186.78 ~ 657.72)	693.17 (331.65 ~ 1213.82)	0.79	29.59 (14.25 ~ 50.18)	39.02 (18.67 ~ 68.33)	1.06 (0.9 ~ 1.21)
Equatorial Guinea	8.85 (4.09 ~ 15.31)	28.24 (13.02 ~ 51.55)	2.19	8.96 (4.14 ~ 15.51)	7.75 (3.57 ~ 14.14)	-0.5 (-0.62 ~ -0.38)
Eritrea	20.72 (9.93 ~ 38.9)	37.98 (17.58 ~ 71.94)	0.83	2.63 (1.26 ~ 4.93)	2.29 (1.06 ~ 4.35)	-0.51 (-0.65 ~ -0.36)
Estonia	896.6 (499.52 ~ 1384.11)	658.02 (366.79 ~ 1035.6)	-0.27	235.3 (131.09 ~ 363.24)	236.39 (131.76 ~ 372.03)	0.06 (-0.09 ~ 0.21)
Eswatini	14.55 (7.14 ~ 26.56)	16.4 (7.98 ~ 31.1)	0.13	7.46 (3.66 ~ 13.63)	5.2 (2.53 ~ 9.87)	-1.66 (-1.95 ~ -1.37)
Ethiopia	525.96 (253.42 ~ 1020)	1115.58 (520.52 ~ 1966.79)	1.12	4.66 (2.25 ~ 9.04)	4.02 (1.88 ~ 7.1)	-0.3 (-0.58 ~ -0.01)
Fiji	131.52 (73.12 ~ 213.51)	162.41 (85.2 ~ 264.96)	0.23	67.28 (37.4 ~ 109.21)	71.11 (37.3 ~ 116.01)	0.23 (0.13 ~ 0.33)
Finland	2927.57 (1689.1 ~ 4475.31)	1841.21 (1006.06 ~ 2948.39)	-0.37	231.74 (133.7 ~ 354.25)	162 (88.52 ~ 259.42)	-1.29 (-1.36 ~ -1.23)

Table S1 The DALYs of Smoking-Related Low Back Pain cases and rates among WCBA in 1990 and 2021 across 204 countries, and the trends from 1990 to 2021.

Location	DALYs cases			DALYs rates		
	1990_(95% UI)	2021_(95% UI)	ercentage change(100%	1990_per 100 000(95% UI)	2021_per 100 000(95% UI)	EAPC(95% CI)
France	33587.68 (19403.61 ~ 51711.76)	36536.94 (20860.78 ~ 56469.15)	0.09	232.09 (134.08 ~ 357.32)	257.35 (146.93 ~ 397.74)	0.35 (0.23 ~ 0.47)
Gabon	20.88 (10.27 ~ 38.84)	58.02 (27.58 ~ 104.31)	1.78	9.43 (4.64 ~ 17.55)	11.85 (5.63 ~ 21.31)	0.76 (0.65 ~ 0.87)
Gambia	10.2 (5.14 ~ 17.9)	18.88 (8.79 ~ 33.79)	0.85	4.49 (2.27 ~ 7.89)	3.07 (1.43 ~ 5.49)	-1.42 (-1.54 ~ -1.3)
Georgia	784.36 (387.43 ~ 1400.13)	654.98 (317.7 ~ 1131.05)	-0.16	57.14 (28.23 ~ 102)	83.23 (40.37 ~ 143.72)	1.35 (1.3 ~ 1.4)
Germany	60563.73 (34862.99 ~ 94038.41)	44792.88 (25158.94 ~ 71335.88)	-0.26	311.62 (179.38 ~ 483.86)	263.01 (147.73 ~ 418.87)	-0.7 (-0.89 ~ -0.51)
Ghana	309.78 (141.19 ~ 561.93)	920.17 (414.6 ~ 1656.37)	1.97	8.78 (4 ~ 15.93)	10.08 (4.54 ~ 18.14)	0.93 (0.77 ~ 1.09)
Greece	6040.59 (3355.46 ~ 9125.79)	6510.18 (3673.36 ~ 10123.55)	0.08	239.73 (133.17 ~ 362.17)	300.35 (169.47 ~ 467.05)	0.85 (0.66 ~ 1.05)
Greenland	43.06 (24.49 ~ 65.23)	37.6 (21.17 ~ 57.67)	-0.13	292.34 (166.24 ~ 442.78)	295.4 (166.31 ~ 453.08)	-0.31 (-0.64 ~ 0.02)
Grenada	6.3 (2.99 ~ 10.5)	8.41 (4.21 ~ 14.68)	0.33	32.38 (15.34 ~ 53.95)	32.85 (16.45 ~ 57.31)	-0.11 (-0.16 ~ -0.06)
Guam	30.74 (16.28 ~ 51.07)	39.03 (20.62 ~ 61.3)	0.27	88.11 (46.66 ~ 146.36)	108.18 (57.16 ~ 169.91)	0.61 (0.47 ~ 0.75)
Guatemala	645.08 (346.63 ~ 1091.23)	1213.53 (616.29 ~ 2073.23)	0.88	35.11 (18.87 ~ 59.4)	27.61 (14.02 ~ 47.17)	-0.84 (-0.87 ~ -0.82)
Guinea	125.51 (59.76 ~ 217.8)	253.93 (118.7 ~ 460.13)	1.02	9.2 (4.38 ~ 15.97)	7.65 (3.58 ~ 13.87)	-0.79 (-0.86 ~ -0.72)
Guinea-Bissau	13.64 (6.89 ~ 23.88)	31.94 (15.23 ~ 56.33)	1.34	5.85 (2.95 ~ 10.24)	6.08 (2.9 ~ 10.72)	0.21 (-0.03 ~ 0.45)
Guyana	50.44 (24.54 ~ 86.72)	49.02 (24.44 ~ 86.25)	-0.03	24.69 (12.02 ~ 42.46)	24.12 (12.03 ~ 42.45)	-0.17 (-0.34 ~ 0)
Haiti	495.14 (253.67 ~ 849.05)	811.75 (401.79 ~ 1336.65)	0.64	32.18 (16.49 ~ 55.18)	22.93 (11.35 ~ 37.76)	-1.49 (-1.72 ~ -1.26)
Honduras	379.68 (186.51 ~ 667.03)	930.49 (437.12 ~ 1626.72)	1.45	35.99 (17.68 ~ 63.22)	32.78 (15.4 ~ 57.31)	-0.46 (-0.6 ~ -0.31)
Hungary	12402.09 (7302.08 ~ 18842.21)	8352.93 (4586.28 ~ 13384.5)	-0.33	489.29 (288.08 ~ 743.36)	390.53 (214.43 ~ 625.78)	-0.8 (-0.88 ~ -0.71)
Iceland	153.32 (84.09 ~ 236.36)	140.97 (77.44 ~ 232.41)	-0.08	236.39 (129.66 ~ 364.42)	177.03 (97.25 ~ 291.85)	-1.4 (-1.55 ~ -1.26)
India	45983.71 (22817.99 ~ 77477.69)	50742.65 (23697.47 ~ 97135.06)	0.1	22.77 (11.3 ~ 38.37)	13.41 (6.26 ~ 25.68)	-1.78 (-1.92 ~ -1.63)
Indonesia	6754.69 (3252.86 ~ 11736.17)	14141.35 (6775.87 ~ 25733.61)	1.09	14.09 (6.79 ~ 24.49)	18.78 (9 ~ 34.18)	1.15 (0.92 ~ 1.38)
Iran (Islamic Republic of)	4349.13 (2203.52 ~ 7575.2)	12019.5 (5815.59 ~ 21667.29)	1.76	34.39 (17.42 ~ 59.9)	51.71 (25.02 ~ 93.21)	1.35 (1.18 ~ 1.52)
Iraq	1051.66 (512.3 ~ 1871.3)	2282.27 (1114.21 ~ 3885.6)	1.17	25.92 (12.63 ~ 46.12)	21.65 (10.57 ~ 36.86)	-0.66 (-0.77 ~ -0.55)
Ireland	2141.81 (1210.46 ~ 3319.45)	2238.63 (1169.97 ~ 3608.43)	0.05	242.55 (137.08 ~ 375.91)	191.88 (100.28 ~ 309.28)	-0.77 (-0.89 ~ -0.65)
Israel	2732.76 (1509.08 ~ 4282.47)	3247.74 (1750.27 ~ 5287.01)	0.19	224.02 (123.71 ~ 351.05)	146 (78.68 ~ 237.68)	-1.57 (-1.64 ~ -1.5)
Italy	34195.15 (19656.97 ~ 53075.66)	25177.43 (13944.04 ~ 40555.74)	-0.26	239.25 (137.53 ~ 371.34)	207.51 (114.92 ~ 334.25)	-0.32 (-0.44 ~ -0.21)
Jamaica	293.3 (154.84 ~ 486.82)	353.99 (181.46 ~ 626.9)	0.21	49.23 (25.99 ~ 81.7)	45.85 (23.5 ~ 81.19)	-0.74 (-0.98 ~ -0.49)
Japan	62056.42 (34047.41 ~ 100217.28)	43692.33 (22036.06 ~ 72171.25)	-0.3	193.3 (106.06 ~ 312.17)	175.71 (88.62 ~ 290.24)	-0.23 (-0.45 ~ 0)
Jordan	512.67 (250.24 ~ 859.31)	2178.78 (1184.55 ~ 3651.6)	3.25	61.48 (30.01 ~ 103.04)	70.31 (38.22 ~ 117.83)	0.94 (0.71 ~ 1.17)
Kazakhstan	2210.7 (1148.47 ~ 3656.04)	3482.57 (1757.32 ~ 6001.22)	0.58	53.72 (27.91 ~ 88.85)	73.54 (37.11 ~ 126.72)	1.11 (0.88 ~ 1.33)
Kenya	492.38 (216.17 ~ 879.03)	881.22 (434.14 ~ 1548.11)	0.79	9.51 (4.17 ~ 16.97)	6.64 (3.27 ~ 11.66)	-1.37 (-1.66 ~ -1.09)
Kiribati	20.95 (11.73 ~ 32.36)	40.9 (23.1 ~ 66.12)	0.95	112.09 (62.73 ~ 173.13)	127.91 (72.24 ~ 206.82)	0.05 (-0.28 ~ 0.38)
Kuwait	140.57 (68.05 ~ 265.5)	660.14 (319.77 ~ 1155.45)	3.7	33.92 (16.42 ~ 64.06)	45.16 (21.88 ~ 79.05)	0.79 (0.64 ~ 0.94)

Table S1 The DALYs of Smoking-Related Low Back Pain cases and rates among WCBA in 1990 and 2021 across 204 countries, and the trends from 1990 to 2021.

Location	DALYs cases			DALYs rates		
	1990_(95% UI)	2021_(95% UI)	ercentage change(100%	1990_per 100 000(95% UI)	2021_per 100 000(95% UI)	EAPC(95% CI)
Kyrgyzstan	287.17 (137.71 ~ 515.47)	914.49 (467.87 ~ 1597.9)	2.18	27.4 (13.14 ~ 49.18)	53.04 (27.14 ~ 92.68)	2.74 (2.45 ~ 3.03)
Lao People's Democratic Republic	201.49 (92.37 ~ 360.08)	550.22 (276.47 ~ 943.07)	1.73	20.82 (9.54 ~ 37.21)	27.73 (13.93 ~ 47.53)	1.12 (0.95 ~ 1.29)
Latvia	1355.66 (724.04 ~ 2205.55)	845.16 (463.18 ~ 1383.51)	-0.38	209.18 (111.72 ~ 340.31)	216.88 (118.86 ~ 355.04)	0.31 (0.22 ~ 0.41)
Lebanon	985.54 (530.49 ~ 1646.8)	2733.21 (1475.71 ~ 4381.75)	1.77	131.51 (70.79 ~ 219.76)	184.23 (99.47 ~ 295.35)	0.77 (0.5 ~ 1.05)
Lesotho	18.09 (9.01 ~ 32.92)	19.9 (9.6 ~ 35.73)	0.1	4.78 (2.38 ~ 8.7)	3.93 (1.89 ~ 7.05)	-0.94 (-1.04 ~ -0.83)
Liberia	50.9 (24.73 ~ 87.75)	112.08 (50.19 ~ 196.74)	1.2	9.17 (4.46 ~ 15.82)	8.03 (3.6 ~ 14.1)	-0.94 (-1.11 ~ -0.76)
Libya	63.6 (29.22 ~ 118.79)	189.71 (85.02 ~ 351.2)	1.98	6.98 (3.21 ~ 13.04)	9.54 (4.27 ~ 17.66)	1.41 (1.15 ~ 1.67)
Lithuania	1526.7 (848.26 ~ 2376.93)	1165.94 (647.12 ~ 1897.97)	-0.24	165.6 (92.01 ~ 257.83)	203.51 (112.95 ~ 331.28)	1.02 (0.91 ~ 1.13)
Luxembourg	256.82 (144.82 ~ 404.05)	320.02 (177.59 ~ 506.45)	0.25	264.28 (149.03 ~ 415.79)	205.55 (114.07 ~ 325.3)	-1.14 (-1.34 ~ -0.93)
Madagascar	468.3 (218.49 ~ 829.34)	653.61 (324.16 ~ 1185.71)	0.4	17.39 (8.11 ~ 30.79)	9.02 (4.48 ~ 16.37)	-2.31 (-2.52 ~ -2.1)
Malawi	211.08 (101.79 ~ 376.61)	419.39 (192.05 ~ 761.07)	0.99	9.35 (4.51 ~ 16.68)	8.37 (3.83 ~ 15.19)	-0.35 (-0.6 ~ -0.1)
Malaysia	581.44 (276.82 ~ 1046.44)	864.83 (408.65 ~ 1648.21)	0.49	12.98 (6.18 ~ 23.36)	10.26 (4.85 ~ 19.56)	-1.17 (-1.42 ~ -0.93)
Maldives	10.3 (4.98 ~ 17.69)	27.34 (12.85 ~ 48.06)	1.65	21.64 (10.46 ~ 37.16)	23.73 (11.15 ~ 41.72)	0.05 (-0.12 ~ 0.23)
Mali	92.82 (44.69 ~ 167.28)	324.41 (158.33 ~ 562.28)	2.5	4.84 (2.33 ~ 8.72)	5.96 (2.91 ~ 10.33)	0.83 (0.74 ~ 0.92)
Malta	246.69 (142.59 ~ 396.2)	230.7 (126.84 ~ 374.28)	-0.06	260.12 (150.35 ~ 417.77)	244.71 (134.55 ~ 397.02)	-0.32 (-0.56 ~ -0.09)
Marshall Islands	3.47 (1.63 ~ 6.35)	7.37 (3.78 ~ 13.13)	1.12	35.49 (16.63 ~ 64.96)	49.72 (25.51 ~ 88.55)	1.25 (1.12 ~ 1.38)
Mauritania	148.24 (71.74 ~ 272.62)	271.97 (129.2 ~ 468.33)	0.83	31.54 (15.26 ~ 58)	25.34 (12.04 ~ 43.63)	-0.65 (-0.83 ~ -0.47)
Mauritius	79.62 (38.9 ~ 142.49)	88.45 (44.84 ~ 164.85)	0.11	26.65 (13.02 ~ 47.69)	27.95 (14.17 ~ 52.08)	0.19 (0.07 ~ 0.32)
Mexico	16296.12 (8808.89 ~ 26406.07)	15331.34 (8113.97 ~ 25205.93)	-0.06	74.43 (40.24 ~ 120.61)	43.75 (23.15 ~ 71.93)	-2.2 (-2.49 ~ -1.91)
Micronesia (Federated States of)	21.73 (11.55 ~ 35.52)	30.12 (16.18 ~ 49.38)	0.39	93.71 (49.82 ~ 153.19)	115.18 (61.88 ~ 188.82)	0.7 (0.6 ~ 0.81)
Monaco	20.33 (11.2 ~ 32.25)	17.37 (9.13 ~ 27.97)	-0.15	291.6 (160.73 ~ 462.68)	243.25 (127.86 ~ 391.71)	-0.64 (-0.73 ~ -0.54)
Mongolia	144.57 (69.67 ~ 249.65)	589.55 (292.31 ~ 1002.21)	3.08	28.25 (13.61 ~ 48.78)	69.87 (34.64 ~ 118.77)	3.47 (3.3 ~ 3.63)
Montenegro	600.02 (341.16 ~ 909.25)	673.67 (390.56 ~ 1013.1)	0.12	384.43 (218.58 ~ 582.56)	467.75 (271.18 ~ 703.42)	0.68 (0.55 ~ 0.81)
Morocco	795.3 (346.32 ~ 1416.81)	917.55 (427.74 ~ 1644)	0.15	12.63 (5.5 ~ 22.5)	9.47 (4.42 ~ 16.97)	-1.03 (-1.1 ~ -0.97)
Mozambique	631.58 (309.59 ~ 1139.56)	1199.76 (568.05 ~ 2107.42)	0.9	20.01 (9.81 ~ 36.11)	15.8 (7.48 ~ 27.76)	-0.42 (-0.58 ~ -0.27)
Myanmar	3217.86 (1553.28 ~ 5329.67)	2515.65 (1222.08 ~ 4410.7)	-0.22	30.88 (14.9 ~ 51.14)	16.64 (8.08 ~ 29.18)	-2.28 (-2.49 ~ -2.07)
Namibia	100.23 (52.08 ~ 170.13)	143.01 (75.19 ~ 243.79)	0.43	29.56 (15.36 ~ 50.17)	21.62 (11.37 ~ 36.87)	-1.41 (-1.59 ~ -1.23)
Nauru	3.41 (1.98 ~ 5.29)	3.96 (2.12 ~ 6.37)	0.16	140.08 (81.42 ~ 217.33)	139.14 (74.58 ~ 223.85)	-0.19 (-0.36 ~ -0.01)
Nepal	7690.19 (4133.53 ~ 12243.83)	6605.4 (3359.98 ~ 11464.78)	-0.14	168.37 (90.5 ~ 268.06)	72.82 (37.04 ~ 126.39)	-3.16 (-3.55 ~ -2.77))
Netherlands	10262.59 (5838.25 ~ 15434.5)	6231.46 (3332.38 ~ 9997.21)	-0.39	259.04 (147.36 ~ 389.58)	169.81 (90.81 ~ 272.43)	-1.59 (-1.78 ~ -1.4)
New Zealand	3355.62 (1901.93 ~ 5263.68)	3335.39 (1867.71 ~ 5190)	-0.01	371.22 (210.4 ~ 582.3)	279.17 (156.33 ~ 434.4)	-1.23 (-1.46 ~ -1)
Nicaragua	419.2 (209.03 ~ 730.66)	965.6 (489.01 ~ 1681.33)	1.3	46.46 (23.17 ~ 80.98)	53.26 (26.97 ~ 92.74)	0.66 (0.49 ~ 0.82)

Table S1 The DALYs of Smoking-Related Low Back Pain cases and rates among WCBA in 1990 and 2021 across 204 countries, and the trends from 1990 to 2021.						
Location	DALYs cases			DALYs rates		
	1990_(95% UI)	2021_(95% UI)	ercentage change(100%	1990_per 100 000(95% UI)	2021_per 100 000(95% UI)	EAPC(95% CI)
Niger	84.08 (39.15 ~ 152.56)	227.49 (102.28 ~ 399.41)	1.71	4.83 (2.25 ~ 8.77)	4.27 (1.92 ~ 7.49)	-0.23 (-0.39 ~ -0.08)
Nigeria	1392.23 (696.06 ~ 2460.31)	2732.63 (1292.68 ~ 4825.17)	0.96	6.87 (3.44 ~ 12.15)	4.76 (2.25 ~ 8.4)	-1.14 (-1.43 ~ -0.85)
Niue	0.4 (0.22 ~ 0.68)	0.34 (0.17 ~ 0.57)	-0.15	81.42 (43.46 ~ 137.9)	87.39 (44.22 ~ 149.16)	0.25 (0.16 ~ 0.34)
North Macedonia	1615.11 (951.74 ~ 2551.25)	2109.58 (1223.25 ~ 3203.8)	0.31	317.36 (187.01 ~ 501.31)	397.49 (230.49 ~ 603.67)	0.74 (0.68 ~ 0.8)
Northern Mariana Islands	9.44 (4.91 ~ 16.02)	10.56 (5.52 ~ 18.55)	0.12	68.36 (35.55 ~ 116.02)	92.31 (48.21 ~ 162.1)	1.09 (0.65 ~ 1.53)
Norway	3128.01 (1803.36 ~ 4738.93)	2327.23 (1292.01 ~ 3797.34)	-0.26	296.36 (170.86 ~ 448.99)	192.16 (106.68 ~ 313.54)	-1.89 (-2.07 ~ -1.71)
Oman	62.71 (30.74 ~ 110.26)	200.32 (97.3 ~ 338.93)	2.19	18.5 (9.07 ~ 32.52)	19.73 (9.58 ~ 33.38)	0.3 (-0.09 ~ 0.69)
Pakistan	7025.38 (3174.09 ~ 12850.33)	12175.23 (6079.54 ~ 21452.07)	0.73	29.7 (13.42 ~ 54.32)	20.1 (10.04 ~ 35.41)	-1.37 (-1.43 ~ -1.32)
Palau	1.88 (0.97 ~ 3.34)	2.13 (1.1 ~ 3.7)	0.13	45.83 (23.59 ~ 81.4)	56.75 (29.44 ~ 98.86)	0.38 (0.23 ~ 0.53)
Palestine	109.25 (55.67 ~ 193.58)	321.43 (149.38 ~ 593.43)	1.94	24.71 (12.59 ~ 43.79)	24.69 (11.47 ~ 45.57)	-0.21 (-0.29 ~ -0.12)
Panama	228.12 (108.21 ~ 394.23)	342.34 (170.46 ~ 589.91)	0.5	37.28 (17.68 ~ 64.42)	32.03 (15.95 ~ 55.19)	-0.58 (-0.62 ~ -0.54)
Papua New Guinea	636.33 (336.23 ~ 1070.95)	2053.64 (1108.53 ~ 3357.41)	2.23	66.03 (34.89 ~ 111.13)	78.5 (42.37 ~ 128.33)	0.47 (0.39 ~ 0.54)
Paraguay	734.2 (381.26 ~ 1236.68)	1193.25 (607.48 ~ 2077.35)	0.63	78.09 (40.55 ~ 131.54)	62.9 (32.02 ~ 109.51)	-1.04 (-1.16 ~ -0.93)
Peru	861.03 (411.67 ~ 1563.61)	2023.95 (1002.32 ~ 3516.54)	1.35	15.85 (7.58 ~ 28.78)	21.04 (10.42 ~ 36.56)	0.6 (0.46 ~ 0.74)
Philippines	8083.03 (4070.82 ~ 13682.01)	9946.36 (5332 ~ 16493.17)	0.23	52.08 (26.23 ~ 88.16)	33.93 (18.19 ~ 56.27)	-1.61 (-1.82 ~ -1.39)
Poland	39397.7 (23013.6 ~ 61480.73)	29968.18 (16650.92 ~ 47414.72)	-0.24	419.42 (245 ~ 654.51)	339.47 (188.61 ~ 537.09)	-0.94 (-1.11 ~ -0.78)
Portugal	3994.21 (2132.23 ~ 6365.62)	5727.71 (3152.54 ~ 8850.42)	0.43	157.88 (84.28 ~ 251.61)	246.05 (135.43 ~ 380.19)	1.95 (1.79 ~ 2.12)
Puerto Rico	675.94 (352.37 ~ 1137.06)	528.9 (283.67 ~ 902.98)	-0.22	70.55 (36.78 ~ 118.68)	70.53 (37.83 ~ 120.42)	-0.08 (-0.11 ~ -0.05)
Qatar	16.28 (7.77 ~ 30.51)	132.61 (65.34 ~ 236.05)	7.15	20.59 (9.83 ~ 38.59)	23.9 (11.78 ~ 42.55)	0.1 (-0.07 ~ 0.26)
Republic of Korea	8318.73 (4068.99 ~ 14227.02)	7442.03 (3767.72 ~ 13269.67)	-0.11	65.84 (32.2 ~ 112.6)	64.2 (32.5 ~ 114.47)	-0.24 (-0.41 ~ -0.07)
Republic of Moldova	737.25 (351.15 ~ 1297.93)	856.32 (410.68 ~ 1431.68)	0.16	65.51 (31.2 ~ 115.32)	97.08 (46.56 ~ 162.31)	1.18 (1.11 ~ 1.25)
Romania	16380.26 (8927.04 ~ 26777.16)	14529.74 (8036.51 ~ 23084.34)	-0.11	291.3 (158.75 ~ 476.19)	357.82 (197.91 ~ 568.5)	0.82 (0.66 ~ 0.99)
Russian Federation	46592.25 (24441.42 ~ 73785.28)	79166.92 (43518.68 ~ 128006.67)	0.7	125.84 (66.01 ~ 199.28)	233.57 (128.4 ~ 377.67)	2.38 (2.17 ~ 2.6)
Rwanda	444.67 (214.52 ~ 763.41)	893.43 (432.32 ~ 1607.17)	1.01	27.41 (13.22 ~ 47.05)	25.37 (12.28 ~ 45.64)	-0.72 (-0.9 ~ -0.55)
Saint Kitts and Nevis	1.7 (0.77 ~ 3.17)	3.11 (1.48 ~ 5.56)	0.83	17.06 (7.68 ~ 31.7)	19.9 (9.46 ~ 35.62)	0.01 (-0.3 ~ 0.33)
Saint Lucia	11.99 (5.94 ~ 21.63)	16.76 (8.63 ~ 29.83)	0.4	35.15 (17.43 ~ 63.42)	36.69 (18.89 ~ 65.31)	-0.14 (-0.36 ~ 0.08)
Saint Vincent and the Grenadines	5.46 (2.69 ~ 9.62)	8.03 (3.79 ~ 13.63)	0.47	20.73 (10.21 ~ 36.52)	28.89 (13.65 ~ 49.03)	0.95 (0.82 ~ 1.08)
Samoa	24.54 (12.22 ~ 42.27)	36.45 (18.98 ~ 59.21)	0.49	66.56 (33.14 ~ 114.65)	74.93 (39.02 ~ 121.7)	0.24 (-0.03 ~ 0.52)
San Marino	14.04 (8.28 ~ 22.85)	15.78 (8.35 ~ 26.36)	0.12	225.63 (133.14 ~ 367.32)	221.6 (117.26 ~ 370.12)	0.02 (-0.12 ~ 0.17)
Sao Tome and Principe	1.2 (0.59 ~ 2.22)	2.7 (1.25 ~ 5)	1.25	4.68 (2.29 ~ 8.66)	4.81 (2.23 ~ 8.91)	-0.11 (-0.27 ~ 0.05)
Saudi Arabia	449.98 (214.93 ~ 814.96)	2043.93 (924.55 ~ 3829.72)	3.54	13.8 (6.59 ~ 24.99)	20.14 (9.11 ~ 37.75)	1 (0.87 ~ 1.13)
Senegal	101.32 (47.95 ~ 184.25)	157.19 (73.59 ~ 280.75)	0.55	5.89 (2.79 ~ 10.71)	4.03 (1.89 ~ 7.2)	-1.55 (-1.77 ~ -1.32)

Table S1 The DALYs of Smoking-Related Low Back Pain cases and rates among WCBA in 1990 and 2021 across 204 countries, and the trends from 1990 to 2021.

Location	DALYs cases			DALYs rates		
	1990_(95% UI)	2021_(95% UI)	ercentage change(100%	1990_per 100 000(95% UI)	2021_per 100 000(95% UI)	EAPC(95% CI)
Serbia	9149.37 (5205.46 ~ 14265.02)	9152.56 (5223.74 ~ 14250.41)	0	391.85 (222.94 ~ 610.94)	450.26 (256.98 ~ 701.04)	0.45 (0.37 ~ 0.53)
Seychelles	4.23 (1.97 ~ 7.46)	7.53 (3.55 ~ 13.5)	0.78	23.38 (10.9 ~ 41.21)	31.07 (14.63 ~ 55.67)	0.9 (0.72 ~ 1.08)
Sierra Leone	311.23 (157.15 ~ 540.7)	369.83 (187.01 ~ 647.98)	0.19	31 (15.65 ~ 53.85)	16.26 (8.22 ~ 28.49)	-2.59 (-2.93 ~ -2.26)
Singapore	771.92 (373.94 ~ 1316.32)	1053.18 (514.93 ~ 1839.47)	0.36	82.59 (40.01 ~ 140.84)	72.4 (35.4 ~ 126.45)	-0.46 (-0.56 ~ -0.35)
Slovakia	4642.61 (2597.68 ~ 7511.5)	4108.58 (2252.59 ~ 6647.81)	-0.12	349.99 (195.83 ~ 566.26)	325.5 (178.46 ~ 526.67)	-0.47 (-0.63 ~ -0.31)
Slovenia	1478.72 (793.03 ~ 2311.62)	1519.38 (822.66 ~ 2426.26)	0.03	297.18 (159.37 ~ 464.57)	361.23 (195.58 ~ 576.84)	1.05 (0.9 ~ 1.21)
Solomon Islands	38.95 (18.69 ~ 63.2)	128.42 (70.92 ~ 210.35)	2.3	52.34 (25.12 ~ 84.93)	74.9 (41.37 ~ 122.69)	1.64 (1.39 ~ 1.88)
Somalia	189.75 (85.83 ~ 354.87)	404.18 (176.12 ~ 748.75)	1.13	11.2 (5.07 ~ 20.95)	8.36 (3.64 ~ 15.48)	-1.15 (-1.42 ~ -0.88)
South Africa	5958.83 (3423.52 ~ 9932.67)	6073.11 (3164.92 ~ 10139.3)	0.02	61.73 (35.46 ~ 102.89)	39.21 (20.44 ~ 65.47)	-1.69 (-1.85 ~ -1.53)
South Sudan	115.09 (54.09 ~ 218.35)	207.45 (96.78 ~ 376.46)	0.8	8.89 (4.18 ~ 16.86)	8.94 (4.17 ~ 16.22)	0.1 (-0.08 ~ 0.28)
Spain	18885.69 (11344.97 ~ 28888.92)	21329.35 (11883.58 ~ 33089.75)	0.13	196.1 (117.8 ~ 299.97)	215.38 (120 ~ 334.13)	0.85 (0.56 ~ 1.14)
Sri Lanka	705.83 (318.93 ~ 1278.44)	591.86 (284.81 ~ 1013.55)	-0.16	15.37 (6.95 ~ 27.85)	10.48 (5.04 ~ 17.95)	-1.44 (-1.61 ~ -1.27)
Sudan	911.16 (412.82 ~ 1644.55)	1830.94 (890.85 ~ 3266.56)	1.01	19.46 (8.82 ~ 35.13)	16.22 (7.89 ~ 28.94)	-0.69 (-0.79 ~ -0.6)
Suriname	57.98 (30.23 ~ 97.47)	79.81 (37.15 ~ 139.48)	0.38	59.62 (31.09 ~ 100.23)	54.99 (25.6 ~ 96.11)	-0.53 (-0.75 ~ -0.31)
Sweden	2874.89 (1729.08 ~ 4414.66)	3076.25 (1636.84 ~ 4927.85)	0.07	140.05 (84.23 ~ 215.06)	140.02 (74.5 ~ 224.3)	-0.47 (-0.82 ~ -0.12)
Switzerland	5072.91 (2997.14 ~ 7747.69)	4611.64 (2481.53 ~ 7174.49)	-0.09	288.19 (170.27 ~ 440.15)	235.7 (126.83 ~ 366.69)	-0.86 (-0.98 ~ -0.74)
Syrian Arab Republic	1251.44 (622.21 ~ 2179.34)	1743.08 (800.13 ~ 3195.98)	0.39	45.38 (22.56 ~ 79.04)	44.8 (20.57 ~ 82.15)	0.13 (-0.12 ~ 0.37)
Taiwan (Province of China)	2751.67 (1311.62 ~ 4778.25)	2402.24 (1201.84 ~ 4161.88)	-0.13	50.01 (23.84 ~ 86.84)	42.76 (21.39 ~ 74.09)	-0.68 (-0.85 ~ -0.52)
Tajikistan	214.29 (104.11 ~ 391.5)	417.82 (205.94 ~ 750.74)	0.95	17.77 (8.64 ~ 32.47)	16.44 (8.1 ~ 29.54)	-0.42 (-0.59 ~ -0.25)
Thailand	3041.51 (1520.67 ~ 5306.66)	2699.12 (1259.93 ~ 4846.91)	-0.11	19.02 (9.51 ~ 33.19)	16.56 (7.73 ~ 29.73)	-0.59 (-0.7 ~ -0.47)
Timor-Leste	32.23 (15.16 ~ 58.86)	47.37 (22.26 ~ 84.23)	0.47	17.3 (8.13 ~ 31.58)	13.68 (6.43 ~ 24.33)	-1.13 (-1.28 ~ -0.99)
Togo	77.53 (36.64 ~ 138.93)	149.05 (70.96 ~ 250.63)	0.92	9.09 (4.29 ~ 16.28)	6.91 (3.29 ~ 11.62)	-0.97 (-1.05 ~ -0.88)
Tokelau	0.24 (0.12 ~ 0.4)	0.25 (0.12 ~ 0.43)	0.04	68.93 (35.35 ~ 113.61)	78.55 (38.38 ~ 134.75)	0.35 (0.22 ~ 0.48)
Tonga	11.44 (5.92 ~ 18.63)	13.96 (7.12 ~ 22.93)	0.22	51.15 (26.48 ~ 83.28)	55.21 (28.16 ~ 90.68)	0.24 (0.18 ~ 0.31)
Trinidad and Tobago	163.13 (85.67 ~ 278)	190.14 (98.27 ~ 320.76)	0.17	53.03 (27.85 ~ 90.37)	55.94 (28.92 ~ 94.38)	0.03 (-0.11 ~ 0.17)
Tunisia	459.46 (217.66 ~ 819.41)	802.13 (391.01 ~ 1442.3)	0.75	22.47 (10.65 ~ 40.08)	26.14 (12.74 ~ 47.01)	0.36 (0.28 ~ 0.43)
Türkiye	15056 (8521.9 ~ 24251.95)	32808.87 (17470.35 ~ 53518.2)	1.18	105.7 (59.83 ~ 170.26)	151.53 (80.69 ~ 247.18)	1.21 (1.14 ~ 1.29)
Turkmenistan	384.02 (177.92 ~ 679.28)	566.29 (263.4 ~ 1027.33)	0.47	43.45 (20.13 ~ 76.85)	45.07 (20.96 ~ 81.75)	0.18 (-0.25 ~ 0.6)
Tuvalu	2.15 (1.12 ~ 3.95)	2.41 (1.31 ~ 4.1)	0.12	88.29 (45.92 ~ 162.32)	82.81 (45.17 ~ 141.29)	-0.38 (-0.52 ~ -0.25)
Uganda	385.52 (185.33 ~ 681.7)	1119.37 (545.69 ~ 2072.98)	1.9	10.02 (4.82 ~ 17.72)	10.75 (5.24 ~ 19.91)	0.08 (-0.01 ~ 0.17)
Ukraine	23632.38 (12919.55 ~ 38841.35)	22549.3 (11795.67 ~ 35920.46)	-0.05	186.69 (102.06 ~ 306.84)	223.14 (116.73 ~ 355.46)	1.05 (0.88 ~ 1.21)
United Arab Emirates	111.62 (52.21 ~ 198.7)	929.51 (435.16 ~ 1672.32)	7.33	32.61 (15.25 ~ 58.06)	53.92 (25.24 ~ 97)	1.77 (1.49 ~ 2.06)

Table S1 The DALYs of Smoking-Related Low Back Pain cases and rates among WCBA in 1990 and 2021 across 204 countries, and the trends from 1990 to 2021.

Location	DALYs cases			DALYs rates		
	1990_(95% UI)	2021_(95% UI)	ercentage change(100%	1990_per 100 000(95% UI)	2021_per 100 000(95% UI)	EAPC(95% CI)
United Kingdom	40776.7 (23693.29 ~ 62354.96)	27524.6 (14791.96 ~ 43408.3)	-0.32	287.33 (166.95 ~ 439.38)	179.44 (96.43 ~ 282.98)	-1.73 (-1.97 ~ -1.49)
United Republic of Tanzania	2396.82 (1276.84 ~ 3945.43)	6294.47 (3457.25 ~ 10199.2)	1.63	39.95 (21.28 ~ 65.76)	42.36 (23.27 ~ 68.64)	0.21 (0.16 ~ 0.25)
United States of America	221387.25 (125265.53 ~ 347172.68)	161678.03 (93035.05 ~ 251773.57)	-0.27	330.45 (186.98 ~ 518.21)	213.54 (122.88 ~ 332.53)	-1.56 (-1.62 ~ -1.49)
United States Virgin Islands	18.96 (8.85 ~ 32.51)	9.4 (4.6 ~ 15.79)	-0.5	65.97 (30.8 ~ 113.12)	54.65 (26.76 ~ 91.76)	-1.03 (-1.19 ~ -0.87)
Uruguay	1917.73 (1071.26 ~ 3017.86)	2161.99 (1267.24 ~ 3492.39)	0.13	255.35 (142.64 ~ 401.84)	259.74 (152.24 ~ 419.57)	-0.25 (-0.44 ~ -0.06)
Uzbekistan	678 (344.3 ~ 1229.93)	2257.81 (1088.02 ~ 4008.83)	2.33	13.84 (7.03 ~ 25.11)	25.37 (12.23 ~ 45.05)	1.96 (1.62 ~ 2.31)
Vanuatu	5.71 (2.71 ~ 9.95)	11.11 (5.54 ~ 19.43)	0.95	16.29 (7.74 ~ 28.37)	14.17 (7.06 ~ 24.78)	-0.87 (-1.13 ~ -0.61)
Venezuela (Bolivarian Republic of)	4391.71 (2069.47 ~ 7224.21)	5101.42 (2544.68 ~ 8694.05)	0.16	90.73 (42.75 ~ 149.24)	74.32 (37.07 ~ 126.67)	-0.98 (-1.1 ~ -0.86)
Viet Nam	1640.53 (805.12 ~ 2864.79)	3327.46 (1523.49 ~ 5691.8)	1.03	9.61 (4.72 ~ 16.79)	13.03 (5.96 ~ 22.28)	0.61 (0.33 ~ 0.89)
Yemen	1953.32 (946.82 ~ 3506)	5688.33 (2881.09 ~ 10631.1)	1.91	71.31 (34.57 ~ 128)	68.08 (34.48 ~ 127.23)	-0.25 (-0.4 ~ -0.1)
Zambia	115.85 (55.54 ~ 218.87)	273.2 (125.33 ~ 504.36)	1.36	6.3 (3.02 ~ 11.9)	5.55 (2.55 ~ 10.25)	-0.69 (-0.93 ~ -0.44)
Zimbabwe	157.7 (78.2 ~ 284.27)	261.99 (124.31 ~ 479.39)	0.66	6.56 (3.25 ~ 11.82)	6.45 (3.06 ~ 11.81)	-0.21 (-0.46 ~ 0.05)

TableS2 Frontier analysis of Smoking-Related Low Back Pain among WCBA

	location	year	val	SDI	frontier	eff_diff
1	Afghanistan	1990	15.00549956237	0.173832165	3.65960675177793	11.3458928105921
2	Afghanistan	1991	14.65491622684	0.17647255	3.62262500489919	11.0322912219408
3	Afghanistan	1992	14.05579288258	0.179633724	3.62857596289619	10.4272169196838
4	Afghanistan	1993	13.18805949697	0.180183713	3.63267320357521	9.55538629339479
5	Afghanistan	1994	12.59221835953	0.17851866	3.63938396770317	8.95283439182683
6	Afghanistan	1995	12.42459919902	0.178279128	3.64969386566255	8.77490533335745
7	Afghanistan	1996	12.6754925734	0.178085404	3.68961770920761	8.9858748641924
8	Afghanistan	1997	13.1832293146	0.177884124	3.67683525065413	9.50639406394587
9	Afghanistan	1998	13.80444048329	0.177513403	3.63805151712803	10.166388966162
10	Afghanistan	1999	14.5263348686	0.177075239	3.6152726113841	10.9110622572159
11	Afghanistan	2000	15.81981035355	0.177025772	3.64085893806465	12.1789514154854
12	Afghanistan	2001	17.16401753564	0.177773144	3.65731510750739	13.5067024281326
13	Afghanistan	2002	18.44006775017	0.18380843	3.64754607671146	14.7925216734585
14	Afghanistan	2003	19.48135773382	0.190518991	3.48614807557662	15.9952096582434
15	Afghanistan	2004	20.56798389624	0.196903541	3.481494081225	17.086489815015
16	Afghanistan	2005	21.60091739787	0.203928133	3.47029496051364	18.1306224373564
17	Afghanistan	2006	22.39970765333	0.211074194	3.48521005222125	18.9144976011087
18	Afghanistan	2007	23.25897721878	0.219627611	2.92298273619443	20.3359944825856
19	Afghanistan	2008	24.18338158051	0.228089171	2.76980900912322	21.4135725713868
20	Afghanistan	2009	24.93448277107	0.237900824	2.60159756162971	22.3328852094403
21	Afghanistan	2010	25.49876754511	0.247759949	2.55821496808982	22.9405525770202
22	Afghanistan	2011	25.91184527636	0.257041676	2.51554729497147	23.3962979813885
23	Afghanistan	2012	26.20468074966	0.266484407	2.48705261765079	23.7176281320092
24	Afghanistan	2013	26.34052726235	0.275636558	2.45911060940765	23.8814166529423
25	Afghanistan	2014	26.47372961894	0.284030351	2.43493791737408	24.0387917015659

TableS2 Frontier analysis of Smoking-Related Low Back Pain among WCBA

	location	year	val	SDI	frontier	eff_diff
26	Afghanistan	2015	26.4481822349	0.291849505	2.41843257795703	24.029749656943
27	Afghanistan	2016	26.30384045928	0.299630696	2.38572171872296	23.918118740557
28	Afghanistan	2017	25.90687733922	0.307424618	2.38435456163344	23.5225227775866
29	Afghanistan	2018	25.3615925116	0.314866093	2.35851277408134	23.0030797375187
30	Afghanistan	2019	24.70984044067	0.322454047	2.3416323758884	22.3682080647816
31	Afghanistan	2020	23.88237768452	0.329830068	2.30468412480253	21.5776935597175
32	Afghanistan	2021	22.9657469654	0.337199998	2.270295904184	20.695451061216
33	Albania	1990	56.24849397238	0.5577733	2.11706405560532	54.1314299167747
34	Albania	1991	57.86779491917	0.555629412	2.11358399455944	55.7542109246106
35	Albania	1992	59.55472084676	0.553107897	2.10951905232726	57.4452017944327
36	Albania	1993	61.30281128431	0.552453328	2.11097037210831	59.1918409122017
37	Albania	1994	63.23969458826	0.553763669	2.11129336899279	61.1284012192672
38	Albania	1995	64.96063722063	0.557040889	2.11775366685366	62.8428835537763
39	Albania	1996	67.03169365691	0.562248886	2.11434659944	64.91734705747
40	Albania	1997	69.16454691527	0.566802804	2.1128875249097	67.0516593903603
41	Albania	1998	71.40367883574	0.572534102	2.11655068736119	69.2871281483788
42	Albania	1999	73.82411598317	0.578295462	2.11695809388222	71.7071578892878
43	Albania	2000	76.65618162951	0.584664188	2.11173954753462	74.5444420819754
44	Albania	2001	79.53092299881	0.592412947	2.1137035869613	77.4172194118487
45	Albania	2002	82.99566980293	0.599865978	2.11320026922608	80.8824695337039
46	Albania	2003	86.78933650717	0.606971166	2.11581087812822	84.6735256290418
47	Albania	2004	90.90160843294	0.613864247	2.11154463146917	88.7900638014708
48	Albania	2005	95.21598805226	0.620679615	2.11388731575292	93.1021007365071
49	Albania	2006	101.22669998227	0.627485617	2.11435499082782	99.1123449914422
50	Albania	2007	109.16182720934	0.634167969	2.11218328910215	107.049643920238

TableS2 Frontier analysis of Smoking-Related Low Back Pain among WCBA

	location	year	val	SDI	frontier	eff_diff
51	Albania	2008	117.30306128579	0.640766134	2.11329943710821	115.189761848682
52	Albania	2009	124.00650477206	0.64681613	2.11274059513854	121.893764176921
53	Albania	2010	127.66173292062	0.652654203	2.11208750858031	125.54964541204
54	Albania	2011	128.89423871504	0.658196773	2.11245224250348	126.781786472537
55	Albania	2012	130.03459502689	0.66355678	2.11175133263986	127.92284369425
56	Albania	2013	130.96187166535	0.668739559	2.11274955527708	128.849122110073
57	Albania	2014	131.81423085833	0.674112985	2.11184915754918	129.702381700781
58	Albania	2015	132.81419913839	0.679668601	2.11196722284012	130.70223191555
59	Albania	2016	133.07024763721	0.685036648	2.1134945009903	130.95675313622
60	Albania	2017	132.25964967382	0.690379199	2.116256962722	130.143392711098
61	Albania	2018	130.92318356542	0.695566878	2.112787372035	128.810396193385
62	Albania	2019	129.92247543886	0.700287863	2.11573769277844	127.806737746082
63	Albania	2020	128.68326939138	0.703790245	2.11419267639432	126.569076714986
64	Albania	2021	127.50559897743	0.706849791	2.11126240145721	125.394336575973
65	Algeria	1990	10.98135549903	0.460486906	2.11590124717122	8.86545425185878
66	Algeria	1991	10.94682612006	0.468319197	2.11481063076837	8.83201548929162
67	Algeria	1992	10.93000182724	0.475950937	2.11509242385864	8.81490940338136
68	Algeria	1993	10.91037083757	0.483255591	2.1161011317437	8.79426970582631
69	Algeria	1994	10.90891122679	0.49045066	2.11291520379947	8.79599602299053
70	Algeria	1995	10.89981179437	0.497957543	2.11170102326742	8.78811077110258
71	Algeria	1996	10.90296527575	0.505990967	2.113573742067	8.789391533683
72	Algeria	1997	10.86531264799	0.514056566	2.1215652583294	8.7437473896606
73	Algeria	1998	10.76806546388	0.522382419	2.11381969892375	8.65424576495625
74	Algeria	1999	10.68679377429	0.530785583	2.1107137029	8.57608007139
75	Algeria	2000	10.58855412745	0.539481508	2.11597219499508	8.47258193245492

TableS2 Frontier analysis of Smoking-Related Low Back Pain among WCBA

	location	year	val	SDI	frontier	eff_diff
76	Algeria	2001	10.5095490305	0.547529277	2.11465291706882	8.39489611343119
77	Algeria	2002	10.41671323259	0.555172222	2.11074360137574	8.30596963121426
78	Algeria	2003	10.3402473513	0.562536956	2.11362665385028	8.22662069744972
79	Algeria	2004	10.27249636295	0.569352852	2.11482528195383	8.15767108099617
80	Algeria	2005	10.21802229831	0.576001965	2.11188435359629	8.10613794471372
81	Algeria	2006	10.19187229382	0.582126362	2.11168591391675	8.08018637990325
82	Algeria	2007	10.21062389868	0.587790538	2.11351300254293	8.09711089613707
83	Algeria	2008	10.23235643498	0.593190973	2.11395021826864	8.11840621671136
84	Algeria	2009	10.30069884762	0.597870384	2.11538676139884	8.18531208622116
85	Algeria	2010	10.37065530748	0.602823535	2.11417130688696	8.25648400059304
86	Algeria	2011	10.3908811796	0.607825529	2.11532770974643	8.27555346985357
87	Algeria	2012	10.37139760609	0.612703533	2.11380520778757	8.25759239830243
88	Algeria	2013	10.35646252809	0.617422204	2.11357832062143	8.24288420746857
89	Algeria	2014	10.4088208269	0.622087292	2.11516367309653	8.29365715380347
90	Algeria	2015	10.54774309225	0.626745568	2.11026664650025	8.43747644574975
91	Algeria	2016	10.7624553475	0.631710736	2.11328269101106	8.64917265648894
92	Algeria	2017	10.99652403667	0.636973176	2.1150982853874	8.8814257512826
93	Algeria	2018	11.2204773309	0.642500039	2.11027872507709	9.11019860582291
94	Algeria	2019	11.43039839659	0.648210785	2.11266010512714	9.31773829146286
95	Algeria	2020	11.62590222401	0.653651466	2.11543329521606	9.51046892879394
96	Algeria	2021	11.81888951164	0.659500924	2.11279186783493	9.70609764380507
97	American Samoa	1990	89.56519836422	0.613633924	2.11549876490835	87.4496995993117
98	American Samoa	1991	91.5700984238	0.615847612	2.11451320752316	89.4555852162768
99	American Samoa	1992	93.916494656	0.618583654	2.11087114349948	91.8056235125005
100	American Samoa	1993	96.35725363135	0.622078598	2.11314881499244	94.2441048163576

TableS2 Frontier analysis of Smoking-Related Low Back Pain among WCBA

	location	year	val	SDI	frontier	eff_diff
101	American Samoa	1994	99.08842434988	0.626053241	2.11125873131654	96.9771656185635
102	American Samoa	1995	101.75911988586	0.629814054	2.11390189722076	99.6452179886392
103	American Samoa	1996	104.47371037421	0.632706983	2.11316702531094	102.360543348899
104	American Samoa	1997	107.17938099716	0.634926685	2.11328534658985	105.06609565057
105	American Samoa	1998	109.6201835442	0.636597976	2.11748959592738	107.502693948273
106	American Samoa	1999	111.70897245423	0.638322193	2.11615347997214	109.592818974258
107	American Samoa	2000	114.16958824431	0.64060955	2.1152371307229	112.054351113587
108	American Samoa	2001	115.49365099754	0.643214421	2.11344328324	113.3802077143
109	American Samoa	2002	117.13692857618	0.646002062	2.11491190510383	115.022016671076
110	American Samoa	2003	118.25900702935	0.648951964	2.11463534327071	116.144371686079
111	American Samoa	2004	119.11852602343	0.651921141	2.11390028039069	117.004625743039
112	American Samoa	2005	119.95455621963	0.655061005	2.11222215203891	117.842334067591
113	American Samoa	2006	120.13725619089	0.657725723	2.11280596272767	118.024450228162
114	American Samoa	2007	119.75322853289	0.660781176	2.11074669592085	117.642481836969
115	American Samoa	2008	118.93802276609	0.664025762	2.11073073491703	116.827292031173
116	American Samoa	2009	118.09800010121	0.667300532	2.115501067994	115.982499033216
117	American Samoa	2010	117.51129817835	0.671248916	2.11248331159256	115.398814866757
118	American Samoa	2011	117.12268885088	0.675979288	2.11612925506622	115.006559595814
119	American Samoa	2012	116.96890459068	0.680861539	2.11485986624851	114.854044724432
120	American Samoa	2013	116.80816290945	0.685833061	2.11139621575301	114.696766693697
121	American Samoa	2014	116.69477052775	0.691343137	2.11270712004557	114.582063407704
122	American Samoa	2015	116.5053270701	0.697162912	2.11076341315551	114.394563656944
123	American Samoa	2016	115.94817944491	0.702726398	2.11326882549857	113.834910619411
124	American Samoa	2017	115.17320160126	0.707647909	2.11504524020482	113.058156361055
125	American Samoa	2018	114.63331165578	0.712800591	2.11683442011418	112.516477235666

TableS2 Frontier analysis of Smoking-Related Low Back Pain among WCBA

	location	year	val	SDI	frontier	eff_diff
126	American Samoa	2019	113.84984449646	0.717308133	2.11361105739661	111.736233439063
127	American Samoa	2020	113.70081359366	0.7208385	2.11048468304825	111.590328910612
128	American Samoa	2021	112.30714401632	0.723727533	2.11016658495132	110.196977431369
129	Andean Latin America	1990	19.03517602682	0.50001149	2.110214251169	16.924961775651
130	Andean Latin America	1991	19.12367400271	0.501652202	2.109201311815	17.014472690895
131	Andean Latin America	1992	19.25610966486	0.50365586	2.11745961025062	17.1386500546094
132	Andean Latin America	1993	19.39235905266	0.506388431	2.11397333358924	17.2783857190708
133	Andean Latin America	1994	19.4841434825	0.510305574	2.11409975512224	17.3700437273778
134	Andean Latin America	1995	19.58651931359	0.514896344	2.11368048518488	17.4728388284051
135	Andean Latin America	1996	19.62002638072	0.519376447	2.11223903166258	17.5077873490574
136	Andean Latin America	1997	19.49641958434	0.52403649	2.11636288196945	17.3800567023705
137	Andean Latin America	1998	19.39091064473	0.528443909	2.11325981724579	17.2776508274842
138	Andean Latin America	1999	19.27109137795	0.532743717	2.11610667585293	17.1549847020971
139	Andean Latin America	2000	19.26028196898	0.537560512	2.11379542557937	17.1464865434006
140	Andean Latin America	2001	19.23842813756	0.54257235	2.11147834952529	17.1269497880347
141	Andean Latin America	2002	19.1408398131	0.547911071	2.11370493264014	17.0271348804599
142	Andean Latin America	2003	18.98803537999	0.553108984	2.11127589069183	16.8767594892982
143	Andean Latin America	2004	18.79748411272	0.558235444	2.11096477985419	16.6865193328658
144	Andean Latin America	2005	18.7011263882	0.563091517	2.11282307805284	16.5883033101472
145	Andean Latin America	2006	18.66680700525	0.567987399	2.11760235156125	16.5492046536888
146	Andean Latin America	2007	18.65129289055	0.572998972	2.1133606157051	16.5379322748449
147	Andean Latin America	2008	18.67686843719	0.578564192	2.11225714732635	16.5646112898636
148	Andean Latin America	2009	18.66756133849	0.583998942	2.11297104302833	16.5545902954617
149	Andean Latin America	2010	18.68642624318	0.59020118	2.1112834586819	16.5751427844981
150	Andean Latin America	2011	18.65531986766	0.59694462	2.11893195819908	16.5363879094609

TableS2 Frontier analysis of Smoking-Related Low Back Pain among WCBA

	location	year	val	SDI	frontier	eff_diff
151	Andean Latin America	2012	18.64682481935	0.603839225	2.1153835530125	16.5314412663375
152	Andean Latin America	2013	18.63738233249	0.610705169	2.11495803282884	16.5224242996612
153	Andean Latin America	2014	18.66511906644	0.617059985	2.11409284387647	16.5510262225635
154	Andean Latin America	2015	18.7461701086	0.622760754	2.11661345988233	16.6295566487177
155	Andean Latin America	2016	18.92026068827	0.628161263	2.11235470246925	16.8079059858007
156	Andean Latin America	2017	19.17317311863	0.633537897	2.11427181916111	17.0589012994689
157	Andean Latin America	2018	19.41858589015	0.638860164	2.112447586833	17.306138303317
158	Andean Latin America	2019	19.57836547957	0.643921325	2.11578976614019	17.4625757134298
159	Andean Latin America	2020	19.5691713259	0.647806819	2.11908423616441	17.4500870897356
160	Andean Latin America	2021	19.67841192151	0.651602456	2.11329295101214	17.5651189704979
161	Andorra	1990	240.49229233363	0.76146388	2.11291614179562	238.379376191834
162	Andorra	1991	232.36860449509	0.764709071	2.11662734603521	230.251977149055
163	Andorra	1992	231.36821545565	0.767188282	2.11284402508612	229.255371430564
164	Andorra	1993	237.71848982142	0.768953828	2.11162141183816	235.606868409582
165	Andorra	1994	241.86758632229	0.770023765	2.11389806641836	239.753688255872
166	Andorra	1995	246.03827498579	0.770988683	2.11346932806673	243.924805657723
167	Andorra	1996	248.15310177383	0.772403471	2.11603920057125	246.037062573259
168	Andorra	1997	250.5897443882	0.774580971	2.11158912235543	248.478155265845
169	Andorra	1998	253.88210120423	0.776972578	2.11371959222953	251.768381612
170	Andorra	1999	258.90203219282	0.779629634	2.11282182402266	256.789210368797
171	Andorra	2000	264.37726991521	0.781976506	2.11430699413731	262.262962921073
172	Andorra	2001	268.85336327175	0.785417035	2.11381829701956	266.73954497473
173	Andorra	2002	272.75334875563	0.790158759	2.11253232307522	270.640816432555
174	Andorra	2003	267.82423866026	0.797278901	2.11264773565592	265.711590924604
175	Andorra	2004	265.86687664787	0.80473198	2.11475759053852	263.752119057331

TableS2 Frontier analysis of Smoking-Related Low Back Pain among WCBA

	location	year	val	SDI	frontier	eff_diff
176	Andorra	2005	263.50869138166	0.81221398	2.11362802033663	261.395063361323
177	Andorra	2006	262.99356807141	0.81978726	2.11650943511356	260.877058636296
178	Andorra	2007	264.9837607821	0.826617254	2.1132634083327	262.870497373767
179	Andorra	2008	266.26004942949	0.831821715	2.11290159506084	264.147147834429
180	Andorra	2009	269.96262521602	0.835929887	2.11775110080466	267.844874115215
181	Andorra	2010	271.76503656076	0.839489966	2.11299277042692	269.652043790333
182	Andorra	2011	272.04652653694	0.842794802	2.11124640980704	269.935280127133
183	Andorra	2012	272.92083279111	0.845810831	2.11581682734237	270.805015963768
184	Andorra	2013	271.66698925864	0.84832991	2.11169157644774	269.555297682192
185	Andorra	2014	270.3740307096	0.851010492	2.11528096412316	268.258749745477
186	Andorra	2015	269.35483642067	0.853591218	2.11589146394542	267.238944956725
187	Andorra	2016	268.42211885588	0.856257053	2.11231750537078	266.309801350509
188	Andorra	2017	266.68460828068	0.85947703	2.11688537499038	264.56772290569
189	Andorra	2018	266.03043676831	0.862588951	2.11251421821875	263.917922550091
190	Andorra	2019	264.88939544361	0.865621024	2.11241431423034	262.77698112938
191	Andorra	2020	261.78417730771	0.867449169	2.1142231175151	259.669954190195
192	Andorra	2021	261.59580842411	0.869444113	2.11322744900885	259.482580975101
193	Angola	1990	18.84920717268	0.270736935	2.50095264981826	16.3482545228617
194	Angola	1991	18.60177282831	0.275160625	2.45289207204457	16.1488807562654
195	Angola	1992	18.35225994553	0.2792315	2.45049063243708	15.9017693130929
196	Angola	1993	18.08623434875	0.281867703	2.44801317618455	15.6382211725655
197	Angola	1994	17.81835322694	0.284487594	2.44968411549349	15.3686691114465
198	Angola	1995	17.70264006967	0.287447813	2.42715183675158	15.2754882329184
199	Angola	1996	17.53922286851	0.291421279	2.44069008499767	15.0985327835123
200	Angola	1997	17.34362203782	0.295433993	2.40703134245172	14.9365906953683

TableS2 Frontier analysis of Smoking-Related Low Back Pain among WCBA

	location	year	val	SDI	frontier	eff_diff
201	Angola	1998	17.16030416475	0.299553353	2.39964516494983	14.7606589998002
202	Angola	1999	17.01495607718	0.303720042	2.3846199620525	14.6303361151275
203	Angola	2000	16.81311406436	0.307603312	2.38064751381918	14.4324665505408
204	Angola	2001	16.70646062194	0.311697534	2.36674663766855	14.3397139842715
205	Angola	2002	16.58285300308	0.316706571	2.36461853847139	14.2182344646086
206	Angola	2003	16.52430112634	0.321777362	2.34088429141185	14.1834168349281
207	Angola	2004	16.46880113762	0.327395266	2.32306982972517	14.1457313078948
208	Angola	2005	16.47888830855	0.333785188	2.29176879485264	14.1871195136974
209	Angola	2006	16.61598280473	0.341012622	2.24196067509773	14.3740221296323
210	Angola	2007	16.79690588222	0.348846841	2.12292338293229	14.6739824992877
211	Angola	2008	17.00137150042	0.357082005	2.11563917318205	14.8857323272379
212	Angola	2009	17.16631610776	0.364407744	2.11372345425121	15.0525926535088
213	Angola	2010	17.3170980564	0.372033537	2.11800819039333	15.1990898660067
214	Angola	2011	17.38700776565	0.380006613	2.11397144269263	15.2730363229574
215	Angola	2012	17.45771040193	0.387891857	2.11313864906818	15.3445717528618
216	Angola	2013	17.51414119419	0.395796329	2.11353105773915	15.4006101364508
217	Angola	2014	17.60605956599	0.403720086	2.11390786205684	15.4921517039332
218	Angola	2015	17.75390012667	0.411372026	2.11113414466426	15.6427659820057
219	Angola	2016	17.95405010731	0.418822062	2.11516338622169	15.8388867210883
220	Angola	2017	18.2249891038	0.426177082	2.11281064155033	16.1121784622497
221	Angola	2018	18.4678196298	0.433355005	2.113664651575	16.354154978225
222	Angola	2019	18.67978255756	0.440454598	2.11478534888468	16.5649972086753
223	Angola	2020	18.69701211018	0.447283992	2.11407672088762	16.5829353892924
224	Angola	2021	18.63465897464	0.453721949	2.113642462248	16.521016512392
225	Antigua and Barbuda	1990	38.14872608525	0.612104591	2.11448593158413	36.0342401536659

TableS2 Frontier analysis of Smoking-Related Low Back Pain among WCBA

	location	year	val	SDI	frontier	eff_diff
226	Antigua and Barbuda	1991	39.14164272159	0.618817129	2.11380770050242	37.0278350210876
227	Antigua and Barbuda	1992	40.03225035887	0.624695818	2.11219008810135	37.9200602707686
228	Antigua and Barbuda	1993	40.8771493844	0.630075854	2.11243039009885	38.7647189943012
229	Antigua and Barbuda	1994	41.65990019964	0.634768955	2.11435130079297	39.545548898847
230	Antigua and Barbuda	1995	42.45392729595	0.638008629	2.11408890125436	40.3398383946956
231	Antigua and Barbuda	1996	43.18942083561	0.641647192	2.11426038992927	41.0751604456807
232	Antigua and Barbuda	1997	43.74203280591	0.645895591	2.11455719526538	41.6274756106446
233	Antigua and Barbuda	1998	44.15210762461	0.650614287	2.11285581856592	42.0392518060441
234	Antigua and Barbuda	1999	44.5148251827	0.655534994	2.11324636460165	42.4015788180984
235	Antigua and Barbuda	2000	44.89686943083	0.660819064	2.11118872430507	42.7856807065249
236	Antigua and Barbuda	2001	45.27591788279	0.665620632	2.116063019452	43.159854863338
237	Antigua and Barbuda	2002	45.48096136138	0.670360801	2.11309393617107	43.3678674252089
238	Antigua and Barbuda	2003	45.65145165486	0.67531149	2.11329450150115	43.5381571533589
239	Antigua and Barbuda	2004	45.81299597343	0.680412429	2.11336409206	43.69963188137
240	Antigua and Barbuda	2005	46.0721738804	0.685486437	2.11144819541884	43.9607256849812
241	Antigua and Barbuda	2006	46.20543598324	0.690826021	2.11176731068917	44.0936686725508
242	Antigua and Barbuda	2007	46.29755829706	0.696360039	2.11285293206337	44.1847053649966
243	Antigua and Barbuda	2008	46.27219673923	0.702175362	2.11489408287282	44.1573026563572
244	Antigua and Barbuda	2009	46.31268859508	0.707128564	2.11662527849067	44.1960633165893
245	Antigua and Barbuda	2010	46.34393745664	0.711432246	2.1157275343784	44.2282099222616
246	Antigua and Barbuda	2011	46.3421637631	0.715489989	2.11556839632092	44.2265953667791
247	Antigua and Barbuda	2012	46.39744272306	0.719509323	2.11713752587595	44.280305197184
248	Antigua and Barbuda	2013	46.46365712494	0.722864978	2.11371182037885	44.3499453045611
249	Antigua and Barbuda	2014	46.50159314511	0.725585978	2.11477031582595	44.3868228292841
250	Antigua and Barbuda	2015	46.59238656391	0.728372762	2.11486972981985	44.4775168340902

TableS2 Frontier analysis of Smoking-Related Low Back Pain among WCBA

	location	year	val	SDI	frontier	eff_diff
251	Antigua and Barbuda	2016	46.75425352395	0.731598944	2.11171171936525	44.6425418045848
252	Antigua and Barbuda	2017	46.93777837864	0.734963713	2.11604384088364	44.8217345377564
253	Antigua and Barbuda	2018	47.24672477482	0.738879106	2.11259539137603	45.134129383444
254	Antigua and Barbuda	2019	47.44300145306	0.74305296	2.11263412334385	45.3303673297162
255	Antigua and Barbuda	2020	47.40687555726	0.74634533	2.11220775615449	45.2946678011055
256	Antigua and Barbuda	2021	47.3596746013	0.749886887	2.11421824411145	45.2454563571886
257	Argentina	1990	289.69076345515	0.587397284	2.11479167098441	287.575971784166
258	Argentina	1991	286.22758171296	0.59170754	2.11209298462943	284.115488728331
259	Argentina	1992	282.8511134459	0.597996324	2.11607653263972	280.73503691326
260	Argentina	1993	279.98255134523	0.603739312	2.11089378404333	277.871657561187
261	Argentina	1994	277.72791113344	0.60978733	2.11430256389899	275.613608569541
262	Argentina	1995	275.27647379851	0.614473706	2.11253028740069	273.163943511109
263	Argentina	1996	273.85283165704	0.618942624	2.11509316540565	271.737738491634
264	Argentina	1997	272.16090295638	0.623934409	2.11568193873262	270.045221017647
265	Argentina	1998	270.96605299696	0.628723436	2.11378132255281	268.852271674407
266	Argentina	1999	270.30594669987	0.633175129	2.11354526960524	268.192401430265
267	Argentina	2000	269.55974614175	0.638194472	2.11285652571676	267.446889616033
268	Argentina	2001	269.10243675045	0.642175705	2.11378464100731	266.988652109443
269	Argentina	2002	269.28645539847	0.644472507	2.11543276655987	267.17102263191
270	Argentina	2003	269.06602925927	0.645906577	2.11323237371113	266.952796885559
271	Argentina	2004	268.88090699195	0.648296828	2.11067007156662	266.770236920383
272	Argentina	2005	267.36449343556	0.65336883	2.11653799329951	265.24795544226
273	Argentina	2006	263.53271515176	0.6574459	2.10928646577118	261.423428685989
274	Argentina	2007	258.02541803842	0.660187576	2.11569012485028	255.90972791357
275	Argentina	2008	251.72156778865	0.663079235	2.11323861440521	249.608329174245

TableS2 Frontier analysis of Smoking-Related Low Back Pain among WCBA

	location	year	val	SDI	frontier	eff_diff
276	Argentina	2009	245.99263759727	0.665911944	2.11557192575013	243.87706567152
277	Argentina	2010	241.25757184721	0.66992588	2.11176341814268	239.145808429067
278	Argentina	2011	236.84860482575	0.675242697	2.11240677386476	234.736198051885
279	Argentina	2012	231.97611592819	0.67921824	2.10989858679457	229.866217341395
280	Argentina	2013	227.17607512315	0.681633215	2.11476014868643	225.061314974464
281	Argentina	2014	223.46392105576	0.684974509	2.11392730286067	221.349993752899
282	Argentina	2015	221.86241525672	0.691710126	2.11321187462136	219.749203382099
283	Argentina	2016	222.69974554507	0.698618031	2.11409976440307	220.585645780667
284	Argentina	2017	224.74584775465	0.705135529	2.11173571824762	222.634112036402
285	Argentina	2018	226.47561700835	0.712888693	2.11478849495151	224.360828513398
286	Argentina	2019	227.87697199065	0.719002843	2.11201266682541	225.764959323825
287	Argentina	2020	220.19293774592	0.721294303	2.11425633037313	218.078681415547
288	Argentina	2021	235.45087488014	0.723122973	2.11252790168767	233.338346978452
289	Armenia	1990	31.20378092028	0.544414535	2.1133374975862	29.0904434226938
290	Armenia	1991	31.39880939009	0.547561863	2.11265826454483	29.2861511255452
291	Armenia	1992	31.91038396018	0.548974724	2.11341960149153	29.7969643586885
292	Armenia	1993	32.86501868908	0.551214674	2.11436505781618	30.7506536312638
293	Armenia	1994	33.92086487213	0.553783844	2.11524754725304	31.805617324877
294	Armenia	1995	34.90545065505	0.556815513	2.11540768326517	32.7900429717848
295	Armenia	1996	35.70172470141	0.560537778	2.11247979267299	33.589244908737
296	Armenia	1997	36.45881664757	0.564627462	2.11118411277532	34.3476325347947
297	Armenia	1998	37.30971423055	0.569556663	2.11148407899914	35.1982301515509
298	Armenia	1999	37.90615254854	0.574323064	2.11057945810389	35.7955730904361
299	Armenia	2000	38.2703156972	0.579437569	2.11270837397975	36.1576073232202
300	Armenia	2001	38.4988464118	0.585419295	2.11302401734928	36.3858223944507

TableS2 Frontier analysis of Smoking-Related Low Back Pain among WCBA

	location	year	val	SDI	frontier	eff_diff
301	Armenia	2002	38.45051669208	0.592710614	2.11268890617548	36.3378277859045
302	Armenia	2003	38.47471817932	0.60080917	2.11161235044864	36.3631058288714
303	Armenia	2004	38.27978704335	0.609031766	2.11192422882119	36.1678628145288
304	Armenia	2005	38.03119172017	0.617759634	2.11378785348153	35.9174038666885
305	Armenia	2006	37.73889269461	0.626688335	2.11227805768754	35.6266146369225
306	Armenia	2007	37.34677655095	0.635870982	2.11378972054063	35.2329868304094
307	Armenia	2008	36.97434697533	0.644431591	2.11221604135115	34.8621309339788
308	Armenia	2009	36.60239192162	0.649714845	2.11548244010045	34.4869094815195
309	Armenia	2010	36.23687142216	0.654811534	2.11340181787183	34.1234696042882
310	Armenia	2011	35.91066029096	0.659818092	2.11165775402359	33.7990025369364
311	Armenia	2012	35.64151163589	0.664833143	2.11318086871582	33.5283307671742
312	Armenia	2013	35.58093373842	0.669534496	2.11093140350083	33.4700023349192
313	Armenia	2014	35.6483144712	0.67393219	2.11352174808443	33.5347927231156
314	Armenia	2015	36.03621691596	0.678054635	2.11622612124958	33.9199907947104
315	Armenia	2016	36.55944513169	0.681746395	2.1131278248362	34.4463173068538
316	Armenia	2017	37.17415155106	0.685980265	2.11335175112697	35.060799799933
317	Armenia	2018	37.85226879775	0.690276872	2.11480730445075	35.7374614932993
318	Armenia	2019	38.61777528597	0.694765962	2.11119034241822	36.5065849435518
319	Armenia	2020	39.45657625406	0.698268899	2.11349762038868	37.3430786336713
320	Armenia	2021	40.23935325744	0.701833194	2.11509070888232	38.1242625485577
321	Australasia	1990	359.05716767158	0.731234397	2.11278348481338	356.944384186767
322	Australasia	1991	359.68365235922	0.734915293	2.11379306444325	357.569859294777
323	Australasia	1992	360.00673796515	0.739005622	2.1135166738925	357.893221291258
324	Australasia	1993	360.6723994168	0.743426014	2.11479605840639	358.557603358394
325	Australasia	1994	361.45440469514	0.747726041	2.1106025896181	359.343802105522

TableS2 Frontier analysis of Smoking-Related Low Back Pain among WCBA

	location	year	val	SDI	frontier	eff_diff
326	Australasia	1995	362.85386531168	0.752015072	2.1116484707267	360.742216840953
327	Australasia	1996	364.62581973709	0.756387302	2.11213364330134	362.513686093789
328	Australasia	1997	364.87087354502	0.760990093	2.11136786386159	362.759505681158
329	Australasia	1998	365.67139977481	0.765299502	2.11449952501889	363.556900249791
330	Australasia	1999	366.74841933174	0.769521272	2.11396922906205	364.634450102678
331	Australasia	2000	367.3649995282	0.773791157	2.11364407837781	365.251355449822
332	Australasia	2001	369.07078995141	0.778073921	2.11305060811283	366.957739343297
333	Australasia	2002	370.26099627071	0.782480024	2.11063279416056	368.150363476549
334	Australasia	2003	369.61365059645	0.786285288	2.11258373712393	367.501066859326
335	Australasia	2004	367.87750893871	0.789427625	2.11546427101915	365.762044667691
336	Australasia	2005	364.49826287693	0.791721729	2.11225596526253	362.386006911667
337	Australasia	2006	359.69786057399	0.792856547	2.11280407096692	357.585056503023
338	Australasia	2007	353.40854397935	0.793949581	2.11324001201459	351.295303967335
339	Australasia	2008	346.40463715519	0.796401957	2.11591104485742	344.288726110333
340	Australasia	2009	339.25191057459	0.79993153	2.11485854021051	337.13705203438
341	Australasia	2010	332.18832874152	0.804112332	2.11117615110968	330.07715259041
342	Australasia	2011	324.53959755086	0.808160633	2.11118509887746	322.428412451983
343	Australasia	2012	315.46527872324	0.812463563	2.11348113926356	313.351797583976
344	Australasia	2013	306.71453316042	0.817349223	2.11466855885961	304.59986460156
345	Australasia	2014	299.51610156145	0.821647278	2.11252039171397	297.403581169736
346	Australasia	2015	294.76296744831	0.825611025	2.11614524665867	292.646822201651
347	Australasia	2016	292.89008421069	0.829456874	2.1099728677353	290.780111342955
348	Australasia	2017	293.09453837039	0.83292442	2.11259488692139	290.981943483469
349	Australasia	2018	293.5438915699	0.836618893	2.11308988344845	291.430801686452
350	Australasia	2019	293.45767693361	0.840581577	2.11379598808167	291.343880945528

TableS2 Frontier analysis of Smoking-Related Low Back Pain among WCBA

	location	year	val	SDI	frontier	eff_diff
351	Australasia	2020	285.18966066395	0.843333427	2.110641294816	283.079019369134
352	Australasia	2021	287.88908337378	0.845514063	2.11315463173491	285.775928742045
353	Australia	1990	356.5936335401	0.725982519	2.11457322429012	354.47906031581
354	Australia	1991	357.56493767622	0.729759859	2.11413720636215	355.450800469858
355	Australia	1992	357.83083018378	0.733901404	2.11331761304629	355.717512570734
356	Australia	1993	358.23697102237	0.738374743	2.11370744013537	356.123263582235
357	Australia	1994	358.72938138706	0.742846572	2.11682156694554	356.612559820114
358	Australia	1995	359.94563880908	0.747426824	2.11173127364043	357.83390753544
359	Australia	1996	361.35334245189	0.751991736	2.11360200351147	359.239740448379
360	Australia	1997	360.97409358558	0.756633901	2.10977867447462	358.864314911105
361	Australia	1998	361.0286741738	0.761133265	2.11250785449594	358.916166319304
362	Australia	1999	361.32540805413	0.765697506	2.11572874830938	359.209679305821
363	Australia	2000	361.30550180947	0.770223843	2.11276421938967	359.19273759008
364	Australia	2001	362.97896241207	0.774662787	2.1131060057575	360.865856406313
365	Australia	2002	364.92346850772	0.77936094	2.11335045904742	362.810118048673
366	Australia	2003	365.55880431429	0.783600181	2.1139863226984	363.444817991592
367	Australia	2004	365.18200271822	0.787016543	2.11030085137671	363.071701866843
368	Australia	2005	362.64261837662	0.789615521	2.11563979208825	360.526978584532
369	Australia	2006	358.71427819049	0.791155688	2.11575794387212	356.598520246618
370	Australia	2007	353.20928540153	0.792532683	2.11463730289851	351.094648098631
371	Australia	2008	346.919426174	0.7952929	2.11203627317129	344.807389900829
372	Australia	2009	340.45476600881	0.799174373	2.11276774172625	338.341998267084
373	Australia	2010	333.69550422418	0.803561544	2.11303984863408	331.582464375546
374	Australia	2011	326.13503837167	0.80752022	2.1123852711717	324.022653100498
375	Australia	2012	316.8942609188	0.811644461	2.11632622486132	314.777934693939

TableS2 Frontier analysis of Smoking-Related Low Back Pain among WCBA

	location	year	val	SDI	frontier	eff_diff
376	Australia	2013	308.01345841235	0.816453661	2.11606476292579	305.897393649424
377	Australia	2014	300.78651810494	0.82084085	2.11006663583542	298.676451469105
378	Australia	2015	296.16246444579	0.824860715	2.11670988448845	294.045754561302
379	Australia	2016	294.58759022298	0.828559943	2.1125849664343	292.475005256546
380	Australia	2017	295.16033357466	0.831798266	2.1148702954406	293.045463279219
381	Australia	2018	295.76940373549	0.835354624	2.11591219589987	293.65349153959
382	Australia	2019	295.67022802758	0.839317426	2.11309514064905	293.557132886931
383	Australia	2020	287.30704160082	0.842051314	2.11675647797756	285.190285122842
384	Australia	2021	289.61811256652	0.844252814	2.11228266219289	287.505829904327
385	Austria	1990	212.74732179725	0.749853693	2.11203932166831	210.635282475582
386	Austria	1991	209.95558759919	0.751652734	2.1142324789263	207.841355120264
387	Austria	1992	208.27240939891	0.754375286	2.11643728855029	206.15597211036
388	Austria	1993	208.46074790906	0.758804554	2.11135341638273	206.349394492677
389	Austria	1994	210.14973036067	0.764704364	2.11531873320014	208.03441162747
390	Austria	1995	214.58160766437	0.769827471	2.11491300139588	212.466694662974
391	Austria	1996	220.7266424009	0.774446565	2.11351448241186	218.613127918488
392	Austria	1997	226.46657121413	0.779232856	2.11180923935757	224.354761974772
393	Austria	1998	232.25550366233	0.783521016	2.11332932548591	230.142174336844
394	Austria	1999	237.57817284172	0.787451584	2.11021161799922	235.467961223721
395	Austria	2000	242.07039401139	0.79184768	2.11217601516101	239.958217996229
396	Austria	2001	244.56411625512	0.795770118	2.11382642494487	242.450289830175
397	Austria	2002	245.44010979127	0.79915412	2.11472361923857	243.325386172031
398	Austria	2003	245.6877055014	0.802043253	2.11271472154379	243.574990779856
399	Austria	2004	244.76954645022	0.804889144	2.1103486441225	242.659197806097
400	Austria	2005	243.62787333539	0.808419194	2.11288201873077	241.514991316659

TableS2 Frontier analysis of Smoking-Related Low Back Pain among WCBA

	location	year	val	SDI	frontier	eff_diff
401	Austria	2006	241.83488302583	0.812064609	2.11315436868562	239.721728657144
402	Austria	2007	239.53685726659	0.815480522	2.11434616016028	237.42251110643
403	Austria	2008	237.13074489871	0.818793658	2.11677326287061	235.013971635839
404	Austria	2009	235.1561737388	0.821541167	2.11445947359066	233.041714265209
405	Austria	2010	233.00338438308	0.824500109	2.1132103250825	230.890174057998
406	Austria	2011	231.78574253489	0.828001999	2.11403789971253	229.671704635177
407	Austria	2012	230.48787351651	0.831113309	2.11182706950936	228.376046447001
408	Austria	2013	228.63799160086	0.83367978	2.11149855236386	226.526493048496
409	Austria	2014	227.00021791245	0.835838577	2.11245748551128	224.887760426939
410	Austria	2015	225.67823228248	0.837579895	2.11621881667019	223.56201346581
411	Austria	2016	225.17780887174	0.83988379	2.11414179534316	223.063667076397
412	Austria	2017	225.76206630514	0.843173331	2.11276850949382	223.649297795646
413	Austria	2018	226.13969895737	0.84661447	2.11361326552531	224.026085691845
414	Austria	2019	225.82371988176	0.849862515	2.11048935587825	223.713230525882
415	Austria	2020	223.07627790391	0.852020385	2.1149996716	220.96127823231
416	Austria	2021	223.20428592982	0.853837004	2.11258661507391	221.091699314746
417	Azerbaijan	1990	14.52331978897	0.595986033	2.11477949389262	12.4085402950774
418	Azerbaijan	1991	14.85930281931	0.595807712	2.11060992669389	12.7486928926161
419	Azerbaijan	1992	15.36464265516	0.594651335	2.1113407694489	13.2533018857111
420	Azerbaijan	1993	16.02424553101	0.593542676	2.11206097230693	13.9121845587031
421	Azerbaijan	1994	16.86530120607	0.592088785	2.11319776274014	14.7521034433299
422	Azerbaijan	1995	17.7129262812	0.589441457	2.1136554876245	15.5992707935755
423	Azerbaijan	1996	18.57740517221	0.586315946	2.11284334612679	16.4645618260832
424	Azerbaijan	1997	19.40367802627	0.583221813	2.11367882289508	17.2899992033749
425	Azerbaijan	1998	20.12940923147	0.580392893	2.11338412900898	18.016025102461

TableS2 Frontier analysis of Smoking-Related Low Back Pain among WCBA

	location	year	val	SDI	frontier	eff_diff
426	Azerbaijan	1999	20.93811266336	0.579192506	2.11151286102657	18.8265998023334
427	Azerbaijan	2000	21.73850411815	0.580020573	2.11550320343057	19.6230009147194
428	Azerbaijan	2001	22.5989925297	0.582193708	2.11273244827426	20.4862600814257
429	Azerbaijan	2002	23.51524839096	0.585716143	2.11306019906212	21.4021881918979
430	Azerbaijan	2003	24.46879595409	0.590038705	2.11383841559494	22.3549575384951
431	Azerbaijan	2004	25.35452884255	0.594646085	2.11221058456724	23.2423182579828
432	Azerbaijan	2005	26.05663809979	0.602485193	2.11952277435348	23.9371153254365
433	Azerbaijan	2006	26.72078877456	0.613771097	2.11403035810176	24.6067584164582
434	Azerbaijan	2007	27.38652112939	0.626110926	2.11601378859556	25.2705073407944
435	Azerbaijan	2008	27.94272550154	0.636901109	2.11129583369371	25.8314296678463
436	Azerbaijan	2009	28.3739167092	0.645163341	2.11403887855477	26.2598778306452
437	Azerbaijan	2010	28.47531199852	0.652056507	2.11212133576739	26.3631906627526
438	Azerbaijan	2011	28.32678294412	0.657971629	2.11352635015942	26.2132565939606
439	Azerbaijan	2012	27.88823639411	0.662897002	2.11327901884413	25.7749573752659
440	Azerbaijan	2013	27.46289800249	0.667658779	2.1124648691372	25.3504331333528
441	Azerbaijan	2014	26.90027516026	0.672162741	2.11356516365447	24.7867099966055
442	Azerbaijan	2015	26.52562507302	0.676244911	2.11212737531078	24.4134976977092
443	Azerbaijan	2016	26.18730450179	0.679807643	2.11321896118029	24.0740855406097
444	Azerbaijan	2017	25.78880025317	0.683263204	2.11357262075902	23.675227632411
445	Azerbaijan	2018	25.44575067852	0.686561093	2.11124705757317	23.3345036209468
446	Azerbaijan	2019	25.17093560927	0.689834715	2.11425926577914	23.0566763434909
447	Azerbaijan	2020	25.12018196057	0.692605192	2.11159261105417	23.0085893495158
448	Azerbaijan	2021	25.02535220875	0.694851274	2.1119651435896	22.9133870651604
449	Bahamas	1990	29.73032723406	0.693509268	2.11207235712817	27.6182548769318
450	Bahamas	1991	30.01038636848	0.689126784	2.112453078282	27.897933290198

TableS2 Frontier analysis of Smoking-Related Low Back Pain among WCBA

	location	year	val	SDI	frontier	eff_diff
451	Bahamas	1992	30.4185225209	0.687343838	2.11144305725177	28.3070794636482
452	Bahamas	1993	30.93413195793	0.693816501	2.11146021312423	28.8226717448058
453	Bahamas	1994	31.48758178533	0.706819892	2.11215381324875	29.3754279720813
454	Bahamas	1995	32.02636337878	0.719381607	2.11159688463804	29.914766494142
455	Bahamas	1996	32.57447802474	0.728478844	2.11126700994317	30.4632110147968
456	Bahamas	1997	33.0732019491	0.734781215	2.11354300345023	30.9596589456498
457	Bahamas	1998	33.5239038596	0.739281293	2.11221653063238	31.4116873289676
458	Bahamas	1999	34.01866207226	0.741622848	2.1129519486735	31.9057101235865
459	Bahamas	2000	34.43387488824	0.742517946	2.11048484757351	32.3233900406665
460	Bahamas	2001	35.00734998561	0.743592882	2.11383521887103	32.893514766739
461	Bahamas	2002	35.57774852167	0.746250322	2.11142788182203	33.466320639848
462	Bahamas	2003	35.98597641234	0.749916714	2.1147318723445	33.8712445399955
463	Bahamas	2004	36.20010816324	0.753759643	2.11199810071093	34.0881100625291
464	Bahamas	2005	36.43228080478	0.756782661	2.11245969325047	34.3198211115295
465	Bahamas	2006	36.41719514849	0.759185939	2.11158973606913	34.3056054124209
466	Bahamas	2007	36.23192855192	0.761990997	2.11534437127939	34.1165841806406
467	Bahamas	2008	36.11410885421	0.765590503	2.11411743891589	33.9999914152941
468	Bahamas	2009	35.88029782599	0.769561674	2.11349213298076	33.7668056930092
469	Bahamas	2010	35.86534492413	0.77411981	2.11425281287923	33.7510921112508
470	Bahamas	2011	35.73547069454	0.778281891	2.11408314263932	33.6213875519007
471	Bahamas	2012	35.51707467509	0.781838639	2.11132770114184	33.4057469739482
472	Bahamas	2013	35.25345906121	0.784623911	2.11160561050892	33.1418534507011
473	Bahamas	2014	34.915064005	0.787253492	2.11288823242875	32.8021757725712
474	Bahamas	2015	34.60031831386	0.789956145	2.11482200543808	32.4854963084219
475	Bahamas	2016	34.33614373683	0.792738696	2.11255277182173	32.2235909650083

TableS2 Frontier analysis of Smoking-Related Low Back Pain among WCBA

	location	year	val	SDI	frontier	eff_diff
476	Bahamas	2017	33.99569680277	0.795347181	2.11070028649949	31.8849965162705
477	Bahamas	2018	33.79789915458	0.798179034	2.11242530099717	31.6854738535828
478	Bahamas	2019	33.64620393517	0.80103307	2.11453699966766	31.5316669355023
479	Bahamas	2020	33.62458983304	0.802948018	2.11451436351533	31.5100754695247
480	Bahamas	2021	33.52301665491	0.805020668	2.11420406090712	31.4088125940029
481	Bahrain	1990	39.04175011477	0.584578852	2.11253628243311	36.9292138323369
482	Bahrain	1991	39.03882343258	0.590596897	2.113080957255	36.925742475325
483	Bahrain	1992	38.82671285152	0.596040229	2.11401991705853	36.7126929344615
484	Bahrain	1993	38.67814281921	0.602165622	2.11474285628776	36.5633999629222
485	Bahrain	1994	38.63584112084	0.608133725	2.11341242657915	36.5224286942608
486	Bahrain	1995	38.57506591938	0.613850722	2.11206771343109	36.4629982059489
487	Bahrain	1996	38.23201479794	0.619485376	2.1126468143725	36.1193679835675
488	Bahrain	1997	37.7500321634	0.624596062	2.11270695407063	35.6373252093294
489	Bahrain	1998	37.52904503583	0.630738301	2.11077610758681	35.4182689282432
490	Bahrain	1999	37.46070891823	0.638083187	2.11409640344661	35.3466125147834
491	Bahrain	2000	37.6594363009	0.646750697	2.11530724474391	35.5441290561561
492	Bahrain	2001	37.92192876628	0.657196064	2.11625794850518	35.8056708177748
493	Bahrain	2002	37.45389490103	0.664901201	2.10952955439846	35.3443653466315
494	Bahrain	2003	36.66979197395	0.669564111	2.11216460710565	34.5576273668444
495	Bahrain	2004	35.83715718324	0.674147775	2.1162548356308	33.7209023476092
496	Bahrain	2005	35.5476615408	0.679790598	2.11705972566056	33.4306018151394
497	Bahrain	2006	35.73381587543	0.686144289	2.11344328266658	33.6203725927634
498	Bahrain	2007	35.6217560929	0.693045147	2.11380891711318	33.5079471757868
499	Bahrain	2008	35.38381370261	0.699806387	2.11499901655608	33.2688146860539
500	Bahrain	2009	35.40405688177	0.70488584	2.11374744949051	33.2903094322795

TableS2 Frontier analysis of Smoking-Related Low Back Pain among WCBA

	location	year	val	SDI	frontier	eff_diff
501	Bahrain	2010	35.89626050949	0.70790262	2.11389748406154	33.7823630254285
502	Bahrain	2011	36.90801386745	0.708958739	2.11188883458013	34.7961250328699
503	Bahrain	2012	37.36081643699	0.710362418	2.11269443205875	35.2481220049312
504	Bahrain	2013	37.33313795002	0.713343156	2.1152469781578	35.2178909718622
505	Bahrain	2014	37.15247579487	0.71676806	2.11244968057562	35.0400261142944
506	Bahrain	2015	37.05617008294	0.719926605	2.1114058776608	34.9447642052792
507	Bahrain	2016	37.21568751961	0.723611297	2.11057597965872	35.1051115399513
508	Bahrain	2017	37.54403866746	0.729323103	2.11307188601966	35.4309667814403
509	Bahrain	2018	37.84084191387	0.736192583	2.11283693696242	35.7280049769076
510	Bahrain	2019	37.94241687302	0.742847804	2.11251837320662	35.8298984998134
511	Bahrain	2020	37.9549436852	0.748103083	2.11625935874012	35.8386843264599
512	Bahrain	2021	37.77517945685	0.753043204	2.11324836877494	35.6619310880751
513	Bangladesh	1990	14.29271297166	0.228548934	2.72911379385	11.56359917781
514	Bangladesh	1991	14.00734758533	0.237207413	2.63258563630015	11.3747619490298
515	Bangladesh	1992	13.72488746895	0.245247956	2.55138022521258	11.1735072437374
516	Bangladesh	1993	13.48589401445	0.25209072	2.52521280087514	10.9606812135749
517	Bangladesh	1994	13.2712948464	0.258997294	2.50845266432552	10.7628421820745
518	Bangladesh	1995	13.08646472566	0.265293918	2.48023666419955	10.6062280614604
519	Bangladesh	1996	12.89087606515	0.271177781	2.49917340777508	10.3917026573749
520	Bangladesh	1997	12.67862258705	0.27777328	2.45813293127638	10.2204896557736
521	Bangladesh	1998	12.5050410581	0.28451594	2.44137191369	10.06366914441
522	Bangladesh	1999	12.34941025238	0.290951431	2.42799041566967	9.92141983671033
523	Bangladesh	2000	12.3108549217	0.297251976	2.40002164217435	9.91083327952565
524	Bangladesh	2001	12.39807427126	0.303188546	2.3799660824903	10.0181081887697
525	Bangladesh	2002	12.51259767029	0.308640093	2.38426566823052	10.1283320020595

TableS2 Frontier analysis of Smoking-Related Low Back Pain among WCBA

	location	year	val	SDI	frontier	eff_diff
526	Bangladesh	2003	12.63631626904	0.31436004	2.36957044438243	10.2667458246576
527	Bangladesh	2004	12.7868508698	0.320922851	2.33817812380729	10.4486727459927
528	Bangladesh	2005	12.97237263559	0.32809051	2.32591846715242	10.6464541684376
529	Bangladesh	2006	13.23956814384	0.335690598	2.28798276817379	10.9515853756662
530	Bangladesh	2007	13.63724679136	0.343932546	2.17827161032408	11.4589751810359
531	Bangladesh	2008	14.11626764912	0.352520033	2.11651091233051	11.9997567367895
532	Bangladesh	2009	14.68152849467	0.361481513	2.11295727414988	12.5685712205201
533	Bangladesh	2010	15.26032398195	0.370598956	2.11270015173988	13.1476238302101
534	Bangladesh	2011	16.02270105894	0.380124886	2.1144620435712	13.9082390153688
535	Bangladesh	2012	17.05182782993	0.390623552	2.11375245662186	14.9380753733081
536	Bangladesh	2013	18.16979429226	0.401706478	2.11471537705029	16.0550789152097
537	Bangladesh	2014	19.22323592119	0.413263169	2.11130334492618	17.1119325762638
538	Bangladesh	2015	20.03032394405	0.425482121	2.11820175563803	17.912122188412
539	Bangladesh	2016	20.43952153463	0.437709906	2.11313553851632	18.3263859961137
540	Bangladesh	2017	20.59859159993	0.449752413	2.1112652522966	18.4873263476334
541	Bangladesh	2018	20.5977709271	0.461717749	2.11577588929122	18.4819950378088
542	Bangladesh	2019	20.70654150775	0.473264998	2.1134810174315	18.5930604903185
543	Bangladesh	2020	20.83798508822	0.483079169	2.11227398511929	18.7257111031007
544	Bangladesh	2021	20.94375129667	0.492420885	2.11728928700972	18.8264620096603
545	Barbados	1990	35.90571845032	0.653582517	2.11282781162046	33.7928906386995
546	Barbados	1991	36.87822532886	0.656654073	2.11385811451525	34.7643672143447
547	Barbados	1992	37.73364177146	0.662446635	2.1157258099183	35.6179159615417
548	Barbados	1993	38.68890095167	0.668815012	2.11346907551791	36.5754318761521
549	Barbados	1994	39.48745150003	0.673409365	2.11330304167486	37.3741484583551
550	Barbados	1995	40.23740615718	0.676012247	2.11278864061672	38.1246175165633

TableS2 Frontier analysis of Smoking-Related Low Back Pain among WCBA

	location	year	val	SDI	frontier	eff_diff
551	Barbados	1996	40.98253075913	0.677193054	2.11124767466611	38.8712830844639
552	Barbados	1997	41.72814979298	0.678644826	2.11253648481337	39.6156133081666
553	Barbados	1998	42.34033049729	0.679827167	2.11213671384468	40.2281937834453
554	Barbados	1999	42.97140046978	0.680398459	2.11548974237416	40.8559107274058
555	Barbados	2000	43.52562459393	0.681304199	2.1126143750203	41.4130102189097
556	Barbados	2001	43.82722630429	0.683329389	2.11146294727493	41.7157633570151
557	Barbados	2002	43.96676150907	0.687807576	2.11093389383611	41.8558276152339
558	Barbados	2003	43.78374694982	0.693235962	2.11372324606824	41.6700237037518
559	Barbados	2004	43.48036426273	0.698029703	2.11453457008409	41.3658296926459
560	Barbados	2005	43.02213433779	0.701510049	2.11355189462288	40.9085824431671
561	Barbados	2006	42.38524903968	0.703856186	2.11495213503833	40.2702969046417
562	Barbados	2007	41.4094890902	0.705871649	2.11361617736338	39.2958729128366
563	Barbados	2008	40.41066500619	0.708514495	2.11174299593879	38.2989220102512
564	Barbados	2009	39.49094625704	0.712143362	2.11101917741972	37.3799270796203
565	Barbados	2010	38.86255412245	0.716208546	2.11357746057468	36.7489766618753
566	Barbados	2011	38.18914434113	0.720060687	2.112486614721	36.076657726409
567	Barbados	2012	37.60635376257	0.724162458	2.11110725843515	35.4952465041348
568	Barbados	2013	37.07458913499	0.72782617	2.11407415157492	34.9605149834151
569	Barbados	2014	36.49296745925	0.730383287	2.11116608292625	34.3818013763238
570	Barbados	2015	36.01042535135	0.732602993	2.1122643999837	33.8981609513663
571	Barbados	2016	35.49291596176	0.734912535	2.11469074090111	33.3782252208589
572	Barbados	2017	35.06193416653	0.737157255	2.1151553350294	32.9467788315006
573	Barbados	2018	34.74403518606	0.739531437	2.11140537232276	32.6326298137372
574	Barbados	2019	34.55305137254	0.742239309	2.11029041683179	32.4427609557082
575	Barbados	2020	34.29920595981	0.744366646	2.11202570123946	32.1871802585705

TableS2 Frontier analysis of Smoking-Related Low Back Pain among WCBA

	location	year	val	SDI	frontier	eff_diff
576	Barbados	2021	34.33007283129	0.746748764	2.11587519624065	32.2141976350494
577	Belarus	1990	192.07030670905	0.622446576	2.11339118195952	189.95691552709
578	Belarus	1991	193.68690045546	0.627038267	2.11199006722021	191.57491038824
579	Belarus	1992	195.52014490143	0.632891737	2.11292747157453	193.407217429855
580	Belarus	1993	197.56815651865	0.639394169	2.11509616714041	195.45306035151
581	Belarus	1994	199.79810615631	0.644652781	2.11397738885247	197.684128767458
582	Belarus	1995	201.23435310444	0.648959504	2.11305780080282	199.121295303637
583	Belarus	1996	202.90324420253	0.652855221	2.11557679241965	200.78766741011
584	Belarus	1997	202.74086597861	0.655627296	2.11084295910597	200.630023019504
585	Belarus	1998	201.77892289593	0.657246467	2.11848502576324	199.660437870167
586	Belarus	1999	200.6229912477	0.66017101	2.11375595806521	198.509235289635
587	Belarus	2000	199.26378031135	0.664705822	2.11321810608462	197.150562205265
588	Belarus	2001	198.48612912218	0.671333269	2.11456767523259	196.371561446947
589	Belarus	2002	197.55683305379	0.678121782	2.11217953501462	195.444653518775
590	Belarus	2003	196.58960549908	0.684868556	2.11683732966394	194.472768169416
591	Belarus	2004	196.14747761574	0.692502135	2.11170313775812	194.035774477982
592	Belarus	2005	195.39312854841	0.698985483	2.11323108078013	193.27989746763
593	Belarus	2006	194.95436520635	0.705119103	2.11314051901522	192.841224687335
594	Belarus	2007	194.74539192991	0.711496562	2.11147721755359	192.633914712356
595	Belarus	2008	194.63162465103	0.719120871	2.11645170661605	192.515172944414
596	Belarus	2009	194.77953567573	0.727292784	2.11512958591	192.66440608982
597	Belarus	2010	194.79156360784	0.734954626	2.11584979501027	192.67571381283
598	Belarus	2011	194.29756903314	0.739504326	2.11555127046286	192.182017762677
599	Belarus	2012	193.38980493728	0.743468837	2.1123911119573	191.277413825323
600	Belarus	2013	192.20208055777	0.748529323	2.111055842705	190.091024715065

TableS2 Frontier analysis of Smoking-Related Low Back Pain among WCBA

	location	year	val	SDI	frontier	eff_diff
601	Belarus	2014	191.70593236647	0.75351148	2.11371404411273	189.592218322357
602	Belarus	2015	191.96822956195	0.758205743	2.11468864956892	189.853540912381
603	Belarus	2016	192.86493972086	0.764328222	2.11261970936938	190.752320011491
604	Belarus	2017	194.17630440761	0.771131466	2.110879899503	192.065424508107
605	Belarus	2018	195.90918121842	0.776568957	2.11293706639494	193.796244152025
606	Belarus	2019	198.12299759207	0.780508019	2.11037729800816	196.012620294062
607	Belarus	2020	200.46808895862	0.782381565	2.11635461175667	198.351734346863
608	Belarus	2021	199.48074110382	0.784484711	2.11175246129773	197.368988642522
609	Belgium	1990	270.5080000434	0.737390656	2.11186543798671	268.396134605413
610	Belgium	1991	274.57142883929	0.742173074	2.11236957915847	272.459059260131
611	Belgium	1992	278.48316549041	0.748012299	2.11132612126015	276.37183936915
612	Belgium	1993	281.80939687678	0.753685087	2.1124662616237	279.696930615156
613	Belgium	1994	284.90779005237	0.758903638	2.11480570618687	282.792984346183
614	Belgium	1995	287.54639543682	0.763170401	2.11354911479222	285.432846322028
615	Belgium	1996	289.38516396902	0.766826075	2.11316159478716	287.272002374233
616	Belgium	1997	289.93248770518	0.770813953	2.11324089413116	287.819246811049
617	Belgium	1998	290.45660815321	0.774228268	2.11750732613812	288.339100827072
618	Belgium	1999	290.65025303526	0.77704104	2.11438664684213	288.535866388418
619	Belgium	2000	290.22863892188	0.780396331	2.11202700539611	288.116611916484
620	Belgium	2001	289.71233598738	0.784713043	2.1118664102869	287.600469577093
621	Belgium	2002	288.40837037053	0.788836054	2.11305809645179	286.295312274078
622	Belgium	2003	285.94013986401	0.792014384	2.11362354001961	283.82651632399
623	Belgium	2004	282.78440412007	0.795224936	2.11634736242737	280.668056757643
624	Belgium	2005	280.1705388591	0.798497236	2.11194614758867	278.058592711511
625	Belgium	2006	276.551852091	0.801572763	2.11242559100979	274.43942649999

TableS2 Frontier analysis of Smoking-Related Low Back Pain among WCBA

	location	year	val	SDI	frontier	eff_diff
626	Belgium	2007	272.25131084366	0.804612664	2.11357887695956	270.1377319667
627	Belgium	2008	267.41083990957	0.807757945	2.11192827535103	265.298911634219
628	Belgium	2009	263.03094387303	0.811055825	2.11293357458013	260.91801029845
629	Belgium	2010	259.40784773113	0.815030207	2.11497233599328	257.292875395137
630	Belgium	2011	255.69738740739	0.819470362	2.11290180419478	253.584485603195
631	Belgium	2012	251.81619641696	0.824226016	2.11334942691899	249.702846990041
632	Belgium	2013	248.15750163425	0.828619832	2.1148654969994	246.042636137251
633	Belgium	2014	244.24184235796	0.832425052	2.11632866300525	242.125513694955
634	Belgium	2015	241.44825325986	0.8358784	2.11486531062958	239.33338794923
635	Belgium	2016	239.06763765092	0.83946294	2.11325064906594	236.954387001854
636	Belgium	2017	237.26777404074	0.843212044	2.1168631824955	235.150910858244
637	Belgium	2018	235.91893972522	0.846617625	2.11323704626743	233.805702678953
638	Belgium	2019	234.96526803019	0.849498395	2.11209128024114	232.853176749949
639	Belgium	2020	231.15282798995	0.851346649	2.11480790516012	229.03802008479
640	Belgium	2021	231.53015917972	0.853654016	2.11184458401032	229.41831459571
641	Belize	1990	22.80991030518	0.423726992	2.10953371621693	20.7003765889631
642	Belize	1991	22.67989816302	0.433834392	2.11157867706237	20.5683194859576
643	Belize	1992	22.52989980229	0.444198507	2.11605485503969	20.4138449472503
644	Belize	1993	22.44689899604	0.455258577	2.11576267457091	20.3311363214691
645	Belize	1994	22.43655199619	0.465729308	2.11465726458108	20.3218947316089
646	Belize	1995	22.37478640288	0.475278511	2.113177056552	20.261609346328
647	Belize	1996	22.33659693204	0.482702165	2.11150494422892	20.2250919878111
648	Belize	1997	22.27544570355	0.488020129	2.11843487878398	20.157010824766
649	Belize	1998	22.19105374917	0.491819335	2.11076428916859	20.0802894600014
650	Belize	1999	22.19350606856	0.495070196	2.1136786434346	20.0798274251254

TableS2 Frontier analysis of Smoking-Related Low Back Pain among WCBA

	location	year	val	SDI	frontier	eff_diff
651	Belize	2000	22.29075554071	0.49864373	2.11192537914278	20.1788301615672
652	Belize	2001	22.45028193073	0.502399434	2.11514412058706	20.3351378101429
653	Belize	2002	22.65692097605	0.506871655	2.11246510158897	20.544455874461
654	Belize	2003	22.85094744579	0.512679459	2.11395116499667	20.7369962807933
655	Belize	2004	23.03415588998	0.519697333	2.11453471740032	20.9196211725797
656	Belize	2005	23.24982399667	0.527231062	2.11587616206921	21.1339478346008
657	Belize	2006	23.48695150999	0.534889621	2.11500780122507	21.3719437087649
658	Belize	2007	23.77651042028	0.541900694	2.11364260357423	21.6628678167058
659	Belize	2008	24.07405050833	0.548093223	2.11310712196646	21.9609433863635
660	Belize	2009	24.38122893311	0.553465869	2.11172771527962	22.2695012178304
661	Belize	2010	24.64674655485	0.558546893	2.11314933103788	22.5335972238121
662	Belize	2011	24.90525342265	0.563440363	2.11264502016561	22.7926084024844
663	Belize	2012	25.22590747656	0.568456236	2.11224688003053	23.1136605965295
664	Belize	2013	25.4713839159	0.573236457	2.11101799337667	23.3603659225233
665	Belize	2014	25.73599605065	0.578091326	2.11102949545288	23.6249665551971
666	Belize	2015	25.9438225697	0.583279094	2.11524397654639	23.8285785931536
667	Belize	2016	26.12221265764	0.588275912	2.11386894570451	24.0083437119355
668	Belize	2017	26.25443991723	0.59333729	2.11288959401042	24.1415503232196
669	Belize	2018	26.40627814011	0.598512015	2.11136785826314	24.2949102818469
670	Belize	2019	26.57330118162	0.603351601	2.11159718618013	24.4617039954399
671	Belize	2020	26.63861554888	0.607060046	2.11566320458971	24.5229523442903
672	Belize	2021	26.80074330373	0.610229002	2.11301285810435	24.6877304456257
673	Benin	1990	13.26501220002	0.218907154	2.86875964509241	10.3962525549276
674	Benin	1991	13.4058052873	0.222539019	2.84664956490608	10.5591557223939
675	Benin	1992	13.52936693538	0.226323465	2.76908086673684	10.7602860686432

TableS2 Frontier analysis of Smoking-Related Low Back Pain among WCBA

	location	year	val	SDI	frontier	eff_diff
676	Benin	1993	13.7185017946	0.230312594	2.57591592992946	11.1425858646705
677	Benin	1994	13.87398725699	0.234032036	2.63837877753638	11.2356084794536
678	Benin	1995	13.92656521233	0.238009696	2.62103005938645	11.3055351529435
679	Benin	1996	14.22818856904	0.241895323	2.57874973271578	11.6494388363242
680	Benin	1997	14.63674566516	0.245731534	2.56634766557569	12.0703979995843
681	Benin	1998	14.84358658347	0.249376451	2.54676796487839	12.2968186185916
682	Benin	1999	14.93809287935	0.253215341	2.53314824301288	12.4049446363371
683	Benin	2000	15.22438632007	0.257380681	2.54567864757224	12.6787076724978
684	Benin	2001	15.07996982789	0.261388011	2.51348484036169	12.5664849875283
685	Benin	2002	14.8045871146	0.265417458	2.48517932995189	12.3194077846481
686	Benin	2003	14.4781575542	0.269544281	2.4783814021168	11.9997761520832
687	Benin	2004	14.09611226211	0.273941145	2.45590268066673	11.6402095814433
688	Benin	2005	13.76673149653	0.27840675	2.46033721452427	11.3063942820057
689	Benin	2006	13.27013257758	0.282933936	2.45468731622671	10.8154452613533
690	Benin	2007	12.57389461595	0.287673055	2.42537962940867	10.1485149865413
691	Benin	2008	11.78045598602	0.292627446	2.43310680669613	9.34734917932387
692	Benin	2009	10.98806348351	0.297702227	2.39810221194265	8.58996127156735
693	Benin	2010	10.3338001518	0.302809008	2.40140224187145	7.93239790992855
694	Benin	2011	9.8132974876	0.307925538	2.38536813577598	7.42792935182402
695	Benin	2012	9.27629187187	0.313373588	2.36079841634507	6.91549345552493
696	Benin	2013	8.76470200833	0.319318688	2.34231687168559	6.42238513664441
697	Benin	2014	8.35056139513	0.325727272	2.31910893551969	6.03145245961031
698	Benin	2015	7.99493393717	0.33221635	2.30539764971662	5.68953628745338
699	Benin	2016	7.74063422841	0.338763921	2.27063852384903	5.46999570456097
700	Benin	2017	7.5168063353	0.345606759	2.15123359618386	5.36557273911614

TableS2 Frontier analysis of Smoking-Related Low Back Pain among WCBA

	location	year	val	SDI	frontier	eff_diff
701	Benin	2018	7.32065210986	0.352652696	2.13158819182872	5.18906391803128
702	Benin	2019	7.13836111334	0.360031163	2.11188870857444	5.02647240476555
703	Benin	2020	6.99034081256	0.366964487	2.11470617793902	4.87563463462098
704	Benin	2021	6.80216838226	0.373486574	2.11382601642246	4.68834236583754
705	Bermuda	1990	75.51403477442	0.696451196	2.11736596089394	73.3966688135261
706	Bermuda	1991	77.06479746739	0.700276851	2.11682766850143	74.9479697988886
707	Bermuda	1992	78.86486531968	0.703984095	2.11237359694462	76.7524917227354
708	Bermuda	1993	80.7515388344	0.707678222	2.11558913271036	78.6359497016897
709	Bermuda	1994	82.41781826795	0.711068998	2.11249968004205	80.3053185879079
710	Bermuda	1995	83.96392191423	0.714468828	2.11231539617767	81.8516065180523
711	Bermuda	1996	85.29468722362	0.717831671	2.11425646763689	83.1804307559831
712	Bermuda	1997	86.48887521384	0.721642813	2.11292365882937	84.3759515550106
713	Bermuda	1998	87.56684335973	0.725853528	2.11434038764875	85.4525029720812
714	Bermuda	1999	88.64989001646	0.730539823	2.11369369709522	86.5361963193648
715	Bermuda	2000	90.08589665019	0.735996502	2.11342193693403	87.972474713256
716	Bermuda	2001	90.99462251234	0.741918908	2.10935681014051	88.8852657021995
717	Bermuda	2002	91.85113083077	0.747544503	2.1140891678413	89.7370416629287
718	Bermuda	2003	92.65563481548	0.753305163	2.11493910442381	90.5406957110562
719	Bermuda	2004	93.11301194242	0.759008885	2.116462842811	90.996549099609
720	Bermuda	2005	93.51357138553	0.764690903	2.11472180033767	91.3988495851923
721	Bermuda	2006	93.28083239454	0.77059677	2.11287433257785	91.1679580619622
722	Bermuda	2007	93.35299787666	0.776114264	2.11328335281458	91.2397145238454
723	Bermuda	2008	93.09547142226	0.780358325	2.11293683586683	90.9825345863932
724	Bermuda	2009	92.77307767592	0.784759107	2.11173017324838	90.6613475026716
725	Bermuda	2010	92.48877986514	0.789181467	2.11178109576813	90.3769987693719

TableS2 Frontier analysis of Smoking-Related Low Back Pain among WCBA

	location	year	val	SDI	frontier	eff_diff
726	Bermuda	2011	92.69791211069	0.793380524	2.11520389624741	90.5827082144426
727	Bermuda	2012	92.64364676225	0.79748487	2.11284580114213	90.5308009611079
728	Bermuda	2013	92.83737149434	0.801388201	2.11074796271135	90.7266235316286
729	Bermuda	2014	92.93387082253	0.804923407	2.1137053620486	90.8201654604814
730	Bermuda	2015	93.17597597189	0.807998795	2.11176980102444	91.0642061708655
731	Bermuda	2016	93.52905741619	0.810550842	2.11312037563137	91.4159370405586
732	Bermuda	2017	93.87016384561	0.812807229	2.11302815315303	91.757135692457
733	Bermuda	2018	93.90119006706	0.814892802	2.11644847511768	91.7847415919423
734	Bermuda	2019	94.0842615566	0.817000338	2.11239021759531	91.9718713390047
735	Bermuda	2020	94.08608036341	0.81920342	2.11387545740451	91.9722049060055
736	Bermuda	2021	94.21677592004	0.821365422	2.11585482264125	92.1009210973987
737	Bhutan	1990	36.38569106985	0.21503985	3.49890910125295	32.886781968597
738	Bhutan	1991	36.3127575945	0.22131244	2.8999790110556	33.4127785834444
739	Bhutan	1992	36.78731005206	0.228148272	2.68358439573528	34.1037256563247
740	Bhutan	1993	37.96439951219	0.236004687	2.61645292027864	35.3479465919114
741	Bhutan	1994	38.53792335153	0.244331246	2.56826789981851	35.9696554517115
742	Bhutan	1995	38.52317325413	0.253291957	2.54669309142903	35.976480162701
743	Bhutan	1996	38.25860068196	0.262362746	2.48097456195219	35.7776261200078
744	Bhutan	1997	37.82228790097	0.271487422	2.49977325452087	35.3225146464491
745	Bhutan	1998	37.39374363447	0.280440384	2.46459301490586	34.9291506195641
746	Bhutan	1999	36.96187363852	0.289510488	2.43171751709041	34.5301561214296
747	Bhutan	2000	36.58843523253	0.298804875	2.39518669495957	34.1932485375704
748	Bhutan	2001	36.16507672773	0.308200652	2.38411741733311	33.7809593103969
749	Bhutan	2002	35.70688886639	0.317943536	2.34203617198859	33.3648526944014
750	Bhutan	2003	35.37458752581	0.327670422	2.32374351867408	33.0508440071359

TableS2 Frontier analysis of Smoking-Related Low Back Pain among WCBA

	location	year	val	SDI	frontier	eff_diff
751	Bhutan	2004	35.31558093293	0.337286495	2.27408983520239	33.0414910977276
752	Bhutan	2005	35.45489862954	0.346998578	2.13985546385887	33.3150431656811
753	Bhutan	2006	35.70280448952	0.356824209	2.11609802532229	33.5867064641977
754	Bhutan	2007	35.95306873069	0.367560498	2.11458652581	33.83848220488
755	Bhutan	2008	36.06469141198	0.378233543	2.11714449729304	33.947546914687
756	Bhutan	2009	36.20687396766	0.388977162	2.1131351350959	34.0937388325641
757	Bhutan	2010	36.19686362556	0.399751936	2.11602128841594	34.0808423371441
758	Bhutan	2011	36.16188773616	0.410149249	2.11474397315707	34.0471437630029
759	Bhutan	2012	35.96091037733	0.419443216	2.1120156753478	33.8488947019822
760	Bhutan	2013	35.98171575526	0.427790675	2.11533151983604	33.866384235424
761	Bhutan	2014	36.17575581734	0.43496927	2.11692998372258	34.0588258336174
762	Bhutan	2015	36.58520850107	0.441810748	2.11379635494608	34.4714121461239
763	Bhutan	2016	37.1862295458	0.448401855	2.11568826940061	35.0705412763994
764	Bhutan	2017	37.82512132394	0.454280497	2.11424031203304	35.710881011907
765	Bhutan	2018	38.62237003991	0.459624711	2.11228876060542	36.5100812793046
766	Bhutan	2019	39.58427824074	0.464633353	2.11316197176035	37.4711162689797
767	Bhutan	2020	40.31659858106	0.468714479	2.11447206553253	38.2021265155275
768	Bhutan	2021	41.23848087577	0.473062378	2.11205100327595	39.1264298724941
769	Bolivia	1990	18.82075436072	0.423917961	2.1128675110918	16.7078868496282
770	Bolivia	1991	18.31201481988	0.429397795	2.11173948121068	16.2002753386693
771	Bolivia	1992	17.9157069957	0.434835542	2.11177358655395	15.8039334091461
772	Bolivia	1993	17.47006840822	0.440601646	2.11313041122305	15.356937996997
773	Bolivia	1994	17.06568699928	0.4468225	2.11709335146419	14.9485936478158
774	Bolivia	1995	16.67946149253	0.453479243	2.11272129668171	14.5667401958483
775	Bolivia	1996	16.37276054034	0.460220669	2.11142137347358	14.2613391668664

TableS2 Frontier analysis of Smoking-Related Low Back Pain among WCBA

	location	year	val	SDI	frontier	eff_diff
776	Bolivia	1997	16.05580689097	0.466982897	2.11960413721945	13.9362027537505
777	Bolivia	1998	15.80299564545	0.473796201	2.11494734876958	13.6880482966804
778	Bolivia	1999	15.56442934857	0.48019431	2.11135728805772	13.4530720605123
779	Bolivia	2000	15.45985387825	0.486327903	2.112711056414	13.347142821836
780	Bolivia	2001	15.36744101014	0.492030764	2.11644927524281	13.2509917348972
781	Bolivia	2002	15.31594778717	0.497348837	2.11121519491364	13.2047325922564
782	Bolivia	2003	15.27818679673	0.502365803	2.11218290766324	13.1660038890668
783	Bolivia	2004	15.28800864984	0.507273036	2.11350567550711	13.1745029743329
784	Bolivia	2005	15.3217811619	0.512166491	2.1169900063313	13.2047911555687
785	Bolivia	2006	15.42661963126	0.517146194	2.11159133799069	13.3150282932693
786	Bolivia	2007	15.55557436657	0.52194613	2.11392517564683	13.4416491909232
787	Bolivia	2008	15.71895917538	0.52694375	2.11288670607736	13.6060724693026
788	Bolivia	2009	15.89956871144	0.53179244	2.11004477028373	13.7895239411563
789	Bolivia	2010	16.05978154005	0.537009068	2.11240268948176	13.9473788505682
790	Bolivia	2011	16.16140029561	0.542680745	2.11364144096084	14.0477588546492
791	Bolivia	2012	16.27865948936	0.548615482	2.11294214652107	14.1657173428389
792	Bolivia	2013	16.39282853247	0.554788216	2.11260803359398	14.280220498876
793	Bolivia	2014	16.5197854575	0.560934581	2.11286150194421	14.4069239555558
794	Bolivia	2015	16.64817273529	0.567000898	2.11224516812725	14.5359275671627
795	Bolivia	2016	16.74238609636	0.573080316	2.11414081699792	14.6282452793621
796	Bolivia	2017	16.81107976334	0.579241285	2.1118976206983	14.6991821426417
797	Bolivia	2018	16.90383490365	0.585184106	2.1108849158832	14.7929499877668
798	Bolivia	2019	17.0524504174	0.590692043	2.11255095901479	14.9398994583852
799	Bolivia	2020	17.02204216902	0.594854443	2.10929235691315	14.9127498121069
800	Bolivia	2021	17.2554596077	0.599010799	2.11231590556903	15.143143702131

TableS2 Frontier analysis of Smoking-Related Low Back Pain among WCBA

	location	year	val	SDI	frontier	eff_diff
801	Bosnia and Herzegovina	1990	269.60411421464	0.54113254	2.11677903841286	267.487335176227
802	Bosnia and Herzegovina	1991	273.52817364279	0.541346251	2.11510074748818	271.413072895302
803	Bosnia and Herzegovina	1992	278.35452632251	0.539303227	2.11479363275814	276.239732689752
804	Bosnia and Herzegovina	1993	287.03446243909	0.536031882	2.11645025337779	284.918012185712
805	Bosnia and Herzegovina	1994	299.81690625311	0.53385339	2.11527876836672	297.701627484743
806	Bosnia and Herzegovina	1995	313.06773560594	0.534025536	2.11635829459222	310.951377311348
807	Bosnia and Herzegovina	1996	325.61608619411	0.545549215	2.11326372225722	323.502822471853
808	Bosnia and Herzegovina	1997	335.81293967313	0.563565415	2.11191913410765	333.701020539022
809	Bosnia and Herzegovina	1998	340.20940797746	0.581829515	2.1126502240717	338.096757753388
810	Bosnia and Herzegovina	1999	339.48308324961	0.597997469	2.11519878477354	337.367884464836
811	Bosnia and Herzegovina	2000	340.68368024472	0.610950685	2.11692896925153	338.566751275468
812	Bosnia and Herzegovina	2001	343.67088324105	0.621989464	2.1146886193619	341.556194621688
813	Bosnia and Herzegovina	2002	348.43418169035	0.632075518	2.11174105522114	346.322440635129
814	Bosnia and Herzegovina	2003	352.14716423322	0.640635102	2.11665531677653	350.030508916443
815	Bosnia and Herzegovina	2004	355.32430254489	0.647793732	2.11194334177819	353.212359203112
816	Bosnia and Herzegovina	2005	356.87528490092	0.653859137	2.11089378404333	354.764391116877
817	Bosnia and Herzegovina	2006	363.65930228037	0.659813662	2.112371386855	361.546930893515
818	Bosnia and Herzegovina	2007	374.49236272389	0.665578735	2.11386010608708	372.378502617803
819	Bosnia and Herzegovina	2008	383.68928026516	0.671100814	2.1124015676804	381.57687869748
820	Bosnia and Herzegovina	2009	390.61527962507	0.6760329	2.11387101691013	388.50140860816
821	Bosnia and Herzegovina	2010	395.67232719103	0.680824196	2.11451775442603	393.557809436604
822	Bosnia and Herzegovina	2011	397.91868600667	0.685399264	2.11507217741286	395.803613829257
823	Bosnia and Herzegovina	2012	400.40294402882	0.689506566	2.11172997741476	398.291214051405
824	Bosnia and Herzegovina	2013	402.19987174977	0.693508711	2.11151203644908	400.088359713321
825	Bosnia and Herzegovina	2014	405.28953235059	0.697031893	2.11184181062662	403.177690539963

TableS2 Frontier analysis of Smoking-Related Low Back Pain among WCBA

	location	year	val	SDI	frontier	eff_diff
826	Bosnia and Herzegovina	2015	408.13204126909	0.700446047	2.11240049274177	406.019640776348
827	Bosnia and Herzegovina	2016	413.15477795576	0.704079344	2.11133909332954	411.04343886243
828	Bosnia and Herzegovina	2017	419.12251947318	0.708053431	2.11594316848382	417.006576304696
829	Bosnia and Herzegovina	2018	425.24854695006	0.712347302	2.11261043799517	423.135936512065
830	Bosnia and Herzegovina	2019	428.69207251623	0.716857125	2.11277723313808	426.579295283092
831	Bosnia and Herzegovina	2020	427.27895397535	0.720202056	2.11234302635514	425.166610948995
832	Bosnia and Herzegovina	2021	425.30869318157	0.723077893	2.11375972816413	423.194933453406
833	Botswana	1990	31.0146189107	0.418077748	2.11103377781743	28.9035851328826
834	Botswana	1991	30.78075470775	0.429752286	2.11383271157721	28.6669219961728
835	Botswana	1992	30.59757718889	0.440490843	2.1099446088695	28.4876325800205
836	Botswana	1993	30.31269673418	0.450523614	2.11288849886547	28.1998082353145
837	Botswana	1994	30.20677130129	0.459954967	2.11387564608284	28.0928956552072
838	Botswana	1995	30.03465426138	0.469266513	2.11285988364974	27.9217943777303
839	Botswana	1996	30.06972307188	0.478588352	2.11416065192708	27.9555624199529
840	Botswana	1997	29.9460321969	0.487767857	2.11320583256025	27.8328263643397
841	Botswana	1998	29.7659559948	0.496193622	2.11336716233	27.65258883247
842	Botswana	1999	29.60797201641	0.504978826	2.11113636849462	27.4968356479154
843	Botswana	2000	29.57069885363	0.513498223	2.11295462949548	27.4577442241345
844	Botswana	2001	29.2846302909	0.521370182	2.11425018655075	27.1703801043493
845	Botswana	2002	28.98499701717	0.529252964	2.11318164634343	26.8718153708266
846	Botswana	2003	28.69546879826	0.537000179	2.11668896131123	26.5787798369488
847	Botswana	2004	28.41849080501	0.544349223	2.11270967294164	26.3057811320684
848	Botswana	2005	28.28794346123	0.55170697	2.11305789567812	26.1748855655519
849	Botswana	2006	28.07325818609	0.55922162	2.11593984048338	25.9573183456066
850	Botswana	2007	27.81730433514	0.566702496	2.11354950033549	25.7037548348045

TableS2 Frontier analysis of Smoking-Related Low Back Pain among WCBA

	location	year	val	SDI	frontier	eff_diff
851	Botswana	2008	27.59205110651	0.573883805	2.11168293495712	25.4803681715529
852	Botswana	2009	27.34133217143	0.579696412	2.11355864435933	25.2277735270707
853	Botswana	2010	27.19511341643	0.58582415	2.11482199319127	25.0802914232387
854	Botswana	2011	27.12259034752	0.591985457	2.11241764228778	25.0101727052322
855	Botswana	2012	27.07465223615	0.597739553	2.11573957559307	24.9589126605569
856	Botswana	2013	27.0826833446	0.604064731	2.11294861710384	24.9697347274962
857	Botswana	2014	27.18072652764	0.610086049	2.11252289786041	25.0682036297796
858	Botswana	2015	27.39998407533	0.615411124	2.11364773189886	25.2863363434311
859	Botswana	2016	27.83194310328	0.620797575	2.11208643116218	25.7198566721178
860	Botswana	2017	28.15626690114	0.625704953	2.11485317161514	26.0414137295249
861	Botswana	2018	28.5324199102	0.630606841	2.11471738245087	26.4177025277491
862	Botswana	2019	28.84967648113	0.635324093	2.11240547780493	26.7372710033251
863	Botswana	2020	29.09559883533	0.639275298	2.11569797906055	26.9799008562694
864	Botswana	2021	29.30736730431	0.642721629	2.11000390228582	27.1973634020242
865	Brazil	1990	196.74988554213	0.500070509	2.11650859418895	194.633376947941
866	Brazil	1991	198.38229872062	0.504784798	2.11561043838462	196.266688282235
867	Brazil	1992	199.45937297976	0.508539943	2.11123286925322	197.348140110507
868	Brazil	1993	200.20708396838	0.512081793	2.1121067867559	198.094977181624
869	Brazil	1994	200.59925667787	0.515760842	2.11448775833855	198.484768919531
870	Brazil	1995	200.53417665597	0.519685963	2.11371231255949	198.420464343411
871	Brazil	1996	199.54500754468	0.523863329	2.11195159950289	197.433055945177
872	Brazil	1997	197.02902087405	0.528177889	2.1126318708625	194.916389003188
873	Brazil	1998	193.66952978596	0.532481169	2.11341327275163	191.556116513208
874	Brazil	1999	190.05684598809	0.536972035	2.11078277197869	187.946063216111
875	Brazil	2000	186.66383332099	0.542051497	2.11598833539173	184.547844985598

TableS2 Frontier analysis of Smoking-Related Low Back Pain among WCBA

	location	year	val	SDI	frontier	eff_diff
876	Brazil	2001	182.43321879321	0.54730443	2.11735395455523	180.315864838655
877	Brazil	2002	177.17067148768	0.552776035	2.11291615711924	175.057755330561
878	Brazil	2003	171.33734845407	0.55814571	2.11154192242627	169.225806531644
879	Brazil	2004	165.36929516921	0.563772907	2.11779815385419	163.251497015356
880	Brazil	2005	159.83366011741	0.569458118	2.11226452972085	157.721395587689
881	Brazil	2006	154.59733437085	0.575338775	2.11528539394662	152.482048976903
882	Brazil	2007	149.09069568437	0.581588755	2.11444033782162	146.976255346548
883	Brazil	2008	143.52912804339	0.588077818	2.11639891769675	141.412729125693
884	Brazil	2009	138.05568998184	0.594073297	2.11366731583964	135.942022666
885	Brazil	2010	132.73155998667	0.600445846	2.11157473411483	130.619985252555
886	Brazil	2011	127.06636511337	0.606777291	2.11340051606059	124.952964597309
887	Brazil	2012	120.8228675991	0.612619337	2.11145202012597	118.711415578974
888	Brazil	2013	114.7567620457	0.618218237	2.11233509166954	112.64442695403
889	Brazil	2014	109.71863736392	0.623433814	2.11357709212987	107.60506027179
890	Brazil	2015	106.44783894822	0.628149803	2.11666876012716	104.331170188093
891	Brazil	2016	104.67599546975	0.632235114	2.11287429225444	102.563121177496
892	Brazil	2017	103.44207925755	0.636422188	2.1117743590607	101.330304898489
893	Brazil	2018	102.7242742178	0.640802974	2.11144338232244	100.612830835478
894	Brazil	2019	102.43437691596	0.645298005	2.11260156083333	100.321775355127
895	Brazil	2020	101.97913119743	0.649201568	2.11126419956211	99.8678669978679
896	Brazil	2021	102.43397831006	0.653043887	2.11577283072833	100.318205479332
897	Brunei	1990	72.0581672858	0.666081917	2.11256279755365	69.9456044882463
898	Brunei	1991	71.0269207695	0.671233831	2.11344211643706	68.9134786530629
899	Brunei	1992	69.82033676684	0.676702526	2.11457236008143	67.7057644067586
900	Brunei	1993	68.82790059744	0.68217544	2.11341842751747	66.7144821699225

TableS2 Frontier analysis of Smoking-Related Low Back Pain among WCBA

	location	year	val	SDI	frontier	eff_diff
901	Brunei	1994	67.76073451986	0.687394093	2.11107743170482	65.6496570881552
902	Brunei	1995	66.85735210737	0.692471156	2.11575353368475	64.7415985736853
903	Brunei	1996	65.78035734527	0.697602233	2.11139020558033	63.6689671396897
904	Brunei	1997	64.16317685096	0.702944386	2.11508693011658	62.0480899208434
905	Brunei	1998	62.66251984921	0.708630119	2.11234800850415	60.5501718407059
906	Brunei	1999	61.54570391399	0.714851237	2.11262235723364	59.4330815567564
907	Brunei	2000	60.92051762967	0.721552493	2.11393135138081	58.8065862782892
908	Brunei	2001	60.86805954631	0.728535069	2.11338126918971	58.7546782771203
909	Brunei	2002	60.52201838412	0.735343916	2.11170746534068	58.4103109187793
910	Brunei	2003	60.15784279421	0.741578091	2.11256370801853	58.0452790861915
911	Brunei	2004	59.91533038301	0.747311715	2.11244138004312	57.8028890029669
912	Brunei	2005	59.76820617405	0.752481793	2.10998114054152	57.6582250335085
913	Brunei	2006	59.47200878509	0.757318655	2.11029061747658	57.3617181676134
914	Brunei	2007	59.08219554736	0.761643129	2.11324040949581	56.9689551378642
915	Brunei	2008	58.52944610264	0.76571426	2.11256873282926	56.4168773698107
916	Brunei	2009	58.09481714421	0.769601682	2.11634275437796	55.978474389832
917	Brunei	2010	57.94765767606	0.773219221	2.11205079494545	55.8356068811145
918	Brunei	2011	57.67170323664	0.776631893	2.11281934103322	55.5588838956068
919	Brunei	2012	57.33549571001	0.780216552	2.11295968905259	55.2225360209574
920	Brunei	2013	56.99476557157	0.784017041	2.11291925636095	54.881846315209
921	Brunei	2014	57.04187394459	0.787733289	2.11344426708696	54.928429677503
922	Brunei	2015	57.15323473544	0.791367567	2.11445111340359	55.0387836220364
923	Brunei	2016	57.60998170931	0.794980772	2.11763782910116	55.4923438802088
924	Brunei	2017	58.38434802885	0.798309936	2.11330757715	56.2710404517
925	Brunei	2018	59.1158910888	0.801436553	2.11166039871782	57.0042306900822

TableS2 Frontier analysis of Smoking-Related Low Back Pain among WCBA

	location	year	val	SDI	frontier	eff_diff
926	Brunei	2019	59.543487736	0.804499388	2.11450864700512	57.4289790889949
927	Brunei	2020	57.56212228009	0.807424091	2.11360821705509	55.4485140630349
928	Brunei	2021	59.22347741857	0.810234367	2.11430711039229	57.1091703081777
929	Bulgaria	1990	450.17132815022	0.633446498	2.11467755939824	448.056650590822
930	Bulgaria	1991	449.38315567806	0.643069488	2.11183191777952	447.27132376028
931	Bulgaria	1992	447.15093149216	0.651186876	2.11335760305375	445.037573889106
932	Bulgaria	1993	445.1356677795	0.659161569	2.11347098711023	443.02219679239
933	Bulgaria	1994	443.39126283658	0.66743046	2.11606228637789	441.275200550202
934	Bulgaria	1995	441.76659170301	0.673999977	2.11240759095545	439.654184112055
935	Bulgaria	1996	438.61968810394	0.678862584	2.11053124711683	436.509156856823
936	Bulgaria	1997	435.90192657508	0.680221466	2.11261587328867	433.789310701791
937	Bulgaria	1998	433.89173812184	0.678496877	2.11085521383375	431.780882908006
938	Bulgaria	1999	431.5193464742	0.677446543	2.11010185209181	429.409244622108
939	Bulgaria	2000	428.7562139393	0.681004524	2.11220329694955	426.64401064235
940	Bulgaria	2001	427.03536101088	0.686487366	2.11665476938311	424.918706241497
941	Bulgaria	2002	424.73406983719	0.691511674	2.11418153798771	422.619888299202
942	Bulgaria	2003	424.15383625176	0.695565965	2.11261515555286	422.041221096207
943	Bulgaria	2004	424.00255394361	0.699881951	2.11372315152605	421.888830792084
944	Bulgaria	2005	424.78584532571	0.704471207	2.11260644503403	422.673238880676
945	Bulgaria	2006	424.98772870085	0.708868294	2.11238402172364	422.875344679126
946	Bulgaria	2007	425.67790561266	0.713192402	2.11068373851946	423.567221874141
947	Bulgaria	2008	427.09242164499	0.71767245	2.1118719914175	424.980549653573
948	Bulgaria	2009	428.84760944277	0.723676627	2.11143290093043	426.73617654184
949	Bulgaria	2010	431.45290317607	0.730701737	2.11343548594892	429.339467690121
950	Bulgaria	2011	433.92682133949	0.735749281	2.1120140661186	431.814807273371

TableS2 Frontier analysis of Smoking-Related Low Back Pain among WCBA

	location	year	val	SDI	frontier	eff_diff
951	Bulgaria	2012	437.26221387202	0.739177517	2.11362151509766	435.148592356922
952	Bulgaria	2013	441.91757742013	0.742135948	2.11205713440121	439.805520285729
953	Bulgaria	2014	445.72616397026	0.745372643	2.11746533903066	443.608698631229
954	Bulgaria	2015	451.00448558221	0.748979058	2.11268477168662	448.891800810523
955	Bulgaria	2016	455.07980834738	0.752259199	2.11490530579525	452.964903041585
956	Bulgaria	2017	459.28904242405	0.755665865	2.11139180633	457.17765061772
957	Bulgaria	2018	462.17842855459	0.75914846	2.11255046745164	460.065878087138
958	Bulgaria	2019	464.87247674212	0.762359791	2.11318363793068	462.759293104189
959	Bulgaria	2020	463.67102745476	0.765089711	2.11357028963667	461.557457165123
960	Bulgaria	2021	459.59425928615	0.768150939	2.11259791218055	457.481661373969
961	Burkina Faso	1990	8.76076896765	0.129695615	4.81715061597254	3.94361835167746
962	Burkina Faso	1991	8.94999494623	0.133299128	4.71112840883926	4.23886653739074
963	Burkina Faso	1992	9.09897729918	0.136594798	4.61864298099763	4.48033431818237
964	Burkina Faso	1993	9.23724413907	0.139825465	4.73604756607937	4.50119657299062
965	Burkina Faso	1994	9.37287381846	0.142848973	4.78257637200963	4.59029744645037
966	Burkina Faso	1995	9.47094913872	0.146115673	4.71578039097879	4.75516874774121
967	Burkina Faso	1996	9.56091881945	0.150041686	4.51310095807102	5.04781786137898
968	Burkina Faso	1997	9.68170177962	0.154228401	4.23678889935364	5.44491288026636
969	Burkina Faso	1998	9.76556579518	0.158707048	3.90836968900027	5.85719610617973
970	Burkina Faso	1999	9.85274989427	0.163468067	3.8275157634966	6.0252341307734
971	Burkina Faso	2000	9.8408337348	0.16808434	3.76221410351621	6.07861963128379
972	Burkina Faso	2001	9.768519197	0.172996395	3.74339835237459	6.02512084462541
973	Burkina Faso	2002	9.6484628325	0.17798199	3.6508170500286	5.9976457824714
974	Burkina Faso	2003	9.51508313282	0.183253539	3.63734248607014	5.87774064674986
975	Burkina Faso	2004	9.33885237401	0.188564995	3.57608476251373	5.76276761149627

TableS2 Frontier analysis of Smoking-Related Low Back Pain among WCBA

	location	year	val	SDI	frontier	eff_diff
976	Burkina Faso	2005	9.1781638812	0.194086128	3.52575331019233	5.65241057100767
977	Burkina Faso	2006	8.99592305037	0.199571952	3.43491781719912	5.56100523317088
978	Burkina Faso	2007	8.72290901542	0.204891092	3.5074570265665	5.2154519888535
979	Burkina Faso	2008	8.42543957419	0.210223295	3.46979134925847	4.95564822493153
980	Burkina Faso	2009	8.13690092528	0.215423374	3.46303444168492	4.67386648359508
981	Burkina Faso	2010	7.89667324669	0.22100103	2.96608057452147	4.93059267216853
982	Burkina Faso	2011	7.68468858837	0.226696632	2.74610826234868	4.93858032602132
983	Burkina Faso	2012	7.47023399861	0.232589135	2.58582597474029	4.88440802386971
984	Burkina Faso	2013	7.25588775806	0.238497491	2.59764444172892	4.65824331633108
985	Burkina Faso	2014	7.08600990667	0.244378901	2.55789024242918	4.52811966424082
986	Burkina Faso	2015	6.9383821727	0.250208261	2.55905037533425	4.37933179736575
987	Burkina Faso	2016	6.83892834823	0.256081774	2.55420447409179	4.2847238741382
988	Burkina Faso	2017	6.77976934097	0.262033009	2.48165208879719	4.29811725217281
989	Burkina Faso	2018	6.75511163918	0.268102073	2.47089943349243	4.28421220568757
990	Burkina Faso	2019	6.69119217686	0.274287092	2.43619844400065	4.25499373285935
991	Burkina Faso	2020	6.58454163355	0.279871317	2.45784717726153	4.12669445628847
992	Burkina Faso	2021	6.43952816876	0.285118402	2.42830784262289	4.0112203261371
993	Burundi	1990	23.20092832896	0.20586736	3.50575652649418	19.6951718024658
994	Burundi	1991	23.06756958153	0.20849572	3.48642370652492	19.5811458750051
995	Burundi	1992	22.80030195983	0.210864619	3.48517865487852	19.3151233049515
996	Burundi	1993	22.85976036533	0.213249386	3.49747299893879	19.3622873663912
997	Burundi	1994	22.93798531905	0.215222973	3.46412196564806	19.4738633534019
998	Burundi	1995	22.44282050603	0.215631676	3.50300190968393	18.9398185963461
999	Burundi	1996	21.96633702494	0.215526325	3.43933660484127	18.5270004200987
1000	Burundi	1997	21.91564588176	0.21576182	3.50263130551453	18.4130145762455

TableS2 Frontier analysis of Smoking-Related Low Back Pain among WCBA

	location	year	val	SDI	frontier	eff_diff
1001	Burundi	1998	21.76464507308	0.216402603	2.82155874150085	18.9430863315792
1002	Burundi	1999	21.48802760464	0.216855767	2.87672632725173	18.6113012773883
1003	Burundi	2000	20.97603835752	0.216869858	2.89505132684708	18.0809870306729
1004	Burundi	2001	20.82334668271	0.216988726	2.94658407145069	17.8767626112593
1005	Burundi	2002	20.73204850826	0.217782477	2.87017833124382	17.8618701770162
1006	Burundi	2003	20.69217932343	0.218961537	2.89139158348254	17.8007877399475
1007	Burundi	2004	20.51113615939	0.220918709	2.96770168212486	17.5434344772651
1008	Burundi	2005	20.2592727236	0.223084372	2.67847476538633	17.5807979582137
1009	Burundi	2006	20.01031040525	0.22582189	2.73077396057171	17.2795364446783
1010	Burundi	2007	19.76168988575	0.228996819	2.6428256648568	17.1188642208932
1011	Burundi	2008	19.52179183034	0.232853551	2.60858341271978	16.9132084176202
1012	Burundi	2009	19.21940281565	0.23694304	2.66501774568854	16.5543850699615
1013	Burundi	2010	18.8917910259	0.241447786	2.55004931083935	16.3417417150606
1014	Burundi	2011	18.43617416093	0.246277277	2.5598567997002	15.8763173612298
1015	Burundi	2012	17.58870787147	0.251572303	2.52104650966649	15.0676613618035
1016	Burundi	2013	16.47913256321	0.257191355	2.51734335803015	13.9617892051798
1017	Burundi	2014	15.38074350505	0.262794722	2.5203548684339	12.8603886366161
1018	Burundi	2015	14.60762661303	0.267327938	2.48193655330519	12.1256900597248
1019	Burundi	2016	14.20255989428	0.271587852	2.46986427948156	11.7326956147984
1020	Burundi	2017	13.77328449569	0.275840505	2.4477044259181	11.3255800697719
1021	Burundi	2018	13.29990405962	0.27973329	2.44685163733859	10.8530524222814
1022	Burundi	2019	12.84778276612	0.283442515	2.45668121568726	10.3911015504327
1023	Burundi	2020	12.44916477307	0.286496	2.44484221604407	10.0043225570259
1024	Burundi	2021	12.08600276727	0.289374365	2.4437884001056	9.6422143671644
1025	Cabo Verde	1990	18.14591701787	0.276723367	2.45616243982457	15.6897545780454

TableS2 Frontier analysis of Smoking-Related Low Back Pain among WCBA

	location	year	val	SDI	frontier	eff_diff
1026	Cabo Verde	1991	18.38655573834	0.282931722	2.45892482866315	15.9276309096769
1027	Cabo Verde	1992	18.60973049086	0.288460371	2.42068364791644	16.1890468429436
1028	Cabo Verde	1993	18.84800684691	0.294517286	2.40529248964683	16.4427143572632
1029	Cabo Verde	1994	19.04050555652	0.302216794	2.39530554837865	16.6452000081414
1030	Cabo Verde	1995	19.15095806298	0.310675634	2.36412611994015	16.7868319430399
1031	Cabo Verde	1996	19.24765859784	0.319736229	2.34222147443926	16.9054371234007
1032	Cabo Verde	1997	19.27447065352	0.329049585	2.30929276623075	16.9651778872893
1033	Cabo Verde	1998	19.24551918375	0.338786069	2.26496298613258	16.9805561976174
1034	Cabo Verde	1999	19.14794056866	0.349296918	2.12082460586681	17.0271159627932
1035	Cabo Verde	2000	19.03338700098	0.359899017	2.11573646630636	16.9176505346736
1036	Cabo Verde	2001	18.58249948725	0.370070276	2.11699601798265	16.4655034692674
1037	Cabo Verde	2002	18.1413275167	0.379846674	2.11626378261127	16.0250637340887
1038	Cabo Verde	2003	17.65884894607	0.389419786	2.11679487805365	15.5420540680164
1039	Cabo Verde	2004	17.21011131228	0.398838732	2.11211568411868	15.0979956281613
1040	Cabo Verde	2005	16.74576311155	0.408009221	2.11313196926403	14.632631142286
1041	Cabo Verde	2006	16.30759986216	0.417433786	2.11498522586717	14.1926146362928
1042	Cabo Verde	2007	15.82675816113	0.427379787	2.11525928009475	13.7114988810353
1043	Cabo Verde	2008	15.36861549857	0.43737213	2.11130485336224	13.2573106452078
1044	Cabo Verde	2009	14.98427114606	0.446691506	2.11251678970203	12.871754356358
1045	Cabo Verde	2010	14.68335935475	0.455647568	2.113343238421	12.570016116329
1046	Cabo Verde	2011	14.46290578837	0.464473969	2.11338964627361	12.3495161420964
1047	Cabo Verde	2012	14.2719636706	0.472857771	2.11628982202024	12.1556738485798
1048	Cabo Verde	2013	14.1108842771	0.480796481	2.11542210818029	11.9954621689197
1049	Cabo Verde	2014	14.05462921263	0.488259896	2.11197240808812	11.9426568045419
1050	Cabo Verde	2015	14.06641605391	0.495229489	2.1140582553665	11.9523577985435

TableS2 Frontier analysis of Smoking-Related Low Back Pain among WCBA

	location	year	val	SDI	frontier	eff_diff
1051	Cabo Verde	2016	14.1569302786	0.501855424	2.11536953890823	12.0415607396918
1052	Cabo Verde	2017	14.31680042598	0.508435792	2.1137646600025	12.2030357659775
1053	Cabo Verde	2018	14.48842710122	0.515285886	2.11459457386642	12.3738325273536
1054	Cabo Verde	2019	14.68105852147	0.522488008	2.11278621014397	12.568272311326
1055	Cabo Verde	2020	14.83394837217	0.528461595	2.12058997417735	12.7133583979926
1056	Cabo Verde	2021	15.00162868309	0.533534539	2.11134293181627	12.8902857512737
1057	Cambodia	1990	19.66329355726	0.289075059	2.43968381364485	17.2236097436151
1058	Cambodia	1991	20.31338641592	0.2924792	2.43554977944589	17.8778366364741
1059	Cambodia	1992	20.98165292055	0.296900955	2.40156670995721	18.5800862105928
1060	Cambodia	1993	21.79405202842	0.301627202	2.39594878341456	19.3981032450054
1061	Cambodia	1994	22.56337533111	0.305722592	2.38646301379759	20.1769123173124
1062	Cambodia	1995	23.39641853725	0.310678881	2.362915927434	21.033502609816
1063	Cambodia	1996	24.25020054209	0.31563097	2.36288007301756	21.8873204690724
1064	Cambodia	1997	25.07169785756	0.32050375	2.34379085188628	22.7279070056737
1065	Cambodia	1998	25.73290696428	0.324974299	2.32480672485076	23.4081002394292
1066	Cambodia	1999	26.22916282663	0.330224246	2.3064985179534	23.9226643086766
1067	Cambodia	2000	26.43158617474	0.33607503	2.28418964062052	24.1473965341195
1068	Cambodia	2001	26.49114274912	0.342507911	2.24491084091792	24.2462319082021
1069	Cambodia	2002	26.4621797706	0.349465712	2.12305999869535	24.3391197719046
1070	Cambodia	2003	26.4125704478	0.35692966	2.11645290061772	24.2961175471823
1071	Cambodia	2004	26.27825242976	0.364854645	2.11295886094	24.16529356882
1072	Cambodia	2005	26.13592788554	0.373331815	2.11293820256881	24.0229896829712
1073	Cambodia	2006	25.95255050952	0.381698166	2.11498815441561	23.8375623551044
1074	Cambodia	2007	25.67226148311	0.38987623	2.11086173530881	23.5613997478012
1075	Cambodia	2008	25.39600778798	0.39767952	2.11340324948574	23.2826045384943

TableS2 Frontier analysis of Smoking-Related Low Back Pain among WCBA

	location	year	val	SDI	frontier	eff_diff
1076	Cambodia	2009	25.17796707411	0.404220933	2.11078544064571	23.0671816334643
1077	Cambodia	2010	24.9687570145	0.410211121	2.11366168983679	22.8550953246632
1078	Cambodia	2011	24.7272924027	0.415964409	2.11163385732792	22.6156585453721
1079	Cambodia	2012	24.47514480173	0.421769754	2.11279681786929	22.3623479838607
1080	Cambodia	2013	24.23188878946	0.42771506	2.11200599172785	22.1198827977322
1081	Cambodia	2014	24.03558723387	0.433597749	2.11192529785349	21.9236619360165
1082	Cambodia	2015	23.91578399207	0.439487358	2.11529161348653	21.8004923785835
1083	Cambodia	2016	23.93900772703	0.445358172	2.11361993125455	21.8253877957755
1084	Cambodia	2017	24.10527765964	0.451221744	2.1114686312019	21.9938090284381
1085	Cambodia	2018	24.26762594811	0.45734149	2.11294591255448	22.1546800355555
1086	Cambodia	2019	24.43359885248	0.46364343	2.11453086169433	22.3190679907857
1087	Cambodia	2020	24.60045103103	0.468876054	2.11538159082787	22.4850694402021
1088	Cambodia	2021	24.83647435358	0.473621491	2.11184980982887	22.7246245437511
1089	Cameroon	1990	7.7305594334	0.303055334	2.39908579829676	5.33147363510324
1090	Cameroon	1991	7.68052286186	0.309623458	2.3863689012075	5.2941539606525
1091	Cameroon	1992	7.64074018568	0.315601269	2.36525583695651	5.27548434872349
1092	Cameroon	1993	7.60210745236	0.320831654	2.341598081401	5.260509370959
1093	Cameroon	1994	7.58464836283	0.325410123	2.32043144946895	5.26421691336105
1094	Cameroon	1995	7.55767396187	0.329539318	2.30604435676173	5.25162960510827
1095	Cameroon	1996	7.53917840189	0.333425982	2.28625784245621	5.25292055943379
1096	Cameroon	1997	7.52863878239	0.337346001	2.27174406445422	5.25689471793578
1097	Cameroon	1998	7.50241941841	0.34119709	2.23731532226158	5.26510409614842
1098	Cameroon	1999	7.49217634661	0.344955364	2.17790973877865	5.31426660783135
1099	Cameroon	2000	7.44997895811	0.348814789	2.11648041328286	5.33349854482714
1100	Cameroon	2001	7.41323185789	0.352631873	2.12200450274413	5.29122735514587

TableS2 Frontier analysis of Smoking-Related Low Back Pain among WCBA

	location	year	val	SDI	frontier	eff_diff
1101	Cameroon	2002	7.35498686949	0.356511817	2.11922070687086	5.23576616261914
1102	Cameroon	2003	7.30337695128	0.360668581	2.11150061308286	5.19187633819714
1103	Cameroon	2004	7.24840091374	0.36510003	2.11363542651292	5.13476548722708
1104	Cameroon	2005	7.18430703849	0.369545756	2.11416803994607	5.07013899854393
1105	Cameroon	2006	7.07888673918	0.374355878	2.1134557326704	4.9654310065096
1106	Cameroon	2007	6.94564170263	0.379490349	2.11337829471358	4.83226340791642
1107	Cameroon	2008	6.77793286066	0.384838304	2.11469817673108	4.66323468392892
1108	Cameroon	2009	6.60215499031	0.390288643	2.11277438635235	4.48938060395765
1109	Cameroon	2010	6.45360027316	0.396067145	2.11266557952944	4.34093469363056
1110	Cameroon	2011	6.30921387139	0.402092799	2.1140378089787	4.1951760624113
1111	Cameroon	2012	6.14809778437	0.408580123	2.11894363640352	4.02915414796648
1112	Cameroon	2013	5.99280163027	0.415607965	2.11265935758514	3.88014227268486
1113	Cameroon	2014	5.85404356583	0.42303356	2.11235549065612	3.74168807517388
1114	Cameroon	2015	5.73966267592	0.430830276	2.11124395328173	3.62841872263827
1115	Cameroon	2016	5.66937552452	0.438878945	2.11175481214699	3.55762071237301
1116	Cameroon	2017	5.61033407972	0.4471851	2.11477251096807	3.49556156875193
1117	Cameroon	2018	5.55438473209	0.455687847	2.11367027705369	3.44071445503631
1118	Cameroon	2019	5.50698359414	0.464246473	2.11585248778989	3.39113110635011
1119	Cameroon	2020	5.47963085737	0.472285846	2.11743159319154	3.36219926417846
1120	Cameroon	2021	5.38879850563	0.479691223	2.11422362606679	3.2745748795632
1121	Canada	1990	320.77392748244	0.781977864	2.11036266155934	318.663564820881
1122	Canada	1991	325.68879657641	0.78415936	2.11148326435307	323.577313312057
1123	Canada	1992	329.59914684344	0.787030537	2.11512078517302	327.484026058267
1124	Canada	1993	332.72924964982	0.789716593	2.11375604035618	330.615493609464
1125	Canada	1994	334.4427368142	0.792598518	2.11292707415317	332.329809740047

TableS2 Frontier analysis of Smoking-Related Low Back Pain among WCBA

	location	year	val	SDI	frontier	eff_diff
1126	Canada	1995	334.78766276556	0.796521166	2.11337232086959	332.67429044469
1127	Canada	1996	331.70464844106	0.80106497	2.11228627027014	329.59236217079
1128	Canada	1997	325.38217747266	0.804833356	2.11341739155986	323.2687600811
1129	Canada	1998	317.48504368667	0.808135441	2.11648103067714	315.368562655993
1130	Canada	1999	310.0429524821	0.81226594	2.11103923308724	307.931913249013
1131	Canada	2000	303.59627138041	0.816685059	2.11297956748803	301.483291812922
1132	Canada	2001	296.77742378538	0.82069625	2.11301318162557	294.664410603754
1133	Canada	2002	289.55899133458	0.824402272	2.113116765645	287.445874568935
1134	Canada	2003	281.50470190336	0.827801096	2.1117225038677	279.392979399492
1135	Canada	2004	274.00634539953	0.83092811	2.11198962254341	271.894355776987
1136	Canada	2005	267.06814632113	0.833288294	2.11233108342906	264.955815237701
1137	Canada	2006	261.20336261863	0.83491566	2.11234662199841	259.091015996632
1138	Canada	2007	254.80322335316	0.836625096	2.11280041800382	252.690422935156
1139	Canada	2008	249.18025005867	0.838913685	2.11245663886313	247.067793419807
1140	Canada	2009	243.9892627114	0.841583671	2.11440368301182	241.874859028388
1141	Canada	2010	239.47645936223	0.844853489	2.11081622446047	237.36564313777
1142	Canada	2011	235.08256782051	0.848017833	2.11068421424816	232.971883606262
1143	Canada	2012	231.03286350162	0.850978048	2.11210503432069	228.920758467299
1144	Canada	2013	227.58480184341	0.853844945	2.11416222760068	225.470639615809
1145	Canada	2014	224.99103010504	0.856759934	2.11245023661465	222.878579868425
1146	Canada	2015	223.12187186688	0.859532904	2.11352544959635	221.008346417284
1147	Canada	2016	223.64160065158	0.862174226	2.11178118105491	221.529819470525
1148	Canada	2017	225.30613440384	0.86505383	2.11394902463418	223.192185379206
1149	Canada	2018	227.17034032416	0.867905541	2.11330575201797	225.057034572142
1150	Canada	2019	228.11010334967	0.870554479	2.118977773707	225.991125575963

TableS2 Frontier analysis of Smoking-Related Low Back Pain among WCBA

	location	year	val	SDI	frontier	eff_diff
1151	Canada	2020	220.76375195691	0.871992631	2.11217313636083	218.651578820549
1152	Canada	2021	211.47323055982	0.87317068	2.11454446950206	209.358686090318
1153	Caribbean	1990	68.45097761847	0.518111381	2.11259981286118	66.3383778056088
1154	Caribbean	1991	68.42076029685	0.522788586	2.11490268935797	66.305857607492
1155	Caribbean	1992	68.63054754107	0.526984192	2.11477495406924	66.5157725870008
1156	Caribbean	1993	69.10330696071	0.530506786	2.11132704905034	66.9919799116597
1157	Caribbean	1994	69.93794470126	0.533584311	2.11272617788929	67.8252185233707
1158	Caribbean	1995	70.99548821516	0.536650593	2.11504143733064	68.8804467778293
1159	Caribbean	1996	71.96041325973	0.539775932	2.11283123140294	69.8475820283271
1160	Caribbean	1997	72.90646140098	0.543302886	2.11282273120845	70.7936386697716
1161	Caribbean	1998	73.57357008199	0.547365066	2.11462784268338	71.4589422393066
1162	Caribbean	1999	74.19433332889	0.552060172	2.11382140426045	72.0805119246295
1163	Caribbean	2000	74.6569878535	0.557368943	2.1120881845167	72.5448996689833
1164	Caribbean	2001	74.85166531185	0.563110488	2.11648696645027	72.7351783453997
1165	Caribbean	2002	74.6581288964	0.569068592	2.11245471248448	72.5456741839155
1166	Caribbean	2003	74.06566569716	0.574888622	2.113504143533	71.952161553627
1167	Caribbean	2004	73.1429583414	0.580533315	2.11239835323861	71.0305599881614
1168	Caribbean	2005	72.13768527079	0.58575799	2.11703357212533	70.0206516986647
1169	Caribbean	2006	70.74702890954	0.590602336	2.1119878246475	68.6350410848925
1170	Caribbean	2007	69.04436178905	0.594709606	2.11439377751667	66.9299680115333
1171	Caribbean	2008	67.17761573061	0.59829691	2.11463607706423	65.0629796535458
1172	Caribbean	2009	65.19870797323	0.601654828	2.11395480993529	63.0847531632947
1173	Caribbean	2010	63.49038566495	0.605480076	2.1132010323882	61.3771846325618
1174	Caribbean	2011	61.73254228736	0.609525046	2.11377933047119	59.6187629568888
1175	Caribbean	2012	59.7408054511	0.613254721	2.11624766028614	57.6245577908139

TableS2 Frontier analysis of Smoking-Related Low Back Pain among WCBA

	location	year	val	SDI	frontier	eff_diff
1176	Caribbean	2013	57.8087785287	0.61687685	2.11323896037167	55.6955395683283
1177	Caribbean	2014	56.04217911756	0.620442645	2.11561649084012	53.9265626267199
1178	Caribbean	2015	54.68394618372	0.624074667	2.11259145685402	52.571354726866
1179	Caribbean	2016	53.73180544082	0.627582074	2.11267617667319	51.6191292641468
1180	Caribbean	2017	53.04741624664	0.630788066	2.11262450013	50.93479174651
1181	Caribbean	2018	52.51159229447	0.633972166	2.11246142729111	50.3991308671789
1182	Caribbean	2019	51.95527791537	0.63732123	2.11388703701293	49.8413908783571
1183	Caribbean	2020	51.50791558614	0.639708532	2.11342267067931	49.3944929154607
1184	Caribbean	2021	50.8738622025	0.642003055	2.11260786941386	48.7612543330861
1185	Central African Republic	1990	11.2790630353	0.216825191	2.8375609198468	8.4415021154532
1186	Central African Republic	1991	11.13633405505	0.220191656	2.963143100211	8.173190954839
1187	Central African Republic	1992	10.98447311288	0.223007541	2.75731402364978	8.22715908923022
1188	Central African Republic	1993	10.79794794018	0.225451125	2.70482714414815	8.09312079603185
1189	Central African Republic	1994	10.61238721532	0.228487724	2.67337191654333	7.93901529877667
1190	Central African Republic	1995	10.44428099614	0.231696792	2.59330922496726	7.85097177117274
1191	Central African Republic	1996	10.26870174173	0.233989591	2.63096261923721	7.63773912249279
1192	Central African Republic	1997	10.06762494451	0.236671999	2.59247325718767	7.47515168732233
1193	Central African Republic	1998	9.83720153413	0.239743529	2.55514520764255	7.28205632648745
1194	Central African Republic	1999	9.58084531587	0.24298853	2.55080704675413	7.03003826911587
1195	Central African Republic	2000	9.34779962452	0.246021132	2.58277804929868	6.76502157522132
1196	Central African Republic	2001	9.15431477226	0.24924674	2.55360767656321	6.60070709569679
1197	Central African Republic	2002	8.95802718799	0.252524369	2.50868104031104	6.44934614767896
1198	Central African Republic	2003	8.76918741168	0.255101519	2.52122383803171	6.24796357364829
1199	Central African Republic	2004	8.60818336121	0.257778778	2.53451806385282	6.07366529735718
1200	Central African Republic	2005	8.46733348426	0.260451918	2.51717887615672	5.95015460810328

TableS2 Frontier analysis of Smoking-Related Low Back Pain among WCBA

	location	year	val	SDI	frontier	eff_diff
1201	Central African Republic	2006	8.36067104577	0.263712446	2.5098820970786	5.8507889486914
1202	Central African Republic	2007	8.27351606912	0.267332195	2.47850368038043	5.79501238873957
1203	Central African Republic	2008	8.18815590091	0.271158118	2.47931060176478	5.70884529914522
1204	Central African Republic	2009	8.12468056254	0.275338515	2.44857104027545	5.67610952226455
1205	Central African Republic	2010	8.10370910823	0.279981382	2.44833718340216	5.65537192482784
1206	Central African Republic	2011	8.11043800538	0.285097245	2.41707171292774	5.69336629245226
1207	Central African Republic	2012	8.1223961628	0.290460023	2.43577581763406	5.68662034516594
1208	Central African Republic	2013	8.15960120732	0.29010493	2.43953243606385	5.72006877125615
1209	Central African Republic	2014	8.28156830522	0.289999498	2.44170456965803	5.83986373556197
1210	Central African Republic	2015	8.44749711797	0.290827871	2.43470168014662	6.01279543782338
1211	Central African Republic	2016	8.56477379401	0.2924304	2.42775664230897	6.13701715170103
1212	Central African Republic	2017	8.65509762981	0.294752243	2.4040131989808	6.2510844308292
1213	Central African Republic	2018	8.75646891786	0.298011223	2.40033982286205	6.35612909499795
1214	Central African Republic	2019	8.82130697541	0.301953064	2.40602691513147	6.41528006027853
1215	Central African Republic	2020	8.79524574069	0.305426298	2.38230925883368	6.41293648185632
1216	Central African Republic	2021	8.81061724653	0.30916769	2.38426731512647	6.42634993140353
1217	Central Asia	1990	31.26738463308	0.553361946	2.11161424322018	29.1557703898598
1218	Central Asia	1991	30.88854725763	0.555147774	2.11376441661337	28.7747828410166
1219	Central Asia	1992	30.801714918	0.557269541	2.11390467801797	28.687810239982
1220	Central Asia	1993	31.01241348345	0.560082892	2.11114157711294	28.9012719063371
1221	Central Asia	1994	31.49148205824	0.562699531	2.11195008521867	29.3795319730213
1222	Central Asia	1995	32.03832867564	0.565585602	2.11278509117015	29.9255435844699
1223	Central Asia	1996	32.75485842476	0.568760584	2.11465430529068	30.6402041194693
1224	Central Asia	1997	33.52060359243	0.571766483	2.112407047825	31.408196544605
1225	Central Asia	1998	34.33058857749	0.574659386	2.11140757029188	32.2191810071981

TableS2 Frontier analysis of Smoking-Related Low Back Pain among WCBA

	location	year	val	SDI	frontier	eff_diff
1226	Central Asia	1999	35.21530076073	0.578120701	2.11217040146056	33.1031303592694
1227	Central Asia	2000	35.95613130989	0.582155536	2.11262707523941	33.8435042346506
1228	Central Asia	2001	36.66559558233	0.586645798	2.11253169990239	34.5530638824276
1229	Central Asia	2002	37.57335109971	0.591457113	2.11256252719044	35.4607885725196
1230	Central Asia	2003	38.50289701062	0.596529769	2.11144301768423	36.3914539929358
1231	Central Asia	2004	39.32423452467	0.601736719	2.11062725162413	37.2136072730459
1232	Central Asia	2005	39.85768490858	0.60729382	2.10990342395226	37.7477814846277
1233	Central Asia	2006	40.25406675927	0.613078276	2.11284061566985	38.1412261436001
1234	Central Asia	2007	40.67295440866	0.619104578	2.11438966918091	38.5585647394791
1235	Central Asia	2008	41.01048561368	0.624930378	2.11179620979907	38.8986894038809
1236	Central Asia	2009	41.23559533658	0.630102885	2.11491375563786	39.1206815809421
1237	Central Asia	2010	41.20080069229	0.635295597	2.11476644449	39.0860342478
1238	Central Asia	2011	40.92021773319	0.640313561	2.11458122199086	38.8056365111991
1239	Central Asia	2012	40.33604240358	0.644673516	2.11145732722237	38.2245850763576
1240	Central Asia	2013	39.70107620335	0.648888141	2.11464136799524	37.5864348353548
1241	Central Asia	2014	39.17541695358	0.65299988	2.11885786745387	37.0565590861261
1242	Central Asia	2015	38.96567121109	0.656817827	2.11164339934899	36.854027811741
1243	Central Asia	2016	39.13238650911	0.660312421	2.10965974369197	37.022726765418
1244	Central Asia	2017	39.46913094691	0.663576782	2.1181649981824	37.3509659487276
1245	Central Asia	2018	39.9199735152	0.666666449	2.11359048751	37.80638302769
1246	Central Asia	2019	40.30471215877	0.669837304	2.11009195680304	38.194620201967
1247	Central Asia	2020	40.46820838442	0.67248671	2.11300722820864	38.3552011562114
1248	Central Asia	2021	40.65886516653	0.675163978	2.11308073558447	38.5457844309455
1249	Central Europe	1990	379.11878675894	0.637272174	2.111236045857	377.007550713083
1250	Central Europe	1991	378.86525892747	0.643082622	2.11420899606591	376.751049931404

TableS2 Frontier analysis of Smoking-Related Low Back Pain among WCBA

	location	year	val	SDI	frontier	eff_diff
1251	Central Europe	1992	378.52564017264	0.648772145	2.1125436873975	376.413096485243
1252	Central Europe	1993	378.0949403342	0.654469912	2.11076932033717	375.984171013863
1253	Central Europe	1994	377.81783572457	0.661219388	2.11165139888724	375.706184325683
1254	Central Europe	1995	377.63635157115	0.667954947	2.118755632295	375.517595938855
1255	Central Europe	1996	376.20704199526	0.674121167	2.11092968981387	374.096112305446
1256	Central Europe	1997	373.49405317583	0.680037255	2.11442258984365	371.379630585986
1257	Central Europe	1998	371.02388715356	0.685968374	2.11609833238776	368.907788821172
1258	Central Europe	1999	368.14746971512	0.692056184	2.1122564418023	366.035213273318
1259	Central Europe	2000	365.1254374234	0.698779713	2.11468392591587	363.010753497484
1260	Central Europe	2001	362.15106961992	0.705656783	2.11197258652016	360.0390970334
1261	Central Europe	2002	358.93342802801	0.712000597	2.11308414751597	356.820343880494
1262	Central Europe	2003	355.9566674091	0.717823644	2.11509762227973	353.84156978682
1263	Central Europe	2004	353.33888538419	0.72349425	2.11294804328292	351.225937340907
1264	Central Europe	2005	351.04786296114	0.728674093	2.11595276956059	348.931910191579
1265	Central Europe	2006	349.59253259851	0.733476441	2.11150061308286	347.481031985427
1266	Central Europe	2007	348.46552636275	0.738076675	2.11398357256625	346.351542790184
1267	Central Europe	2008	347.76724821346	0.743147843	2.11648857540931	345.650759638051
1268	Central Europe	2009	347.91980934234	0.748765718	2.11108166354247	345.808727678798
1269	Central Europe	2010	348.8454680691	0.754919495	2.11321849810377	346.732249570996
1270	Central Europe	2011	350.04282025	0.760349288	2.11064254562869	347.932177704371
1271	Central Europe	2012	351.31892633026	0.764803271	2.11404958267298	349.204876747587
1272	Central Europe	2013	353.11132021804	0.768567712	2.11437858913631	350.996941628904
1273	Central Europe	2014	355.23640764262	0.771755473	2.11730929584182	353.119098346778
1274	Central Europe	2015	358.10039065997	0.774844012	2.11445865621422	355.985932003756
1275	Central Europe	2016	361.08416969445	0.777817247	2.11204692195425	358.972122772496

TableS2 Frontier analysis of Smoking-Related Low Back Pain among WCBA

	location	year	val	SDI	frontier	eff_diff
1276	Central Europe	2017	363.41963133784	0.78135932	2.11179662297036	361.30783471487
1277	Central Europe	2018	364.83024074717	0.785476334	2.11253964780725	362.717701099363
1278	Central Europe	2019	366.52369913431	0.789748976	2.11499601925875	364.408703115051
1279	Central Europe	2020	367.23826962109	0.793111123	2.11290473388671	365.125364887203
1280	Central Europe	2021	366.5955374086	0.796244448	2.11376222366548	364.481775184934
1281	Central Latin America	1990	72.26749889786	0.48578741	2.11489696605	70.15260193181
1282	Central Latin America	1991	71.4673587011	0.489558534	2.1137716152324	69.3535870858676
1283	Central Latin America	1992	70.70129203336	0.494110188	2.11746453800708	68.5838274953529
1284	Central Latin America	1993	69.99949223321	0.499417966	2.11304372695522	67.8864485062548
1285	Central Latin America	1994	69.47505289535	0.505060641	2.10990292699443	67.3651499683556
1286	Central Latin America	1995	69.04457667586	0.509874246	2.11523441119877	66.9293422646612
1287	Central Latin America	1996	68.55695074007	0.514693467	2.11650022524787	66.4404505148221
1288	Central Latin America	1997	67.96794286328	0.520170671	2.11268804132173	65.8552548219583
1289	Central Latin America	1998	67.27779488877	0.526131813	2.11526318017213	65.1625317085979
1290	Central Latin America	1999	66.58451058818	0.531807023	2.11357142054633	64.4709391676337
1291	Central Latin America	2000	65.8756155139	0.537300373	2.111495012064	63.764120501836
1292	Central Latin America	2001	64.92834971345	0.542439835	2.11227158615438	62.8160781272956
1293	Central Latin America	2002	63.62576722277	0.547134354	2.11527223599871	61.5104949867713
1294	Central Latin America	2003	62.16552685284	0.551446098	2.11514805794276	60.0503787948972
1295	Central Latin America	2004	60.61975750014	0.556011499	2.11291283799968	58.5068446621403
1296	Central Latin America	2005	59.16676861481	0.560749531	2.11466249450919	57.0521061203008
1297	Central Latin America	2006	57.54988952229	0.565648691	2.11350294841636	55.4363865738736
1298	Central Latin America	2007	55.72057483996	0.570707268	2.11229534650857	53.6082794934514
1299	Central Latin America	2008	53.91120153813	0.575839326	2.11747847358266	51.7937230645473
1300	Central Latin America	2009	52.36704085988	0.579940059	2.11229827543547	50.2547425844445

TableS2 Frontier analysis of Smoking-Related Low Back Pain among WCBA

	location	year	val	SDI	frontier	eff_diff
1301	Central Latin America	2010	51.29624877728	0.58425317	2.11736923931015	49.1788795379698
1302	Central Latin America	2011	50.72412413376	0.589307552	2.11257805497595	48.6115460787841
1303	Central Latin America	2012	50.24617956172	0.594827165	2.11541921204587	48.1307603496741
1304	Central Latin America	2013	49.84550110033	0.600731022	2.11420381581568	47.7312972845143
1305	Central Latin America	2014	49.57108091174	0.60650929	2.11520072315397	47.455880188586
1306	Central Latin America	2015	49.42318628939	0.611815329	2.11302340192472	47.3101628874653
1307	Central Latin America	2016	49.49534232536	0.617086095	2.11267601650781	47.3826663088522
1308	Central Latin America	2017	49.8551153447	0.622442429	2.11198905556724	47.7431262891328
1309	Central Latin America	2018	50.26279474898	0.627764642	2.11373727650877	48.1490574724712
1310	Central Latin America	2019	50.53946720515	0.632847071	2.11568500289382	48.4237822022562
1311	Central Latin America	2020	50.22014819768	0.636995887	2.11159744313392	48.1085507545461
1312	Central Latin America	2021	50.29053761521	0.6406851	2.11405434952384	48.1764832656862
1313	Central sub-Saharan Africa	1990	10.8138912334	0.302374953	2.4033164520881	8.4105747813119
1314	Central sub-Saharan Africa	1991	10.75518050983	0.305220591	2.38237512662436	8.37280538320564
1315	Central sub-Saharan Africa	1992	10.69310788445	0.307803569	2.38209973012701	8.31100815432299
1316	Central sub-Saharan Africa	1993	10.62557214258	0.309082037	2.37120268294697	8.25436945963303
1317	Central sub-Saharan Africa	1994	10.54427825532	0.309897978	2.38410817178286	8.16017008353714
1318	Central sub-Saharan Africa	1995	10.47178511594	0.311059245	2.35901908262737	8.11276603331263
1319	Central sub-Saharan Africa	1996	10.40350328098	0.312674396	2.36703795471074	8.03646532626927
1320	Central sub-Saharan Africa	1997	10.30986388269	0.314501685	2.36272795969203	7.94713592299797
1321	Central sub-Saharan Africa	1998	10.21632418083	0.316506276	2.36178631440398	7.85453786642602
1322	Central sub-Saharan Africa	1999	10.12214599366	0.318568997	2.34135369735852	7.78079229630148
1323	Central sub-Saharan Africa	2000	10.05701129095	0.320841477	2.34222778779	7.71478350316
1324	Central sub-Saharan Africa	2001	9.95604186737	0.323749194	2.32516190137064	7.63087996599936
1325	Central sub-Saharan Africa	2002	9.85461416236	0.327566904	2.31829312372644	7.53632103863356

TableS2 Frontier analysis of Smoking-Related Low Back Pain among WCBA

	location	year	val	SDI	frontier	eff_diff
1326	Central sub-Saharan Africa	2003	9.76652368065	0.331934652	2.29824346766946	7.46828021298054
1327	Central sub-Saharan Africa	2004	9.6877970514	0.337420017	2.27479230504597	7.41300474635403
1328	Central sub-Saharan Africa	2005	9.6540249747	0.344099267	2.17485904863057	7.47916592606943
1329	Central sub-Saharan Africa	2006	9.66717173737	0.351459736	2.12583711522655	7.54133462214345
1330	Central sub-Saharan Africa	2007	9.68944860849	0.359565506	2.11784350198854	7.57160510650146
1331	Central sub-Saharan Africa	2008	9.7127883273	0.368488175	2.11331707004029	7.59947125725972
1332	Central sub-Saharan Africa	2009	9.73638726709	0.376669289	2.11311488016516	7.62327238692484
1333	Central sub-Saharan Africa	2010	9.76881452552	0.385380946	2.10920346634721	7.65961105917279
1334	Central sub-Saharan Africa	2011	9.76795347364	0.394691759	2.11244735357917	7.65550612006083
1335	Central sub-Saharan Africa	2012	9.77154207949	0.404233879	2.11384604405158	7.65769603543842
1336	Central sub-Saharan Africa	2013	9.76094576627	0.413522207	2.11453463706258	7.64641112920742
1337	Central sub-Saharan Africa	2014	9.78398885653	0.422578651	2.11468215629522	7.66930670023478
1338	Central sub-Saharan Africa	2015	9.8266735526	0.430987531	2.11079175507864	7.71588179752136
1339	Central sub-Saharan Africa	2016	9.90443253349	0.438745917	2.11367335296811	7.79075918052189
1340	Central sub-Saharan Africa	2017	10.00516716039	0.446178585	2.11539031486899	7.88977684552101
1341	Central sub-Saharan Africa	2018	10.09407942228	0.45324977	2.11444228069047	7.97963714158953
1342	Central sub-Saharan Africa	2019	10.18411028502	0.460159559	2.11362923956074	8.07048104545926
1343	Central sub-Saharan Africa	2020	10.11753646536	0.466456368	2.11446368064606	8.00307278471394
1344	Central sub-Saharan Africa	2021	10.12412077232	0.472255651	2.11188927539583	8.01223149692417
1345	Chad	1990	15.20603297443	0.114638829	4.66634018405525	10.5396927903747
1346	Chad	1991	15.26857952424	0.117954634	4.80040312305515	10.4681764011849
1347	Chad	1992	15.29645776596	0.121132832	4.78552972266791	10.5109280432921
1348	Chad	1993	15.26020558036	0.123600072	4.78608581571673	10.4741197646433
1349	Chad	1994	15.21196571055	0.126080572	4.69423232059296	10.517733389957
1350	Chad	1995	15.08759345262	0.128289492	4.60177724454361	10.4858162080764

TableS2 Frontier analysis of Smoking-Related Low Back Pain among WCBA

	location	year	val	SDI	frontier	eff_diff
1351	Chad	1996	15.00298799077	0.130169484	4.7824346253231	10.2205533654469
1352	Chad	1997	14.92602755306	0.132136021	4.7558442223977	10.1701833306623
1353	Chad	1998	14.88787040524	0.134090304	4.73609741693596	10.151772988304
1354	Chad	1999	14.81738432315	0.135825261	4.7641023577583	10.0532819653917
1355	Chad	2000	14.82616875087	0.137412374	4.74885271759568	10.0773160332743
1356	Chad	2001	14.69181950218	0.139663692	4.73465913255047	9.95716036962953
1357	Chad	2002	14.53367126376	0.1420417	4.69610531230803	9.83756595145197
1358	Chad	2003	14.38198436233	0.145348052	4.7382728198882	9.6437115424418
1359	Chad	2004	14.23244945597	0.150910868	4.39313778539896	9.83931167057105
1360	Chad	2005	14.11278987144	0.157033036	4.09084891209871	10.0219409593413
1361	Chad	2006	13.8761953115	0.162576402	3.8957066563235	9.9804886551765
1362	Chad	2007	13.55955074164	0.168161062	3.69223561434426	9.86731512729574
1363	Chad	2008	13.15721379801	0.173396784	3.73937227204217	9.41784152596783
1364	Chad	2009	12.78256825692	0.178335361	3.6370138257821	9.1455544311379
1365	Chad	2010	12.42424688819	0.184110621	3.64773970756044	8.77650718062956
1366	Chad	2011	12.05024783473	0.189591609	3.53978633628084	8.51046149844916
1367	Chad	2012	11.64405422597	0.195262749	3.42640448012764	8.21764974584236
1368	Chad	2013	11.23950054971	0.200952884	3.51470882975905	7.72479171995095
1369	Chad	2014	10.88634459402	0.206658836	3.48260077117423	7.40374382284577
1370	Chad	2015	10.63241930906	0.212428697	3.4982575991978	7.13416170986219
1371	Chad	2016	10.32493535167	0.21753218	2.90856600494378	7.41636934672622
1372	Chad	2017	9.91801588965	0.22220695	2.98115146416099	6.93686442548901
1373	Chad	2018	9.48709329461	0.226913881	2.69817481219064	6.78891848241937
1374	Chad	2019	9.24587217647	0.231704755	2.64407664398571	6.60179553248429
1375	Chad	2020	9.15002617089	0.236103083	2.60311913236886	6.54690703852114

TableS2 Frontier analysis of Smoking-Related Low Back Pain among WCBA

	location	year	val	SDI	frontier	eff_diff
1376	Chad	2021	9.01868296066	0.240436019	2.54289889601036	6.47578406464964
1377	Chile	1990	299.29298266099	0.5864951	2.11165809747535	297.181324563515
1378	Chile	1991	301.0576403869	0.59300658	2.11381974954931	298.943820637351
1379	Chile	1992	303.84552852756	0.599106083	2.11241855962	301.73310996794
1380	Chile	1993	307.15353791946	0.604828509	2.1118421186772	305.041695800783
1381	Chile	1994	311.01247849487	0.610673933	2.11500754708563	308.897470947784
1382	Chile	1995	314.92093560735	0.617234677	2.11275107817824	312.808184529172
1383	Chile	1996	319.2440603385	0.62369446	2.11329804531605	317.130762293184
1384	Chile	1997	322.48984376556	0.630540085	2.11439872262667	320.375445042933
1385	Chile	1998	325.63177041248	0.638082758	2.11315261535595	323.518617797124
1386	Chile	1999	327.70867771121	0.645928084	2.11519591643179	325.593481794778
1387	Chile	2000	330.56497971177	0.652989028	2.11285800765943	328.452121704111
1388	Chile	2001	333.80966949222	0.66026551	2.11186007776685	331.697809414453
1389	Chile	2002	338.55822403118	0.667888408	2.11261711978579	336.445606911394
1390	Chile	2003	342.67087131463	0.673445927	2.11060855510273	340.560262759527
1391	Chile	2004	345.39310809414	0.678258212	2.11280076613194	343.280307328008
1392	Chile	2005	345.296107268	0.682589191	2.11259616787605	343.183511100124
1393	Chile	2006	343.13236160924	0.685892748	2.11204111482843	341.020320494412
1394	Chile	2007	339.16127158664	0.688930323	2.11421419600108	337.047057390639
1395	Chile	2008	335.09936599742	0.692359787	2.11536658913667	332.983999408283
1396	Chile	2009	330.72887872033	0.696602141	2.11512702782554	328.613751692504
1397	Chile	2010	326.68471015424	0.702304704	2.11049590707594	324.574214247164
1398	Chile	2011	323.10723594145	0.708597936	2.11372592749372	320.993510013956
1399	Chile	2012	318.54389881095	0.714743477	2.11384378206361	316.430055028886
1400	Chile	2013	313.37032688545	0.720448781	2.1172945714254	311.253032314025

TableS2 Frontier analysis of Smoking-Related Low Back Pain among WCBA

	location	year	val	SDI	frontier	eff_diff
1401	Chile	2014	309.19160821606	0.727275927	2.11206649003931	307.079541726021
1402	Chile	2015	306.29481270278	0.736098082	2.11454145501366	304.180271247766
1403	Chile	2016	305.98322561976	0.744972224	2.11396537849627	303.869260241264
1404	Chile	2017	307.48605054977	0.752058077	2.11283182502404	305.373218724746
1405	Chile	2018	309.6430127852	0.758625714	2.11007722021344	307.532935564987
1406	Chile	2019	310.7904639507	0.765120323	2.11454276803746	308.675921182663
1407	Chile	2020	299.42397227265	0.769213676	2.113628886356	297.310343386294
1408	Chile	2021	321.19909092946	0.771514716	2.1148423235885	319.084248605871
1409	China	1990	17.14761190036	0.458668935	2.11264421272	15.03496768764
1410	China	1991	16.32891497475	0.46730197	2.112167906115	14.216747068635
1411	China	1992	15.56997633634	0.475784031	2.11518435930629	13.4547919770337
1412	China	1993	14.98363888838	0.484353831	2.11326768733783	12.8703712010422
1413	China	1994	14.75568881522	0.492578645	2.11095390253406	12.6447349126859
1414	China	1995	14.89628256522	0.501500908	2.11681290532781	12.7794696598922
1415	China	1996	15.24656469413	0.512033996	2.11514682295588	13.1314178711741
1416	China	1997	15.4324380407	0.522326866	2.11442277897175	13.3180152617282
1417	China	1998	15.53647204198	0.531379484	2.11094339080435	13.4255286511757
1418	China	1999	15.43651105573	0.540333688	2.1114939845908	13.3250170711392
1419	China	2000	15.38900684458	0.547989193	2.11465031102222	13.2743565335578
1420	China	2001	15.27005130989	0.553680374	2.11412190972027	13.1559294001697
1421	China	2002	15.11718848697	0.560542936	2.11222371126224	13.0049647757078
1422	China	2003	14.95501737841	0.569018827	2.11576327574492	12.8392541026651
1423	China	2004	14.83755540379	0.578178091	2.11447284646077	12.7230825573292
1424	China	2005	14.82230311702	0.588052194	2.11278220607227	12.7095209109477
1425	China	2006	14.96100743065	0.59875817	2.11689164066429	12.8441157899857

TableS2 Frontier analysis of Smoking-Related Low Back Pain among WCBA

	location	year	val	SDI	frontier	eff_diff
1426	China	2007	15.24230794128	0.609520246	2.11473440194474	13.1275735393353
1427	China	2008	15.62125570952	0.619454833	2.11553128702684	13.5057244224932
1428	China	2009	16.02493630457	0.629307727	2.11498234123568	13.9099539633343
1429	China	2010	16.35229611584	0.641521443	2.11124498145473	14.2410511343853
1430	China	2011	16.61758983437	0.651447978	2.1112197297063	14.5063701046637
1431	China	2012	16.83317204141	0.657144292	2.11685005626	14.71632198515
1432	China	2013	16.9937132622	0.663103007	2.11514436172162	14.8785689004784
1433	China	2014	17.11390658438	0.668640847	2.11262867003349	15.0012779143465
1434	China	2015	17.18101983883	0.671940111	2.11518308900607	15.0658367498239
1435	China	2016	17.20510787175	0.676218437	2.11254058543049	15.0925672863195
1436	China	2017	17.18994608125	0.684619661	2.11371357068407	15.0762325105659
1437	China	2018	17.17303214245	0.693717996	2.11314477519729	15.0598873672527
1438	China	2019	17.12446825651	0.703686824	2.11153550146012	15.0129327550499
1439	China	2020	16.91834567653	0.713364585	2.11331430413338	14.8050313723966
1440	China	2021	16.56366537042	0.72162976	2.11257577869628	14.4510895917237
1441	Colombia	1990	79.39979673842	0.480720054	2.11254058543049	77.2872561529895
1442	Colombia	1991	78.99736813827	0.482339894	2.11476405145571	76.8826040868143
1443	Colombia	1992	78.89300915659	0.484572359	2.11298482288951	76.7800243337005
1444	Colombia	1993	78.85322698911	0.488229373	2.11449005442988	76.7387369346801
1445	Colombia	1994	79.32625526641	0.493278182	2.11160389879603	77.214651367614
1446	Colombia	1995	79.96767567203	0.499102757	2.11586490097	77.85181077106
1447	Colombia	1996	80.76645250764	0.504986969	2.11361138740479	78.6528411202352
1448	Colombia	1997	81.79112975334	0.511079502	2.11010185195147	79.6810279013885
1449	Colombia	1998	82.87852913033	0.517022505	2.11595371681507	80.7625754135149
1450	Colombia	1999	83.84031038377	0.52243594	2.11287315259211	81.7274372311779

TableS2 Frontier analysis of Smoking-Related Low Back Pain among WCBA

	location	year	val	SDI	frontier	eff_diff
1451	Colombia	2000	84.52095811006	0.527839094	2.11554629630169	82.4054118137583
1452	Colombia	2001	84.80167640503	0.533076501	2.11681390899762	82.6848624960324
1453	Colombia	2002	84.42128072343	0.538189342	2.11256509509912	82.3087156283309
1454	Colombia	2003	83.59866943272	0.543265131	2.11455657893636	81.4841128537836
1455	Colombia	2004	82.49303280339	0.548601832	2.11357220190373	80.3794606014863
1456	Colombia	2005	81.38457555799	0.554089191	2.1137457579352	79.2708298000548
1457	Colombia	2006	79.78378092728	0.560182735	2.11591985374451	77.6678610735355
1458	Colombia	2007	77.84450083995	0.566822644	2.11344277471432	75.7310580652357
1459	Colombia	2008	76.01157455809	0.573494652	2.11226934688013	73.8993052112099
1460	Colombia	2009	74.35451400669	0.579876469	2.11202481397615	72.2424891927138
1461	Colombia	2010	73.18228030619	0.586408108	2.11377886334042	71.0685014428496
1462	Colombia	2011	72.77318115975	0.593340477	2.11243900305986	70.6607421566901
1463	Colombia	2012	72.33035184123	0.600254238	2.11408600031716	70.2162658409128
1464	Colombia	2013	71.92110331476	0.607250637	2.11337804413301	69.807725270627
1465	Colombia	2014	71.4908902348	0.614151923	2.11442619345092	69.3764640413491
1466	Colombia	2015	70.94755669941	0.620889228	2.11219568575744	68.8353610136526
1467	Colombia	2016	70.23867469092	0.627554497	2.11172010397412	68.1269545869459
1468	Colombia	2017	69.55602485167	0.634130359	2.11590059650585	67.4401242551642
1469	Colombia	2018	68.5697765793	0.640255072	2.1142218779008	66.4555547013992
1470	Colombia	2019	67.69648063247	0.646038852	2.11239203498081	65.5840885974892
1471	Colombia	2020	67.06700730377	0.650855354	2.11283039813362	64.9541769056364
1472	Colombia	2021	67.14285637172	0.655442913	2.11223652640074	65.0306198453193
1473	Comoros	1990	10.87856749329	0.270048116	2.48573859485491	8.39282889843509
1474	Comoros	1991	10.8492547557	0.279145649	2.45792684359368	8.39132791210632
1475	Comoros	1992	10.85218893558	0.288506271	2.42730995184493	8.42487898373507

TableS2 Frontier analysis of Smoking-Related Low Back Pain among WCBA

	location	year	val	SDI	frontier	eff_diff
1476	Comoros	1993	10.85450584765	0.297607417	2.40337933745928	8.45112651019072
1477	Comoros	1994	10.8063681798	0.305706587	2.38471796764286	8.42165021215714
1478	Comoros	1995	10.70955895184	0.314220341	2.35757149173505	8.35198746010495
1479	Comoros	1996	10.73341879825	0.322249069	2.34344168061772	8.38997711763228
1480	Comoros	1997	10.6448394531	0.330148125	2.30569144563941	8.33914800746059
1481	Comoros	1998	10.58458264689	0.337517914	2.27113968158812	8.31344296530188
1482	Comoros	1999	10.58547654975	0.344774694	2.17859225348694	8.40688429626305
1483	Comoros	2000	10.67567226366	0.351875506	2.1202968042873	8.5553754593727
1484	Comoros	2001	10.69375807189	0.359211999	2.12544989854357	8.56830817334643
1485	Comoros	2002	10.70194422971	0.366486127	2.11360702593877	8.58833720377123
1486	Comoros	2003	10.71861524027	0.373410566	2.11174997970243	8.60686526056757
1487	Comoros	2004	10.75305119904	0.380035976	2.113574366948	8.639476832092
1488	Comoros	2005	10.78457364226	0.386891845	2.11346790089616	8.67110574136384
1489	Comoros	2006	10.78803232766	0.393521434	2.11346877622321	8.67456355143679
1490	Comoros	2007	10.79283300765	0.399051094	2.11080867706614	8.68202433058386
1491	Comoros	2008	10.77942379311	0.404649755	2.1138601885925	8.6655636045175
1492	Comoros	2009	10.77491018601	0.410253575	2.11176024413369	8.66314994187631
1493	Comoros	2010	10.80205204337	0.415543376	2.11165088528891	8.69040115808109
1494	Comoros	2011	10.83872707194	0.421092972	2.11598201840972	8.72274505353028
1495	Comoros	2012	10.86730770579	0.426729957	2.11337980382279	8.75392790196721
1496	Comoros	2013	10.89544648429	0.432789932	2.11605314034658	8.77939334394342
1497	Comoros	2014	10.93579655996	0.438844505	2.11519241399148	8.82060414596852
1498	Comoros	2015	10.98555763154	0.444712394	2.1173764269945	8.8681812045455
1499	Comoros	2016	11.07579116941	0.450506979	2.1124157489523	8.9633754204577
1500	Comoros	2017	11.17328762332	0.456280841	2.11607193964859	9.05721568367141

TableS2 Frontier analysis of Smoking-Related Low Back Pain among WCBA

	location	year	val	SDI	frontier	eff_diff
1501	Comoros	2018	11.27509749653	0.461951344	2.11625289349024	9.15884460303976
1502	Comoros	2019	11.37853088667	0.467388014	2.11530051072259	9.26323037594741
1503	Comoros	2020	11.44407434058	0.472003132	2.11418748250195	9.32988685807805
1504	Comoros	2021	11.47106616129	0.475978688	2.11134255439247	9.35972360689753
1505	Congo (Brazzaville)	1990	7.75059042074	0.420654655	2.1132555202181	5.63733490052191
1506	Congo (Brazzaville)	1991	7.71516611743	0.426734374	2.11298030020761	5.60218581722239
1507	Congo (Brazzaville)	1992	7.67517623369	0.431987662	2.11353354407356	5.56164268961644
1508	Congo (Brazzaville)	1993	7.65290227228	0.436606594	2.11478545371182	5.53811681856818
1509	Congo (Brazzaville)	1994	7.62848023695	0.440168347	2.11527504936969	5.51320518758031
1510	Congo (Brazzaville)	1995	7.5723084931	0.443744125	2.11127622030565	5.46103227279435
1511	Congo (Brazzaville)	1996	7.54347990595	0.447566642	2.1144330097432	5.4290468962068
1512	Congo (Brazzaville)	1997	7.54048572311	0.451026961	2.11338518465986	5.42710053845014
1513	Congo (Brazzaville)	1998	7.53564290965	0.454445045	2.11580537922693	5.41983753042307
1514	Congo (Brazzaville)	1999	7.54321549264	0.45717654	2.11427578694125	5.42893970569875
1515	Congo (Brazzaville)	2000	7.60332470629	0.460764814	2.11568607235942	5.48763863393058
1516	Congo (Brazzaville)	2001	7.59990731114	0.463751682	2.11179641404453	5.48811089709547
1517	Congo (Brazzaville)	2002	7.61800672208	0.467037268	2.11422771792747	5.50377900415253
1518	Congo (Brazzaville)	2003	7.64277897993	0.470134374	2.11513407619743	5.52764490373257
1519	Congo (Brazzaville)	2004	7.68214331303	0.473612436	2.11324056234446	5.56890275068554
1520	Congo (Brazzaville)	2005	7.74231716404	0.478137627	2.11196054892593	5.63035661511407
1521	Congo (Brazzaville)	2006	7.80468167894	0.483355822	2.11592821677524	5.68875346216476
1522	Congo (Brazzaville)	2007	7.84698624565	0.48764074	2.11470816520062	5.73227808044938
1523	Congo (Brazzaville)	2008	7.89660635027	0.492822855	2.11307916054014	5.78352718972986
1524	Congo (Brazzaville)	2009	7.96014023592	0.498299969	2.11296555576058	5.84717468015942
1525	Congo (Brazzaville)	2010	8.03415732905	0.505542849	2.1100107567315	5.9241465723185

TableS2 Frontier analysis of Smoking-Related Low Back Pain among WCBA

	location	year	val	SDI	frontier	eff_diff
1526	Congo (Brazzaville)	2011	8.13709025252	0.513815777	2.11004452047361	6.02704573204639
1527	Congo (Brazzaville)	2012	8.2402205744	0.522928283	2.11211995160955	6.12810062279046
1528	Congo (Brazzaville)	2013	8.35344545055	0.531551679	2.11273890351913	6.24070654703088
1529	Congo (Brazzaville)	2014	8.46544501985	0.540594486	2.11592847298316	6.34951654686684
1530	Congo (Brazzaville)	2015	8.58613421148	0.54841403	2.11277525099826	6.47335896048174
1531	Congo (Brazzaville)	2016	8.74200042389	0.555108608	2.11498386547341	6.62701655841659
1532	Congo (Brazzaville)	2017	8.91259198786	0.5618341	2.11486386589153	6.79772812196847
1533	Congo (Brazzaville)	2018	9.0782060297	0.56779397	2.113444033235	6.964761996465
1534	Congo (Brazzaville)	2019	9.19291502073	0.573373665	2.11687754365844	7.07603747707156
1535	Congo (Brazzaville)	2020	9.1522266623	0.578451546	2.11235302959016	7.03987363270984
1536	Congo (Brazzaville)	2021	9.18203540738	0.583075236	2.11374386870859	7.06829153867141
1537	Cook Islands	1990	108.90779532937	0.564514854	2.11368089969189	106.794114429678
1538	Cook Islands	1991	109.95930423394	0.570622776	2.1122948647292	107.847009369211
1539	Cook Islands	1992	110.62804810741	0.577123468	2.11517988791654	108.512868219493
1540	Cook Islands	1993	111.56962218976	0.584059175	2.11282879588333	109.456793393877
1541	Cook Islands	1994	113.09662221751	0.591540868	2.11444983066403	110.982172386846
1542	Cook Islands	1995	115.86532725671	0.598789968	2.11593625401542	113.749391002695
1543	Cook Islands	1996	119.38593303464	0.606414151	2.11423343384833	117.271699600792
1544	Cook Islands	1997	124.1021033029	0.613991564	2.11128883483581	121.990814468064
1545	Cook Islands	1998	128.89076401049	0.621202228	2.11638528310483	126.774378727385
1546	Cook Islands	1999	132.90823305606	0.62802903	2.10945059418097	130.798782461879
1547	Cook Islands	2000	135.10949131653	0.635365607	2.11237315715709	132.997118159373
1548	Cook Islands	2001	136.53692732697	0.644421442	2.11095328167548	134.425974045295
1549	Cook Islands	2002	136.34543283633	0.652790488	2.11202458561661	134.233408250713
1550	Cook Islands	2003	135.46068792463	0.661465038	2.11253571987156	133.348152204758

TableS2 Frontier analysis of Smoking-Related Low Back Pain among WCBA

	location	year	val	SDI	frontier	eff_diff
1551	Cook Islands	2004	134.52080952035	0.668769675	2.11306353758848	132.407745982761
1552	Cook Islands	2005	133.71480206405	0.674004103	2.11260559606889	131.602196467981
1553	Cook Islands	2006	133.07596617391	0.680644001	2.11346669387772	130.962499480032
1554	Cook Islands	2007	132.05080932655	0.687505191	2.11095023835155	129.939859088198
1555	Cook Islands	2008	130.65718015177	0.695592069	2.1124927784163	128.544687373354
1556	Cook Islands	2009	129.24142691511	0.70377698	2.11422363911615	127.127203275994
1557	Cook Islands	2010	128.21610117793	0.711861612	2.11354058605787	126.102560591872
1558	Cook Islands	2011	127.04487498857	0.719444666	2.11445022751984	124.93042476105
1559	Cook Islands	2012	125.20825785351	0.726963385	2.11041856635085	123.097839287159
1560	Cook Islands	2013	123.47342473084	0.733317015	2.11369243157652	121.359732299263
1561	Cook Islands	2014	121.8037621848	0.739595704	2.11056325069208	119.693198934108
1562	Cook Islands	2015	120.20136896482	0.746550297	2.11506020211183	118.086308762708
1563	Cook Islands	2016	118.79543029194	0.752353087	2.11280234690919	116.682627945031
1564	Cook Islands	2017	117.38045961717	0.758233572	2.1117503696269	115.268709247543
1565	Cook Islands	2018	115.84790209067	0.764041182	2.11289279828726	113.735009292383
1566	Cook Islands	2019	114.15354704661	0.769795913	2.10991915850373	112.043627888106
1567	Cook Islands	2020	112.33835181295	0.774700578	2.11458177401652	110.223770038933
1568	Cook Islands	2021	111.35868639155	0.779109955	2.11344664455431	109.245239746996
1569	Costa Rica	1990	83.0943091714	0.534125181	2.11309609435434	80.9812130770457
1570	Costa Rica	1991	83.33006073769	0.539342164	2.11455195610288	81.2155087815871
1571	Costa Rica	1992	83.63856920953	0.544624238	2.11525266169097	81.523316547839
1572	Costa Rica	1993	83.8749121062	0.549946626	2.11530399381125	81.7596081123887
1573	Costa Rica	1994	84.26186278966	0.55517125	2.11831571626839	82.1435470733916
1574	Costa Rica	1995	84.97411717626	0.560547086	2.11229039942662	82.8618267768334
1575	Costa Rica	1996	85.66721427901	0.565180862	2.11660666759395	83.5506076114161

TableS2 Frontier analysis of Smoking-Related Low Back Pain among WCBA

	location	year	val	SDI	frontier	eff_diff
1576	Costa Rica	1997	86.39514788688	0.570128417	2.11753218359638	84.2776157032836
1577	Costa Rica	1998	87.12748995677	0.576067051	2.11377375724553	85.0137161995245
1578	Costa Rica	1999	87.78813381735	0.582229492	2.11401200437557	85.6741218129744
1579	Costa Rica	2000	87.9803251044	0.588243191	2.11368134814792	85.8666437562521
1580	Costa Rica	2001	88.20468423682	0.594889115	2.114577478068	86.090106758752
1581	Costa Rica	2002	88.30811106276	0.600894433	2.11303971513529	86.1950713476247
1582	Costa Rica	2003	88.2789193461	0.605765687	2.11134414097579	86.1675752051242
1583	Costa Rica	2004	87.95461204502	0.610315699	2.11468696208103	85.839925082939
1584	Costa Rica	2005	87.58948402658	0.614412619	2.11115479690533	85.4783292296747
1585	Costa Rica	2006	86.45827486258	0.617850162	2.11347034564268	84.3448045169373
1586	Costa Rica	2007	84.4121605608	0.621200571	2.11244360082603	82.299716959974
1587	Costa Rica	2008	81.91405418057	0.62570391	2.11402631963037	79.8000278609396
1588	Costa Rica	2009	79.37433359894	0.631291379	2.11443082163222	77.2599027773078
1589	Costa Rica	2010	77.4030393656	0.636595426	2.11301999300474	75.2900193725953
1590	Costa Rica	2011	75.94707688272	0.641201295	2.11198789338613	73.8350889893339
1591	Costa Rica	2012	74.28677314882	0.646574093	2.11552441151369	72.1712487373063
1592	Costa Rica	2013	72.98130362711	0.651945813	2.11422404439415	70.8670795827158
1593	Costa Rica	2014	71.98167243212	0.656890456	2.11259330999955	69.8690791221204
1594	Costa Rica	2015	71.34635328166	0.662030136	2.11245492649218	69.2338983551678
1595	Costa Rica	2016	71.08568461159	0.667430092	2.11343414805032	68.9722504635397
1596	Costa Rica	2017	70.77032402198	0.673621392	2.11345397874085	68.6568700432392
1597	Costa Rica	2018	70.8038296799	0.681296147	2.11270486467957	68.6911248152204
1598	Costa Rica	2019	70.86776364446	0.690049088	2.11312446541253	68.7546391790475
1599	Costa Rica	2020	70.69782730386	0.696194605	2.11182117397169	68.5860061298883
1600	Costa Rica	2021	71.18883223967	0.700340477	2.11345555699798	69.075376682672

TableS2 Frontier analysis of Smoking-Related Low Back Pain among WCBA

	location	year	val	SDI	frontier	eff_diff
1601	Cote d'Ivoire	1990	12.5651839882	0.279320353	2.45197734683213	10.1132066413679
1602	Cote d'Ivoire	1991	12.92508316605	0.286492681	2.43643350832178	10.4886496577282
1603	Cote d'Ivoire	1992	13.3046295944	0.293451293	2.43495801048054	10.8696715839195
1604	Cote d'Ivoire	1993	13.64865075327	0.299743215	2.40270882503366	11.2459419282363
1605	Cote d'Ivoire	1994	13.88567868448	0.305210653	2.38277562424122	11.5029030602388
1606	Cote d'Ivoire	1995	14.12922977273	0.310105508	2.38731249494493	11.7419172777851
1607	Cote d'Ivoire	1996	14.33179262933	0.314965385	2.36310883676474	11.9686837925653
1608	Cote d'Ivoire	1997	14.53840664981	0.319843979	2.34298175627026	12.1954248935397
1609	Cote d'Ivoire	1998	14.75319555257	0.324712873	2.31232176285726	12.4408737897127
1610	Cote d'Ivoire	1999	14.84001176262	0.329201009	2.30383503285164	12.5361767297684
1611	Cote d'Ivoire	2000	14.87691023138	0.333015393	2.28834147983917	12.5885687515408
1612	Cote d'Ivoire	2001	14.79972467449	0.336545511	2.29103909326692	12.5086855812231
1613	Cote d'Ivoire	2002	14.60020721474	0.339977638	2.25156065695167	12.3486465577883
1614	Cote d'Ivoire	2003	14.27152882172	0.343348932	2.22083496699293	12.0506938547271
1615	Cote d'Ivoire	2004	13.89813950429	0.346375411	2.14632516434274	11.7518143399473
1616	Cote d'Ivoire	2005	13.5447568819	0.348727913	2.11929289319038	11.4254639887096
1617	Cote d'Ivoire	2006	13.18537165143	0.350715944	2.11965562638377	11.0657160250462
1618	Cote d'Ivoire	2007	12.7483153625	0.352727478	2.11903763552393	10.6292777269761
1619	Cote d'Ivoire	2008	12.28686559783	0.355087643	2.11350298982324	10.1733626080068
1620	Cote d'Ivoire	2009	11.8478656177	0.357938755	2.1113220927097	9.7365435249903
1621	Cote d'Ivoire	2010	11.47361024637	0.361205187	2.11487846363667	9.35873178273333
1622	Cote d'Ivoire	2011	11.14166020731	0.36408365	2.112627935314	9.029032271996
1623	Cote d'Ivoire	2012	10.79965491968	0.367831685	2.11398915519926	8.68566576448073
1624	Cote d'Ivoire	2013	10.4571608781	0.37235379	2.11318332455844	8.34397755354156
1625	Cote d'Ivoire	2014	10.21029929727	0.377575444	2.11165611434012	8.09864318292988

TableS2 Frontier analysis of Smoking-Related Low Back Pain among WCBA

	location	year	val	SDI	frontier	eff_diff
1626	Cote d'Ivoire	2015	10.07876477057	0.38349138	2.11169287724914	7.96707189332086
1627	Cote d'Ivoire	2016	10.00467504446	0.389825192	2.11276120490253	7.89191383955747
1628	Cote d'Ivoire	2017	9.98368425421	0.396693974	2.11425179107928	7.86943246313072
1629	Cote d'Ivoire	2018	9.96383043354	0.404091933	2.11702031093271	7.84681012260729
1630	Cote d'Ivoire	2019	10.00431362147	0.41172558	2.11534153355442	7.88897208791558
1631	Cote d'Ivoire	2020	10.00565650459	0.419040103	2.11197238377817	7.89368412081183
1632	Cote d'Ivoire	2021	10.02576679755	0.425941883	2.11170821594123	7.91405858160877
1633	Croatia	1990	414.4740907937	0.668906358	2.11265290194816	412.361437891752
1634	Croatia	1991	411.1619320779	0.6747246	2.11187703386917	409.050055044031
1635	Croatia	1992	407.97220724688	0.678093133	2.11295773490607	405.859249511974
1636	Croatia	1993	405.1187843696	0.678221047	2.11184207564467	403.006942293955
1637	Croatia	1994	402.72249285996	0.677548133	2.11439623868819	400.608096621272
1638	Croatia	1995	402.52977875879	0.677029465	2.11070196691558	400.419076791874
1639	Croatia	1996	402.44624565874	0.679507318	2.11398423050316	400.332261428237
1640	Croatia	1997	398.39897530085	0.68536501	2.11233351175763	396.286641789092
1641	Croatia	1998	395.00290272135	0.691365519	2.11013736834349	392.892765353007
1642	Croatia	1999	391.01960989047	0.696636411	2.11240834541762	388.907201545052
1643	Croatia	2000	388.45160916602	0.703039899	2.11213791999339	386.339471246027
1644	Croatia	2001	387.90477060466	0.709536841	2.11700850401941	385.787762100641
1645	Croatia	2002	389.00098207163	0.715383422	2.11160474371864	386.889377327911
1646	Croatia	2003	388.41070634661	0.721130377	2.11290566635134	386.297800680259
1647	Croatia	2004	388.58689657801	0.726327652	2.11279482342074	386.474101754589
1648	Croatia	2005	388.69591491467	0.73215414	2.1167355880197	386.57917932665
1649	Croatia	2006	389.40246366355	0.738066879	2.11012515842636	387.292338505124
1650	Croatia	2007	391.91148779205	0.743148777	2.1162660010624	389.795221790988

TableS2 Frontier analysis of Smoking-Related Low Back Pain among WCBA

	location	year	val	SDI	frontier	eff_diff
1651	Croatia	2008	396.03624231401	0.748469602	2.11526954608076	393.920972767929
1652	Croatia	2009	399.58114745011	0.754065605	2.11133088871042	397.4698165614
1653	Croatia	2010	402.52219517803	0.759472252	2.11362141250048	400.40857376553
1654	Croatia	2011	404.63310462343	0.763686381	2.11564486261722	402.517459760813
1655	Croatia	2012	405.09359510999	0.767405336	2.11578066187602	402.977814448114
1656	Croatia	2013	406.22808612531	0.77115188	2.11073843554919	404.117347689761
1657	Croatia	2014	407.66538523929	0.774479241	2.11340001457067	405.551985224719
1658	Croatia	2015	409.31555650487	0.777865888	2.11277082568494	407.202785679185
1659	Croatia	2016	406.70657361903	0.781443605	2.1111715335769	404.595402085453
1660	Croatia	2017	402.54630937188	0.785074537	2.11616037088137	400.430149000999
1661	Croatia	2018	398.05121054956	0.78867141	2.11130869401061	395.939901855549
1662	Croatia	2019	395.96385820796	0.792475415	2.11514422778348	393.848713980177
1663	Croatia	2020	396.32578753861	0.795462272	2.11512307449125	394.210664464119
1664	Croatia	2021	395.05436634392	0.798341027	2.11130512856396	392.943061215356
1665	Cuba	1990	104.81422195991	0.558019071	2.11179257028694	102.702429389623
1666	Cuba	1991	103.50087659135	0.563413638	2.11260838945813	101.388268201892
1667	Cuba	1992	103.2903939581	0.566169006	2.11412632896614	101.176267629134
1668	Cuba	1993	104.05220289147	0.565441047	2.11133250814362	101.940870383326
1669	Cuba	1994	106.12346452791	0.563186313	2.11341992726456	104.010044600645
1670	Cuba	1995	109.24388982595	0.560765902	2.11426703158268	107.129622794367
1671	Cuba	1996	112.50598579004	0.559346329	2.1123473292864	110.393638460754
1672	Cuba	1997	115.89555142269	0.558924568	2.11553269320628	113.780018729484
1673	Cuba	1998	118.96348299584	0.55936322	2.111469415417	116.852013580423
1674	Cuba	1999	121.99370684894	0.561820953	2.11358630944432	119.880120539496
1675	Cuba	2000	124.50162413124	0.566676902	2.11114601736681	122.390478113873

TableS2 Frontier analysis of Smoking-Related Low Back Pain among WCBA

	location	year	val	SDI	frontier	eff_diff
1676	Cuba	2001	126.62565332929	0.572753861	2.11471401570487	124.510939313585
1677	Cuba	2002	127.68441207627	0.57996829	2.11121888252	125.57319319375
1678	Cuba	2003	127.61554126064	0.587647762	2.11098340358284	125.504557857057
1679	Cuba	2004	126.57846383386	0.594348715	2.11339481043012	124.46506902343
1680	Cuba	2005	125.36284291917	0.599863799	2.1116471594034	123.251195759767
1681	Cuba	2006	123.3251598743	0.604970567	2.11392820802667	121.211231666273
1682	Cuba	2007	120.75044777611	0.607618099	2.1128851629481	118.637562613162
1683	Cuba	2008	117.73073865196	0.609400923	2.11310275427907	115.617635897681
1684	Cuba	2009	114.19975949218	0.611775641	2.11229743718444	112.087462054996
1685	Cuba	2010	111.16812649128	0.615261262	2.11538565204141	109.052740839239
1686	Cuba	2011	107.74871323894	0.62038669	2.11271350590075	105.635999733039
1687	Cuba	2012	103.92336217547	0.626156025	2.11499628135101	101.808365894119
1688	Cuba	2013	100.17702076343	0.631839801	2.11299899587477	98.0640217675552
1689	Cuba	2014	96.69892904924	0.637304129	2.11328721256759	94.5856418366724
1690	Cuba	2015	94.0857773656	0.642818963	2.11303879893625	91.9727385666638
1691	Cuba	2016	92.37646701041	0.647907866	2.11200593443139	90.2644610759786
1692	Cuba	2017	91.09746424392	0.652309286	2.11292689692897	88.984537346991
1693	Cuba	2018	90.08273446325	0.656858654	2.1178852476715	87.9648492155785
1694	Cuba	2019	89.15741816859	0.661674717	2.11466853336815	87.0427496352218
1695	Cuba	2020	88.60746466753	0.665210747	2.11175856333	86.4957061042
1696	Cuba	2021	87.40559984368	0.668729864	2.11194714824364	85.2936526954364
1697	Cyprus	1990	207.00081412195	0.648230872	2.11469283276845	204.886121289182
1698	Cyprus	1991	208.79497222226	0.655726169	2.11474983747788	206.680222384782
1699	Cyprus	1992	209.39681540608	0.666011849	2.1116375654906	207.285177840589
1700	Cyprus	1993	209.71850263881	0.677547477	2.11414956275667	207.604353076053

TableS2 Frontier analysis of Smoking-Related Low Back Pain among WCBA

	location	year	val	SDI	frontier	eff_diff
1701	Cyprus	1994	209.45750081544	0.688829361	2.11692367652164	207.340577138918
1702	Cyprus	1995	209.13740145375	0.699923898	2.11009300979377	207.027308443956
1703	Cyprus	1996	208.53007844447	0.709839946	2.11180479881403	206.418273645656
1704	Cyprus	1997	207.22771515658	0.718864244	2.11274016796186	205.114974988618
1705	Cyprus	1998	205.3642398289	0.727964382	2.11264815158365	203.251591677316
1706	Cyprus	1999	203.65237973181	0.736846135	2.11649298319869	201.535886748611
1707	Cyprus	2000	201.99522035832	0.745199301	2.11392454648907	199.881295811831
1708	Cyprus	2001	200.92846299672	0.753594717	2.11227075938382	198.816192237336
1709	Cyprus	2002	200.37628417806	0.761502465	2.11249889780236	198.263785280258
1710	Cyprus	2003	199.83348640562	0.767896216	2.11490216985087	197.718584235769
1711	Cyprus	2004	199.19465904824	0.773764775	2.11632971792246	197.078329330318
1712	Cyprus	2005	198.99370164842	0.779031361	2.1113103834791	196.882391264941
1713	Cyprus	2006	199.19423211831	0.784560965	2.11014748143014	197.08408463688
1714	Cyprus	2007	199.90087763223	0.792068383	2.11430629705078	197.786571335179
1715	Cyprus	2008	200.91676394946	0.799454089	2.11388928894543	198.802874660515
1716	Cyprus	2009	202.07428944089	0.805112085	2.11364257918453	199.960646861705
1717	Cyprus	2010	203.82432463115	0.809840188	2.11443547584053	201.709889155309
1718	Cyprus	2011	205.36836999572	0.813726132	2.11325545177548	203.255114543945
1719	Cyprus	2012	206.83877990673	0.816978525	2.11466992368974	204.72410998304
1720	Cyprus	2013	208.90524481306	0.819380602	2.11578015529076	206.789464657769
1721	Cyprus	2014	211.52134977348	0.821184302	2.11307420804191	209.408275565438
1722	Cyprus	2015	214.40440338631	0.822732851	2.11122175863455	212.293181627675
1723	Cyprus	2016	218.61112253524	0.824283624	2.11253993488873	216.498582600351
1724	Cyprus	2017	223.93191492298	0.826139878	2.11271402406349	221.819200898917
1725	Cyprus	2018	228.73947039427	0.828343243	2.11180435224673	226.627666042023

TableS2 Frontier analysis of Smoking-Related Low Back Pain among WCBA

	location	year	val	SDI	frontier	eff_diff
1726	Cyprus	2019	233.23946750823	0.830980728	2.11404747795662	231.125420030273
1727	Cyprus	2020	233.6820980432	0.833059523	2.11280234690919	231.569295696291
1728	Cyprus	2021	238.21164362246	0.835630545	2.11293851880029	236.09870510366
1729	Czechia	1990	427.23534106632	0.681848021	2.11233704695754	425.123004019362
1730	Czechia	1991	424.7952042466	0.688002859	2.11466211963889	422.680542126961
1731	Czechia	1992	421.83444495981	0.697577809	2.1117762525587	419.722668707251
1732	Czechia	1993	418.77142712413	0.710853416	2.11654345301534	416.654883671115
1733	Czechia	1994	415.61345097233	0.725969686	2.11245673991115	413.500994232419
1734	Czechia	1995	412.59178124041	0.737981722	2.11684658099667	410.474934659413
1735	Czechia	1996	407.62309029966	0.74604138	2.10966320064429	405.513427099016
1736	Czechia	1997	400.51746066835	0.751680344	2.11347207501949	398.40398859333
1737	Czechia	1998	393.61904988136	0.757033755	2.11467263406506	391.504377247295
1738	Czechia	1999	386.6526403114	0.76242888	2.113538976433	384.539101334967
1739	Czechia	2000	380.05654939327	0.767766159	2.11319258287645	377.943356810394
1740	Czechia	2001	373.34181180942	0.77216724	2.11449355954059	371.227318249879
1741	Czechia	2002	366.37075504028	0.776927934	2.11155965325025	364.25919538703
1742	Czechia	2003	361.11066684118	0.782448081	2.11063560595934	359.000031235221
1743	Czechia	2004	357.09055940725	0.787126405	2.11402830146753	354.976531105782
1744	Czechia	2005	353.90577749886	0.791333948	2.11239076434716	351.793386734513
1745	Czechia	2006	351.03797138127	0.794922784	2.11563815448863	348.922333226781
1746	Czechia	2007	348.83076840901	0.798254118	2.11460147895592	346.716166930054
1747	Czechia	2008	347.74753054344	0.801764846	2.11268015515048	345.63485038829
1748	Czechia	2009	348.40043724503	0.804962225	2.11529013432731	346.285147110703
1749	Czechia	2010	349.70308756148	0.808747328	2.11311209463841	347.589975466842
1750	Czechia	2011	350.64719893402	0.812054784	2.11538304656509	348.531815887455

TableS2 Frontier analysis of Smoking-Related Low Back Pain among WCBA

	location	year	val	SDI	frontier	eff_diff
1751	Czechia	2012	351.63501040521	0.814230401	2.11059314480823	349.524417260402
1752	Czechia	2013	352.36800001774	0.815615361	2.11391683317853	350.254083184562
1753	Czechia	2014	353.28347264972	0.816227257	2.11455922676726	351.168913422953
1754	Czechia	2015	355.09953987249	0.816564684	2.11341861698111	352.986121255509
1755	Czechia	2016	358.7541968467	0.817153026	2.11274664180148	356.641450204898
1756	Czechia	2017	362.63142881728	0.81906768	2.11511450402672	360.516314313253
1757	Czechia	2018	367.75379818364	0.822034486	2.11328657486703	365.640511608773
1758	Czechia	2019	372.05300635861	0.824784473	2.11258762164833	369.940418736962
1759	Czechia	2020	374.94858902444	0.826626631	2.11046861216347	372.838120412277
1760	Czechia	2021	376.53771131949	0.828450433	2.11321634309222	374.424494976398
1761	DR Congo	1990	8.87124020081	0.28984321	2.41760062636962	6.45363957444038
1762	DR Congo	1991	8.8429625464	0.290431567	2.41828465457082	6.42467789182918
1763	DR Congo	1992	8.81117146985	0.290959242	2.43642779973161	6.37474367011839
1764	DR Congo	1993	8.77862255948	0.28962879	2.43181164053549	6.34681091894451
1765	DR Congo	1994	8.72753241643	0.286859146	2.43650124271304	6.29103117371696
1766	DR Congo	1995	8.64591330886	0.283967532	2.44140728716294	6.20450602169706
1767	DR Congo	1996	8.58205132171	0.28041316	2.45495604822492	6.12709527348508
1768	DR Congo	1997	8.48924486352	0.276336503	2.45515450374897	6.03409035977103
1769	DR Congo	1998	8.39493561616	0.271813823	2.491157995673	5.903777620487
1770	DR Congo	1999	8.28566329871	0.267367662	2.4767534748884	5.8089098238216
1771	DR Congo	2000	8.24078412498	0.262456747	2.47780977067077	5.76297435430923
1772	DR Congo	2001	8.11456074329	0.257857505	2.51288623336365	5.60167450992635
1773	DR Congo	2002	7.98902796043	0.25413636	2.51174339058517	5.47728456984483
1774	DR Congo	2003	7.86109804965	0.252333885	2.52120386057956	5.33989418907044
1775	DR Congo	2004	7.74357124536	0.252470919	2.5470691489421	5.1965020964179

TableS2 Frontier analysis of Smoking-Related Low Back Pain among WCBA

	location	year	val	SDI	frontier	eff_diff
1776	DR Congo	2005	7.66940460667	0.254161023	2.51259138349609	5.15681322317391
1777	DR Congo	2006	7.62268589827	0.257355123	2.50837729180593	5.11430860646407
1778	DR Congo	2007	7.57537531166	0.262272267	2.4879370688552	5.0874382428048
1779	DR Congo	2008	7.52241470971	0.268103872	2.49401481489923	5.02839989481077
1780	DR Congo	2009	7.48039045903	0.274361144	2.45256298406245	5.02782747496755
1781	DR Congo	2010	7.45453093522	0.282223754	2.4589836717272	4.9955472634928
1782	DR Congo	2011	7.40313237531	0.290872953	2.43584398697108	4.96728838833892
1783	DR Congo	2012	7.35947821691	0.300454783	2.39778090308851	4.96169731382149
1784	DR Congo	2013	7.29732904749	0.310831256	2.36557487730826	4.93175417018174
1785	DR Congo	2014	7.26658414922	0.321622927	2.3348211360359	4.9317630131841
1786	DR Congo	2015	7.2427108444	0.332421307	2.29795555876507	4.94475528563493
1787	DR Congo	2016	7.25645071579	0.341988787	2.24566236392329	5.01078835186671
1788	DR Congo	2017	7.28034264912	0.351102035	2.12304499254153	5.15729765657847
1789	DR Congo	2018	7.29542704479	0.360287677	2.112632888016	5.182794156774
1790	DR Congo	2019	7.32555604174	0.369340684	2.11559411128886	5.20996193045114
1791	DR Congo	2020	7.20317245801	0.376712271	2.11593781065365	5.08723464735635
1792	DR Congo	2021	7.20818733363	0.383179849	2.11114761454167	5.09703971908833
1793	Democratic Republic of Sao Tome and Principe	1990	4.68368421046	0.309542852	2.38509259094087	2.29859161951913
1794	Democratic Republic of Sao Tome and Principe	1991	4.75525549563	0.310601028	2.36302009208354	2.39223540354646
1795	Democratic Republic of Sao Tome and Principe	1992	4.83396918498	0.311515447	2.36194060856925	2.47202857641075
1796	Democratic Republic of Sao Tome and Principe	1993	4.92188970284	0.312743558	2.35938658368821	2.56250311915179
1797	Democratic Republic of Sao Tome and Principe	1994	4.99879935506	0.314261087	2.35815560792382	2.64064374713618
1798	Democratic Republic of Sao Tome and Principe	1995	5.05858010264	0.31584392	2.36240743639771	2.69617266624229
1799	Democratic Republic of Sao Tome and Principe	1996	5.11832136434	0.317729288	2.34717513956587	2.77114622477413
1800	Democratic Republic of Sao Tome and Principe	1997	5.1800439629	0.31979567	2.34332271226225	2.83672125063775

TableS2 Frontier analysis of Smoking-Related Low Back Pain among WCBA

	location	year	val	SDI	frontier	eff_diff
1801	Democratic Republic of Sao Tome and Principe	1998	5.24815055573	0.322519219	2.34718879749676	2.90096175823323
1802	Democratic Republic of Sao Tome and Principe	1999	5.31720238183	0.325651916	2.32459421736203	2.99260816446797
1803	Democratic Republic of Sao Tome and Principe	2000	5.34974119281	0.329460109	2.30648604619071	3.04325514661929
1804	Democratic Republic of Sao Tome and Principe	2001	5.35950096007	0.334484786	2.28856864143046	3.07093231863954
1805	Democratic Republic of Sao Tome and Principe	2002	5.36410290467	0.340302837	2.24425610900037	3.11984679566963
1806	Democratic Republic of Sao Tome and Principe	2003	5.36748770633	0.346851981	2.14101746476357	3.22647024156643
1807	Democratic Republic of Sao Tome and Principe	2004	5.36537598722	0.353722384	2.12277077517352	3.24260521204648
1808	Democratic Republic of Sao Tome and Principe	2005	5.3693595278	0.361105881	2.11559851922859	3.25376100857141
1809	Democratic Republic of Sao Tome and Principe	2006	5.33680889364	0.369413007	2.11561579031299	3.22119310332701
1810	Democratic Republic of Sao Tome and Principe	2007	5.26434521966	0.377813517	2.11510052526845	3.14924469439155
1811	Democratic Republic of Sao Tome and Principe	2008	5.14961553332	0.386688944	2.11405550230436	3.03556003101564
1812	Democratic Republic of Sao Tome and Principe	2009	5.05274914691	0.395821238	2.11434780010403	2.93840134680597
1813	Democratic Republic of Sao Tome and Principe	2010	4.99062109864	0.405530666	2.11414829145369	2.87647280718631
1814	Democratic Republic of Sao Tome and Principe	2011	4.96574891204	0.415783728	2.11396938182182	2.85177953021818
1815	Democratic Republic of Sao Tome and Principe	2012	4.94395911887	0.425721315	2.11270276771369	2.83125635115631
1816	Democratic Republic of Sao Tome and Principe	2013	4.91198789781	0.43559246	2.11111731711931	2.80087058069069
1817	Democratic Republic of Sao Tome and Principe	2014	4.91089851225	0.445146237	2.11658102939455	2.79431748285545
1818	Democratic Republic of Sao Tome and Principe	2015	4.90380805471	0.454051473	2.11524924713619	2.78855880757381
1819	Democratic Republic of Sao Tome and Principe	2016	4.89627030322	0.463230082	2.115070424015	2.781199879205
1820	Democratic Republic of Sao Tome and Principe	2017	4.8716676916	0.47217093	2.11166105413806	2.76000663746194
1821	Democratic Republic of Sao Tome and Principe	2018	4.85432928565	0.481098204	2.11392606397889	2.74040322167111
1822	Democratic Republic of Sao Tome and Principe	2019	4.84381034346	0.48983823	2.11288616722741	2.73092417623259
1823	Democratic Republic of Sao Tome and Principe	2020	4.82775560325	0.497861409	2.11165368691609	2.71610191633391
1824	Democratic Republic of Sao Tome and Principe	2021	4.81086740003	0.505413747	2.11183110369	2.69903629634
1825	Denmark	1990	422.94002924293	0.801154655	2.11404090418444	420.825988338746

TableS2 Frontier analysis of Smoking-Related Low Back Pain among WCBA

	location	year	val	SDI	frontier	eff_diff
1826	Denmark	1991	428.16487551845	0.80454035	2.11369897253492	426.051176545915
1827	Denmark	1992	432.9051004792	0.808164434	2.11569315408577	430.789407325114
1828	Denmark	1993	435.7622142873	0.811585713	2.11677373895714	433.645440548343
1829	Denmark	1994	436.93638292942	0.815138614	2.11370627199646	434.822676657424
1830	Denmark	1995	436.62579874371	0.819104612	2.11273280077685	434.513065942933
1831	Denmark	1996	434.21426565162	0.823375243	2.11106453233068	432.103201119289
1832	Denmark	1997	430.75243555865	0.827679979	2.11388999996447	428.638545558686
1833	Denmark	1998	424.56407646467	0.831683237	2.11201574118889	422.452060723481
1834	Denmark	1999	417.73137549982	0.835381543	2.11389203203456	415.617483467785
1835	Denmark	2000	411.04176858297	0.839184298	2.11275892049838	408.929009662472
1836	Denmark	2001	396.75624526346	0.84317775	2.11306810318013	394.64317716028
1837	Denmark	2002	373.85229075804	0.847086357	2.11359142393418	371.738699334106
1838	Denmark	2003	347.78141169277	0.850441326	2.109881522485	345.671530170285
1839	Denmark	2004	324.54539631405	0.853304103	2.116116729769	322.429279584281
1840	Denmark	2005	311.70626665475	0.855690732	2.11137649275371	309.594890161996
1841	Denmark	2006	306.82841536269	0.85803321	2.11304985884678	304.715365503843
1842	Denmark	2007	300.97257242512	0.859781734	2.1120466936723	298.860525731448
1843	Denmark	2008	295.67983956152	0.861782061	2.112597451205	293.567242110315
1844	Denmark	2009	290.93650410291	0.86394805	2.11773105481188	288.818773048098
1845	Denmark	2010	285.97723178556	0.866767079	2.11564908423559	283.861582701324
1846	Denmark	2011	280.84716024389	0.869778682	2.11320535612094	278.733954887769
1847	Denmark	2012	275.02066022682	0.87251751	2.11416415568441	272.906496071136
1848	Denmark	2013	269.66601847504	0.874932974	2.11119325027406	267.554825224766
1849	Denmark	2014	263.45872745309	0.876814932	2.11502424452491	261.343703208565
1850	Denmark	2015	258.18123472741	0.878543945	2.11393059112383	256.067304136286

TableS2 Frontier analysis of Smoking-Related Low Back Pain among WCBA

	location	year	val	SDI	frontier	eff_diff
1851	Denmark	2016	254.60791826201	0.880589783	2.11199915394115	252.495919108069
1852	Denmark	2017	252.53447812802	0.883688487	2.11501938496277	250.419458743057
1853	Denmark	2018	251.8916155166	0.887529846	2.11211695225231	249.779498564348
1854	Denmark	2019	251.04464325616	0.891624571	2.11441422898437	248.930229027176
1855	Denmark	2020	248.67566489295	0.894368297	2.11458657898983	246.56107831396
1856	Denmark	2021	249.49320715652	0.896424204	2.11175443407346	247.381452722447
1857	Djibouti	1990	16.04889697639	0.337781789	2.2719297230828	13.7769672533072
1858	Djibouti	1991	15.16268753581	0.338770011	2.2733124162	12.88937511961
1859	Djibouti	1992	14.84580734575	0.340986061	2.24660649670873	12.5992008490413
1860	Djibouti	1993	15.13725311619	0.344904394	2.17411305903146	12.9631400571585
1861	Djibouti	1994	15.22647730392	0.347981404	2.13470791108377	13.0917693928362
1862	Djibouti	1995	15.19502729731	0.351245633	2.12632580631442	13.0687014909956
1863	Djibouti	1996	14.9821728864	0.354385634	2.11389490738303	12.868277979017
1864	Djibouti	1997	14.65832188182	0.358096401	2.11771334048074	12.5406085413393
1865	Djibouti	1998	14.27161743517	0.361596764	2.11478438470948	12.1568330504605
1866	Djibouti	1999	13.9675470108	0.365305211	2.11384047256037	11.8537065382396
1867	Djibouti	2000	13.7390027699	0.369015095	2.11305608764064	11.6259466822594
1868	Djibouti	2001	13.7313712222	0.372853155	2.11136860523784	11.6200026169622
1869	Djibouti	2002	13.67952386096	0.376650004	2.11360040915195	11.5659234518081
1870	Djibouti	2003	13.65209462888	0.380686615	2.11383529746636	11.5382593314136
1871	Djibouti	2004	13.6800245511	0.384987706	2.11328060439016	11.5667439467098
1872	Djibouti	2005	13.79013596573	0.389568706	2.11617353852135	11.6739624272086
1873	Djibouti	2006	13.94405886601	0.395037471	2.11435685265135	11.8297020133586
1874	Djibouti	2007	14.0384698187	0.400618546	2.11554317377698	11.922926644923
1875	Djibouti	2008	14.14487696111	0.406658303	2.11294443253367	12.0319325285763

TableS2 Frontier analysis of Smoking-Related Low Back Pain among WCBA

	location	year	val	SDI	frontier	eff_diff
1876	Djibouti	2009	14.2603994565	0.411969335	2.11317120981635	12.1472282466836
1877	Djibouti	2010	14.35667893852	0.41699935	2.11266815122942	12.2440107872906
1878	Djibouti	2011	14.51145206732	0.42231614	2.11126994716459	12.4001821201554
1879	Djibouti	2012	14.63755547676	0.42771144	2.11491109987205	12.5226443768879
1880	Djibouti	2013	14.7491181762	0.433360437	2.11387539947774	12.6352427767223
1881	Djibouti	2014	14.90381759868	0.439455722	2.11512572206583	12.7886918766142
1882	Djibouti	2015	15.10776171651	0.445855285	2.11449001178833	12.9932717047217
1883	Djibouti	2016	15.35653680986	0.453442854	2.11293167635753	13.2436051335025
1884	Djibouti	2017	15.57055118677	0.460440152	2.11386539109531	13.4566857956747
1885	Djibouti	2018	15.82967414798	0.467705672	2.11545089417208	13.7142232538079
1886	Djibouti	2019	16.00713440089	0.475263243	2.11603651776241	13.8910978831276
1887	Djibouti	2020	16.09428436562	0.481879945	2.11089233929362	13.9833920263264
1888	Djibouti	2021	16.18632301261	0.487958371	2.1151519295424	14.0711710830676
1889	Dominica	1990	31.76086841997	0.56360259	2.1127050368191	29.6481633831509
1890	Dominica	1991	32.33850174661	0.565237843	2.11115027507962	30.2273514715304
1891	Dominica	1992	32.91717254392	0.569570684	2.1109238853035	30.8062486586165
1892	Dominica	1993	33.46303087585	0.578811811	2.11192499466507	31.3511058811849
1893	Dominica	1994	33.83653518908	0.590119147	2.11306155232	31.72347363676
1894	Dominica	1995	34.22688462559	0.601986932	2.11640401170781	32.1104806138822
1895	Dominica	1996	34.37978463974	0.61438205	2.11224988143741	32.2675347583026
1896	Dominica	1997	34.37484034427	0.625057353	2.11370436648634	32.2611359777837
1897	Dominica	1998	34.24497204759	0.633017994	2.11342956290407	32.1315424846859
1898	Dominica	1999	34.16938526977	0.639453915	2.11332080331659	32.0560644664534
1899	Dominica	2000	34.25999859972	0.645830937	2.11247455525829	32.1475240444617
1900	Dominica	2001	34.30719380675	0.652368567	2.11264383209579	32.1945499746542

TableS2 Frontier analysis of Smoking-Related Low Back Pain among WCBA

	location	year	val	SDI	frontier	eff_diff
1901	Dominica	2002	34.16888584656	0.658423554	2.11433753566056	32.0545483108994
1902	Dominica	2003	34.05256402673	0.663678868	2.11080957832595	31.9417544484041
1903	Dominica	2004	33.80832261847	0.668952132	2.116516392235	31.691806226235
1904	Dominica	2005	33.69676989633	0.674549077	2.11205581772524	31.5847140786048
1905	Dominica	2006	33.68220153522	0.680342482	2.11254537838594	31.5696561568341
1906	Dominica	2007	33.59068899239	0.685415917	2.11382668846145	31.4768623039285
1907	Dominica	2008	33.48514725633	0.690197519	2.11324128524355	31.3719059710864
1908	Dominica	2009	33.45515467215	0.694461869	2.11576239788268	31.3393922742673
1909	Dominica	2010	33.48366822542	0.698489193	2.11340673954944	31.3702614858706
1910	Dominica	2011	33.53215503468	0.70246326	2.11518024741851	31.4169747872615
1911	Dominica	2012	33.65939176151	0.706870608	2.11479982989219	31.5445919316178
1912	Dominica	2013	33.68743571841	0.712210475	2.11547514741557	31.5719605709944
1913	Dominica	2014	33.64176944364	0.718455556	2.11257575129649	31.5291936923435
1914	Dominica	2015	33.56588974356	0.724785536	2.11537319691268	31.4505165466473
1915	Dominica	2016	33.40122240456	0.731760801	2.11680138653317	31.2844210180268
1916	Dominica	2017	33.18259490665	0.736062473	2.11783172624371	31.0647631804063
1917	Dominica	2018	33.01454623599	0.738935954	2.11267326734189	30.9018729686481
1918	Dominica	2019	32.85150712105	0.742132066	2.11268967380067	30.7388174472493
1919	Dominica	2020	32.68606677517	0.744419944	2.11191825836534	30.5741485168047
1920	Dominica	2021	32.47036205712	0.746967185	2.11323637693643	30.3571256801836
1921	Dominican Republic	1990	57.85729746025	0.442654076	2.11619535573662	55.7411021045134
1922	Dominican Republic	1991	59.62301181185	0.446144578	2.11221611589477	57.5107956959552
1923	Dominican Republic	1992	61.26980069472	0.449953081	2.11074052808286	59.1590601666371
1924	Dominican Republic	1993	62.97920018178	0.454229297	2.11538386859464	60.8638163131854
1925	Dominican Republic	1994	64.66527390023	0.458620614	2.11172315617606	62.5535507440539

TableS2 Frontier analysis of Smoking-Related Low Back Pain among WCBA

	location	year	val	SDI	frontier	eff_diff
1926	Dominican Republic	1995	66.23939294793	0.463313869	2.1109020615645	64.1284908863655
1927	Dominican Republic	1996	67.44629147648	0.468516938	2.11162410804118	65.3346673684388
1928	Dominican Republic	1997	68.87079933878	0.474504389	2.11258611555775	66.7582132232223
1929	Dominican Republic	1998	69.74911108442	0.481170939	2.11272885329625	67.6363822311237
1930	Dominican Republic	1999	70.72644015732	0.488178541	2.1123728436758	68.6140673136442
1931	Dominican Republic	2000	71.68888173543	0.495468586	2.11822033540435	69.5706614000256
1932	Dominican Republic	2001	72.2454797251	0.502775415	2.11509944672482	70.1303802783752
1933	Dominican Republic	2002	72.49154988862	0.510298019	2.11320384758324	70.3783460410368
1934	Dominican Republic	2003	72.52304451594	0.517395574	2.11301323150823	70.4100312844318
1935	Dominican Republic	2004	72.36595497593	0.524543275	2.11281194327559	70.2531430326544
1936	Dominican Republic	2005	72.00859917672	0.532228649	2.11487821689081	69.8937209598292
1937	Dominican Republic	2006	71.02531369459	0.54022269	2.11571941443368	68.9095942801563
1938	Dominican Republic	2007	69.46806725584	0.548187021	2.11309351153743	67.3549737443026
1939	Dominican Republic	2008	67.74938683582	0.555371313	2.11419379420338	65.6351930416166
1940	Dominican Republic	2009	65.91435024665	0.561783754	2.11549169973014	63.7988585469199
1941	Dominican Republic	2010	64.05634003469	0.568130582	2.11499033214712	61.9413497025429
1942	Dominican Republic	2011	62.26339614153	0.573834813	2.1117216293873	60.1516745121427
1943	Dominican Republic	2012	60.26278120456	0.578966381	2.11326304580771	58.1495181587523
1944	Dominican Republic	2013	58.42534328673	0.583625825	2.11180292250224	56.3135403642278
1945	Dominican Republic	2014	56.68855323998	0.588236743	2.11334068201891	54.5752125579611
1946	Dominican Republic	2015	55.51409710375	0.592924164	2.11452469009274	53.3995724136573
1947	Dominican Republic	2016	54.79364582402	0.597619609	2.11293881980241	52.6807070042176
1948	Dominican Republic	2017	54.45548299474	0.602140291	2.1124612619558	52.3430217327842
1949	Dominican Republic	2018	54.40033417044	0.606960839	2.11221006685886	52.2881241035811
1950	Dominican Republic	2019	54.23289830641	0.611913021	2.11202965320576	52.1208686532042

TableS2 Frontier analysis of Smoking-Related Low Back Pain among WCBA

	location	year	val	SDI	frontier	eff_diff
1951	Dominican Republic	2020	53.98539130956	0.615635625	2.11557328536387	51.8698180241961
1952	Dominican Republic	2021	53.68621123845	0.619388201	2.11490608563988	51.5713051528101
1953	East Asia	1990	17.80878786584	0.471179162	2.11123637750227	15.6975514883377
1954	East Asia	1991	17.0322821964	0.479935584	2.11406067982914	14.9182215165709
1955	East Asia	1992	16.31617258477	0.488431396	2.11147714502871	14.2046954397413
1956	East Asia	1993	15.76849110849	0.496859473	2.1151240136391	13.6533670948509
1957	East Asia	1994	15.56234906717	0.504885881	2.11522675562627	13.4471223115437
1958	East Asia	1995	15.71741042016	0.513530275	2.11567566013211	13.6017347600279
1959	East Asia	1996	16.0735087976	0.5237577	2.11194714824364	13.9615616493564
1960	East Asia	1997	16.2684128947	0.533815513	2.11328240558719	14.1551304891128
1961	East Asia	1998	16.38317554231	0.542652548	2.11192034246811	14.2712551998419
1962	East Asia	1999	16.30084690197	0.551367028	2.11419331878718	14.1866535831828
1963	East Asia	2000	16.26198542938	0.558815666	2.11125263859831	14.1507327907817
1964	East Asia	2001	16.14242948563	0.564234748	2.11299696162753	14.0294325240025
1965	East Asia	2002	15.98716862334	0.570798377	2.11205398932107	13.8751146340189
1966	East Asia	2003	15.81933527465	0.578904729	2.11178613396635	13.7075491406836
1967	East Asia	2004	15.69414939582	0.587682668	2.11481739417756	13.5793320016424
1968	East Asia	2005	15.66870430542	0.597096172	2.11058376235913	13.5581205430609
1969	East Asia	2006	15.79335309138	0.607273415	2.11183120770148	13.6815218836785
1970	East Asia	2007	16.05415921468	0.617523836	2.1140460725693	13.9401131421107
1971	East Asia	2008	16.40710432541	0.626955795	2.11218058181163	14.2949237435984
1972	East Asia	2009	16.78112871616	0.636271151	2.113040322222	14.668088393938
1973	East Asia	2010	17.08731854119	0.64798501	2.11406955838466	14.9732489828053
1974	East Asia	2011	17.33520243031	0.65750207	2.11199716994971	15.2232052603603
1975	East Asia	2012	17.53408785187	0.662929873	2.11532747130203	15.418760380568

TableS2 Frontier analysis of Smoking-Related Low Back Pain among WCBA

	location	year	val	SDI	frontier	eff_diff
1976	East Asia	2013	17.67834644709	0.668681911	2.11384605964051	15.5645003874495
1977	East Asia	2014	17.78818247348	0.67406275	2.11157330012255	15.6766091733574
1978	East Asia	2015	17.8498390533	0.677306858	2.11199592884716	15.7378431244528
1979	East Asia	2016	17.87157620266	0.681528332	2.10981477484919	15.7617614278108
1980	East Asia	2017	17.85598768777	0.689747572	2.11407682322768	15.7419108645423
1981	East Asia	2018	17.83977365462	0.698625829	2.1144296076873	15.7253440469327
1982	East Asia	2019	17.78955775189	0.708327813	2.1132646173134	15.6762931345766
1983	East Asia	2020	17.58900769082	0.717709308	2.11587517903367	15.4731325117863
1984	East Asia	2021	17.2381511016	0.725704902	2.1112972769127	15.1268538246873
1985	Eastern Europe	1990	143.97031080447	0.664250165	2.11455459087067	141.855756213599
1986	Eastern Europe	1991	142.70853182265	0.671452375	2.11485311955702	140.593678703093
1987	Eastern Europe	1992	142.47961243291	0.678752887	2.11292710824449	140.366685324666
1988	Eastern Europe	1993	143.62980475807	0.683359195	2.11443523976176	141.515369518308
1989	Eastern Europe	1994	145.57898526103	0.686011586	2.11415070936762	143.464834551662
1990	Eastern Europe	1995	148.00670352197	0.689459344	2.11182830556676	145.894875216403
1991	Eastern Europe	1996	151.47948275399	0.692791353	2.11319042161938	149.366292332371
1992	Eastern Europe	1997	155.89155078833	0.695202953	2.11310385742253	153.778446930907
1993	Eastern Europe	1998	160.98535260273	0.696939833	2.11503458619283	158.870318016537
1994	Eastern Europe	1999	165.77867491364	0.69881365	2.11232193718194	163.666352976458
1995	Eastern Europe	2000	169.68508856955	0.70101118	2.1137400446419	167.571348524908
1996	Eastern Europe	2001	173.84422604014	0.703506217	2.11396758911118	171.730258451029
1997	Eastern Europe	2002	179.08103442054	0.706727141	2.11205500565815	176.968979414882
1998	Eastern Europe	2003	184.62764299954	0.71202517	2.11129368792339	182.516349311617
1999	Eastern Europe	2004	189.83692299265	0.719215436	2.11188078058899	187.725042212061
2000	Eastern Europe	2005	194.13875398126	0.726265709	2.11122756896241	192.027526412298

TableS2 Frontier analysis of Smoking-Related Low Back Pain among WCBA

	location	year	val	SDI	frontier	eff_diff
2001	Eastern Europe	2006	198.48528607102	0.732617452	2.11564143280699	196.369644638213
2002	Eastern Europe	2007	203.60603352489	0.738786858	2.1160728183723	201.489960706518
2003	Eastern Europe	2008	208.62252146704	0.745450424	2.11209964235944	206.510421824681
2004	Eastern Europe	2009	212.8495179049	0.751070158	2.11682242145102	210.732695483449
2005	Eastern Europe	2010	215.67067986041	0.756606481	2.11778040741683	213.552899452993
2006	Eastern Europe	2011	217.33056727882	0.761025305	2.1148211557535	215.215746123067
2007	Eastern Europe	2012	218.8927935689	0.76542275	2.11537399428671	216.777419574613
2008	Eastern Europe	2013	220.66558906667	0.770338293	2.11094080708455	218.554648259585
2009	Eastern Europe	2014	222.7275419067	0.775091704	2.11371965039762	220.613822256302
2010	Eastern Europe	2015	225.13119133416	0.779663484	2.11185425433945	223.019337079821
2011	Eastern Europe	2016	226.97724036423	0.784804691	2.11456345917797	224.862676905052
2012	Eastern Europe	2017	227.58957096856	0.790014867	2.11509643891381	225.474474529646
2013	Eastern Europe	2018	227.72002713394	0.794485575	2.1104741800462	225.609552953894
2014	Eastern Europe	2019	228.10982485067	0.798138377	2.11478246124615	225.995042389424
2015	Eastern Europe	2020	228.36227915827	0.800505061	2.1144595861174	226.247819572153
2016	Eastern Europe	2021	226.91913960283	0.802851009	2.11091674059868	224.808222862231
2017	Eastern sub-Saharan Africa	1990	14.65024006351	0.233622123	2.62821387430662	12.0220261892034
2018	Eastern sub-Saharan Africa	1991	14.63704955438	0.236340461	2.61451527621829	12.0225342781617
2019	Eastern sub-Saharan Africa	1992	14.61576225039	0.238615291	2.57208888630912	12.0436733640809
2020	Eastern sub-Saharan Africa	1993	14.60302580483	0.240816973	2.53942766570676	12.0635981391232
2021	Eastern sub-Saharan Africa	1994	14.51844398541	0.242901814	2.56312328167744	11.9553207037326
2022	Eastern sub-Saharan Africa	1995	14.34717529606	0.245423542	2.558727981293	11.788447314767
2023	Eastern sub-Saharan Africa	1996	14.24950069566	0.24858486	2.54533160210103	11.704169093559
2024	Eastern sub-Saharan Africa	1997	14.21294959888	0.252144544	2.51235444764554	11.7005951512345
2025	Eastern sub-Saharan Africa	1998	14.16941009253	0.255890509	2.53278292558472	11.6366271669453

TableS2 Frontier analysis of Smoking-Related Low Back Pain among WCBA

	location	year	val	SDI	frontier	eff_diff
2026	Eastern sub-Saharan Africa	1999	14.11990810996	0.259957122	2.52328707853708	11.5966210314229
2027	Eastern sub-Saharan Africa	2000	14.09274706812	0.264165312	2.50462237280154	11.5881246953185
2028	Eastern sub-Saharan Africa	2001	14.03410045803	0.26877226	2.50167333112509	11.5324271269049
2029	Eastern sub-Saharan Africa	2002	13.97242584123	0.273484445	2.45549689025102	11.516928950979
2030	Eastern sub-Saharan Africa	2003	13.95252239938	0.278433675	2.46116418273554	11.4913582166445
2031	Eastern sub-Saharan Africa	2004	13.92391215232	0.283936614	2.4390302638424	11.4848818884776
2032	Eastern sub-Saharan Africa	2005	13.8943297652	0.28997039	2.43605154968027	11.4582782155197
2033	Eastern sub-Saharan Africa	2006	13.81702148829	0.296455257	2.40311706654987	11.4139044217401
2034	Eastern sub-Saharan Africa	2007	13.73550233474	0.30357591	2.38164193954687	11.3538603951931
2035	Eastern sub-Saharan Africa	2008	13.63605622026	0.310918495	2.35861421306129	11.2774420071987
2036	Eastern sub-Saharan Africa	2009	13.54008456259	0.318456773	2.344001815166	11.196082747424
2037	Eastern sub-Saharan Africa	2010	13.48057657754	0.326295972	2.32162822349222	11.1589483540478
2038	Eastern sub-Saharan Africa	2011	13.43267905652	0.334253401	2.28866172813153	11.1440173283885
2039	Eastern sub-Saharan Africa	2012	13.35693334492	0.341628076	2.24193307007474	11.1150002748453
2040	Eastern sub-Saharan Africa	2013	13.27446201958	0.3492073	2.11411286832409	11.1603491512559
2041	Eastern sub-Saharan Africa	2014	13.20202956849	0.35700952	2.11355595338375	11.0884736151063
2042	Eastern sub-Saharan Africa	2015	13.1615651716	0.364907041	2.1123829716592	11.0491821999408
2043	Eastern sub-Saharan Africa	2016	13.16565913819	0.372758832	2.11251870687158	11.0531404313184
2044	Eastern sub-Saharan Africa	2017	13.19954827654	0.380499991	2.11424684154547	11.0853014349945
2045	Eastern sub-Saharan Africa	2018	13.23735434366	0.388355112	2.1154184026884	11.1219359409716
2046	Eastern sub-Saharan Africa	2019	13.23808665185	0.396344656	2.11115593453465	11.1269307173153
2047	Eastern sub-Saharan Africa	2020	13.14042371725	0.403480212	2.11409437035861	11.0263293468914
2048	Eastern sub-Saharan Africa	2021	13.03835166205	0.409720983	2.11704718729747	10.9213044747525
2049	Ecuador	1990	26.02514627336	0.518430614	2.11328511621089	23.9118611571491
2050	Ecuador	1991	25.7756583796	0.517679421	2.11452901987149	23.6611293597285

TableS2 Frontier analysis of Smoking-Related Low Back Pain among WCBA

	location	year	val	SDI	frontier	eff_diff
2051	Ecuador	1992	25.59334333617	0.51837907	2.11530680215513	23.4780365340149
2052	Ecuador	1993	25.45627275831	0.520707291	2.11344787666333	23.3428248816467
2053	Ecuador	1994	25.29834164705	0.524908291	2.11637330824236	23.1819683388076
2054	Ecuador	1995	25.16155969414	0.529380752	2.11431785358523	23.0472418405548
2055	Ecuador	1996	24.63202577743	0.532691399	2.11131542597171	22.5207103514583
2056	Ecuador	1997	23.6195840456	0.535349652	2.11431353756615	21.5052705080338
2057	Ecuador	1998	22.60101317258	0.53719633	2.11266389836	20.48834927422
2058	Ecuador	1999	21.69079781457	0.539032308	2.11134841544351	19.5794493991265
2059	Ecuador	2000	21.24428366845	0.543044779	2.1172028788418	19.1270807896082
2060	Ecuador	2001	21.12761486912	0.54855215	2.11242182926247	19.0151930398575
2061	Ecuador	2002	21.06641380207	0.554897325	2.11261288488633	18.9538009171837
2062	Ecuador	2003	21.03342190336	0.560955485	2.11104923098413	18.9223726723759
2063	Ecuador	2004	20.9201179606	0.566290293	2.11478677947163	18.8053311811284
2064	Ecuador	2005	20.80820812603	0.569927345	2.11456197700093	18.6936461490291
2065	Ecuador	2006	20.6904807032	0.572516273	2.11370123873017	18.5767794644698
2066	Ecuador	2007	20.57409085211	0.574704702	2.11207446438905	18.4620163877209
2067	Ecuador	2008	20.47784827558	0.57812504	2.11377617724063	18.3640720983394
2068	Ecuador	2009	20.33230835716	0.582484258	2.116632805339	18.215675551821
2069	Ecuador	2010	20.16831034823	0.588763056	2.11443400304862	18.0538763451814
2070	Ecuador	2011	19.90240823641	0.596729076	2.1141985039872	17.7882097324228
2071	Ecuador	2012	19.63139960746	0.605454319	2.1151292087727	17.5162703986873
2072	Ecuador	2013	19.32649247522	0.614080275	2.11459892404435	17.2118935511756
2073	Ecuador	2014	19.02204250334	0.622027433	2.11198764239162	16.9100548609484
2074	Ecuador	2015	18.80615474668	0.62854671	2.11567728585913	16.6904774608209
2075	Ecuador	2016	18.74631214719	0.634281143	2.11526761982323	16.6310445273668

TableS2 Frontier analysis of Smoking-Related Low Back Pain among WCBA

	location	year	val	SDI	frontier	eff_diff
2076	Ecuador	2017	18.82236926432	0.640011219	2.11029140060164	16.7120778637184
2077	Ecuador	2018	18.91424351517	0.64588852	2.11192756408078	16.8023159510892
2078	Ecuador	2019	18.83808046524	0.651787987	2.11343294192045	16.7246475233195
2079	Ecuador	2020	18.60934609729	0.656714457	2.11403444700794	16.4953116502821
2080	Ecuador	2021	18.49997570619	0.661017053	2.11366723857154	16.3863084676185
2081	Egypt	1990	7.33400038666	0.417182742	2.11312547263526	5.22087491402474
2082	Egypt	1991	7.39097218861	0.426909452	2.11134623540016	5.27962595320984
2083	Egypt	1992	7.43596521472	0.437744555	2.11208910793562	5.32387610678438
2084	Egypt	1993	7.49634227148	0.448490053	2.11568759838547	5.38065467309454
2085	Egypt	1994	7.56151511639	0.458727004	2.11294400132585	5.44857111506415
2086	Egypt	1995	7.63378997666	0.468035368	2.11262522909822	5.52116474756178
2087	Egypt	1996	7.68758530265	0.476116627	2.11043209405527	5.57715320859473
2088	Egypt	1997	7.78919926163	0.482970886	2.11297314938896	5.67622611224104
2089	Egypt	1998	7.83033998305	0.489184071	2.11452550332782	5.71581447972218
2090	Egypt	1999	7.86565966131	0.495502619	2.11092169050782	5.75473797080218
2091	Egypt	2000	7.88360139241	0.502169886	2.11328649387908	5.77031489853092
2092	Egypt	2001	7.89578044898	0.508716824	2.11282031652761	5.78296013245239
2093	Egypt	2002	7.90782858116	0.515038129	2.11356746635273	5.79426111480727
2094	Egypt	2003	7.94819741203	0.520623395	2.11430261278077	5.83389479924923
2095	Egypt	2004	7.99037275417	0.525181678	2.11461416194626	5.87575859222374
2096	Egypt	2005	8.0201577327	0.528665483	2.11107966588956	5.90907806681044
2097	Egypt	2006	7.99701265999	0.530395807	2.11518553155737	5.88182712843263
2098	Egypt	2007	7.90254931206	0.529457594	2.11125188297534	5.79129742908466
2099	Egypt	2008	7.78621505786	0.524743833	2.11463038658289	5.67158467127711
2100	Egypt	2009	7.64976049986	0.516255831	2.11229557134115	5.53746492851885

TableS2 Frontier analysis of Smoking-Related Low Back Pain among WCBA

	location	year	val	SDI	frontier	eff_diff
2101	Egypt	2010	7.56573521677	0.507715477	2.11523508565141	5.45050013111859
2102	Egypt	2011	7.49559302358	0.504594044	2.11270545046893	5.38288757311107
2103	Egypt	2012	7.40333693924	0.509157068	2.11286267325597	5.29047426598403
2104	Egypt	2013	7.32215259817	0.519334464	2.11513674107149	5.20701585709851
2105	Egypt	2014	7.22971590466	0.531749809	2.111349357095	5.118366547565
2106	Egypt	2015	7.21087524045	0.544370149	2.11163584313812	5.09923939731187
2107	Egypt	2016	7.21873482697	0.556012469	2.11558890000875	5.10314592696125
2108	Egypt	2017	7.23642160818	0.566914632	2.11311471375694	5.12330689442306
2109	Egypt	2018	7.26740588489	0.577442786	2.11371532903378	5.15369055585622
2110	Egypt	2019	7.3114351062	0.587736877	2.11290989968114	5.19852520651886
2111	Egypt	2020	7.33972719535	0.597363341	2.11195681407373	5.22777038127627
2112	Egypt	2021	7.3766627756	0.606787094	2.11317402162853	5.26348875397147
2113	El Salvador	1990	29.59242628999	0.373057975	2.11524008733923	27.4771862026508
2114	El Salvador	1991	29.48824795275	0.374855325	2.11165293050469	27.3765950222453
2115	El Salvador	1992	29.40094367001	0.377076643	2.11342225995076	27.2875214100592
2116	El Salvador	1993	29.44700044963	0.380307326	2.11340219798387	27.3335982516461
2117	El Salvador	1994	29.59579652924	0.385149094	2.11266451356744	27.4831320156726
2118	El Salvador	1995	29.81321544192	0.391780266	2.11722312685235	27.6959923150677
2119	El Salvador	1996	30.20330238464	0.400178468	2.11578563755273	28.0875167470873
2120	El Salvador	1997	30.75252626135	0.410455826	2.11288711165093	28.6396391496991
2121	El Salvador	1998	31.34673934312	0.42142119	2.11298216354833	29.2337571795717
2122	El Salvador	1999	32.12667408378	0.432733148	2.11549826000774	30.0111758237723
2123	El Salvador	2000	32.98067341493	0.443962666	2.11434025016553	30.8663331647645
2124	El Salvador	2001	33.75211480631	0.454177659	2.1134817265275	31.6386330797825
2125	El Salvador	2002	34.60259450573	0.46296886	2.11359605041074	32.4889984553193

TableS2 Frontier analysis of Smoking-Related Low Back Pain among WCBA

	location	year	val	SDI	frontier	eff_diff
2126	El Salvador	2003	35.50823995036	0.470397413	2.11309108017581	33.3951488701842
2127	El Salvador	2004	36.26661008674	0.476536664	2.11577459232155	34.1508354944184
2128	El Salvador	2005	36.85837834796	0.482041719	2.11180399344867	34.7465743545113
2129	El Salvador	2006	37.26012767942	0.486826	2.11200995473596	35.148117724684
2130	El Salvador	2007	37.67314991053	0.490809752	2.11605482817762	35.5570950823524
2131	El Salvador	2008	38.0314106872	0.495077739	2.11631690313964	35.9150937840604
2132	El Salvador	2009	38.28406298921	0.499489714	2.11351724758176	36.1705457416282
2133	El Salvador	2010	38.4303658038	0.504171335	2.11650826681527	36.3138575369847
2134	El Salvador	2011	38.357058973	0.508969239	2.11626042994625	36.2407985430537
2135	El Salvador	2012	38.13523714018	0.513396874	2.11645086380133	36.0187862763787
2136	El Salvador	2013	37.8510526045	0.518121908	2.11415670616137	35.7368958983386
2137	El Salvador	2014	37.59845577055	0.523468901	2.11672471702092	35.4817310535291
2138	El Salvador	2015	37.532005131	0.52938695	2.11273987797114	35.4192652530289
2139	El Salvador	2016	37.58757571289	0.535650269	2.11584112334539	35.4717345895446
2140	El Salvador	2017	37.86048165173	0.541998957	2.11302273522492	35.7474589165051
2141	El Salvador	2018	38.20098025688	0.548143174	2.11092325345894	36.0900570034211
2142	El Salvador	2019	38.57250271334	0.554031648	2.11397711320444	36.4585256001356
2143	El Salvador	2020	38.70252300672	0.558919894	2.11718985094861	36.5853331557714
2144	El Salvador	2021	39.02339913795	0.563775188	2.11470738200179	36.9086917559482
2145	Equatorial Guinea	1990	8.95829867143	0.268783629	2.47295106402455	6.48534760740545
2146	Equatorial Guinea	1991	8.86074015304	0.275296992	2.45602874152882	6.40471141151118
2147	Equatorial Guinea	1992	8.74804917701	0.283487764	2.45764690819557	6.29040226881443
2148	Equatorial Guinea	1993	8.63888827991	0.292236032	2.43786615422494	6.20102212568506
2149	Equatorial Guinea	1994	8.51721022326	0.30068634	2.40563436801642	6.11157585524358
2150	Equatorial Guinea	1995	8.40673621035	0.310443551	2.35738805822397	6.04934815212603

TableS2 Frontier analysis of Smoking-Related Low Back Pain among WCBA

	location	year	val	SDI	frontier	eff_diff
2151	Equatorial Guinea	1996	8.31655682059	0.324601221	2.31653839757797	6.00001842301203
2152	Equatorial Guinea	1997	8.2231592392	0.350550963	2.12105547440323	6.10210376479677
2153	Equatorial Guinea	1998	8.16110611067	0.371237163	2.11281278414851	6.04829332652149
2154	Equatorial Guinea	1999	8.13450339495	0.391128023	2.11115399497576	6.02334939997424
2155	Equatorial Guinea	2000	8.08758272889	0.41138345	2.11179471661057	5.97578801227943
2156	Equatorial Guinea	2001	7.96335186395	0.433590057	2.11290025069667	5.85045161325333
2157	Equatorial Guinea	2002	7.84657494102	0.451939	2.11382552643472	5.73274941458528
2158	Equatorial Guinea	2003	7.74314092385	0.467846833	2.11414808265771	5.62899284119229
2159	Equatorial Guinea	2004	7.63882576682	0.483879612	2.11358048972947	5.52524527709053
2160	Equatorial Guinea	2005	7.57075182568	0.498683715	2.11272517490442	5.45802665077558
2161	Equatorial Guinea	2006	7.52942217689	0.511579828	2.11208599035917	5.41733618653083
2162	Equatorial Guinea	2007	7.52638411154	0.524076461	2.11707780165473	5.40930630988527
2163	Equatorial Guinea	2008	7.49384664951	0.53701899	2.11279313197471	5.38105351753529
2164	Equatorial Guinea	2009	7.50143943468	0.548514683	2.11275208893733	5.38868734574267
2165	Equatorial Guinea	2010	7.51087448304	0.559109377	2.11256453645842	5.39830994658158
2166	Equatorial Guinea	2011	7.50455007962	0.570277424	2.11227825405159	5.39227182556841
2167	Equatorial Guinea	2012	7.48990272907	0.581833899	2.11251939300742	5.37738333606258
2168	Equatorial Guinea	2013	7.48434684835	0.592800613	2.11543713877293	5.36890970957707
2169	Equatorial Guinea	2014	7.48895298731	0.603473167	2.11457512630471	5.37437786100529
2170	Equatorial Guinea	2015	7.50801615245	0.613134624	2.11173002202795	5.39628613042205
2171	Equatorial Guinea	2016	7.56629159032	0.621858157	2.11478731587894	5.45150427444106
2172	Equatorial Guinea	2017	7.64055044003	0.630234538	2.11510857487494	5.52544186515506
2173	Equatorial Guinea	2018	7.69560484653	0.638022823	2.11214819485577	5.58345665167423
2174	Equatorial Guinea	2019	7.75431000128	0.645303093	2.11462038275121	5.63968961852879
2175	Equatorial Guinea	2020	7.69815157176	0.652124903	2.11360376410048	5.58454780765952

TableS2 Frontier analysis of Smoking-Related Low Back Pain among WCBA

	location	year	val	SDI	frontier	eff_diff
2176	Equatorial Guinea	2021	7.74700384095	0.657857456	2.11236608296708	5.63463775798292
2177	Eritrea	1990	2.62751555786	0.216028239	2.62751555786	0
2178	Eritrea	1991	2.59862861237	0.222574737	2.59650102382452	0.00212758854548145
2179	Eritrea	1992	2.56257927384	0.230296066	2.56232247328269	0.000256800557313586
2180	Eritrea	1993	2.52816799916	0.239385041	2.52220320270946	0.00596479645054071
2181	Eritrea	1994	2.49913693305	0.250752541	2.49913693305	0
2182	Eritrea	1995	2.46826516978	0.261498485	2.46196668570551	0.00629848407449352
2183	Eritrea	1996	2.44340796888	0.272527055	2.43722060221811	0.00618736666188813
2184	Eritrea	1997	2.41706136009	0.283551721	2.414906028355	0.00215533173500004
2185	Eritrea	1998	2.39125529679	0.293764828	2.39125529679	0
2186	Eritrea	1999	2.37392967945	0.30314234	2.37392967945	0
2187	Eritrea	2000	2.34835038845	0.310248162	2.34758901737727	0.000761371072727446
2188	Eritrea	2001	2.33152990858	0.317247988	2.33089623587242	0.000633672707579525
2189	Eritrea	2002	2.31140415873	0.323566585	2.3092997713475	0.00210438738249996
2190	Eritrea	2003	2.29809989765	0.328593581	2.29679781392619	0.00130208372380958
2191	Eritrea	2004	2.28065258261	0.33287938	2.28054268114694	0.000109901463061224
2192	Eritrea	2005	2.26449706754	0.336682511	2.26352487944112	0.000972188098881777
2193	Eritrea	2006	2.23204329349	0.339835824	2.22957115920389	0.00247213428611115
2194	Eritrea	2007	2.19192435341	0.342843174	2.19192435341	0
2195	Eritrea	2008	2.15147071766	0.343427801	2.15147071766	0
2196	Eritrea	2009	2.11672447651	0.345147743	2.11672447651	0
2197	Eritrea	2010	2.10669359025	0.348146177	2.10669359025	0
2198	Eritrea	2011	2.11936362454	0.354163147	2.11221999476723	0.00714362977276606
2199	Eritrea	2012	2.14337396943	0.360112751	2.11556915190712	0.0278048175228767
2200	Eritrea	2013	2.16976207153	0.364343653	2.11637793952652	0.0533841320034782

TableS2 Frontier analysis of Smoking-Related Low Back Pain among WCBA

	location	year	val	SDI	frontier	eff_diff
2201	Eritrea	2014	2.19482409449	0.372527588	2.11449547383169	0.0803286206583098
2202	Eritrea	2015	2.21570488379	0.376935162	2.11190591113671	0.103798972653286
2203	Eritrea	2016	2.23562301191	0.382171666	2.11351276099263	0.122110250917368
2204	Eritrea	2017	2.25210001305	0.385936029	2.11230784428079	0.139792168769211
2205	Eritrea	2018	2.26407216997	0.391154884	2.11365698781537	0.15041518215463
2206	Eritrea	2019	2.28076733875	0.396330558	2.11291101871473	0.167856320035275
2207	Eritrea	2020	2.2910513147	0.400459411	2.11372383404273	0.177327480657273
2208	Eritrea	2021	2.29456044932	0.403863943	2.11047819562095	0.184082253699054
2209	Estonia	1990	235.29752650922	0.674967632	2.11695641376795	233.180570095452
2210	Estonia	1991	233.73953676491	0.685223089	2.11516952409621	231.624367240814
2211	Estonia	1992	232.22381150131	0.695586674	2.11624239988255	230.107569101427
2212	Estonia	1993	232.8508922008	0.70369315	2.11511527629586	230.735776924504
2213	Estonia	1994	234.31348212678	0.708456181	2.11463015567606	232.198851971104
2214	Estonia	1995	236.0335944645	0.713172725	2.1139050018393	233.919689462661
2215	Estonia	1996	238.23284984822	0.718813024	2.11397907042333	236.118870777797
2216	Estonia	1997	238.73978243072	0.72465244	2.11284628080297	236.626936149917
2217	Estonia	1998	239.41514869404	0.729027035	2.11160224502278	237.303546449017
2218	Estonia	1999	240.53269111948	0.733250616	2.11629072243	238.41640039705
2219	Estonia	2000	241.9060484259	0.739713154	2.11483688569415	239.791211540206
2220	Estonia	2001	245.62972499104	0.746602207	2.11265221570288	243.517072775337
2221	Estonia	2002	251.3359039777	0.752629898	2.11071080309384	249.225193174606
2222	Estonia	2003	256.52711366082	0.757815678	2.11172012986262	254.415393530957
2223	Estonia	2004	260.46477535582	0.763223727	2.11394439348306	258.350830962337
2224	Estonia	2005	261.33887903624	0.769112182	2.11281400837023	259.22606502787
2225	Estonia	2006	260.4128803357	0.774451842	2.11262341832472	258.300256917375

TableS2 Frontier analysis of Smoking-Related Low Back Pain among WCBA

	location	year	val	SDI	frontier	eff_diff
2226	Estonia	2007	259.46527731476	0.781074443	2.11219904435282	257.353078270407
2227	Estonia	2008	258.14666756596	0.788598874	2.11223607680184	256.034431489158
2228	Estonia	2009	256.74854154655	0.794728213	2.11310697061141	254.635434575939
2229	Estonia	2010	255.17655154938	0.800829718	2.11415871211352	253.062392837266
2230	Estonia	2011	252.60519538161	0.806633078	2.11254753874013	250.49264784287
2231	Estonia	2012	247.97747231498	0.811109358	2.11510771658708	245.862364598393
2232	Estonia	2013	243.70487843608	0.814887177	2.11417674707917	241.590701689001
2233	Estonia	2014	239.69161148514	0.818770732	2.112169104586	237.579442380554
2234	Estonia	2015	237.30566222394	0.823221278	2.11212312173041	235.19353910221
2235	Estonia	2016	235.71829229583	0.82736436	2.11485859814985	233.60343369768
2236	Estonia	2017	235.53473782769	0.830975311	2.11225892118517	233.422478906505
2237	Estonia	2018	236.48855512823	0.834566296	2.11438810593896	234.374167022291
2238	Estonia	2019	237.42733289268	0.838573509	2.11295592530746	235.314376967373
2239	Estonia	2020	238.8510748117	0.841817139	2.11271790888814	236.738356902812
2240	Estonia	2021	236.38594369488	0.844917787	2.11327205738268	234.272671637497
2241	Ethiopia	1990	4.66131593419	0.148033885	4.62599718768257	0.0353187465074285
2242	Ethiopia	1991	4.54492888728	0.14959971	4.45515376121206	0.0897751260679369
2243	Ethiopia	1992	4.41958740409	0.150174386	4.41958740409	0
2244	Ethiopia	1993	4.32265590349	0.151582364	4.31016295210507	0.0124929513849272
2245	Ethiopia	1994	4.21934389679	0.152982637	4.21088326606231	0.00846063072769265
2246	Ethiopia	1995	4.08117871186	0.155085605	4.078112337932	0.00306637392800013
2247	Ethiopia	1996	3.97477890584	0.158127229	3.92745973940425	0.0473191664357446
2248	Ethiopia	1997	3.86709172181	0.161874743	3.83246868724709	0.0346230345629079
2249	Ethiopia	1998	3.75828616796	0.16507771	3.72141000361951	0.0368761643404918
2250	Ethiopia	1999	3.67617940896	0.168903545	3.65559683592278	0.0205825730372222

TableS2 Frontier analysis of Smoking-Related Low Back Pain among WCBA

	location	year	val	SDI	frontier	eff_diff
2251	Ethiopia	2000	3.65416725711	0.17341853	3.63413431060727	0.020032946502734
2252	Ethiopia	2001	3.6000473266	0.178972969	3.58107127516443	0.0189760514355712
2253	Ethiopia	2002	3.55353842693	0.184138013	3.52306773813112	0.0304706887988799
2254	Ethiopia	2003	3.5221769318	0.188694814	3.51254779342211	0.00962913837789126
2255	Ethiopia	2004	3.5028851273	0.195030701	3.50282573917123	5.93881287680453e-05
2256	Ethiopia	2005	3.49468956553	0.202868243	3.48714232732596	0.00754723820404424
2257	Ethiopia	2006	3.49860368463	0.211524229	3.48894896386682	0.00965472076317742
2258	Ethiopia	2007	3.51781221178	0.221819931	2.88426808965173	0.633544122128267
2259	Ethiopia	2008	3.54536950506	0.232872186	2.58266478571449	0.962704719345513
2260	Ethiopia	2009	3.57953644654	0.244265818	2.54282651724068	1.03670992929931
2261	Ethiopia	2010	3.61110375428	0.25608146	2.52487206893812	1.08623168534188
2262	Ethiopia	2011	3.65308759007	0.267880862	2.48641931353792	1.16666827653208
2263	Ethiopia	2012	3.69581432563	0.279006958	2.45599622940908	1.23981809622092
2264	Ethiopia	2013	3.74038302166	0.289885267	2.4278114222352	1.3125715994248
2265	Ethiopia	2014	3.78070718173	0.300484548	2.40238534479289	1.37832183693711
2266	Ethiopia	2015	3.82093383394	0.310736419	2.3574328163467	1.4635010175933
2267	Ethiopia	2016	3.87151481761	0.320431867	2.34119468795571	1.53032012965429
2268	Ethiopia	2017	3.92713555312	0.329469244	2.30899123926403	1.61814431385597
2269	Ethiopia	2018	3.98331962088	0.338050075	2.26897546329507	1.71434415758492
2270	Ethiopia	2019	4.028846045	0.346422664	2.13811812801846	1.89072791698154
2271	Ethiopia	2020	4.03152957475	0.35321635	2.11301042117423	1.91851915357577
2272	Ethiopia	2021	4.02457678749	0.358823295	2.12245854249242	1.90211824499758
2273	Fiji	1990	67.27871386312	0.534648908	2.11292713980333	65.1657867233167
2274	Fiji	1991	67.32392480967	0.53877826	2.11281934103322	65.2111054686368
2275	Fiji	1992	67.08316904892	0.543188552	2.11433679301541	64.9688322559046

TableS2 Frontier analysis of Smoking-Related Low Back Pain among WCBA

	location	year	val	SDI	frontier	eff_diff
2276	Fiji	1993	66.91086666496	0.547776474	2.11336845304275	64.7974982119172
2277	Fiji	1994	66.66246607567	0.55284902	2.11184039256569	64.5506256831043
2278	Fiji	1995	66.4393217432	0.558530972	2.11104420011266	64.3282775430873
2279	Fiji	1996	66.2832808856	0.565041879	2.11304274597931	64.1702381396207
2280	Fiji	1997	66.01489484249	0.571187753	2.11192542355152	63.9029694189385
2281	Fiji	1998	65.78534297505	0.577172354	2.1111027661125	63.6742402089375
2282	Fiji	1999	65.35310735039	0.583573942	2.11444181716956	63.2386655332204
2283	Fiji	2000	64.9316736087	0.589196631	2.11124641212861	62.8204271965714
2284	Fiji	2001	64.74599478076	0.594497419	2.11264302016727	62.6333517605927
2285	Fiji	2002	64.51322780202	0.599322031	2.11307942080284	62.4001483812172
2286	Fiji	2003	64.29256376028	0.603489622	2.11282461892743	62.1797391413526
2287	Fiji	2004	64.23698982731	0.60754489	2.11616073843692	62.1208290888731
2288	Fiji	2005	64.18465684898	0.610812944	2.11529269057788	62.0693641584021
2289	Fiji	2006	64.42115994514	0.613932767	2.11320767802167	62.3079522671183
2290	Fiji	2007	64.93747689111	0.616582915	2.11162660219342	62.8258502889166
2291	Fiji	2008	65.37720538568	0.618958809	2.11396774119783	63.2632376444822
2292	Fiji	2009	66.02615053138	0.620883465	2.11086848765883	63.9152820437212
2293	Fiji	2010	66.56159272509	0.623308786	2.11358451119431	64.4480082138957
2294	Fiji	2011	67.24774592581	0.626438251	2.11325982175761	65.1344861040524
2295	Fiji	2012	67.97667209882	0.629881335	2.11346400338433	65.8632080954357
2296	Fiji	2013	68.50646165201	0.634278114	2.1114410663012	66.3950205857088
2297	Fiji	2014	69.20061481154	0.639390066	2.11145714355873	67.0891576679813
2298	Fiji	2015	69.7326235682	0.645026353	2.11425955937818	67.6183640088218
2299	Fiji	2016	70.20468444695	0.65074268	2.11156811957312	68.0931163273769
2300	Fiji	2017	70.62187357809	0.65646401	2.11525164336226	68.5066219347277

TableS2 Frontier analysis of Smoking-Related Low Back Pain among WCBA

	location	year	val	SDI	frontier	eff_diff
2301	Fiji	2018	70.97734206961	0.661991454	2.11246931178029	68.8648727578297
2302	Fiji	2019	71.21803650675	0.667060979	2.11378639169696	69.104250115053
2303	Fiji	2020	70.80966193633	0.671431491	2.11053893626705	68.699123000063
2304	Fiji	2021	71.11123945325	0.675051631	2.1136883291502	68.9975511240998
2305	Finland	1990	231.73569772268	0.756221509	2.11053721222852	229.625160510451
2306	Finland	1991	229.78768813321	0.758410687	2.11716346989638	227.670524663314
2307	Finland	1992	228.73328075255	0.7608041	2.11409436692507	226.619186385625
2308	Finland	1993	226.91044688875	0.763526604	2.11274748160522	224.797699407145
2309	Finland	1994	225.47480630283	0.767101901	2.11426381579074	223.360542487039
2310	Finland	1995	223.13280962549	0.771163742	2.11226604803878	221.020543577451
2311	Finland	1996	219.99710329931	0.774809059	2.11463994343914	217.882463355871
2312	Finland	1997	216.03662463868	0.778814215	2.1104004574591	213.926224181221
2313	Finland	1998	211.20488945044	0.782305059	2.11374669433172	209.091142756108
2314	Finland	1999	206.26558430544	0.785773988	2.11515264109653	204.150431664344
2315	Finland	2000	202.63794149159	0.78998523	2.11465431832443	200.523287173266
2316	Finland	2001	199.30219357575	0.794453835	2.11576250176817	197.186431073982
2317	Finland	2002	195.65447639801	0.798770274	2.11333780165442	193.541138596356
2318	Finland	2003	191.91102258012	0.802325431	2.11484381001494	189.796178770105
2319	Finland	2004	188.82972319962	0.805694227	2.11445087869468	186.715272320925
2320	Finland	2005	186.60234491233	0.808936787	2.11411751008479	184.488227402245
2321	Finland	2006	184.38158390538	0.812081482	2.11251274352987	182.26907116185
2322	Finland	2007	182.49023143271	0.815597613	2.11155344034691	180.378677992363
2323	Finland	2008	180.93468839936	0.818875544	2.11422726200231	178.820461137358
2324	Finland	2009	178.94850749545	0.821457588	2.11947122486538	176.829036270585
2325	Finland	2010	177.27308339897	0.824844982	2.11380297696648	175.159280422004

TableS2 Frontier analysis of Smoking-Related Low Back Pain among WCBA

	location	year	val	SDI	frontier	eff_diff
2326	Finland	2011	175.09410558828	0.828270577	2.11516852633667	172.978937061943
2327	Finland	2012	172.98505431643	0.831434484	2.11294528475769	170.872109031672
2328	Finland	2013	170.59174920381	0.834425401	2.1124825986641	168.479266605146
2329	Finland	2014	168.55885028062	0.837431872	2.11178115947058	166.447069121149
2330	Finland	2015	166.5829282079	0.840878477	2.11574249444651	164.467185713453
2331	Finland	2016	165.24298999443	0.844622338	2.11681203335121	163.126177961079
2332	Finland	2017	163.89216187627	0.848586128	2.11128645358695	161.780875422683
2333	Finland	2018	163.03475383241	0.852202943	2.11500886622506	160.919744966185
2334	Finland	2019	163.12915279333	0.855365179	2.111460632852	161.017692160478
2335	Finland	2020	161.47827959353	0.857655553	2.11418167723433	159.364097916296
2336	Finland	2021	162.0040516773	0.859831368	2.11355707107274	159.890494606227
2337	France	1990	232.08858688085	0.730747466	2.11217105523419	229.976415825616
2338	France	1991	235.60157766739	0.736452382	2.11211223542783	233.489465431962
2339	France	1992	238.72075380564	0.742871973	2.11523265505744	236.605521150583
2340	France	1993	242.22245163811	0.748582327	2.11187437147463	240.110577266635
2341	France	1994	245.18700148535	0.753176132	2.11376725108	243.07323423427
2342	France	1995	248.16999104921	0.757125789	2.11200022146205	246.057990827748
2343	France	1996	250.97719411388	0.760899579	2.1147799301431	248.862414183737
2344	France	1997	253.96259448355	0.764650672	2.1123827010012	251.850211782549
2345	France	1998	256.60826346624	0.767852561	2.11678078347127	254.491482682769
2346	France	1999	259.13723242394	0.770304363	2.11668248610492	257.020549937835
2347	France	2000	261.51307826677	0.772792033	2.11443504967984	259.39864321709
2348	France	2001	263.53200479264	0.776225157	2.11516322839873	261.416841564241
2349	France	2002	265.92785523544	0.780088872	2.11451745938235	263.813337776058
2350	France	2003	267.62981757523	0.783178757	2.1135867813267	265.516230793903

TableS2 Frontier analysis of Smoking-Related Low Back Pain among WCBA

	location	year	val	SDI	frontier	eff_diff
2351	France	2004	268.89264688405	0.786084655	2.1124045725722	266.780242311478
2352	France	2005	270.23493819115	0.789042086	2.11442475758554	268.120513433564
2353	France	2006	270.2004145108	0.79232192	2.11155148754696	268.088863023253
2354	France	2007	270.88982301583	0.795557651	2.1137131367118	268.776109879118
2355	France	2008	270.50422965306	0.79817743	2.11288057216323	268.391349080897
2356	France	2009	270.22866522899	0.80050867	2.10993431885463	268.118730910135
2357	France	2010	270.1226013282	0.803240247	2.11357075424859	268.009030573951
2358	France	2011	270.14540288933	0.806378738	2.11388576326373	268.031517126066
2359	France	2012	269.85337486115	0.809615389	2.11467371576389	267.738701145386
2360	France	2013	269.13427759251	0.812984015	2.11456105776726	267.019716534743
2361	France	2014	267.89448141444	0.816551448	2.11265963190068	265.781821782539
2362	France	2015	267.45425640573	0.820238385	2.11247451875747	265.341781886973
2363	France	2016	266.7790075716	0.823730929	2.11307545503575	264.665932116564
2364	France	2017	267.109096939	0.827098441	2.11032785760187	264.998769081398
2365	France	2018	267.03309593059	0.83044859	2.11461914678701	264.918476783803
2366	France	2019	265.56200519572	0.833772805	2.11270999044838	263.449295205272
2367	France	2020	259.0834322409	0.836049443	2.11353141444623	256.969900826454
2368	France	2021	257.35065365929	0.838364875	2.11258077182793	255.238072887462
2369	Gabon	1990	9.43102717662	0.455421187	2.11384299434053	7.31718418227947
2370	Gabon	1991	9.46673536968	0.461563098	2.11413963780093	7.35259573187907
2371	Gabon	1992	9.53605562192	0.467024048	2.1148278765388	7.4212277453812
2372	Gabon	1993	9.61694566541	0.472394463	2.11074674832974	7.50619891708026
2373	Gabon	1994	9.71866153316	0.478061947	2.11412375390644	7.60453777925356
2374	Gabon	1995	9.80825119122	0.483826218	2.11546257962629	7.69278861159371
2375	Gabon	1996	9.97181874793	0.48946146	2.11014805294908	7.86167069498092

TableS2 Frontier analysis of Smoking-Related Low Back Pain among WCBA

	location	year	val	SDI	frontier	eff_diff
2376	Gabon	1997	10.17734447203	0.495074162	2.11328940049452	8.06405507153548
2377	Gabon	1998	10.39488334822	0.50037255	2.11450150947442	8.28038183874558
2378	Gabon	1999	10.6948375094	0.50480635	2.11523098590229	8.57960652349772
2379	Gabon	2000	10.95699130829	0.509133087	2.11435332151667	8.84263798677333
2380	Gabon	2001	10.99992799983	0.513045709	2.11295201556141	8.8869759842686
2381	Gabon	2002	11.07858466786	0.516917621	2.11385026724586	8.96473440061414
2382	Gabon	2003	11.1685091563	0.521051412	2.11782957510539	9.05067958119461
2383	Gabon	2004	11.23308434445	0.525479071	2.11096970340815	9.12211464104185
2384	Gabon	2005	11.30648110238	0.530488575	2.11238207610882	9.19409902627118
2385	Gabon	2006	11.36517655006	0.534797716	2.11136995716791	9.25380659289209
2386	Gabon	2007	11.39997527073	0.539441424	2.11437647935652	9.28559879137348
2387	Gabon	2008	11.41926006691	0.544110892	2.11364370381851	9.30561636309148
2388	Gabon	2009	11.45277273354	0.548591319	2.11245387676446	9.34031885677554
2389	Gabon	2010	11.47425040919	0.554022706	2.11152617147821	9.36272423771178
2390	Gabon	2011	11.49729674006	0.560232082	2.11313998707655	9.38415675298345
2391	Gabon	2012	11.52234888601	0.566842964	2.11183347824522	9.41051540776478
2392	Gabon	2013	11.52810331259	0.57380813	2.11137871045847	9.41672460213153
2393	Gabon	2014	11.5569036831	0.580915656	2.11295468005787	9.44394900304213
2394	Gabon	2015	11.59390359485	0.588365	2.11477624632577	9.47912734852423
2395	Gabon	2016	11.63863774902	0.596062305	2.11381538162036	9.52482236739964
2396	Gabon	2017	11.73654028682	0.603816252	2.11513167612812	9.62140861069187
2397	Gabon	2018	11.78278771864	0.61172438	2.11208670965149	9.67070100898851
2398	Gabon	2019	11.85671980313	0.619904727	2.11163231229159	9.74508749083841
2399	Gabon	2020	11.81955160298	0.627609647	2.115784344415	9.703767258565
2400	Gabon	2021	11.85221801666	0.634691393	2.11398276110418	9.73823525555582

TableS2 Frontier analysis of Smoking-Related Low Back Pain among WCBA

	location	year	val	SDI	frontier	eff_diff
2401	Gambia	1990	4.49363764282	0.238714846	2.67378125534693	1.81985638747307
2402	Gambia	1991	4.43659097958	0.245010164	2.56504262247972	1.87154835710028
2403	Gambia	1992	4.40049074367	0.251087863	2.50232895881494	1.89816178485506
2404	Gambia	1993	4.37194906498	0.256996141	2.51085222473282	1.86109684024718
2405	Gambia	1994	4.35636415835	0.262424792	2.50312291809933	1.85324124025067
2406	Gambia	1995	4.35903771871	0.267696507	2.48545131682955	1.87358640188045
2407	Gambia	1996	4.33821127536	0.27295019	2.46333329047242	1.87487798488758
2408	Gambia	1997	4.32175099135	0.278274122	2.45767818268771	1.86407280866229
2409	Gambia	1998	4.29705353338	0.283946056	2.43798256099914	1.85907097238086
2410	Gambia	1999	4.25992570225	0.290070621	2.43061886366175	1.82930683858825
2411	Gambia	2000	4.21505987045	0.296354282	2.39822063939826	1.81683923105174
2412	Gambia	2001	4.19165689476	0.302543257	2.40346767626164	1.78818921849836
2413	Gambia	2002	4.15993663677	0.307712927	2.38291222198291	1.77702441478709
2414	Gambia	2003	4.11660662667	0.312930916	2.36931240673447	1.74729421993553
2415	Gambia	2004	4.05861920235	0.318615029	2.34094424210643	1.71767496024357
2416	Gambia	2005	4.00568230538	0.32356835	2.31800252112138	1.68767978425862
2417	Gambia	2006	3.92388870747	0.328151431	2.31634103059313	1.60754767687687
2418	Gambia	2007	3.84188630819	0.332640147	2.3087352177905	1.5331510903995
2419	Gambia	2008	3.74941375111	0.337315245	2.27140995110119	1.47800380000881
2420	Gambia	2009	3.65779163574	0.342389614	2.24899834465684	1.40879329108316
2421	Gambia	2010	3.57565535353	0.34799783	2.14724770665616	1.42840764687384
2422	Gambia	2011	3.48672488696	0.352869236	2.12084192817385	1.36588295878615
2423	Gambia	2012	3.39102444333	0.35837365	2.11595290197632	1.27507154135368
2424	Gambia	2013	3.30200745022	0.364255878	2.11913239726258	1.18287505295742
2425	Gambia	2014	3.21374079047	0.3699013	2.11509261001418	1.09864818045582

TableS2 Frontier analysis of Smoking-Related Low Back Pain among WCBA

	location	year	val	SDI	frontier	eff_diff
2426	Gambia	2015	3.16921315134	0.375840485	2.11851035523279	1.05070279610721
2427	Gambia	2016	3.14487941337	0.381569217	2.11358510621935	1.03129430715064
2428	Gambia	2017	3.12127968381	0.387273644	2.11480649748122	1.00647318632878
2429	Gambia	2018	3.10755964576	0.393155679	2.11351249079436	0.994047154965637
2430	Gambia	2019	3.09953600401	0.39918978	2.11457291477037	0.984963089239625
2431	Gambia	2020	3.07992373003	0.404524194	2.11647736584784	0.963446364182162
2432	Gambia	2021	3.06987489781	0.40971416	2.112425875635	0.957449022175
2433	Georgia	1990	57.14330509443	0.656136044	2.1154428601688	55.0278622342612
2434	Georgia	1991	56.14704713754	0.662113186	2.11059779541938	54.0364493421206
2435	Georgia	1992	55.74867554126	0.665021428	2.1120804626871	53.6365950785729
2436	Georgia	1993	56.11901358431	0.663466862	2.11489182898758	54.0041217553224
2437	Georgia	1994	57.02895858491	0.658134139	2.11396323275184	54.9149953521582
2438	Georgia	1995	58.05894195156	0.651363604	2.1148877716862	55.9440541798738
2439	Georgia	1996	59.18449362656	0.64455809	2.11461001350284	57.0698836130572
2440	Georgia	1997	60.22400373808	0.638525631	2.11043283876889	58.1135708993111
2441	Georgia	1998	61.19281996406	0.633347931	2.11562787859955	59.0771920854605
2442	Georgia	1999	62.26702747671	0.630277946	2.11518442291394	60.1518430537961
2443	Georgia	2000	62.96804563535	0.630265991	2.11584721345711	60.8521984218929
2444	Georgia	2001	63.79679602083	0.6333883	2.11429875868149	61.6824972621485
2445	Georgia	2002	64.71917176821	0.638370108	2.11216450660983	62.6070072616002
2446	Georgia	2003	65.77810383148	0.644065579	2.11394991208132	63.6641539193987
2447	Georgia	2004	66.85591670898	0.648920978	2.1139191588009	64.7419975501791
2448	Georgia	2005	67.7096334508	0.653616005	2.11488862994303	65.594744820857
2449	Georgia	2006	68.68776149865	0.658403372	2.11803789413314	66.5697236045169
2450	Georgia	2007	69.79295143492	0.663697966	2.11593294798443	67.6770184869356

TableS2 Frontier analysis of Smoking-Related Low Back Pain among WCBA

	location	year	val	SDI	frontier	eff_diff
2451	Georgia	2008	71.12086133189	0.668442496	2.11331518454267	69.0075461473473
2452	Georgia	2009	72.29538182807	0.672449381	2.1131046029811	70.1822772250889
2453	Georgia	2010	72.96853866968	0.677034892	2.11354711297338	70.8549915567066
2454	Georgia	2011	73.51726075474	0.681567044	2.11653269827583	71.4007280564642
2455	Georgia	2012	73.83997684703	0.685519223	2.11434519381105	71.725631653219
2456	Georgia	2013	74.25711882664	0.688603268	2.1120274202618	72.1450914063782
2457	Georgia	2014	74.71181605334	0.692155207	2.11217885564739	72.5996371976926
2458	Georgia	2015	75.58100913891	0.697316133	2.11306502702141	73.4679441118886
2459	Georgia	2016	76.86096484272	0.70373149	2.11437098775	74.74659385497
2460	Georgia	2017	78.53221748783	0.710874718	2.11676067672846	76.4154568111015
2461	Georgia	2018	80.35006535159	0.717993654	2.11382412617385	78.2362412254162
2462	Georgia	2019	81.72029621287	0.724536462	2.11431272447232	79.6059834883977
2463	Georgia	2020	82.55259188555	0.729125889	2.11494881664851	80.4376430689015
2464	Georgia	2021	83.22832775184	0.732473604	2.11223005387847	81.1160976979615
2465	Germany	1990	311.61844686016	0.817077666	2.11084006710371	309.507606793056
2466	Germany	1991	313.07415049089	0.823339898	2.11340575695615	310.960744733934
2467	Germany	1992	316.88921766893	0.828323503	2.11257549805229	314.776642170878
2468	Germany	1993	320.17450530145	0.832286809	2.11570098762106	318.058804313829
2469	Germany	1994	323.77588295779	0.835561963	2.11094766907137	321.664935288719
2470	Germany	1995	328.55156221788	0.837643152	2.11213083256587	326.439431385314
2471	Germany	1996	333.97392710633	0.839188129	2.11074921897667	331.863177887353
2472	Germany	1997	337.71361963328	0.841385903	2.113187590562	335.600432042718
2473	Germany	1998	341.06571661397	0.843854978	2.11262028197836	338.953096331992
2474	Germany	1999	343.27531253872	0.846232398	2.11338078428167	341.161931754438
2475	Germany	2000	343.54848646398	0.848925756	2.11110642884059	341.437380035139

TableS2 Frontier analysis of Smoking-Related Low Back Pain among WCBA

	location	year	val	SDI	frontier	eff_diff
2476	Germany	2001	342.78040253717	0.852070413	2.11823006046779	340.662172476702
2477	Germany	2002	339.04940290164	0.855066793	2.11303351059864	336.936369391041
2478	Germany	2003	334.50203670872	0.85767579	2.11056953492356	332.391467173796
2479	Germany	2004	330.44706719765	0.860240333	2.11161403876691	328.335453158883
2480	Germany	2005	326.10215327945	0.862894178	2.11290547441473	323.989247805035
2481	Germany	2006	323.22687481286	0.865873707	2.11167245490066	321.115202357959
2482	Germany	2007	320.47832842064	0.86914044	2.112586515415	318.365741905225
2483	Germany	2008	317.65587680031	0.872552849	2.11370734789574	315.542169452414
2484	Germany	2009	314.69251476418	0.875087911	2.11264209511632	312.579872669064
2485	Germany	2010	311.76281103278	0.87794671	2.1133452662783	309.649465766502
2486	Germany	2011	307.9588356898	0.881027508	2.11271472743819	305.846120962362
2487	Germany	2012	302.21915599196	0.883656408	2.11474050641973	300.10441548554
2488	Germany	2013	295.60385837536	0.885926953	2.11950692478257	293.484351450577
2489	Germany	2014	289.25887326123	0.887651614	2.11359188171424	287.145281379516
2490	Germany	2015	283.51213973386	0.888867854	2.11027664034098	281.401863093519
2491	Germany	2016	278.9891046293	0.890896369	2.1127140279415	276.876390601359
2492	Germany	2017	275.10235954027	0.894050472	2.11266505372314	272.989694486547
2493	Germany	2018	272.44385053115	0.896975545	2.11179646677771	270.332054064372
2494	Germany	2019	270.58773221657	0.899703158	2.11464806734595	268.473084149224
2495	Germany	2020	262.34794199639	0.901438614	2.11511872747783	260.232823268912
2496	Germany	2021	263.01221472328	0.902957091	2.11153592739474	260.900678795885
2497	Ghana	1990	8.78352908068	0.373112005	2.11390929623785	6.66961978444215
2498	Ghana	1991	8.52598099959	0.379239256	2.11474559274112	6.41123540684888
2499	Ghana	1992	8.31796801682	0.384856767	2.11398638647782	6.20398163034218
2500	Ghana	1993	8.15848615903	0.389968494	2.11141627648708	6.04706988254292

TableS2 Frontier analysis of Smoking-Related Low Back Pain among WCBA

	location	year	val	SDI	frontier	eff_diff
2501	Ghana	1994	8.03774582579	0.394953663	2.11903933791922	5.91870648787078
2502	Ghana	1995	7.96886280507	0.399930204	2.11341034511125	5.85545245995875
2503	Ghana	1996	7.95975556983	0.405003047	2.1151008047775	5.8446547650525
2504	Ghana	1997	7.99243245062	0.410038073	2.10971957054606	5.88271288007394
2505	Ghana	1998	8.02450031331	0.414888532	2.11507628815231	5.90942402515769
2506	Ghana	1999	8.07243906951	0.419755496	2.11528343880313	5.95715563070687
2507	Ghana	2000	8.14396079356	0.424725212	2.11371217254651	6.03024862101349
2508	Ghana	2001	8.28868337534	0.429411846	2.11046566716817	6.17821770817183
2509	Ghana	2002	8.49472372905	0.434104128	2.11427891610493	6.38044481294507
2510	Ghana	2003	8.70511925242	0.439118213	2.11282704052683	6.59229221189317
2511	Ghana	2004	8.90111674379	0.444236977	2.11162923588373	6.78948750790627
2512	Ghana	2005	9.11061849689	0.44936002	2.11353768551712	6.99708081137288
2513	Ghana	2006	9.24999374328	0.454692737	2.11393326942516	7.13606047385484
2514	Ghana	2007	9.49020798427	0.460606245	2.11241439390988	7.37779359036012
2515	Ghana	2008	9.66626267862	0.467259067	2.11178000449973	7.55448267412027
2516	Ghana	2009	9.86205536496	0.473848047	2.1174943059408	7.7445610590192
2517	Ghana	2010	9.97525824225	0.480777892	2.113077099886	7.862181142364
2518	Ghana	2011	10.00422821117	0.489020974	2.11472597500176	7.88950223616824
2519	Ghana	2012	10.02416983453	0.497513932	2.11324720144119	7.91092263308881
2520	Ghana	2013	10.04451544687	0.506004647	2.11704672358453	7.92746872328547
2521	Ghana	2014	10.03352114041	0.514119061	2.1177618698618	7.9157592705482
2522	Ghana	2015	10.04280400183	0.521539202	2.11421249271279	7.92859150911721
2523	Ghana	2016	10.0510635056	0.528629586	2.11191737080811	7.93914613479189
2524	Ghana	2017	10.10340169773	0.53616994	2.11536346585508	7.98803823187492
2525	Ghana	2018	10.16776359078	0.543840539	2.11373683061039	8.05402676016961

TableS2 Frontier analysis of Smoking-Related Low Back Pain among WCBA

	location	year	val	SDI	frontier	eff_diff
2526	Ghana	2019	10.16493899518	0.551632027	2.11175470688246	8.05318428829754
2527	Ghana	2020	10.12405436437	0.558462173	2.11440426024632	8.00965010412368
2528	Ghana	2021	10.0781583161	0.56493039	2.11373999607506	7.96441832002494
2529	Global	1990	84.57556680492	0.525528856	2.11160952443986	82.4639572804801
2530	Global	1991	83.51646415287	0.530272485	2.11406187485444	81.4024022780156
2531	Global	1992	82.65183927857	0.534636544	2.11504479216491	80.5367944864051
2532	Global	1993	81.89384218087	0.538598319	2.11346245828859	79.7803797225814
2533	Global	1994	81.32915006858	0.542389433	2.11157716355461	79.2175729050254
2534	Global	1995	81.01044785618	0.546327999	2.11173240184506	78.8987154543349
2535	Global	1996	80.67273406907	0.550703413	2.11165508690476	78.5610789821652
2536	Global	1997	79.81182737551	0.555139426	2.11775183174453	77.6940755437655
2537	Global	1998	78.85762756035	0.559380567	2.11971468127383	76.7379128790762
2538	Global	1999	77.90970924999	0.563738017	2.11394329949072	75.7957659504993
2539	Global	2000	76.97937938193	0.568003164	2.11084058051045	74.8685388014196
2540	Global	2001	76.1434507231	0.571777531	2.1140151816736	74.0294355414264
2541	Global	2002	75.22663474469	0.57568796	2.11244588579613	73.1141888588939
2542	Global	2003	74.24071459216	0.579907077	2.11119873571647	72.1295158564435
2543	Global	2004	73.21365666868	0.584378588	2.11393603248514	71.0997206361949
2544	Global	2005	72.12407722524	0.58897852	2.11208131179489	70.0119959134451
2545	Global	2006	70.94068338223	0.593756623	2.11133257620915	68.8293508060209
2546	Global	2007	69.66503510059	0.598641368	2.112913658368	67.552121442222
2547	Global	2008	68.31160867901	0.603370099	2.11420621782507	66.1974024611849
2548	Global	2009	66.98160445232	0.60775944	2.11193861105202	64.869665841268
2549	Global	2010	65.75603126427	0.612952497	2.11582649391015	63.6402047703598
2550	Global	2011	64.56153912342	0.617759223	2.11525963309877	62.4462794903212

TableS2 Frontier analysis of Smoking-Related Low Back Pain among WCBA

	location	year	val	SDI	frontier	eff_diff
2551	Global	2012	63.32504623438	0.621844977	2.11631135943363	61.2087348749464
2552	Global	2013	62.13755877874	0.626343279	2.11070197296663	60.0268568057734
2553	Global	2014	61.07985234546	0.630982367	2.11255339706397	58.967298948396
2554	Global	2015	60.24734394673	0.635441488	2.11487872041227	58.1324652263177
2555	Global	2016	59.63733294938	0.640153586	2.11351942301778	57.5238135263622
2556	Global	2017	59.054874021	0.645564304	2.11372707006177	56.9411469509382
2557	Global	2018	58.48954298366	0.651048474	2.11185679965712	56.3776861840029
2558	Global	2019	57.96300845267	0.656575898	2.11327959356593	55.8497288591041
2559	Global	2020	56.96198370931	0.661343543	2.11509256812958	54.8468911411804
2560	Global	2021	56.54082767157	0.665820975	2.11288358566958	54.4279440859004
2561	Greece	1990	239.73253262051	0.674186465	2.11509234584911	237.617440274661
2562	Greece	1991	242.43710771339	0.68031648	2.11067466300226	240.326433050388
2563	Greece	1992	245.4466934043	0.687055389	2.1133567019576	243.333336702342
2564	Greece	1993	249.91110421958	0.693328926	2.11212746219914	247.798976757381
2565	Greece	1994	253.67193557635	0.699153022	2.11828679976175	251.553648776588
2566	Greece	1995	258.12235166354	0.704862873	2.11396949144185	256.008382172098
2567	Greece	1996	262.49142659767	0.710139336	2.11308101823418	260.378345579436
2568	Greece	1997	267.38942560918	0.715687268	2.11280112115291	265.276624488027
2569	Greece	1998	272.19993752319	0.721593982	2.1138860738042	270.086051449386
2570	Greece	1999	278.14316324182	0.727529455	2.11531885983472	276.027844381985
2571	Greece	2000	284.02874170951	0.73271054	2.11187541062538	281.916866298885
2572	Greece	2001	289.89560483707	0.737154008	2.11443836230213	287.781166474768
2573	Greece	2002	296.13324977574	0.741945221	2.11514711696429	294.018102658776
2574	Greece	2003	302.5120788697	0.747185956	2.11531471834159	300.396764151358
2575	Greece	2004	308.17455617777	0.752418607	2.11492604154845	306.059630136222

TableS2 Frontier analysis of Smoking-Related Low Back Pain among WCBA

	location	year	val	SDI	frontier	eff_diff
2576	Greece	2005	312.00984573803	0.756384396	2.11836568002187	309.891480058008
2577	Greece	2006	314.20126399212	0.760209696	2.111357715122	312.089906276998
2578	Greece	2007	315.4316572851	0.763927173	2.11369654219319	313.317960742907
2579	Greece	2008	315.90597076662	0.767527658	2.11561226122698	313.790358505393
2580	Greece	2009	316.7930865423	0.771247356	2.11191751578329	314.681169026517
2581	Greece	2010	317.36129872435	0.775155815	2.11562965240159	315.245669071948
2582	Greece	2011	317.35386406535	0.778525867	2.11411874772614	315.239745317624
2583	Greece	2012	316.50639447791	0.780789483	2.11295011505551	314.393444362854
2584	Greece	2013	314.84999265517	0.781949052	2.116544385146	312.733448270024
2585	Greece	2014	312.80094263728	0.782442013	2.11349440965592	310.687448227624
2586	Greece	2015	311.48721800098	0.782489523	2.11058977380809	309.376628227172
2587	Greece	2016	311.0344536288	0.782798443	2.11261788827338	308.921835740527
2588	Greece	2017	311.20284520347	0.784108594	2.11507683532397	309.087768368146
2589	Greece	2018	311.04070895282	0.785999753	2.113693564036	308.927015388784
2590	Greece	2019	309.45460324768	0.788138138	2.11527692592568	307.339326321754
2591	Greece	2020	303.55242451899	0.78963222	2.11473521891261	301.437689300077
2592	Greece	2021	300.34795381744	0.791854408	2.11071292969421	298.237240887746
2593	Greenland	1990	292.33501844968	0.732258252	2.11726438932774	290.217754060352
2594	Greenland	1991	302.41446358918	0.730626532	2.11546744398463	300.298996145195
2595	Greenland	1992	315.36736097297	0.729781103	2.11329181274429	313.254069160226
2596	Greenland	1993	323.19789850001	0.729894842	2.11385000216741	321.084048497843
2597	Greenland	1994	333.56290441519	0.730875896	2.11410328996887	331.448801125221
2598	Greenland	1995	345.24257744731	0.730831638	2.11442199859788	343.128155448712
2599	Greenland	1996	356.12270946776	0.730216225	2.11303568919343	354.009673778567
2600	Greenland	1997	365.87559410644	0.730551087	2.11114653241158	363.764447574028

TableS2 Frontier analysis of Smoking-Related Low Back Pain among WCBA

	location	year	val	SDI	frontier	eff_diff
2601	Greenland	1998	371.72656510978	0.733242588	2.11448951661618	369.612075593164
2602	Greenland	1999	381.04789744802	0.73655713	2.112446052194	378.935451395826
2603	Greenland	2000	388.2291813053	0.74085701	2.11266411610534	386.116517189195
2604	Greenland	2001	388.01226714821	0.746289605	2.11516187731514	385.897105270895
2605	Greenland	2002	388.60087128959	0.752878371	2.11547265388485	386.485398635705
2606	Greenland	2003	387.887222642	0.759444152	2.11487889085125	385.772343751149
2607	Greenland	2004	383.25709614275	0.765401567	2.1109213397963	381.146174802954
2608	Greenland	2005	378.31261032402	0.77209958	2.11676516402797	376.195845159992
2609	Greenland	2006	372.50757484528	0.779141406	2.1123038913741	370.395270953906
2610	Greenland	2007	366.03632997073	0.785321922	2.11125014216425	363.925079828566
2611	Greenland	2008	359.40822208832	0.791441257	2.11540120366291	357.292820884657
2612	Greenland	2009	352.61921546986	0.79702575	2.11293782680442	350.506277643056
2613	Greenland	2010	344.47902292887	0.803003131	2.11211353519476	342.366909393675
2614	Greenland	2011	337.19810944319	0.808105185	2.11284847701673	335.085260966173
2615	Greenland	2012	330.63702590575	0.811684562	2.11203638079689	328.524989524953
2616	Greenland	2013	326.21453748706	0.814368308	2.11295583886268	324.101581648197
2617	Greenland	2014	322.14259980412	0.81652706	2.11268367128117	320.029916132839
2618	Greenland	2015	315.70692646194	0.817719189	2.11217846043029	313.59474800151
2619	Greenland	2016	314.57721706943	0.818961902	2.11414575170781	312.463071317722
2620	Greenland	2017	310.94065835532	0.819781101	2.11453730480225	308.826121050518
2621	Greenland	2018	309.94661041555	0.820815514	2.11170826619817	307.834902149352
2622	Greenland	2019	311.01906321478	0.822339996	2.1100457423784	308.909017472402
2623	Greenland	2020	300.80511987416	0.824359028	2.11419439666528	298.690925477495
2624	Greenland	2021	295.40233463499	0.826210336	2.11176035634724	293.290574278643
2625	Grenada	1990	32.38219866698	0.436734419	2.11458927660647	30.2676093903735

TableS2 Frontier analysis of Smoking-Related Low Back Pain among WCBA

	location	year	val	SDI	frontier	eff_diff
2626	Grenada	1991	33.09768066871	0.445887953	2.11495330284739	30.9827273658626
2627	Grenada	1992	33.66963598515	0.455910058	2.11434050220468	31.5552954829453
2628	Grenada	1993	34.0402955836	0.46599489	2.11352896413833	31.9267666194617
2629	Grenada	1994	34.24622564268	0.476410341	2.1108964553091	32.1353291873709
2630	Grenada	1995	34.27917929586	0.486558999	2.11564160666284	32.1635376891972
2631	Grenada	1996	34.19739541121	0.496589808	2.11295630990559	32.0844391013044
2632	Grenada	1997	33.99888709088	0.506217602	2.11401359255354	31.8848734983265
2633	Grenada	1998	33.68931764555	0.516590019	2.11598384272038	31.5733338028296
2634	Grenada	1999	33.48516615195	0.527333411	2.11207451694521	31.3730916350048
2635	Grenada	2000	33.37794019137	0.538388116	2.113868483361	31.264071708009
2636	Grenada	2001	33.13284789982	0.54905897	2.11370854328523	31.0191393565348
2637	Grenada	2002	32.96938566933	0.559473128	2.11292286733899	30.856462801991
2638	Grenada	2003	32.81769729026	0.569718037	2.11104409253833	30.7066531977217
2639	Grenada	2004	32.75641871629	0.578487456	2.11481672834877	30.6416019879412
2640	Grenada	2005	32.78783130276	0.587158877	2.11195178770594	30.6758795150541
2641	Grenada	2006	32.79198720749	0.59405452	2.11448631900562	30.6775008884844
2642	Grenada	2007	32.74921513212	0.600766336	2.11364652706464	30.6355686050554
2643	Grenada	2008	32.6933766847	0.606879282	2.11315634195694	30.5802203427431
2644	Grenada	2009	32.63994888043	0.611805257	2.1140918886196	30.5258569918104
2645	Grenada	2010	32.62815426377	0.616486997	2.11370640451838	30.5144478592516
2646	Grenada	2011	32.60310762891	0.620978521	2.11305243329405	30.490055195616
2647	Grenada	2012	32.61117457614	0.624977512	2.11381446063419	30.4973601155058
2648	Grenada	2013	32.66725391921	0.629048336	2.1107144787146	30.5565394404954
2649	Grenada	2014	32.66032917578	0.633734696	2.11104936959918	30.5492798061808
2650	Grenada	2015	32.72021860443	0.638936084	2.11277195144706	30.6074466529829

TableS2 Frontier analysis of Smoking-Related Low Back Pain among WCBA

	location	year	val	SDI	frontier	eff_diff
2651	Grenada	2016	32.80205556537	0.644335234	2.11283262778635	30.6892229375837
2652	Grenada	2017	32.76080609541	0.649831522	2.11298065756825	30.6478254378417
2653	Grenada	2018	32.82851062043	0.655372772	2.11411380291119	30.7143968175188
2654	Grenada	2019	32.85339513157	0.660808512	2.11576007246446	30.7376350591055
2655	Grenada	2020	32.85771855095	0.665086347	2.11259863203309	30.7451199189169
2656	Grenada	2021	32.84812334592	0.668993028	2.11333018026582	30.7347931656542
2657	Guam	1990	88.10835435113	0.676220305	2.11227444227592	85.9960799088541
2658	Guam	1991	89.78840406859	0.669974292	2.11232954969507	87.6760745188949
2659	Guam	1992	91.81988593601	0.66635634	2.11379766688112	89.7060882691289
2660	Guam	1993	93.93162723892	0.667318181	2.11537637217806	91.8162508667419
2661	Guam	1994	96.06986330818	0.672111712	2.1153568107225	93.9545064974575
2662	Guam	1995	98.00909309679	0.678120215	2.11260343300904	95.896489663781
2663	Guam	1996	99.70757533398	0.684173735	2.11171902221	97.59585631177
2664	Guam	1997	101.24203773942	0.691684871	2.11138487857845	99.1306528608416
2665	Guam	1998	102.53991457136	0.701819289	2.11398176939705	100.425932801963
2666	Guam	1999	103.68131465956	0.713641593	2.11138000518043	101.56993465438
2667	Guam	2000	104.89379586033	0.724802357	2.11339735354986	102.78039850678
2668	Guam	2001	105.96802316224	0.734267376	2.11395896124747	103.854064200993
2669	Guam	2002	107.06975746308	0.741284245	2.11447452757302	104.955282935507
2670	Guam	2003	108.09967998796	0.74591153	2.11264486885013	105.98703511911
2671	Guam	2004	109.10284556406	0.74901652	2.11043984772821	106.992405716332
2672	Guam	2005	109.62649328064	0.750865875	2.11211981007823	107.514373470562
2673	Guam	2006	109.79347982933	0.751176245	2.11142090740551	107.682058921924
2674	Guam	2007	109.80958828098	0.752625471	2.11139093561432	107.698197345366
2675	Guam	2008	109.94155865236	0.755152408	2.11298911209065	107.828569540269

TableS2 Frontier analysis of Smoking-Related Low Back Pain among WCBA

	location	year	val	SDI	frontier	eff_diff
2676	Guam	2009	110.01443452416	0.758252296	2.11687073301776	107.897563791142
2677	Guam	2010	109.96640108074	0.761484317	2.11174601267	107.85465506807
2678	Guam	2011	110.04048889783	0.763987394	2.1152310331087	107.925257864721
2679	Guam	2012	110.22840707084	0.766845984	2.110490138983	108.117916931857
2680	Guam	2013	110.65228593117	0.770224957	2.11049040750351	108.541795523666
2681	Guam	2014	110.94961266587	0.773441684	2.11052893862212	108.839083727248
2682	Guam	2015	111.16061531747	0.776723614	2.11743420853569	109.043181108934
2683	Guam	2016	111.15788065669	0.780511644	2.11213827582639	109.045742380864
2684	Guam	2017	110.58602269602	0.78555492	2.11067887068115	108.475343825339
2685	Guam	2018	110.22364275081	0.791294773	2.11382192476482	108.109820826045
2686	Guam	2019	109.514113615	0.796775452	2.11225282999618	107.401860785004
2687	Guam	2020	108.97997062938	0.800969472	2.11408594999943	106.865884679381
2688	Guam	2021	108.18081445237	0.803982203	2.11482981166061	106.065984640709
2689	Guatemala	1990	35.11247262631	0.311792455	2.36633562112931	32.7461370051807
2690	Guatemala	1991	34.91162261784	0.314329484	2.36093350519083	32.5506891126492
2691	Guatemala	1992	34.5896788828	0.319165481	2.34141699646438	32.2482618863356
2692	Guatemala	1993	34.17044565282	0.326840819	2.32280829311833	31.8476373597017
2693	Guatemala	1994	33.77060920286	0.3334691	2.2891962923152	31.4814129105448
2694	Guatemala	1995	33.32194559791	0.339642101	2.26832940266603	31.053616195244
2695	Guatemala	1996	32.99070217205	0.346961212	2.1307812858263	30.8599208862237
2696	Guatemala	1997	32.72321578183	0.355995963	2.1195552031106	30.6036605787194
2697	Guatemala	1998	32.48375387134	0.366461193	2.11158617366246	30.3721676976775
2698	Guatemala	1999	32.19546964109	0.374455316	2.115565118413	30.079904522677
2699	Guatemala	2000	31.99329237565	0.379877297	2.11075665118571	29.8825357244643
2700	Guatemala	2001	31.76735950303	0.386927686	2.10993751761623	29.6574219854138

TableS2 Frontier analysis of Smoking-Related Low Back Pain among WCBA

	location	year	val	SDI	frontier	eff_diff
2701	Guatemala	2002	31.48992970768	0.396899639	2.11236248113844	29.3775672265416
2702	Guatemala	2003	31.23794778911	0.408234164	2.11114628584754	29.1268015032625
2703	Guatemala	2004	30.94408811246	0.419777065	2.11494276007879	28.8291453523812
2704	Guatemala	2005	30.7828894297	0.430327529	2.11404979152145	28.6688396381786
2705	Guatemala	2006	30.55915739708	0.438856376	2.11117462502759	28.4479827720524
2706	Guatemala	2007	30.25445093926	0.447245965	2.11180946493407	28.1426414743259
2707	Guatemala	2008	30.00590272833	0.456541865	2.11381473614826	27.8920879921817
2708	Guatemala	2009	29.75837393142	0.464003077	2.11106508289273	27.6473088485273
2709	Guatemala	2010	29.48719236508	0.470477364	2.11228048874026	27.3749118763397
2710	Guatemala	2011	29.2123069724	0.476909684	2.11247434802371	27.0998326243763
2711	Guatemala	2012	28.88564733581	0.481862853	2.11322084704141	26.7724264887686
2712	Guatemala	2013	28.50801692508	0.487286692	2.11354222122485	26.3944747038552
2713	Guatemala	2014	28.20616657544	0.494027188	2.1123477830188	26.0938187924212
2714	Guatemala	2015	27.97378731665	0.500655607	2.11337262457103	25.860414692079
2715	Guatemala	2016	27.80735875486	0.507149792	2.11233763289024	25.6950211219698
2716	Guatemala	2017	27.53286903249	0.513735682	2.11516493284974	25.4177040996403
2717	Guatemala	2018	27.39056336179	0.520995469	2.11283087001269	25.2777324917773
2718	Guatemala	2019	27.36526836797	0.52863047	2.11654122941891	25.2487271385511
2719	Guatemala	2020	27.3847561713	0.534571414	2.11510084732055	25.2696553239795
2720	Guatemala	2021	27.60924515229	0.539972424	2.11431857062656	25.4949265816634
2721	Guinea	1990	9.20116379837	0.178295421	3.69298833369745	5.50817546467255
2722	Guinea	1991	9.24878609038	0.181459391	3.571222650033	5.677563440347
2723	Guinea	1992	9.25926832991	0.184485016	3.55387302795261	5.70539530195739
2724	Guinea	1993	9.27122663621	0.187686242	3.5480905084591	5.7231361277509
2725	Guinea	1994	9.26248337619	0.191128837	3.51209686382375	5.75038651236625

TableS2 Frontier analysis of Smoking-Related Low Back Pain among WCBA

	location	year	val	SDI	frontier	eff_diff
2726	Guinea	1995	9.22310502998	0.194573245	3.44206877132216	5.78103625865784
2727	Guinea	1996	9.13200383514	0.198633755	3.49857577497855	5.63342806016145
2728	Guinea	1997	9.05427851139	0.203280449	3.46754863266754	5.58672987872246
2729	Guinea	1998	9.01531383593	0.208003383	3.50512742356508	5.51018641236492
2730	Guinea	1999	8.88783838926	0.212412705	3.48151166041957	5.40632672884043
2731	Guinea	2000	8.71742879686	0.216649792	2.95352384269282	5.76390495416718
2732	Guinea	2001	8.63648325072	0.221090941	2.786764522599	5.849718728121
2733	Guinea	2002	8.57129311197	0.225898779	2.7411113767632	5.8301817352068
2734	Guinea	2003	8.42809669204	0.230522594	2.55979202767172	5.86830466436828
2735	Guinea	2004	8.27487716381	0.23513497	2.64664292114254	5.62823424266747
2736	Guinea	2005	8.18218207348	0.239691845	2.53755978389886	5.64462228958114
2737	Guinea	2006	8.11080035875	0.244010127	2.5572967815916	5.5535035771584
2738	Guinea	2007	8.05428421357	0.24847169	2.54665645485565	5.50762775871435
2739	Guinea	2008	8.00550444857	0.253167689	2.51959101423507	5.48591343433493
2740	Guinea	2009	7.97404068122	0.257552785	2.53688968431067	5.43715099690933
2741	Guinea	2010	7.94469499886	0.262405949	2.49891437778113	5.44578062107887
2742	Guinea	2011	7.88581094715	0.2676246	2.47859716654213	5.40721378060787
2743	Guinea	2012	7.78490562832	0.273428319	2.45469541593514	5.33021021238486
2744	Guinea	2013	7.69802173759	0.279600444	2.45366367780411	5.24435805978589
2745	Guinea	2014	7.63915580538	0.285961264	2.43048457637977	5.20867122900023
2746	Guinea	2015	7.59076616766	0.292363614	2.42381298056795	5.16695318709206
2747	Guinea	2016	7.58569931911	0.299112412	2.40091772913203	5.18478158997797
2748	Guinea	2017	7.59171788831	0.306881462	2.38329038168115	5.20842750662885
2749	Guinea	2018	7.60982143661	0.314839588	2.35530671785449	5.25451471875551
2750	Guinea	2019	7.62991777043	0.322839848	2.3355724436226	5.2943453268074

TableS2 Frontier analysis of Smoking-Related Low Back Pain among WCBA

	location	year	val	SDI	frontier	eff_diff
2751	Guinea	2020	7.64715845602	0.329877997	2.2994978259834	5.3476606300366
2752	Guinea	2021	7.6518692258	0.336401293	2.28772616935889	5.36414305644111
2753	Guinea-Bissau	1990	5.84821657063	0.207614839	3.46355905925618	2.38465751137382
2754	Guinea-Bissau	1991	5.74593268864	0.212505984	3.49819991991447	2.24773276872553
2755	Guinea-Bissau	1992	5.64762284507	0.217026088	2.89727800205436	2.75034484301564
2756	Guinea-Bissau	1993	5.55306398041	0.221678978	2.97793062370589	2.57513335670411
2757	Guinea-Bissau	1994	5.44590014575	0.226434933	2.72558407028544	2.72031607546456
2758	Guinea-Bissau	1995	5.34952306304	0.231223526	2.61408602277386	2.73543704026614
2759	Guinea-Bissau	1996	5.25981160329	0.236106374	2.61257533947477	2.64723626381523
2760	Guinea-Bissau	1997	5.16262201678	0.241360584	2.55100615111465	2.61161586566535
2761	Guinea-Bissau	1998	5.07583801875	0.244371797	2.5613264633134	2.5145115554366
2762	Guinea-Bissau	1999	5.0135139894	0.248217317	2.55040782387755	2.46310616552245
2763	Guinea-Bissau	2000	4.9910002279	0.252026519	2.54429493915681	2.44670528874319
2764	Guinea-Bissau	2001	4.94572144325	0.255786768	2.54444533834232	2.40127610490768
2765	Guinea-Bissau	2002	4.93017855906	0.259321816	2.53186183583471	2.39831672322529
2766	Guinea-Bissau	2003	4.92567881957	0.262747724	2.47046428053013	2.45521453903987
2767	Guinea-Bissau	2004	4.92459360976	0.266271736	2.48936095731934	2.43523265244066
2768	Guinea-Bissau	2005	4.92699856171	0.270056132	2.51831269282463	2.40868586888537
2769	Guinea-Bissau	2006	4.95039773926	0.273803987	2.45068643752213	2.49971130173787
2770	Guinea-Bissau	2007	5.00958222266	0.277805017	2.45594208266373	2.55364013999627
2771	Guinea-Bissau	2008	5.0765860298	0.282151502	2.45924258774814	2.61734344205186
2772	Guinea-Bissau	2009	5.15405075413	0.286423345	2.42281299719694	2.73123775693306
2773	Guinea-Bissau	2010	5.22067096961	0.291141639	2.42844982241197	2.79222114719803
2774	Guinea-Bissau	2011	5.27008939077	0.296682967	2.40099640045613	2.86909299031387
2775	Guinea-Bissau	2012	5.32228567663	0.301562342	2.39945168778261	2.92283398884739

TableS2 Frontier analysis of Smoking-Related Low Back Pain among WCBA

	location	year	val	SDI	frontier	eff_diff
2776	Guinea-Bissau	2013	5.36661814295	0.306602009	2.38726963517016	2.97934850777984
2777	Guinea-Bissau	2014	5.42812812229	0.311602905	2.35168179620456	3.07644632608544
2778	Guinea-Bissau	2015	5.50621048577	0.317090015	2.35890250789059	3.14730797787941
2779	Guinea-Bissau	2016	5.58985701068	0.32297967	2.34401458957866	3.24584242110134
2780	Guinea-Bissau	2017	5.69350332109	0.329326492	2.30449800779188	3.38900531329812
2781	Guinea-Bissau	2018	5.79456976708	0.335667833	2.28836368255025	3.50620608452975
2782	Guinea-Bissau	2019	5.8987719854	0.342156115	2.2459000801427	3.6528719052573
2783	Guinea-Bissau	2020	5.97162711542	0.347798998	2.1446327608761	3.8269943545439
2784	Guinea-Bissau	2021	6.07989072378	0.353109621	2.11133802715544	3.96855269662456
2785	Guyana	1990	24.69433181985	0.460430129	2.11334762502141	22.5809841948286
2786	Guyana	1991	24.83602933358	0.462677069	2.11384855421361	22.7221807793664
2787	Guyana	1992	24.85816393906	0.466753989	2.11425466580231	22.7439092732577
2788	Guyana	1993	25.07659608296	0.472174229	2.11298524090706	22.9636108420529
2789	Guyana	1994	25.29372911519	0.478992924	2.1125978835542	23.1811312316358
2790	Guyana	1995	25.59545006782	0.486573457	2.11367878731554	23.4817712805045
2791	Guyana	1996	25.86781813547	0.495216036	2.11238787472671	23.7554302607433
2792	Guyana	1997	26.3276035022	0.504362779	2.11068555563741	24.2169179465626
2793	Guyana	1998	26.67354133888	0.513068898	2.10932489939635	24.5642164394837
2794	Guyana	1999	26.96467589871	0.521595978	2.11113970697567	24.8535361917343
2795	Guyana	2000	27.19966684986	0.528693992	2.11255862497159	25.0871082248884
2796	Guyana	2001	27.41823643575	0.534869806	2.11335437635053	25.3048820593995
2797	Guyana	2002	27.50461386411	0.540514273	2.11406739919662	25.3905464649134
2798	Guyana	2003	27.55671868768	0.545516473	2.11466060398353	25.4420580836965
2799	Guyana	2004	27.54359439365	0.55032637	2.11186916663033	25.4317252270197
2800	Guyana	2005	27.56293366084	0.554844797	2.11260754382971	25.4503261170103

TableS2 Frontier analysis of Smoking-Related Low Back Pain among WCBA

	location	year	val	SDI	frontier	eff_diff
2801	Guyana	2006	27.43322588296	0.559465611	2.11155027315057	25.3216756098094
2802	Guyana	2007	27.29854028367	0.564541135	2.11491711300595	25.183623170664
2803	Guyana	2008	27.00217230425	0.569595206	2.11072520541899	24.891447098831
2804	Guyana	2009	26.65771540996	0.574769931	2.11461493283795	24.5431004771221
2805	Guyana	2010	26.36398294515	0.580083911	2.11354905635679	24.2504338887932
2806	Guyana	2011	26.02614640981	0.585688269	2.11198182925158	23.9141645805584
2807	Guyana	2012	25.63518173486	0.591336582	2.11266873048672	23.5225130043733
2808	Guyana	2013	25.30905413694	0.596826295	2.11520939438107	23.1938447425589
2809	Guyana	2014	24.96292215714	0.602136511	2.11572866703059	22.8471934901094
2810	Guyana	2015	24.71176569829	0.607605487	2.11365605764877	22.5981096406412
2811	Guyana	2016	24.57191151095	0.61391436	2.11380722130718	22.4581042896428
2812	Guyana	2017	24.48426974537	0.620602607	2.11013496522667	22.3741347801433
2813	Guyana	2018	24.29275624294	0.627167041	2.11105813637087	22.1816981065691
2814	Guyana	2019	24.00330797977	0.633640347	2.11301476375577	21.8902932160142
2815	Guyana	2020	24.03694083208	0.642284645	2.11325607553527	21.9236847565447
2816	Guyana	2021	24.12431113404	0.650812335	2.1150318693025	22.0092792647375
2817	Haiti	1990	32.18142229771	0.31033463	2.35823925537836	29.8231830423316
2818	Haiti	1991	32.19737355448	0.315455441	2.36547797783393	29.8318955766461
2819	Haiti	1992	32.03287216936	0.320389979	2.33906449378301	29.693807675577
2820	Haiti	1993	31.77856648552	0.324895851	2.321149487459	29.457416998061
2821	Haiti	1994	31.3973959858	0.328399456	2.32541032317691	29.0719856626231
2822	Haiti	1995	30.94893946753	0.332999152	2.29175605174018	28.6571834157898
2823	Haiti	1996	30.56671225564	0.338229396	2.26838737566072	28.2983248799793
2824	Haiti	1997	29.94297765452	0.343880049	2.17443504970182	27.7685426048182
2825	Haiti	1998	29.3493667478	0.349698073	2.11309350833721	27.2362732394628

TableS2 Frontier analysis of Smoking-Related Low Back Pain among WCBA

	location	year	val	SDI	frontier	eff_diff
2826	Haiti	1999	28.74232457115	0.355980743	2.11118059867254	26.6311439724775
2827	Haiti	2000	28.09369447802	0.362572209	2.11554591650493	25.9781485615151
2828	Haiti	2001	27.1958414637	0.368643508	2.11435811826462	25.0814833454354
2829	Haiti	2002	26.27233528856	0.374473325	2.11223996341535	24.1600953251446
2830	Haiti	2003	25.38085864578	0.380067255	2.11317776948	23.2676808763
2831	Haiti	2004	24.59533870223	0.385061512	2.11068212113486	22.4846565810951
2832	Haiti	2005	23.90035530958	0.38984542	2.11194444202063	21.7884108675594
2833	Haiti	2006	23.25810319098	0.394322001	2.11540432708293	21.1426988638971
2834	Haiti	2007	22.70921600384	0.398973441	2.11870727039812	20.5905087334419
2835	Haiti	2008	22.20870562545	0.403285871	2.11550518599735	20.0932004394526
2836	Haiti	2009	21.86304543375	0.407944424	2.11408127643636	19.7489641573136
2837	Haiti	2010	21.69994784128	0.411840105	2.11292138568031	19.5870264555997
2838	Haiti	2011	21.59153538742	0.41617719	2.11723016042731	19.4743052269927
2839	Haiti	2012	21.51808397028	0.420021528	2.11158458367873	19.4064993866013
2840	Haiti	2013	21.49549645691	0.423985549	2.11266302977866	19.3828334271313
2841	Haiti	2014	21.57232128602	0.427940902	2.11349463300423	19.4588266530158
2842	Haiti	2015	21.68933624789	0.43175676	2.11398049958675	19.5753557483033
2843	Haiti	2016	21.85495362875	0.435288722	2.11355103808472	19.7414025906653
2844	Haiti	2017	22.08271983145	0.438541003	2.11411915000065	19.9686006814494
2845	Haiti	2018	22.26894880814	0.441678925	2.11088109219946	20.1580677159405
2846	Haiti	2019	22.48253484757	0.444328854	2.11181308141462	20.3707217661554
2847	Haiti	2020	22.73488106545	0.446390614	2.1148474800625	20.6200335853875
2848	Haiti	2021	22.93085647993	0.448278285	2.11452920447292	20.8163272754571
2849	High SDI	1996	251.66490152324	NA	251.66490152324	0
2850	High SDI	1997	249.58822461132	NA	249.58822461132	0

TableS2 Frontier analysis of Smoking-Related Low Back Pain among WCBA

	location	year	val	SDI	frontier	eff_diff
2851	High SDI	2001	241.72301964131	NA	241.72301964131	0
2852	High SDI	2005	231.08580488287	NA	231.08580488287	0
2853	High SDI	2007	222.5551815558	NA	222.5551815558	0
2854	High SDI	2009	213.15870895133	NA	213.15870895133	0
2855	High SDI	2018	188.4728558177	NA	188.4728558177	0
2856	High-income Asia Pacific	1990	155.65275183189	0.767803786	2.11393254329257	153.538819288597
2857	High-income Asia Pacific	1991	154.71082711044	0.773332908	2.11527626758348	152.595550842857
2858	High-income Asia Pacific	1992	154.3652303976	0.778834454	2.11283589754096	152.252394500059
2859	High-income Asia Pacific	1993	154.68019632101	0.783967344	2.11133502437377	152.568861296636
2860	High-income Asia Pacific	1994	155.25420468155	0.789028457	2.11012007600759	153.144084605542
2861	High-income Asia Pacific	1995	157.12753207289	0.794099242	2.11163052926062	155.015901543629
2862	High-income Asia Pacific	1996	158.60544237528	0.798862943	2.11216264307382	156.493279732206
2863	High-income Asia Pacific	1997	159.65548483178	0.803127763	2.1117873189018	157.543697512878
2864	High-income Asia Pacific	1998	160.63912043823	0.806831345	2.11405277744314	158.525067660787
2865	High-income Asia Pacific	1999	161.42259875707	0.810468966	2.11645146306141	159.306147294009
2866	High-income Asia Pacific	2000	162.2997541675	0.814089018	2.11110404963803	160.188650117862
2867	High-income Asia Pacific	2001	163.5782766612	0.817544764	2.11593745443462	161.462339206765
2868	High-income Asia Pacific	2002	165.20855990149	0.821110491	2.112618481836	163.095941419654
2869	High-income Asia Pacific	2003	167.20961879637	0.824635809	2.1130803013271	165.096538495043
2870	High-income Asia Pacific	2004	169.09615448937	0.828073761	2.11275062192064	166.983403867449
2871	High-income Asia Pacific	2005	170.41173945475	0.831062145	2.11365040511292	168.298089049637
2872	High-income Asia Pacific	2006	170.23633688433	0.833766326	2.11493023588232	168.121406648448
2873	High-income Asia Pacific	2007	168.27930443036	0.836546108	2.1128026487079	166.166501781652
2874	High-income Asia Pacific	2008	165.20660421972	0.839279641	2.11377459521803	163.092829624502
2875	High-income Asia Pacific	2009	161.91606043967	0.841770635	2.11350736846621	159.802553071204

TableS2 Frontier analysis of Smoking-Related Low Back Pain among WCBA

	location	year	val	SDI	frontier	eff_diff
2876	High-income Asia Pacific	2010	159.32728210979	0.844682036	2.11429526209324	157.212986847697
2877	High-income Asia Pacific	2011	156.84869349736	0.847721965	2.1132732032885	154.735420294072
2878	High-income Asia Pacific	2012	154.13137067884	0.850693448	2.11584824020471	152.015522438635
2879	High-income Asia Pacific	2013	151.30647888623	0.853671415	2.11349288613509	149.192986000095
2880	High-income Asia Pacific	2014	148.74299574088	0.856611561	2.11356121354354	146.629434527336
2881	High-income Asia Pacific	2015	146.38489637849	0.859602611	2.11567256391016	144.26922381458
2882	High-income Asia Pacific	2016	145.38109282655	0.862733183	2.11315082480603	143.267942001744
2883	High-income Asia Pacific	2017	144.24717648637	0.86587024	2.11248036683355	142.134696119536
2884	High-income Asia Pacific	2018	143.19079435444	0.869072595	2.1118952200412	141.078899134399
2885	High-income Asia Pacific	2019	141.76878551549	0.872218835	2.11515496929733	139.653630546193
2886	High-income Asia Pacific	2020	134.86687939866	0.874437255	2.11627103020045	132.75060836846
2887	High-income Asia Pacific	2021	137.39044084084	0.876767	2.11271073164964	135.27773010919
2888	High-income North America	1990	329.48953015667	0.765659862	2.11411021043147	327.375419946239
2889	High-income North America	1991	327.03434296787	0.768049984	2.11421301143889	324.920129956431
2890	High-income North America	1992	324.21703469654	0.771549819	2.11199963876625	322.105035057774
2891	High-income North America	1993	320.69135987323	0.774977626	2.11554484522368	318.575815028006
2892	High-income North America	1994	317.74987812532	0.778206755	2.11541708788619	315.634461037434
2893	High-income North America	1995	315.19824562765	0.781348237	2.11552810072641	313.082717526924
2894	High-income North America	1996	312.04727372704	0.784104747	2.11089942400525	309.936374303035
2895	High-income North America	1997	306.41984429147	0.78634761	2.115851503158	304.303992788312
2896	High-income North America	1998	300.65572756761	0.788569028	2.11316995944356	298.542557608166
2897	High-income North America	1999	295.92680797832	0.791196294	2.11331859484695	293.813489383473
2898	High-income North America	2000	292.464532044	0.794997034	2.11424678429656	290.350285259703
2899	High-income North America	2001	290.0452141344	0.799274822	2.11237489382088	287.932839240579
2900	High-income North America	2002	287.40438625268	0.802961331	2.11270502733486	285.291681225345

TableS2 Frontier analysis of Smoking-Related Low Back Pain among WCBA

	location	year	val	SDI	frontier	eff_diff
2901	High-income North America	2003	284.14621074616	0.805821251	2.1146034042461	282.031607341914
2902	High-income North America	2004	280.12931276777	0.807991191	2.11210439810075	278.017208369669
2903	High-income North America	2005	275.14472539381	0.808886691	2.115071056811	273.029654336999
2904	High-income North America	2006	269.13566995065	0.809721162	2.11205344645985	267.02361650419
2905	High-income North America	2007	262.39437776258	0.812486106	2.11352316999431	260.280854592586
2906	High-income North America	2008	255.67315499671	0.817083931	2.11386441059018	253.55929058612
2907	High-income North America	2009	249.61528854906	0.822470006	2.11384680129696	247.501441747763
2908	High-income North America	2010	244.72384394168	0.827814138	2.11585935322861	242.607984588451
2909	High-income North America	2011	240.2123478888	0.832060879	2.11151824779739	238.100829641003
2910	High-income North America	2012	235.42890227844	0.835668937	2.11286180318085	233.316040475259
2911	High-income North America	2013	230.72863521787	0.839058685	2.11276818754746	228.615867030323
2912	High-income North America	2014	226.37792546204	0.842340953	2.11580084096493	224.262124621075
2913	High-income North America	2015	223.06361924887	0.845876702	2.11289440130623	220.950724847564
2914	High-income North America	2016	220.9338483247	0.849423368	2.11162334223061	218.822224982469
2915	High-income North America	2017	219.09103229761	0.852960115	2.11430253787097	216.976729759739
2916	High-income North America	2018	217.66180087874	0.856316178	2.11348182356647	215.548319055174
2917	High-income North America	2019	216.65488585934	0.859749397	2.11441290908189	214.540472950258
2918	High-income North America	2020	215.12254019499	0.861874247	2.11389068070797	213.008649514282
2919	High-income North America	2021	213.34604828973	0.863465474	2.11385916074767	211.232189128982
2920	High-middle SDI	2003	106.6614091367	NA	106.6614091367	0
2921	High-middle SDI	2008	108.71549604298	NA	108.71549604298	0
2922	High-middle SDI	2015	109.44457131066	NA	109.44457131066	0
2923	High-middle SDI	2018	110.91454165784	NA	110.91454165784	0
2924	High-middle SDI	2019	111.25790033296	NA	111.25790033296	0
2925	Honduras	1990	35.98737636794	0.332042889	2.31047880896261	33.6768975589774

TableS2 Frontier analysis of Smoking-Related Low Back Pain among WCBA

	location	year	val	SDI	frontier	eff_diff
2926	Honduras	1991	35.8399652404	0.337034649	2.27299775803904	33.566967482361
2927	Honduras	1992	35.59739963754	0.342305657	2.24482976877529	33.3525698687647
2928	Honduras	1993	35.34525660525	0.347838446	2.1433955810294	33.2018610242206
2929	Honduras	1994	35.09176620996	0.353164852	2.12555786732795	32.9662083426321
2930	Honduras	1995	34.82875254003	0.358787268	2.11466311907136	32.7140894209586
2931	Honduras	1996	34.49008871601	0.364328424	2.11220842161689	32.3778802943931
2932	Honduras	1997	34.17498451561	0.369983894	2.11408926622933	32.0608952493807
2933	Honduras	1998	33.84897951799	0.375950111	2.1158317197622	31.7331477982278
2934	Honduras	1999	33.50694411996	0.381752316	2.11601006062214	31.3909340593379
2935	Honduras	2000	33.33947189455	0.387988053	2.11591039062792	31.2235615039221
2936	Honduras	2001	32.98332821383	0.394174012	2.11286247590286	30.8704657379271
2937	Honduras	2002	32.50601131468	0.400371798	2.11370377393328	30.3923075407467
2938	Honduras	2003	32.1462400334	0.406668381	2.11708312706333	30.0291569063367
2939	Honduras	2004	31.7735406626	0.413061816	2.11528633957732	29.6582543230227
2940	Honduras	2005	31.40878589491	0.419612332	2.11802128765672	29.2907646072533
2941	Honduras	2006	31.10503538938	0.426352997	2.11821753542323	28.9868178539568
2942	Honduras	2007	30.74720212439	0.433240315	2.11716537576156	28.6300367486284
2943	Honduras	2008	30.40980272848	0.439998164	2.11267976461275	28.2971229638673
2944	Honduras	2009	30.14041741921	0.446151054	2.11396835348703	28.026449065723
2945	Honduras	2010	30.08156985075	0.452382226	2.11168265724804	27.969887193502
2946	Honduras	2011	30.1811374443	0.458700329	2.11347468568175	28.0676627586183
2947	Honduras	2012	30.36137653862	0.464925155	2.11365636638943	28.2477201722306
2948	Honduras	2013	30.59795716256	0.470766833	2.11234117920976	28.4856159833502
2949	Honduras	2014	30.80828416628	0.476587799	2.1115040960282	28.6967800702518
2950	Honduras	2015	31.06065680852	0.482294176	2.11446971479869	28.9461870937213

TableS2 Frontier analysis of Smoking-Related Low Back Pain among WCBA

	location	year	val	SDI	frontier	eff_diff
2951	Honduras	2016	31.28772129302	0.487705932	2.11538435203862	29.1723369409814
2952	Honduras	2017	31.67870568971	0.493130694	2.11346036484478	29.5652453248652
2953	Honduras	2018	31.97078766805	0.498657227	2.11487487516411	29.8559127928859
2954	Honduras	2019	32.32936521398	0.504156505	2.11650351131352	30.2128617026665
2955	Honduras	2020	32.40811659867	0.508669246	2.11618120286543	30.2919353958046
2956	Honduras	2021	32.78122389194	0.513037248	2.11269506370924	30.6685288282308
2957	Hungary	1990	489.28648190078	0.649419991	2.111655869411	487.174826031369
2958	Hungary	1991	486.58449981388	0.653707155	2.11353098859045	484.47096882529
2959	Hungary	1992	483.24227296453	0.660382905	2.1131978851513	481.129075079379
2960	Hungary	1993	479.51110889309	0.667543556	2.11476255301721	477.396346340073
2961	Hungary	1994	476.95416910754	0.675051406	2.11389148776158	474.840277619778
2962	Hungary	1995	475.10796506711	0.683186412	2.1113181323809	472.996646934729
2963	Hungary	1996	475.68215164626	0.690883334	2.111624846688	473.570526799572
2964	Hungary	1997	477.83994174803	0.697935514	2.11229823335738	475.727643514673
2965	Hungary	1998	478.39584263865	0.70420272	2.11042024355711	476.285422395093
2966	Hungary	1999	479.12648389662	0.709980627	2.11346454880443	477.013019347816
2967	Hungary	2000	479.5446342288	0.715805281	2.11148848213879	477.433145746661
2968	Hungary	2001	478.1503428713	0.722099055	2.11608241859785	476.034260452702
2969	Hungary	2002	474.42275483769	0.728384596	2.11407109844907	472.308683739241
2970	Hungary	2003	469.13413341429	0.734450658	2.11306342723969	467.02106998705
2971	Hungary	2004	464.05439245827	0.740099975	2.110237630608	461.944154827662
2972	Hungary	2005	459.03803365574	0.745356806	2.11550090198239	456.922532753758
2973	Hungary	2006	455.16067158925	0.750515381	2.11157168419988	453.04909990505
2974	Hungary	2007	450.74077829327	0.754942942	2.11280579061057	448.627972502659
2975	Hungary	2008	446.49711603513	0.759317468	2.11389748750674	444.383218547623

TableS2 Frontier analysis of Smoking-Related Low Back Pain among WCBA

	location	year	val	SDI	frontier	eff_diff
2976	Hungary	2009	442.16998278307	0.763563687	2.1115656846023	440.058417098468
2977	Hungary	2010	437.44947397768	0.767328649	2.11532258397833	435.334151393702
2978	Hungary	2011	431.0430353925	0.769701942	2.11306093347681	428.929974459023
2979	Hungary	2012	424.06683843868	0.770784245	2.11447910230949	421.95235933637
2980	Hungary	2013	416.91567684171	0.771561698	2.11539376850924	414.800283073201
2981	Hungary	2014	410.54283616016	0.772575046	2.11246549466115	408.430370665499
2982	Hungary	2015	405.91445446034	0.774047383	2.11337527967411	403.801079180666
2983	Hungary	2016	401.96564941737	0.776150305	2.11450071585575	399.851148701514
2984	Hungary	2017	398.24275374466	0.779116417	2.11502838484185	396.127725359818
2985	Hungary	2018	394.08690803838	0.782083661	2.11388967845573	391.973018359924
2986	Hungary	2019	391.38257662413	0.784910309	2.11295555715644	389.269621066974
2987	Hungary	2020	390.02445604485	0.78762039	2.11048675220257	387.913969292647
2988	Hungary	2021	390.53148065304	0.790754768	2.11499180422485	388.416488848815
2989	Iceland	1990	236.39098026982	0.764212517	2.11487829713338	234.276101972687
2990	Iceland	1991	239.15715863266	0.769027393	2.11256874240547	237.044589890255
2991	Iceland	1992	241.30727532372	0.772706415	2.11427336006842	239.193001963652
2992	Iceland	1993	243.09723994968	0.776389423	2.11129258400085	240.985947365679
2993	Iceland	1994	244.68226441407	0.779967961	2.11226054991319	242.570003864157
2994	Iceland	1995	246.02855387351	0.782740057	2.11414584752087	243.914408025989
2995	Iceland	1996	245.31055801781	0.78535642	2.10995051368354	243.200607504126
2996	Iceland	1997	244.78547825041	0.788528335	2.11714198225185	242.668336268158
2997	Iceland	1998	242.01650925081	0.792626368	2.11542318928561	239.901086061524
2998	Iceland	1999	237.60137611556	0.797368808	2.11223613017442	235.489139985386
2999	Iceland	2000	234.12662437392	0.80303033	2.11301804331403	232.013606330606
3000	Iceland	2001	229.8962975118	0.808965786	2.11377941696983	227.78251809483

TableS2 Frontier analysis of Smoking-Related Low Back Pain among WCBA

	location	year	val	SDI	frontier	eff_diff
3001	Iceland	2002	224.72689280259	0.813400392	2.11406989648348	222.612822906107
3002	Iceland	2003	221.03656988878	0.816597067	2.11428343287918	218.922286455901
3003	Iceland	2004	216.5094097596	0.819528087	2.11128041024033	214.39812934936
3004	Iceland	2005	211.31259532218	0.822766536	2.1133045472519	209.199290774928
3005	Iceland	2006	205.46534763983	0.826244743	2.11825896864963	203.34708867118
3006	Iceland	2007	199.8471117199	0.83028989	2.11283605079	197.73427566911
3007	Iceland	2008	193.7648245264	0.834622678	2.11058863022153	191.654235896178
3008	Iceland	2009	188.47362671564	0.838121013	2.11033772406542	186.363288991575
3009	Iceland	2010	185.37770218169	0.841384642	2.11281720615494	183.264884975535
3010	Iceland	2011	183.57654757969	0.84461993	2.11416854020557	181.462379039484
3011	Iceland	2012	181.31678159547	0.847299444	2.11521169432293	179.201569901147
3012	Iceland	2013	179.38221107894	0.850173948	2.11361106788855	177.268600011051
3013	Iceland	2014	177.68120165288	0.853558697	2.11164386862773	175.569557784252
3014	Iceland	2015	175.81818753778	0.857724976	2.11774461005508	173.700442927725
3015	Iceland	2016	174.64974137423	0.861844467	2.11128006074277	172.538461313487
3016	Iceland	2017	173.71723589106	0.865702891	2.11279748432873	171.604438406731
3017	Iceland	2018	173.63216772262	0.869219822	2.114274456521	171.517893266099
3018	Iceland	2019	174.40340487467	0.87242558	2.11270052015356	172.290704354516
3019	Iceland	2020	174.13001089078	0.874323153	2.11041294746078	172.019597943319
3020	Iceland	2021	177.02736815513	0.87636168	2.11307579791879	174.914292357211
3021	India	1990	22.77464789787	0.332593603	2.30637765179	20.46827024608
3022	India	1991	22.58988382674	0.338591779	2.27507099467783	20.3148128320622
3023	India	1992	22.36248209141	0.344591727	2.173800362267	20.188681729143
3024	India	1993	22.06153547056	0.350685397	2.11929402747902	19.942241443081
3025	India	1994	21.71503080736	0.35699603	2.11740410269875	19.5976267046612

TableS2 Frontier analysis of Smoking-Related Low Back Pain among WCBA

	location	year	val	SDI	frontier	eff_diff
3026	India	1995	21.31622811041	0.363418501	2.112246354576	19.203981755834
3027	India	1996	20.71351264103	0.370018644	2.11500198904183	18.5985106519882
3028	India	1997	19.7237730544	0.376358763	2.11445786072847	17.6093151936715
3029	India	1998	18.66250561535	0.382817848	2.11082050950917	16.5516851058408
3030	India	1999	17.76691942258	0.389453889	2.11313061271625	15.6537888098637
3031	India	2000	17.28630160799	0.395752331	2.11866014225	15.16764146574
3032	India	2001	16.99050791793	0.401837472	2.11294501737633	14.8775629005537
3033	India	2002	16.75212408034	0.407411414	2.11394001701618	14.6381840633238
3034	India	2003	16.55956117051	0.413115783	2.11301111978389	14.4465500507261
3035	India	2004	16.41623478859	0.419184961	2.11171613406797	14.304518654522
3036	India	2005	16.30794447803	0.425913062	2.11153473130658	14.1964097467234
3037	India	2006	16.18789509761	0.433321558	2.11136024207389	14.0765348555361
3038	India	2007	16.02430414152	0.44135146	2.11481063067899	13.909493510841
3039	India	2008	15.81657653701	0.449235851	2.11372474321833	13.7028517937917
3040	India	2009	15.59908594322	0.457672332	2.1147856546306	13.4843002885894
3041	India	2010	15.38121164344	0.467018996	2.11473787321766	13.2664737702223
3042	India	2011	15.169300168	0.476832909	2.11315040851895	13.0561497594811
3043	India	2012	14.95736328699	0.487210876	2.11197842537288	12.8453848616171
3044	India	2013	14.75441799674	0.498317981	2.1143083083157	12.6401096884243
3045	India	2014	14.56025589882	0.5097221	2.11226362241943	12.4479922764006
3046	India	2015	14.39032464082	0.521242719	2.11517771987019	12.2751469209498
3047	India	2016	14.15029640142	0.532220023	2.11610667649219	12.0341897249278
3048	India	2017	13.80599754199	0.542388507	2.11483852289463	11.6911590190954
3049	India	2018	13.47892212696	0.551930326	2.11356045396309	11.3653616729969
3050	India	2019	13.2897867744	0.560809927	2.11225329479387	11.1775334796061

TableS2 Frontier analysis of Smoking-Related Low Back Pain among WCBA

	location	year	val	SDI	frontier	eff_diff
3051	India	2020	13.49035070056	0.568138149	2.11535201230693	11.3749986882531
3052	India	2021	13.41371760274	0.575401649	2.11515340393988	11.2985641988001
3053	Indonesia	1990	14.09442931769	0.457134954	2.11241688940724	11.9820124282828
3054	Indonesia	1991	14.29265049856	0.466505583	2.1120462100725	12.1806042884875
3055	Indonesia	1992	14.50228501364	0.475753157	2.11191535324253	12.3903696603975
3056	Indonesia	1993	14.72565412379	0.484775241	2.11097021707866	12.6146839067113
3057	Indonesia	1994	14.97531559495	0.4936071	2.11451480124964	12.8608007937004
3058	Indonesia	1995	15.26433175659	0.502008381	2.11392193176034	13.1504098248297
3059	Indonesia	1996	15.53982202713	0.510108146	2.11378491980772	13.4260371073223
3060	Indonesia	1997	15.85515692056	0.518102189	2.11323504531	13.74192187525
3061	Indonesia	1998	16.18942965006	0.524045604	2.11117598290177	14.0782536671582
3062	Indonesia	1999	16.51893262255	0.52917633	2.11454117384959	14.4043914487004
3063	Indonesia	2000	16.84193647978	0.533789587	2.11485234005909	14.7270841397209
3064	Indonesia	2001	17.2345213376	0.537977981	2.1151176462636	15.1194036913364
3065	Indonesia	2002	17.64883166356	0.542245676	2.11195112121533	15.5368805423447
3066	Indonesia	2003	18.06140192485	0.546550264	2.11289339813604	15.948508526714
3067	Indonesia	2004	18.46703752524	0.550902314	2.11366079878337	16.3533767264566
3068	Indonesia	2005	18.82460791246	0.555424913	2.11304800166873	16.7115599107913
3069	Indonesia	2006	19.17547686571	0.560265924	2.11291946448029	17.0625574012297
3070	Indonesia	2007	19.54354888078	0.565811896	2.11317829187985	17.4303705889002
3071	Indonesia	2008	19.89712876442	0.572225267	2.11533405098453	17.7817947134355
3072	Indonesia	2009	20.20722017584	0.578866902	2.11374306258211	18.0934771132579
3073	Indonesia	2010	20.42419581873	0.585982419	2.11529220418143	18.3089036145486
3074	Indonesia	2011	20.49300137953	0.593652985	2.11311043071101	18.379890948819
3075	Indonesia	2012	20.4095368343	0.601185701	2.11296590482262	18.2965709294774

TableS2 Frontier analysis of Smoking-Related Low Back Pain among WCBA

	location	year	val	SDI	frontier	eff_diff
3076	Indonesia	2013	20.24818501429	0.608509904	2.11564958251931	18.1325354317707
3077	Indonesia	2014	20.04657409375	0.615499887	2.1112768625308	17.9352972312192
3078	Indonesia	2015	19.86761111553	0.622222469	2.1122635113994	17.7553476041306
3079	Indonesia	2016	19.6796420352	0.628666625	2.11481268736912	17.5648293478309
3080	Indonesia	2017	19.42667174896	0.634895745	2.11059148220561	17.3160802667544
3081	Indonesia	2018	19.19815556976	0.64094875	2.11305367892597	17.085101890834
3082	Indonesia	2019	19.03778535155	0.646865457	2.11294411858469	16.9248412329653
3083	Indonesia	2020	18.98519777199	0.651926648	2.11616759089154	16.8690301810985
3084	Indonesia	2021	18.78161101727	0.656868336	2.11362266487179	16.6679883523982
3085	Iran	1990	34.38734957733	0.453799944	2.11252117536657	32.2748284019634
3086	Iran	1991	34.32244600495	0.468982858	2.11509315147534	32.2073528534747
3087	Iran	1992	34.41009748349	0.480626697	2.11145243538779	32.2986450481022
3088	Iran	1993	34.80637315171	0.492277025	2.11382689702217	32.6925462546878
3089	Iran	1994	35.33057005407	0.505072857	2.11269610404253	33.2178739500275
3090	Iran	1995	35.64495377499	0.517531494	2.11325965443377	33.5316941205562
3091	Iran	1996	35.61695944476	0.52860072	2.1120385893163	33.5049208554437
3092	Iran	1997	35.43659180738	0.537727779	2.11245679399776	33.3241350133822
3093	Iran	1998	35.22976572752	0.546366017	2.11282118431269	33.1169445432073
3094	Iran	1999	35.10356588547	0.555624332	2.11353875594389	32.9900271295261
3095	Iran	2000	35.16893484685	0.565474561	2.11559487627045	33.0533399705795
3096	Iran	2001	35.47033356999	0.574618208	2.11380724231032	33.3565263276797
3097	Iran	2002	35.91891938192	0.584097268	2.11739850940265	33.8015208725174
3098	Iran	2003	36.53844859215	0.593963012	2.11582992131579	34.4226186708342
3099	Iran	2004	37.18994501805	0.60371745	2.11411974333314	35.0758252747169
3100	Iran	2005	37.70982918399	0.613291056	2.11576575322057	35.5940634307694

TableS2 Frontier analysis of Smoking-Related Low Back Pain among WCBA

	location	year	val	SDI	frontier	eff_diff
3101	Iran	2006	38.10614280188	0.621268095	2.11244516803559	35.9936976338444
3102	Iran	2007	38.37729651986	0.627749442	2.11281377659173	36.2644827432683
3103	Iran	2008	38.68397924785	0.63286223	2.11275162402523	36.5712276238248
3104	Iran	2009	39.14444710798	0.63739838	2.11158705155809	37.0328600564219
3105	Iran	2010	39.86930894111	0.64255917	2.11320801363425	37.7561009274757
3106	Iran	2011	40.79534181995	0.647899089	2.11642417218833	38.6789176477617
3107	Iran	2012	41.78859698521	0.651500587	2.11160593057931	39.6769910546307
3108	Iran	2013	42.79242697508	0.654475289	2.11304867731921	40.6793782977608
3109	Iran	2014	43.87830693932	0.657961511	2.1149043518164	41.7634025875036
3110	Iran	2015	45.14120956735	0.662057987	2.11092527715424	43.0302842901958
3111	Iran	2016	46.57396583612	0.667652445	2.11150365569491	44.4624621804251
3112	Iran	2017	48.03633569222	0.674343462	2.11417434076304	45.922161351457
3113	Iran	2018	49.4171504348	0.680909621	2.11538366960072	47.3017667651993
3114	Iran	2019	50.63950829475	0.686741662	2.11441851257743	48.5250897821726
3115	Iran	2020	51.31917987143	0.691918763	2.11236530761651	49.2068145638135
3116	Iran	2021	51.70691578951	0.697207398	2.11541161047173	49.5915041790383
3117	Iraq	1990	25.91766002531	0.412044173	2.11046991391239	23.8071901113976
3118	Iraq	1991	26.11516308141	0.415814116	2.11229782921987	24.0028652521901
3119	Iraq	1992	26.17223410617	0.420353594	2.1159491978208	24.0562849083492
3120	Iraq	1993	25.96666167905	0.424834933	2.11354709478229	23.8531145842677
3121	Iraq	1994	25.60076169606	0.428751731	2.1121705197124	23.4885911763476
3122	Iraq	1995	25.36455506994	0.432075545	2.11356026018443	23.2509948097556
3123	Iraq	1996	25.3163052105	0.436818479	2.11114454032197	23.205160670178
3124	Iraq	1997	25.25733688925	0.443104027	2.11319838855187	23.1441385006981
3125	Iraq	1998	25.22201609132	0.452384092	2.112805044115	23.109211047205

TableS2 Frontier analysis of Smoking-Related Low Back Pain among WCBA

	location	year	val	SDI	frontier	eff_diff
3126	Iraq	1999	25.15290867076	0.462736441	2.11497646155339	23.0379322092066
3127	Iraq	2000	25.12272916727	0.472376399	2.11318301008593	23.0095461571841
3128	Iraq	2001	25.06070413457	0.48145832	2.11030213327403	22.950402001296
3129	Iraq	2002	25.07469244131	0.488664737	2.11092928840268	22.9637631529073
3130	Iraq	2003	25.1036900154	0.492174445	2.1146781738294	22.9890118415706
3131	Iraq	2004	25.11450534044	0.498933881	2.11403641186518	23.0004689285748
3132	Iraq	2005	25.08150876604	0.504719431	2.11492970107485	22.9665790649651
3133	Iraq	2006	25.11879804926	0.510918049	2.11564740986875	23.0031506393913
3134	Iraq	2007	25.27593477478	0.517698972	2.11376942822918	23.1621653465508
3135	Iraq	2008	25.23328929107	0.525242908	2.11237936304887	23.1209099280211
3136	Iraq	2009	24.79954428754	0.533200914	2.11042029329898	22.689123994241
3137	Iraq	2010	24.32266789924	0.542343278	2.11604578165863	22.2066221175814
3138	Iraq	2011	23.82310750748	0.553048555	2.11583516394072	21.7072723435393
3139	Iraq	2012	23.21092330076	0.564745467	2.1130029522178	21.0979203485422
3140	Iraq	2013	22.48166334898	0.576968998	2.11315824885391	20.3685051001261
3141	Iraq	2014	21.94734615042	0.588317652	2.11271448885246	19.8346316615675
3142	Iraq	2015	21.76277233488	0.599494007	2.11224157274884	19.6505307621312
3143	Iraq	2016	21.73073777406	0.611508646	2.11508255881423	19.6156552152458
3144	Iraq	2017	21.75460084123	0.622674532	2.11318827951851	19.6414125617115
3145	Iraq	2018	21.78300316182	0.633232108	2.11352248520224	19.6694806766178
3146	Iraq	2019	21.82827229764	0.643635318	2.11325154702985	19.7150207506101
3147	Iraq	2020	21.74928425311	0.65330038	2.11451611267192	19.6347681404381
3148	Iraq	2021	21.64823064097	0.662626231	2.11597307848866	19.5322575624813
3149	Ireland	1990	242.54656157986	0.719891819	2.11689583070859	240.429665749151
3150	Ireland	1991	245.89011032282	0.725195019	2.11502112412315	243.775089198697

TableS2 Frontier analysis of Smoking-Related Low Back Pain among WCBA

	location	year	val	SDI	frontier	eff_diff
3151	Ireland	1992	247.98924795522	0.731918691	2.11285202299554	245.876395932224
3152	Ireland	1993	248.78283648077	0.738384074	2.11389910925679	246.668937371513
3153	Ireland	1994	249.23660594367	0.744011728	2.11425095218455	247.122354991485
3154	Ireland	1995	247.78457965417	0.748921575	2.11350244217258	245.671077211997
3155	Ireland	1996	246.15817558749	0.754010124	2.1139602861291	244.044215301361
3156	Ireland	1997	244.010017207	0.759970384	2.11206165238687	241.897955554613
3157	Ireland	1998	242.32696304699	0.766488721	2.11364504370589	240.213318003284
3158	Ireland	1999	240.40266655872	0.773538834	2.11406200124865	238.288604557471
3159	Ireland	2000	240.31870799303	0.780190655	2.11574010689027	238.20296788614
3160	Ireland	2001	240.23854329692	0.786353741	2.11022965927466	238.128313637645
3161	Ireland	2002	239.99530508111	0.793010606	2.11546079393531	237.879844287175
3162	Ireland	2003	239.13376731294	0.800005865	2.11474528445451	237.019022028486
3163	Ireland	2004	238.54559106937	0.80685314	2.11234644367312	236.433244625697
3164	Ireland	2005	237.78543284936	0.811713818	2.1128679212797	235.67256492808
3165	Ireland	2006	236.89754464211	0.814505479	2.11273912336433	234.784805518746
3166	Ireland	2007	234.67750653203	0.816931026	2.11498811781566	232.562518414214
3167	Ireland	2008	233.48897959552	0.819932128	2.11364471815465	231.375334877365
3168	Ireland	2009	232.56841489711	0.823929666	2.11085644184258	230.457558455267
3169	Ireland	2010	231.52230982474	0.828103848	2.11227635045586	229.410033474284
3170	Ireland	2011	229.49522790527	0.831804792	2.112439988125	227.382787917145
3171	Ireland	2012	225.68341135314	0.835325359	2.11494548280213	223.568465870338
3172	Ireland	2013	220.69905370033	0.838644433	2.11233948162162	218.586714218708
3173	Ireland	2014	215.3796078762	0.842082115	2.11165416502271	213.267953711177
3174	Ireland	2015	210.83644895507	0.847437887	2.11352201161821	208.722926943452
3175	Ireland	2016	207.9260947293	0.852642502	2.11337719198753	205.812717537312

TableS2 Frontier analysis of Smoking-Related Low Back Pain among WCBA

	location	year	val	SDI	frontier	eff_diff
3176	Ireland	2017	204.49231168469	0.857816164	2.1129695429097	202.37934214178
3177	Ireland	2018	201.2737948374	0.863925284	2.11333995136943	199.160454886031
3178	Ireland	2019	198.34347902021	0.869412503	2.1148086557603	196.22867036445
3179	Ireland	2020	193.4513172291	0.871958822	2.1118364214083	191.339480807692
3180	Ireland	2021	191.87545304048	0.87375385	2.11420762336062	189.761245417119
3181	Israel	1990	224.01643136442	0.709178347	2.11421643625446	221.902214928166
3182	Israel	1991	222.21157779232	0.713371633	2.1133282274527	220.098249564867
3183	Israel	1992	221.32909759553	0.717897923	2.11563843309732	219.213459162433
3184	Israel	1993	220.03635093218	0.722250176	2.11425948461357	217.922091447566
3185	Israel	1994	218.19540412487	0.726654736	2.11264119291375	216.082762931956
3186	Israel	1995	215.88163157575	0.731127859	2.11413182960618	213.767499746144
3187	Israel	1996	212.77710663253	0.734714026	2.11527977692208	210.661826855608
3188	Israel	1997	208.58531317525	0.738015277	2.11272879602382	206.472584379226
3189	Israel	1998	204.54302449724	0.74192719	2.11058876547621	202.432435731764
3190	Israel	1999	200.94551679421	0.745443338	2.11783201492492	198.827684779285
3191	Israel	2000	197.39926057797	0.749184084	2.11525355685775	195.284007021112
3192	Israel	2001	193.36304635868	0.752834297	2.11467935575228	191.248367002928
3193	Israel	2002	189.04619953858	0.755988076	2.11589321549893	186.930306323081
3194	Israel	2003	184.99034225867	0.758875409	2.11417765186805	182.876164606802
3195	Israel	2004	181.22400676188	0.762213291	2.1136933730879	179.110313388792
3196	Israel	2005	178.23969597751	0.765543461	2.11450510516895	176.125190872341
3197	Israel	2006	175.12096761867	0.768733898	2.11701201819507	173.003955600475
3198	Israel	2007	171.52299295671	0.770756143	2.11370619303104	169.409286763679
3199	Israel	2008	167.66002345687	0.772011615	2.11465585587018	165.545367601
3200	Israel	2009	164.48893950355	0.773303913	2.11484331062986	162.37409619292

TableS2 Frontier analysis of Smoking-Related Low Back Pain among WCBA

	location	year	val	SDI	frontier	eff_diff
3201	Israel	2010	161.72116126818	0.77525953	2.11568317103	159.60547809715
3202	Israel	2011	159.59632742244	0.777649669	2.11506479963842	157.481262622802
3203	Israel	2012	157.30458146583	0.780403415	2.11216899536253	155.192412470467
3204	Israel	2013	155.7852920424	0.783267167	2.11363111878772	153.671660923612
3205	Israel	2014	153.99068714642	0.786151084	2.11112528071306	151.879561865707
3206	Israel	2015	153.10487645897	0.788874457	2.11122597428557	150.993650484684
3207	Israel	2016	152.65684361528	0.791772445	2.11492890139489	150.541914713885
3208	Israel	2017	150.99332218435	0.795173437	2.11346968394819	148.879852500402
3209	Israel	2018	149.20689995632	0.799124994	2.11337841638211	147.093521539938
3210	Israel	2019	147.56370855762	0.803232991	2.11284555572708	145.450863001893
3211	Israel	2020	145.22230133133	0.806351589	2.11281758198667	143.109483749343
3212	Israel	2021	146.00239693378	0.809011652	2.11386894138695	143.888527992393
3213	Italy	1990	239.24669157494	0.706255224	2.11263853107082	237.134053043869
3214	Italy	1991	234.81351615623	0.711457349	2.112082229708	232.701433926522
3215	Italy	1992	231.93837442565	0.716649746	2.11688411576541	229.821490309885
3216	Italy	1993	229.90029862253	0.721720992	2.11113989340103	227.789158729129
3217	Italy	1994	229.05703180896	0.726870913	2.1148565819993	226.942175226961
3218	Italy	1995	229.9460452292	0.731644082	2.11343065950956	227.83261456969
3219	Italy	1996	230.99059095624	0.73605087	2.11219595855972	228.87839499768
3220	Italy	1997	231.29368903932	0.739972336	2.11469602527667	229.178993014043
3221	Italy	1998	231.77110450505	0.743101596	2.11133111177767	229.659773393272
3222	Italy	1999	232.44743006061	0.746074084	2.11402418125879	230.333405879351
3223	Italy	2000	233.55998307575	0.749675986	2.11147359568194	231.448509480068
3224	Italy	2001	234.59498554345	0.753554839	2.11479976721592	232.480185776234
3225	Italy	2002	235.45287036701	0.757191479	2.11318906324724	233.339681303763

TableS2 Frontier analysis of Smoking-Related Low Back Pain among WCBA

	location	year	val	SDI	frontier	eff_diff
3226	Italy	2003	236.40034402327	0.760093744	2.11386643171483	234.286477591555
3227	Italy	2004	237.22478781919	0.762787749	2.11502965429057	235.109758164899
3228	Italy	2005	238.31905387775	0.765689914	2.11369144930397	236.205362428446
3229	Italy	2006	238.73387415622	0.768402408	2.11291564151424	236.620958514706
3230	Italy	2007	238.36614366569	0.77069838	2.11458516464026	236.25155850105
3231	Italy	2008	237.1320730671	0.77306847	2.11389004090143	235.018183026199
3232	Italy	2009	235.99704837518	0.775499262	2.11416768002611	233.882880695154
3233	Italy	2010	234.6605260626	0.778109206	2.11338988672962	232.54713617587
3234	Italy	2011	232.580806442	0.780703468	2.11499900811687	230.465807433883
3235	Italy	2012	229.61937568324	0.783090981	2.11475652198587	227.504619161254
3236	Italy	2013	226.24699179071	0.785356877	2.11179196960797	224.135199821102
3237	Italy	2014	222.7407858764	0.787533956	2.11161754557766	220.629168330822
3238	Italy	2015	219.4375729542	0.789902119	2.11424059797977	217.32333235622
3239	Italy	2016	216.59376991495	0.792477244	2.11552058125538	214.478249333695
3240	Italy	2017	213.76038146637	0.795311186	2.11584963595716	211.644531830413
3241	Italy	2018	211.31852191097	0.79836269	2.11359988771721	209.204922023253
3242	Italy	2019	209.21687800383	0.801530021	2.113158818849	207.103719184981
3243	Italy	2020	208.66288211546	0.80363568	2.11641607668094	206.546466038779
3244	Italy	2021	207.50625690592	0.805773534	2.11146950557582	205.394787400344
3245	Jamaica	1990	49.22574330616	0.534781234	2.11083123080514	47.1149120753549
3246	Jamaica	1991	50.65084046337	0.53959448	2.11341655644083	48.5374239069292
3247	Jamaica	1992	51.99320833771	0.545088753	2.11348968668368	49.8797186510263
3248	Jamaica	1993	53.2527742317	0.551218492	2.11107813645325	51.1416960952467
3249	Jamaica	1994	54.49106007053	0.557643773	2.11343439994743	52.3776256705826
3250	Jamaica	1995	55.49986220279	0.564315038	2.11329148739215	53.3865707153978

TableS2 Frontier analysis of Smoking-Related Low Back Pain among WCBA

	location	year	val	SDI	frontier	eff_diff
3251	Jamaica	1996	56.24797730852	0.570940131	2.11144467837921	54.1365326301408
3252	Jamaica	1997	56.80103969218	0.577212091	2.1122730869715	54.6887666052085
3253	Jamaica	1998	57.11857320036	0.583048035	2.11515424867138	55.0034189516886
3254	Jamaica	1999	57.30302545571	0.588846467	2.11292243846309	55.1901030172469
3255	Jamaica	2000	57.46554062231	0.594592917	2.1127238221248	55.3528168001852
3256	Jamaica	2001	57.51856221824	0.600192022	2.11162419556646	55.4069380226735
3257	Jamaica	2002	57.54810131101	0.605591748	2.11903229958424	55.4290690114258
3258	Jamaica	2003	57.29546074768	0.610877424	2.11393239246545	55.1815283552145
3259	Jamaica	2004	56.81831574306	0.615836278	2.11107181456707	54.7072439284929
3260	Jamaica	2005	56.16795771949	0.620400057	2.11365941472153	54.0542983047685
3261	Jamaica	2006	55.25250865452	0.62501886	2.11245322233955	53.1400554321805
3262	Jamaica	2007	53.79379060045	0.62954947	2.11225829201542	51.6815323084346
3263	Jamaica	2008	52.22443357692	0.633967851	2.11478886077647	50.1096447161435
3264	Jamaica	2009	50.68998243684	0.638110835	2.11625177676	48.57373066008
3265	Jamaica	2010	49.35378141662	0.642129388	2.11493465970506	47.2388467569149
3266	Jamaica	2011	48.25044502238	0.64639583	2.11378672170089	46.1366583006791
3267	Jamaica	2012	47.18921263861	0.65058376	2.11234720453107	45.0768654340789
3268	Jamaica	2013	46.28602178626	0.654573624	2.11318831543468	44.1728334708253
3269	Jamaica	2014	45.64773049479	0.658505341	2.11278679455786	43.5349437002321
3270	Jamaica	2015	45.28765665656	0.662465809	2.11334928434809	43.1743073722119
3271	Jamaica	2016	45.09591216014	0.666469279	2.11143337387225	42.9844787862677
3272	Jamaica	2017	45.01612798473	0.670291045	2.11692283891893	42.8992051458111
3273	Jamaica	2018	45.0954621744	0.674075313	2.10959700938959	42.9858651650104
3274	Jamaica	2019	45.23255645323	0.677778539	2.1126125449943	43.1199439082357
3275	Jamaica	2020	45.53091203079	0.680567177	2.11484507094139	43.4160669598486

TableS2 Frontier analysis of Smoking-Related Low Back Pain among WCBA

	location	year	val	SDI	frontier	eff_diff
3276	Jamaica	2021	45.84650990034	0.683263064	2.11441310116206	43.7320967991779
3277	Japan	1990	193.30445360274	0.790253516	2.1180743293554	191.186379273385
3278	Japan	1991	191.95818422073	0.794616189	2.11351759015916	189.844666630571
3279	Japan	1992	191.54507640868	0.799156225	2.11372653480889	189.431349873871
3280	Japan	1993	191.89285237033	0.802972994	2.11511782411968	189.77773454621
3281	Japan	1994	192.75753044185	0.806689483	2.11436472896253	190.643165712887
3282	Japan	1995	195.47749819006	0.810510771	2.11576830740447	193.361729882656
3283	Japan	1996	197.66705951006	0.813870746	2.11255130023957	195.55450820982
3284	Japan	1997	199.93309544074	0.816675782	2.11411186042351	197.818983580316
3285	Japan	1998	202.06326932799	0.819016202	2.1125630945619	199.950706233428
3286	Japan	1999	203.92907186816	0.821066637	2.11508506806753	201.813986800092
3287	Japan	2000	205.75787599059	0.822938999	2.11453655025667	203.643339440333
3288	Japan	2001	208.10353155263	0.824723241	2.11387730308276	205.989654249547
3289	Japan	2002	210.86459413102	0.826936774	2.11314859223436	208.751445538786
3290	Japan	2003	214.25212468488	0.829392223	2.11110888450188	212.141015800378
3291	Japan	2004	217.57047338489	0.832037381	2.11313784123175	215.457335543658
3292	Japan	2005	220.08106019454	0.834288685	2.1127919643813	217.968268230159
3293	Japan	2006	220.48698411409	0.836222264	2.11406494570514	218.372919168385
3294	Japan	2007	218.23788062296	0.83829416	2.11315195235616	216.124728670604
3295	Japan	2008	214.39023740382	0.840301217	2.11384189768265	212.276395506137
3296	Japan	2009	209.95232788505	0.842133664	2.11348853443756	207.838839350612
3297	Japan	2010	206.46050464854	0.844361811	2.11300683616414	204.347497812376
3298	Japan	2011	203.23583688243	0.846750267	2.1117678661831	201.124069016247
3299	Japan	2012	199.74020109067	0.849061489	2.11128803568808	197.628913054982
3300	Japan	2013	196.10995216029	0.851356994	2.11445736420912	193.995494796081

TableS2 Frontier analysis of Smoking-Related Low Back Pain among WCBA

	location	year	val	SDI	frontier	eff_diff
3301	Japan	2014	192.84932507162	0.853692749	2.11319567834446	190.736129393276
3302	Japan	2015	189.81625954104	0.856097499	2.11187023428582	187.704389306754
3303	Japan	2016	188.20191497953	0.858698173	2.11343603504571	186.088478944484
3304	Japan	2017	186.25706534445	0.861393309	2.11660851600638	184.140456828444
3305	Japan	2018	184.37701250404	0.864237137	2.11673770865344	182.260274795387
3306	Japan	2019	182.01321231554	0.867148183	2.11372344636845	179.899488869172
3307	Japan	2020	172.77799395024	0.869106879	2.1143816295	170.66361232074
3308	Japan	2021	175.70873014633	0.871241813	2.11555417018254	173.593175976147
3309	Jordan	1990	61.47556118604	0.539147468	2.11489050068843	59.3606706853516
3310	Jordan	1991	60.89489286123	0.542819411	2.11283767839797	58.782055182832
3311	Jordan	1992	60.66145023884	0.547458846	2.11144383615602	58.550006402684
3312	Jordan	1993	60.63815826569	0.55218235	2.11087423495017	58.5272840307398
3313	Jordan	1994	60.75218992572	0.557254335	2.11295483744091	58.6392350882791
3314	Jordan	1995	61.34924661799	0.562571536	2.11425522389065	59.2349913940994
3315	Jordan	1996	61.88645383573	0.567529655	2.11745420773417	59.7689996279958
3316	Jordan	1997	62.55235943958	0.572581508	2.11660385440439	60.4357555851756
3317	Jordan	1998	63.21846907625	0.57764357	2.1125779159477	61.1058911603023
3318	Jordan	1999	64.08891615577	0.582759199	2.11585891071896	61.973057245051
3319	Jordan	2000	65.62502002125	0.587909394	2.11252756017265	63.5124924610774
3320	Jordan	2001	67.00490824531	0.593182803	2.11081396493305	64.894094280377
3321	Jordan	2002	68.61068836687	0.599000385	2.11707733356159	66.4936110333084
3322	Jordan	2003	70.40447938352	0.60494595	2.11150980935123	68.2929695741688
3323	Jordan	2004	72.06257964865	0.611934404	2.11262376753031	69.9499558811197
3324	Jordan	2005	73.6718770449	0.619909228	2.11253812583351	71.5593389190665
3325	Jordan	2006	74.49141435915	0.627803813	2.11561246393895	72.3758018952111

TableS2 Frontier analysis of Smoking-Related Low Back Pain among WCBA

	location	year	val	SDI	frontier	eff_diff
3326	Jordan	2007	74.8846258923	0.635670276	2.11130864490632	72.7733172473937
3327	Jordan	2008	76.2377087008	0.643892693	2.11260576849129	74.1251029323087
3328	Jordan	2009	78.32064445184	0.652196319	2.11338218513176	76.2072622667082
3329	Jordan	2010	80.16442797724	0.659949495	2.11316798547	78.05125999177
3330	Jordan	2011	81.2391934553	0.66739668	2.11425185107353	79.1249416042265
3331	Jordan	2012	81.80740513041	0.674568925	2.11619088306379	79.6912142473462
3332	Jordan	2013	81.58842383174	0.681119962	2.11426959080397	79.474154240936
3333	Jordan	2014	80.25185636425	0.68713404	2.1137697034109	78.1380866608391
3334	Jordan	2015	79.01174058522	0.692952032	2.11171247420636	76.9000281110136
3335	Jordan	2016	78.01184034938	0.698473302	2.11485657450286	75.8969837748771
3336	Jordan	2017	76.60208933503	0.703923826	2.11159781645649	74.4904915185735
3337	Jordan	2018	74.99593472164	0.709412879	2.11470496059339	72.8812297610466
3338	Jordan	2019	73.53491133402	0.714998946	2.11274660706569	71.4221647269543
3339	Jordan	2020	71.80852443972	0.72006824	2.11209278552083	69.6964316541992
3340	Jordan	2021	70.3055523294	0.725307227	2.11270926095818	68.1928430684418
3341	Kazakhstan	1990	53.72400764737	0.589435804	2.11549132060096	51.608516326769
3342	Kazakhstan	1991	53.16563910852	0.590967832	2.11498953933297	51.050649569187
3343	Kazakhstan	1992	53.03628929166	0.596127664	2.11245798318603	50.923831308474
3344	Kazakhstan	1993	53.3556138438	0.603138328	2.11190392881788	51.2437099149821
3345	Kazakhstan	1994	54.22796767244	0.6105911	2.1127807054945	52.1151869669455
3346	Kazakhstan	1995	55.27106458308	0.618573582	2.11427145172645	53.1567931313536
3347	Kazakhstan	1996	56.49622270888	0.626013138	2.11259054342167	54.3836321654583
3348	Kazakhstan	1997	57.85353089597	0.631166626	2.11400682821053	55.7395240677595
3349	Kazakhstan	1998	59.33715414624	0.635388624	2.11362900181916	57.2235251444208
3350	Kazakhstan	1999	60.89501287083	0.640180485	2.11247190515922	58.7825409656708

TableS2 Frontier analysis of Smoking-Related Low Back Pain among WCBA

	location	year	val	SDI	frontier	eff_diff
3351	Kazakhstan	2000	62.224390859	0.644689623	2.11282746861507	60.1115633903849
3352	Kazakhstan	2001	63.96759767867	0.648397258	2.11416656542228	61.8534311132477
3353	Kazakhstan	2002	66.28034707299	0.651529335	2.113120146258	64.167226926732
3354	Kazakhstan	2003	68.66986563453	0.654835926	2.11676089576773	66.5531047387623
3355	Kazakhstan	2004	70.6499112439	0.658733071	2.11308510869104	68.536826135209
3356	Kazakhstan	2005	71.74158426073	0.662943942	2.11417902469574	69.6274052360343
3357	Kazakhstan	2006	72.20846398721	0.667048858	2.11568427446789	70.0927797127421
3358	Kazakhstan	2007	72.73405207836	0.671367208	2.11528317662872	70.6187689017313
3359	Kazakhstan	2008	73.05440692282	0.676352869	2.11192861959353	70.9424783032265
3360	Kazakhstan	2009	73.26172588475	0.681606839	2.11581150674696	71.145914378003
3361	Kazakhstan	2010	73.23269146652	0.686996643	2.11380487587031	71.1188865906497
3362	Kazakhstan	2011	72.6255722671	0.691758952	2.11294223985029	70.5126300272497
3363	Kazakhstan	2012	71.22775907503	0.695312767	2.11690214917938	69.1108569258506
3364	Kazakhstan	2013	69.64622916621	0.699053593	2.11265531941625	67.5335738467937
3365	Kazakhstan	2014	68.35290092497	0.703511921	2.11343078241327	66.2394701425567
3366	Kazakhstan	2015	67.83999639669	0.708229736	2.1138742367225	65.7261221599675
3367	Kazakhstan	2016	68.55760755821	0.71256904	2.11281773190686	66.4447898263031
3368	Kazakhstan	2017	69.92917401238	0.716026688	2.1132541489605	67.8159198634195
3369	Kazakhstan	2018	71.5800523277	0.718531999	2.11459135995187	69.4654609677481
3370	Kazakhstan	2019	72.76776332697	0.720797602	2.1145166367573	70.6532466902127
3371	Kazakhstan	2020	73.15014785672	0.722788768	2.11138713521942	71.0387607215006
3372	Kazakhstan	2021	73.53754074094	0.725144495	2.11398212014603	71.423558620794
3373	Kenya	1990	9.5064175871	0.333850293	2.28710297203576	7.21931461506424
3374	Kenya	1991	9.71798214679	0.34047622	2.24134464252235	7.47663750426765
3375	Kenya	1992	9.911019139	0.346298636	2.13855706848924	7.77246207051076

TableS2 Frontier analysis of Smoking-Related Low Back Pain among WCBA

	location	year	val	SDI	frontier	eff_diff
3376	Kenya	1993	10.08590193927	0.351611769	2.11259837622383	7.97330356304617
3377	Kenya	1994	10.22940720973	0.356779194	2.11723451923	8.1121726905
3378	Kenya	1995	10.31912931679	0.361535807	2.1135930267215	8.2055362900685
3379	Kenya	1996	10.38872912078	0.366203076	2.11238199818532	8.27634712259468
3380	Kenya	1997	10.44153010727	0.370436465	2.11201980654318	8.32951030072682
3381	Kenya	1998	10.47171294166	0.374581401	2.11406119245929	8.35765174920071
3382	Kenya	1999	10.49227220807	0.378630009	2.11798292136469	8.37428928670531
3383	Kenya	2000	10.51385426301	0.382395616	2.11172777141512	8.40212649159488
3384	Kenya	2001	10.48365565469	0.386188415	2.11566696509549	8.36798868959451
3385	Kenya	2002	10.4163629902	0.389886256	2.11192310922987	8.30443988097013
3386	Kenya	2003	10.32865250465	0.39368048	2.11384930189025	8.21480320275975
3387	Kenya	2004	10.22927246555	0.397743651	2.11270795831424	8.11656450723576
3388	Kenya	2005	10.12485637673	0.402444011	2.11214968187419	8.01270669485581
3389	Kenya	2006	9.96924176979	0.407745764	2.11392715121296	7.85531461857704
3390	Kenya	2007	9.72805633709	0.413736227	2.11264178735831	7.61541454973169
3391	Kenya	2008	9.45019012095	0.419738147	2.11188435359629	7.33830576735372
3392	Kenya	2009	9.16286100973	0.426123888	2.11443092491975	7.04843008481025
3393	Kenya	2010	8.92361931405	0.433305839	2.11166244484072	6.81195686920927
3394	Kenya	2011	8.69153291077	0.44089383	2.11342083061247	6.57811208015753
3395	Kenya	2012	8.43248800697	0.448666396	2.11191283560597	6.32057517136403
3396	Kenya	2013	8.16337875224	0.456666942	2.11395033638767	6.04942841585233
3397	Kenya	2014	7.9099301568	0.464724302	2.11108188827986	5.79884826852014
3398	Kenya	2015	7.69898069154	0.473084051	2.11682476520778	5.58215592633222
3399	Kenya	2016	7.52576056611	0.481709072	2.11391541261633	5.41184515349367
3400	Kenya	2017	7.35602418997	0.490296619	2.11767102348108	5.23835316648892

TableS2 Frontier analysis of Smoking-Related Low Back Pain among WCBA

	location	year	val	SDI	frontier	eff_diff
3401	Kenya	2018	7.19472997402	0.499101274	2.11427024760825	5.08045972641175
3402	Kenya	2019	7.03899950265	0.508004211	2.11520792460411	4.92379157804589
3403	Kenya	2020	6.80963399009	0.516136572	2.11259361355861	4.69704037653139
3404	Kenya	2021	6.63934798142	0.523768077	2.11549761703844	4.52385036438156
3405	Kiribati	1990	112.08732013196	0.410389821	2.11226627505508	109.975053856905
3406	Kiribati	1991	115.05018972997	0.412884199	2.11869285616621	112.931496873804
3407	Kiribati	1992	118.22243044543	0.415577609	2.11199626299255	116.110434182437
3408	Kiribati	1993	121.36001515081	0.418481661	2.11154083167984	119.24847431913
3409	Kiribati	1994	124.44774047187	0.421716217	2.11428118555984	122.33345928631
3410	Kiribati	1995	127.86731827505	0.425024354	2.11226823752727	125.755050037523
3411	Kiribati	1996	132.74695201682	0.428300682	2.11359034574237	130.633361671078
3412	Kiribati	1997	138.0975494912	0.431641941	2.11382971048159	135.983719780718
3413	Kiribati	1998	143.39489367537	0.435741904	2.11592007462347	141.278973600747
3414	Kiribati	1999	148.29470281465	0.439692923	2.11043800272314	146.184264811927
3415	Kiribati	2000	150.4183645859	0.444436339	2.11240010021542	148.305964485685
3416	Kiribati	2001	150.88541455894	0.448782301	2.11544937942486	148.769965179515
3417	Kiribati	2002	150.7958962043	0.453462298	2.11651563934972	148.67938056495
3418	Kiribati	2003	150.56538295673	0.458208374	2.11591145236908	148.449471504361
3419	Kiribati	2004	150.12782317753	0.462336213	2.11236484763923	148.015458329891
3420	Kiribati	2005	149.57318113823	0.466722276	2.11418620335974	147.45899493487
3421	Kiribati	2006	148.57909085114	0.470557197	2.11570520560306	146.463385645537
3422	Kiribati	2007	146.47120138915	0.47422745	2.11256365666935	144.358637732481
3423	Kiribati	2008	143.90919881533	0.477201933	2.11425610656432	141.794942708766
3424	Kiribati	2009	140.93406216393	0.480035601	2.11958308706767	138.814479076862
3425	Kiribati	2010	138.67271195516	0.482447689	2.11418206414689	136.558529891013

TableS2 Frontier analysis of Smoking-Related Low Back Pain among WCBA

	location	year	val	SDI	frontier	eff_diff
3426	Kiribati	2011	136.17837465932	0.485028211	2.11210209938836	134.066272559932
3427	Kiribati	2012	134.09895908421	0.488175864	2.11503031754566	131.983928766664
3428	Kiribati	2013	131.70754180573	0.491880767	2.11442140895565	129.593120396774
3429	Kiribati	2014	129.86948135149	0.495317241	2.11310233396514	127.756379017525
3430	Kiribati	2015	128.23143084083	0.500059699	2.11418947180694	126.117241369023
3431	Kiribati	2016	127.13220141733	0.505207837	2.11426988865164	125.017931528678
3432	Kiribati	2017	126.26825788086	0.510019241	2.11295375247355	124.155304128386
3433	Kiribati	2018	125.9392094874	0.514810384	2.11420301363974	123.82500647376
3434	Kiribati	2019	126.23362163732	0.519441321	2.11371811774917	124.119903519571
3435	Kiribati	2020	126.27992365063	0.523454113	2.11358529578042	124.16633835485
3436	Kiribati	2021	127.91438509291	0.527186583	2.11600942511456	125.798375667795
3437	Kuwait	1990	33.91923952691	0.664517904	2.11811432682921	31.8011252000808
3438	Kuwait	1991	33.72473543013	0.668809621	2.1120810082744	31.6126544218556
3439	Kuwait	1992	34.15166421272	0.67015911	2.11354082806922	32.0381233846508
3440	Kuwait	1993	34.51483901764	0.670036252	2.11359685486711	32.4012421627729
3441	Kuwait	1994	34.98777525423	0.671393213	2.11255570777329	32.8752195464567
3442	Kuwait	1995	35.46403128521	0.677215523	2.11253497146603	33.351496313744
3443	Kuwait	1996	35.7784854812	0.686093304	2.11295747794094	33.6655280032591
3444	Kuwait	1997	35.61104204241	0.695789389	2.11331921367091	33.4977228287391
3445	Kuwait	1998	35.0934310552	0.704383736	2.11415176745722	32.9792792877428
3446	Kuwait	1999	34.78437414959	0.711841328	2.11219763969119	32.6721765098988
3447	Kuwait	2000	34.9129476659	0.718357562	2.11340181103537	32.7995458548646
3448	Kuwait	2001	34.95505424725	0.722840631	2.11078592125783	32.8442683259922
3449	Kuwait	2002	34.87183162174	0.726816976	2.11141099920215	32.7604206225378
3450	Kuwait	2003	34.92613658879	0.731778685	2.11318591164531	32.8129506771447

TableS2 Frontier analysis of Smoking-Related Low Back Pain among WCBA

	location	year	val	SDI	frontier	eff_diff
3451	Kuwait	2004	35.19356226351	0.738153707	2.1134549420023	33.0801073215077
3452	Kuwait	2005	35.49842758779	0.746384913	2.1182111310122	33.3802164567778
3453	Kuwait	2006	35.837819762	0.754928493	2.11582699303283	33.7219927689672
3454	Kuwait	2007	36.21694084288	0.762411484	2.11377062871027	34.1031702141697
3455	Kuwait	2008	36.41222158563	0.770002615	2.11376134033632	34.2984602452937
3456	Kuwait	2009	36.68994070827	0.777546551	2.11174176774089	34.5781989405291
3457	Kuwait	2010	37.01120891601	0.784873929	2.11524980981356	34.8959591061964
3458	Kuwait	2011	37.25962971012	0.791812065	2.11177158893014	35.1478581211899
3459	Kuwait	2012	37.44749520956	0.798356988	2.1152380127743	35.3322571967857
3460	Kuwait	2013	37.81294220207	0.804583834	2.11145986656787	35.7014823355021
3461	Kuwait	2014	38.3244704887	0.810743233	2.11113560301382	36.2133348856862
3462	Kuwait	2015	39.22311048304	0.816998413	2.1178830836739	37.1052273993661
3463	Kuwait	2016	40.36450280622	0.823026527	2.11435163885141	38.2501511673686
3464	Kuwait	2017	41.68497494283	0.828886987	2.114030570955	39.570944371875
3465	Kuwait	2018	42.94441650635	0.834371461	2.11224406489523	40.8321724414548
3466	Kuwait	2019	43.90855524325	0.839688307	2.1109737506657	41.7975814925843
3467	Kuwait	2020	44.78970027849	0.843459812	2.11510888612164	42.6745913923684
3468	Kuwait	2021	45.16116956598	0.846651055	2.11440854624549	43.0467610197345
3469	Kyrgyzstan	1990	27.3967927504	0.519407652	2.11366158429041	25.2831311661096
3470	Kyrgyzstan	1991	26.69135520274	0.523746714	2.114386782754	24.576968419986
3471	Kyrgyzstan	1992	26.45486253343	0.527901552	2.11472194967277	24.3401405837572
3472	Kyrgyzstan	1993	26.57099265377	0.532431008	2.112902421193	24.458090232577
3473	Kyrgyzstan	1994	27.06995583947	0.53623298	2.11388567375083	24.9560701657192
3474	Kyrgyzstan	1995	27.73807721867	0.53905282	2.110729583506	25.627347635164
3475	Kyrgyzstan	1996	28.55396357684	0.541628927	2.11345605651246	26.4405075203275

TableS2 Frontier analysis of Smoking-Related Low Back Pain among WCBA

	location	year	val	SDI	frontier	eff_diff
3476	Kyrgyzstan	1997	29.43720590521	0.54334115	2.11129583369371	27.3259100715163
3477	Kyrgyzstan	1998	30.51295380754	0.543258546	2.11572368795854	28.3972301195815
3478	Kyrgyzstan	1999	31.84077052187	0.542867282	2.11294843185824	29.7278220900118
3479	Kyrgyzstan	2000	32.9949081485	0.543036487	2.11135277850231	30.8835553699977
3480	Kyrgyzstan	2001	34.45275848645	0.543805551	2.11210773129842	32.3406507551516
3481	Kyrgyzstan	2002	36.49292818203	0.54437467	2.1156885994626	34.3772395825674
3482	Kyrgyzstan	2003	38.68032477296	0.545649262	2.11489178871794	36.5654329842421
3483	Kyrgyzstan	2004	40.83105629932	0.547262343	2.11367092590419	38.7173853734158
3484	Kyrgyzstan	2005	42.66412118614	0.548260449	2.11044441325507	40.5536767728849
3485	Kyrgyzstan	2006	44.46339477442	0.549383594	2.11154600657792	42.3518487678421
3486	Kyrgyzstan	2007	46.39016156583	0.551146192	2.11097641604607	44.2791851497839
3487	Kyrgyzstan	2008	48.01721763995	0.553206382	2.11134232671707	45.9058753132329
3488	Kyrgyzstan	2009	49.23624638171	0.555123866	2.11288783651121	47.1233585451988
3489	Kyrgyzstan	2010	49.61495419674	0.556416545	2.11238934111358	47.5025648556264
3490	Kyrgyzstan	2011	49.64895688551	0.558923335	2.11616004334886	47.5327968421611
3491	Kyrgyzstan	2012	49.68211136251	0.561541574	2.11168517406962	47.5704261884404
3492	Kyrgyzstan	2013	49.75625315182	0.565776769	2.11376880793388	47.6424843438861
3493	Kyrgyzstan	2014	50.00135204129	0.570569357	2.11252223768535	47.8888298036046
3494	Kyrgyzstan	2015	50.46704341133	0.575463231	2.11402473173333	48.3530186795967
3495	Kyrgyzstan	2016	50.97489891195	0.580388551	2.11703853499921	48.8578603769508
3496	Kyrgyzstan	2017	51.53046779502	0.585766096	2.11293322755031	49.4175345674697
3497	Kyrgyzstan	2018	52.11150847283	0.591237134	2.11417282619662	49.9973356466334
3498	Kyrgyzstan	2019	52.4833558979	0.596501638	2.1151789619094	50.3681769359906
3499	Kyrgyzstan	2020	52.79676810534	0.600499554	2.11511781001328	50.6816502953267
3500	Kyrgyzstan	2021	53.04101700172	0.603979328	2.11376693754726	50.9272500641727

TableS2 Frontier analysis of Smoking-Related Low Back Pain among WCBA

	location	year	val	SDI	frontier	eff_diff
3501	Laos	1990	20.82100212774	0.264283164	2.507436961215	18.313565166525
3502	Laos	1991	20.51119739435	0.268787618	2.4902642051423	18.0209331892077
3503	Laos	1992	20.29684664544	0.273435312	2.45741569326187	17.8394309521781
3504	Laos	1993	20.05971394	0.278140418	2.45434770802758	17.6053662319724
3505	Laos	1994	19.88678824008	0.28327023	2.436139369296	17.450648870784
3506	Laos	1995	19.84735241089	0.288795341	2.42570782093437	17.4216445899556
3507	Laos	1996	19.85344052656	0.294838372	2.40062940609873	17.4528111204613
3508	Laos	1997	19.89536490545	0.301477967	2.40037803539384	17.4949868700562
3509	Laos	1998	19.94619932029	0.308402545	2.38370599740729	17.5624933228827
3510	Laos	1999	20.00136281364	0.315972615	2.36505469013597	17.636308123504
3511	Laos	2000	19.93196231394	0.323947359	2.32317598517338	17.6087863287666
3512	Laos	2001	20.07958937089	0.332580409	2.30657562083055	17.7730137500595
3513	Laos	2002	20.25727785686	0.341667271	2.25096859640323	18.0063092604568
3514	Laos	2003	20.48407905229	0.350658178	2.12027919978817	18.3637998525018
3515	Laos	2004	20.71373638428	0.359681759	2.11773922035773	18.5959971639223
3516	Laos	2005	20.9575334412	0.368728678	2.11123048778	18.84630295342
3517	Laos	2006	21.25754460857	0.378122281	2.1147459551892	19.1427986533808
3518	Laos	2007	21.66807084811	0.387723649	2.11133294264067	19.5567379054693
3519	Laos	2008	22.10835255793	0.397011299	2.11362615656417	19.9947264013658
3520	Laos	2009	22.55396991517	0.406139022	2.11527600432394	20.4386939108461
3521	Laos	2010	22.92202862715	0.414865566	2.11035858944562	20.8116700377044
3522	Laos	2011	23.28588572408	0.423354523	2.11216716550954	21.1737185585705
3523	Laos	2012	23.68594235875	0.431149361	2.1127946778604	21.5731476808896
3524	Laos	2013	24.07929973062	0.438691039	2.11414410547033	21.9651556251497
3525	Laos	2014	24.45523116992	0.445869424	2.11360332109016	22.3416278488298

TableS2 Frontier analysis of Smoking-Related Low Back Pain among WCBA

	location	year	val	SDI	frontier	eff_diff
3526	Laos	2015	24.75978207917	0.452749603	2.11333451681274	22.6464475623573
3527	Laos	2016	25.27949288124	0.459583996	2.11216844714185	23.1673244340982
3528	Laos	2017	26.00730270882	0.466168892	2.11745963272288	23.8898430760971
3529	Laos	2018	26.76714107767	0.472618103	2.1122308547158	24.6549102229542
3530	Laos	2019	27.36273922895	0.478811373	2.11252877045969	25.2502104584903
3531	Laos	2020	27.5796759419	0.484192959	2.11246554590763	25.4672103959924
3532	Laos	2021	27.7282098213	0.489136091	2.11300115346696	25.615208667833
3533	Latvia	1990	209.17528733108	0.680193638	2.11563158270351	207.059655748376
3534	Latvia	1991	205.92261913146	0.688117297	2.11298448608851	203.809634645371
3535	Latvia	1992	203.05357999285	0.696157036	2.11387529495027	200.9397046979
3536	Latvia	1993	200.97325697427	0.705249855	2.11405033978	198.85920663449
3537	Latvia	1994	199.77308296949	0.712531353	2.1118936292788	197.661189340211
3538	Latvia	1995	199.48282486224	0.717639459	2.11344560278306	197.369379259457
3539	Latvia	1996	199.20390419122	0.721139538	2.11144435639795	197.092459834822
3540	Latvia	1997	198.61032032544	0.72374658	2.11444831417658	196.495872011263
3541	Latvia	1998	197.65175446952	0.725427336	2.11170822815391	195.540046241366
3542	Latvia	1999	196.32343814253	0.727219685	2.1167851804326	194.206652962097
3543	Latvia	2000	194.96104882541	0.730951449	2.11256381800086	192.848485007409
3544	Latvia	2001	194.62450521734	0.736294196	2.10874775201164	192.515757465328
3545	Latvia	2002	195.52707073056	0.74187044	2.11410246097708	193.412968269583
3546	Latvia	2003	196.19933394408	0.748168162	2.11077186439103	194.088562079689
3547	Latvia	2004	197.61978063212	0.755263211	2.11261885331524	195.507161778805
3548	Latvia	2005	198.94563888343	0.761929433	2.11347213995162	196.832166743478
3549	Latvia	2006	200.59865123683	0.768459929	2.11542954348151	198.483221693349
3550	Latvia	2007	202.86972498349	0.776481959	2.11157113413822	200.758153849352

TableS2 Frontier analysis of Smoking-Related Low Back Pain among WCBA

	location	year	val	SDI	frontier	eff_diff
3551	Latvia	2008	205.09097125664	0.786324573	2.11750770795868	202.973463548681
3552	Latvia	2009	207.7756403203	0.794504427	2.11427306770014	205.6613672526
3553	Latvia	2010	209.28424892388	0.798171594	2.11301877762676	207.171230146253
3554	Latvia	2011	210.30719560358	0.799080776	2.11617924004942	208.191016363531
3555	Latvia	2012	210.65101307555	0.801026474	2.11525676684193	208.535756308708
3556	Latvia	2013	211.19699216428	0.80266049	2.11265860350637	209.084333560774
3557	Latvia	2014	212.34623294921	0.803721486	2.11252259695449	210.233710352256
3558	Latvia	2015	213.39445515579	0.806985995	2.11288131178931	211.281573844001
3559	Latvia	2016	215.00665625413	0.811463591	2.11275678802985	212.8938994661
3560	Latvia	2017	217.17491880104	0.816923118	2.11361743143413	215.061301369606
3561	Latvia	2018	218.57696583922	0.821419809	2.11557501849594	216.461390820724
3562	Latvia	2019	219.57177072478	0.824781777	2.11491337862642	217.456857346154
3563	Latvia	2020	218.5136217155	0.827900448	2.1103887432131	216.403232972287
3564	Latvia	2021	216.8839904587	0.830663516	2.11353800259174	214.770452456108
3565	Lebanon	1990	131.51482372644	0.536718969	2.11175584049826	129.403067885942
3566	Lebanon	1991	134.91738520968	0.539197492	2.11622982542302	132.801155384257
3567	Lebanon	1992	138.50667837648	0.541793516	2.11074695580658	136.395931420673
3568	Lebanon	1993	142.02208984976	0.544581306	2.11801059650901	139.904079253251
3569	Lebanon	1994	146.01182704274	0.547684033	2.11209249848757	143.899734544252
3570	Lebanon	1995	150.32786739774	0.55147172	2.11292701494426	148.214940382796
3571	Lebanon	1996	154.94450307947	0.556436159	2.11368387035272	152.830819209117
3572	Lebanon	1997	160.11023726516	0.562120818	2.11508634118424	157.995150923976
3573	Lebanon	1998	165.30591126915	0.56905907	2.11291034466676	163.193000924483
3574	Lebanon	1999	169.95826048373	0.576078528	2.11654540238944	167.841715081341
3575	Lebanon	2000	173.27197146327	0.582698277	2.11230271831371	171.159668744956

TableS2 Frontier analysis of Smoking-Related Low Back Pain among WCBA

	location	year	val	SDI	frontier	eff_diff
3576	Lebanon	2001	174.92510588686	0.589185502	2.11252401557815	172.812581871282
3577	Lebanon	2002	176.69267744734	0.595978788	2.1150918240623	174.577585623278
3578	Lebanon	2003	178.37995190984	0.60296724	2.11153073295229	176.268421176888
3579	Lebanon	2004	180.05034610927	0.61084576	2.11326659262597	177.937079516644
3580	Lebanon	2005	181.45619650227	0.619391355	2.11535649213603	179.340840010134
3581	Lebanon	2006	182.58411884092	0.627791217	2.11080367806867	180.473315162851
3582	Lebanon	2007	183.99248417019	0.636938234	2.11250023557043	181.87998393462
3583	Lebanon	2008	185.08967414266	0.646667663	2.11588635252383	182.973787790136
3584	Lebanon	2009	185.41685893857	0.657433186	2.11297740673254	183.303881531837
3585	Lebanon	2010	185.75438653523	0.668804525	2.11340056707346	183.640985968157
3586	Lebanon	2011	185.42661489207	0.680920617	2.11386348789307	183.312751404177
3587	Lebanon	2012	183.68543325274	0.693910983	2.11325291797844	181.572180334762
3588	Lebanon	2013	178.09890496013	0.704811001	2.11510217208986	175.98380278804
3589	Lebanon	2014	169.06703905303	0.712423873	2.11412378937921	166.952915263651
3590	Lebanon	2015	164.25422590916	0.718697292	2.11444760788154	162.139778301278
3591	Lebanon	2016	165.92983509624	0.724583347	2.11333255951612	163.816502536724
3592	Lebanon	2017	169.6277162945	0.729963294	2.11136671214706	167.516349582353
3593	Lebanon	2018	172.83023694243	0.734746243	2.11619778606545	170.714039156365
3594	Lebanon	2019	176.3920566018	0.738644009	2.11453494326056	174.277521658539
3595	Lebanon	2020	181.33542852066	0.74200972	2.11351944902892	179.221909071631
3596	Lebanon	2021	184.22823627689	0.744746351	2.11386168190904	182.114374594981
3597	Lesotho	1990	4.78081065467	0.339155125	2.27380657288672	2.50700408178328
3598	Lesotho	1991	4.78684129174	0.345496571	2.13346918502756	2.65337210671244
3599	Lesotho	1992	4.78804920503	0.35216963	2.12030414546059	2.66774505956941
3600	Lesotho	1993	4.77909585251	0.358780457	2.11817321734677	2.66092263516323

TableS2 Frontier analysis of Smoking-Related Low Back Pain among WCBA

	location	year	val	SDI	frontier	eff_diff
3601	Lesotho	1994	4.76518654152	0.365310801	2.11527315285537	2.64991338866463
3602	Lesotho	1995	4.73720221624	0.371265315	2.1130633184193	2.6241388978207
3603	Lesotho	1996	4.71924559112	0.377221202	2.11334626293333	2.60589932818667
3604	Lesotho	1997	4.68212975355	0.383096648	2.11346184226103	2.56866791128897
3605	Lesotho	1998	4.63844113734	0.388406478	2.11348595352528	2.52495518381472
3606	Lesotho	1999	4.5903968789	0.393495001	2.11450610967526	2.47589076922474
3607	Lesotho	2000	4.52720036461	0.398635802	2.11477914078456	2.41242122382544
3608	Lesotho	2001	4.46755495317	0.403990126	2.11380368235076	2.35375127081924
3609	Lesotho	2002	4.41818337528	0.409021657	2.11304596675588	2.30513740852412
3610	Lesotho	2003	4.37312215079	0.413982571	2.11201645599314	2.26110569479686
3611	Lesotho	2004	4.31760300995	0.418670919	2.11291052477339	2.20469248517661
3612	Lesotho	2005	4.26213565783	0.423211228	2.11505237907833	2.14708327875167
3613	Lesotho	2006	4.18951661209	0.427806732	2.1114395523376	2.0780770597524
3614	Lesotho	2007	4.11025009636	0.432745038	2.1122957606824	1.9979543356776
3615	Lesotho	2008	4.02865388115	0.438340567	2.1152766434157	1.9133772377343
3616	Lesotho	2009	3.95089010276	0.443580127	2.1118690098925	1.8390210928675
3617	Lesotho	2010	3.90166299224	0.448966476	2.11411241199656	1.78755058024344
3618	Lesotho	2011	3.86850808368	0.454530098	2.11211635214024	1.75639173153976
3619	Lesotho	2012	3.82487371151	0.460419689	2.11165532616659	1.71321838534341
3620	Lesotho	2013	3.78280400565	0.466692256	2.11376025826157	1.66904374738843
3621	Lesotho	2014	3.75551508733	0.473188157	2.11654591514057	1.63896917218943
3622	Lesotho	2015	3.74269037455	0.479754456	2.11433994679493	1.62835042775507
3623	Lesotho	2016	3.75506177881	0.486144827	2.11629693049779	1.63876484831221
3624	Lesotho	2017	3.78755572266	0.491978155	2.11300321765514	1.67455250500486
3625	Lesotho	2018	3.82463606396	0.497381791	2.11421133993879	1.71042472402121

TableS2 Frontier analysis of Smoking-Related Low Back Pain among WCBA

	location	year	val	SDI	frontier	eff_diff
3626	Lesotho	2019	3.86569279279	0.502457317	2.11347988568045	1.75221290710955
3627	Lesotho	2020	3.89758214206	0.506746655	2.11827011410544	1.77931202795456
3628	Lesotho	2021	3.92669454063	0.510393066	2.11444853529627	1.81224600533373
3629	Liberia	1990	9.17442117288	0.235296847	2.62231398983056	6.55210718304945
3630	Liberia	1991	10.56169907414	0.238212218	2.60745322592263	7.95424584821737
3631	Liberia	1992	10.83997125073	0.238283313	2.72400659766364	8.11596465306636
3632	Liberia	1993	10.74815597421	0.235984406	2.58598447283671	8.16217150137329
3633	Liberia	1994	10.8996793137	0.232656774	2.65250441376551	8.24717489993449
3634	Liberia	1995	11.02193869869	0.228424478	2.79720299081527	8.22473570787473
3635	Liberia	1996	10.96390835351	0.222682591	2.69652363654158	8.26738471696842
3636	Liberia	1997	10.71521006936	0.219672765	2.92631757190037	7.78889249745963
3637	Liberia	1998	10.19012723031	0.219053855	2.93320925317847	7.25691797713153
3638	Liberia	1999	9.88885709948	0.222212867	2.89852370860701	6.99033339087299
3639	Liberia	2000	9.75359326002	0.231173362	2.65150322091379	7.10209003910621
3640	Liberia	2001	9.66172991841	0.239594386	2.58875482504509	7.07297509336491
3641	Liberia	2002	9.72438286244	0.248311971	2.58154907613886	7.14283378630114
3642	Liberia	2003	9.92502860367	0.251595458	2.5459383691854	7.3790902344846
3643	Liberia	2004	10.12423800754	0.25517961	2.50897225608918	7.61526575145082
3644	Liberia	2005	10.14903007577	0.25896799	2.50891756886872	7.64011250690128
3645	Liberia	2006	10.07924902864	0.262897258	2.48099570955183	7.59825331908817
3646	Liberia	2007	9.91005166508	0.267239568	2.48165356674417	7.42839809833583
3647	Liberia	2008	9.70571502248	0.271853596	2.48045938692983	7.22525563555017
3648	Liberia	2009	9.53821492707	0.276804111	2.45583964721238	7.08237527985762
3649	Liberia	2010	9.39702865469	0.282235752	2.4567406903615	6.9402879643285
3650	Liberia	2011	9.18358473892	0.288015145	2.44900017922446	6.73458455969554

TableS2 Frontier analysis of Smoking-Related Low Back Pain among WCBA

	location	year	val	SDI	frontier	eff_diff
3651	Liberia	2012	8.92192960243	0.294854315	2.39978848642329	6.52214111600671
3652	Liberia	2013	8.71259191287	0.302985656	2.3973889137325	6.3152029991375
3653	Liberia	2014	8.51427303723	0.31095544	2.36112439214291	6.15314864508709
3654	Liberia	2015	8.3900407126	0.318485166	2.34605503422182	6.04398567837818
3655	Liberia	2016	8.3443998111	0.325085174	2.32234709503567	6.02205271606433
3656	Liberia	2017	8.32589930728	0.331527786	2.30022203279667	6.02567727448333
3657	Liberia	2018	8.31283726017	0.337784578	2.27646494599606	6.03637231417394
3658	Liberia	2019	8.27141896001	0.343403262	2.21449367476775	6.05692528524225
3659	Liberia	2020	8.15854480052	0.34813389	2.1337019533052	6.0248428472148
3660	Liberia	2021	8.03252331511	0.352442452	2.11437557371492	5.91814774139508
3661	Libya	1990	6.9836276709	0.527998168	2.11357764277325	4.87005002812675
3662	Libya	1991	6.83445966855	0.541432399	2.11219228381819	4.7222673847318
3663	Libya	1992	6.68394426623	0.554468227	2.11611134602847	4.56783292020153
3664	Libya	1993	6.57334489393	0.566798415	2.11168747829701	4.46165741563299
3665	Libya	1994	6.48963083778	0.578651416	2.11360750603831	4.37602333174169
3666	Libya	1995	6.42041396607	0.589578634	2.11005337113871	4.31036059493128
3667	Libya	1996	6.35722455214	0.600350044	2.11620500366921	4.24101954847079
3668	Libya	1997	6.31906030986	0.610620315	2.11304538141411	4.20601492844589
3669	Libya	1998	6.2923304912	0.619973984	2.11526029613377	4.17707019506623
3670	Libya	1999	6.29886965075	0.628600604	2.11791969130186	4.18094995944814
3671	Libya	2000	6.3476909817	0.637047935	2.11350092216878	4.23419005953122
3672	Libya	2001	6.3797232744	0.644685131	2.112978991026	4.266744283374
3673	Libya	2002	6.41818530053	0.651669147	2.11264185660576	4.30554344392424
3674	Libya	2003	6.48266095636	0.659345125	2.11630735205481	4.36635360430519
3675	Libya	2004	6.56432025756	0.666913926	2.11204776131297	4.45227249624703

TableS2 Frontier analysis of Smoking-Related Low Back Pain among WCBA

	location	year	val	SDI	frontier	eff_diff
3676	Libya	2005	6.73157928345	0.675322268	2.11322827510101	4.61835100834899
3677	Libya	2006	6.93248376872	0.683702	2.11229303625221	4.82019073246779
3678	Libya	2007	7.14750709639	0.691855134	2.11164918679282	5.03585790959718
3679	Libya	2008	7.33376103469	0.698873253	2.11243082027566	5.22133021441434
3680	Libya	2009	7.50616819586	0.704998471	2.11389308939274	5.39227510646726
3681	Libya	2010	7.685153749	0.711362496	2.11052973825397	5.57462401074603
3682	Libya	2011	7.87563999889	0.711466657	2.11487781722339	5.76076218166661
3683	Libya	2012	8.09263540018	0.716700693	2.11345968298859	5.97917571719141
3684	Libya	2013	8.3194821336	0.71766525	2.11246055215217	6.20702158144783
3685	Libya	2014	8.52713335504	0.716042207	2.11363801122695	6.41349534381305
3686	Libya	2015	8.7377271807	0.713651367	2.11152254044574	6.62620464025426
3687	Libya	2016	8.9529959941	0.71076014	2.11576674404676	6.83722925005324
3688	Libya	2017	9.12892346237	0.710666264	2.11050944206848	7.01841402030152
3689	Libya	2018	9.29377315225	0.712720438	2.11358073591049	7.18019241633951
3690	Libya	2019	9.40963494415	0.716157239	2.11281984480352	7.29681509934648
3691	Libya	2020	9.43022466958	0.720270393	2.11566930951986	7.31455536006014
3692	Libya	2021	9.53732154388	0.725771399	2.11118798323839	7.42613356064161
3693	Lithuania	1990	165.60473809244	0.668503938	2.11232492978376	163.492413162656
3694	Lithuania	1991	163.48473223179	0.673234121	2.11408550249247	161.370646729298
3695	Lithuania	1992	161.74639665538	0.68099288	2.11517346520127	159.631223190179
3696	Lithuania	1993	160.06232396347	0.687480621	2.11468505442161	157.947638909048
3697	Lithuania	1994	159.64933925304	0.691151653	2.11440031464782	157.534938938392
3698	Lithuania	1995	160.75860807038	0.69473438	2.11178612789473	158.646821942485
3699	Lithuania	1996	162.02267013675	0.698591301	2.11340939358329	159.909260743167
3700	Lithuania	1997	164.22341477176	0.70284095	2.11259221098143	162.110822560779

TableS2 Frontier analysis of Smoking-Related Low Back Pain among WCBA

	location	year	val	SDI	frontier	eff_diff
3701	Lithuania	1998	166.12842105144	0.707468182	2.11358394053197	164.014837110908
3702	Lithuania	1999	167.91968176438	0.712834171	2.11269771909216	165.806984045288
3703	Lithuania	2000	169.49563435105	0.71989696	2.11274647854625	167.382887872504
3704	Lithuania	2001	171.73655087081	0.727133585	2.11261253704514	169.623938333765
3705	Lithuania	2002	175.37758191315	0.733793398	2.1150775403273	173.262504372823
3706	Lithuania	2003	179.5080650453	0.741229601	2.11145082005506	177.396614225245
3707	Lithuania	2004	183.90511709633	0.748949762	2.11391126914317	181.791205827187
3708	Lithuania	2005	187.51930994536	0.75635236	2.11398812107342	185.405321824287
3709	Lithuania	2006	190.82131845308	0.763059124	2.11249612553167	188.708822327548
3710	Lithuania	2007	194.14974811741	0.770229639	2.11227697199474	192.037471145415
3711	Lithuania	2008	196.64085496304	0.778273712	2.118092482596	194.522762480444
3712	Lithuania	2009	198.65686234573	0.785689739	2.11222610938077	196.544636236349
3713	Lithuania	2010	200.23354204591	0.793364682	2.11369378109835	198.119848264812
3714	Lithuania	2011	201.06008080205	0.800924994	2.11056588092595	198.949514921124
3715	Lithuania	2012	201.47152724813	0.807827026	2.11122676550035	199.36030048263
3716	Lithuania	2013	202.03572761691	0.813219722	2.1131510857681	199.922576531142
3717	Lithuania	2014	202.84456629537	0.817886376	2.1163337279845	200.728232567385
3718	Lithuania	2015	203.74192753912	0.823112629	2.11302898260737	201.628898556513
3719	Lithuania	2016	204.81064682988	0.829303695	2.11051532204344	202.700131507837
3720	Lithuania	2017	206.04535184633	0.83587174	2.11109821411953	203.93425363221
3721	Lithuania	2018	207.26571330654	0.84191891	2.11284953856356	205.152863767976
3722	Lithuania	2019	207.83101412426	0.847906339	2.11261521516424	205.718398909096
3723	Lithuania	2020	207.16631751603	0.852752388	2.11547491087551	205.050842605154
3724	Lithuania	2021	203.5066371628	0.856484049	2.11260508358886	201.394032079211
3725	Low SDI	1993	24.97826785452	NA	24.97826785452	0

TableS2 Frontier analysis of Smoking-Related Low Back Pain among WCBA

	location	year	val	SDI	frontier	eff_diff
3726	Low SDI	2009	20.05679953656	NA	20.05679953656	0
3727	Low SDI	2010	19.52161720555	NA	19.52161720555	0
3728	Low SDI	2012	18.46895973024	NA	18.46895973024	0
3729	Low SDI	2015	17.11027274141	NA	17.11027274141	0
3730	Low SDI	2016	16.7884102859	NA	16.7884102859	0
3731	Low-middle SDI	1992	28.75020326224	NA	28.75020326224	0
3732	Low-middle SDI	1996	27.69039102224	NA	27.69039102224	0
3733	Low-middle SDI	1998	26.37733009987	NA	26.37733009987	0
3734	Low-middle SDI	2003	24.51582318864	NA	24.51582318864	0
3735	Low-middle SDI	2004	24.23350127648	NA	24.23350127648	0
3736	Low-middle SDI	2008	23.20119078871	NA	23.20119078871	0
3737	Low-middle SDI	2010	22.63145995441	NA	22.63145995441	0
3738	Low-middle SDI	2011	22.35790201306	NA	22.35790201306	0
3739	Low-middle SDI	2016	21.25661493092	NA	21.25661493092	0
3740	Low-middle SDI	2017	21.00755944263	NA	21.00755944263	0
3741	Low-middle SDI	2019	20.61991641202	NA	20.61991641202	0
3742	Low-middle SDI	2020	20.59314668154	NA	20.59314668154	0
3743	Low-middle SDI	2021	20.51758321601	NA	20.51758321601	0
3744	Luxembourg	1990	264.27717830682	0.781051609	2.11575476062958	262.16142354619
3745	Luxembourg	1991	265.9798204434	0.785961442	2.1122169247179	263.867603518682
3746	Luxembourg	1992	267.55862529681	0.789053364	2.11414156846134	265.444483728349
3747	Luxembourg	1993	268.56257816391	0.792843836	2.11161675289871	266.450961411011
3748	Luxembourg	1994	270.1939363867	0.797824008	2.11516020273532	268.078776183965
3749	Luxembourg	1995	272.47177784439	0.80268247	2.11193949168479	270.359838352705
3750	Luxembourg	1996	274.87061134609	0.806516138	2.11424226892987	272.75636907716

TableS2 Frontier analysis of Smoking-Related Low Back Pain among WCBA

	location	year	val	SDI	frontier	eff_diff
3751	Luxembourg	1997	276.70417008755	0.810235288	2.11163257575287	274.592537511797
3752	Luxembourg	1998	277.08517113978	0.813889026	2.11375508871574	274.971416051064
3753	Luxembourg	1999	277.97303168581	0.817570995	2.11016441881985	275.86286726699
3754	Luxembourg	2000	279.12524311983	0.822006568	2.11479756210581	277.010445557724
3755	Luxembourg	2001	278.90914120946	0.825312816	2.11327986325961	276.7958613462
3756	Luxembourg	2002	278.4177005609	0.828223491	2.11345963467392	276.304240926226
3757	Luxembourg	2003	276.280417454	0.830983946	2.11159994783938	274.168817506161
3758	Luxembourg	2004	271.75707262949	0.833672567	2.11248563709091	269.644586992399
3759	Luxembourg	2005	267.93280698411	0.836675264	2.11554310294089	265.817263881169
3760	Luxembourg	2006	262.45473032992	0.840574453	2.11388419706321	260.340846132857
3761	Luxembourg	2007	255.32865573496	0.844707006	2.11397737561245	253.214678359348
3762	Luxembourg	2008	247.83458917102	0.847978883	2.11384965808056	245.720739512939
3763	Luxembourg	2009	241.40849201561	0.850688725	2.11243122314885	239.296060792461
3764	Luxembourg	2010	235.62837713004	0.853258277	2.11404960619215	233.514327523848
3765	Luxembourg	2011	229.40053255255	0.856216834	2.11343708661667	227.287095465933
3766	Luxembourg	2012	223.11561982972	0.859789031	2.11853179285098	220.997088036869
3767	Luxembourg	2013	217.83125108362	0.863368792	2.11186846654268	215.719382617077
3768	Luxembourg	2014	213.63901365303	0.866693556	2.11391711889825	211.525096534132
3769	Luxembourg	2015	210.54009909666	0.870112937	2.11112576203416	208.428973334626
3770	Luxembourg	2016	208.37751916862	0.87342088	2.11011415364633	206.267405014974
3771	Luxembourg	2017	206.66264519515	0.876347684	2.11172163279747	204.550923562353
3772	Luxembourg	2018	205.48993335052	0.878637895	2.1153419376596	203.37459141286
3773	Luxembourg	2019	205.66536034287	0.880593895	2.11034359064966	203.55501675222
3774	Luxembourg	2020	203.45248967256	0.882495316	2.11198761303652	201.340502059523
3775	Luxembourg	2021	205.55432228888	0.884428955	2.1106409707725	203.443681318107

TableS2 Frontier analysis of Smoking-Related Low Back Pain among WCBA

	location	year	val	SDI	frontier	eff_diff
3776	Madagascar	1990	17.38795548029	0.279889465	2.45589049563889	14.9320649846511
3777	Madagascar	1991	17.01613297695	0.280429589	2.45569204204806	14.5604409349019
3778	Madagascar	1992	16.62946318138	0.280501283	2.45271908794914	14.1767440934309
3779	Madagascar	1993	16.22424033035	0.280204299	2.45980255231708	13.7644377780329
3780	Madagascar	1994	15.81767195322	0.279615149	2.46215414699167	13.3555178062283
3781	Madagascar	1995	15.42387731897	0.279164003	2.45603690752615	12.9678404114438
3782	Madagascar	1996	14.96827631661	0.279066493	2.44441834617206	12.5238579704379
3783	Madagascar	1997	14.42684258671	0.27942619	2.45736812098667	11.9694744657233
3784	Madagascar	1998	13.84288287514	0.280317671	2.45935655527896	11.383526319861
3785	Madagascar	1999	13.31126622882	0.281571072	2.45863697326382	10.8526292555562
3786	Madagascar	2000	12.86186865245	0.283190721	2.45622940947529	10.4056392429747
3787	Madagascar	2001	12.43810758572	0.28566818	2.44015417173682	9.99795341398318
3788	Madagascar	2002	12.02618578173	0.287153149	2.43395890392593	9.59222687780407
3789	Madagascar	2003	11.62146823199	0.289675457	2.43221566835449	9.18925256363551
3790	Madagascar	2004	11.26549946228	0.292629885	2.42291146370016	8.84258799857984
3791	Madagascar	2005	10.94612517513	0.295919327	2.39842164655929	8.54770352857071
3792	Madagascar	2006	10.64373784875	0.299723392	2.40498697473188	8.23875087401812
3793	Madagascar	2007	10.3623486651	0.304026245	2.3859619462125	7.9763867188875
3794	Madagascar	2008	10.08503152024	0.308990265	2.38624187563742	7.69878964460258
3795	Madagascar	2009	9.85912378623	0.313450891	2.36349040741926	7.49563337881074
3796	Madagascar	2010	9.68952327104	0.318353184	2.34326499574857	7.34625827529143
3797	Madagascar	2011	9.56092843786	0.323801043	2.32205453771591	7.23887390014409
3798	Madagascar	2012	9.43689697732	0.329947541	2.30849999001955	7.12839698730046
3799	Madagascar	2013	9.31445898598	0.336519232	2.28858688917688	7.02587209680312
3800	Madagascar	2014	9.22600214775	0.343352611	2.21926789221534	7.00673425553466

TableS2 Frontier analysis of Smoking-Related Low Back Pain among WCBA

	location	year	val	SDI	frontier	eff_diff
3801	Madagascar	2015	9.18138688892	0.350443595	2.12627065539229	7.05511623352771
3802	Madagascar	2016	9.167988175	0.358026341	2.11442931460929	7.05355886039071
3803	Madagascar	2017	9.15519163404	0.366235587	2.11445930804411	7.04073232599589
3804	Madagascar	2018	9.17029726899	0.374969644	2.11312534510935	7.05717192388065
3805	Madagascar	2019	9.16753933728	0.384209196	2.11176483420141	7.05577450307859
3806	Madagascar	2020	9.1228040697	0.392778709	2.11358927443021	7.00921479526979
3807	Madagascar	2021	9.02353928132	0.400246943	2.1117401803877	6.91179910093229
3808	Malawi	1990	9.34830527992	0.204010245	3.50388147039791	5.84442380952209
3809	Malawi	1991	9.30537247229	0.206043786	3.49160167784236	5.81377079444764
3810	Malawi	1992	9.32003191659	0.206732359	3.4556345067677	5.8643974098223
3811	Malawi	1993	9.3709958408	0.208091205	3.48428377003702	5.88671207076298
3812	Malawi	1994	9.63086631166	0.209144566	3.50626736890862	6.12459894275139
3813	Malawi	1995	9.95323825705	0.211756232	3.46561365577146	6.48762460127854
3814	Malawi	1996	10.10474441832	0.215264475	3.48542891279485	6.61931550552515
3815	Malawi	1997	10.13169222601	0.219416252	2.90087720093204	7.23081502507796
3816	Malawi	1998	10.20826485336	0.22312233	2.66520774645938	7.54305710690062
3817	Malawi	1999	10.25157904241	0.227068368	2.72475268295174	7.52682635945826
3818	Malawi	2000	10.28451468117	0.23090081	2.56924996816369	7.71526471300631
3819	Malawi	2001	10.38918395921	0.234020507	2.56986880471625	7.81931515449375
3820	Malawi	2002	10.5099720417	0.237602155	2.61666925772282	7.89330278397718
3821	Malawi	2003	10.61545810516	0.241559228	2.54368685847873	8.07177124668127
3822	Malawi	2004	10.69481884595	0.24643454	2.5448393450511	8.1499795008989
3823	Malawi	2005	10.709356067	0.251672318	2.5131095793458	8.1962464876542
3824	Malawi	2006	10.658484775	0.257595649	2.52075731931051	8.13772745568949
3825	Malawi	2007	10.59193254406	0.265226928	2.4904985046455	8.1014340394145

TableS2 Frontier analysis of Smoking-Related Low Back Pain among WCBA

	location	year	val	SDI	frontier	eff_diff
3826	Malawi	2008	10.4871682318	0.273689856	2.45852715604324	8.02864107575676
3827	Malawi	2009	10.36929353973	0.282296509	2.4433226166659	7.9259709230641
3828	Malawi	2010	10.26236359359	0.29117962	2.43845686262	7.82390673097
3829	Malawi	2011	10.09718602114	0.300448509	2.40519686037059	7.69198916076941
3830	Malawi	2012	9.85835068384	0.309197624	2.38431223970724	7.47403844413276
3831	Malawi	2013	9.57704240885	0.317936213	2.34366194502835	7.23338046382165
3832	Malawi	2014	9.29724289352	0.327042426	2.31766649237387	6.97957640114613
3833	Malawi	2015	9.0776687029	0.336140138	2.28679600011828	6.79087270278172
3834	Malawi	2016	8.9567882187	0.344731542	2.17380769182242	6.78298052687758
3835	Malawi	2017	8.86632742136	0.353121067	2.12469829604322	6.74162912531678
3836	Malawi	2018	8.78832604533	0.36147041	2.11362126049698	6.67470478483302
3837	Malawi	2019	8.67318674499	0.370114605	2.11143013208527	6.56175661290473
3838	Malawi	2020	8.49221258526	0.37787902	2.11149537277387	6.38071721248613
3839	Malawi	2021	8.37236723455	0.384553634	2.1149272915631	6.25743994298691
3840	Malaysia	1990	12.97726969936	0.545799405	2.11193278208931	10.8653369172707
3841	Malaysia	1991	13.16968718728	0.551683548	2.11520430202471	11.0544828852553
3842	Malaysia	1992	13.35664181394	0.558186752	2.11544681966269	11.2411949942773
3843	Malaysia	1993	13.53241115931	0.565845656	2.11343030570864	11.4189808536014
3844	Malaysia	1994	13.65835491337	0.574380446	2.11196394569518	11.5463909676748
3845	Malaysia	1995	13.78546099568	0.583441103	2.11296362617393	11.6724973695061
3846	Malaysia	1996	13.85364351856	0.593152135	2.11533446578424	11.7383090527758
3847	Malaysia	1997	14.04567089878	0.603418105	2.11645979733925	11.9292111014408
3848	Malaysia	1998	14.21068257232	0.612645412	2.1117525042	12.09893006812
3849	Malaysia	1999	14.27831306852	0.621724901	2.11286865485756	12.1654444136624
3850	Malaysia	2000	14.33789573684	0.630523544	2.11494213869487	12.2229535981451

TableS2 Frontier analysis of Smoking-Related Low Back Pain among WCBA

	location	year	val	SDI	frontier	eff_diff
3851	Malaysia	2001	14.26340174497	0.638257477	2.1139446652761	12.1494570796939
3852	Malaysia	2002	14.14174087229	0.645044248	2.11419167089889	12.0275492013911
3853	Malaysia	2003	13.95840859277	0.650885678	2.113599904055	11.844808688715
3854	Malaysia	2004	13.79846860416	0.656243326	2.11338304702276	11.6850855571372
3855	Malaysia	2005	13.64674060894	0.661242241	2.11075578264703	11.535984826293
3856	Malaysia	2006	13.41642485951	0.665956287	2.11298184250443	11.3034430170056
3857	Malaysia	2007	13.05474117681	0.670843726	2.11479387949522	10.9399472973148
3858	Malaysia	2008	12.65530923009	0.67639991	2.11269429524987	10.5426149348401
3859	Malaysia	2009	12.25704355715	0.682070654	2.11328641095871	10.1437571461913
3860	Malaysia	2010	11.87991187381	0.688258622	2.11183675783831	9.76807511597169
3861	Malaysia	2011	11.54954064092	0.694083403	2.11396787315493	9.43557276776508
3862	Malaysia	2012	11.1789680354	0.699664734	2.11413161712203	9.06483641827797
3863	Malaysia	2013	10.83887086569	0.704930717	2.11242787832682	8.72644298736318
3864	Malaysia	2014	10.53459127285	0.709953756	2.11243627916437	8.42215499368562
3865	Malaysia	2015	10.33669022075	0.714659871	2.11442026057577	8.22226996017423
3866	Malaysia	2016	10.23317577553	0.719223425	2.11125341248855	8.12192236304145
3867	Malaysia	2017	10.24205963011	0.724235412	2.11223021000171	8.12982942010829
3868	Malaysia	2018	10.2926702675	0.729388078	2.11002317910771	8.18264708839229
3869	Malaysia	2019	10.32006509253	0.734587751	2.11197216411319	8.20809292841681
3870	Malaysia	2020	10.33048916046	0.738743993	2.11312672058822	8.21736243987178
3871	Malaysia	2021	10.26298019317	0.742523828	2.11334380776761	8.14963638540239
3872	Maldives	1990	21.64050592717	0.331601544	2.31033009489286	19.3301758322771
3873	Maldives	1991	21.32878511851	0.344398607	2.16872573369451	19.1600593848155
3874	Maldives	1992	21.0718627309	0.35825234	2.11499762644138	18.9568651044586
3875	Maldives	1993	20.88955638528	0.372806999	2.11584716917641	18.7737092161036

TableS2 Frontier analysis of Smoking-Related Low Back Pain among WCBA

	location	year	val	SDI	frontier	eff_diff
3876	Maldives	1994	20.86605824209	0.388081604	2.11179622676923	18.7542620153208
3877	Maldives	1995	20.91310484207	0.403945583	2.11525319614838	18.7978516459216
3878	Maldives	1996	21.00907849669	0.420519128	2.11363253087891	18.8954459658111
3879	Maldives	1997	21.0593510393	0.437436026	2.11531944435183	18.9440315949482
3880	Maldives	1998	21.07697126115	0.453973361	2.1138520593061	18.9631192018439
3881	Maldives	1999	20.9066954834	0.469338066	2.11761065510115	18.7890848282988
3882	Maldives	2000	21.12555732791	0.483495671	2.11825396358475	19.0073033643253
3883	Maldives	2001	21.2245400826	0.495923695	2.11541583829803	19.109124244302
3884	Maldives	2002	21.16374249309	0.50740486	2.11511611775429	19.0486263753357
3885	Maldives	2003	20.95383274897	0.518926864	2.11199724100305	18.8418355079669
3886	Maldives	2004	20.69677887334	0.529910191	2.11306909385032	18.5837097794897
3887	Maldives	2005	20.43268864619	0.538253332	2.11244747552543	18.3202411706646
3888	Maldives	2006	20.20382112278	0.547950412	2.11254424760727	18.0912768751727
3889	Maldives	2007	19.9974544321	0.557529247	2.11244754425522	17.8850068878448
3890	Maldives	2008	19.79551565282	0.566839634	2.11323766724229	17.6822779855777
3891	Maldives	2009	19.6833308481	0.57444206	2.11295392197043	17.5703769261296
3892	Maldives	2010	19.59941534331	0.581980986	2.11275359211613	17.4866617511939
3893	Maldives	2011	19.60494260289	0.58968446	2.1151950117848	17.4897475911052
3894	Maldives	2012	19.59803525753	0.596948519	2.11223632656797	17.485798930962
3895	Maldives	2013	19.73076464537	0.604238519	2.11427001547273	17.6164946298973
3896	Maldives	2014	19.97156610124	0.611578071	2.11477642587015	17.8567896753698
3897	Maldives	2015	20.29335009332	0.61845439	2.1115815502048	18.1817685431152
3898	Maldives	2016	20.77997228015	0.62487595	2.11546537862726	18.6645069015227
3899	Maldives	2017	21.36443810229	0.631083288	2.11170842730559	19.2527296749844
3900	Maldives	2018	21.97981463794	0.637205659	2.11153242557912	19.8682822123609

TableS2 Frontier analysis of Smoking-Related Low Back Pain among WCBA

	location	year	val	SDI	frontier	eff_diff
3901	Maldives	2019	22.58055290379	0.643082794	2.11262870019	20.4679242036
3902	Maldives	2020	23.13447981786	0.647608589	2.11272233809057	21.0217574797694
3903	Maldives	2021	23.73325266898	0.650886627	2.11402367039227	21.6192289985877
3904	Mali	1990	4.83691199411	0.126526428	4.79326593903585	0.0436460550741504
3905	Mali	1991	4.87246269786	0.12912815	4.77633254514152	0.0961301527184855
3906	Mali	1992	4.91051748122	0.131537449	4.78030317262849	0.130214308591508
3907	Mali	1993	4.94958563275	0.134177054	4.81785586587265	0.131729766877347
3908	Mali	1994	5.00009008253	0.136937829	4.70533170573937	0.294758376790625
3909	Mali	1995	5.0202312341	0.139866225	4.811752443989	0.208478790111
3910	Mali	1996	5.02685347362	0.143078916	4.69392509658116	0.332928377038841
3911	Mali	1997	5.07629463942	0.146414326	4.46484133910927	0.611453300310727
3912	Mali	1998	5.14935579523	0.14998673	4.54901198423707	0.600343810992931
3913	Mali	1999	5.19247058841	0.153737336	4.24931977343936	0.943150814970645
3914	Mali	2000	5.25002936602	0.157190028	4.09720973147313	1.15281963454687
3915	Mali	2001	5.30033995514	0.161344448	4.00747283747086	1.29286711766914
3916	Mali	2002	5.3691884078	0.165420696	3.86396642081656	1.50522198698344
3917	Mali	2003	5.42811813703	0.169733502	3.75934079880093	1.66877733822907
3918	Mali	2004	5.5033056892	0.173779552	3.6854671423038	1.8178385468962
3919	Mali	2005	5.57330467483	0.178029844	3.6545866467132	1.9187180281168
3920	Mali	2006	5.6594134764	0.182452468	3.64962843473357	2.00978504166643
3921	Mali	2007	5.78148225447	0.186912995	3.57721614707463	2.20426610739537
3922	Mali	2008	5.89352632675	0.191536746	3.5083591684296	2.3851671583204
3923	Mali	2009	5.97689242714	0.196537651	3.47258595140634	2.50430647573366
3924	Mali	2010	6.01451798835	0.201803536	3.50463864883197	2.50987933951803
3925	Mali	2011	6.01322089311	0.207097221	3.48475041396667	2.52847047914333

TableS2 Frontier analysis of Smoking-Related Low Back Pain among WCBA

	location	year	val	SDI	frontier	eff_diff
3926	Mali	2012	6.01660813139	0.21246506	3.48271618371881	2.53389194767119
3927	Mali	2013	6.01906807044	0.217993867	2.89675722531773	3.12231084512227
3928	Mali	2014	6.01852286225	0.22372998	2.73054910461305	3.28797375763695
3929	Mali	2015	5.9820771122	0.229826263	2.7422698198442	3.2398072923558
3930	Mali	2016	6.00022927854	0.236186231	2.62707602002474	3.37315325851526
3931	Mali	2017	6.00132128429	0.242726135	2.5426415335429	3.4586797507471
3932	Mali	2018	5.99473352642	0.249419731	2.56281427253789	3.43191925388211
3933	Mali	2019	5.99958080724	0.256250746	2.51066085558147	3.48891995165853
3934	Mali	2020	5.9905683299	0.262629219	2.47127322709802	3.51929510280198
3935	Mali	2021	5.95865229735	0.268579941	2.49119354314138	3.46745875420861
3936	Malta	1990	260.12032006349	0.656504584	2.11281175536481	258.007508308125
3937	Malta	1991	261.90463996614	0.661112691	2.11446516326768	259.790174802872
3938	Malta	1992	263.40378893678	0.666324648	2.113598106245	261.290190830535
3939	Malta	1993	262.625509351	0.672120257	2.11484066106197	260.510668689938
3940	Malta	1994	259.64675758226	0.677847314	2.11240209591905	257.534355486341
3941	Malta	1995	255.96716927089	0.682617704	2.1120804626871	253.855088808203
3942	Malta	1996	250.48229431537	0.686623261	2.1133120564047	248.368982258965
3943	Malta	1997	243.81419693379	0.691756406	2.11197464568076	241.702222288109
3944	Malta	1998	237.63721104901	0.698095287	2.11322080631113	235.523990242699
3945	Malta	1999	231.97826958985	0.704734545	2.11219693213513	229.866072657715
3946	Malta	2000	227.18306404353	0.711805254	2.11202013766409	225.071043905866
3947	Malta	2001	223.9928669223	0.717803038	2.11572512560607	221.877141796694
3948	Malta	2002	220.67342741038	0.722790275	2.11278191687914	218.560645493501
3949	Malta	2003	217.90726021692	0.727511133	2.11108122681513	215.796178990105
3950	Malta	2004	214.91599680343	0.731933853	2.11224169139936	212.803755112031

TableS2 Frontier analysis of Smoking-Related Low Back Pain among WCBA

	location	year	val	SDI	frontier	eff_diff
3951	Malta	2005	213.16914723722	0.736188747	2.11399927806985	211.05514795915
3952	Malta	2006	213.05859056815	0.740140181	2.11688869951615	210.941701868634
3953	Malta	2007	214.40821413727	0.744237812	2.11381833455013	212.29439580272
3954	Malta	2008	215.92998240956	0.748427095	2.113802516243	213.816179893317
3955	Malta	2009	218.35186514718	0.752171059	2.11435993194429	216.237505215236
3956	Malta	2010	220.22212624468	0.7562505	2.11343044369186	218.108695800988
3957	Malta	2011	222.00444620466	0.760310547	2.11324754266257	219.891198661997
3958	Malta	2012	224.15192962663	0.76453788	2.11496414852658	222.036965478103
3959	Malta	2013	226.46073496941	0.76861614	2.11452383357906	224.346211135831
3960	Malta	2014	228.58473016011	0.772728741	2.11303170679381	226.471698453316
3961	Malta	2015	230.46041079658	0.777276905	2.11130454891282	228.349106247667
3962	Malta	2016	232.37424793548	0.78167341	2.11425552781957	230.25999240766
3963	Malta	2017	235.03201048073	0.786410184	2.11261225999512	232.919398220735
3964	Malta	2018	238.22631194338	0.790965618	2.11568524066568	236.110626702714
3965	Malta	2019	241.51880199407	0.795197846	2.11533047035016	239.40347152372
3966	Malta	2020	241.48244813483	0.798375166	2.11120987151857	239.371238263311
3967	Malta	2021	244.71277432811	0.801585034	2.11409710899259	242.598677219117
3968	Marshall Islands	1990	35.49219976759	0.430839288	2.11819042269697	33.374009344893
3969	Marshall Islands	1991	35.87641302369	0.435571412	2.1115165436284	33.7648964800616
3970	Marshall Islands	1992	36.11732276325	0.440491018	2.11551360004661	34.0018091632034
3971	Marshall Islands	1993	36.32142040401	0.445479084	2.11355999985911	34.2078604041509
3972	Marshall Islands	1994	36.41706230791	0.450848221	2.11588201151721	34.3011802963928
3973	Marshall Islands	1995	36.38743020692	0.456859674	2.11130864490632	34.2761215620137
3974	Marshall Islands	1996	36.36763063453	0.46120943	2.11284420413559	34.2547864303944
3975	Marshall Islands	1997	36.33868775533	0.464752728	2.11508875571515	34.2235989996149

TableS2 Frontier analysis of Smoking-Related Low Back Pain among WCBA

	location	year	val	SDI	frontier	eff_diff
3976	Marshall Islands	1998	36.3580428582	0.468133032	2.11524675251639	34.2427961056836
3977	Marshall Islands	1999	36.55434691529	0.471292727	2.1117276754708	34.4426192398192
3978	Marshall Islands	2000	36.77817134143	0.474562536	2.11252772025781	34.6656436211722
3979	Marshall Islands	2001	36.93259688706	0.478588316	2.11561114653349	34.8169857405265
3980	Marshall Islands	2002	37.21024181507	0.482776259	2.11412971926333	35.0961120958067
3981	Marshall Islands	2003	37.61230190973	0.48624862	2.11033177799811	35.5019701317319
3982	Marshall Islands	2004	38.06456771571	0.489573077	2.11432958268108	35.9502381330289
3983	Marshall Islands	2005	38.5856347219	0.493271391	2.11333423210787	36.4723004897921
3984	Marshall Islands	2006	39.24034118138	0.497456175	2.11245347929667	37.1278877020833
3985	Marshall Islands	2007	39.91548849966	0.502250042	2.11360707524235	37.8018814244176
3986	Marshall Islands	2008	40.80941270752	0.506359588	2.11309724399317	38.6963154635268
3987	Marshall Islands	2009	41.66587728699	0.510794536	2.11098832520062	39.5548889617894
3988	Marshall Islands	2010	42.47099217183	0.516085138	2.11330335358206	40.3576888182479
3989	Marshall Islands	2011	43.50178738876	0.521177087	2.11456772689937	41.3872196618606
3990	Marshall Islands	2012	44.49512580304	0.526042019	2.11525598606098	42.379869816979
3991	Marshall Islands	2013	45.63088966555	0.5314463	2.11417665977045	43.5167130057795
3992	Marshall Islands	2014	46.53694808651	0.536640131	2.11289851848091	44.4240495680291
3993	Marshall Islands	2015	47.36939058331	0.541793167	2.11221758450257	45.2571729988074
3994	Marshall Islands	2016	47.96655457454	0.546782936	2.11116326938457	45.8553913051554
3995	Marshall Islands	2017	48.54266080018	0.552065152	2.11257673261478	46.4300840675652
3996	Marshall Islands	2018	49.03665452088	0.557599278	2.115196516027	46.921458004853
3997	Marshall Islands	2019	49.34603035933	0.56342875	2.1138560307739	47.2321743285561
3998	Marshall Islands	2020	49.24641330712	0.568837509	2.11119765918623	47.1352156479338
3999	Marshall Islands	2021	49.71760856713	0.574091128	2.11208765512974	47.6055209120003
4000	Mauritania	1990	31.53592823727	0.335780942	2.28959920724953	29.2463290300205

TableS2 Frontier analysis of Smoking-Related Low Back Pain among WCBA

	location	year	val	SDI	frontier	eff_diff
4001	Mauritania	1991	31.61991910373	0.341135175	2.24809746377134	29.3718216399587
4002	Mauritania	1992	31.68358058258	0.346531288	2.14221426091215	29.5413663216678
4003	Mauritania	1993	31.70534669573	0.352176572	2.12166429670718	29.5836823990228
4004	Mauritania	1994	31.69068191891	0.357585912	2.12420978316071	29.5664721357493
4005	Mauritania	1995	31.69234916987	0.363037695	2.11125520859141	29.5810939612786
4006	Mauritania	1996	31.68913476694	0.368508328	2.11266646171838	29.5764683052216
4007	Mauritania	1997	31.62317782029	0.373021339	2.11362908057043	29.5095487397196
4008	Mauritania	1998	31.4943643632	0.37708025	2.11051831803765	29.3838460451624
4009	Mauritania	1999	31.43045965913	0.381112297	2.11443406653641	29.3160255925936
4010	Mauritania	2000	31.60976669992	0.38420538	2.11367944754408	29.4960872523759
4011	Mauritania	2001	31.74365154946	0.386487416	2.11406699801325	29.6295845514468
4012	Mauritania	2002	31.99709818501	0.388564478	2.11161655405435	29.8854816309557
4013	Mauritania	2003	32.27027547525	0.391104879	2.11477023539387	30.1555052398561
4014	Mauritania	2004	32.46504509081	0.394012934	2.11238237893932	30.3526627118707
4015	Mauritania	2005	32.58190501621	0.397499105	2.11260303307591	30.4693019831341
4016	Mauritania	2006	32.52713313539	0.403159467	2.11509891564222	30.4120342197478
4017	Mauritania	2007	32.25519063476	0.408377122	2.11443180821198	30.140758826548
4018	Mauritania	2008	32.007101592	0.413233621	2.11592127363568	29.8911803183643
4019	Mauritania	2009	31.72581304362	0.417910609	2.11462669589627	29.6111863477237
4020	Mauritania	2010	31.46523632624	0.423202436	2.11372477327947	29.3515115529605
4021	Mauritania	2011	30.87303403291	0.42886729	2.11317030279302	28.759863730117
4022	Mauritania	2012	30.12224217159	0.434724376	2.11193800627551	28.0103041653145
4023	Mauritania	2013	29.20905579439	0.44106891	2.11484920706875	27.0942065873212
4024	Mauritania	2014	28.44067966701	0.447653881	2.11410992999011	26.3265697370199
4025	Mauritania	2015	27.78897845598	0.454523488	2.11424263012586	25.6747358258541

TableS2 Frontier analysis of Smoking-Related Low Back Pain among WCBA

	location	year	val	SDI	frontier	eff_diff
4026	Mauritania	2016	27.32301349257	0.461269545	2.11592409613317	25.2070893964368
4027	Mauritania	2017	26.8847076704	0.468452365	2.11727223489722	24.7674354355028
4028	Mauritania	2018	26.54340438992	0.475839507	2.11395661459814	24.4294477753219
4029	Mauritania	2019	26.21579952367	0.483767738	2.11384317741493	24.1019563462551
4030	Mauritania	2020	25.77321996198	0.491365603	2.11570721094774	23.6575127510323
4031	Mauritania	2021	25.33641190452	0.4989451	2.11204599442712	23.2243659100929
4032	Mauritius	1990	26.65050933215	0.544586533	2.11254083518014	24.5379684969699
4033	Mauritius	1991	26.9145814207	0.548209121	2.11277099700417	24.8018104236958
4034	Mauritius	1992	27.16755770589	0.55345463	2.11267829347282	25.0548794124172
4035	Mauritius	1993	27.21733750049	0.56068864	2.11461874424148	25.1027187562485
4036	Mauritius	1994	27.21824853857	0.569092171	2.11329164076923	25.1049568978008
4037	Mauritius	1995	27.12989751281	0.57731939	2.11595374162787	25.0139437711821
4038	Mauritius	1996	27.05859735409	0.584797061	2.11552706637025	24.9430702877198
4039	Mauritius	1997	26.67743009484	0.591497735	2.11424556603453	24.5631845288055
4040	Mauritius	1998	26.37453948027	0.597363039	2.114010139283	24.260529340987
4041	Mauritius	1999	26.09587651122	0.602034234	2.11279908108524	23.9830774301348
4042	Mauritius	2000	25.92831516264	0.607087901	2.11505714570918	23.8132580169308
4043	Mauritius	2001	25.67318239021	0.612240817	2.11290467901426	23.5602777111957
4044	Mauritius	2002	25.4850920642	0.617190007	2.11346019695492	23.3716318672451
4045	Mauritius	2003	25.33421076934	0.622764533	2.11104721628016	23.2231635530598
4046	Mauritius	2004	25.24770317656	0.629030193	2.11402904935058	23.1336741272094
4047	Mauritius	2005	25.31087695978	0.63476658	2.1174330864723	23.1934438733077
4048	Mauritius	2006	25.57084783309	0.639545183	2.11438266109957	23.4564651719904
4049	Mauritius	2007	25.95685823739	0.644358656	2.11382000027032	23.8430382371197
4050	Mauritius	2008	26.48871432657	0.649630852	2.11045955347967	24.3782547730903

TableS2 Frontier analysis of Smoking-Related Low Back Pain among WCBA

	location	year	val	SDI	frontier	eff_diff
4051	Mauritius	2009	27.05523616127	0.655000295	2.115880563953	24.939355597317
4052	Mauritius	2010	27.49869308259	0.6604698	2.11130439104735	25.3873886915427
4053	Mauritius	2011	27.8702975848	0.666497627	2.11313491678946	25.7571626680105
4054	Mauritius	2012	28.15231684028	0.67312765	2.11195629114195	26.0403605491381
4055	Mauritius	2013	28.29714851932	0.680114023	2.11413278124662	26.1830157380734
4056	Mauritius	2014	28.36808462061	0.686616503	2.11570998978593	26.2523746308241
4057	Mauritius	2015	28.41192628488	0.69223973	2.11726932280764	26.2946569620724
4058	Mauritius	2016	28.20828726768	0.697020562	2.11133569373841	26.0969515739416
4059	Mauritius	2017	27.85491506998	0.701618136	2.11257717356871	25.7423378964113
4060	Mauritius	2018	27.53833799885	0.706266651	2.11273699608585	25.4256010027642
4061	Mauritius	2019	27.46663478989	0.710935045	2.11251952000275	25.3541152698873
4062	Mauritius	2020	27.74966288292	0.714629804	2.11756601998754	25.6320968629325
4063	Mauritius	2021	27.94678018279	0.718260446	2.11413452726064	25.8326456555294
4064	Mexico	1990	74.43478785617	0.504996083	2.112234868607	72.322552987563
4065	Mexico	1991	73.1808463327	0.50951067	2.11222374154012	71.0686225911599
4066	Mexico	1992	71.91980542821	0.514638057	2.11356325241133	69.8062421757987
4067	Mexico	1993	70.7862837505	0.520493167	2.11361365123466	68.6726700992653
4068	Mexico	1994	69.74794829507	0.526749241	2.11240240542671	67.6355458896433
4069	Mexico	1995	68.81843303014	0.531554141	2.11173302500413	66.7067000051359
4070	Mexico	1996	67.68319687529	0.536530827	2.11341938463717	65.5697774906528
4071	Mexico	1997	66.36411787266	0.541988171	2.11603634892942	64.2480815237306
4072	Mexico	1998	64.834168176	0.547842121	2.112756318131	62.721411857869
4073	Mexico	1999	63.29934210767	0.55381337	2.11339869446627	61.1859434132037
4074	Mexico	2000	61.83016561732	0.559532547	2.11408554728481	59.7160800700352
4075	Mexico	2001	60.01760273202	0.564774176	2.1115864156808	57.9060163163392

TableS2 Frontier analysis of Smoking-Related Low Back Pain among WCBA

	location	year	val	SDI	frontier	eff_diff
4076	Mexico	2002	57.89107889364	0.570113531	2.11387515718756	55.7772037364524
4077	Mexico	2003	55.60516170244	0.575698215	2.11352829530451	53.4916334071355
4078	Mexico	2004	53.35576871782	0.580984982	2.11260804070667	51.2431606771133
4079	Mexico	2005	51.31705330982	0.58522408	2.11216886836544	49.2048844414546
4080	Mexico	2006	49.21798686115	0.588543226	2.11272492270194	47.1052619384481
4081	Mexico	2007	46.84727913149	0.591447408	2.11324965459091	44.7340294768991
4082	Mexico	2008	44.57626214059	0.594454558	2.1158601132878	42.4604020273022
4083	Mexico	2009	42.70441543358	0.596684358	2.11295383022952	40.5914616033505
4084	Mexico	2010	41.54728376538	0.599527028	2.11378228179282	39.4335014835872
4085	Mexico	2011	41.01042790779	0.603646095	2.11392934811944	38.8964985596706
4086	Mexico	2012	40.7014388789	0.608914289	2.11384395495031	38.5875949239497
4087	Mexico	2013	40.55337585099	0.615089993	2.11593981412576	38.4374360368642
4088	Mexico	2014	40.55737603034	0.621857405	2.11273042355208	38.4446456067879
4089	Mexico	2015	40.71207790118	0.628896724	2.11647798273926	38.5955999184407
4090	Mexico	2016	41.30765722082	0.635925349	2.11277346899196	39.194883751828
4091	Mexico	2017	42.39915974071	0.642667198	2.11035680816833	40.2888029325417
4092	Mexico	2018	43.45680361621	0.649053317	2.11336369951143	41.3434399166986
4093	Mexico	2019	44.03483430486	0.655095338	2.111727556858	41.923106748002
4094	Mexico	2020	43.71878160048	0.660119062	2.11069932298753	41.6080822774925
4095	Mexico	2021	43.74884043524	0.664575304	2.11419342782778	41.6346470074122
4096	Micronesia	1990	93.70926909979	0.462511617	2.11015052967377	91.5991185701162
4097	Micronesia	1991	94.97227052743	0.468144831	2.11701412955037	92.8552563978796
4098	Micronesia	1992	96.42846847707	0.473792936	2.11266905798067	94.3157994190893
4099	Micronesia	1993	97.61698112695	0.479939595	2.11437902083613	95.5026021061139
4100	Micronesia	1994	99.01618614891	0.485300006	2.1157310574473	96.9004550914627

TableS2 Frontier analysis of Smoking-Related Low Back Pain among WCBA

	location	year	val	SDI	frontier	eff_diff
4101	Micronesia	1995	99.92445031621	0.491015185	2.11510593522892	97.8093443809811
4102	Micronesia	1996	101.2315525182	0.495570308	2.11477408373813	99.1167784344619
4103	Micronesia	1997	102.42344592887	0.499110106	2.11164147790587	100.311804450964
4104	Micronesia	1998	103.72030753875	0.502861564	2.11554778329606	101.604759755454
4105	Micronesia	1999	105.15970463238	0.506663431	2.1200873807881	103.039617251592
4106	Micronesia	2000	107.24200657785	0.511004228	2.11495511915	105.1270514587
4107	Micronesia	2001	107.72248522656	0.515316814	2.11140121558778	105.611084010972
4108	Micronesia	2002	108.71207152578	0.519545672	2.11520727150559	106.596864254274
4109	Micronesia	2003	110.13573959231	0.523829599	2.11291934629116	108.022820246019
4110	Micronesia	2004	111.50354212298	0.527610965	2.11477839193597	109.388763731044
4111	Micronesia	2005	112.77721139299	0.531703321	2.11292064530838	110.664290747682
4112	Micronesia	2006	113.74854644492	0.535718682	2.11074401408787	111.637802430832
4113	Micronesia	2007	114.48003313048	0.539431464	2.115866922264	112.364166208216
4114	Micronesia	2008	115.09841593724	0.542766354	2.11219150126701	112.986224435973
4115	Micronesia	2009	115.32482172548	0.546275496	2.11555553119819	113.209266194282
4116	Micronesia	2010	115.59289518474	0.550122546	2.11472150515062	113.478173679589
4117	Micronesia	2011	115.80974241559	0.554240411	2.11227311943952	113.69746929615
4118	Micronesia	2012	116.09704208998	0.557965853	2.11111403291861	113.985928057061
4119	Micronesia	2013	116.26028304692	0.561082821	2.11420113653054	114.146081910389
4120	Micronesia	2014	116.62637179836	0.56384003	2.1129054643175	114.513466334042
4121	Micronesia	2015	116.93698690972	0.567151342	2.11261436777807	114.824372541942
4122	Micronesia	2016	116.7268792856	0.570458101	2.11364504370589	114.613234241894
4123	Micronesia	2017	116.35182930619	0.574039833	2.11336976963914	114.238459536551
4124	Micronesia	2018	115.95778563795	0.577555081	2.11200589853282	113.845779739417
4125	Micronesia	2019	115.64419997719	0.581195255	2.11378611782684	113.530413859363

TableS2 Frontier analysis of Smoking-Related Low Back Pain among WCBA

	location	year	val	SDI	frontier	eff_diff
4126	Micronesia	2020	114.6913768287	0.584527229	2.11315496684819	112.578221861852
4127	Micronesia	2021	115.17548477511	0.587534967	2.11224882927474	113.063235945835
4128	Middle SDI	1995	34.4963827516	NA	34.4963827516	0
4129	Middle SDI	1999	34.05568446973	NA	34.05568446973	0
4130	Middle SDI	2002	32.83547129182	NA	32.83547129182	0
4131	Middle SDI	2005	31.00032910628	NA	31.00032910628	0
4132	Middle SDI	2015	26.66881832486	NA	26.66881832486	0
4133	Middle SDI	2018	26.65578691021	NA	26.65578691021	0
4134	Middle SDI	2020	26.6691662276	NA	26.6691662276	0
4135	Moldova	1990	65.50667030645	0.604251762	2.11409409922776	63.3925762072222
4136	Moldova	1991	65.69733489123	0.607270774	2.11389350203304	63.583441389197
4137	Moldova	1992	66.00312192114	0.609664558	2.1136091626543	63.8895127584857
4138	Moldova	1993	66.52791105023	0.61415292	2.11331790142131	64.4145931488087
4139	Moldova	1994	67.74627933144	0.617603767	2.11307906783843	65.6332002636016
4140	Moldova	1995	69.36305404623	0.620951321	2.10979521967234	67.2532588265577
4141	Moldova	1996	71.41926612603	0.623113984	2.11190182346567	69.3073643025643
4142	Moldova	1997	72.66066401119	0.624370105	2.1143342737104	70.5463297374796
4143	Moldova	1998	74.07402535742	0.624116761	2.11759633800818	71.9564290194118
4144	Moldova	1999	74.43901474796	0.622834529	2.1122350628269	72.3267796851331
4145	Moldova	2000	74.35462644997	0.622004934	2.11503342262961	72.2395930273404
4146	Moldova	2001	74.49160563108	0.62293641	2.11436945025411	72.3772361808259
4147	Moldova	2002	75.13536075526	0.625874845	2.11577194335658	73.0195888119034
4148	Moldova	2003	76.20332038844	0.629985045	2.11242143726318	74.0908989511768
4149	Moldova	2004	77.16835566223	0.635394822	2.1126970334269	75.0556586288031
4150	Moldova	2005	77.91580284796	0.641530965	2.11371100044383	75.8020918475162

TableS2 Frontier analysis of Smoking-Related Low Back Pain among WCBA

	location	year	val	SDI	frontier	eff_diff
4151	Moldova	2006	78.52367461298	0.647996264	2.11559270143391	76.4080819115461
4152	Moldova	2007	79.02380294716	0.654215851	2.11175611740654	76.9120468297535
4153	Moldova	2008	79.5256367572	0.661046597	2.1134308910007	77.4122058661993
4154	Moldova	2009	79.80015558152	0.666284394	2.11289978017389	77.6872558013461
4155	Moldova	2010	80.37548694254	0.671989616	2.11258110166953	78.2629058408705
4156	Moldova	2011	81.07174569798	0.678004137	2.11291547092019	78.9588302270598
4157	Moldova	2012	81.94763709244	0.682979796	2.11081510701654	79.8368219854235
4158	Moldova	2013	83.06484863902	0.688519175	2.11341620963444	80.9514324293855
4159	Moldova	2014	84.48072904576	0.694104557	2.11309694452557	82.3676321012344
4160	Moldova	2015	86.21200912023	0.699355166	2.11039537121296	84.101613749017
4161	Moldova	2016	88.21538718027	0.705144542	2.11207268862722	86.1033144916428
4162	Moldova	2017	90.26354946008	0.711206637	2.1117400344156	88.1518094256644
4163	Moldova	2018	92.13253462945	0.717244427	2.11199112657697	90.020543502873
4164	Moldova	2019	94.18462607097	0.723011648	2.1127814876115	92.0718445833585
4165	Moldova	2020	95.86369134366	0.72771054	2.11484571188282	93.7488456317772
4166	Moldova	2021	97.07859517424	0.732214875	2.11449532190986	94.9640998523302
4167	Monaco	1990	291.60412078873	0.845495153	2.112465159127	289.491655629603
4168	Monaco	1991	293.03055822247	0.84830369	2.11575594614827	290.914802276322
4169	Monaco	1992	293.51056652108	0.85086394	2.11170054921397	291.398865971866
4170	Monaco	1993	293.35438282411	0.853264992	2.11206516857254	291.242317655537
4171	Monaco	1994	293.71696231629	0.855626927	2.1138694699369	291.603092846353
4172	Monaco	1995	293.11410457174	0.857986147	2.11146005151541	291.002644520225
4173	Monaco	1996	293.7375264479	0.860245432	2.112603307775	291.624923140125
4174	Monaco	1997	294.24750257845	0.86244672	2.11486776746027	292.13263481099
4175	Monaco	1998	294.30729699875	0.864716513	2.11130165434541	292.195995344405

TableS2 Frontier analysis of Smoking-Related Low Back Pain among WCBA

	location	year	val	SDI	frontier	eff_diff
4176	Monaco	1999	293.68905479312	0.866842394	2.11308616165987	291.57596863146
4177	Monaco	2000	293.90175096745	0.868973583	2.11311442919933	291.788636538251
4178	Monaco	2001	294.5328845434	0.871094755	2.11195369671321	292.420930846687
4179	Monaco	2002	293.71008908321	0.873182769	2.11241729588431	291.597671787326
4180	Monaco	2003	292.35252529827	0.875197652	2.11401055141159	290.238514746858
4181	Monaco	2004	290.40355187477	0.877231549	2.11209731952218	288.291454555248
4182	Monaco	2005	288.33466292576	0.879288089	2.11125800194604	286.223404923814
4183	Monaco	2006	284.72498852132	0.881274148	2.11289777274065	282.612090748579
4184	Monaco	2007	281.15035980127	0.883262291	2.11998719259412	279.030372608676
4185	Monaco	2008	278.61459887935	0.885331394	2.11325945687403	276.501339422476
4186	Monaco	2009	276.11351093589	0.887328279	2.11340175359953	274.00010918229
4187	Monaco	2010	273.69353534397	0.889262841	2.11467899252123	271.578856351449
4188	Monaco	2011	271.73540959541	0.891237592	2.11259451662877	269.622815078781
4189	Monaco	2012	269.65191850534	0.893073822	2.11140471905566	267.540513786284
4190	Monaco	2013	266.11179642714	0.894864472	2.11160358624541	264.000192840895
4191	Monaco	2014	262.97406160443	0.896547874	2.11814049698809	260.855921107442
4192	Monaco	2015	259.78018434379	0.898356337	2.11414839056574	257.666035953224
4193	Monaco	2016	256.05070803233	0.900051303	2.11117163508852	253.939536397242
4194	Monaco	2017	252.82450361141	0.901752712	2.11193064961016	250.7125729618
4195	Monaco	2018	250.34987011435	0.903388687	2.11445354070455	248.235416573645
4196	Monaco	2019	248.19527820724	0.905030991	2.11160020462688	246.083678002613
4197	Monaco	2020	244.36667956683	0.906685437	2.11760353871764	242.249076028112
4198	Monaco	2021	243.25277539974	0.908262831	2.11101482362129	241.141760576119
4199	Mongolia	1990	28.25029704121	0.466550118	2.11642083013014	26.1338762110799
4200	Mongolia	1991	27.91944911163	0.472199014	2.11687059805549	25.8025785135745

TableS2 Frontier analysis of Smoking-Related Low Back Pain among WCBA

	location	year	val	SDI	frontier	eff_diff
4201	Mongolia	1992	27.83991440585	0.477586554	2.11362499228986	25.7262894135601
4202	Mongolia	1993	27.95028526792	0.482726727	2.11246841720933	25.8378168507107
4203	Mongolia	1994	28.27565102067	0.488276683	2.11114096861603	26.164510052054
4204	Mongolia	1995	28.77454258335	0.495006781	2.11502785854284	26.6595147248072
4205	Mongolia	1996	29.44514591792	0.501703718	2.11658176700985	27.3285641509102
4206	Mongolia	1997	30.28332824462	0.508558268	2.113629860253	28.169698384367
4207	Mongolia	1998	31.35123888988	0.514793383	2.11278444278628	29.2384544470937
4208	Mongolia	1999	32.50664564616	0.520781189	2.11387489019714	30.3927707559629
4209	Mongolia	2000	33.6334853498	0.526650345	2.11199370626758	31.5214916435324
4210	Mongolia	2001	34.83641997362	0.532620201	2.11219093838757	32.7242290352324
4211	Mongolia	2002	36.42812765499	0.538424835	2.11075029848726	34.3173773565027
4212	Mongolia	2003	38.28979060844	0.543971651	2.11252918745123	36.1772614209888
4213	Mongolia	2004	40.18588237597	0.5495515	2.11399374948549	38.0718886264845
4214	Mongolia	2005	42.09280756682	0.554680284	2.11070172137	39.98210584545
4215	Mongolia	2006	44.06270631017	0.559561138	2.11298247179783	41.9497238383722
4216	Mongolia	2007	46.28982933828	0.564236795	2.11257242068765	44.1772569175924
4217	Mongolia	2008	48.65901979898	0.568500599	2.11339473270183	46.5456250662782
4218	Mongolia	2009	50.86850376392	0.571341686	2.11447625879338	48.7540275051266
4219	Mongolia	2010	52.48355914092	0.574406669	2.11303392279851	50.3705252181215
4220	Mongolia	2011	53.88668676316	0.578600663	2.11244733309112	51.7742394300689
4221	Mongolia	2012	55.18267325122	0.583255886	2.11371641441893	53.0689568368011
4222	Mongolia	2013	56.41228082564	0.588080811	2.11242065410662	54.2998601715334
4223	Mongolia	2014	57.70394294611	0.592867353	2.11633290367294	55.5876100424371
4224	Mongolia	2015	59.21893566626	0.596859648	2.11400461933359	57.1049310469264
4225	Mongolia	2016	61.04589778804	0.600260767	2.11303287527662	58.9328649127634

TableS2 Frontier analysis of Smoking-Related Low Back Pain among WCBA

	location	year	val	SDI	frontier	eff_diff
4226	Mongolia	2017	62.99819018088	0.603721965	2.11350796733068	60.8846822135493
4227	Mongolia	2018	64.95665001397	0.607501833	2.10935655581676	62.8472934581532
4228	Mongolia	2019	66.8732519019	0.611250681	2.1146097161046	64.7586421857954
4229	Mongolia	2020	68.45763740081	0.614629637	2.11264467356191	66.3449927272481
4230	Mongolia	2021	69.86632467614	0.617621565	2.11280890195846	67.7535157741815
4231	Montenegro	1990	384.43064506195	0.67422572	2.11192948323562	382.318715578714
4232	Montenegro	1991	388.03218760569	0.674305723	2.11275502498597	385.919432580704
4233	Montenegro	1992	393.15002409779	0.672664772	2.11272246745412	391.037301630336
4234	Montenegro	1993	400.43851454619	0.668665719	2.11471928765182	398.323795258538
4235	Montenegro	1994	408.64518629611	0.665011931	2.11352194460347	406.531664351506
4236	Montenegro	1995	417.70534252319	0.662765437	2.11215895822288	415.593183564967
4237	Montenegro	1996	427.01428164773	0.663414769	2.11208806054844	424.902193587182
4238	Montenegro	1997	434.17865368397	0.665542032	2.11161931624542	432.067034367725
4239	Montenegro	1998	440.3035775367	0.669024771	2.11146998222302	438.192107554477
4240	Montenegro	1999	445.5767550073	0.672096103	2.11429762811	443.46245737919
4241	Montenegro	2000	448.55482265046	0.67730685	2.11326082493141	446.441561825529
4242	Montenegro	2001	451.74690604411	0.683433454	2.11020321240476	449.636702831705
4243	Montenegro	2002	454.75212230262	0.689939483	2.11217792841896	452.639944374201
4244	Montenegro	2003	457.53200069495	0.696517506	2.11544210775149	455.416558587198
4245	Montenegro	2004	460.31802413539	0.702998276	2.11202061594701	458.206003519443
4246	Montenegro	2005	462.33816830505	0.709065465	2.11883205604629	460.219336249004
4247	Montenegro	2006	465.58317458737	0.715459074	2.11192176412288	463.471252823247
4248	Montenegro	2007	469.9911263578	0.72250394	2.11204133728015	467.87908502052
4249	Montenegro	2008	474.28482494964	0.729981756	2.11469108759944	472.170133862041
4250	Montenegro	2009	477.3032013947	0.736538926	2.11262051851092	475.190580876189

TableS2 Frontier analysis of Smoking-Related Low Back Pain among WCBA

	location	year	val	SDI	frontier	eff_diff
4251	Montenegro	2010	480.37820195926	0.743347873	2.11243235879054	478.265769600469
4252	Montenegro	2011	482.19488849729	0.750080328	2.11249524503878	480.082393252251
4253	Montenegro	2012	483.40625060811	0.75557838	2.11465182601949	481.29159878209
4254	Montenegro	2013	484.19094267377	0.760886907	2.11037379841754	482.080568875352
4255	Montenegro	2014	484.45547659685	0.765776527	2.11209817170198	482.343378425148
4256	Montenegro	2015	484.7386453059	0.770561278	2.11617254346464	482.622472762435
4257	Montenegro	2016	483.87775392022	0.77520077	2.11262561924191	481.765128300978
4258	Montenegro	2017	481.56885939634	0.779764632	2.11416125356592	479.454698142774
4259	Montenegro	2018	478.92790924855	0.784408971	2.11173533826439	476.816173910286
4260	Montenegro	2019	476.39721233466	0.789026903	2.1118385972287	474.285373737431
4261	Montenegro	2020	472.33739067154	0.792554393	2.11293209790229	470.224458573638
4262	Montenegro	2021	467.74904894013	0.795800584	2.11304053871081	465.636008401419
4263	Morocco	1990	12.62861763365	0.35807287	2.11370669275443	10.5149109408956
4264	Morocco	1991	12.56049598637	0.364984629	2.11450298057198	10.445993005798
4265	Morocco	1992	12.52463241519	0.371187194	2.11444854369883	10.4101838714912
4266	Morocco	1993	12.47181004808	0.376778977	2.11198895859321	10.3598210894868
4267	Morocco	1994	12.41515980142	0.382849451	2.11139093561432	10.3037688658057
4268	Morocco	1995	12.34315016374	0.38816411	2.11150163351762	10.2316485302224
4269	Morocco	1996	12.28090187438	0.394150096	2.11298069842197	10.167921175958
4270	Morocco	1997	12.2498831988	0.399504009	2.11450742500143	10.1353757737986
4271	Morocco	1998	12.17200195824	0.405038792	2.11274816735442	10.0592537908856
4272	Morocco	1999	12.10500889733	0.410303191	2.115637671164	9.989371226166
4273	Morocco	2000	12.06507426996	0.415344026	2.11355793496338	9.95151633499662
4274	Morocco	2001	11.90970728061	0.420595133	2.11280883708831	9.79689844352169
4275	Morocco	2002	11.763739862	0.42577575	2.11743092255508	9.64630893944492

TableS2 Frontier analysis of Smoking-Related Low Back Pain among WCBA

	location	year	val	SDI	frontier	eff_diff
4276	Morocco	2003	11.61714637315	0.43121781	2.11160416727058	9.50554220587942
4277	Morocco	2004	11.4540377687	0.436822352	2.11180933624512	9.34222843245488
4278	Morocco	2005	11.31026601916	0.442531339	2.11276384116149	9.19750217799851
4279	Morocco	2006	11.18214644289	0.44867616	2.11242922593397	9.06971721695603
4280	Morocco	2007	11.0768946441	0.455036325	2.11379134219172	8.96310330190828
4281	Morocco	2008	10.96764285506	0.461803552	2.11384162152566	8.85380123353434
4282	Morocco	2009	10.86618364833	0.468929893	2.11298775724065	8.75319589108935
4283	Morocco	2010	10.75169433363	0.476269799	2.1129190228772	8.6387753107528
4284	Morocco	2011	10.62561765321	0.483922152	2.11153878718157	8.51407886602843
4285	Morocco	2012	10.46301478593	0.491603392	2.11500557372672	8.34800921220328
4286	Morocco	2013	10.29169158405	0.499528607	2.11207039461524	8.17962118943476
4287	Morocco	2014	10.14058611971	0.507443265	2.11200218539435	8.02858393431564
4288	Morocco	2015	10.0501146373	0.515582893	2.11696102162681	7.93315361567319
4289	Morocco	2016	9.90550077281	0.523518592	2.11288800765921	7.79261276515079
4290	Morocco	2017	9.67769994645	0.531610862	2.11511611574603	7.56258383070397
4291	Morocco	2018	9.47188512779	0.539729671	2.11321224061518	7.35867288717482
4292	Morocco	2019	9.41730076077	0.547837444	2.11261285726732	7.30468790350268
4293	Morocco	2020	9.48921515106	0.555246069	2.11825047233913	7.37096467872087
4294	Morocco	2021	9.47321222007	0.562698301	2.11779430090859	7.35541791916141
4295	Mozambique	1990	20.01499473878	0.173064715	3.7060222634771	16.3089724753029
4296	Mozambique	1991	19.75101823867	0.175550435	3.67135474364292	16.0796634950271
4297	Mozambique	1992	19.47755939304	0.177285188	3.67736750972818	15.8001918833118
4298	Mozambique	1993	19.13630600646	0.179851807	3.541351711958	15.594954294502
4299	Mozambique	1994	18.6296345876	0.182236861	3.65402486692281	14.9756097206772
4300	Mozambique	1995	17.9844765914	0.183427982	3.64706782087028	14.3374087705297

TableS2 Frontier analysis of Smoking-Related Low Back Pain among WCBA

	location	year	val	SDI	frontier	eff_diff
4301	Mozambique	1996	17.54848304022	0.185705869	3.58749673878517	13.9609863014348
4302	Mozambique	1997	17.30305960808	0.188650436	3.5666387380759	13.7364208700041
4303	Mozambique	1998	17.10546770219	0.191998629	3.51793118330871	13.5875365188813
4304	Mozambique	1999	17.0202760226	0.195435288	3.51267602122164	13.5076000013784
4305	Mozambique	2000	17.15270727418	0.198534397	3.48125355720333	13.6714537169767
4306	Mozambique	2001	17.35700140607	0.202241924	3.46303367029367	13.8939677357763
4307	Mozambique	2002	17.69084259768	0.20639006	3.43253953707922	14.2583030606008
4308	Mozambique	2003	18.07853253229	0.21053449	3.47182055825156	14.6067119740384
4309	Mozambique	2004	18.3887834499	0.214972457	3.48162425951603	14.907159190384
4310	Mozambique	2005	18.51919106603	0.219692372	2.86343651203844	15.6557545539916
4311	Mozambique	2006	18.5332799019	0.224758087	2.76761580358811	15.7656640983119
4312	Mozambique	2007	18.43581655427	0.230158227	2.63653167671656	15.7992848775534
4313	Mozambique	2008	18.35961767486	0.23579017	2.60440629725953	15.7552113776005
4314	Mozambique	2009	18.30879799968	0.241360656	2.53046432295892	15.7783336767211
4315	Mozambique	2010	18.20593003788	0.2467515	2.55261200302317	15.6533180348568
4316	Mozambique	2011	18.07835824462	0.252232471	2.53061206312329	15.5477461814967
4317	Mozambique	2012	17.86975303131	0.258315562	2.51004024656544	15.3597127847446
4318	Mozambique	2013	17.62156413827	0.265280462	2.51205054756776	15.1095135907022
4319	Mozambique	2014	17.33885222588	0.272892814	2.45535372790323	14.8834984979768
4320	Mozambique	2015	17.11145869922	0.281032685	2.45472405352158	14.6567346456984
4321	Mozambique	2016	16.91506800414	0.289152425	2.43460709225947	14.4804609118805
4322	Mozambique	2017	16.72707830702	0.297164772	2.39937846629406	14.3276998407259
4323	Mozambique	2018	16.5886273819	0.305226772	2.3915055402604	14.1971218416396
4324	Mozambique	2019	16.40694963145	0.313174508	2.36123929841103	14.045710333039
4325	Mozambique	2020	16.11948346975	0.320291182	2.33691032644966	13.7825731433003

TableS2 Frontier analysis of Smoking-Related Low Back Pain among WCBA

	location	year	val	SDI	frontier	eff_diff
4326	Mozambique	2021	15.80232637638	0.326462614	2.32131496070022	13.4810114156798
4327	Myanmar	1990	30.8753061008	0.319219724	2.34175891791404	28.533547182886
4328	Myanmar	1991	30.39133770157	0.322034719	2.33812956721517	28.0532081343548
4329	Myanmar	1992	30.02687846604	0.325628884	2.31725238684306	27.7096260791969
4330	Myanmar	1993	29.66558863245	0.329707434	2.30044854508765	27.3651400873624
4331	Myanmar	1994	29.45138545842	0.33429012	2.28532736415536	27.1660580942646
4332	Myanmar	1995	29.09162670386	0.339590976	2.27095668731147	26.8206700165485
4333	Myanmar	1996	29.01901825225	0.345191641	2.13341254752314	26.8856057047269
4334	Myanmar	1997	29.03326719086	0.350892883	2.11485847450048	26.9184087163595
4335	Myanmar	1998	29.00973511331	0.356696902	2.11889461072492	26.8908405025851
4336	Myanmar	1999	28.89869011137	0.363037732	2.11872998680808	26.7799601245619
4337	Myanmar	2000	28.91091605148	0.370360017	2.11445973160489	26.7964563198751
4338	Myanmar	2001	28.49593823974	0.37817198	2.11486035252027	26.3810778872197
4339	Myanmar	2002	27.95651211827	0.386343628	2.11180598773438	25.8447061305356
4340	Myanmar	2003	27.32980024665	0.395218766	2.11212112723745	25.2176791194125
4341	Myanmar	2004	26.61917819357	0.404692358	2.11089878712297	24.508279406447
4342	Myanmar	2005	25.93156849368	0.414662082	2.11156049108121	23.8200080025988
4343	Myanmar	2006	25.08464134389	0.424705071	2.11291600893593	22.9717253349541
4344	Myanmar	2007	24.12434375022	0.434536927	2.11420121628147	22.0101425339385
4345	Myanmar	2008	23.14201825143	0.443595531	2.11242559490646	21.0295926565235
4346	Myanmar	2009	22.15041418598	0.451954884	2.110058913917	20.040355272063
4347	Myanmar	2010	21.329964199	0.460180429	2.11408735082375	19.2158768481763
4348	Myanmar	2011	20.51940560709	0.467850872	2.11244077708577	18.4069648300042
4349	Myanmar	2012	19.69442094888	0.475336423	2.11815531479345	17.5762656340866
4350	Myanmar	2013	18.90645679609	0.482821855	2.11505944167261	16.7913973544174

TableS2 Frontier analysis of Smoking-Related Low Back Pain among WCBA

	location	year	val	SDI	frontier	eff_diff
4351	Myanmar	2014	18.26037904919	0.490255145	2.11538375545265	16.1449952937374
4352	Myanmar	2015	17.76975612217	0.497374156	2.11012154723408	15.6596345749359
4353	Myanmar	2016	17.48953716656	0.504115978	2.11454504913406	15.3749921174259
4354	Myanmar	2017	17.28815213866	0.510823431	2.113082000222	15.175070138438
4355	Myanmar	2018	17.18565477045	0.517557469	2.11521289682386	15.0704418736261
4356	Myanmar	2019	17.06665118282	0.52410897	2.1117263516042	14.9549248312158
4357	Myanmar	2020	16.83958025258	0.529434703	2.11537203847155	14.7242082141085
4358	Myanmar	2021	16.64018776998	0.53390084	2.11279654848055	14.5273912214995
4359	Namibia	1990	29.55855440934	0.450040233	2.11303431139975	27.4455200979402
4360	Namibia	1991	29.45432807669	0.45453444	2.11558166979134	27.3387464068987
4361	Namibia	1992	29.29733086352	0.460021314	2.11593171411854	27.1813991494015
4362	Namibia	1993	29.17176899886	0.465734135	2.11611772818942	27.0556512706706
4363	Namibia	1994	29.12253220512	0.471873217	2.113550839427	27.008981365693
4364	Namibia	1995	29.07216601944	0.477794329	2.11469835803727	26.9574676614027
4365	Namibia	1996	29.0570791042	0.483116378	2.11370119033253	26.9433779138675
4366	Namibia	1997	29.02578004687	0.488012938	2.11726413268889	26.9085159141811
4367	Namibia	1998	28.89354530986	0.492812548	2.11858469679356	26.7749606130664
4368	Namibia	1999	28.82318048316	0.497613862	2.113126179366	26.710054303794
4369	Namibia	2000	28.75781195294	0.502406958	2.11466792292096	26.643144030019
4370	Namibia	2001	28.53377451014	0.506719033	2.11035352380925	26.4234209863307
4371	Namibia	2002	28.23726462198	0.510818275	2.1124047369207	26.1248598850593
4372	Namibia	2003	27.89009933707	0.514574645	2.11418841366828	25.7759109234017
4373	Namibia	2004	27.48732356326	0.519103605	2.11386559292233	25.3734579703377
4374	Namibia	2005	27.01447041111	0.523881736	2.113237957233	24.901232453877
4375	Namibia	2006	26.32924831359	0.529350118	2.11260074965969	24.2166475639303

TableS2 Frontier analysis of Smoking-Related Low Back Pain among WCBA

	location	year	val	SDI	frontier	eff_diff
4376	Namibia	2007	25.38940573425	0.535062126	2.11092222458747	23.2784835096625
4377	Namibia	2008	24.48409385164	0.540896995	2.11498796024221	22.3691058913978
4378	Namibia	2009	23.55927551739	0.546516477	2.11211326632654	21.4471622510635
4379	Namibia	2010	22.811041787	0.552741565	2.11019905522063	20.7008427317794
4380	Namibia	2011	22.23230487131	0.559618899	2.11351554190472	20.1187893294053
4381	Namibia	2012	21.64614395797	0.566978697	2.11142922013351	19.5347147378365
4382	Namibia	2013	21.12549037322	0.574590344	2.11328584142813	19.0122045317919
4383	Namibia	2014	20.77577207255	0.582321425	2.10950221423203	18.666269858318
4384	Namibia	2015	20.60215434916	0.58982104	2.11350193495625	18.4886524142037
4385	Namibia	2016	20.69457704341	0.596283059	2.11426667535742	18.5803103680526
4386	Namibia	2017	20.89323329662	0.601754645	2.11068301943403	18.782550277186
4387	Namibia	2018	21.17285422436	0.606618403	2.11371640618962	19.0591378181704
4388	Namibia	2019	21.36767894641	0.610921091	2.11410146131708	19.2535774850929
4389	Namibia	2020	21.54543874737	0.614435889	2.11065414215	19.43478460522
4390	Namibia	2021	21.62441138248	0.617564872	2.11297202350636	19.5114393589736
4391	Nauru	1990	140.0782538154	0.539145981	2.11409142304657	137.964162392353
4392	Nauru	1991	142.1108402229	0.538433557	2.11342567120836	139.997414551692
4393	Nauru	1992	143.96024763514	0.537038893	2.11418088349697	141.846066751643
4394	Nauru	1993	145.95695494106	0.535059742	2.1148931772512	143.842061763809
4395	Nauru	1994	147.80792348137	0.533001097	2.11225297434959	145.69567050702
4396	Nauru	1995	149.74048596375	0.530890925	2.11203391144432	147.628452052306
4397	Nauru	1996	152.03763320993	0.528544474	2.11171086319014	149.92592234674
4398	Nauru	1997	154.4513283195	0.52624159	2.11393468792578	152.337393631574
4399	Nauru	1998	156.68478221042	0.523885302	2.11323039603217	154.571551814388
4400	Nauru	1999	158.47137454483	0.521894324	2.1125995409827	156.358775003847

TableS2 Frontier analysis of Smoking-Related Low Back Pain among WCBA

	location	year	val	SDI	frontier	eff_diff
4401	Nauru	2000	159.48086339091	0.520254888	2.1132425717841	157.367620819126
4402	Nauru	2001	159.66720364448	0.519235425	2.11010868486797	157.557094959612
4403	Nauru	2002	159.77019507022	0.518438705	2.11361088688357	157.656584183336
4404	Nauru	2003	160.01257616277	0.518459986	2.1177825452093	157.894793617561
4405	Nauru	2004	159.83071518022	0.51880954	2.11632302006971	157.71439216015
4406	Nauru	2005	159.41593563485	0.519892863	2.11387661459771	157.302059020252
4407	Nauru	2006	158.28417630611	0.522673179	2.11831098029452	156.165865325815
4408	Nauru	2007	156.32474975771	0.52320135	2.11513992135564	154.209609836354
4409	Nauru	2008	153.83527749997	0.526084096	2.11568357459113	151.719593925379
4410	Nauru	2009	151.64939030456	0.530221092	2.11439171923765	149.534998585322
4411	Nauru	2010	149.80036988103	0.535689995	2.11361836057425	147.686751520456
4412	Nauru	2011	148.4439485196	0.542342743	2.11337596984246	146.330572549758
4413	Nauru	2012	146.92498337681	0.549890621	2.11193126077071	144.813052116039
4414	Nauru	2013	145.98914749806	0.560112906	2.115513681065	143.873633816995
4415	Nauru	2014	145.05540706656	0.571424364	2.11136862076205	142.944038445798
4416	Nauru	2015	144.08469050354	0.581283234	2.11495575196884	141.969734751571
4417	Nauru	2016	143.2599985728	0.590102988	2.11280425171595	141.147194321084
4418	Nauru	2017	142.34953273026	0.597619076	2.1126839352089	140.236848795051
4419	Nauru	2018	141.47326858702	0.605311879	2.11221817677159	139.361050410248
4420	Nauru	2019	140.59210326294	0.612697253	2.11225326511552	138.479849997824
4421	Nauru	2020	139.14377887438	0.619330076	2.11128006074277	137.032498813637
4422	Nauru	2021	139.14427796875	0.625177834	2.10985738086083	137.034420587889
4423	Nepal	1990	168.36670115345	0.199560654	3.46195618169492	164.904744971755
4424	Nepal	1991	169.82907479576	0.205687443	3.4660848960782	166.362989899682
4425	Nepal	1992	170.92593159662	0.21211309	3.49734764857657	167.428583948043

TableS2 Frontier analysis of Smoking-Related Low Back Pain among WCBA

	location	year	val	SDI	frontier	eff_diff
4426	Nepal	1993	171.79423884032	0.218843431	2.94656444536465	168.847674394955
4427	Nepal	1994	171.8680283535	0.22607306	2.69394580850936	169.174082544991
4428	Nepal	1995	172.10336845562	0.233253154	2.60176284923524	169.501605606385
4429	Nepal	1996	172.06675917554	0.240700616	2.54649390261673	169.520265272923
4430	Nepal	1997	171.73736957569	0.248382797	2.57984272793902	169.157526847751
4431	Nepal	1998	171.49172673427	0.256227179	2.52456619722831	168.967160537042
4432	Nepal	1999	170.94652435946	0.26435918	2.47729532081567	168.469229038644
4433	Nepal	2000	169.36174549267	0.272719115	2.46591725252638	166.895828240144
4434	Nepal	2001	166.92439468578	0.281287467	2.45609518392714	164.468299501853
4435	Nepal	2002	163.2134310708	0.289513608	2.43428696163957	160.77914410916
4436	Nepal	2003	158.57522692766	0.297772595	2.4034006058313	156.171826321829
4437	Nepal	2004	153.20003444486	0.30619653	2.38541512015612	150.814619324704
4438	Nepal	2005	148.20981128351	0.314661796	2.36471838363882	145.845092899871
4439	Nepal	2006	143.17448346659	0.323056346	2.34173725174487	140.832746214845
4440	Nepal	2007	137.21635574158	0.33144561	2.2960463948915	134.920309346688
4441	Nepal	2008	130.71779280312	0.340150706	2.24140653560774	128.476386267512
4442	Nepal	2009	124.17561589715	0.348701893	2.11513151145541	122.060484385695
4443	Nepal	2010	118.04761610991	0.3568681	2.11392685224117	115.933689257669
4444	Nepal	2011	111.74059476194	0.364642605	2.11298666594303	109.627608095997
4445	Nepal	2012	104.93212028613	0.372437588	2.11398945167011	102.81813083446
4446	Nepal	2013	98.37464063213	0.380022326	2.11482591761415	96.2598147145159
4447	Nepal	2014	92.74837086489	0.387689088	2.11587325287582	90.6324976120142
4448	Nepal	2015	88.47139776265	0.395021154	2.11477363700031	86.3566241256497
4449	Nepal	2016	84.3855249096	0.401628776	2.11292794455573	82.2725969650443
4450	Nepal	2017	79.60442910929	0.408463874	2.11143530708435	77.4929938022056

TableS2 Frontier analysis of Smoking-Related Low Back Pain among WCBA

	location	year	val	SDI	frontier	eff_diff
4451	Nepal	2018	75.4445728482	0.415236684	2.11388801707523	73.3306848311248
4452	Nepal	2019	73.35091964996	0.422007252	2.11608520045278	71.2348344495072
4453	Nepal	2020	73.1391777999	0.427417089	2.11439627778649	71.0247815221135
4454	Nepal	2021	72.81641296767	0.433174635	2.11271827196719	70.7036946957028
4455	Netherlands	1990	259.03796126866	0.794612123	2.11255363775574	256.925407630904
4456	Netherlands	1991	258.25489448851	0.799285437	2.11340480627417	256.141489682236
4457	Netherlands	1992	257.70808668671	0.803965978	2.11774582733514	255.590340859375
4458	Netherlands	1993	257.61326557796	0.808278967	2.11119243863933	255.502073139321
4459	Netherlands	1994	257.36549418019	0.812530216	2.11128042244434	255.254213757746
4460	Netherlands	1995	257.40141622104	0.816662769	2.11253387124745	255.288882349793
4461	Netherlands	1996	257.46110375356	0.81998422	2.11176569597744	255.349338057583
4462	Netherlands	1997	255.38932453349	0.82288177	2.11648510554149	253.272839427949
4463	Netherlands	1998	253.38755205923	0.825882234	2.11200347222791	251.275548587002
4464	Netherlands	1999	252.67239206178	0.828693827	2.11605434202103	250.556337719759
4465	Netherlands	2000	252.14333714968	0.831913984	2.11202328902842	250.031313860652
4466	Netherlands	2001	251.8086258646	0.835518148	2.11245475944393	249.696171105156
4467	Netherlands	2002	250.53573562314	0.838676077	2.11257018805394	248.423165435086
4468	Netherlands	2003	248.33078455011	0.841639477	2.11348730376778	246.217297246342
4469	Netherlands	2004	245.73118424141	0.844807348	2.1118490390187	243.619335202391
4470	Netherlands	2005	243.01196639616	0.84793419	2.11387096329291	240.898095432867
4471	Netherlands	2006	238.04106755774	0.850798808	2.11213911846015	235.92892843928
4472	Netherlands	2007	232.05893050157	0.853595391	2.11420243623222	229.944728065338
4473	Netherlands	2008	224.95041884379	0.856368467	2.11306815834162	222.837350685448
4474	Netherlands	2009	217.48779144475	0.858600459	2.11239107153226	215.375400373218
4475	Netherlands	2010	211.00945181653	0.861255298	2.11158559556059	208.897866220969

TableS2 Frontier analysis of Smoking-Related Low Back Pain among WCBA

	location	year	val	SDI	frontier	eff_diff
4476	Netherlands	2011	204.94395573148	0.864246678	2.11065185978923	202.833303871691
4477	Netherlands	2012	198.17500062003	0.866950626	2.11072757044338	196.064273049587
4478	Netherlands	2013	191.31035022286	0.869402979	2.11491156689359	189.195438655966
4479	Netherlands	2014	185.24871133396	0.871848914	2.11252951446148	183.136181819499
4480	Netherlands	2015	180.88498441981	0.874402841	2.11533573343153	178.769648686378
4481	Netherlands	2016	178.08157273247	0.876784052	2.11593734112377	175.965635391346
4482	Netherlands	2017	176.14561969182	0.879300152	2.11079635516818	174.034823336652
4483	Netherlands	2018	174.27227762863	0.881900218	2.11534517280527	172.156932455825
4484	Netherlands	2019	172.94948283956	0.88464384	2.11606453321534	170.833418306345
4485	Netherlands	2020	169.14496425776	0.886558566	2.11613887378732	167.028825383973
4486	Netherlands	2021	169.81238744944	0.888464256	2.11177665625271	167.700610793187
4487	New Zealand	1990	371.22170475475	0.752321655	2.11148281571417	369.110221939036
4488	New Zealand	1991	370.20402744134	0.755616091	2.11393184605232	368.090095595288
4489	New Zealand	1992	370.83722739557	0.759461372	2.11576640755869	368.721460988011
4490	New Zealand	1993	372.7712724434	0.763634604	2.11567927853698	370.655593164863
4491	New Zealand	1994	374.90214622843	0.767118658	2.1159545212922	372.786191707138
4492	New Zealand	1995	377.08454933597	0.769998036	2.1124512604125	374.972098075557
4493	New Zealand	1996	380.49648949587	0.773575283	2.11586051571218	378.380628980158
4494	New Zealand	1997	383.64000693932	0.778205776	2.11224778371113	381.527759155609
4495	New Zealand	1998	388.04880251979	0.781730757	2.11053931302591	385.938263206764
4496	New Zealand	1999	393.02210546875	0.784428638	2.11499130706645	390.907114161684
4497	New Zealand	2000	396.92557542286	0.787659557	2.11239990958962	394.81317551327
4498	New Zealand	2001	398.95471021564	0.791426079	2.11022102453784	396.844489191102
4499	New Zealand	2002	396.33240054452	0.794519591	2.11471554896102	394.217684995559
4500	New Zealand	2003	389.11500136337	0.796286883	2.1137875194513	387.001213843919

TableS2 Frontier analysis of Smoking-Related Low Back Pain among WCBA

	location	year	val	SDI	frontier	eff_diff
4501	New Zealand	2004	380.70958063531	0.798177477	2.11167380058393	378.597906834726
4502	New Zealand	2005	373.30325286492	0.799107294	2.1120195764	371.19123328852
4503	New Zealand	2006	364.35957653968	0.798456866	2.11426876037821	362.245307779302
4504	New Zealand	2007	354.35284418536	0.798447616	2.1130289922282	352.239815193132
4505	New Zealand	2008	343.96172308822	0.799669514	2.11232220075475	341.849400887465
4506	New Zealand	2009	333.52255476551	0.801727535	2.11428360343547	331.408271162075
4507	New Zealand	2010	324.96878183633	0.805133185	2.11516202265728	322.853619813673
4508	New Zealand	2011	316.81397075261	0.809760352	2.11458255087471	314.699388201735
4509	New Zealand	2012	308.43647777513	0.814957564	2.11208824136	306.32438953377
4510	New Zealand	2013	300.22878502278	0.82014599	2.1131283903419	298.115656632438
4511	New Zealand	2014	293.11453441874	0.823908944	2.11301183155222	291.001522587188
4512	New Zealand	2015	287.69579875409	0.827462493	2.11013147598588	285.585667278104
4513	New Zealand	2016	284.33375819479	0.831843967	2.11309926129111	282.220658933499
4514	New Zealand	2017	282.69778859269	0.836301795	2.11285085271883	280.584937739971
4515	New Zealand	2018	282.33373180998	0.840597863	2.11475493879174	280.218976871188
4516	New Zealand	2019	282.29863161929	0.844533209	2.11418667121844	280.184444948072
4517	New Zealand	2020	274.5467776447	0.847398737	2.11256398407823	272.434213660622
4518	New Zealand	2021	279.17073162996	0.849442499	2.11468020606018	277.0560514239
4519	Nicaragua	1990	46.46202256952	0.346035235	2.14280471430806	44.3192178552119
4520	Nicaragua	1991	45.50391755813	0.351740622	2.12154138808281	43.3823761700472
4521	Nicaragua	1992	44.69967014098	0.357712704	2.11459366397667	42.5850764770033
4522	Nicaragua	1993	43.97671369966	0.363562069	2.11219162033518	41.8645220793248
4523	Nicaragua	1994	43.36790150991	0.369727968	2.11622573908962	41.2516757708204
4524	Nicaragua	1995	43.03942302465	0.376625345	2.11435310426661	40.9250699203834
4525	Nicaragua	1996	42.75241320603	0.384087266	2.1143955218924	40.6380176841376

TableS2 Frontier analysis of Smoking-Related Low Back Pain among WCBA

	location	year	val	SDI	frontier	eff_diff
4526	Nicaragua	1997	42.48610695647	0.391775893	2.11421595954676	40.3718909969232
4527	Nicaragua	1998	42.26521023916	0.399807535	2.11492759650917	40.1502826426508
4528	Nicaragua	1999	42.13709197452	0.408085965	2.11148088607518	40.0256110884448
4529	Nicaragua	2000	42.22681243363	0.416108363	2.11427817778083	40.1125342558492
4530	Nicaragua	2001	42.49752592861	0.423790295	2.1103624733607	40.3871634552493
4531	Nicaragua	2002	42.6754390363	0.430813755	2.11380515106477	40.5616338852352
4532	Nicaragua	2003	42.91646619086	0.437248152	2.11767520942467	40.7987909814353
4533	Nicaragua	2004	43.22409191299	0.443274511	2.11209599500237	41.1119959179876
4534	Nicaragua	2005	43.64430278814	0.448844739	2.11406629818162	41.5302364899584
4535	Nicaragua	2006	43.98421116224	0.453950499	2.11478123761413	41.8694299246259
4536	Nicaragua	2007	44.50868065308	0.459042798	2.12015238778407	42.3885282652959
4537	Nicaragua	2008	45.11689716937	0.463837699	2.11163003829982	43.0052671310702
4538	Nicaragua	2009	45.75620359208	0.467961839	2.11130869401061	43.6448948980694
4539	Nicaragua	2010	46.36308719281	0.472172	2.11478372843475	44.2483034643752
4540	Nicaragua	2011	47.07680872815	0.476593466	2.11222609234507	44.9645826358049
4541	Nicaragua	2012	47.76435181803	0.4812969	2.11522030055627	45.6491315174737
4542	Nicaragua	2013	48.46403779561	0.48610547	2.11511522207365	46.3489225735364
4543	Nicaragua	2014	49.17467886583	0.491207346	2.11568603535818	47.0589928304718
4544	Nicaragua	2015	49.89848743034	0.496391663	2.11456537000341	47.7839220603366
4545	Nicaragua	2016	50.55245444651	0.501853345	2.11208598004942	48.4403684664606
4546	Nicaragua	2017	51.06382174872	0.507482022	2.11675849730096	48.947063251419
4547	Nicaragua	2018	51.61495344775	0.512338228	2.11532581056707	49.4996276371829
4548	Nicaragua	2019	52.25759727568	0.516597046	2.11305651909548	50.1445407565845
4549	Nicaragua	2020	52.50531270528	0.52029267	2.11516460700435	50.3901480982756
4550	Nicaragua	2021	53.26304217119	0.523958472	2.1117866633875	51.1512555078025

TableS2 Frontier analysis of Smoking-Related Low Back Pain among WCBA

	location	year	val	SDI	frontier	eff_diff
4551	Niger	1990	4.83330329356	0.08086848	4.81377417419648	0.0195291193635203
4552	Niger	1991	4.85979752228	0.082848208	4.84936372890857	0.0104337933714289
4553	Niger	1992	4.83058249819	0.084771578	4.83038712017936	0.000195378010644731
4554	Niger	1993	4.82661506482	0.08669649	4.79471194840049	0.0319031164195067
4555	Niger	1994	4.81384689733	0.088608733	4.79256113001561	0.021285767314394
4556	Niger	1995	4.8229353428	0.090473708	4.81858449124521	0.00435085155478721
4557	Niger	1996	4.82309122573	0.092346359	4.75582771367526	0.067263512054744
4558	Niger	1997	4.83236734067	0.094219115	4.80365404136957	0.0287132993004349
4559	Niger	1998	4.88288413196	0.096545153	4.81820654367068	0.0646775882893182
4560	Niger	1999	4.9148423956	0.098813686	4.78046519391672	0.134377201683279
4561	Niger	2000	4.85797706766	0.100886948	4.68338110747221	0.174595960187792
4562	Niger	2001	4.90142177058	0.103271777	4.69975632729027	0.201665443289726
4563	Niger	2002	4.946237056	0.105741109	4.76881417577458	0.177422880225417
4564	Niger	2003	4.99043711083	0.108322891	4.77133169071904	0.219105420110962
4565	Niger	2004	5.0309440935	0.110764495	4.81281620354684	0.218127889953159
4566	Niger	2005	5.07195599765	0.113452023	4.8180915379268	0.2538644597232
4567	Niger	2006	5.09761012834	0.116247074	4.7644925531382	0.333117575201804
4568	Niger	2007	5.10066768314	0.118971626	4.8190080187686	0.281659664371404
4569	Niger	2008	5.08701928375	0.121957786	4.81811474251361	0.268904541236394
4570	Niger	2009	5.06707077113	0.124829381	4.77057347915915	0.296497291970848
4571	Niger	2010	5.04604172711	0.127952996	4.70404565366788	0.341996073442117
4572	Niger	2011	5.00127339783	0.130958067	4.81723136090245	0.184042036927548
4573	Niger	2012	4.93802058311	0.134469718	4.7987237254471	0.139296857662903
4574	Niger	2013	4.87306764112	0.13797817	4.73688012403039	0.136187517089606
4575	Niger	2014	4.79888378169	0.141731738	4.78381354080425	0.0150702408857537

TableS2 Frontier analysis of Smoking-Related Low Back Pain among WCBA

	location	year	val	SDI	frontier	eff_diff
4576	Niger	2015	4.71782799459	0.145440503	4.70948064394693	0.00834735064306535
4577	Niger	2016	4.63790169397	0.149200109	4.59274857128353	0.0451531226864663
4578	Niger	2017	4.56510216093	0.15295917	4.35245794264169	0.212644218288312
4579	Niger	2018	4.50139449715	0.156875196	4.14170928259419	0.359685214555807
4580	Niger	2019	4.43066381968	0.160889484	4.07548242720183	0.355181392478171
4581	Niger	2020	4.35283032712	0.164539265	3.91656477339159	0.436265553728406
4582	Niger	2021	4.26730955785	0.168072774	3.81168509583641	0.455624462013594
4583	Nigeria	1990	6.87455727758	0.305868047	2.3860992524997	4.4884580250803
4584	Nigeria	1991	6.84236331737	0.308489225	2.38102369172875	4.46133962564125
4585	Nigeria	1992	6.78713131295	0.311234052	2.36115983120803	4.42597148174197
4586	Nigeria	1993	6.73910724408	0.31398306	2.36692646774197	4.37218077633803
4587	Nigeria	1994	6.69291516839	0.316747447	2.36425093111986	4.32866423727013
4588	Nigeria	1995	6.77713068866	0.319637439	2.33989021938932	4.43724046927068
4589	Nigeria	1996	6.85188535146	0.323137685	2.34166063238902	4.51022471907098
4590	Nigeria	1997	6.91329916479	0.326690553	2.31990136018932	4.59339780460068
4591	Nigeria	1998	6.98770356979	0.330263851	2.30886352762676	4.67884004216324
4592	Nigeria	1999	7.07702428379	0.3339365	2.28366573442449	4.79335854936551
4593	Nigeria	2000	7.06309372866	0.338201743	2.27271778253603	4.79037594612397
4594	Nigeria	2001	7.09086230887	0.343029751	2.21400177723706	4.87686053163294
4595	Nigeria	2002	7.1124010677	0.349092528	2.11268321733131	4.99971785036869
4596	Nigeria	2003	7.12393384661	0.355911392	2.1198779616244	5.0040558849856
4597	Nigeria	2004	7.12931344997	0.363780814	2.11219832861584	5.01711512135416
4598	Nigeria	2005	7.12418867304	0.372583553	2.11400476988	5.01018390316
4599	Nigeria	2006	7.07289639428	0.38102604	2.11305650943122	4.95983988484878
4600	Nigeria	2007	6.96272447672	0.389779649	2.11400184881127	4.84872262790873

TableS2 Frontier analysis of Smoking-Related Low Back Pain among WCBA

	location	year	val	SDI	frontier	eff_diff
4601	Nigeria	2008	6.80958156838	0.398037706	2.11491818421	4.69466338417
4602	Nigeria	2009	6.64258235959	0.406353307	2.11402156331686	4.52856079627314
4603	Nigeria	2010	6.48615776879	0.414997748	2.11798941156049	4.36816835722951
4604	Nigeria	2011	6.32127738446	0.423351915	2.11314785497613	4.20812952948387
4605	Nigeria	2012	6.12149532497	0.431780376	2.11339860917509	4.00809671579491
4606	Nigeria	2013	5.90483578209	0.440124664	2.11599795307045	3.78883782901955
4607	Nigeria	2014	5.70316268698	0.448766823	2.11246791020612	3.59069477677388
4608	Nigeria	2015	5.53286303639	0.457240991	2.11432454979779	3.41853848659221
4609	Nigeria	2016	5.37825153873	0.465183781	2.11334573346462	3.26490580526537
4610	Nigeria	2017	5.2153741356	0.472972869	2.11570497882373	3.09966915677627
4611	Nigeria	2018	5.06105780742	0.480844413	2.11063130478431	2.95042650263569
4612	Nigeria	2019	4.93905568194	0.488787361	2.11358772252892	2.82546795941108
4613	Nigeria	2020	4.83834696227	0.496204741	2.11284125891507	2.72550570335493
4614	Nigeria	2021	4.7589530053	0.503390833	2.11859200671063	2.64036099858937
4615	Niue	1990	81.41582779314	0.587532984	2.11165001606961	79.3041777770704
4616	Niue	1991	81.65542837073	0.593882057	2.11181513243722	79.5436132382928
4617	Niue	1992	81.42540418069	0.599881243	2.11878979254254	79.3066143881475
4618	Niue	1993	81.50526069541	0.605514779	2.1137057661008	79.3915549293092
4619	Niue	1994	81.97658220276	0.610650175	2.11534100152253	79.8612412012375
4620	Niue	1995	82.77896422636	0.615374609	2.11152872210985	80.6674355042502
4621	Niue	1996	83.70037761011	0.619734555	2.1144123168224	81.5859652932876
4622	Niue	1997	83.97076797584	0.623913848	2.11342951324217	81.8573384625978
4623	Niue	1998	85.70188271736	0.627578934	2.11212357424744	83.5897591431126
4624	Niue	1999	87.88710065301	0.630857172	2.11420031550434	85.7729003375056
4625	Niue	2000	89.66807925577	0.63406895	2.1119678777291	87.5561113780409

TableS2 Frontier analysis of Smoking-Related Low Back Pain among WCBA

	location	year	val	SDI	frontier	eff_diff
4626	Niue	2001	90.39011829719	0.637366627	2.11308089127563	88.2770374059144
4627	Niue	2002	90.25945910709	0.641122021	2.11324057038461	88.1462185367054
4628	Niue	2003	89.83820858485	0.64577954	2.11235052790469	87.7258580569453
4629	Niue	2004	88.94794295073	0.650614832	2.11219797255778	86.8357449781722
4630	Niue	2005	87.91877038349	0.657281265	2.1119651385606	85.8068052449294
4631	Niue	2006	87.12099611541	0.664162983	2.11270426876456	85.0082918466454
4632	Niue	2007	87.18247029767	0.670199299	2.11407578524951	85.0683945124205
4633	Niue	2008	87.45160785595	0.676396859	2.11533490014042	85.3362729558096
4634	Niue	2009	87.41676464214	0.682579173	2.11130869401061	85.3054559481294
4635	Niue	2010	87.45521740349	0.68628063	2.11211949196209	85.3430979115279
4636	Niue	2011	87.51980512619	0.690315578	2.11142788182203	85.408377244368
4637	Niue	2012	88.31672896819	0.694150949	2.11219498484727	86.2045339833427
4638	Niue	2013	88.9347931107	0.697221078	2.11383379845267	86.8209593122473
4639	Niue	2014	89.1597738733	0.700545924	2.1154627652558	87.0443111080442
4640	Niue	2015	89.01625352527	0.704232979	2.11457348486639	86.9016800404036
4641	Niue	2016	88.64943345452	0.707476099	2.11402204164391	86.5354114128761
4642	Niue	2017	88.74440358925	0.711220996	2.115278286155	86.629125303095
4643	Niue	2018	88.45857497979	0.71510823	2.1123681437425	86.3462068360475
4644	Niue	2019	88.00045091427	0.719078885	2.11579818650333	85.8846527277667
4645	Niue	2020	87.78841457327	0.72279025	2.11164019737408	85.6767743758959
4646	Niue	2021	87.39166325713	0.72622205	2.11442459893393	85.2772386581961
4647	North Africa and Middle East	1990	38.74731412343	0.437420668	2.1117941101077	36.6355200133223
4648	North Africa and Middle East	1991	38.89353821173	0.445920934	2.11493797743437	36.7786002342956
4649	North Africa and Middle East	1992	39.03263612387	0.453971432	2.11374909578618	36.9188870280838
4650	North Africa and Middle East	1993	39.26658838578	0.462102156	2.11671867574387	37.1498697100361

TableS2 Frontier analysis of Smoking-Related Low Back Pain among WCBA

	location	year	val	SDI	frontier	eff_diff
4651	North Africa and Middle East	1994	39.51903851591	0.470393819	2.11216189396854	37.4068766219415
4652	North Africa and Middle East	1995	39.83460167596	0.478553399	2.11166780169641	37.7229338742636
4653	North Africa and Middle East	1996	40.18161056225	0.486462858	2.11360364891458	38.0680069133354
4654	North Africa and Middle East	1997	40.5161250998	0.493980049	2.11173481118925	38.4043902886107
4655	North Africa and Middle East	1998	40.807027554	0.501445054	2.11168957358892	38.6953379804111
4656	North Africa and Middle East	1999	41.02960944459	0.508955784	2.11612073651603	38.913488708074
4657	North Africa and Middle East	2000	41.28312473772	0.516824215	2.1128951466372	39.1702295910828
4658	North Africa and Middle East	2001	41.43090404881	0.524091271	2.11104497379571	39.3198590750143
4659	North Africa and Middle East	2002	41.53868217935	0.531149449	2.11416955760649	39.4245126217435
4660	North Africa and Middle East	2003	41.64276901286	0.538292126	2.11027869049926	39.5324903223607
4661	North Africa and Middle East	2004	41.73853233206	0.545678163	2.11221101448671	39.6263213175733
4662	North Africa and Middle East	2005	41.77574066706	0.553047386	2.11440355424154	39.6613371128185
4663	North Africa and Middle East	2006	41.73468355016	0.560088959	2.11306284320181	39.6216207069582
4664	North Africa and Middle East	2007	41.69325290956	0.566516099	2.11356261103242	39.5796902985276
4665	North Africa and Middle East	2008	41.63055613394	0.572339255	2.11242198549937	39.5181341484406
4666	North Africa and Middle East	2009	41.58953135959	0.577062164	2.11583772520234	39.4736936343877
4667	North Africa and Middle East	2010	41.72014601126	0.581849041	2.11103010538493	39.6091159058751
4668	North Africa and Middle East	2011	42.09265755416	0.587593075	2.11492380494224	39.9777337492178
4669	North Africa and Middle East	2012	42.69203753491	0.594111701	2.11247142328047	40.5795661116295
4670	North Africa and Middle East	2013	43.38744053889	0.601127066	2.1100790529442	41.2773614859458
4671	North Africa and Middle East	2014	44.01844265669	0.608418687	2.11369151460319	41.9047511420868
4672	North Africa and Middle East	2015	44.49038382231	0.61577742	2.1132553661084	42.3771284562016
4673	North Africa and Middle East	2016	44.83746692319	0.623142325	2.1134848928361	42.7239820303539
4674	North Africa and Middle East	2017	45.18807934711	0.630560773	2.11327667396103	43.074802673149
4675	North Africa and Middle East	2018	45.44500974411	0.637917915	2.11293150166027	43.3320782424497

TableS2 Frontier analysis of Smoking-Related Low Back Pain among WCBA

	location	year	val	SDI	frontier	eff_diff
4676	North Africa and Middle East	2019	45.65870168379	0.645094792	2.11545648016082	43.5432452036292
4677	North Africa and Middle East	2020	45.73591541416	0.651673493	2.11478639511062	43.6211290190494
4678	North Africa and Middle East	2021	45.54012415228	0.658224715	2.11471005818299	43.425414094097
4679	North Korea	1990	24.1320952052	0.497780128	2.11417856921	22.01791663599
4680	North Korea	1991	24.39766540915	0.499120512	2.11312055932821	22.2845448498218
4681	North Korea	1992	24.70814916673	0.499629841	2.11329085242551	22.5948583143045
4682	North Korea	1993	24.94672304057	0.499700646	2.11348195103827	22.8332410895317
4683	North Korea	1994	25.09925953234	0.49953134	2.1187764564925	22.9804830758475
4684	North Korea	1995	25.23999911613	0.499080529	2.11281698658886	23.1271821295411
4685	North Korea	1996	25.44168721833	0.498429014	2.11508371544686	23.3266035028831
4686	North Korea	1997	25.83665890659	0.497136869	2.11099109101141	23.7256678155786
4687	North Korea	1998	26.45502403846	0.49615921	2.11387778567507	24.3411462527849
4688	North Korea	1999	27.04815159638	0.496484754	2.11910320064683	24.9290483957332
4689	North Korea	2000	27.72154051046	0.497461995	2.112561558609	25.608978951851
4690	North Korea	2001	28.07953155188	0.499674043	2.11191515985408	25.9676163920259
4691	North Korea	2002	28.22650767656	0.502633569	2.11265303300585	26.1138546435542
4692	North Korea	2003	28.21788862203	0.506165243	2.1133969841169	26.1044916379131
4693	North Korea	2004	28.11886049586	0.510229134	2.11563985264879	26.0032206432112
4694	North Korea	2005	28.18863510974	0.515097763	2.1140352276693	26.0745998820707
4695	North Korea	2006	28.24168485605	0.51974144	2.11214935288692	26.1295355031631
4696	North Korea	2007	28.28153955878	0.524066965	2.11701911683449	26.1645204419455
4697	North Korea	2008	28.31829927971	0.528813498	2.11273381506165	26.2055654646484
4698	North Korea	2009	28.33665106939	0.533114411	2.11474841675471	26.2219026526353
4699	North Korea	2010	28.42197114384	0.537192918	2.11651719412387	26.3054539497161
4700	North Korea	2011	28.50843286726	0.54121227	2.11714765119922	26.3912852160608

TableS2 Frontier analysis of Smoking-Related Low Back Pain among WCBA

	location	year	val	SDI	frontier	eff_diff
4701	North Korea	2012	28.71080891834	0.545331713	2.112161641501	26.598647276839
4702	North Korea	2013	28.83415659008	0.549483297	2.11683748314338	26.7173191069366
4703	North Korea	2014	28.96545980422	0.55360356	2.11310800925821	26.8523517949618
4704	North Korea	2015	29.06074540294	0.557321271	2.11184915754918	26.9488962453908
4705	North Korea	2016	29.11339630277	0.560743563	2.1119596427431	27.0014366600269
4706	North Korea	2017	29.04230381385	0.563388289	2.11515140671413	26.9271524071359
4707	North Korea	2018	28.91248017783	0.565161484	2.11219960877769	26.8002805690523
4708	North Korea	2019	28.69285520624	0.566765706	2.11192243934956	26.5809327668904
4709	North Korea	2020	28.5393938721	0.568188551	2.11244729459182	26.4269465775082
4710	North Korea	2021	28.09884467731	0.569854634	2.11301023354623	25.9858344437638
4711	North Macedonia	1990	317.3599802615	0.609026094	2.11288663069527	315.247093630805
4712	North Macedonia	1991	320.01453201468	0.611337453	2.11448640400958	317.90004561067
4713	North Macedonia	1992	322.4848402444	0.612708856	2.11415544219875	320.370684802201
4714	North Macedonia	1993	324.77876451385	0.613195099	2.11258811802716	322.666176395823
4715	North Macedonia	1994	329.03880851212	0.614362905	2.11357425349313	326.925234258627
4716	North Macedonia	1995	334.44068390812	0.616994878	2.11720070088494	332.323483207235
4717	North Macedonia	1996	338.4747968137	0.620944017	2.11314578305014	336.36165103065
4718	North Macedonia	1997	342.53589965552	0.626223811	2.11152651138961	340.42437314413
4719	North Macedonia	1998	345.27200222434	0.63205941	2.11649092829811	343.155511296042
4720	North Macedonia	1999	344.04296243506	0.636687259	2.11167756293079	341.931284872129
4721	North Macedonia	2000	340.93774091499	0.641651243	2.11288956527647	338.824851349714
4722	North Macedonia	2001	342.8323571654	0.648049676	2.11280738136522	340.719549784035
4723	North Macedonia	2002	343.40546445154	0.65539879	2.11142438735284	341.294040064187
4724	North Macedonia	2003	343.50685951183	0.663408891	2.11722269192325	341.389636819907
4725	North Macedonia	2004	342.97214125558	0.67123752	2.11533377732014	340.85680747826

TableS2 Frontier analysis of Smoking-Related Low Back Pain among WCBA

	location	year	val	SDI	frontier	eff_diff
4726	North Macedonia	2005	343.21401721318	0.678047273	2.11348406868661	341.100533144493
4727	North Macedonia	2006	345.30609291921	0.684044336	2.11283427825329	343.193258640957
4728	North Macedonia	2007	349.66129268145	0.689729481	2.11429720608528	347.546995475365
4729	North Macedonia	2008	354.87558047761	0.695376955	2.11293129169789	352.762649185912
4730	North Macedonia	2009	359.8135128627	0.700453052	2.11133908062971	357.70217378207
4731	North Macedonia	2010	364.27878965889	0.705589324	2.11193717090423	362.166852487986
4732	North Macedonia	2011	368.94845546328	0.710592521	2.10990669291552	366.838548770365
4733	North Macedonia	2012	374.04930581422	0.715173148	2.11430130145024	371.93500451277
4734	North Macedonia	2013	379.14379966259	0.719671149	2.11454833262625	377.029251329964
4735	North Macedonia	2014	384.54768538347	0.724089589	2.11001333830085	382.437672045169
4736	North Macedonia	2015	388.67105148216	0.728603773	2.11195528359306	386.559096198567
4737	North Macedonia	2016	391.87217803593	0.733173624	2.11243886786242	389.759739168068
4738	North Macedonia	2017	393.67485037594	0.737339955	2.11456152326853	391.560288852671
4739	North Macedonia	2018	395.01528567581	0.74133437	2.11904456861025	392.8962411072
4740	North Macedonia	2019	397.48016785246	0.745193569	2.11158727114033	395.36858058132
4741	North Macedonia	2020	396.63401031681	0.7478499	2.11363189973676	394.520378417073
4742	North Macedonia	2021	397.4947543517	0.750629703	2.11591068482771	395.378843666872
4743	Northern Mariana Islands	1990	68.36185320643	0.708593838	2.11269905822987	66.2491541482001
4744	Northern Mariana Islands	1991	65.86377014784	0.712223642	2.1132084561815	63.7505616916585
4745	Northern Mariana Islands	1992	67.20716785143	0.715787607	2.11204475229806	65.0951230991319
4746	Northern Mariana Islands	1993	71.61996693143	0.719179396	2.11147192365431	69.5084950077757
4747	Northern Mariana Islands	1994	77.50986117934	0.722510494	2.11523891540161	75.3946222639384
4748	Northern Mariana Islands	1995	82.91417589562	0.726219053	2.11424248175082	80.7999334138692
4749	Northern Mariana Islands	1996	86.64243349071	0.730074086	2.11209265623316	84.5303408344768
4750	Northern Mariana Islands	1997	85.83918978826	0.733795366	2.11244439320322	83.7267453950568

TableS2 Frontier analysis of Smoking-Related Low Back Pain among WCBA

	location	year	val	SDI	frontier	eff_diff
4751	Northern Mariana Islands	1998	80.36215819453	0.737423127	2.11089596294289	78.2512622315871
4752	Northern Mariana Islands	1999	73.56986422303	0.740934815	2.11021940779261	71.4596448152374
4753	Northern Mariana Islands	2000	68.89846059885	0.744492865	2.11137647859839	66.7870841202516
4754	Northern Mariana Islands	2001	70.41944834503	0.747170606	2.11376068230134	68.3056876627286
4755	Northern Mariana Islands	2002	77.78980350483	0.748755189	2.1129085521264	75.6768949527036
4756	Northern Mariana Islands	2003	87.36191423202	0.750171533	2.11289878158802	85.249015450432
4757	Northern Mariana Islands	2004	94.82283408724	0.751528841	2.11355455290529	92.7092795343347
4758	Northern Mariana Islands	2005	101.31130700072	0.751826692	2.11181645945057	99.1994905412694
4759	Northern Mariana Islands	2006	104.46653384446	0.751797809	2.11422347671608	102.352310367744
4760	Northern Mariana Islands	2007	103.63884848257	0.751608416	2.11648147870864	101.522367003861
4761	Northern Mariana Islands	2008	101.61095705044	0.750807192	2.11429863779689	99.4966584126431
4762	Northern Mariana Islands	2009	101.11301390692	0.748597173	2.11241974044809	99.0005941664719
4763	Northern Mariana Islands	2010	102.73096669196	0.746617262	2.11288382770227	100.618082864258
4764	Northern Mariana Islands	2011	105.43630579377	0.744411908	2.11297287708569	103.323332916684
4765	Northern Mariana Islands	2012	106.04024425585	0.742937351	2.11313833052942	103.927105925321
4766	Northern Mariana Islands	2013	105.07577509539	0.74228436	2.11353138922114	102.962243706169
4767	Northern Mariana Islands	2014	102.07497655137	0.742605449	2.11184397290259	99.9631325784674
4768	Northern Mariana Islands	2015	98.02575198385	0.743797398	2.11219490393382	95.9135570799162
4769	Northern Mariana Islands	2016	91.76259967249	0.748455466	2.11233796512609	89.6502617073639
4770	Northern Mariana Islands	2017	86.81048909961	0.756495479	2.11802358324329	84.6924655163667
4771	Northern Mariana Islands	2018	85.11466626401	0.7612554	2.1134223178668	83.0012439461432
4772	Northern Mariana Islands	2019	86.63055431452	0.765770647	2.1147970733577	84.5157572411623
4773	Northern Mariana Islands	2020	89.53874426245	0.76942766	2.11614037116373	87.4226038912863
4774	Northern Mariana Islands	2021	92.30992352531	0.771535213	2.11628484038048	90.1936386849295
4775	Norway	1990	296.36088924008	0.795887277	2.11156129222429	294.249327947856

TableS2 Frontier analysis of Smoking-Related Low Back Pain among WCBA

	location	year	val	SDI	frontier	eff_diff
4776	Norway	1991	297.39568736364	0.800468886	2.11172196057328	295.283965403067
4777	Norway	1992	298.04063727568	0.805437473	2.11753427169283	295.923103003987
4778	Norway	1993	297.59411064538	0.810565689	2.11044175303893	295.483668892341
4779	Norway	1994	296.35838673517	0.815414668	2.11627746051754	294.242109274652
4780	Norway	1995	294.37984946235	0.820223522	2.11435206654342	292.265497395807
4781	Norway	1996	290.35203566112	0.825701668	2.11244845455852	288.239587206561
4782	Norway	1997	283.26976929619	0.83146877	2.11566681856658	281.154102477623
4783	Norway	1998	275.32336089735	0.836562809	2.11279840470584	273.210562492644
4784	Norway	1999	268.16690299397	0.841130237	2.11253694550145	266.054366048469
4785	Norway	2000	261.92456151992	0.846475451	2.11115823926602	259.813403280654
4786	Norway	2001	255.21915732402	0.852111936	2.11323005496648	253.105927269054
4787	Norway	2002	247.83680296962	0.856757888	2.11275328827411	245.724049681346
4788	Norway	2003	239.51616433412	0.860302576	2.11141397777556	237.404750356344
4789	Norway	2004	231.46159943308	0.863468908	2.11161922496319	229.349980208117
4790	Norway	2005	224.79703820239	0.866217488	2.11523630021925	222.681801902171
4791	Norway	2006	218.66067343303	0.868588435	2.11216505879154	216.548508374238
4792	Norway	2007	212.55895846446	0.87092967	2.11368409415761	210.445274370302
4793	Norway	2008	206.75882199203	0.873745121	2.11265452627128	204.646167465759
4794	Norway	2009	201.8531001275	0.876582817	2.11248044754636	199.740619679954
4795	Norway	2010	198.06854369739	0.880378381	2.1110579944114	195.957485702979
4796	Norway	2011	194.72114363939	0.884351667	2.11408290837045	192.60706073102
4797	Norway	2012	191.32961465989	0.888160475	2.11332796187368	189.216286698016
4798	Norway	2013	188.53858428895	0.891989618	2.11295966401254	186.425624624937
4799	Norway	2014	186.38209612945	0.895499546	2.11356813908877	184.268527990361
4800	Norway	2015	185.35809322427	0.898912644	2.11567572806577	183.242417496204

TableS2 Frontier analysis of Smoking-Related Low Back Pain among WCBA

	location	year	val	SDI	frontier	eff_diff
4801	Norway	2016	185.65598682207	0.902584187	2.11362508242143	183.542361739649
4802	Norway	2017	187.15026706598	0.906044049	2.11148230493074	185.038784761049
4803	Norway	2018	189.26061872449	0.909261782	2.11668278323693	187.143935941253
4804	Norway	2019	190.60468278023	0.912278761	2.11372218978385	188.490960590446
4805	Norway	2020	188.93623056902	0.91452992	2.11531073265594	186.820919836364
4806	Norway	2021	192.15567086426	0.91613281	2.11331613808206	190.042354726178
4807	Oceania	1990	65.90153321138	0.391195082	2.11197499745533	63.7895582139247
4808	Oceania	1991	66.47248153012	0.394418822	2.11467337026093	64.3578081598591
4809	Oceania	1992	66.93336514088	0.397572264	2.11077546872521	64.8225896721548
4810	Oceania	1993	67.38497473694	0.401113099	2.11217463939381	65.2728000975462
4811	Oceania	1994	67.89770993568	0.404892361	2.11167279468091	65.7860371409991
4812	Oceania	1995	68.32344202009	0.408507251	2.11154200152214	66.2119000185679
4813	Oceania	1996	69.0847533692	0.412437065	2.11209564034781	66.9726577288522
4814	Oceania	1997	69.335469353	0.415893127	2.11285167649042	67.2226176765096
4815	Oceania	1998	69.89106838926	0.419224844	2.11396241146537	67.7771059777946
4816	Oceania	1999	70.63980752546	0.422520527	2.12036114478645	68.5194463806735
4817	Oceania	2000	71.66210949394	0.425069659	2.11436258536254	69.5477469085775
4818	Oceania	2001	72.19120010551	0.427004026	2.1187162169288	70.0724838885812
4819	Oceania	2002	72.64194641526	0.42853491	2.11282521745894	70.5291211978011
4820	Oceania	2003	73.23502377954	0.43005967	2.11161110342147	71.1234126761185
4821	Oceania	2004	73.59418748585	0.431581333	2.114828579246	71.479358906604
4822	Oceania	2005	73.9142345159	0.433151845	2.11559736644667	71.7986371494533
4823	Oceania	2006	74.0393568352	0.434584099	2.11310399100931	71.9262528441907
4824	Oceania	2007	73.82961976467	0.436101755	2.11381564474129	71.7158041199287
4825	Oceania	2008	73.47889094913	0.43739244	2.11352605323054	71.3653648958995

TableS2 Frontier analysis of Smoking-Related Low Back Pain among WCBA

	location	year	val	SDI	frontier	eff_diff
4826	Oceania	2009	73.00529001652	0.438807984	2.11740323594623	70.8878867805738
4827	Oceania	2010	72.80644766494	0.440751869	2.1118007211953	70.6946469437447
4828	Oceania	2011	72.8406547245	0.442617769	2.1162189810625	70.7244357434375
4829	Oceania	2012	73.06796650686	0.444524399	2.11617119626281	70.9517953105972
4830	Oceania	2013	73.5185045093	0.446593514	2.1120182449536	71.4064862643464
4831	Oceania	2014	74.09372551685	0.449363524	2.11450081299518	71.9792247038548
4832	Oceania	2015	74.66345271527	0.452397261	2.11414559064013	72.5493071246299
4833	Oceania	2016	75.25910030766	0.455453805	2.11465820910512	73.1444420985549
4834	Oceania	2017	75.89402379941	0.458377792	2.11170696005354	73.7823168393565
4835	Oceania	2018	76.41468660162	0.460818103	2.11413952052151	74.3005470810985
4836	Oceania	2019	76.84973957834	0.463333934	2.11344252838304	74.736297049957
4837	Oceania	2020	76.55183723611	0.465534643	2.11452152832533	74.4373157077847
4838	Oceania	2021	77.25314626694	0.467445126	2.11861730161859	75.1345289653214
4839	Oman	1990	18.4979337912	0.429270949	2.112381237185	16.385552554015
4840	Oman	1991	18.19848099895	0.442268122	2.11218774830962	16.0862932506404
4841	Oman	1992	17.85830616197	0.457737972	2.11328717442855	15.7450189875414
4842	Oman	1993	17.41217012003	0.474999033	2.11206434994765	15.3001057700824
4843	Oman	1994	16.95629217323	0.493513107	2.1155949271308	14.8406972460992
4844	Oman	1995	16.52271590829	0.513194359	2.1161715898158	14.4065443184742
4845	Oman	1996	15.99116507225	0.533246394	2.11618882903182	13.8749762432182
4846	Oman	1997	15.34650223031	0.554315537	2.11230345054284	13.2341987797672
4847	Oman	1998	14.88382785268	0.574474902	2.11461421570575	12.7692136369743
4848	Oman	1999	14.53688007952	0.59239847	2.11320258627442	12.4236774932456
4849	Oman	2000	14.15067531543	0.608421541	2.11557193623195	12.035103379198
4850	Oman	2001	13.97224911425	0.622160627	2.11250291156455	11.8597462026855

TableS2 Frontier analysis of Smoking-Related Low Back Pain among WCBA

	location	year	val	SDI	frontier	eff_diff
4851	Oman	2002	13.96704539111	0.635162126	2.11541692976025	11.8516284613498
4852	Oman	2003	14.03461409491	0.647249093	2.111945301085	11.922668793825
4853	Oman	2004	14.16739893908	0.657626118	2.11066662885158	12.0567323102284
4854	Oman	2005	14.33446983713	0.666401231	2.11329563495542	12.2211742021746
4855	Oman	2006	14.45459119442	0.675353885	2.11301557617937	12.3415756182406
4856	Oman	2007	14.61190054068	0.685055583	2.11008713256746	12.5018134081125
4857	Oman	2008	14.80873199495	0.694952263	2.11211119217973	12.6966208027703
4858	Oman	2009	15.08494908332	0.703098571	2.11281120903071	12.9721378742893
4859	Oman	2010	15.40553087716	0.710688695	2.11572365225158	13.2898072249084
4860	Oman	2011	15.69597797067	0.718264528	2.1106274438326	13.5853505268374
4861	Oman	2012	15.97513940772	0.726450074	2.11349765210623	13.8616417556138
4862	Oman	2013	16.24524599984	0.73343249	2.11801088428424	14.1272351155558
4863	Oman	2014	16.56266609464	0.738743772	2.11085274463515	14.4518133500048
4864	Oman	2015	16.92723017008	0.743597182	2.11314560306169	14.8140845670183
4865	Oman	2016	17.35416070813	0.748797193	2.112853135255	15.241307572875
4866	Oman	2017	17.80229727676	0.753786058	2.11019003042351	15.6921072463365
4867	Oman	2018	18.25550600848	0.759179514	2.11752661330059	16.1379793951794
4868	Oman	2019	18.76772937646	0.764527863	2.1111239534057	16.6566054230543
4869	Oman	2020	19.30076785835	0.768854216	2.11285373451159	17.1879141238384
4870	Oman	2021	19.7269241096	0.773391602	2.11344105490862	17.6134830546914
4871	Pakistan	1990	29.69978792496	0.310467621	2.36987922778565	27.3299086971743
4872	Pakistan	1991	29.66271380566	0.316265667	2.35902725121611	27.3036865544439
4873	Pakistan	1992	29.5832073991	0.322248051	2.35015620453931	27.2330511945607
4874	Pakistan	1993	29.48092794447	0.328219252	2.31581036472096	27.165117579749
4875	Pakistan	1994	29.31965973622	0.334433128	2.28735995339119	27.0322997828288

TableS2 Frontier analysis of Smoking-Related Low Back Pain among WCBA

	location	year	val	SDI	frontier	eff_diff
4876	Pakistan	1995	29.0986049151	0.340926296	2.24580269695155	26.8528022181485
4877	Pakistan	1996	28.85063766628	0.347656203	2.14505587114676	26.7055817951332
4878	Pakistan	1997	28.54272211601	0.354218627	2.11632109409948	26.4264010219105
4879	Pakistan	1998	28.1785925702	0.360875336	2.11683582020258	26.0617567499974
4880	Pakistan	1999	27.76375038311	0.367641397	2.11294105825413	25.6508093248559
4881	Pakistan	2000	27.38205245235	0.374319132	2.11302936976379	25.2690230825862
4882	Pakistan	2001	27.02935451262	0.380593927	2.11240171764817	24.9169527949718
4883	Pakistan	2002	26.73021026549	0.386629992	2.11550547460153	24.6147047908885
4884	Pakistan	2003	26.39304307437	0.392719263	2.11457423757521	24.2784688367948
4885	Pakistan	2004	25.99448801509	0.399084167	2.11557075073759	23.8789172643524
4886	Pakistan	2005	25.63195646871	0.405667437	2.11460457245194	23.5173518962581
4887	Pakistan	2006	25.20664588445	0.41223483	2.11635470325553	23.0902911811945
4888	Pakistan	2007	24.66539244622	0.418519756	2.11421018861864	22.5511822576014
4889	Pakistan	2008	24.09493003031	0.4243482	2.11337009077106	21.9815599395389
4890	Pakistan	2009	23.53456262442	0.430019833	2.1110574928355	21.4235051315845
4891	Pakistan	2010	23.00717889903	0.435640657	2.1132629615855	20.8939159374445
4892	Pakistan	2011	22.65862760519	0.441455585	2.11426042815557	20.5443671770344
4893	Pakistan	2012	22.48842745887	0.447096599	2.11316849518813	20.3752589636819
4894	Pakistan	2013	22.34551422671	0.452823095	2.11532529052983	20.2301889361802
4895	Pakistan	2014	22.15843272245	0.458781424	2.11086610580662	20.0475666166434
4896	Pakistan	2015	21.89807319184	0.464792668	2.11393008074716	19.7841431110928
4897	Pakistan	2016	21.57710778919	0.471022168	2.11204928789	19.4650585013
4898	Pakistan	2017	21.29421135975	0.477544137	2.11174792737928	19.1824634323707
4899	Pakistan	2018	21.02891358057	0.484326128	2.11446089338	18.91445268719
4900	Pakistan	2019	20.82130511486	0.491022981	2.11301322169402	18.708291893166

TableS2 Frontier analysis of Smoking-Related Low Back Pain among WCBA

	location	year	val	SDI	frontier	eff_diff
4901	Pakistan	2020	20.29407548461	0.497364406	2.11343510229714	18.1806403823129
4902	Pakistan	2021	20.09967467965	0.504028689	2.11489812850211	17.9847765511479
4903	Palau	1990	45.83381714653	0.66290951	2.11381700200857	43.7200001445214
4904	Palau	1991	47.10064025093	0.668230731	2.11270529109263	44.9879349598374
4905	Palau	1992	48.20939071302	0.673302462	2.11236076434389	46.0970299486761
4906	Palau	1993	49.2403977527	0.676648715	2.11379222621433	47.1266055264857
4907	Palau	1994	50.53077840815	0.680195775	2.11061711189298	48.420161296257
4908	Palau	1995	51.81702319664	0.68483214	2.11346541826859	49.7035577783714
4909	Palau	1996	52.58443318388	0.690095673	2.11448724934041	50.4699459345396
4910	Palau	1997	53.08323493498	0.695122123	2.11258583799489	50.9706490969851
4911	Palau	1998	53.82638453253	0.69965291	2.11826676738208	51.7081177651479
4912	Palau	1999	54.1868645485	0.703115322	2.114146611973	52.072717936527
4913	Palau	2000	54.25481292887	0.705196945	2.11465943588146	52.1401534929885
4914	Palau	2001	54.53156812213	0.707402386	2.11573603082813	52.4158320913019
4915	Palau	2002	54.63933397493	0.709587644	2.11351625470841	52.5258177202216
4916	Palau	2003	54.4213626747	0.71100022	2.11406274371729	52.3072999309827
4917	Palau	2004	54.02010426346	0.712543811	2.11027901789675	51.9098252455632
4918	Palau	2005	53.29327692676	0.714339831	2.11172415942392	51.1815527673361
4919	Palau	2006	52.51896791027	0.716353431	2.11288930810408	50.4060786021659
4920	Palau	2007	51.66082581103	0.718809542	2.11225062657043	49.5485751844596
4921	Palau	2008	51.07559438725	0.720806623	2.11251646730476	48.9630779199452
4922	Palau	2009	50.49665009297	0.722288267	2.11223140295704	48.384418690013
4923	Palau	2010	50.55441006253	0.724065956	2.11392016319935	48.4404898993307
4924	Palau	2011	50.98663756728	0.726733446	2.11396506033265	48.8726725069474
4925	Palau	2012	51.6684931025	0.729612899	2.11469107250726	49.5538020299927

TableS2 Frontier analysis of Smoking-Related Low Back Pain among WCBA

	location	year	val	SDI	frontier	eff_diff
4926	Palau	2013	52.43179402853	0.731953475	2.11539289921347	50.3164011293165
4927	Palau	2014	53.40275207596	0.734768297	2.11332693470933	51.2894251412507
4928	Palau	2015	54.32992047151	0.738166425	2.11175171189869	52.2181687596113
4929	Palau	2016	55.27573835397	0.741361911	2.1108326810987	53.1649056728713
4930	Palau	2017	55.94722809101	0.744224767	2.11111867387484	53.8361094171352
4931	Palau	2018	56.32506455601	0.74776125	2.1136553740389	54.2114091819711
4932	Palau	2019	56.70992855395	0.750715307	2.1176992769252	54.5922292770248
4933	Palau	2020	56.8201484301	0.752780929	2.11244915503932	54.7076992750607
4934	Palau	2021	56.74750952449	0.754046931	2.11442039821569	54.6330891262743
4935	Palestine	1990	24.71448273067	0.40179221	2.11586743495899	22.598615295711
4936	Palestine	1991	24.77796768553	0.40572715	2.11168354219581	22.6662841433342
4937	Palestine	1992	24.80916068864	0.411146869	2.11340284496478	22.6957578436752
4938	Palestine	1993	24.92886672985	0.416779649	2.11087818323789	22.8179885466121
4939	Palestine	1994	25.06444114616	0.423136673	2.11476199030973	22.9496791558503
4940	Palestine	1995	25.19514103611	0.429307027	2.11434453850492	23.0807964976051
4941	Palestine	1996	25.40836160054	0.434731371	2.11344035493103	23.294921245609
4942	Palestine	1997	25.67266607178	0.440901776	2.11208386560377	23.5605822061762
4943	Palestine	1998	25.67889054632	0.447782415	2.11530253030012	23.5635880160199
4944	Palestine	1999	25.80244824973	0.454909692	2.11248910599681	23.6899591437332
4945	Palestine	2000	25.87613087986	0.460681496	2.11250019286987	23.7636306869901
4946	Palestine	2001	25.90243778375	0.465299102	2.112825473412	23.789612310338
4947	Palestine	2002	25.69285693749	0.468600257	2.11312854278613	23.5797283947039
4948	Palestine	2003	25.48952859631	0.472881732	2.11193483941342	23.3775937568966
4949	Palestine	2004	25.22605045256	0.478527322	2.11081929103649	23.1152311615235
4950	Palestine	2005	24.99303399844	0.485320586	2.11293853228526	22.8800954661547

TableS2 Frontier analysis of Smoking-Related Low Back Pain among WCBA

	location	year	val	SDI	frontier	eff_diff
4951	Palestine	2006	24.78903898907	0.491890407	2.11480246314145	22.6742365259285
4952	Palestine	2007	24.59426146185	0.498978426	2.11141345837758	22.4828480034724
4953	Palestine	2008	24.39836671489	0.506849479	2.1145922974648	22.2837744174252
4954	Palestine	2009	24.18591647724	0.515815836	2.11105855323164	22.0748579240084
4955	Palestine	2010	24.04923807356	0.525711905	2.11182392505411	21.9374141485059
4956	Palestine	2011	23.91240333547	0.53677763	2.11094257041514	21.8014607650549
4957	Palestine	2012	23.82373092113	0.548295425	2.11491695468322	21.7088139664468
4958	Palestine	2013	23.71275023919	0.559163481	2.11788781700661	21.5948624221834
4959	Palestine	2014	23.63235732466	0.569239882	2.11316175223262	21.5191955724274
4960	Palestine	2015	23.65810991922	0.579204261	2.11649892768917	21.5416109915308
4961	Palestine	2016	23.73372978036	0.58949277	2.11525557151101	21.618474208849
4962	Palestine	2017	23.88304904436	0.599157187	2.11286841389785	21.7701806304622
4963	Palestine	2018	24.07485593331	0.608124861	2.11374444575221	21.9611114875578
4964	Palestine	2019	24.27960632953	0.616466839	2.11366745279968	22.1659388767303
4965	Palestine	2020	24.67562116285	0.623810015	2.11379946106098	22.561821701789
4966	Palestine	2021	24.68549057588	0.631011665	2.11316409978052	22.5723264760995
4967	Panama	1990	37.27661245273	0.546048123	2.11258608661718	35.1640263661128
4968	Panama	1991	37.31497803353	0.550484218	2.11346402382	35.20151400971
4969	Panama	1992	37.45107217854	0.554972755	2.11362087331479	35.3374513052252
4970	Panama	1993	37.48315330111	0.559168666	2.11226293270975	35.3708903684003
4971	Panama	1994	37.41939245095	0.561714534	2.11143784832299	35.307954602627
4972	Panama	1995	37.41650100213	0.563353023	2.11234735047684	35.3041536516532
4973	Panama	1996	37.34089257452	0.56531007	2.11937060009618	35.2215219744238
4974	Panama	1997	37.26641676086	0.569395939	2.11309473712944	35.1533220237306
4975	Panama	1998	37.10583381176	0.574697318	2.1161812192151	34.9896525925449

TableS2 Frontier analysis of Smoking-Related Low Back Pain among WCBA

	location	year	val	SDI	frontier	eff_diff
4976	Panama	1999	36.95621113345	0.580432842	2.11146623087449	34.8447449025755
4977	Panama	2000	36.72637542311	0.587378393	2.11664409621043	34.6097313268996
4978	Panama	2001	36.5944685392	0.594535701	2.11453206856366	34.4799364706363
4979	Panama	2002	36.26012131238	0.600138677	2.11257843425746	34.1475428781225
4980	Panama	2003	35.81056961142	0.604043365	2.11837516641	33.69219444501
4981	Panama	2004	35.38872815719	0.60687703	2.11173418246219	33.2769939747278
4982	Panama	2005	34.9702298855	0.609494125	2.1104697072429	32.8597601782571
4983	Panama	2006	34.70261152049	0.612249713	2.1163333593329	32.5862781611571
4984	Panama	2007	34.46335837368	0.615285417	2.11428052803597	32.349077845644
4985	Panama	2008	34.26753760321	0.618958907	2.1106694360092	32.1568681672008
4986	Panama	2009	34.05286571269	0.622402806	2.1149663919029	31.9378993207871
4987	Panama	2010	33.89280738119	0.625116323	2.11544902431562	31.7773583568744
4988	Panama	2011	33.67610199134	0.628633942	2.11383637184362	31.5622656194964
4989	Panama	2012	33.37305454053	0.634263435	2.11540943226761	31.2576451082624
4990	Panama	2013	33.18108755446	0.641324353	2.11364962291597	31.067437931544
4991	Panama	2014	33.02079999861	0.649108174	2.11042200864232	30.9103779899677
4992	Panama	2015	32.96008997697	0.657962047	2.1152614947664	30.8448284822036
4993	Panama	2016	32.93095034091	0.667276758	2.11519093512149	30.8157594057885
4994	Panama	2017	32.88013411103	0.676783503	2.11595010230766	30.7641840087223
4995	Panama	2018	32.72370865644	0.686719925	2.11244392761839	30.6112647288216
4996	Panama	2019	32.43832373408	0.69667068	2.11349570937699	30.324828024703
4997	Panama	2020	31.98994557541	0.70378849	2.11390905955951	29.8760365158505
4998	Panama	2021	32.02788818641	0.708864828	2.11448118414269	29.9134070022673
4999	Papua New Guinea	1990	66.02797442305	0.310668629	2.36855368614062	63.6594207369094
5000	Papua New Guinea	1991	66.76649891923	0.314967305	2.35085184535923	64.4156470738708

TableS2 Frontier analysis of Smoking-Related Low Back Pain among WCBA

	location	year	val	SDI	frontier	eff_diff
5001	Papua New Guinea	1992	67.33357505399	0.319530259	2.34550835325844	64.9880667007316
5002	Papua New Guinea	1993	67.81242169289	0.325005984	2.3140962777879	65.4983254151021
5003	Papua New Guinea	1994	68.36426279662	0.330664468	2.30263924789352	66.0616235487265
5004	Papua New Guinea	1995	68.7761292113	0.335421285	2.28377848914812	66.4923507221519
5005	Papua New Guinea	1996	69.62047301022	0.340348698	2.24251160470833	67.3779614055117
5006	Papua New Guinea	1997	69.77210709903	0.344258325	2.17076154165049	67.6013455573795
5007	Papua New Guinea	1998	70.43638394576	0.347801408	2.13335627044779	68.3030276753122
5008	Papua New Guinea	1999	71.46345600024	0.351250621	2.12641100412015	69.3370449961199
5009	Papua New Guinea	2000	72.88285962893	0.354135791	2.12485913334017	70.7580004955898
5010	Papua New Guinea	2001	73.51813390113	0.356588986	2.11480261195491	71.4033312891751
5011	Papua New Guinea	2002	73.9482022231	0.358572086	2.12134270653641	71.8268595165636
5012	Papua New Guinea	2003	74.57602336132	0.360736816	2.1120445086859	72.4639788526341
5013	Papua New Guinea	2004	74.90572728745	0.362827542	2.11041068273225	72.7953166047178
5014	Papua New Guinea	2005	75.20270328501	0.365294965	2.11785802453843	73.0848452604716
5015	Papua New Guinea	2006	75.25340038398	0.367826759	2.11567033552259	73.1377300484574
5016	Papua New Guinea	2007	74.86951356545	0.370794147	2.11336844410623	72.7561451213438
5017	Papua New Guinea	2008	74.33843099519	0.373426209	2.11546784923846	72.2229631459515
5018	Papua New Guinea	2009	73.62088410639	0.376528285	2.11489399215466	71.5059901142353
5019	Papua New Guinea	2010	73.27402304637	0.380286778	2.11627548972808	71.1577475566419
5020	Papua New Guinea	2011	73.21696258651	0.383587539	2.11499276233905	71.101969824171
5021	Papua New Guinea	2012	73.40714435136	0.386781028	2.11301516654868	71.2941291848113
5022	Papua New Guinea	2013	73.91957658638	0.389926437	2.11662886025288	71.8029477261271
5023	Papua New Guinea	2014	74.57503553986	0.394004571	2.11507078288891	72.4599647569711
5024	Papua New Guinea	2015	75.26438742161	0.398285864	2.11453306171677	73.1498543598932
5025	Papua New Guinea	2016	76.00745889877	0.402407267	2.11186317861648	73.8955957201535

TableS2 Frontier analysis of Smoking-Related Low Back Pain among WCBA

	location	year	val	SDI	frontier	eff_diff
5026	Papua New Guinea	2017	76.816073516	0.406153435	2.11433734194522	74.7017361740548
5027	Papua New Guinea	2018	77.4567559646	0.409165083	2.11490658056301	75.341849384037
5028	Papua New Guinea	2019	77.98688496202	0.412311147	2.11244091257571	75.8744440494443
5029	Papua New Guinea	2020	77.6941873069	0.415186879	2.11316210269657	75.5810252042034
5030	Papua New Guinea	2021	78.49686860151	0.417797443	2.11028411329423	76.3865844882158
5031	Paraguay	1990	78.09258793227	0.469527785	2.11671961737	75.9758683149
5032	Paraguay	1991	78.84792128178	0.474352286	2.11695372599684	76.7309675557832
5033	Paraguay	1992	79.33468245117	0.479314169	2.11552299717169	77.2191594539983
5034	Paraguay	1993	79.49754728187	0.4846142	2.11390653771262	77.3836407441574
5035	Paraguay	1994	79.40923898168	0.490226935	2.11568603837647	77.2935529433035
5036	Paraguay	1995	79.04255305732	0.496336876	2.11351576208091	76.9290372952391
5037	Paraguay	1996	78.69631719998	0.502322804	2.1143800914536	76.5819371085264
5038	Paraguay	1997	78.05503128616	0.508286948	2.11225746953825	75.9427738166218
5039	Paraguay	1998	77.0418204076	0.513819896	2.11208415178235	74.9297362558177
5040	Paraguay	1999	75.97850680682	0.518720583	2.11343624102295	73.865070565797
5041	Paraguay	2000	74.98450057091	0.523007793	2.11361264812875	72.8708879227813
5042	Paraguay	2001	73.36956607497	0.526916763	2.11125656623793	71.2583095087321
5043	Paraguay	2002	71.58341681103	0.531177358	2.11375721780581	69.4696595932242
5044	Paraguay	2003	69.90526840052	0.535360126	2.1131620527618	67.7921063477582
5045	Paraguay	2004	68.410385606	0.539556655	2.11186184406258	66.2985237619374
5046	Paraguay	2005	67.14220395438	0.543622697	2.11111782986525	65.0310861245147
5047	Paraguay	2006	66.18652072253	0.547943305	2.11269191685722	64.0738288056728
5048	Paraguay	2007	65.36081985016	0.552758676	2.11159451339704	63.249225336763
5049	Paraguay	2008	64.69956198402	0.558016903	2.11314515017639	62.5864168338436
5050	Paraguay	2009	64.02969628849	0.562900059	2.115480346991	61.914215941499

TableS2 Frontier analysis of Smoking-Related Low Back Pain among WCBA

	location	year	val	SDI	frontier	eff_diff
5051	Paraguay	2010	63.58542668752	0.568846562	2.11154413609524	61.4738825514248
5052	Paraguay	2011	63.04173250907	0.574954463	2.11291142975203	60.928821079318
5053	Paraguay	2012	62.49345147906	0.580650186	2.11627243771454	60.3771790413455
5054	Paraguay	2013	61.95619699962	0.587247042	2.11428218976	59.84191480986
5055	Paraguay	2014	61.62806398181	0.593953153	2.11096022904657	59.5171037527634
5056	Paraguay	2015	61.47179455204	0.600484125	2.11261223634429	59.3591823156957
5057	Paraguay	2016	61.44961427249	0.607018145	2.11545351150732	59.3341607609827
5058	Paraguay	2017	61.33141916686	0.613581591	2.11758040226169	59.2138387645983
5059	Paraguay	2018	61.31510998578	0.620032362	2.11245443959227	59.2026555461877
5060	Paraguay	2019	61.58957581607	0.626070139	2.11369089371909	59.4758849223509
5061	Paraguay	2020	62.2455491658	0.631057689	2.1159664373477	60.1295827284523
5062	Paraguay	2021	62.90131540698	0.635718099	2.11255710765518	60.7887582993248
5063	Peru	1990	15.85027934186	0.510419852	2.11267892941643	13.7376004124436
5064	Peru	1991	16.26136563508	0.512161525	2.11242882648216	14.1489368085978
5065	Peru	1992	16.68737246167	0.513821552	2.11381046708859	14.5735619945814
5066	Peru	1993	17.10922002288	0.515981506	2.11631498739838	14.9929050354816
5067	Peru	1994	17.45419160968	0.51930608	2.11942364515919	15.3347679645208
5068	Peru	1995	17.80720578945	0.523640527	2.11169989917568	15.6955058902743
5069	Peru	1996	18.20654672556	0.528312042	2.11465768004882	16.0918890455112
5070	Peru	1997	18.56534838982	0.533576491	2.11259764721794	16.4527507426021
5071	Peru	1998	18.94153664456	0.538721717	2.11662382981173	16.8249128147483
5072	Peru	1999	19.24195422864	0.543853377	2.11053755126204	17.131416677378
5073	Peru	2000	19.47676105687	0.548952201	2.11248096421261	17.3642800926574
5074	Peru	2001	19.53171000677	0.553775453	2.11470874946203	17.417001257308
5075	Peru	2002	19.41121496701	0.558853263	2.11367215073792	17.2975428162721

TableS2 Frontier analysis of Smoking-Related Low Back Pain among WCBA

	location	year	val	SDI	frontier	eff_diff
5076	Peru	2003	19.17201440751	0.563871072	2.11273510433083	17.0592793031792
5077	Peru	2004	18.88628086139	0.569063024	2.11009373878447	16.7761871226055
5078	Peru	2005	18.76045259233	0.574517001	2.11385715587444	16.6465954364556
5079	Peru	2006	18.72569781804	0.580503365	2.11404830390548	16.6116495141345
5080	Peru	2007	18.71590131456	0.586958489	2.11244522297851	16.6034560915815
5081	Peru	2008	18.75943069569	0.593750811	2.11540456184526	16.6440261338447
5082	Peru	2009	18.75778214499	0.599954993	2.11364463456167	16.6441375104283
5083	Peru	2010	18.82372158086	0.606520932	2.11490391102941	16.7088176698306
5084	Peru	2011	18.86807823849	0.613051221	2.109618669436	16.758459569054
5085	Peru	2012	18.94945165568	0.619355264	2.11212385813721	16.8373277975428
5086	Peru	2013	19.04364904457	0.625519024	2.11189869703926	16.9317503475307
5087	Peru	2014	19.19982270868	0.631114354	2.11129285141685	17.0885298572631
5088	Peru	2015	19.41019727912	0.636278271	2.11301615450774	17.2971811246123
5089	Peru	2016	19.72791660974	0.641285279	2.11340480151	17.61451180823
5090	Peru	2017	20.12942851847	0.646180855	2.11425891742485	18.0151696010452
5091	Peru	2018	20.49723964834	0.650977317	2.11216039531018	18.3850792530298
5092	Peru	2019	20.76810472855	0.655433735	2.11359200396738	18.6545127245826
5093	Peru	2020	20.86742867443	0.658672244	2.11306088773479	18.7543677866952
5094	Peru	2021	21.04153787677	0.662054037	2.11281189657711	18.9287259801929
5095	Philippines	1990	52.08388819103	0.510011796	2.11414643950081	49.9697417515292
5096	Philippines	1991	52.06308978857	0.513707183	2.11311813896078	49.9499716496092
5097	Philippines	1992	51.99964845118	0.517109502	2.11618905493012	49.8834593962499
5098	Philippines	1993	52.0486343025	0.520600378	2.11727516317232	49.9313591393277
5099	Philippines	1994	52.11418128632	0.524613607	2.11572580446866	49.9984554818513
5100	Philippines	1995	52.16001591877	0.528827907	2.11186874098468	50.0481471777853

TableS2 Frontier analysis of Smoking-Related Low Back Pain among WCBA

	location	year	val	SDI	frontier	eff_diff
5101	Philippines	1996	52.065246005	0.532938849	2.11438688276055	49.9508591222395
5102	Philippines	1997	51.86199773509	0.536589868	2.11249595315354	49.7495017819365
5103	Philippines	1998	51.53912706626	0.53949647	2.11242433535712	49.4267027309029
5104	Philippines	1999	51.27556555068	0.542257968	2.11045580917714	49.1651097415029
5105	Philippines	2000	51.1452441727	0.544997723	2.11346993656021	49.0317742361398
5106	Philippines	2001	51.0532183335	0.547409432	2.11656043764286	48.9366578958571
5107	Philippines	2002	50.79107021034	0.549521153	2.11200840946872	48.6790618008713
5108	Philippines	2003	50.36374753834	0.551567968	2.11577010832734	48.2479774300127
5109	Philippines	2004	49.79701293903	0.553704287	2.11326069724299	47.683752241787
5110	Philippines	2005	49.16844633527	0.555664103	2.11509449825481	47.0533518370152
5111	Philippines	2006	48.35562429446	0.557976265	2.11413469991459	46.2414895945454
5112	Philippines	2007	47.27431704146	0.560822024	2.11389600105027	45.1604210404097
5113	Philippines	2008	46.05011123451	0.563974721	2.11465919459026	43.9354520399197
5114	Philippines	2009	44.77650846372	0.56735582	2.11308206187984	42.6634264018402
5115	Philippines	2010	43.55026526947	0.571765888	2.12051955997155	41.4297457094984
5116	Philippines	2011	42.20107766072	0.577240283	2.11344791051531	40.0876297502047
5117	Philippines	2012	40.59969377902	0.583767625	2.1123387879674	38.4873549910526
5118	Philippines	2013	38.94834343963	0.590837549	2.11281552074789	36.8355279188821
5119	Philippines	2014	37.4697254684	0.59838452	2.1182173372403	35.3515081311597
5120	Philippines	2015	36.35545679844	0.606215624	2.11408357886012	34.2413732195799
5121	Philippines	2016	35.63840014633	0.614224961	2.11592262406734	33.5224775222627
5122	Philippines	2017	35.16691786289	0.622315029	2.11237666411977	33.0545411987702
5123	Philippines	2018	34.82361328803	0.630464276	2.11550707700429	32.7081062110257
5124	Philippines	2019	34.52293615532	0.638547384	2.1119978173047	32.4109383380153
5125	Philippines	2020	34.11051771409	0.644867233	2.11134888669725	31.9991688273928

TableS2 Frontier analysis of Smoking-Related Low Back Pain among WCBA

	location	year	val	SDI	frontier	eff_diff
5126	Philippines	2021	33.93163199155	0.651219329	2.112925144337	31.818706847213
5127	Poland	1990	419.41674844186	0.627227888	2.11279469572652	417.303953746134
5128	Poland	1991	421.23358338558	0.632671471	2.11028825821158	419.123295127368
5129	Poland	1992	422.79301302947	0.640661793	2.11227488399329	420.680738145477
5130	Poland	1993	423.74774629788	0.6489505	2.11572262049738	421.632023677383
5131	Poland	1994	423.23317383713	0.658010514	2.11399527967883	421.119178557451
5132	Poland	1995	422.05194037664	0.666678867	2.11476316099781	419.937177215642
5133	Poland	1996	416.83794247337	0.674378829	2.11333508229869	414.724607391071
5134	Poland	1997	407.37011848708	0.682587612	2.11415447239221	405.255964014688
5135	Poland	1998	396.27042787571	0.690805671	2.11281563988107	394.157612235829
5136	Poland	1999	385.43360374166	0.698536056	2.11201075155	383.32159299011
5137	Poland	2000	376.34182821725	0.706662052	2.11146970963661	374.230358507613
5138	Poland	2001	368.6611646868	0.714831304	2.11323229078956	366.54793239601
5139	Poland	2002	361.54516930752	0.722009458	2.11799612597438	359.427173181546
5140	Poland	2003	355.06332446332	0.728155254	2.11207634997492	352.951248113345
5141	Poland	2004	349.30873090826	0.733673192	2.11305677166607	347.195674136594
5142	Poland	2005	344.25721778132	0.738428767	2.11492837474192	342.142289406578
5143	Poland	2006	339.90828111266	0.742363709	2.11263880180324	337.795642310857
5144	Poland	2007	335.80751155104	0.746008817	2.11545961136528	333.692051939675
5145	Poland	2008	332.45435834196	0.750521426	2.11255358062561	330.341804761334
5146	Poland	2009	330.44407451336	0.756427378	2.11404002538564	328.330034487974
5147	Poland	2010	329.95163863701	0.763184974	2.1138989345771	327.837739702433
5148	Poland	2011	329.96181866572	0.769407164	2.11211270111833	327.849705964602
5149	Poland	2012	329.48566352222	0.774680767	2.11517537287648	327.370488149344
5150	Poland	2013	329.20613229832	0.779434257	2.11495740655106	327.091174891769

TableS2 Frontier analysis of Smoking-Related Low Back Pain among WCBA

	location	year	val	SDI	frontier	eff_diff
5151	Poland	2014	329.62973556425	0.783682369	2.11402826485358	327.515707299396
5152	Poland	2015	331.0155206002	0.787445343	2.11056769868917	328.904952901511
5153	Poland	2016	332.72580596836	0.790651015	2.116347387335	330.609458581025
5154	Poland	2017	333.79418467277	0.794530345	2.11396464424807	331.680220028522
5155	Poland	2018	335.01376841041	0.799478982	2.11665799737522	332.897110413035
5156	Poland	2019	337.15435166993	0.804791332	2.11394423055453	335.040407439375
5157	Poland	2020	339.33519107444	0.808795318	2.11503618341112	337.220154891029
5158	Poland	2021	339.46501514597	0.812042809	2.11452988290488	337.350485263065
5159	Portugal	1990	157.87834106152	0.599777757	2.11123870744329	155.767102354077
5160	Portugal	1991	156.71752152958	0.607269449	2.1152511009392	154.602270428641
5161	Portugal	1992	156.50507910476	0.61480808	2.11313471705452	154.391944387705
5162	Portugal	1993	156.26486178061	0.622101177	2.11758994790446	154.147271832706
5163	Portugal	1994	156.49503523088	0.629306387	2.11086098443818	154.384174246442
5164	Portugal	1995	157.41521838636	0.635498761	2.11436343339101	155.300854952969
5165	Portugal	1996	159.41964110123	0.640864526	2.11260976995329	157.307031331277
5166	Portugal	1997	162.83157537648	0.646192904	2.11043332738634	160.721142049094
5167	Portugal	1998	166.77101339658	0.651335994	2.11537493267013	164.65563846391
5168	Portugal	1999	171.12188288236	0.656120667	2.1120013822446	169.009881500115
5169	Portugal	2000	175.31913883362	0.661610852	2.11424881417222	173.204890019448
5170	Portugal	2001	180.27740224985	0.667247151	2.1163849486012	178.161017301249
5171	Portugal	2002	186.37385175336	0.67258394	2.11674255073957	184.25710920262
5172	Portugal	2003	193.19061847893	0.677663412	2.1131262037203	191.07749227521
5173	Portugal	2004	199.96078285059	0.682352926	2.11432825037856	197.846454600211
5174	Portugal	2005	206.49452345015	0.686887105	2.11273244765948	204.381791002491
5175	Portugal	2006	212.37653516983	0.691158795	2.11795030511137	210.258584864719

TableS2 Frontier analysis of Smoking-Related Low Back Pain among WCBA

	location	year	val	SDI	frontier	eff_diff
5176	Portugal	2007	217.46595946207	0.694974853	2.11247783416468	215.353481627905
5177	Portugal	2008	222.23540831317	0.698604599	2.11461107353162	220.120797239638
5178	Portugal	2009	225.96212732625	0.702223399	2.11412088543175	223.848006440818
5179	Portugal	2010	229.6211822286	0.706563555	2.11499704104553	227.506185187554
5180	Portugal	2011	233.73225318875	0.711262893	2.11309222091318	231.619160967837
5181	Portugal	2012	238.25387465333	0.715804084	2.11539361618041	236.13848103715
5182	Portugal	2013	242.28239110073	0.719836991	2.11242215266187	240.169968948068
5183	Portugal	2014	245.89037915303	0.723021282	2.11673148430742	243.773647668723
5184	Portugal	2015	248.51631467183	0.72575622	2.11405001874295	246.402264653087
5185	Portugal	2016	250.43262141477	0.728300849	2.11186107944696	248.320760335323
5186	Portugal	2017	251.84429029052	0.731225535	2.11461883329533	249.729671457225
5187	Portugal	2018	252.75581660759	0.734402947	2.11124417415897	250.644572433431
5188	Portugal	2019	252.03381377612	0.738090481	2.11335939940482	249.920454376715
5189	Portugal	2020	247.72816517506	0.741037384	2.11235793422896	245.615807240831
5190	Portugal	2021	246.048208607	0.744151851	2.11184033842125	243.936368268579
5191	Puerto Rico	1990	70.55189938361	0.658758146	2.11375401606966	68.4381453675404
5192	Puerto Rico	1991	71.25692846574	0.66326172	2.11277776012966	69.1441507056103
5193	Puerto Rico	1992	71.64441070584	0.667272192	2.11306386642284	69.5313468394172
5194	Puerto Rico	1993	71.89946061791	0.671024105	2.11697944845849	69.7824811694515
5195	Puerto Rico	1994	72.20582010068	0.67545081	2.11564613299112	70.0901739676889
5196	Puerto Rico	1995	72.43030153645	0.68067395	2.11340412614609	70.3168974103039
5197	Puerto Rico	1996	72.49331541235	0.685865893	2.11420954288082	70.3791058694692
5198	Puerto Rico	1997	72.23329069872	0.691274382	2.1130343986289	70.1202563000911
5199	Puerto Rico	1998	72.0113028501	0.697040445	2.11338534522385	69.8979175048762
5200	Puerto Rico	1999	71.63163994817	0.702850671	2.11434732746611	69.5172926207039

TableS2 Frontier analysis of Smoking-Related Low Back Pain among WCBA

	location	year	val	SDI	frontier	eff_diff
5201	Puerto Rico	2000	71.75521627387	0.710035652	2.11523441240344	69.6399818614666
5202	Puerto Rico	2001	71.39131132853	0.718955861	2.11410686510128	69.2772044634287
5203	Puerto Rico	2002	71.23878024765	0.725956856	2.11615650513213	69.1226237425179
5204	Puerto Rico	2003	71.13560049854	0.730475138	2.11394175509573	69.0216587434443
5205	Puerto Rico	2004	70.99431730284	0.734219546	2.11113721358705	68.883180089253
5206	Puerto Rico	2005	70.97941156427	0.737742266	2.11513234691727	68.8642792173527
5207	Puerto Rico	2006	71.16151515068	0.741629431	2.11103469929107	69.0504804513889
5208	Puerto Rico	2007	71.25590233688	0.745883807	2.11700711172109	69.1388952251589
5209	Puerto Rico	2008	71.27585440435	0.750922167	2.11311276799627	69.1627416363537
5210	Puerto Rico	2009	71.40107840422	0.756361695	2.11226542191568	69.2888129823043
5211	Puerto Rico	2010	71.26638566522	0.761763253	2.11556587903717	69.1508197861828
5212	Puerto Rico	2011	71.22109240577	0.766784882	2.11413336197095	69.1069590437991
5213	Puerto Rico	2012	71.10501006996	0.772311804	2.11549068260634	68.9895193873537
5214	Puerto Rico	2013	70.55094482902	0.778461162	2.11135778669171	68.4395870423283
5215	Puerto Rico	2014	70.04390357332	0.785120345	2.11321199846781	67.9306915748522
5216	Puerto Rico	2015	69.70316345523	0.791800167	2.11531204423187	67.5878514109981
5217	Puerto Rico	2016	69.51888618592	0.799084083	2.11274994954257	67.4061362363774
5218	Puerto Rico	2017	69.96908756411	0.806648961	2.11426240791564	67.8548251561944
5219	Puerto Rico	2018	70.52725043463	0.812906066	2.11458425799046	68.4126661766395
5220	Puerto Rico	2019	70.50160731163	0.818843191	2.11526098729467	68.3863463243353
5221	Puerto Rico	2020	70.65520499218	0.822918436	2.110995785712	68.544209206468
5222	Puerto Rico	2021	70.53391063466	0.825525847	2.11415663956154	68.4197539950984
5223	Qatar	1990	20.59117391421	0.651208376	2.11471343621926	18.4764604779907
5224	Qatar	1991	21.03017574468	0.655338767	2.11174055361037	18.9184351910696
5225	Qatar	1992	21.34226331861	0.660673763	2.11763494789098	19.224628370719

TableS2 Frontier analysis of Smoking-Related Low Back Pain among WCBA

	location	year	val	SDI	frontier	eff_diff
5226	Qatar	1993	21.63894028127	0.666194399	2.11241466386703	19.526525617403
5227	Qatar	1994	21.89930516691	0.672025384	2.11685145196	19.78245371495
5228	Qatar	1995	22.13190822902	0.678320339	2.11164629962691	20.0202619293931
5229	Qatar	1996	22.25591442962	0.684835919	2.112276863162	20.143637566458
5230	Qatar	1997	22.35622717843	0.693109654	2.11459407464803	20.241633103782
5231	Qatar	1998	22.35680618543	0.701409064	2.11296295714985	20.2438432282801
5232	Qatar	1999	22.34447196609	0.708880981	2.11171289890953	20.2327590671805
5233	Qatar	2000	22.24605346292	0.714831609	2.11290381200467	20.1331496509153
5234	Qatar	2001	22.17547382629	0.720831064	2.11494729527463	20.0605265310154
5235	Qatar	2002	22.0612296083	0.727112174	2.11256152300905	19.948668085291
5236	Qatar	2003	21.88425529027	0.733536599	2.11655065499452	19.7677046352755
5237	Qatar	2004	21.63303238175	0.740223208	2.11556109341039	19.5174712883396
5238	Qatar	2005	21.25131594006	0.747189359	2.1153192956325	19.1359966444275
5239	Qatar	2006	20.87155285475	0.754123028	2.11597554973582	18.7555773050142
5240	Qatar	2007	20.57746336143	0.760960134	2.11420216130623	18.4632612001238
5241	Qatar	2008	20.33448018295	0.767659233	2.11208599261193	18.2223941903381
5242	Qatar	2009	20.19628845158	0.774276849	2.11442023596716	18.0818682156128
5243	Qatar	2010	20.16402392787	0.780807074	2.11462321276988	18.0494007151001
5244	Qatar	2011	20.33567023244	0.78721245	2.11530197984205	18.2203682525979
5245	Qatar	2012	20.58748084562	0.793494901	2.11582783850122	18.4716530071188
5246	Qatar	2013	20.92800493756	0.799611555	2.11559731208269	18.8124076254773
5247	Qatar	2014	21.27607873029	0.80572571	2.1141615711859	19.1619171591041
5248	Qatar	2015	21.59900078578	0.811872852	2.11175018941662	19.4872505963634
5249	Qatar	2016	21.98528189675	0.817979841	2.1144838874915	19.8707980092585
5250	Qatar	2017	22.37223057544	0.824016868	2.11567980524067	20.2565507701993

TableS2 Frontier analysis of Smoking-Related Low Back Pain among WCBA

	location	year	val	SDI	frontier	eff_diff
5251	Qatar	2018	22.76218556759	0.829962127	2.11546316289461	20.6467224046954
5252	Qatar	2019	23.16575024214	0.835805284	2.11226808382097	21.053482158319
5253	Qatar	2020	23.5633434761	0.841467395	2.11304612112186	21.4502973549781
5254	Qatar	2021	23.90308420134	0.846860584	2.11158732467094	21.7914968766691
5255	Romania	1990	291.29894003276	0.619298862	2.11304393188925	289.185896100871
5256	Romania	1991	287.74722335005	0.626795627	2.11205772818246	285.635165621868
5257	Romania	1992	285.15623687515	0.630475082	2.11274436304382	283.043492512106
5258	Romania	1993	282.86165568192	0.633801422	2.11385938861438	280.747796293306
5259	Romania	1994	281.12583959607	0.638371932	2.11534609984675	279.010493496223
5260	Romania	1995	279.34533513415	0.643718879	2.11300734398262	277.232327790167
5261	Romania	1996	278.60830468009	0.648104195	2.11260394212076	276.495700737969
5262	Romania	1997	279.31518280821	0.651225576	2.1185711962866	277.196611611923
5263	Romania	1998	286.45513667837	0.654927769	2.11225331952639	284.342883358844
5264	Romania	1999	291.95375303342	0.659544846	2.11463067346694	289.839122359953
5265	Romania	2000	293.55593611862	0.665294346	2.11429135593416	291.441644762686
5266	Romania	2001	293.97840533641	0.672020167	2.11280298712457	291.865602349285
5267	Romania	2002	293.18058769354	0.678297417	2.11347176688796	291.067115926652
5268	Romania	2003	292.26905686578	0.684074929	2.11468197074416	290.154374895036
5269	Romania	2004	291.99784391662	0.690417055	2.11439732942951	289.883446587191
5270	Romania	2005	291.84308867788	0.69499895	2.11610677303356	289.726981904846
5271	Romania	2006	292.09664965937	0.699490766	2.11299749746654	289.983652161903
5272	Romania	2007	291.89308530651	0.704523727	2.113762268455	289.779323038055
5273	Romania	2008	291.85383215554	0.710757128	2.11157685087045	289.74225530467
5274	Romania	2009	293.43698069929	0.717445122	2.11668195164814	291.320298747642
5275	Romania	2010	296.64815237451	0.723891349	2.11250832265862	294.535644051851

TableS2 Frontier analysis of Smoking-Related Low Back Pain among WCBA

	location	year	val	SDI	frontier	eff_diff
5276	Romania	2011	302.48161167162	0.729018272	2.1122469469091	300.369364724711
5277	Romania	2012	310.11506973385	0.733892482	2.11213165778304	308.002938076067
5278	Romania	2013	319.64487318596	0.737880473	2.11511916705603	317.529754018904
5279	Romania	2014	329.04202049561	0.740395467	2.11364376120047	326.92837673441
5280	Romania	2015	338.74599460595	0.74347353	2.11166869130429	336.634325914646
5281	Romania	2016	348.6259943756	0.74705871	2.11338298253517	346.512611393065
5282	Romania	2017	356.67843563106	0.751117367	2.1125482596139	354.565887371446
5283	Romania	2018	359.2542457858	0.755533333	2.11315426265582	357.141091523144
5284	Romania	2019	360.98964618569	0.760283971	2.11420926470979	358.87543692098
5285	Romania	2020	359.39185232016	0.764276444	2.11150603016288	357.280346289997
5286	Romania	2021	357.82340451838	0.768453864	2.11798043892164	355.705424079458
5287	Russia	1990	125.83792089	0.671600578	2.11347792738729	123.724442962613
5288	Russia	1991	125.10590940559	0.680099211	2.11575358776971	122.99015581782
5289	Russia	1992	125.42282238012	0.688068022	2.11396578185629	123.308856598264
5290	Russia	1993	127.51707123733	0.692036194	2.11515413778577	125.401917099544
5291	Russia	1994	130.57976767127	0.694144966	2.11531435198697	128.464453319283
5292	Russia	1995	133.9777697072	0.697854214	2.11353564775	131.86423405945
5293	Russia	1996	138.79243413866	0.701468231	2.11215465658868	136.680279482071
5294	Russia	1997	144.90050092323	0.703752867	2.11454780715928	142.785953116071
5295	Russia	1998	152.07880363713	0.705198518	2.11248566172955	149.9663179754
5296	Russia	1999	158.92435242748	0.706863538	2.11162403819987	156.81272838928
5297	Russia	2000	164.4104103045	0.708643648	2.11390280891333	162.296507495587
5298	Russia	2001	169.75950845963	0.710570359	2.11124168357692	167.648266776053
5299	Russia	2002	175.89697428513	0.713460957	2.11665206237288	173.780322222757
5300	Russia	2003	182.10768641132	0.718875116	2.11354488696726	179.994141524353

TableS2 Frontier analysis of Smoking-Related Low Back Pain among WCBA

	location	year	val	SDI	frontier	eff_diff
5301	Russia	2004	187.75170251848	0.726305319	2.11154637741959	185.64015614106
5302	Russia	2005	192.33368487023	0.733592777	2.11411703982513	190.219567830405
5303	Russia	2006	197.10995086546	0.740064664	2.11293292717109	194.997017938289
5304	Russia	2007	202.85228215543	0.746191512	2.11126720186791	200.741014953562
5305	Russia	2008	208.58656453374	0.752623243	2.114864074085	206.471700459655
5306	Russia	2009	213.41340582595	0.757960405	2.11441492899443	211.298990896956
5307	Russia	2010	216.68738153593	0.763320116	2.11425539972609	214.573126136204
5308	Russia	2011	218.68758664316	0.767633588	2.11121113045957	216.5763755127
5309	Russia	2012	220.52806731409	0.771920384	2.11446248676836	218.413604827322
5310	Russia	2013	222.56910108114	0.776526979	2.11745653869689	220.451644542443
5311	Russia	2014	224.89083238968	0.780617524	2.1113301130995	222.77950227658
5312	Russia	2015	227.60619997013	0.784913774	2.11456319803485	225.491636772095
5313	Russia	2016	230.18019451932	0.790330149	2.11213759227731	228.068056927043
5314	Russia	2017	232.01703102329	0.795669484	2.11364998235529	229.903381040935
5315	Russia	2018	233.29111557676	0.800108144	2.1124631507629	231.178652425997
5316	Russia	2019	234.49603297718	0.803658201	2.11629973696095	232.379733240219
5317	Russia	2020	235.01409338359	0.806011474	2.11295847250325	232.901134911087
5318	Russia	2021	233.57386464214	0.808536005	2.1102020247875	231.463662617352
5319	Rwanda	1990	27.40718280743	0.27509719	2.44855909713917	24.9586237102908
5320	Rwanda	1991	28.25930219704	0.276194698	2.455330914855	25.803971282185
5321	Rwanda	1992	28.99489537398	0.277178171	2.45826236125923	26.5366330127208
5322	Rwanda	1993	29.65567339453	0.276980133	2.4431699607841	27.2125034337459
5323	Rwanda	1994	30.46610723779	0.272331258	2.50263576552525	27.9634714722648
5324	Rwanda	1995	31.01712544301	0.271102227	2.46958224484553	28.5475431981645
5325	Rwanda	1996	31.23770850796	0.271097922	2.48079776723564	28.7569107407244

TableS2 Frontier analysis of Smoking-Related Low Back Pain among WCBA

	location	year	val	SDI	frontier	eff_diff
5326	Rwanda	1997	31.39898127639	0.272455861	2.47702414278154	28.9219571336085
5327	Rwanda	1998	31.48350070837	0.274582551	2.46114978290254	29.0223509254675
5328	Rwanda	1999	31.37808810567	0.277014387	2.46253781551267	28.9155502901573
5329	Rwanda	2000	31.12234286727	0.280983454	2.45496377548074	28.6673790917893
5330	Rwanda	2001	30.5984604818	0.286082055	2.43290885270899	28.165551629091
5331	Rwanda	2002	29.97815719962	0.29295543	2.42889603729671	27.5492611623233
5332	Rwanda	2003	29.2815586692	0.299676918	2.41268368634495	26.8688749828551
5333	Rwanda	2004	28.66223150645	0.306943916	2.38586583794089	26.2763656685091
5334	Rwanda	2005	28.15649375434	0.314219578	2.36449277905045	25.7920009752895
5335	Rwanda	2006	27.74229392169	0.321998519	2.34791668895787	25.3943772327321
5336	Rwanda	2007	27.26654617691	0.329616591	2.30723635789075	24.9593098190193
5337	Rwanda	2008	26.77642314023	0.337039226	2.27362188274656	24.5028012574834
5338	Rwanda	2009	26.46664360117	0.344494633	2.16218180486578	24.3044617963042
5339	Rwanda	2010	26.24254537708	0.351659416	2.11606532638177	24.1264800506982
5340	Rwanda	2011	25.97869716981	0.35894034	2.1237782888661	23.8549188809439
5341	Rwanda	2012	25.67510087547	0.366411821	2.11344593140588	23.5616549440641
5342	Rwanda	2013	25.42977620627	0.373769682	2.11485212415754	23.3149240821125
5343	Rwanda	2014	25.29907243317	0.381533179	2.11350800541847	23.1855644277515
5344	Rwanda	2015	25.24704391483	0.389895415	2.11459699532783	23.1324469195022
5345	Rwanda	2016	25.30210636087	0.398287959	2.11270408236357	23.1894022785064
5346	Rwanda	2017	25.35645307975	0.406411475	2.11081147206164	23.2456416076884
5347	Rwanda	2018	25.38226124444	0.414657912	2.11554382635674	23.2667174180833
5348	Rwanda	2019	25.50065398788	0.423086727	2.11233161614607	23.3883223717339
5349	Rwanda	2020	25.54327048443	0.429896596	2.11302172889212	23.4302487555379
5350	Rwanda	2021	25.37041103343	0.435588706	2.11918549636118	23.2512255370688

TableS2 Frontier analysis of Smoking-Related Low Back Pain among WCBA

	location	year	val	SDI	frontier	eff_diff
5351	Saint Kitts and Nevis	1990	17.0560900029	0.580685877	2.11317337661929	14.9429166262807
5352	Saint Kitts and Nevis	1991	17.69063992086	0.59025495	2.11594845809871	15.5746914627613
5353	Saint Kitts and Nevis	1992	18.36746220259	0.59918719	2.11209877760603	16.255363424984
5354	Saint Kitts and Nevis	1993	19.01527177687	0.607235892	2.11213425354482	16.9031375233252
5355	Saint Kitts and Nevis	1994	19.69167654491	0.614514658	2.11570770496384	17.5759688399462
5356	Saint Kitts and Nevis	1995	20.27435741276	0.620716498	2.11356950197859	18.1607879107814
5357	Saint Kitts and Nevis	1996	20.82794879896	0.626207411	2.11041818488769	18.7175306140723
5358	Saint Kitts and Nevis	1997	21.40453602672	0.63127474	2.1136831412798	19.2908528854402
5359	Saint Kitts and Nevis	1998	21.9552537567	0.635539988	2.11207352091192	19.8431802357881
5360	Saint Kitts and Nevis	1999	22.54921778273	0.639713739	2.11258696507119	20.4366308176588
5361	Saint Kitts and Nevis	2000	23.20837725515	0.644353377	2.11210867960027	21.0962685755497
5362	Saint Kitts and Nevis	2001	23.51456322166	0.649226653	2.11260137013083	21.4019618515292
5363	Saint Kitts and Nevis	2002	23.50489635861	0.654593409	2.11051850478618	21.3943778538238
5364	Saint Kitts and Nevis	2003	23.34109323069	0.659925312	2.11411809007635	21.2269751406136
5365	Saint Kitts and Nevis	2004	23.02985324303	0.66756813	2.11449777324981	20.9153554697802
5366	Saint Kitts and Nevis	2005	22.7168944801	0.679145457	2.11623580139623	20.6006586787038
5367	Saint Kitts and Nevis	2006	22.2619726292	0.690078667	2.11414505993618	20.1478275692638
5368	Saint Kitts and Nevis	2007	21.7630444704	0.697727628	2.11585663271244	19.6471878376876
5369	Saint Kitts and Nevis	2008	21.18285181927	0.704141494	2.11295797060154	19.0698938486685
5370	Saint Kitts and Nevis	2009	20.71397871149	0.709499498	2.11223998750041	18.6017387239896
5371	Saint Kitts and Nevis	2010	20.31421859615	0.714460415	2.11485288375773	18.1993657123923
5372	Saint Kitts and Nevis	2011	20.11000326524	0.719324381	2.11392386440354	17.9960794008365
5373	Saint Kitts and Nevis	2012	19.99676783837	0.723436152	2.11487264941	17.88189518896
5374	Saint Kitts and Nevis	2013	19.88605112989	0.726706593	2.11246914940042	17.7735819804896
5375	Saint Kitts and Nevis	2014	19.83031107512	0.729530219	2.11601180808595	17.7142992670341

TableS2 Frontier analysis of Smoking-Related Low Back Pain among WCBA

	location	year	val	SDI	frontier	eff_diff
5376	Saint Kitts and Nevis	2015	19.77237255536	0.732220672	2.11575105794254	17.6566214974175
5377	Saint Kitts and Nevis	2016	19.7777296107	0.735566138	2.11231890658833	17.6654107041117
5378	Saint Kitts and Nevis	2017	19.71387934684	0.739470681	2.11107809332127	17.6028012535187
5379	Saint Kitts and Nevis	2018	19.73997227312	0.743736461	2.11412373726011	17.6258485358599
5380	Saint Kitts and Nevis	2019	19.8046917809	0.748152066	2.11546767823096	17.689224102669
5381	Saint Kitts and Nevis	2020	19.80809043404	0.751568409	2.11364784814951	17.6944425858905
5382	Saint Kitts and Nevis	2021	19.90002299523	0.754987055	2.11397474816692	17.7860482470631
5383	Saint Lucia	1990	35.15282858197	0.49629657	2.11112167502466	33.0417069069453
5384	Saint Lucia	1991	35.7507688447	0.505975555	2.11111056187295	33.639658282827
5385	Saint Lucia	1992	36.34019138191	0.515368261	2.11413065862928	34.2260607232807
5386	Saint Lucia	1993	36.9433677283	0.524234949	2.11153670345475	34.8318310248452
5387	Saint Lucia	1994	37.5337907798	0.532731083	2.11392320518822	35.4198675746118
5388	Saint Lucia	1995	38.18154022259	0.541479794	2.11454582073881	36.0669944018512
5389	Saint Lucia	1996	38.87033995091	0.550242412	2.11341798118224	36.7569219697278
5390	Saint Lucia	1997	39.54449609205	0.558293461	2.11200267768315	37.4324934143669
5391	Saint Lucia	1998	40.22305643195	0.566138703	2.11032465607746	38.1127317758725
5392	Saint Lucia	1999	40.95283821075	0.573428555	2.11120430467691	38.8416339060731
5393	Saint Lucia	2000	41.68096002505	0.579788896	2.11400471745224	39.5669553075978
5394	Saint Lucia	2001	42.1926992743	0.585087558	2.11216176707528	40.0805375072247
5395	Saint Lucia	2002	42.34392951535	0.590167578	2.11220173426823	40.2317277810818
5396	Saint Lucia	2003	42.38531385605	0.595675146	2.11550121277188	40.2698126432781
5397	Saint Lucia	2004	42.19492107957	0.601625482	2.11182702303435	40.0830940565357
5398	Saint Lucia	2005	41.95556680525	0.607239866	2.11318229998703	39.842384505263
5399	Saint Lucia	2006	41.50712129169	0.6127553	2.114557084102	39.392564207588
5400	Saint Lucia	2007	40.69982475454	0.617994714	2.11234023478283	38.5874845197572

TableS2 Frontier analysis of Smoking-Related Low Back Pain among WCBA

	location	year	val	SDI	frontier	eff_diff
5401	Saint Lucia	2008	39.73168007128	0.623273001	2.1115357339509	37.6201443373291
5402	Saint Lucia	2009	38.81685435348	0.628169849	2.11457270521455	36.7022816482655
5403	Saint Lucia	2010	38.21848979436	0.632782905	2.11551742922273	36.1029723651373
5404	Saint Lucia	2011	37.76967725422	0.637439442	2.11782800767177	35.6518492465482
5405	Saint Lucia	2012	37.35954884709	0.641567127	2.1121404105397	35.2474084365503
5406	Saint Lucia	2013	37.05302250741	0.645153079	2.11484972689212	34.9381727805179
5407	Saint Lucia	2014	36.79977405964	0.648743507	2.11182592453054	34.6879481351095
5408	Saint Lucia	2015	36.58581242458	0.65215748	2.11495211116558	34.4708603134144
5409	Saint Lucia	2016	36.59517592487	0.65565334	2.11142271079452	34.4837532140755
5410	Saint Lucia	2017	36.48891480889	0.659341346	2.11725013603388	34.3716646728561
5411	Saint Lucia	2018	36.46693888288	0.663086682	2.11295461696515	34.3539842659148
5412	Saint Lucia	2019	36.47056832567	0.666840359	2.11122146667045	34.3593468589996
5413	Saint Lucia	2020	36.53685210053	0.66972049	2.113643957838	34.423208142692
5414	Saint Lucia	2021	36.69439789473	0.672509735	2.1134820910582	34.5809158036718
5415	Saint Vincent and the Grenadines	1990	20.73181812304	0.475930186	2.11456011085433	18.6172580121857
5416	Saint Vincent and the Grenadines	1991	21.23425119674	0.481075658	2.11115225750754	19.1230989392325
5417	Saint Vincent and the Grenadines	1992	21.77957990148	0.485977871	2.11684289424921	19.6627370072308
5418	Saint Vincent and the Grenadines	1993	22.24919838338	0.490998791	2.11337102999988	20.1358273533801
5419	Saint Vincent and the Grenadines	1994	22.78129420377	0.496379132	2.11185084087897	20.669443362891
5420	Saint Vincent and the Grenadines	1995	23.22035887344	0.50298609	2.11465673649074	21.1057021369493
5421	Saint Vincent and the Grenadines	1996	23.75486935851	0.509527158	2.11090456602648	21.6439647924835
5422	Saint Vincent and the Grenadines	1997	24.1530668773	0.515482619	2.11510205230342	22.0379648249966
5423	Saint Vincent and the Grenadines	1998	24.60673500848	0.521209679	2.11139538866831	22.4953396198117
5424	Saint Vincent and the Grenadines	1999	25.06322335497	0.527099552	2.11805978485552	22.9451635701145
5425	Saint Vincent and the Grenadines	2000	25.53009157542	0.53317329	2.11315593587037	23.4169356395496

TableS2 Frontier analysis of Smoking-Related Low Back Pain among WCBA

	location	year	val	SDI	frontier	eff_diff
5426	Saint Vincent and the Grenadines	2001	25.91667763017	0.539455712	2.1126860740075	23.8039915561625
5427	Saint Vincent and the Grenadines	2002	26.28540198679	0.546063616	2.11379831065987	24.1716036761301
5428	Saint Vincent and the Grenadines	2003	26.56454469681	0.552693724	2.1121700755225	24.4523746212875
5429	Saint Vincent and the Grenadines	2004	26.76894417796	0.558663543	2.1141494798318	24.6547946981282
5430	Saint Vincent and the Grenadines	2005	26.98055449065	0.563588116	2.1175986958424	24.8629557948076
5431	Saint Vincent and the Grenadines	2006	27.11801113818	0.568221748	2.11405349401477	25.0039576441652
5432	Saint Vincent and the Grenadines	2007	27.19866434985	0.572819052	2.11609693287922	25.0825674169708
5433	Saint Vincent and the Grenadines	2008	27.27238775394	0.577611145	2.11011136715797	25.162276386782
5434	Saint Vincent and the Grenadines	2009	27.35615463781	0.582549107	2.11590747775191	25.2402471600581
5435	Saint Vincent and the Grenadines	2010	27.44197856452	0.587190384	2.11594799792727	25.3260305665927
5436	Saint Vincent and the Grenadines	2011	27.56208350827	0.590259096	2.113562052974	25.448521455296
5437	Saint Vincent and the Grenadines	2012	27.68668865759	0.592153908	2.11802999538678	25.5686586622032
5438	Saint Vincent and the Grenadines	2013	27.74304220843	0.594614075	2.11692458513	25.6261176233
5439	Saint Vincent and the Grenadines	2014	27.80756820504	0.598378672	2.11321253269855	25.6943556723414
5440	Saint Vincent and the Grenadines	2015	27.87460897011	0.603754135	2.1165063246389	25.7581026454711
5441	Saint Vincent and the Grenadines	2016	28.03737934466	0.61005921	2.11488466519646	25.9224946794635
5442	Saint Vincent and the Grenadines	2017	28.17749607591	0.616438473	2.1137187758529	26.0637773000571
5443	Saint Vincent and the Grenadines	2018	28.38751459257	0.622634944	2.11450867049829	26.2730059220717
5444	Saint Vincent and the Grenadines	2019	28.56870604189	0.628325986	2.11036813629176	26.4583379055982
5445	Saint Vincent and the Grenadines	2020	28.62774123984	0.632860904	2.11056974937109	26.5171714904689
5446	Saint Vincent and the Grenadines	2021	28.89001171275	0.637195963	2.11162993577759	26.7783817769724
5447	Samoa	1990	66.56300333381	0.487491428	2.1146037524	64.44839958141
5448	Samoa	1991	67.53920505233	0.490987146	2.11605858453823	65.4231464677918
5449	Samoa	1992	68.49569001923	0.494027448	2.11217707560965	66.3835129436203
5450	Samoa	1993	69.70280899084	0.497079264	2.1147199151902	67.5880890756498

TableS2 Frontier analysis of Smoking-Related Low Back Pain among WCBA

	location	year	val	SDI	frontier	eff_diff
5451	Samoa	1994	70.91926106055	0.499484836	2.11370420301391	68.8055568575361
5452	Samoa	1995	72.32763125043	0.502369735	2.11260325852732	70.2150279919027
5453	Samoa	1996	74.1745601977	0.505778026	2.11202440569697	72.062535792003
5454	Samoa	1997	76.19008463401	0.509096382	2.11605957006386	74.0740250639462
5455	Samoa	1998	78.32443542881	0.512367566	2.11757774408377	76.2068576847262
5456	Samoa	1999	80.4858853382	0.51540905	2.11640430647286	78.3694810317271
5457	Samoa	2000	82.36664292647	0.518902327	2.11378322717486	80.2528596992951
5458	Samoa	2001	83.73229098656	0.522971626	2.1117263516042	81.6205646349558
5459	Samoa	2002	84.89191849821	0.52724267	2.11241347897781	82.7795050192322
5460	Samoa	2003	85.67670362069	0.531603736	2.11516478197712	83.5615388387129
5461	Samoa	2004	86.26344247611	0.536003276	2.11320810511512	84.1502343709949
5462	Samoa	2005	86.45519503454	0.540557908	2.11598621894226	84.3392088155977
5463	Samoa	2006	86.10878625099	0.544812068	2.1143173033481	83.9944689476419
5464	Samoa	2007	85.29405792122	0.549110752	2.11513349369148	83.1789244275285
5465	Samoa	2008	83.99434881379	0.553164596	2.11776573247352	81.8765830813165
5466	Samoa	2009	82.5063033117	0.556474015	2.11036896477081	80.3959343469292
5467	Samoa	2010	81.28966957205	0.559622905	2.11432136487741	79.1753482071726
5468	Samoa	2011	80.24800317314	0.562891574	2.11232749595123	78.1356756771888
5469	Samoa	2012	79.05356665904	0.565320388	2.11800625281176	76.9355604062282
5470	Samoa	2013	77.91954078814	0.567448536	2.11106420324094	75.8084765848991
5471	Samoa	2014	77.06111852734	0.569493661	2.11231915810716	74.9487993692328
5472	Samoa	2015	76.3107160769	0.571998983	2.11367468327743	74.1970413936226
5473	Samoa	2016	75.82009608274	0.575402051	2.11296095114234	73.7071351315977
5474	Samoa	2017	75.56384531874	0.578925435	2.11330558168224	73.4505397370578
5475	Samoa	2018	75.42016113572	0.582320661	2.11236336002845	73.3077977756916

TableS2 Frontier analysis of Smoking-Related Low Back Pain among WCBA

	location	year	val	SDI	frontier	eff_diff
5476	Samoa	2019	75.34202283603	0.586256695	2.1134916220203	73.2285312140097
5477	Samoa	2020	74.74816491072	0.590075296	2.11131108601911	72.6368538247009
5478	Samoa	2021	74.92530368221	0.593392769	2.11159587238311	72.8137078098269
5479	San Marino	1990	225.63065256591	0.813244888	2.11380904561717	223.516843520293
5480	San Marino	1991	224.9544668104	0.818282513	2.11148578197397	222.842981028426
5481	San Marino	1992	225.31343399051	0.822782758	2.11662506683382	223.196808923676
5482	San Marino	1993	226.03200854391	0.827382324	2.11338218386386	223.918626360046
5483	San Marino	1994	227.37542833765	0.831970983	2.11163441385478	225.263793923795
5484	San Marino	1995	229.30797249595	0.836762726	2.11005739261183	227.197915103338
5485	San Marino	1996	231.55997142023	0.841389495	2.11416403108086	229.445807389149
5486	San Marino	1997	233.64437762002	0.845864865	2.1122456373087	231.532131982711
5487	San Marino	1998	235.36051193344	0.850474771	2.11164561033652	233.248866323103
5488	San Marino	1999	237.21183837708	0.855338613	2.11273273083605	235.099105646244
5489	San Marino	2000	239.18753637455	0.859695363	2.11166967828301	237.075866696267
5490	San Marino	2001	241.82765548191	0.863747757	2.11311068766481	239.714544794245
5491	San Marino	2002	243.76573129623	0.865680592	2.11362000633037	241.6521112899
5492	San Marino	2003	245.3844975321	0.867522009	2.11126983390588	243.273227698194
5493	San Marino	2004	246.82243338361	0.869287134	2.1109759886675	244.711457394942
5494	San Marino	2005	248.3048609656	0.870979596	2.11481352906717	246.190047436533
5495	San Marino	2006	248.55296011745	0.87265215	2.1099929594178	246.442967158032
5496	San Marino	2007	248.45183786973	0.874307253	2.1132627468574	246.338575122873
5497	San Marino	2008	247.76223081488	0.875991881	2.11623612987129	245.645994685009
5498	San Marino	2009	246.88952024924	0.877705228	2.12012509899131	244.769395150249
5499	San Marino	2010	245.71474560081	0.879440814	2.11363359508627	243.601112005724
5500	San Marino	2011	243.84091658302	0.881197518	2.11141685153702	241.729499731483

TableS2 Frontier analysis of Smoking-Related Low Back Pain among WCBA

	location	year	val	SDI	frontier	eff_diff
5501	San Marino	2012	241.74232804764	0.882924531	2.11242469647912	239.629903351161
5502	San Marino	2013	238.67267293784	0.884592627	2.11343357981315	236.559239358027
5503	San Marino	2014	236.05211463284	0.885353694	2.11137286073909	233.940741772101
5504	San Marino	2015	233.64934599005	0.884693365	2.11123398995621	231.538112000094
5505	San Marino	2016	231.90212069493	0.884477665	2.11094231378414	229.791178381146
5506	San Marino	2017	230.27446345274	0.884557607	2.11014031213754	228.164323140602
5507	San Marino	2018	228.60710952612	0.885133516	2.11478595760597	226.492323568514
5508	San Marino	2019	226.54492549704	0.88620066	2.11254108270333	224.432384414337
5509	San Marino	2020	221.13933072804	0.886867937	2.11447359104661	219.024857136993
5510	San Marino	2021	221.60496878258	0.888005474	2.11221006840963	219.49275871417
5511	Saudi Arabia	1990	13.79864236946	0.538954515	2.1159484508705	11.6826939185895
5512	Saudi Arabia	1991	13.97752949777	0.549907717	2.11225635870726	11.8652731390627
5513	Saudi Arabia	1992	14.14648876376	0.561144266	2.11539725199066	12.0310915117693
5514	Saudi Arabia	1993	14.3139490257	0.572186913	2.11190902236468	12.2020400033353
5515	Saudi Arabia	1994	14.45246254368	0.582878871	2.11522308941207	12.3372394542679
5516	Saudi Arabia	1995	14.61407834969	0.593251423	2.11390230239815	12.5001760472919
5517	Saudi Arabia	1996	14.71811312191	0.603648418	2.11296099744104	12.605152124469
5518	Saudi Arabia	1997	14.80747745186	0.613749116	2.11351929669205	12.6939581551679
5519	Saudi Arabia	1998	14.89055022724	0.623518214	2.11347856007714	12.7770716671629
5520	Saudi Arabia	1999	14.94638973811	0.632796447	2.11211253293239	12.8342772051776
5521	Saudi Arabia	2000	15.02072405963	0.642311774	2.1132648033461	12.9074592562839
5522	Saudi Arabia	2001	15.11116778647	0.651502092	2.1126999402255	12.9984678462445
5523	Saudi Arabia	2002	15.16370649645	0.660356856	2.11198308821277	13.0517234082372
5524	Saudi Arabia	2003	15.18995271555	0.669839577	2.11232220691931	13.0776305086307
5525	Saudi Arabia	2004	15.22809332216	0.679917856	2.1124507689372	13.1156425532228

TableS2 Frontier analysis of Smoking-Related Low Back Pain among WCBA

	location	year	val	SDI	frontier	eff_diff
5526	Saudi Arabia	2005	15.28470054123	0.690520852	2.11099858726207	13.1737019539679
5527	Saudi Arabia	2006	15.33589058961	0.701152613	2.11369769697754	13.2221928926325
5528	Saudi Arabia	2007	15.44800773809	0.711442141	2.11237065689843	13.3356370811916
5529	Saudi Arabia	2008	15.5844178993	0.721960205	2.11337332310545	13.4710445761945
5530	Saudi Arabia	2009	15.74640455163	0.731158488	2.11088132174462	13.6355232298854
5531	Saudi Arabia	2010	15.92862242553	0.740300669	2.11129488906333	13.8173275364667
5532	Saudi Arabia	2011	16.07549914457	0.749959451	2.11465406738469	13.9608450771853
5533	Saudi Arabia	2012	16.23561830882	0.759054935	2.11275569591	14.12286261291
5534	Saudi Arabia	2013	16.43307375015	0.767387864	2.11385670687099	14.319217043279
5535	Saudi Arabia	2014	16.68472613419	0.775178133	2.1123984293273	14.5723277048627
5536	Saudi Arabia	2015	17.0606133529	0.782300802	2.11286588468186	14.9477474682181
5537	Saudi Arabia	2016	17.52487047671	0.788867882	2.11240441213467	15.4124660645753
5538	Saudi Arabia	2017	18.06225595606	0.794886288	2.11092694148455	15.9513290145755
5539	Saudi Arabia	2018	18.60201047214	0.800656985	2.11228697010263	16.4897235020374
5540	Saudi Arabia	2019	19.16016453993	0.806087841	2.11231055764986	17.0478539822801
5541	Saudi Arabia	2020	19.66139621031	0.810541861	2.11352758606159	17.5478686242484
5542	Saudi Arabia	2021	20.14481593383	0.815143493	2.11423174262476	18.0305841912052
5543	Senegal	1990	5.88849529027	0.238047613	2.59863463207034	3.28986065819966
5544	Senegal	1991	5.916366417	0.244482178	2.5556581280595	3.3607082889405
5545	Senegal	1992	5.94834552169	0.250416532	2.59393493944538	3.35441058224462
5546	Senegal	1993	6.0036698253	0.255767964	2.52977352865187	3.47389629664813
5547	Senegal	1994	6.01762440403	0.260379997	2.51259393511915	3.50503046891085
5548	Senegal	1995	6.03056994435	0.264438773	2.4701140022559	3.5604559420941
5549	Senegal	1996	6.02601411449	0.268318965	2.48016740668797	3.54584670780203
5550	Senegal	1997	6.06121820588	0.272072875	2.47347783884299	3.58774036703701

TableS2 Frontier analysis of Smoking-Related Low Back Pain among WCBA

	location	year	val	SDI	frontier	eff_diff
5551	Senegal	1998	6.11580556088	0.275995656	2.45198834243616	3.66381721844383
5552	Senegal	1999	6.12629435286	0.280111918	2.45401995958592	3.67227439327408
5553	Senegal	2000	6.08980140808	0.283784903	2.43719044795494	3.65261096012506
5554	Senegal	2001	6.07100924989	0.287451899	2.4307205453691	3.64028870452089
5555	Senegal	2002	6.0208052524	0.291046288	2.42395939350344	3.59684585889656
5556	Senegal	2003	5.94684797783	0.294887172	2.39300223640217	3.55384574142783
5557	Senegal	2004	5.8498502517	0.298984521	2.40622855550919	3.44362169619081
5558	Senegal	2005	5.7500090855	0.303257209	2.39300005065273	3.35700903484727
5559	Senegal	2006	5.62055952708	0.30763391	2.38898274782753	3.23157677925247
5560	Senegal	2007	5.44548780121	0.312570298	2.37105068015333	3.07443712105667
5561	Senegal	2008	5.25297797258	0.317894089	2.34703869718988	2.90593927539012
5562	Senegal	2009	5.04342542827	0.323657726	2.32220905865138	2.72121636961862
5563	Senegal	2010	4.88391808241	0.329804072	2.30560189047054	2.57831619193946
5564	Senegal	2011	4.72728196232	0.336059897	2.28886112845897	2.43842083386103
5565	Senegal	2012	4.58725023963	0.342977114	2.21617846312014	2.37107177650986
5566	Senegal	2013	4.45134991612	0.350134004	2.117427938628	2.333921977492
5567	Senegal	2014	4.32153899194	0.357181573	2.11741856641026	2.20412042552974
5568	Senegal	2015	4.23825117647	0.364144496	2.11446018149817	2.12379099497183
5569	Senegal	2016	4.1835475383	0.371114525	2.11416606195481	2.06938147634519
5570	Senegal	2017	4.18838299532	0.378351477	2.11464603036946	2.07373696495054
5571	Senegal	2018	4.18052486311	0.386079666	2.11266930378827	2.06785555932173
5572	Senegal	2019	4.16304630454	0.394012316	2.11329164640375	2.04975465813625
5573	Senegal	2020	4.08753772792	0.401197516	2.11253409841643	1.97500362950357
5574	Senegal	2021	4.02953916315	0.408054193	2.11560872954355	1.91393043360645
5575	Serbia	1990	391.84853993473	0.63051102	2.11381748815043	389.73472244658

TableS2 Frontier analysis of Smoking-Related Low Back Pain among WCBA

	location	year	val	SDI	frontier	eff_diff
5576	Serbia	1991	394.71197688196	0.638016934	2.1109847360215	392.600992145938
5577	Serbia	1992	396.64753958694	0.641971994	2.11220295234977	394.53533663459
5578	Serbia	1993	396.83439531783	0.642096034	2.1161513852289	394.718243932601
5579	Serbia	1994	398.77179740945	0.642223221	2.11372399902174	396.658073410428
5580	Serbia	1995	402.47081934303	0.642739671	2.11477284327883	400.356046499751
5581	Serbia	1996	406.88678755025	0.644846417	2.11352805075031	404.7732594995
5582	Serbia	1997	413.8190377743	0.647162094	2.11214925319205	411.706888521108
5583	Serbia	1998	420.77957433284	0.650245989	2.11677929071492	418.662795042125
5584	Serbia	1999	428.96386621293	0.655534174	2.11238457660027	426.85148163633
5585	Serbia	2000	435.59522304328	0.6607528	2.11387656618059	433.481346477099
5586	Serbia	2001	438.01110872047	0.665407335	2.11454292397092	435.896565796499
5587	Serbia	2002	440.02530883744	0.671511072	2.11336365548023	437.91194518196
5588	Serbia	2003	441.8821274758	0.678334929	2.11365798737716	439.768469488423
5589	Serbia	2004	440.82796660501	0.686819842	2.11183342980204	438.716133175208
5590	Serbia	2005	438.39381813458	0.696097568	2.1126492153442	436.281168919236
5591	Serbia	2006	437.99300370823	0.704945018	2.11353515039453	435.879468557835
5592	Serbia	2007	437.02592347591	0.713242305	2.11189759416926	434.914025881741
5593	Serbia	2008	435.09310019931	0.721505306	2.11561940740114	432.977480791909
5594	Serbia	2009	434.43425346104	0.729965596	2.11379766688112	432.320455794159
5595	Serbia	2010	435.53998003939	0.740091311	2.11447954457741	433.425500494813
5596	Serbia	2011	437.27431973756	0.750072762	2.10998342780672	435.164336309753
5597	Serbia	2012	439.0343574842	0.757105082	2.1157878656554	436.918569618545
5598	Serbia	2013	441.46786881904	0.762170908	2.11183496216636	439.356033856874
5599	Serbia	2014	443.89327755807	0.766043091	2.11355143227624	441.779726125794
5600	Serbia	2015	446.61216866745	0.769842223	2.1138499161684	444.498318751282

TableS2 Frontier analysis of Smoking-Related Low Back Pain among WCBA

	location	year	val	SDI	frontier	eff_diff
5601	Serbia	2016	449.27700024917	0.773654077	2.11408354600405	447.162916703166
5602	Serbia	2017	451.56494603525	0.777460384	2.11469817971805	449.450247855532
5603	Serbia	2018	453.44846437629	0.781494974	2.1122436899138	451.336220686376
5604	Serbia	2019	454.86369629052	0.7857683	2.11419391119179	452.749502379328
5605	Serbia	2020	454.42308406756	0.789183333	2.1122583559844	452.310825711576
5606	Serbia	2021	450.25729799359	0.792416294	2.11144574129432	448.145852252296
5607	Seychelles	1990	23.37843489181	0.575526496	2.1138342964267	21.2646005953833
5608	Seychelles	1991	23.86922325858	0.582698331	2.11138991287671	21.7578333457033
5609	Seychelles	1992	24.49366459087	0.590052919	2.11302366649754	22.3806409243725
5610	Seychelles	1993	25.07093216129	0.598044194	2.11393779982726	22.9569943614627
5611	Seychelles	1994	25.6467895177	0.606041268	2.11399637618746	23.5327931415125
5612	Seychelles	1995	26.29796215975	0.613853281	2.11127668277215	24.1866854769778
5613	Seychelles	1996	26.96557003653	0.62213517	2.11073653025733	24.8548335062727
5614	Seychelles	1997	27.46400982775	0.630697732	2.11276301167474	25.3512468160753
5615	Seychelles	1998	27.97788786678	0.638438882	2.11138059260372	25.8665072741763
5616	Seychelles	1999	28.43936000778	0.645339666	2.11415991365582	26.3252000941242
5617	Seychelles	2000	28.92169868415	0.651551988	2.11174362381425	26.8099550603357
5618	Seychelles	2001	29.40984900024	0.656490668	2.10953088814141	27.3003181120986
5619	Seychelles	2002	29.84731852155	0.66090779	2.11500978537629	27.7323087361737
5620	Seychelles	2003	30.24735475611	0.664162027	2.11389379182238	28.1334609642876
5621	Seychelles	2004	30.64895697906	0.666461353	2.11239997874672	28.5365570003133
5622	Seychelles	2005	30.97624206929	0.669264742	2.11391690433803	28.862325164952
5623	Seychelles	2006	31.2535482559	0.672149914	2.1108127340313	29.1427355218687
5624	Seychelles	2007	31.39840160162	0.675077438	2.11551184982196	29.282889751798
5625	Seychelles	2008	31.56666334145	0.676590946	2.11312611981536	29.4535372216346

TableS2 Frontier analysis of Smoking-Related Low Back Pain among WCBA

	location	year	val	SDI	frontier	eff_diff
5626	Seychelles	2009	31.68029949794	0.677847028	2.11302561477671	29.5672738831633
5627	Seychelles	2010	31.75594347796	0.68016364	2.11637653657493	29.6395669413851
5628	Seychelles	2011	31.87129077221	0.683681491	2.11125341248855	29.7600373597214
5629	Seychelles	2012	31.9040352856	0.687629394	2.1175504996962	29.7864847859038
5630	Seychelles	2013	31.80456300376	0.692525734	2.11174908552831	29.6928139182317
5631	Seychelles	2014	31.75710221126	0.697114589	2.1113117908435	29.6457904204165
5632	Seychelles	2015	31.68370380562	0.70172282	2.11434841210914	29.5693553935109
5633	Seychelles	2016	31.61799893582	0.706183623	2.11272327149261	29.5052756643274
5634	Seychelles	2017	31.55239469291	0.711027281	2.11500435805864	29.4373903348514
5635	Seychelles	2018	31.4883976939	0.716407819	2.11700072859716	29.3713969653028
5636	Seychelles	2019	31.40534946873	0.722073685	2.11464170428046	29.2907077644495
5637	Seychelles	2020	31.23668452541	0.726663769	2.11368983322333	29.1229946921867
5638	Seychelles	2021	31.06734677271	0.730150775	2.11366155671614	28.9536852159939
5639	Sierra Leone	1990	30.99902878664	0.211569335	3.50021588881797	27.498812897822
5640	Sierra Leone	1991	31.01379067364	0.213013567	3.45760318422632	27.5561874894137
5641	Sierra Leone	1992	31.67888727193	0.21422304	3.49800093575	28.18088633618
5642	Sierra Leone	1993	32.34646831663	0.216089201	3.02941028180746	29.3170580348225
5643	Sierra Leone	1994	32.60941434558	0.218277411	2.88814922145571	29.7212651241243
5644	Sierra Leone	1995	32.69285753013	0.219822604	2.88693809961746	29.8059194305125
5645	Sierra Leone	1996	32.86291644056	0.21996927	2.91889574340412	29.9440206971559
5646	Sierra Leone	1997	32.78328949619	0.219089791	2.8353341169451	29.9479553792449
5647	Sierra Leone	1998	32.633698375	0.218584313	2.86182455470661	29.7718738202934
5648	Sierra Leone	1999	32.70522277469	0.217943042	2.932149180618	29.773073594072
5649	Sierra Leone	2000	32.62519243842	0.218492837	2.93280468251661	29.6923877559034
5650	Sierra Leone	2001	31.8602576032	0.220391233	2.9306293114075	28.9296282917925

TableS2 Frontier analysis of Smoking-Related Low Back Pain among WCBA

	location	year	val	SDI	frontier	eff_diff
5651	Sierra Leone	2002	30.6846772366	0.224585825	2.70410288789683	27.9805743487032
5652	Sierra Leone	2003	29.66404041681	0.2292359	2.74434966800983	26.9196907488002
5653	Sierra Leone	2004	28.86594389365	0.234131674	2.63137564777516	26.2345682458748
5654	Sierra Leone	2005	28.169821659	0.23954994	2.58130863489929	25.5885130241007
5655	Sierra Leone	2006	27.45723189304	0.245647886	2.5426859722771	24.9145459207629
5656	Sierra Leone	2007	26.58060898705	0.252523077	2.52082320237247	24.0597857846775
5657	Sierra Leone	2008	25.60235529037	0.259781562	2.51637657080883	23.0859787195612
5658	Sierra Leone	2009	24.5829545895	0.26705838	2.48018905948927	22.1027655300107
5659	Sierra Leone	2010	23.55658289923	0.274270275	2.45860254999615	21.0979803492338
5660	Sierra Leone	2011	22.52955760465	0.281673575	2.4549031567247	20.0746544479253
5661	Sierra Leone	2012	21.29446631643	0.290338512	2.43507952453435	18.8593867918956
5662	Sierra Leone	2013	20.1203848933	0.300736693	2.39574721402919	17.7246376792708
5663	Sierra Leone	2014	19.04952661987	0.310348867	2.36300969096365	16.6865169289063
5664	Sierra Leone	2015	18.22514067834	0.31699865	2.35644230804941	15.8686983702906
5665	Sierra Leone	2016	17.58729294677	0.324091565	2.32256968141562	15.2647232653544
5666	Sierra Leone	2017	17.07668699597	0.331213431	2.30589755576506	14.7707894402049
5667	Sierra Leone	2018	16.66919426626	0.338341564	2.26856259223662	14.4006316740234
5668	Sierra Leone	2019	16.41168328205	0.345770019	2.13803663047282	14.2736466515772
5669	Sierra Leone	2020	16.34095913033	0.352412022	2.11780123416816	14.2231578961618
5670	Sierra Leone	2021	16.26170618392	0.358665881	2.11341983438709	14.1482863495329
5671	Singapore	1990	82.59112836006	0.686404444	2.11355183235746	80.4775765277025
5672	Singapore	1991	80.02332932048	0.695055558	2.11114855230422	77.9121807681758
5673	Singapore	1992	77.72879670974	0.703427834	2.11066106790306	75.6181356418369
5674	Singapore	1993	75.90465742206	0.712315204	2.10905748186179	73.7955999401982
5675	Singapore	1994	74.47845655127	0.721256797	2.11300700409968	72.3654495471703

TableS2 Frontier analysis of Smoking-Related Low Back Pain among WCBA

	location	year	val	SDI	frontier	eff_diff
5676	Singapore	1995	74.01633517896	0.729982231	2.11270876086741	71.9036264180926
5677	Singapore	1996	74.1304857558	0.739028235	2.11217095034657	72.0183148054534
5678	Singapore	1997	73.49312676692	0.747571898	2.11287876789945	71.3802479990206
5679	Singapore	1998	72.80471015955	0.754873204	2.11400634046648	70.6907038190835
5680	Singapore	1999	72.05754622185	0.760831136	2.11355263924135	69.9439935826087
5681	Singapore	2000	71.50586580282	0.76771737	2.11587634469421	69.3899894581258
5682	Singapore	2001	72.0849342108	0.774294657	2.11207924559819	69.9728549652018
5683	Singapore	2002	73.10088934218	0.77973577	2.11386071465617	70.9870286275238
5684	Singapore	2003	74.19339461539	0.784165555	2.11429711627852	72.0790974991115
5685	Singapore	2004	74.4271350708	0.789852774	2.11444020301628	72.3126948677837
5686	Singapore	2005	73.57524648781	0.796455751	2.11389172064597	71.461354767164
5687	Singapore	2006	72.57452505429	0.803342694	2.11356197025149	70.4609630840385
5688	Singapore	2007	71.26609036015	0.810625304	2.11473447258392	69.1513558875661
5689	Singapore	2008	69.97537989058	0.818124162	2.11498614869211	67.8603937418879
5690	Singapore	2009	69.15521184499	0.824251516	2.11070165816857	67.0445101868214
5691	Singapore	2010	68.46541226007	0.83022216	2.11251136815736	66.3529008919126
5692	Singapore	2011	68.29138017169	0.833829608	2.11781340469149	66.1735667669985
5693	Singapore	2012	68.03006527005	0.837391807	2.11370811596086	65.9163571540891
5694	Singapore	2013	67.72392286423	0.840420178	2.11356025349443	65.6103626107356
5695	Singapore	2014	67.44843213729	0.842894538	2.11147513247567	65.3369570048143
5696	Singapore	2015	67.23721013795	0.845401857	2.11332249021912	65.1238876477309
5697	Singapore	2016	67.0190083639	0.847596999	2.11265372164433	64.9063546422557
5698	Singapore	2017	67.40824965344	0.849051518	2.11387857724679	65.2943710761932
5699	Singapore	2018	68.69648630906	0.850762729	2.11513568674614	66.5813506223139
5700	Singapore	2019	70.34339499957	0.852497338	2.11463297777273	68.2287620217973

TableS2 Frontier analysis of Smoking-Related Low Back Pain among WCBA

	location	year	val	SDI	frontier	eff_diff
5701	Singapore	2020	68.9115669426	0.85429601	2.11250880748905	66.799058135111
5702	Singapore	2021	72.3999596377	0.856097766	2.11526116476227	70.2846984729377
5703	Slovakia	1990	349.98888618266	0.653853505	2.11543938778809	347.873446794872
5704	Slovakia	1991	349.56712060867	0.658731402	2.11358948275127	347.453531125919
5705	Slovakia	1992	348.64627026724	0.665830111	2.11606341853	346.53020684871
5706	Slovakia	1993	347.59778083634	0.675967937	2.11073531602311	345.487045520317
5707	Slovakia	1994	346.54794723518	0.688136477	2.11230873675534	344.435638498425
5708	Slovakia	1995	345.5690032328	0.697476536	2.11084542803689	343.458157804763
5709	Slovakia	1996	344.08852707357	0.704405003	2.11240313893567	341.976123934634
5710	Slovakia	1997	340.66157039301	0.71151321	2.112845196762	338.548725196248
5711	Slovakia	1998	335.87876206988	0.719058766	2.11275111515893	333.766010954721
5712	Slovakia	1999	330.77854497625	0.726144173	2.11291515230781	328.665629823942
5713	Slovakia	2000	326.47763536203	0.73404624	2.11325395457833	324.364381407452
5714	Slovakia	2001	321.93688871172	0.741392554	2.11321634709234	319.823672364628
5715	Slovakia	2002	317.07906958374	0.746791142	2.11184665148157	314.967222932258
5716	Slovakia	2003	312.95925084062	0.75116727	2.11036694334583	310.848883897274
5717	Slovakia	2004	309.54463326225	0.755836694	2.113537924678	307.431095337572
5718	Slovakia	2005	306.34043079192	0.761724185	2.11212463662932	304.228306155291
5719	Slovakia	2006	302.69275730288	0.767673036	2.11473518142627	300.578022121454
5720	Slovakia	2007	299.18002139988	0.772914153	2.11203516205514	297.067986237825
5721	Slovakia	2008	296.4330985795	0.7771974	2.11456360492687	294.318534974573
5722	Slovakia	2009	295.04128490962	0.78105226	2.11272518258417	292.928559727036
5723	Slovakia	2010	294.87844907272	0.786254422	2.11437768292519	292.764071389795
5724	Slovakia	2011	295.12229419104	0.791465382	2.11319938542762	293.009094805612
5725	Slovakia	2012	295.56909352774	0.795456216	2.11145961235289	293.457633915387

TableS2 Frontier analysis of Smoking-Related Low Back Pain among WCBA

	location	year	val	SDI	frontier	eff_diff
5726	Slovakia	2013	296.77454176174	0.797903723	2.11362207024627	294.660919691494
5727	Slovakia	2014	298.57685266781	0.799618796	2.11186080780844	296.464991860002
5728	Slovakia	2015	301.68377145795	0.80039418	2.11637752326012	299.56739393469
5729	Slovakia	2016	305.58552294563	0.80087821	2.11503379906357	303.470489146566
5730	Slovakia	2017	309.64567408771	0.802277317	2.11267788977189	307.532996197938
5731	Slovakia	2018	313.78976018028	0.804357302	2.11278501184324	311.676975168437
5732	Slovakia	2019	318.46936348776	0.806448457	2.1113033636106	316.358060124149
5733	Slovakia	2020	323.3089754275	0.808305966	2.11084299753177	321.198132429968
5734	Slovakia	2021	325.5030739532	0.81061053	2.11217922299554	323.390894730204
5735	Slovenia	1990	297.17926843954	0.727463928	2.11474330363585	295.064525135904
5736	Slovenia	1991	294.20594835575	0.732666986	2.11248528486625	292.093463070884
5737	Slovenia	1992	292.40258633938	0.737391552	2.11432432414675	290.288262015233
5738	Slovenia	1993	290.39757309739	0.741774172	2.11350904870901	288.284064048681
5739	Slovenia	1994	289.574022645	0.746234861	2.11543730743343	287.458585337567
5740	Slovenia	1995	291.40539608009	0.750994975	2.11457386434882	289.290822215741
5741	Slovenia	1996	295.56657453019	0.756348987	2.11338076212656	293.453193768063
5742	Slovenia	1997	297.6308571573	0.762275292	2.11036683057167	295.520490326728
5743	Slovenia	1998	299.20267537166	0.767728526	2.11633014040197	297.086345231258
5744	Slovenia	1999	300.72873846825	0.773236192	2.11356867456841	298.615169793682
5745	Slovenia	2000	301.9812517219	0.77957801	2.11415133166048	299.86710039024
5746	Slovenia	2001	305.07492650108	0.786240776	2.11112606023311	302.963800440847
5747	Slovenia	2002	311.66026546501	0.791805569	2.1165489809994	309.543716484011
5748	Slovenia	2003	319.94510924298	0.796371531	2.11321150759358	317.831897735386
5749	Slovenia	2004	327.55222839962	0.80096224	2.11177999565383	325.440448403966
5750	Slovenia	2005	334.70931857753	0.805601248	2.11159044948068	332.597728128049

TableS2 Frontier analysis of Smoking-Related Low Back Pain among WCBA

	location	year	val	SDI	frontier	eff_diff
5751	Slovenia	2006	342.50683217139	0.809708595	2.11357004404384	340.393262127346
5752	Slovenia	2007	350.97767272974	0.813006319	2.11238707999679	348.865285649743
5753	Slovenia	2008	359.42297829515	0.81634725	2.11193212728243	357.311046167868
5754	Slovenia	2009	367.26074702564	0.81887856	2.11512948284556	365.145617542794
5755	Slovenia	2010	372.39940489688	0.821178486	2.11388407135776	370.285520825522
5756	Slovenia	2011	375.91761893982	0.823023561	2.11239271557074	373.805226224249
5757	Slovenia	2012	378.12442372347	0.824336563	2.11233683111267	376.012086892357
5758	Slovenia	2013	379.71465082615	0.825826275	2.11375261476344	377.600898211387
5759	Slovenia	2014	380.03070526967	0.827799246	2.11139805029414	377.919307219376
5760	Slovenia	2015	380.43885236941	0.829837071	2.11112256040736	378.327729809003
5761	Slovenia	2016	377.67836929205	0.831367915	2.11389937621918	375.564469915831
5762	Slovenia	2017	372.54878685631	0.833038139	2.11089234785703	370.437894508453
5763	Slovenia	2018	367.94703594312	0.835472481	2.11100669042723	365.836029252693
5764	Slovenia	2019	365.13576641201	0.838266782	2.11598226180477	363.019784150205
5765	Slovenia	2020	365.09718829019	0.840373846	2.1136239035653	362.983564386625
5766	Slovenia	2021	361.22907052884	0.842430731	2.11552859650569	359.113541932334
5767	Solomon Islands	1990	52.341987379	0.301217167	2.40604793597965	49.9359394430204
5768	Solomon Islands	1991	51.52510641526	0.305830668	2.38103509653391	49.1440713187261
5769	Solomon Islands	1992	50.62712391978	0.311431697	2.36415801927278	48.2629659005072
5770	Solomon Islands	1993	49.95291650142	0.317151092	2.36076278253935	47.5921537188806
5771	Solomon Islands	1994	49.38243698206	0.323300247	2.33333222089469	47.0491047611653
5772	Solomon Islands	1995	48.94036908726	0.329968564	2.30505453800564	46.6353145492544
5773	Solomon Islands	1996	48.62565895063	0.336208053	2.28562316870744	46.3400357819226
5774	Solomon Islands	1997	48.39168530992	0.341835627	2.2454672769719	46.1462180329481
5775	Solomon Islands	1998	48.26200294978	0.34713506	2.14436889980851	46.1176340499715

TableS2 Frontier analysis of Smoking-Related Low Back Pain among WCBA

	location	year	val	SDI	frontier	eff_diff
5776	Solomon Islands	1999	48.34806368198	0.351867191	2.11966119770101	46.228402484279
5777	Solomon Islands	2000	48.66999110125	0.354799143	2.11977939016271	46.5502117110873
5778	Solomon Islands	2001	49.24194482941	0.356626745	2.11799646654654	47.1239483628635
5779	Solomon Islands	2002	49.95791649948	0.357910116	2.11204111896609	47.8458753805139
5780	Solomon Islands	2003	50.89335134917	0.359424359	2.12120229255424	48.7721490566158
5781	Solomon Islands	2004	51.9074442577	0.361290558	2.11315994751875	49.7942843101812
5782	Solomon Islands	2005	53.12314749552	0.363580019	2.11468791697475	51.0084595785452
5783	Solomon Islands	2006	54.54664549261	0.366443698	2.11904739559954	52.4275980970105
5784	Solomon Islands	2007	56.42669572009	0.369827951	2.112317072772	54.314378647318
5785	Solomon Islands	2008	58.45835902129	0.373764884	2.11503975755759	56.3433192637324
5786	Solomon Islands	2009	60.58482815182	0.377821028	2.11442891875527	58.4703992330647
5787	Solomon Islands	2010	62.44224918916	0.38271759	2.11298921609534	60.3292599730647
5788	Solomon Islands	2011	64.15573416635	0.388055337	2.11269318380967	62.0430409825403
5789	Solomon Islands	2012	65.99672027365	0.39300464	2.11222759156716	63.8844926820828
5790	Solomon Islands	2013	67.79250351968	0.39790625	2.11416078822672	65.6783427314533
5791	Solomon Islands	2014	69.47497630994	0.402244745	2.11307766039835	67.3618986495417
5792	Solomon Islands	2015	70.79256803396	0.406233897	2.11822575164868	68.6743422823113
5793	Solomon Islands	2016	71.98010898318	0.410406962	2.11382933112456	69.8662796520554
5794	Solomon Islands	2017	72.87770805123	0.414676394	2.11244461019039	70.7652634410396
5795	Solomon Islands	2018	73.62889126958	0.418889221	2.11432494141333	71.5145663281667
5796	Solomon Islands	2019	74.19969859248	0.422736386	2.11444594669636	72.0852526457836
5797	Solomon Islands	2020	74.17795110877	0.426112429	2.11401168432766	72.0639394244423
5798	Solomon Islands	2021	74.90304698437	0.429360316	2.1127907869029	72.7902561974671
5799	Somalia	1990	11.20325355468	0.048848564	11.1155946934667	0.0876588612132601
5800	Somalia	1991	11.35793870794	0.049422382	11.1574153744243	0.200523333515697

TableS2 Frontier analysis of Smoking-Related Low Back Pain among WCBA

	location	year	val	SDI	frontier	eff_diff
5801	Somalia	1992	11.4922788452	0.050115531	11.2103574097979	0.281921435402063
5802	Somalia	1993	11.4679571594	0.050797999	11.0699350194285	0.3980221399715
5803	Somalia	1994	11.23297269038	0.051471696	10.7230554507065	0.50991723967347
5804	Somalia	1995	10.91362196549	0.052148606	10.6727741094919	0.240847855998096
5805	Somalia	1996	10.58940390046	0.052840548	10.4989362187165	0.090467681743549
5806	Somalia	1997	10.23807827432	0.053570967	10.0515781576094	0.186500116710583
5807	Somalia	1998	9.85948948105	0.054321527	9.76936278707712	0.0901266939728842
5808	Somalia	1999	9.49039321893	0.055071838	9.45634618766574	0.0340470312642598
5809	Somalia	2000	9.14168741655	0.055842063	9.07385038230974	0.0678370342402648
5810	Somalia	2001	8.86121727016	0.056643165	8.81229079627101	0.0489264738889847
5811	Somalia	2002	8.61882220214	0.057457612	8.5496748652562	0.0691473368837983
5812	Somalia	2003	8.43734539665	0.05827215	8.29320664821211	0.144138748437895
5813	Somalia	2004	8.29963149797	0.059076945	8.26062942034444	0.0390020776255628
5814	Somalia	2005	8.20744970541	0.059898539	8.08970434567071	0.117745359739292
5815	Somalia	2006	8.11480151847	0.060741484	8.07174974881389	0.043051769656115
5816	Somalia	2007	8.05416742353	0.061598151	7.94262338557779	0.111544037952206
5817	Somalia	2008	8.01147241271	0.062475387	7.87145268850216	0.140019724207835
5818	Somalia	2009	8.01720625192	0.063382225	7.86698733185513	0.150218920064872
5819	Somalia	2010	8.0607248384	0.064322128	7.91492597640428	0.145798861995714
5820	Somalia	2011	8.12143420352	0.065306811	8.01752187298373	0.103912330536273
5821	Somalia	2012	8.1930136204	0.066354879	7.75373917894302	0.439274441456982
5822	Somalia	2013	8.2092564267	0.067459486	7.7369447123925	0.472311714307501
5823	Somalia	2014	8.21629005888	0.068609299	7.87984994675976	0.336440112120239
5824	Somalia	2015	8.23354837049	0.069811521	7.68446562314442	0.549082747345578
5825	Somalia	2016	8.27993513301	0.071064047	7.81941243682578	0.460522696184219

TableS2 Frontier analysis of Smoking-Related Low Back Pain among WCBA

	location	year	val	SDI	frontier	eff_diff
5826	Somalia	2017	8.30098322168	0.072334776	7.85144334551214	0.449539876167857
5827	Somalia	2018	8.35823768199	0.073642248	7.8308711437218	0.527366538268199
5828	Somalia	2019	8.3919197766	0.074979811	7.82812906713984	0.563790709460162
5829	Somalia	2020	8.37880160161	0.07636337	7.83127103904327	0.547530562566732
5830	Somalia	2021	8.35694630185	0.077688109	7.467452753336	0.889493548514
5831	South Africa	1990	61.72820547604	0.541571435	2.113237957233	59.614967518807
5832	South Africa	1991	59.75696318695	0.546921136	2.11122694929487	57.6457362376551
5833	South Africa	1992	58.08030528899	0.552066899	2.11198690773761	55.9683183812524
5834	South Africa	1993	56.64803705045	0.557198439	2.11447045991423	54.5335665905358
5835	South Africa	1994	55.4908800173	0.562448636	2.11572339527339	53.3751566220266
5836	South Africa	1995	54.56114347573	0.567905304	2.11818973223513	52.4429537434949
5837	South Africa	1996	53.64970574879	0.573453988	2.11266205492959	51.5370436938604
5838	South Africa	1997	52.59646545776	0.579099278	2.11336941072729	50.4830960470327
5839	South Africa	1998	51.60821314141	0.584437313	2.11135798267224	49.4968551587378
5840	South Africa	1999	50.69629041224	0.589407349	2.1117671987371	48.5845232135029
5841	South Africa	2000	50.03619831865	0.594093683	2.11431697281176	47.9218813458382
5842	South Africa	2001	49.26887305088	0.597884168	2.11755871035299	47.151314340527
5843	South Africa	2002	48.54169468035	0.600921676	2.11543357055522	46.4262611097948
5844	South Africa	2003	47.78230985729	0.603515602	2.11347456502892	45.6688352922611
5845	South Africa	2004	46.96269959389	0.606262514	2.11403768748398	44.848661906406
5846	South Africa	2005	46.06835727972	0.609989979	2.11552459579961	43.9528326839204
5847	South Africa	2006	44.87931541759	0.61501469	2.11354906633456	42.7657663512554
5848	South Africa	2007	43.47788168828	0.62060815	2.11266741872822	41.3652142695518
5849	South Africa	2008	42.03267854225	0.625948031	2.11509832518224	39.9175802170678
5850	South Africa	2009	40.68994834277	0.630645626	2.11430413252761	38.5756442102424

TableS2 Frontier analysis of Smoking-Related Low Back Pain among WCBA

	location	year	val	SDI	frontier	eff_diff
5851	South Africa	2010	39.67720811781	0.635268538	2.11203589473083	37.5651722230792
5852	South Africa	2011	38.84433299694	0.639991722	2.11251488372563	36.7318181132144
5853	South Africa	2012	38.03586186388	0.644715661	2.11221918140677	35.9236426824732
5854	South Africa	2013	37.39240677255	0.649430554	2.11713225341886	35.2752745191311
5855	South Africa	2014	36.96505570932	0.653964495	2.11231022919984	34.8527454801202
5856	South Africa	2015	36.86393465988	0.658417761	2.11410956414701	34.749825095733
5857	South Africa	2016	37.1214778783	0.662642487	2.11221970278029	35.0092581755197
5858	South Africa	2017	37.55060608052	0.666622608	2.11490534941797	35.435700731102
5859	South Africa	2018	38.03690525	0.670335365	2.11411485751975	35.9227903924802
5860	South Africa	2019	38.5262047401	0.674041037	2.11028552304527	36.4159192170547
5861	South Africa	2020	39.01168530107	0.677166096	2.11314195819036	36.8985433428796
5862	South Africa	2021	39.2124485751	0.679626598	2.11260592731536	37.0998426477846
5863	South Asia	1990	25.21463357286	0.319796517	2.34309967337617	22.8715338994838
5864	South Asia	1991	25.06103317099	0.325854134	2.32059688651955	22.7404362844705
5865	South Asia	1992	24.86378189733	0.331940961	2.3019573816526	22.5618245156774
5866	South Asia	1993	24.6066976415	0.338013384	2.271045327464	22.335652314036
5867	South Asia	1994	24.29700194099	0.344322901	2.17940290385733	22.1175990371327
5868	South Asia	1995	23.94698349584	0.350726453	2.11826189899016	21.8287215968498
5869	South Asia	1996	23.42890231135	0.357286569	2.12082150779827	21.3080808035517
5870	South Asia	1997	22.59671965385	0.363719215	2.11309420363837	20.4836254502116
5871	South Asia	1998	21.7097488161	0.370287576	2.11393123531012	19.5958175807899
5872	South Asia	1999	20.94496152192	0.376979322	2.11401185280727	18.8309496691127
5873	South Asia	2000	20.49770614797	0.383388457	2.11981450876192	18.3778916392081
5874	South Asia	2001	20.20419978733	0.389533341	2.11402147660392	18.0901783107261
5875	South Asia	2002	19.94278668419	0.395194069	2.11623081543533	17.8265558687547

TableS2 Frontier analysis of Smoking-Related Low Back Pain among WCBA

	location	year	val	SDI	frontier	eff_diff
5876	South Asia	2003	19.69961708945	0.400983424	2.11476446477846	17.5848526246715
5877	South Asia	2004	19.47956065312	0.407180554	2.11252289704988	17.3670377560701
5878	South Asia	2005	19.30119232968	0.413986658	2.11087451268551	17.1903178169945
5879	South Asia	2006	19.11432765574	0.421367361	2.11117732241582	17.0031503333242
5880	South Asia	2007	18.8769455495	0.429261848	2.11535860856646	16.7615869409335
5881	South Asia	2008	18.59904392259	0.437011859	2.11436268004565	16.4846812425443
5882	South Asia	2009	18.32130512868	0.445197355	2.11149531768654	16.2098098109935
5883	South Asia	2010	18.0546953096	0.454075792	2.11392458007338	15.9407707295266
5884	South Asia	2011	17.82560683188	0.463339273	2.11542274829357	15.7101840835864
5885	South Asia	2012	17.63166073441	0.473080558	2.11410931417964	15.5175514202304
5886	South Asia	2013	17.45997317536	0.483434269	2.11367733764918	15.3462958377108
5887	South Asia	2014	17.29950791283	0.494084362	2.11446546475279	15.1850424480772
5888	South Asia	2015	17.14859187175	0.504891461	2.11158799325959	15.0370038784904
5889	South Asia	2016	16.90098163766	0.515310997	2.11426582232988	14.7867158153301
5890	South Asia	2017	16.54053246645	0.525128437	2.11476083791446	14.4257716285355
5891	South Asia	2018	16.19119812946	0.534477408	2.11250714427197	14.078690985188
5892	South Asia	2019	16.00207277412	0.543245696	2.11206538530045	13.8900073888196
5893	South Asia	2020	16.10836339352	0.550585566	2.11292684113304	13.995436552387
5894	South Asia	2021	16.03639714858	0.557864657	2.11340062777184	13.9229965208082
5895	South Korea	1990	65.83887792915	0.692329307	2.11159817522149	63.7272797539285
5896	South Korea	1991	67.0676987715	0.702631128	2.11294861710384	64.9547501543962
5897	South Korea	1992	68.01132684906	0.712440842	2.11418831782839	65.8971385312316
5898	South Korea	1993	69.37201941691	0.72228674	2.11247489588662	67.2595445210234
5899	South Korea	1994	70.70059606839	0.732197906	2.11088595488417	68.5897101135058
5900	South Korea	1995	71.80806991347	0.74194003	2.11541868613026	69.6926512273397

TableS2 Frontier analysis of Smoking-Related Low Back Pain among WCBA

	location	year	val	SDI	frontier	eff_diff
5901	South Korea	1996	72.68946738275	0.75155324	2.11346454821962	70.5760028345304
5902	South Korea	1997	72.96522946119	0.760591683	2.11635072222132	70.8488787389687
5903	South Korea	1998	73.52013803652	0.768281885	2.11215724962351	71.4079807868965
5904	South Korea	1999	73.91833115649	0.776773436	2.11290092839277	71.8054302280972
5905	South Korea	2000	74.36110121154	0.785428821	2.11457002125606	72.2465311902839
5906	South Korea	2001	74.85127362265	0.793496281	2.11082143899958	72.7404521836504
5907	South Korea	2002	74.8768097602	0.801024295	2.11205912744262	72.7647506327574
5908	South Korea	2003	74.7199188224	0.807687786	2.11606326683368	72.6038555555663
5909	South Korea	2004	74.37699700855	0.813720927	2.11514101856282	72.2618559899872
5910	South Korea	2005	73.9448324312	0.8190934	2.11212742053941	71.8327050106606
5911	South Korea	2006	73.49543687284	0.82417982	2.11241665838608	71.3830202144539
5912	South Korea	2007	72.9296252431	0.829127953	2.11071944107915	70.8189058020208
5913	South Korea	2008	72.19264549018	0.833894627	2.11202081399788	70.0806246761821
5914	South Korea	2009	71.76993843696	0.838108331	2.11522341450558	69.6547150224544
5915	South Korea	2010	71.35013457248	0.842511429	2.1157449599775	69.2343896125025
5916	South Korea	2011	70.69766556835	0.846913229	2.10963649412075	68.5880290742293
5917	South Korea	2012	69.89826490027	0.851140263	2.11330594011855	67.7849589601515
5918	South Korea	2013	68.82443134716	0.855441	2.11274625880511	66.7116850883549
5919	South Korea	2014	67.86218379972	0.859665568	2.11201389636245	65.7501699033575
5920	South Korea	2015	67.01607822697	0.863907408	2.11248125751093	64.9035969694591
5921	South Korea	2016	66.71513353366	0.868317824	2.11381190062946	64.6013216330305
5922	South Korea	2017	66.80387318639	0.872617764	2.11395446951032	64.6899187168797
5923	South Korea	2018	66.66243334564	0.876823317	2.11181894975	64.55061439589
5924	South Korea	2019	66.28346657348	0.88073151	2.11634374605551	64.1671228274245
5925	South Korea	2020	63.10172131777	0.883717569	2.11171733757441	60.9900039801956

TableS2 Frontier analysis of Smoking-Related Low Back Pain among WCBA

	location	year	val	SDI	frontier	eff_diff
5926	South Korea	2021	64.19552045161	0.886675267	2.11147194914866	62.0840485024613
5927	South Sudan	1990	8.88717624684	0.2066565	3.47450556282611	5.41267068401389
5928	South Sudan	1991	8.82741346797	0.208932716	3.500282806778	5.327130661192
5929	South Sudan	1992	8.73607331283	0.211199401	3.43803212485725	5.29804118797274
5930	South Sudan	1993	8.63658928191	0.213416471	3.49894660114387	5.13764268076613
5931	South Sudan	1994	8.57873023891	0.215612089	3.47560254908889	5.10312768982111
5932	South Sudan	1995	8.5146988037	0.217920463	2.90390469274768	5.61079411095232
5933	South Sudan	1996	8.39684676799	0.220399437	2.93596537442608	5.46088139356392
5934	South Sudan	1997	8.28856293585	0.223037171	2.68074572529143	5.60781721055857
5935	South Sudan	1998	8.18336967558	0.225788869	2.78356151668161	5.39980815889839
5936	South Sudan	1999	8.08258860719	0.228648585	2.70171192170238	5.38087668548762
5937	South Sudan	2000	8.01153323405	0.231730344	2.64139815402061	5.37013508002939
5938	South Sudan	2001	8.01994924609	0.235014128	2.61756913244762	5.40238011364238
5939	South Sudan	2002	8.03861934028	0.238341186	2.63820523451039	5.40041410576961
5940	South Sudan	2003	8.05375997467	0.241797191	2.54927598233213	5.50448399233787
5941	South Sudan	2004	8.06821931726	0.245462499	2.55958953885807	5.50862977840193
5942	South Sudan	2005	8.08169505884	0.249280904	2.56041693357431	5.52127812526569
5943	South Sudan	2006	8.07775989293	0.253300508	2.51543933461915	5.56232055831085
5944	South Sudan	2007	8.05423504258	0.257491764	2.51168100928046	5.54255403329954
5945	South Sudan	2008	8.02995478654	0.261740458	2.50111911926956	5.52883566727044
5946	South Sudan	2009	8.02633704098	0.266179235	2.48475776370879	5.54157927727121
5947	South Sudan	2010	8.03290396187	0.270572622	2.48129866240653	5.55160529946347
5948	South Sudan	2011	8.06305006411	0.274719143	2.45443179026328	5.60861827384672
5949	South Sudan	2012	8.09553578338	0.275066143	2.45476907500029	5.6407667083797
5950	South Sudan	2013	8.11255703617	0.275813942	2.44600384381622	5.66655319235378

TableS2 Frontier analysis of Smoking-Related Low Back Pain among WCBA

	location	year	val	SDI	frontier	eff_diff
5951	South Sudan	2014	8.20047066627	0.276311461	2.46267469737081	5.73779596889919
5952	South Sudan	2015	8.35481090038	0.275833634	2.45531475031914	5.89949615006086
5953	South Sudan	2016	8.57982787996	0.274455491	2.45502589819843	6.12480198176157
5954	South Sudan	2017	8.96636786575	0.274387859	2.45044021353205	6.51592765221795
5955	South Sudan	2018	9.31319660789	0.274987848	2.45668491368328	6.85651169420672
5956	South Sudan	2019	9.30485126768	0.275706201	2.45266143097577	6.85218983670423
5957	South Sudan	2020	9.11999956884	0.276787924	2.45735515939864	6.66264440944136
5958	South Sudan	2021	8.93561741316	0.278371125	2.46296922624815	6.47264818691185
5959	Southeast Asia	1990	20.70180478852	0.46410364	2.11535308658958	18.5864517019304
5960	Southeast Asia	1991	20.84479677311	0.471749871	2.11622036575316	18.7285764073568
5961	Southeast Asia	1992	20.99101056084	0.479490406	2.11245471566578	18.8785558451742
5962	Southeast Asia	1993	21.16401338428	0.487349418	2.11378988352958	19.0502235007504
5963	Southeast Asia	1994	21.3550140355	0.495348107	2.11647613588536	19.2385378996146
5964	Southeast Asia	1995	21.52943444832	0.50335114	2.113399912789	19.416034535531
5965	Southeast Asia	1996	21.71171283408	0.511232528	2.11301082208061	19.5987020119994
5966	Southeast Asia	1997	21.89129116581	0.518664155	2.11540995301744	19.7758812127926
5967	Southeast Asia	1998	22.03598242658	0.52443938	2.11670530014059	19.9192771264394
5968	Southeast Asia	1999	22.16808207549	0.529667781	2.1130303498662	20.0550517256238
5969	Southeast Asia	2000	22.33928024114	0.534566608	2.11182227874326	20.2274579623967
5970	Southeast Asia	2001	22.43905425005	0.539010433	2.11087658917121	20.3281776608788
5971	Southeast Asia	2002	22.50121697239	0.543478548	2.11306205826756	20.3881549141224
5972	Southeast Asia	2003	22.51684748444	0.548051006	2.11193068734316	20.4049167970968
5973	Southeast Asia	2004	22.50926345216	0.552707418	2.11305489820529	20.3962085539547
5974	Southeast Asia	2005	22.48258636722	0.557391298	2.11790628046135	20.3646800867587
5975	Southeast Asia	2006	22.40954222046	0.562302171	2.11295540178238	20.2965868186776

TableS2 Frontier analysis of Smoking-Related Low Back Pain among WCBA

	location	year	val	SDI	frontier	eff_diff
5976	Southeast Asia	2007	22.2970194136	0.567654259	2.11493367974983	20.1820857338502
5977	Southeast Asia	2008	22.13630950339	0.573208389	2.11280717189942	20.0235023314906
5978	Southeast Asia	2009	21.93790481815	0.578497394	2.11680919562314	19.8210956225269
5979	Southeast Asia	2010	21.76593461652	0.58426847	2.11577541091985	19.6501592056001
5980	Southeast Asia	2011	21.57266000732	0.590414618	2.1147121836325	19.4579478236875
5981	Southeast Asia	2012	21.27264217076	0.596780286	2.11476631591776	19.1578758548422
5982	Southeast Asia	2013	20.93322386081	0.60314492	2.11415640344785	18.8190674573621
5983	Southeast Asia	2014	20.61070877196	0.609438681	2.1155518448884	18.4951569270716
5984	Southeast Asia	2015	20.35791685641	0.615689065	2.11211634133529	18.2458005150747
5985	Southeast Asia	2016	20.17345295248	0.621839858	2.11089878712297	18.062554165357
5986	Southeast Asia	2017	20.01422265788	0.627978129	2.11473174517618	17.8994909127038
5987	Southeast Asia	2018	19.89133799145	0.634095876	2.11112740576379	17.7802105856862
5988	Southeast Asia	2019	19.80481705396	0.640106755	2.11353407696831	17.6912829769917
5989	Southeast Asia	2020	19.74365390766	0.645069663	2.11477918150493	17.6288747261551
5990	Southeast Asia	2021	19.63952140856	0.649777295	2.111919460315	17.527601948245
5991	Southern Latin America	1990	290.41039766577	0.587308121	2.11263988157679	288.297757784193
5992	Southern Latin America	1991	288.74404629935	0.592118694	2.11488637115423	286.629159928196
5993	Southern Latin America	1992	287.38942033294	0.598079761	2.1143455914208	285.275074741519
5994	Southern Latin America	1993	286.55471977744	0.603588571	2.11381433970873	284.440905437731
5995	Southern Latin America	1994	286.28552081872	0.609392016	2.11121536636303	284.174305452357
5996	Southern Latin America	1995	285.95544520304	0.614372849	2.111254751919	283.844190451121
5997	Southern Latin America	1996	286.4884872734	0.619260048	2.11190297203689	284.376584301363
5998	Southern Latin America	1997	286.54266537264	0.624756487	2.11350129926138	284.429164073379
5999	Southern Latin America	1998	286.91403750468	0.630349086	2.11463344090518	284.799404063775
6000	Southern Latin America	1999	287.26932526982	0.635748461	2.11291621985571	285.156409049964

TableS2 Frontier analysis of Smoking-Related Low Back Pain among WCBA

	location	year	val	SDI	frontier	eff_diff
6001	Southern Latin America	2000	287.73672162303	0.641237434	2.11165495972788	285.625066663302
6002	Southern Latin America	2001	288.52637545379	0.646040371	2.11382310105225	286.412552352738
6003	Southern Latin America	2002	290.20158588275	0.649825844	2.11298481961053	288.088601063139
6004	Southern Latin America	2003	291.43513356459	0.652552403	2.11230163683946	289.322831927751
6005	Southern Latin America	2004	292.20787164927	0.655718876	2.11293592023222	290.094935729038
6006	Southern Latin America	2005	291.18491972954	0.660519129	2.1159055026	289.06901422694
6007	Southern Latin America	2006	287.8828104911	0.664386241	2.11554863033547	285.767261860765
6008	Southern Latin America	2007	282.7978374635	0.6673925	2.11286491773961	280.68497254576
6009	Southern Latin America	2008	277.09186021449	0.670688619	2.11332345272381	274.978536761766
6010	Southern Latin America	2009	271.63023511779	0.67415239	2.11561605074456	269.514619067045
6011	Southern Latin America	2010	266.93085839057	0.678761222	2.11121880571592	264.819639584854
6012	Southern Latin America	2011	262.54519531068	0.684134275	2.11518283976286	260.430012470917
6013	Southern Latin America	2012	257.53971430538	0.688427908	2.11321833935215	255.426495966028
6014	Southern Latin America	2013	252.39861978293	0.691715992	2.11425122756274	250.284368555367
6015	Southern Latin America	2014	248.30661095138	0.696039552	2.114587630564	246.192023320816
6016	Southern Latin America	2015	246.07194420271	0.703287229	2.11535657481685	243.956587627893
6017	Southern Latin America	2016	246.41521465956	0.710787735	2.11406032423838	244.301154335322
6018	Southern Latin America	2017	248.28656116489	0.717548418	2.11128219476922	246.175278970121
6019	Southern Latin America	2018	250.25016060307	0.724952157	2.11396615916476	248.136194443905
6020	Southern Latin America	2019	251.63930859824	0.731133817	2.11485084136413	249.524457756876
6021	Southern Latin America	2020	242.85118105238	0.733963357	2.11065287512583	240.740528177254
6022	Southern Latin America	2021	259.78432573974	0.735984717	2.11792455253484	257.666401187205
6023	Southern sub-Saharan Africa	1990	47.76727023444	0.506946633	2.11476843997163	45.6525017944684
6024	Southern sub-Saharan Africa	1991	46.29927335327	0.512377786	2.11546625254239	44.1838071007276
6025	Southern sub-Saharan Africa	1992	45.06355749267	0.517569473	2.11248884596032	42.9510686467097

TableS2 Frontier analysis of Smoking-Related Low Back Pain among WCBA

	location	year	val	SDI	frontier	eff_diff
6026	Southern sub-Saharan Africa	1993	44.0202222198	0.522772503	2.11412066755036	41.9061015522496
6027	Southern sub-Saharan Africa	1994	43.19958623084	0.528063317	2.11425352016884	41.0853327106712
6028	Southern sub-Saharan Africa	1995	42.5556059878	0.533379781	2.11435477971295	40.4412512080871
6029	Southern sub-Saharan Africa	1996	41.94664818057	0.538780402	2.11220199739629	39.8344461831737
6030	Southern sub-Saharan Africa	1997	41.24651068756	0.544134279	2.11402305319346	39.1324876343665
6031	Southern sub-Saharan Africa	1998	40.6004639045	0.549141665	2.11492972849111	38.4855341760089
6032	Southern sub-Saharan Africa	1999	40.02260125188	0.553884659	2.11436010579487	37.9082411460851
6033	Southern sub-Saharan Africa	2000	39.63236789546	0.558490622	2.1131708445297	37.5191970509303
6034	Southern sub-Saharan Africa	2001	39.13399637683	0.562511271	2.11407578631942	37.0199205905106
6035	Southern sub-Saharan Africa	2002	38.65779214041	0.56604984	2.1186063111976	36.5391858292124
6036	Southern sub-Saharan Africa	2003	38.14969490704	0.569215186	2.1133420653504	36.0363528416896
6037	Southern sub-Saharan Africa	2004	37.57768914521	0.572389699	2.11441309805042	35.4632760471596
6038	Southern sub-Saharan Africa	2005	36.93647391319	0.576115686	2.11428516303464	34.8221887501554
6039	Southern sub-Saharan Africa	2006	36.04168746551	0.580684833	2.11186639451169	33.9298210709983
6040	Southern sub-Saharan Africa	2007	34.95280380768	0.585672998	2.11536463840848	32.8374391692715
6041	Southern sub-Saharan Africa	2008	33.81530854	0.590405319	2.11334932376393	31.7019592162361
6042	Southern sub-Saharan Africa	2009	32.73897291511	0.594585068	2.11668164617215	30.6222912689378
6043	Southern sub-Saharan Africa	2010	31.91272088446	0.598835553	2.11691041076683	29.7958104736932
6044	Southern sub-Saharan Africa	2011	31.2262643892	0.603346853	2.11249342615326	29.1137709630467
6045	Southern sub-Saharan Africa	2012	30.54693450378	0.60803777	2.1118660607491	28.4350684430309
6046	Southern sub-Saharan Africa	2013	29.9877955152	0.612843814	2.11306290686069	27.8747326083393
6047	Southern sub-Saharan Africa	2014	29.59730335239	0.617452594	2.11161624113955	27.4856871112504
6048	Southern sub-Saharan Africa	2015	29.45497339525	0.62183758	2.11418279394844	27.3407906013016
6049	Southern sub-Saharan Africa	2016	29.60349770332	0.625919059	2.11162170696448	27.4918759963555
6050	Southern sub-Saharan Africa	2017	29.87916234608	0.629761503	2.11441390759866	27.7647484384813

TableS2 Frontier analysis of Smoking-Related Low Back Pain among WCBA

	location	year	val	SDI	frontier	eff_diff
6051	Southern sub-Saharan Africa	2018	30.20845531103	0.633410656	2.11640259363	28.0920527174
6052	Southern sub-Saharan Africa	2019	30.53092852807	0.63694019	2.11483451878606	28.4160940092839
6053	Southern sub-Saharan Africa	2020	30.83788770421	0.639881827	2.11241417989931	28.7254735243107
6054	Southern sub-Saharan Africa	2021	30.92201000752	0.642200282	2.11533762705986	28.8066723804601
6055	Spain	1990	196.0985082095	0.636673166	2.11196858807162	193.986539621428
6056	Spain	1991	190.82824795252	0.644130745	2.11133173496481	188.716916217555
6057	Spain	1992	186.79250218434	0.651597597	2.11495168726667	184.677550497073
6058	Spain	1993	183.88352516143	0.658637569	2.11117240249738	181.772352758933
6059	Spain	1994	182.77741626884	0.665321549	2.1140842929578	180.663331975882
6060	Spain	1995	184.53562697284	0.671576034	2.11562739876477	182.419999574075
6061	Spain	1996	188.07906446174	0.677088943	2.11553122156574	185.963533240174
6062	Spain	1997	192.47035860016	0.682429497	2.1145855120975	190.355773088062
6063	Spain	1998	197.5701983506	0.687490717	2.11513077307289	195.455067577527
6064	Spain	1999	203.07006252228	0.692245197	2.114718155998	200.955344366282
6065	Spain	2000	209.05125821719	0.697056031	2.11226671259987	206.93899150459
6066	Spain	2001	215.62095594748	0.70172607	2.11190758657184	213.509048360908
6067	Spain	2002	223.25659652675	0.706309655	2.11241828150678	221.144178245243
6068	Spain	2003	230.78668156968	0.710678584	2.11231594633969	228.67436562334
6069	Spain	2004	237.32463547696	0.71488623	2.11268231112397	235.211953165836
6070	Spain	2005	242.65666390006	0.718717289	2.11238965295114	240.544274247109
6071	Spain	2006	245.53566373569	0.721970128	2.11625899623169	243.419404739458
6072	Spain	2007	247.2086163216	0.725211443	2.11413121685689	245.094485104743
6073	Spain	2008	247.90321379486	0.729411041	2.11307715021869	245.790136644641
6074	Spain	2009	247.67120960925	0.733956055	2.11185386362988	245.55935574562
6075	Spain	2010	248.30014883646	0.738352055	2.11247330474901	246.187675531711

TableS2 Frontier analysis of Smoking-Related Low Back Pain among WCBA

	location	year	val	SDI	frontier	eff_diff
6076	Spain	2011	247.97028023694	0.74209645	2.1119899813469	245.858290255593
6077	Spain	2012	246.3682472397	0.745181867	2.11175882695792	244.256488412742
6078	Spain	2013	243.78764518095	0.747651982	2.11245291936418	241.675192261586
6079	Spain	2014	240.4267068203	0.749800929	2.11151711516211	238.315189705138
6080	Spain	2015	237.12064051981	0.752269823	2.11376419144649	235.006876328364
6081	Spain	2016	234.48747549857	0.754757939	2.1144582489187	232.373017249651
6082	Spain	2017	232.14454481383	0.757663276	2.11300560048766	230.031539213342
6083	Spain	2018	228.89864522809	0.760659575	2.112254070814	226.786391157276
6084	Spain	2019	225.01768722641	0.763942918	2.11182216916113	222.905865057249
6085	Spain	2020	218.53965202906	0.766506025	2.1149816552438	216.424670373816
6086	Spain	2021	215.37832514578	0.769283698	2.11483153044872	213.263493615331
6087	Sri Lanka	1990	15.3747247392	0.522622553	2.11349839136101	13.261226347839
6088	Sri Lanka	1991	15.43854245741	0.528239203	2.11182285435451	13.3267196030555
6089	Sri Lanka	1992	15.45562937963	0.533345535	2.11113706725613	13.3444923123739
6090	Sri Lanka	1993	15.43866324655	0.538891102	2.11684207196052	13.3218211745895
6091	Sri Lanka	1994	15.32938891183	0.545124551	2.11480644689974	13.2145824649303
6092	Sri Lanka	1995	15.13805353269	0.551744058	2.11418561383397	13.023867918856
6093	Sri Lanka	1996	14.8994366073	0.558938888	2.11316633256362	12.7862702747364
6094	Sri Lanka	1997	14.62145588794	0.566128422	2.11696209014188	12.5044937977981
6095	Sri Lanka	1998	14.25570730067	0.572326187	2.11310031523516	12.1426069854348
6096	Sri Lanka	1999	13.86204285498	0.577630048	2.11190020726679	11.7501426477132
6097	Sri Lanka	2000	13.49902577182	0.582942638	2.11270513580099	11.386320636019
6098	Sri Lanka	2001	13.0863671949	0.587556599	2.11282118275697	10.973546012143
6099	Sri Lanka	2002	12.62175431147	0.592359127	2.11282046636747	10.5089338451025
6100	Sri Lanka	2003	12.18058169768	0.597410284	2.11051027977534	10.0700714179047

TableS2 Frontier analysis of Smoking-Related Low Back Pain among WCBA

	location	year	val	SDI	frontier	eff_diff
6101	Sri Lanka	2004	11.79350283594	0.602250062	2.11437170015903	9.67913113578097
6102	Sri Lanka	2005	11.56465155117	0.606906656	2.11274090006643	9.45191065110357
6103	Sri Lanka	2006	11.40169841454	0.611807083	2.11202519931485	9.28967321522515
6104	Sri Lanka	2007	11.27218379978	0.616979265	2.11308559592143	9.15909820385857
6105	Sri Lanka	2008	11.18486859459	0.622414317	2.11864968161206	9.06621891297794
6106	Sri Lanka	2009	11.12994000658	0.62814963	2.11760473454177	9.01233527203823
6107	Sri Lanka	2010	11.08447544541	0.634263123	2.11521644072939	8.96925900468061
6108	Sri Lanka	2011	11.07058973192	0.640799571	2.11453478051427	8.95605495140573
6109	Sri Lanka	2012	11.05610178345	0.648049816	2.11049828529849	8.94560349815151
6110	Sri Lanka	2013	11.04637559604	0.655435838	2.11072913752232	8.93564645851768
6111	Sri Lanka	2014	11.04449600878	0.662782434	2.11629876485113	8.92819724392887
6112	Sri Lanka	2015	11.01752151265	0.669760292	2.11073448804494	8.90678702460506
6113	Sri Lanka	2016	10.95213692087	0.676277441	2.11169853874508	8.84043838212492
6114	Sri Lanka	2017	10.84128029486	0.682405274	2.11244212851146	8.72883816634854
6115	Sri Lanka	2018	10.72928973531	0.688125858	2.111657719844	8.617632015466
6116	Sri Lanka	2019	10.6439239915	0.693516063	2.11322711138638	8.53069688011362
6117	Sri Lanka	2020	10.5890606935	0.697648535	2.11293174264184	8.47612895085816
6118	Sri Lanka	2021	10.4806941935	0.701534935	2.11167827659391	8.36901591690609
6119	Sudan	1990	19.46148865637	0.292178643	2.43864202816914	17.0228466282009
6120	Sudan	1991	19.27854701992	0.296473446	2.3967093072224	16.8818377126976
6121	Sudan	1992	19.14486999474	0.300863677	2.39742155401075	16.7474484407293
6122	Sudan	1993	19.07215291102	0.305409326	2.38192823168845	16.6902246793315
6123	Sudan	1994	19.02255119831	0.31009643	2.38650895783648	16.6360422404735
6124	Sudan	1995	18.94469869978	0.315208259	2.363383461715	16.581315238065
6125	Sudan	1996	18.9037267597	0.320680229	2.34174730242113	16.5619794572789

TableS2 Frontier analysis of Smoking-Related Low Back Pain among WCBA

	location	year	val	SDI	frontier	eff_diff
6126	Sudan	1997	18.89394233145	0.326761462	2.31961779643941	16.5743245350106
6127	Sudan	1998	18.86898495944	0.333087761	2.28709220133759	16.5818927581024
6128	Sudan	1999	18.81371018601	0.339754884	2.26768546500516	16.5460247210048
6129	Sudan	2000	18.84201199081	0.346875809	2.14182993833053	16.7001820524795
6130	Sudan	2001	18.81573570663	0.354215515	2.11310878735673	16.7026269192733
6131	Sudan	2002	18.78541422369	0.361728237	2.11668782994324	16.6687263937468
6132	Sudan	2003	18.78612059438	0.369693653	2.11329501093417	16.6728255834458
6133	Sudan	2004	18.80205675558	0.377920656	2.11263655691203	16.689420198668
6134	Sudan	2005	18.79823461812	0.386685712	2.11535024051544	16.6828843776046
6135	Sudan	2006	18.65246251958	0.396151374	2.11204455302561	16.5404179665544
6136	Sudan	2007	18.40260200291	0.406079992	2.11405788563194	16.2885441172781
6137	Sudan	2008	18.04917676433	0.4160876	2.1167903407872	15.9323864235428
6138	Sudan	2009	17.7659309872	0.425975917	2.11466047831791	15.6512705088821
6139	Sudan	2010	17.48626147159	0.436171097	2.11326867313683	15.3729927984532
6140	Sudan	2011	17.23440559537	0.447239442	2.11575048193791	15.1186551134321
6141	Sudan	2012	16.91144060225	0.456790265	2.11477299738813	14.7966676048619
6142	Sudan	2013	16.56157978637	0.466435995	2.11350159401926	14.4480781923507
6143	Sudan	2014	16.2629509817	0.476362135	2.11669038916739	14.1462605925326
6144	Sudan	2015	16.1510393141	0.486549437	2.11489648699119	14.0361428271088
6145	Sudan	2016	16.12770988875	0.496848219	2.1133785658736	14.0143313228764
6146	Sudan	2017	16.13115465516	0.506883534	2.11557791791865	14.0155767372414
6147	Sudan	2018	16.1202812615	0.516214086	2.1147466671	14.0055345944
6148	Sudan	2019	16.12422759386	0.525009122	2.11121750651068	14.0130100873493
6149	Sudan	2020	16.17863678301	0.533455422	2.11222442410567	14.0664123589043
6150	Sudan	2021	16.21886155769	0.541949735	2.11070532118527	14.1081562365047

TableS2 Frontier analysis of Smoking-Related Low Back Pain among WCBA

	location	year	val	SDI	frontier	eff_diff
6151	Suriname	1990	59.62417873689	0.502054305	2.11155357824839	57.5126251586416
6152	Suriname	1991	60.88391582448	0.506655795	2.11240524821438	58.7715105762656
6153	Suriname	1992	62.13549131015	0.510959772	2.10977673811453	60.0257145720355
6154	Suriname	1993	63.39158295182	0.514419437	2.11381572890633	61.2777672229137
6155	Suriname	1994	64.50816573317	0.517650245	2.11185978191387	62.3963059512561
6156	Suriname	1995	65.22886289575	0.521002622	2.1152724454539	63.1135904502961
6157	Suriname	1996	65.95581268048	0.525059318	2.11533551362437	63.8404771668556
6158	Suriname	1997	66.81140999945	0.529750812	2.11645611590507	64.6949538835449
6159	Suriname	1998	67.45754290491	0.534599696	2.11492922935657	65.3426136755534
6160	Suriname	1999	68.08540390281	0.538932791	2.10946046862894	65.9759434341811
6161	Suriname	2000	68.73596419345	0.543660023	2.11348920513684	66.6224749883132
6162	Suriname	2001	69.01806169594	0.548683524	2.11409916084281	66.9039625350972
6163	Suriname	2002	69.16553166981	0.553879432	2.11301328463519	67.0525183851748
6164	Suriname	2003	68.89571749928	0.559418373	2.11426485902269	66.7814526402573
6165	Suriname	2004	68.39870327027	0.565109708	2.11767180712792	66.2810314631421
6166	Suriname	2005	67.87227165973	0.570462186	2.11311830797218	65.7591533517578
6167	Suriname	2006	67.07293337042	0.575647048	2.11003176190588	64.9629016085141
6168	Suriname	2007	65.93856219802	0.580654574	2.11647886324988	63.8220833347701
6169	Suriname	2008	64.64839539022	0.585644825	2.11075452801719	62.5376408622028
6170	Suriname	2009	63.24590881142	0.590512805	2.11351631906284	61.1323924923572
6171	Suriname	2010	61.99331042636	0.595469745	2.11317786604787	59.8801325603121
6172	Suriname	2011	60.73124347463	0.600356336	2.11214708370045	58.6190963909295
6173	Suriname	2012	59.59776161558	0.60474187	2.1104187043475	57.4873429112325
6174	Suriname	2013	58.59115633499	0.608910612	2.1120219854753	56.4791343495147
6175	Suriname	2014	57.91552220133	0.612704423	2.11293961868459	55.8025825826454

TableS2 Frontier analysis of Smoking-Related Low Back Pain among WCBA

	location	year	val	SDI	frontier	eff_diff
6176	Suriname	2015	57.21923104113	0.616097315	2.11299432852352	55.1062367126065
6177	Suriname	2016	56.89693024137	0.618868088	2.11323704010195	54.783693201268
6178	Suriname	2017	56.51461971004	0.62168418	2.1110663687087	54.4035533413313
6179	Suriname	2018	56.27786940356	0.624708011	2.11652534123276	54.1613440623272
6180	Suriname	2019	56.05964748085	0.627896781	2.11956143449222	53.9400860463578
6181	Suriname	2020	55.50074464404	0.630683705	2.11284569545915	53.3878989485808
6182	Suriname	2021	54.99475014207	0.633665739	2.11310998930587	52.8816401527641
6183	Swaziland	1990	7.46410939298	0.399420955	2.11363707440794	5.35047231857206
6184	Swaziland	1991	7.36040980664	0.408269969	2.11290523692684	5.24750456971316
6185	Swaziland	1992	7.2662549326	0.416683379	2.11400756280737	5.15224736979263
6186	Swaziland	1993	7.15093132031	0.424802995	2.10992026160692	5.04101105870308
6187	Swaziland	1994	7.05294290088	0.432503472	2.11356061713458	4.93938228374542
6188	Swaziland	1995	6.94146921933	0.440127047	2.11170217787741	4.82976704145259
6189	Swaziland	1996	6.83880755028	0.447399238	2.11198320725919	4.72682434302081
6190	Swaziland	1997	6.7391778894	0.454089854	2.11687892318846	4.62229896621154
6191	Swaziland	1998	6.63280881922	0.460136984	2.11216992542519	4.52063889379481
6192	Swaziland	1999	6.49986720375	0.465990845	2.11279353156864	4.38707367218136
6193	Swaziland	2000	6.35140822314	0.471852843	2.11727357782671	4.23413464531329
6194	Swaziland	2001	6.1941316634	0.477233374	2.11266949217769	4.08146217122231
6195	Swaziland	2002	6.00891014577	0.482474423	2.11600651130671	3.89290363446329
6196	Swaziland	2003	5.8270500501	0.487608829	2.11126751436651	3.71578253573349
6197	Swaziland	2004	5.63867571994	0.492669159	2.11659062493531	3.52208509500469
6198	Swaziland	2005	5.44360199974	0.498043849	2.11364955456808	3.32995244517192
6199	Swaziland	2006	5.26425197217	0.503658294	2.11193337270065	3.15231859946935
6200	Swaziland	2007	5.07216723091	0.509260219	2.11317325575455	2.95899397515545

TableS2 Frontier analysis of Smoking-Related Low Back Pain among WCBA

	location	year	val	SDI	frontier	eff_diff
6201	Swaziland	2008	4.89597458762	0.514730896	2.11284484918258	2.78312973843742
6202	Swaziland	2009	4.75904139352	0.520427398	2.11688742928779	2.64215396423221
6203	Swaziland	2010	4.65831351663	0.526245802	2.114875059197	2.543438457433
6204	Swaziland	2011	4.59707687507	0.53191391	2.11255714891917	2.48451972615083
6205	Swaziland	2012	4.54884088077	0.537798023	2.11428410451726	2.43455677625274
6206	Swaziland	2013	4.52284635568	0.543836416	2.112312130684	2.410534224996
6207	Swaziland	2014	4.52062351103	0.549613848	2.11280228855095	2.40782122247905
6208	Swaziland	2015	4.55389253971	0.555373739	2.1126716872205	2.4412208524895
6209	Swaziland	2016	4.64545930986	0.561061186	2.11097339158678	2.53448591827322
6210	Swaziland	2017	4.79963462029	0.566698493	2.11116412230928	2.68847049798072
6211	Swaziland	2018	4.9600142977	0.572154807	2.11158617495537	2.84842812274463
6212	Swaziland	2019	5.09077230832	0.577206305	2.1160672231596	2.9747050851604
6213	Swaziland	2020	5.17110290863	0.581653071	2.11121497033726	3.05988793829274
6214	Swaziland	2021	5.20393766042	0.585459713	2.11014771491848	3.09378994550152
6215	Sweden	1990	140.04897119892	0.785535792	2.11243475194529	137.936536446975
6216	Sweden	1991	139.2685691721	0.790096441	2.11219376584398	137.156375406256
6217	Sweden	1992	138.60155795674	0.795431458	2.11961789802462	136.481940058715
6218	Sweden	1993	137.70129495137	0.801009522	2.1142851697738	135.587009781596
6219	Sweden	1994	136.814373883	0.806700264	2.11295637083092	134.701417512169
6220	Sweden	1995	135.96144518294	0.812244654	2.11431799001371	133.847127192926
6221	Sweden	1996	134.74510161798	0.817227025	2.11067707780686	132.634424540173
6222	Sweden	1997	133.53226494163	0.821694059	2.11548808095157	131.416776860678
6223	Sweden	1998	132.11717557628	0.825731995	2.11407545105791	130.003100125222
6224	Sweden	1999	130.29125588822	0.829487099	2.11218145425812	128.179074433962
6225	Sweden	2000	128.81181380989	0.833028112	2.11311157825232	126.698702231638

TableS2 Frontier analysis of Smoking-Related Low Back Pain among WCBA

	location	year	val	SDI	frontier	eff_diff
6226	Sweden	2001	127.1173697576	0.835956398	2.112250557199	125.005119200401
6227	Sweden	2002	125.31500205724	0.838741527	2.11350767642984	123.20149438081
6228	Sweden	2003	122.77181865906	0.841663138	2.11438247250118	120.657436186559
6229	Sweden	2004	120.5749450572	0.844506882	2.11771148305689	118.457233574143
6230	Sweden	2005	117.92445694528	0.846876344	2.11220943278839	115.812247512492
6231	Sweden	2006	114.82070722118	0.849115871	2.11457395093053	112.706133270249
6232	Sweden	2007	111.50089724606	0.851420847	2.11433012928373	109.386567116776
6233	Sweden	2008	108.66933614086	0.853705851	2.11243317954066	106.556902961319
6234	Sweden	2009	106.46445789901	0.855702748	2.11230059176019	104.35215730725
6235	Sweden	2010	105.61405002716	0.858727941	2.11405249940644	103.499997527754
6236	Sweden	2011	105.47363225351	0.861892379	2.11307248340768	103.360559770102
6237	Sweden	2012	106.25923904801	0.864706672	2.11540738974159	104.143831658268
6238	Sweden	2013	107.46153093928	0.867383768	2.1129257164071	105.348605222873
6239	Sweden	2014	108.94054291747	0.869923325	2.11108906016101	106.829453857309
6240	Sweden	2015	111.465758982	0.872388515	2.11321055361615	109.352548428384
6241	Sweden	2016	116.80745173683	0.874661655	2.11215029865073	114.695301438179
6242	Sweden	2017	124.7616858507	0.877120611	2.11190906483338	122.649776785867
6243	Sweden	2018	132.58933674265	0.879888912	2.11355548774027	130.47578125491
6244	Sweden	2019	137.12578723411	0.882984116	2.11013133685586	135.015655897254
6245	Sweden	2020	137.15671630233	0.88506201	2.11652054683463	135.040195755495
6246	Sweden	2021	140.02315291237	0.886880299	2.11313338345169	137.910019528918
6247	Switzerland	1990	288.19351359378	0.862766844	2.11534376532571	286.078169828454
6248	Switzerland	1991	291.99403371331	0.864796867	2.11598620648678	289.878047506823
6249	Switzerland	1992	296.50494841449	0.867736079	2.11369222122518	294.391256193265
6250	Switzerland	1993	300.51277816217	0.870863558	2.11453363364396	298.398244528526

TableS2 Frontier analysis of Smoking-Related Low Back Pain among WCBA

	location	year	val	SDI	frontier	eff_diff
6251	Switzerland	1994	303.55027896386	0.873140871	2.1133688157281	301.436910148132
6252	Switzerland	1995	305.78091455501	0.874835331	2.11296755349257	303.667947001517
6253	Switzerland	1996	306.82572643102	0.876640557	2.11379976831028	304.71192666271
6254	Switzerland	1997	306.17328825219	0.878485545	2.11233290747794	304.060955344712
6255	Switzerland	1998	305.26171845084	0.880035152	2.11103651767625	303.150681933164
6256	Switzerland	1999	303.12757854637	0.882045586	2.11305784737486	301.014520698995
6257	Switzerland	2000	300.62518967894	0.885243531	2.11578355933817	298.509406119602
6258	Switzerland	2001	296.47465376087	0.888445951	2.11961380400083	294.355039956869
6259	Switzerland	2002	290.84910189829	0.890902811	2.11317185313333	288.735930045157
6260	Switzerland	2003	284.22750087031	0.892780807	2.1138408592962	282.113660011014
6261	Switzerland	2004	277.93710446458	0.894909812	2.11162049017433	275.825483974406
6262	Switzerland	2005	272.64664830456	0.89727263	2.11211133095145	270.534536973609
6263	Switzerland	2006	270.24669961725	0.899962305	2.11493260432	268.13176701293
6264	Switzerland	2007	270.5182799405	0.902913413	2.11423689936691	268.404043041133
6265	Switzerland	2008	271.75435685899	0.905778454	2.11764421108477	269.636712647905
6266	Switzerland	2009	272.95575387871	0.908049998	2.11249138599779	270.843262492712
6267	Switzerland	2010	272.52117519308	0.910530522	2.11721871779307	270.403956475287
6268	Switzerland	2011	269.2655745906	0.913276573	2.11042553186	267.15514905874
6269	Switzerland	2012	265.92650404735	0.915714893	2.11377788364414	263.812726163706
6270	Switzerland	2013	261.64568734698	0.917955006	2.11295434728164	259.532732999698
6271	Switzerland	2014	257.48055763443	0.919994182	2.11235402729169	255.368203607138
6272	Switzerland	2015	254.0875294283	0.921832991	2.11158188463241	251.975947543668
6273	Switzerland	2016	249.84488126978	0.923930598	2.11069089432633	247.734190375454
6274	Switzerland	2017	243.85988410438	0.926202291	2.11321604636521	241.746668058015
6275	Switzerland	2018	238.68262677288	0.92855059	2.11517853591931	236.567448236961

TableS2 Frontier analysis of Smoking-Related Low Back Pain among WCBA

	location	year	val	SDI	frontier	eff_diff
6276	Switzerland	2019	235.84678335287	0.930682102	2.11526351294836	233.731519839922
6277	Switzerland	2020	234.71872933658	0.932027645	2.11174474287902	232.606984593701
6278	Switzerland	2021	235.70157276356	0.933059111	2.11028814052636	233.591284623034
6279	Syria	1990	45.38403883978	0.430492643	2.11213814116767	43.2719006986123
6280	Syria	1991	44.88314275329	0.437907493	2.11306170961096	42.770081043679
6281	Syria	1992	44.58549387994	0.445904236	2.1099488769775	42.4755450029625
6282	Syria	1993	44.2377183512	0.45414786	2.1117545525019	42.1259637986981
6283	Syria	1994	43.95708597046	0.462437298	2.11291273166902	41.844173238791
6284	Syria	1995	43.83811224643	0.470692043	2.11259509951516	41.7255171469148
6285	Syria	1996	43.73986737742	0.478743085	2.11169262591292	41.6281747515071
6286	Syria	1997	43.59281598693	0.48623235	2.1116144883011	41.4812014986289
6287	Syria	1998	43.48757601952	0.493707992	2.11209833045985	41.3754776890602
6288	Syria	1999	43.37580171283	0.500474869	2.11532336256557	41.2604783502644
6289	Syria	2000	43.13529446689	0.507143686	2.11066079960597	41.024633667284
6290	Syria	2001	42.77721299954	0.513850111	2.11183741060214	40.6653755889379
6291	Syria	2002	42.30981676328	0.521309744	2.11297080107566	40.1968459622043
6292	Syria	2003	41.70517356714	0.528935756	2.11372313339349	39.5914504337465
6293	Syria	2004	41.06685062917	0.538084469	2.11253475919619	38.9543158699738
6294	Syria	2005	40.65694335034	0.549750449	2.11148861154473	38.5454547387953
6295	Syria	2006	40.29471748048	0.560484761	2.11187220417319	38.1828452763068
6296	Syria	2007	39.64920276169	0.57002812	2.11082557019831	37.5383771914917
6297	Syria	2008	39.21424884339	0.578990493	2.11491696824784	37.0993318751422
6298	Syria	2009	39.36589466204	0.587789118	2.11506087060382	37.2508337914362
6299	Syria	2010	39.60451369666	0.595967764	2.11483039328672	37.4896833033733
6300	Syria	2011	39.8343177455	0.601524456	2.1145009152506	37.7198168302494

TableS2 Frontier analysis of Smoking-Related Low Back Pain among WCBA

	location	year	val	SDI	frontier	eff_diff
6301	Syria	2012	40.31796538657	0.602872601	2.11108000633942	38.2068853802306
6302	Syria	2013	41.92694403117	0.602410134	2.11703913152379	39.8099048996462
6303	Syria	2014	44.53131348026	0.60223576	2.11188062162706	42.4194328586329
6304	Syria	2015	46.91766731837	0.602282914	2.11407710199103	44.803590216379
6305	Syria	2016	48.28661600777	0.602911181	2.11361863564328	46.1729973721267
6306	Syria	2017	48.68972445017	0.604896366	2.11488667772095	46.5748377724491
6307	Syria	2018	48.5482967419	0.608507164	2.11468462754239	46.4336121143576
6308	Syria	2019	47.7071961697	0.613336836	2.11207536183338	45.5951208078666
6309	Syria	2020	46.58149628368	0.617727593	2.11619720383824	44.4652990798418
6310	Syria	2021	44.8040498511	0.623004075	2.11372955263587	42.6903202984641
6311	Taiwan (Province of China)	1990	50.01031886141	0.667633854	2.11484515497344	47.8954737064366
6312	Taiwan (Province of China)	1991	50.60387356687	0.676537237	2.11207286416986	48.4918007027001
6313	Taiwan (Province of China)	1992	51.19903026379	0.684803636	2.11161185458098	49.087418409209
6314	Taiwan (Province of China)	1993	51.86667654046	0.69292413	2.11791041734312	49.7487661231169
6315	Taiwan (Province of China)	1994	52.24547651126	0.70107875	2.11294119922319	50.1325353120368
6316	Taiwan (Province of China)	1995	52.82975180293	0.709626821	2.11299587867726	50.7167559242527
6317	Taiwan (Province of China)	1996	53.18367401676	0.719128415	2.11248188503364	51.0711921317264
6318	Taiwan (Province of China)	1997	53.29988066047	0.729145497	2.11383813992823	51.1860425205418
6319	Taiwan (Province of China)	1998	53.34628032744	0.738125787	2.11368113661169	51.2325991908283
6320	Taiwan (Province of China)	1999	53.47398127041	0.745338399	2.11272439712677	51.3612568732832
6321	Taiwan (Province of China)	2000	53.52283240449	0.752926841	2.11278990652328	51.4100424979667
6322	Taiwan (Province of China)	2001	53.40292175841	0.760829809	2.11329316924701	51.289628589163
6323	Taiwan (Province of China)	2002	53.34334699274	0.769008984	2.11135861131218	51.2319883814278
6324	Taiwan (Province of China)	2003	53.13803357173	0.776955803	2.11545601572582	51.0225775560042
6325	Taiwan (Province of China)	2004	52.96054001731	0.784677978	2.11251463998901	50.848025377321

TableS2 Frontier analysis of Smoking-Related Low Back Pain among WCBA

	location	year	val	SDI	frontier	eff_diff
6326	Taiwan (Province of China)	2005	52.61597399899	0.792445129	2.11167142439323	50.5043025745968
6327	Taiwan (Province of China)	2006	52.26838161083	0.800174675	2.11424287470444	50.1541387361256
6328	Taiwan (Province of China)	2007	51.75492160021	0.808137694	2.1145040507478	49.6404175494622
6329	Taiwan (Province of China)	2008	51.09197418822	0.815400691	2.11103915920652	48.9809350290135
6330	Taiwan (Province of China)	2009	50.2439299312	0.821432279	2.11451789344463	48.1294120377554
6331	Taiwan (Province of China)	2010	49.62738192844	0.827226752	2.11271841767747	47.5146635107625
6332	Taiwan (Province of China)	2011	48.96394545753	0.832064074	2.11248571787662	46.8514597396534
6333	Taiwan (Province of China)	2012	47.99174099585	0.836677665	2.11560157866815	45.8761394171818
6334	Taiwan (Province of China)	2013	46.87140862155	0.841280463	2.11287094889253	44.7585376726575
6335	Taiwan (Province of China)	2014	45.88792205742	0.84569556	2.11213147361063	43.7757905838094
6336	Taiwan (Province of China)	2015	45.05349631096	0.850055269	2.11538430306062	42.9381120078994
6337	Taiwan (Province of China)	2016	44.37619045532	0.854466038	2.11326648575776	42.2629239695622
6338	Taiwan (Province of China)	2017	43.88372368399	0.858888019	2.11022263378047	41.7735010502095
6339	Taiwan (Province of China)	2018	43.58670473234	0.863158361	2.11385405318864	41.4728506791514
6340	Taiwan (Province of China)	2019	43.26348324734	0.86741185	2.11636016698956	41.1471230803504
6341	Taiwan (Province of China)	2020	43.03787097692	0.871247947	2.11537625001	40.92249472691
6342	Taiwan (Province of China)	2021	42.76246373246	0.874747053	2.114580960342	40.647882772118
6343	Tajikistan	1990	17.77454477905	0.466155413	2.11063549668167	15.6639092823683
6344	Tajikistan	1991	17.61550254949	0.472342173	2.11699288984147	15.4985096596485
6345	Tajikistan	1992	17.63056822504	0.475745195	2.11449239586956	15.5160758291704
6346	Tajikistan	1993	17.73767719044	0.478238813	2.115572269606	15.622104920834
6347	Tajikistan	1994	18.00212843166	0.478726011	2.11261977952679	15.8895086521332
6348	Tajikistan	1995	18.26377851639	0.477586868	2.11384091366538	16.1499376027246
6349	Tajikistan	1996	18.50479591237	0.474346027	2.11368767164362	16.3911082407264
6350	Tajikistan	1997	18.61162752013	0.470274796	2.11310784382788	16.4985196763021

TableS2 Frontier analysis of Smoking-Related Low Back Pain among WCBA

	location	year	val	SDI	frontier	eff_diff
6351	Tajikistan	1998	18.66228656257	0.465341742	2.11424600720394	16.5480405553661
6352	Tajikistan	1999	18.78014987176	0.460505616	2.11165563140866	16.6684942403513
6353	Tajikistan	2000	18.88194991105	0.457061333	2.11332831718547	16.7686215938645
6354	Tajikistan	2001	18.95868451035	0.456256513	2.11234002196154	16.8463444883885
6355	Tajikistan	2002	19.00261635764	0.45880778	2.1142621674327	16.8883541902073
6356	Tajikistan	2003	19.04250674732	0.463329706	2.11474521058275	16.9277615367372
6357	Tajikistan	2004	19.02685067386	0.469154214	2.11378361988771	16.9130670539723
6358	Tajikistan	2005	18.97204826547	0.474637786	2.11249997001823	16.8595482954518
6359	Tajikistan	2006	18.86647522906	0.479651831	2.11293058591147	16.7535446431485
6360	Tajikistan	2007	18.71427080161	0.484211156	2.11292254600712	16.6013482556029
6361	Tajikistan	2008	18.51769523416	0.488441136	2.11241251183377	16.4052827223262
6362	Tajikistan	2009	18.25852579494	0.492064041	2.11746057848015	16.1410652164598
6363	Tajikistan	2010	17.99616950479	0.495893964	2.11678970515701	15.879379799633
6364	Tajikistan	2011	17.66356350242	0.499869804	2.11711895178773	15.5464445506323
6365	Tajikistan	2012	17.24953962731	0.503953885	2.1099831919663	15.1395564353437
6366	Tajikistan	2013	16.8289211542	0.507970676	2.10963226004954	14.7192888941505
6367	Tajikistan	2014	16.4517854841	0.511989518	2.11154594040923	14.3402395436908
6368	Tajikistan	2015	16.21251194208	0.515783245	2.11718995341481	14.0953219886652
6369	Tajikistan	2016	16.1676187694	0.519897002	2.11456569697889	14.0530530724211
6370	Tajikistan	2017	16.17750310289	0.524492607	2.11315069999434	14.0643524028957
6371	Tajikistan	2018	16.24638214873	0.529153916	2.11237614467213	14.1340060040579
6372	Tajikistan	2019	16.34483077199	0.533769951	2.11302016998237	14.2318106020076
6373	Tajikistan	2020	16.33355165819	0.537690531	2.11634902342762	14.2172026347624
6374	Tajikistan	2021	16.44043911647	0.541511187	2.1135510756819	14.3268880407881
6375	Tanzania	1990	39.94575696595	0.259306074	2.52045690449016	37.4253000614598

TableS2 Frontier analysis of Smoking-Related Low Back Pain among WCBA

	location	year	val	SDI	frontier	eff_diff
6376	Tanzania	1991	39.98695276481	0.262416238	2.47653706712964	37.5104156976804
6377	Tanzania	1992	40.00906534264	0.264989448	2.49155975967473	37.5175055829653
6378	Tanzania	1993	40.01764480435	0.267254915	2.47698557823133	37.5406592261187
6379	Tanzania	1994	39.96099469019	0.269417204	2.48793114866778	37.4730635415222
6380	Tanzania	1995	39.93949607937	0.272078202	2.47740712811532	37.4620889512547
6381	Tanzania	1996	39.84251499336	0.275507909	2.4558149803603	37.3867000129997
6382	Tanzania	1997	39.75251852092	0.279259184	2.45640624871389	37.2961122722061
6383	Tanzania	1998	39.68257622483	0.28315261	2.46015403884479	37.2224221859852
6384	Tanzania	1999	39.69980462443	0.287381679	2.43041014966513	37.2693944747649
6385	Tanzania	2000	39.77398497214	0.291827265	2.42579000409178	37.3481949680482
6386	Tanzania	2001	39.76982821774	0.296715645	2.40729771061833	37.3625305071217
6387	Tanzania	2002	39.70075457771	0.301983995	2.40230226303225	37.2984523146777
6388	Tanzania	2003	39.89951670525	0.307615488	2.38715647323292	37.5123602320171
6389	Tanzania	2004	40.07251234373	0.313772983	2.360340554825	37.712171788905
6390	Tanzania	2005	40.2741572584	0.320437339	2.34207357362024	37.9320836847798
6391	Tanzania	2006	40.24313538006	0.327234002	2.31748908886651	37.9256462911935
6392	Tanzania	2007	40.33567966405	0.334313237	2.28361223921592	38.0520674248341
6393	Tanzania	2008	40.3274393923	0.34135263	2.24504165034831	38.0823977419517
6394	Tanzania	2009	40.27343256897	0.348378035	2.12100273885603	38.152429830114
6395	Tanzania	2010	40.40095502399	0.355567563	2.11783582737085	38.2831191966191
6396	Tanzania	2011	40.59654763979	0.36303632	2.11545759052635	38.4810900492637
6397	Tanzania	2012	40.76520668547	0.370412488	2.11306978087887	38.6521369045911
6398	Tanzania	2013	40.96495092578	0.377963171	2.11424122470958	38.8507097010704
6399	Tanzania	2014	41.15581989692	0.385974579	2.11650526296041	39.0393146339596
6400	Tanzania	2015	41.35476796883	0.394277757	2.11069046753365	39.2440775012964

TableS2 Frontier analysis of Smoking-Related Low Back Pain among WCBA

	location	year	val	SDI	frontier	eff_diff
6401	Tanzania	2016	41.61982358305	0.402879308	2.11542334022232	39.5044002428277
6402	Tanzania	2017	41.98652071242	0.411824974	2.11146378122641	39.8750569311936
6403	Tanzania	2018	42.35229714848	0.420985364	2.11398981754222	40.2383073309378
6404	Tanzania	2019	42.5813144699	0.430225379	2.11223629539432	40.4690781745057
6405	Tanzania	2020	42.44402239996	0.438747238	2.11179928983794	40.3322231101221
6406	Tanzania	2021	42.35996790571	0.446568273	2.11533393770015	40.2446339680099
6407	Thailand	1990	19.02109380891	0.506644861	2.11710540551859	16.9039884033914
6408	Thailand	1991	19.16725461007	0.515911466	2.11569213735237	17.0515624727176
6409	Thailand	1992	19.31256403331	0.525161869	2.11684966214333	17.1957143711667
6410	Thailand	1993	19.4186632951	0.534303709	2.11361854866438	17.3050447464356
6411	Thailand	1994	19.547036016	0.543325547	2.11107072136556	17.4359652946344
6412	Thailand	1995	19.65021373027	0.552452209	2.11623082767589	17.5339829025941
6413	Thailand	1996	19.68295954989	0.561302487	2.11420770879962	17.5687518410904
6414	Thailand	1997	19.6909130532	0.568449492	2.11230879396565	17.5786042592343
6415	Thailand	1998	19.62227496119	0.573817902	2.11399909130986	17.5082758698801
6416	Thailand	1999	19.56079917376	0.578854168	2.1121756338601	17.4486235398999
6417	Thailand	2000	19.52947856397	0.583500917	2.11334020386405	17.416138360106
6418	Thailand	2001	19.31843206025	0.587661561	2.11462523360491	17.2038068266451
6419	Thailand	2002	19.02885905446	0.591759098	2.11407068632757	16.9147883681324
6420	Thailand	2003	18.64428777221	0.59604892	2.1132011899903	16.5310865822197
6421	Thailand	2004	18.2990386408	0.600520665	2.11385394134483	16.1851846994552
6422	Thailand	2005	18.01097048794	0.605040775	2.11256252976892	15.8984079581711
6423	Thailand	2006	17.73328716742	0.610356678	2.11442628078737	15.6188608866326
6424	Thailand	2007	17.50852503555	0.616368591	2.11357644859259	15.3949485869574
6425	Thailand	2008	17.14727323567	0.621067543	2.11140959040154	15.0358636452685

TableS2 Frontier analysis of Smoking-Related Low Back Pain among WCBA

	location	year	val	SDI	frontier	eff_diff
6426	Thailand	2009	16.72371534294	0.62432907	2.1128187457013	14.6108965972387
6427	Thailand	2010	16.54635602856	0.62858944	2.11522290988667	14.4311331186733
6428	Thailand	2011	16.75069142452	0.633226728	2.11511764790152	14.6355737766185
6429	Thailand	2012	17.09225346109	0.638410972	2.11473088139305	14.9775225796969
6430	Thailand	2013	17.44450395479	0.643672916	2.11361877483756	15.3308851799524
6431	Thailand	2014	17.69508814768	0.648772057	2.11226880935292	15.5828193383271
6432	Thailand	2015	17.72678165531	0.654015623	2.11533520403095	15.6114464512791
6433	Thailand	2016	17.54670639365	0.659369303	2.11414105104027	15.4325653426097
6434	Thailand	2017	17.33378444203	0.664804134	2.11322930522061	15.2205551368094
6435	Thailand	2018	17.1014930426	0.670225587	2.11157662606703	14.989916416533
6436	Thailand	2019	16.90476784942	0.675273901	2.11262954712215	14.7921383022978
6437	Thailand	2020	16.73476313079	0.679119989	2.11323589387216	14.6215272369178
6438	Thailand	2021	16.55757598974	0.682547933	2.11329881014092	14.4442771795991
6439	Timor-Leste	1990	17.2972032782	0.262468083	2.47927854688328	14.8179247313167
6440	Timor-Leste	1991	17.61782854441	0.270943349	2.49549968762027	15.1223288567897
6441	Timor-Leste	1992	17.86297931255	0.27991676	2.45809581343097	15.404883499119
6442	Timor-Leste	1993	18.05203301375	0.289266629	2.42233669880627	15.6296963149437
6443	Timor-Leste	1994	18.07654452832	0.298763189	2.40417378401891	15.6723707443011
6444	Timor-Leste	1995	17.95616780135	0.308018684	2.37956767966088	15.5766001216891
6445	Timor-Leste	1996	17.95410460094	0.31758875	2.34165941261836	15.6124451883216
6446	Timor-Leste	1997	17.87866880522	0.326705374	2.32645504168578	15.5522137635342
6447	Timor-Leste	1998	17.89494787524	0.334894497	2.2895937977565	15.6053540774835
6448	Timor-Leste	1999	17.98549481727	0.33925214	2.27308160686537	15.7124132104046
6449	Timor-Leste	2000	17.32528440339	0.343793649	2.17845710713217	15.1468272962578
6450	Timor-Leste	2001	17.10416861882	0.34949032	2.12596825629333	14.9782003625267

TableS2 Frontier analysis of Smoking-Related Low Back Pain among WCBA

	location	year	val	SDI	frontier	eff_diff
6451	Timor-Leste	2002	16.80274509188	0.35416202	2.12417532251444	14.6785697693656
6452	Timor-Leste	2003	16.49681466444	0.358440025	2.11874374323506	14.3780709212049
6453	Timor-Leste	2004	16.07978376887	0.362687998	2.11327513579159	13.9665086330784
6454	Timor-Leste	2005	15.63358621795	0.366913584	2.11294657859623	13.5206396393538
6455	Timor-Leste	2006	15.27568341447	0.370654092	2.11586780296149	13.1598156115085
6456	Timor-Leste	2007	14.90573376438	0.375390768	2.11828262760792	12.7874511367721
6457	Timor-Leste	2008	14.58300495031	0.381227528	2.11507831467191	12.4679266356381
6458	Timor-Leste	2009	14.24111999297	0.387944823	2.11166708807257	12.1294529048974
6459	Timor-Leste	2010	14.02773348692	0.394887019	2.11182178859475	11.9159116983253
6460	Timor-Leste	2011	13.90103601514	0.401872356	2.11350752289769	11.7875284922423
6461	Timor-Leste	2012	13.86550944543	0.4089103	2.11074979970612	11.7547596457239
6462	Timor-Leste	2013	13.86919692785	0.415217878	2.11247664062	11.75672028723
6463	Timor-Leste	2014	13.89877637858	0.421155538	2.11413444690203	11.784641931678
6464	Timor-Leste	2015	13.8759201302	0.42638362	2.11497662395908	11.7609435062409
6465	Timor-Leste	2016	13.85550945812	0.431207272	2.11476493219235	11.7407445259276
6466	Timor-Leste	2017	13.8543524664	0.434519734	2.11109237751378	11.7432600888862
6467	Timor-Leste	2018	13.84089066359	0.437018663	2.11510479475661	11.7257858688334
6468	Timor-Leste	2019	13.80959838585	0.440195845	2.11134656216143	11.6982518236886
6469	Timor-Leste	2020	13.72551310283	0.442349336	2.11457256472825	11.6109405381017
6470	Timor-Leste	2021	13.68138033137	0.444667619	2.11183755441948	11.5695427769505
6471	Togo	1990	9.08624998914	0.269692273	2.48397626392296	6.60227372521704
6472	Togo	1991	9.07854277381	0.274530568	2.45367809680984	6.62486467700016
6473	Togo	1992	9.03670957497	0.27878931	2.45432952048473	6.58238005448527
6474	Togo	1993	8.99963978626	0.281224631	2.44685623821013	6.55278354804987
6475	Togo	1994	8.93404287876	0.284653387	2.43039075977591	6.50365211898409

TableS2 Frontier analysis of Smoking-Related Low Back Pain among WCBA

	location	year	val	SDI	frontier	eff_diff
6476	Togo	1995	8.86138008751	0.288536692	2.4266122131225	6.4347678743875
6477	Togo	1996	8.79460219249	0.292606677	2.42838599209971	6.36621620039029
6478	Togo	1997	8.70836426586	0.296947835	2.406501429516	6.301862836344
6479	Togo	1998	8.67430496191	0.300022333	2.40677894498373	6.26752601692627
6480	Togo	1999	8.60315176474	0.303020232	2.39267637379116	6.21047539094884
6481	Togo	2000	8.57206096131	0.305472963	2.37296587472922	6.19909508658078
6482	Togo	2001	8.51758292551	0.307828258	2.38459975041523	6.13298317509477
6483	Togo	2002	8.48922997418	0.31046991	2.3607822572525	6.1284477169275
6484	Togo	2003	8.45175814504	0.313792064	2.36185490870425	6.08990323633575
6485	Togo	2004	8.39986669023	0.316687944	2.36156346738469	6.03830322284531
6486	Togo	2005	8.40178511798	0.31902558	2.33659244397333	6.06519267400667
6487	Togo	2006	8.34693916994	0.32165939	2.34309658375444	6.00384258618556
6488	Togo	2007	8.26837253886	0.324382497	2.32171537437258	5.94665716448742
6489	Togo	2008	8.1595901708	0.327743136	2.32320719342368	5.83638297737632
6490	Togo	2009	8.04554972562	0.331558542	2.3050021579325	5.7405475676875
6491	Togo	2010	7.93732044075	0.335899583	2.28882427316676	5.64849616758324
6492	Togo	2011	7.81738933314	0.34077726	2.24859248721729	5.56879684592271
6493	Togo	2012	7.63665222404	0.346320495	2.13623194512795	5.50042027891205
6494	Togo	2013	7.43920462619	0.352278605	2.12781196447386	5.31139266171614
6495	Togo	2014	7.26965582521	0.358661442	2.11537805460577	5.15427777060423
6496	Togo	2015	7.15086540859	0.365600169	2.11538001639328	5.03548539219672
6497	Togo	2016	7.08839728174	0.372910028	2.1123793668305	4.9760179149095
6498	Togo	2017	7.03518109561	0.380336231	2.113423253728	4.921757841882
6499	Togo	2018	6.98586762458	0.387921229	2.11404378214876	4.87182384243124
6500	Togo	2019	6.96067338941	0.395667269	2.11180708768384	4.84886630172617

TableS2 Frontier analysis of Smoking-Related Low Back Pain among WCBA

	location	year	val	SDI	frontier	eff_diff
6501	Togo	2020	6.9371377489	0.402356922	2.11484993770013	4.82228781119988
6502	Togo	2021	6.91207168856	0.408533695	2.11267031167253	4.79940137688747
6503	Tokelau	1990	68.92907704593	0.521942386	2.11250023618863	66.8165768097414
6504	Tokelau	1991	69.71031837133	0.526628473	2.1108158521029	67.5995025192271
6505	Tokelau	1992	70.34987130733	0.53130785	2.11827671732765	68.2315945900023
6506	Tokelau	1993	70.22856985394	0.535935005	2.11556996408435	68.1129998898557
6507	Tokelau	1994	69.81538087017	0.540523665	2.11180653416148	67.7035743360085
6508	Tokelau	1995	69.93995454805	0.545295634	2.11236760616446	67.8275869418855
6509	Tokelau	1996	70.52499418436	0.550282652	2.10998940046689	68.4150047838931
6510	Tokelau	1997	71.3468845514	0.555631326	2.11357121878061	69.2333133326194
6511	Tokelau	1998	72.20603273259	0.561047611	2.11594595845794	70.090086774132
6512	Tokelau	1999	72.8728684916	0.566415406	2.11555966477206	70.7573088268279
6513	Tokelau	2000	73.25427002105	0.57181437	2.1146202325667	71.1396497884833
6514	Tokelau	2001	72.88867022602	0.576915597	2.11385130073593	70.7748189252841
6515	Tokelau	2002	73.44667659147	0.581858317	2.11574857521414	71.3309280162559
6516	Tokelau	2003	76.008886825	0.586854346	2.11199905851921	73.8968877664808
6517	Tokelau	2004	79.12792184099	0.592342442	2.11331863021596	77.014603210774
6518	Tokelau	2005	80.93148223139	0.598176117	2.11289507277216	78.8185871586178
6519	Tokelau	2006	80.45040865938	0.60442939	2.11435722947986	78.3360514299001
6520	Tokelau	2007	77.92206663291	0.611163237	2.11308303228944	75.8089836006206
6521	Tokelau	2008	74.69167354118	0.617940247	2.11070940580735	72.5809641353726
6522	Tokelau	2009	71.59022575149	0.624070078	2.11436044953733	69.4758653019527
6523	Tokelau	2010	70.20280382436	0.63017554	2.11454068594815	68.0882631384118
6524	Tokelau	2011	71.73390380966	0.636016518	2.11182653064469	69.6220772790153
6525	Tokelau	2012	74.68062722583	0.641611991	2.11002363045585	72.5706035953742

TableS2 Frontier analysis of Smoking-Related Low Back Pain among WCBA

	location	year	val	SDI	frontier	eff_diff
6526	Tokelau	2013	77.42510171082	0.647966148	2.11034794689986	75.3147537639201
6527	Tokelau	2014	78.79333044587	0.653231999	2.11195113936387	76.6813793065061
6528	Tokelau	2015	78.57871412778	0.658798995	2.1121596306028	76.4665544971772
6529	Tokelau	2016	77.39591848815	0.663974319	2.11140697477543	75.2845115133746
6530	Tokelau	2017	76.32757572611	0.669054134	2.11206161226937	74.2155141138406
6531	Tokelau	2018	75.57806082806	0.674179514	2.11213247626607	73.4659283517939
6532	Tokelau	2019	75.83867464844	0.679247839	2.11728267407719	73.7213919743628
6533	Tokelau	2020	76.71845507141	0.682937378	2.11074812933208	74.6077069420779
6534	Tokelau	2021	78.5537634095	0.686425621	2.1155294479534	76.4382339615466
6535	Tonga	1990	51.14613974226	0.49180684	2.11604308058451	49.0300966616755
6536	Tonga	1991	51.34896422985	0.498166205	2.11036025645678	49.2386039733932
6537	Tonga	1992	51.56915265955	0.503659848	2.11462384647797	49.454528813072
6538	Tonga	1993	51.68302210921	0.50890445	2.11501978504953	49.5680023241605
6539	Tonga	1994	51.83988947762	0.514207835	2.11371712279753	49.7261723548225
6540	Tonga	1995	52.15270556889	0.52021502	2.11362889199212	50.0390766768979
6541	Tonga	1996	52.4276956949	0.525808135	2.10956066647462	50.3181350284254
6542	Tonga	1997	52.80541426143	0.531009092	2.11824356199654	50.6871706994335
6543	Tonga	1998	53.29671649571	0.536172314	2.1151939233488	51.1815225723612
6544	Tonga	1999	53.7518808227	0.54131702	2.11266571580117	51.6392151068988
6545	Tonga	2000	54.36400162674	0.546303195	2.11494912207352	52.2490525046665
6546	Tonga	2001	54.75075483812	0.551054251	2.1139627171164	52.6367921210036
6547	Tonga	2002	55.05947360481	0.556008345	2.11188179210857	52.9475918127014
6548	Tonga	2003	55.33729863835	0.560736066	2.11529563794203	53.222003000408
6549	Tonga	2004	55.52990404909	0.564806379	2.11464964653821	53.4152544025518
6550	Tonga	2005	55.70811645908	0.568449169	2.11608971699738	53.5920267420826

TableS2 Frontier analysis of Smoking-Related Low Back Pain among WCBA

	location	year	val	SDI	frontier	eff_diff
6551	Tonga	2006	55.71644623927	0.571619868	2.11424356004623	53.6022026792238
6552	Tonga	2007	55.54343742094	0.57435151	2.11478889596163	53.4286485249784
6553	Tonga	2008	55.22755261339	0.577305415	2.11127385947531	53.1162787539147
6554	Tonga	2009	54.9699152513	0.57917075	2.11315968479209	52.8567555665079
6555	Tonga	2010	54.93605252106	0.580657299	2.11388041016324	52.8221721108968
6556	Tonga	2011	55.03585155997	0.582656723	2.11681944069149	52.9190321192785
6557	Tonga	2012	55.16472665311	0.58477088	2.11117692458308	53.0535497285269
6558	Tonga	2013	55.182539294	0.587351141	2.11477610297146	53.0677631910285
6559	Tonga	2014	55.23527026282	0.590826853	2.1110648621803	53.1242054006397
6560	Tonga	2015	55.26609438646	0.594965748	2.11462395178059	53.1514704346794
6561	Tonga	2016	55.31139005879	0.600343669	2.11204408653643	53.1993459722536
6562	Tonga	2017	55.29495192998	0.606243592	2.11330101560313	53.1816509143769
6563	Tonga	2018	55.26511691198	0.611953942	2.11379050761209	53.1513264043679
6564	Tonga	2019	55.31692240192	0.617324063	2.11832611695025	53.1985962849697
6565	Tonga	2020	54.78612693953	0.622104456	2.11301282245155	52.6731141170784
6566	Tonga	2021	55.20832228164	0.626349936	2.11548140694262	53.0928408746974
6567	Trinidad and Tobago	1990	53.02963471407	0.62397015	2.11392556518337	50.9157091488866
6568	Trinidad and Tobago	1991	53.47996647684	0.628758918	2.11274549596701	51.367220980873
6569	Trinidad and Tobago	1992	53.84437295301	0.634039552	2.11130732115107	51.7330656318589
6570	Trinidad and Tobago	1993	54.15616507305	0.639191914	2.11293610924652	52.0432289638035
6571	Trinidad and Tobago	1994	54.33204395747	0.64424291	2.11087383667219	52.2211701207978
6572	Trinidad and Tobago	1995	54.40168762425	0.649220055	2.11323441139759	52.2884532128524
6573	Trinidad and Tobago	1996	54.2481027931	0.654169455	2.11130461673616	52.1367981763638
6574	Trinidad and Tobago	1997	54.00948686082	0.659293633	2.11237323718722	51.8971136236328
6575	Trinidad and Tobago	1998	53.51273430566	0.664507714	2.1135471505875	51.3991871550725

TableS2 Frontier analysis of Smoking-Related Low Back Pain among WCBA

	location	year	val	SDI	frontier	eff_diff
6576	Trinidad and Tobago	1999	52.9212283649	0.670244574	2.11636230525468	50.8048660596453
6577	Trinidad and Tobago	2000	52.35223033341	0.676683835	2.11100137282189	50.2412289605881
6578	Trinidad and Tobago	2001	51.83972330607	0.682763494	2.11323327954429	49.7264900265257
6579	Trinidad and Tobago	2002	51.3160854454	0.688872271	2.11192724603771	49.2041581993623
6580	Trinidad and Tobago	2003	50.91153341311	0.695828341	2.11001541757466	48.8015179955353
6581	Trinidad and Tobago	2004	50.57403035483	0.702610809	2.11530743933537	48.4587229154946
6582	Trinidad and Tobago	2005	50.35336153758	0.708520985	2.1133146168313	48.2400469207487
6583	Trinidad and Tobago	2006	50.16034203831	0.71460021	2.11085835219881	48.0494836861112
6584	Trinidad and Tobago	2007	49.96217203941	0.720196417	2.11330477214536	47.8488672672646
6585	Trinidad and Tobago	2008	49.86452984137	0.725720702	2.11238499455824	47.7521448468118
6586	Trinidad and Tobago	2009	50.00838289263	0.729538892	2.11257471976192	47.8958081728681
6587	Trinidad and Tobago	2010	50.23444044387	0.733237077	2.11503461140774	48.1194058324623
6588	Trinidad and Tobago	2011	50.66258270211	0.736802405	2.11428360784055	48.5482990942694
6589	Trinidad and Tobago	2012	51.21869904772	0.739999073	2.11280174124854	49.1058973064715
6590	Trinidad and Tobago	2013	51.82620034582	0.743593955	2.11593470992734	49.7102656358927
6591	Trinidad and Tobago	2014	52.68009046454	0.747338963	2.11191364808875	50.5681768164513
6592	Trinidad and Tobago	2015	53.2945809626	0.751510682	2.11179238843232	51.1827885741677
6593	Trinidad and Tobago	2016	54.04621237122	0.754953348	2.11409311883	51.93211925239
6594	Trinidad and Tobago	2017	54.64507117158	0.758226988	2.11499623972479	52.5300749318552
6595	Trinidad and Tobago	2018	55.21952342662	0.761241991	2.11139447488494	53.1081289517351
6596	Trinidad and Tobago	2019	55.60087355531	0.764169599	2.11055194310043	53.4903216122096
6597	Trinidad and Tobago	2020	56.0081103842	0.766422081	2.11442830292455	53.8936820812755
6598	Trinidad and Tobago	2021	55.94425002956	0.768763254	2.11320964030467	53.8310403892553
6599	Tropical Latin America	1990	193.95333714337	0.499587794	2.11613408038449	191.837203062986
6600	Tropical Latin America	1991	195.54788010464	0.504290478	2.11427564827914	193.433604456361

TableS2 Frontier analysis of Smoking-Related Low Back Pain among WCBA

	location	year	val	SDI	frontier	eff_diff
6601	Tropical Latin America	1992	196.59433885726	0.508056827	2.11345218895268	194.480886668307
6602	Tropical Latin America	1993	197.31312816553	0.511622219	2.114919644144	195.198208521386
6603	Tropical Latin America	1994	197.67819684016	0.515330113	2.11574507389983	195.56245176626
6604	Tropical Latin America	1995	197.5891263613	0.519290875	2.11429988899711	195.474826472303
6605	Tropical Latin America	1996	196.59603869291	0.523498539	2.11235604716688	194.483682645743
6606	Tropical Latin America	1997	194.10171180595	0.527841481	2.11600142288189	191.985710383068
6607	Tropical Latin America	1998	190.77736926885	0.532164921	2.11337976416909	188.663989504681
6608	Tropical Latin America	1999	187.20276813471	0.536658546	2.11397685445339	185.088791280257
6609	Tropical Latin America	2000	183.84358908816	0.541715646	2.11072622057815	181.732862867582
6610	Tropical Latin America	2001	179.6557895309	0.546931515	2.11111261994806	177.544676910952
6611	Tropical Latin America	2002	174.45712549873	0.552364618	2.11188170664554	172.345243792084
6612	Tropical Latin America	2003	168.70493796605	0.557691442	2.11293400359538	166.592003962455
6613	Tropical Latin America	2004	162.82695263137	0.563268657	2.11561684593444	160.711335785436
6614	Tropical Latin America	2005	157.37710658784	0.568897321	2.11445776884121	155.262648818999
6615	Tropical Latin America	2006	152.22877421938	0.574720306	2.1108914180831	150.117882801297
6616	Tropical Latin America	2007	146.82392215845	0.580915353	2.11140072752441	144.712521430926
6617	Tropical Latin America	2008	141.37360189615	0.587353638	2.11317185313333	139.260430043017
6618	Tropical Latin America	2009	136.01221132766	0.593303117	2.11374581807557	133.898465509584
6619	Tropical Latin America	2010	130.80525837287	0.599646005	2.11026586579512	128.694992507075
6620	Tropical Latin America	2011	125.26669240028	0.605956975	2.1122887428608	123.154403657419
6621	Tropical Latin America	2012	119.16847602932	0.61178731	2.11216821554273	117.056307813777
6622	Tropical Latin America	2013	113.24531905606	0.617401724	2.11194280311819	111.133376252942
6623	Tropical Latin America	2014	108.3286755648	0.622646118	2.11779474254013	106.21088082226
6624	Tropical Latin America	2015	105.13435955356	0.627399632	2.1137975391948	103.020562014365
6625	Tropical Latin America	2016	103.39940920007	0.631533364	2.11136875755364	101.288040442516

TableS2 Frontier analysis of Smoking-Related Low Back Pain among WCBA

	location	year	val	SDI	frontier	eff_diff
6626	Tropical Latin America	2017	102.18406527056	0.635765178	2.11553124538681	100.068534025173
6627	Tropical Latin America	2018	101.4728059344	0.640179901	2.11333139575924	99.3594745386408
6628	Tropical Latin America	2019	101.18564332446	0.644693634	2.11467187859721	99.0709714458628
6629	Tropical Latin America	2020	100.75022695121	0.648602851	2.11267012859813	98.6375568226119
6630	Tropical Latin America	2021	101.19671446075	0.652442394	2.11267198253667	99.0840424782133
6631	Tunisia	1990	22.47185368694	0.471138521	2.11253643726471	20.3593172496753
6632	Tunisia	1991	22.45558530875	0.479543057	2.11546584983782	20.3401194589122
6633	Tunisia	1992	22.50393164427	0.488285281	2.11358531011156	20.3903463341584
6634	Tunisia	1993	22.57239157274	0.496852039	2.10974725898828	20.4626443137517
6635	Tunisia	1994	22.7002324529	0.505564882	2.11374052265158	20.5864919302484
6636	Tunisia	1995	22.81848072128	0.51419694	2.1124830097893	20.7059977114907
6637	Tunisia	1996	22.88550442587	0.523294274	2.11292164788592	20.7725827779841
6638	Tunisia	1997	22.9574782865	0.532064044	2.11639107820681	20.8410872082932
6639	Tunisia	1998	23.00856167695	0.540498161	2.11317445166427	20.8953872252857
6640	Tunisia	1999	23.03974859847	0.548724563	2.11285343715259	20.9268951613174
6641	Tunisia	2000	23.0645261126	0.55667265	2.11205537058347	20.9524707420165
6642	Tunisia	2001	23.1199680683	0.56442101	2.11472859979836	21.0052394685016
6643	Tunisia	2002	23.15117858289	0.571719534	2.11798869806831	21.0331898848217
6644	Tunisia	2003	23.218245395	0.578882669	2.11160810160359	21.1066372933964
6645	Tunisia	2004	23.23985951679	0.586032594	2.11241785442623	21.1274416623638
6646	Tunisia	2005	23.28946910664	0.592937759	2.11138952658522	21.1780795800548
6647	Tunisia	2006	23.27572821708	0.599758513	2.11236924250086	21.1633589745791
6648	Tunisia	2007	23.29505113724	0.606696784	2.11390530279957	21.1811458344404
6649	Tunisia	2008	23.2564898701	0.613529322	2.11332373129266	21.1431661388073
6650	Tunisia	2009	23.30412923815	0.620106833	2.1148667884327	21.1892624497173

TableS2 Frontier analysis of Smoking-Related Low Back Pain among WCBA

	location	year	val	SDI	frontier	eff_diff
6651	Tunisia	2010	23.35682922737	0.626432774	2.11483044656603	21.241998780804
6652	Tunisia	2011	23.41307137989	0.63201984	2.11598039021343	21.2970909896766
6653	Tunisia	2012	23.41323494374	0.637578345	2.11291556990461	21.3003193738354
6654	Tunisia	2013	23.40177982576	0.643008512	2.11704260844903	21.284737217311
6655	Tunisia	2014	23.46570951583	0.648297395	2.11238096989484	21.3533285459352
6656	Tunisia	2015	23.60241578262	0.653413538	2.1140744515385	21.4883413310815
6657	Tunisia	2016	23.9635575008	0.658353517	2.11399577807754	21.8495617227225
6658	Tunisia	2017	24.46807755709	0.663222845	2.11552877782836	22.3525487792616
6659	Tunisia	2018	25.01822694123	0.668218997	2.11271532789862	22.9055116133314
6660	Tunisia	2019	25.50116728432	0.673170552	2.11070644451424	23.3904608398058
6661	Tunisia	2020	26.01508865709	0.677615148	2.11162782317049	23.9034608339195
6662	Tunisia	2021	26.14385211012	0.682432216	2.11536446195552	24.0284876481645
6663	Turkiye	1990	105.69913549931	0.461606984	2.11282934547426	103.586306153836
6664	Turkiye	1991	106.75857611377	0.469244066	2.11305092733841	104.645525186432
6665	Turkiye	1992	107.75992531516	0.476814569	2.11377988365718	105.646145431503
6666	Turkiye	1993	109.42433077132	0.48465697	2.11307402413619	107.311256747184
6667	Turkiye	1994	111.10747481222	0.49161995	2.11225848654017	108.99521632568
6668	Turkiye	1995	113.08055798182	0.49882484	2.1130982697284	110.967459712092
6669	Turkiye	1996	115.47392387747	0.506238546	2.11500128034403	113.358922597126
6670	Turkiye	1997	118.02241845824	0.513933356	2.11045494525868	115.911963512981
6671	Turkiye	1998	120.61169451048	0.521509898	2.11136368700517	118.500330823475
6672	Turkiye	1999	122.94393907305	0.528512416	2.11149744320164	120.832441629848
6673	Turkiye	2000	125.01597794523	0.535866331	2.11256353089083	122.903414414339
6674	Turkiye	2001	126.56832447162	0.54235913	2.11569932221175	124.452625149408
6675	Turkiye	2002	127.98479991844	0.549023644	2.11380870795234	125.870991210488

TableS2 Frontier analysis of Smoking-Related Low Back Pain among WCBA

	location	year	val	SDI	frontier	eff_diff
6676	Turkiye	2003	129.41541242723	0.556102527	2.113771248854	127.301641178376
6677	Turkiye	2004	130.63069398068	0.564024	2.11313599991258	128.517557980767
6678	Turkiye	2005	131.49333981914	0.572495886	2.11526182936782	129.378077989772
6679	Turkiye	2006	132.02066157409	0.581383179	2.1114151212103	129.90924645288
6680	Turkiye	2007	132.45888694074	0.590442387	2.11471307142386	130.344173869316
6681	Turkiye	2008	132.64204543517	0.599212061	2.11697647974032	130.52506895543
6682	Turkiye	2009	132.7260252617	0.607104618	2.11173849669541	130.614286765005
6683	Turkiye	2010	133.51505200316	0.615597228	2.11405527541619	131.400996727744
6684	Turkiye	2011	135.86579375485	0.624787211	2.11325213084589	133.752541624004
6685	Turkiye	2012	140.10774904205	0.634097092	2.10999741745692	137.997751624593
6686	Turkiye	2013	144.64912419523	0.643723823	2.11055907320444	142.538565122026
6687	Turkiye	2014	147.88313432414	0.653388835	2.11259763410726	145.770536690033
6688	Turkiye	2015	148.98550805209	0.662971739	2.11444254599776	146.871065506092
6689	Turkiye	2016	148.9746415939	0.671948148	2.11277954591286	146.861862047987
6690	Turkiye	2017	149.56825431104	0.681096272	2.11128321916341	147.456971091877
6691	Turkiye	2018	150.05221223807	0.689895867	2.11346727551316	147.938744962557
6692	Turkiye	2019	150.7883477595	0.698263774	2.11464412699128	148.673703632509
6693	Turkiye	2020	151.73250512875	0.705799744	2.11186535754357	149.620639771206
6694	Turkiye	2021	151.53118389156	0.712692673	2.11167876294958	149.41950512861
6695	Turkmenistan	1990	43.44848573533	0.563126887	2.11388506503151	41.3346006702985
6696	Turkmenistan	1991	43.47809492566	0.564939386	2.11715800009	41.36093692557
6697	Turkmenistan	1992	43.84624100589	0.565587505	2.11289006325896	41.733350942631
6698	Turkmenistan	1993	44.41242505973	0.567174449	2.11278309578986	42.2996419639401
6699	Turkmenistan	1994	45.16831244804	0.567288976	2.11229141856923	43.0560210294708
6700	Turkmenistan	1995	46.23223066819	0.567198635	2.11274780239671	44.1194828657933

TableS2 Frontier analysis of Smoking-Related Low Back Pain among WCBA

	location	year	val	SDI	frontier	eff_diff
6701	Turkmenistan	1996	47.83680753929	0.567061261	2.1144465295639	45.7223610097261
6702	Turkmenistan	1997	49.73807435967	0.565623084	2.11360989305912	47.6244644666109
6703	Turkmenistan	1998	51.77206685975	0.564405264	2.11254631597279	49.6595205437772
6704	Turkmenistan	1999	53.77662687315	0.564716006	2.11360650850732	51.6630203646427
6705	Turkmenistan	2000	55.42486249476	0.567421033	2.112868365877	53.311994128883
6706	Turkmenistan	2001	56.33349829142	0.571043819	2.11535662673368	54.2181416646863
6707	Turkmenistan	2002	57.30646431751	0.575596166	2.11449566529674	55.1919686522133
6708	Turkmenistan	2003	57.92581478528	0.580820126	2.11452788631385	55.8112868989662
6709	Turkmenistan	2004	58.54149377841	0.586712349	2.1167743346537	56.4247194437563
6710	Turkmenistan	2005	58.97997875593	0.593388895	2.11053214800037	56.8694466079296
6711	Turkmenistan	2006	59.19111985279	0.599896069	2.11226135700364	57.0788584957864
6712	Turkmenistan	2007	59.25328711742	0.606042198	2.11239924399942	57.1408878734206
6713	Turkmenistan	2008	59.01171919662	0.611923309	2.10964190958471	56.9020772870353
6714	Turkmenistan	2009	58.57754938608	0.617652905	2.11668305337797	56.460866332702
6715	Turkmenistan	2010	57.95205243604	0.623234589	2.1146755111739	55.8373769248661
6716	Turkmenistan	2011	56.8830266264	0.629233722	2.11261670744529	54.7704099189547
6717	Turkmenistan	2012	55.12570214627	0.635263183	2.11383769903906	53.0118644472309
6718	Turkmenistan	2013	53.21793982813	0.641361893	2.11344350131053	51.1044963268195
6719	Turkmenistan	2014	51.40131869982	0.647542417	2.11324083135237	49.2880778684676
6720	Turkmenistan	2015	49.88600854285	0.65335279	2.11515906761129	47.7708494752387
6721	Turkmenistan	2016	48.58090102908	0.658945613	2.11396733935733	46.4669336897227
6722	Turkmenistan	2017	47.37251958491	0.664284692	2.11640396800137	45.2561156169086
6723	Turkmenistan	2018	46.30289794096	0.669433599	2.11216527989941	44.1907326610606
6724	Turkmenistan	2019	45.68060430361	0.674303536	2.11442753158348	43.5661767720265
6725	Turkmenistan	2020	45.41430353912	0.67829818	2.11335855082679	43.3009449882932

TableS2 Frontier analysis of Smoking-Related Low Back Pain among WCBA

	location	year	val	SDI	frontier	eff_diff
6726	Turkmenistan	2021	45.06533000192	0.682160776	2.11530393648	42.95002606544
6727	Tuvalu	1990	88.29246215584	0.406247566	2.1142202483625	86.1782419074775
6728	Tuvalu	1991	89.44646199215	0.414819937	2.11319749460509	87.3332644975449
6729	Tuvalu	1992	90.51437697525	0.42290198	2.11420028708099	88.400176688169
6730	Tuvalu	1993	91.51165843096	0.43051826	2.11375431527342	89.3979041156866
6731	Tuvalu	1994	92.27031868875	0.438563051	2.11378310987151	90.1565355788785
6732	Tuvalu	1995	92.94203849987	0.444962242	2.1127731724471	90.8292653274229
6733	Tuvalu	1996	93.91399428888	0.44980498	2.11480084540169	91.7991934434783
6734	Tuvalu	1997	94.76947169542	0.455368428	2.11478252556526	92.6546891698547
6735	Tuvalu	1998	95.44380972685	0.462454678	2.11344225720159	93.3303674696484
6736	Tuvalu	1999	96.51049609647	0.468845802	2.11220875509239	94.3982873413776
6737	Tuvalu	2000	96.96568901137	0.474956823	2.11780414846486	94.8478848629051
6738	Tuvalu	2001	96.70972952977	0.480850886	2.11085691922753	94.5988726105425
6739	Tuvalu	2002	96.26533508304	0.487252252	2.11459481510478	94.1507402679352
6740	Tuvalu	2003	95.50016099936	0.492754711	2.11320184671202	93.386959152648
6741	Tuvalu	2004	95.14009744821	0.497692152	2.11241691533987	93.0276805328701
6742	Tuvalu	2005	94.30823923951	0.50185131	2.11798098572818	92.1902582537818
6743	Tuvalu	2006	93.21809997338	0.505975411	2.11549072514	91.10260924824
6744	Tuvalu	2007	92.47829940314	0.510504301	2.1158127226977	90.3624866804423
6745	Tuvalu	2008	91.31225580893	0.515611601	2.11561635535429	89.1966394535757
6746	Tuvalu	2009	90.33756870133	0.519870566	2.11768648415798	88.219882217172
6747	Tuvalu	2010	89.23218973432	0.52345551	2.11256583814125	87.1196238961788
6748	Tuvalu	2011	88.19473802579	0.527744245	2.11369843876418	86.0810395870258
6749	Tuvalu	2012	87.5731972289	0.531203235	2.11353025735697	85.459666971543
6750	Tuvalu	2013	87.00305646592	0.535166235	2.11206222434678	84.8909942415732

TableS2 Frontier analysis of Smoking-Related Low Back Pain among WCBA

	location	year	val	SDI	frontier	eff_diff
6751	Tuvalu	2014	86.76083362683	0.539122244	2.11360828976459	84.6472253370654
6752	Tuvalu	2015	85.97044369615	0.54404788	2.1120921240469	83.8583515721031
6753	Tuvalu	2016	85.28253223298	0.549173928	2.11389056905156	83.1686416639284
6754	Tuvalu	2017	84.67310082831	0.55446648	2.11211175668206	82.5609890716279
6755	Tuvalu	2018	83.87078948068	0.559859395	2.11363184773293	81.7571576329471
6756	Tuvalu	2019	83.2691701403	0.566045489	2.11222333092371	81.1569468093763
6757	Tuvalu	2020	82.42667296574	0.571666102	2.11316006503013	80.3135129007099
6758	Tuvalu	2021	82.81466245684	0.576620529	2.11444642002052	80.7002160368195
6759	UK	1990	287.33100910202	0.744334126	2.11304813466432	285.217960967356
6760	UK	1991	288.49986127169	0.749148231	2.11409465229403	286.385766619396
6761	UK	1992	290.14863044392	0.754556689	2.11365380438136	288.034976639539
6762	UK	1993	291.71877304782	0.76015435	2.11450528080537	289.604267767015
6763	UK	1994	293.23367638014	0.7651646	2.1131008511825	291.120575528957
6764	UK	1995	294.57317458612	0.768656864	2.11432539532725	292.458849190793
6765	UK	1996	296.03881068994	0.771576484	2.11345202539547	293.925358664544
6766	UK	1997	295.68025725443	0.775050122	2.1158202679732	293.564436986457
6767	UK	1998	295.06965094967	0.779057165	2.11153151652732	292.958119433143
6768	UK	1999	294.53981574201	0.784036475	2.11167925494185	292.428136487068
6769	UK	2000	293.72058586422	0.78921896	2.11389807066123	291.606687793559
6770	UK	2001	292.31686777375	0.793513165	2.11340117991657	290.203466593833
6771	UK	2002	288.74046679737	0.79665556	2.1127155724531	286.627751224917
6772	UK	2003	283.49953523573	0.799186111	2.11341151475094	281.386123720979
6773	UK	2004	277.55373799146	0.80195087	2.11388356338351	275.439854428077
6774	UK	2005	271.87220477702	0.804489194	2.11688823126128	269.755316545759
6775	UK	2006	266.13764940477	0.806817046	2.11246932318704	264.025180081583

TableS2 Frontier analysis of Smoking-Related Low Back Pain among WCBA

	location	year	val	SDI	frontier	eff_diff
6776	UK	2007	259.94621816147	0.809352772	2.11224610635986	257.83397205511
6777	UK	2008	253.73193677931	0.812039287	2.11376065701753	251.618176122292
6778	UK	2009	247.87178347767	0.814793642	2.11274913020241	245.759034347468
6779	UK	2010	242.15670611514	0.818033994	2.1141424441402	240.042563671
6780	UK	2011	235.16107925101	0.821718995	2.1155732666124	233.045505984398
6781	UK	2012	226.40810939013	0.826453754	2.11304350256702	224.295065887563
6782	UK	2013	217.27602126088	0.831508668	2.11216679589875	215.163854464981
6783	UK	2014	208.97029442022	0.835516642	2.11268944316627	206.857604977054
6784	UK	2015	202.70892609854	0.838951919	2.11201202088115	200.596914077659
6785	UK	2016	198.22478855935	0.842460233	2.11252323944923	196.112265319901
6786	UK	2017	194.54013824391	0.846263618	2.11315290367903	192.426985340231
6787	UK	2018	191.59587664296	0.849969788	2.11243451214678	189.483442130813
6788	UK	2019	189.22642396338	0.854130573	2.11432275777062	187.112101205609
6789	UK	2020	179.11750799472	0.85692029	2.11342160380825	177.004086390912
6790	UK	2021	179.4357118654	0.859000182	2.1135061360849	177.322205729315
6791	USA	1990	330.45468785349	0.76364769	2.11448610446531	328.340201749025
6792	USA	1991	327.18821487971	0.766044295	2.11250714427197	325.075707735438
6793	USA	1992	323.62248768254	0.769595492	2.11160593057931	321.510881751961
6794	USA	1993	319.35212708254	0.773103517	2.11512970684231	317.236997375698
6795	USA	1994	315.88772195846	0.776368588	2.11457902014719	313.773142938313
6796	USA	1995	313.01110771208	0.779410254	2.11198412693067	310.899123585149
6797	USA	1996	309.85566397766	0.78193477	2.11436642098767	307.741297556672
6798	USA	1997	304.30481288066	0.783979278	2.11409033820109	302.190722542459
6799	USA	1998	298.77740644691	0.786076332	2.11664685877745	296.660759588133
6800	USA	1999	294.34974086251	0.788544535	2.11215511070756	292.237585751802

TableS2 Frontier analysis of Smoking-Related Low Back Pain among WCBA

	location	year	val	SDI	frontier	eff_diff
6801	USA	2000	291.21710196245	0.79231665	2.11245021646104	289.104651745989
6802	USA	2001	289.28456889343	0.796676793	2.11278593157108	287.171782961859
6803	USA	2002	287.14756921452	0.800403318	2.11144352766296	285.036125686857
6804	USA	2003	284.41576554403	0.803228025	2.11389157358721	282.301873970443
6805	USA	2004	280.77989640112	0.805304692	2.11276411129819	278.667132289822
6806	USA	2005	276.00857597997	0.80603308	2.11490687622656	273.893669103743
6807	USA	2006	269.98348094023	0.806783583	2.1140750972396	267.86940584299
6808	USA	2007	263.20489203999	0.80968805	2.11559078780721	261.089301252183
6809	USA	2008	256.36385186445	0.814577102	2.11273645115851	254.251115413292
6810	USA	2009	250.21133585125	0.820294574	2.11290658695306	248.098429264297
6811	USA	2010	245.27945702699	0.825884658	2.11285444097032	243.16660258602
6812	USA	2011	240.75538600977	0.830266388	2.11274541264261	238.642640597127
6813	USA	2012	235.89144539659	0.833958192	2.11768275551629	233.773762641074
6814	USA	2013	231.05350586261	0.837412988	2.11466984532585	228.938836017284
6815	USA	2014	226.51094744314	0.840746528	2.11325789224455	224.397689550895
6816	USA	2015	223.04043734589	0.844370336	2.11513962147042	220.92529772442
6817	USA	2016	220.62373503843	0.848015521	2.11238259240548	218.511352446025
6818	USA	2017	218.40122397188	0.851630299	2.11404985948571	216.287174112394
6819	USA	2018	216.61396879395	0.855049193	2.1129994920045	214.500969301946
6820	USA	2019	215.39295561518	0.858578065	2.11253645759227	213.280419157588
6821	USA	2020	214.4923678186	0.860792773	2.11226627888443	212.380101539716
6822	USA	2021	213.53740747663	0.862448354	2.11269866942333	211.424708807207
6823	Uganda	1990	10.01867209278	0.187001096	3.55113718249797	6.46753491028203
6824	Uganda	1991	10.16847421277	0.188924156	3.45953603291667	6.70893817985333
6825	Uganda	1992	10.3437548816	0.191003433	3.50710994918667	6.83664493241333

TableS2 Frontier analysis of Smoking-Related Low Back Pain among WCBA

	location	year	val	SDI	frontier	eff_diff
6826	Uganda	1993	10.49455307976	0.193557456	3.50152906174361	6.99302401801638
6827	Uganda	1994	10.62466315208	0.197293193	3.48943016031318	7.13523299176682
6828	Uganda	1995	10.68059171596	0.201835423	3.47414704334743	7.20644467261257
6829	Uganda	1996	10.80652440098	0.207080731	3.40791827262159	7.3986061283584
6830	Uganda	1997	10.92779150178	0.213091696	3.41283391435797	7.51495758742203
6831	Uganda	1998	11.02543135473	0.219712442	2.91364294128776	8.11178841344224
6832	Uganda	1999	11.11533674909	0.226916945	2.79670869928406	8.31862804980594
6833	Uganda	2000	11.26337732041	0.234323225	2.63901205048583	8.62436526992417
6834	Uganda	2001	11.18062748434	0.242186388	2.5374195188	8.64320796554
6835	Uganda	2002	11.07374811865	0.250552388	2.54443042185152	8.52931769679848
6836	Uganda	2003	10.98960236093	0.259164883	2.55075371714246	8.43884864378754
6837	Uganda	2004	10.91932364052	0.268004611	2.47466477473769	8.44465886578231
6838	Uganda	2005	10.8825226892	0.277369193	2.4540779243327	8.4284447648673
6839	Uganda	2006	10.83898190784	0.287030583	2.43254408591395	8.40643782192605
6840	Uganda	2007	10.76981276219	0.29674042	2.40097198836344	8.36884077382656
6841	Uganda	2008	10.70971140968	0.306748843	2.39021964949544	8.31949176018456
6842	Uganda	2009	10.66786879818	0.316731153	2.36385476555303	8.30401403262697
6843	Uganda	2010	10.65102963735	0.326543661	2.31667336999441	8.33435626735559
6844	Uganda	2011	10.675895157	0.336217716	2.28817116735448	8.38772398964552
6845	Uganda	2012	10.70558971738	0.345364515	2.14766088751123	8.55792882986877
6846	Uganda	2013	10.74709398046	0.354381615	2.11271252860971	8.63438145185029
6847	Uganda	2014	10.79972315193	0.363430903	2.11362143727047	8.68610171465953
6848	Uganda	2015	10.83807590183	0.372537986	2.11419861578373	8.72387728604627
6849	Uganda	2016	10.84004996289	0.381312226	2.11310967941676	8.72694028347324
6850	Uganda	2017	10.84968091488	0.390082259	2.11063898241667	8.73904193246333

TableS2 Frontier analysis of Smoking-Related Low Back Pain among WCBA

	location	year	val	SDI	frontier	eff_diff
6851	Uganda	2018	10.83135048514	0.399117777	2.112961243395	8.718389241745
6852	Uganda	2019	10.83803920279	0.408431557	2.11211085774808	8.72592834504192
6853	Uganda	2020	10.81763467539	0.416846079	2.11540675573662	8.70222791965338
6854	Uganda	2021	10.74901983074	0.423261181	2.112829987045	8.636189843695
6855	Ukraine	1990	186.69232540157	0.647461466	2.11181496959422	184.580510431976
6856	Ukraine	1991	183.72455615436	0.651950021	2.11431974836212	181.610236405998
6857	Ukraine	1992	181.9853544027	0.657600333	2.11219972798	179.87315467472
6858	Ukraine	1993	181.09868645629	0.662867405	2.11219783281174	178.986488623478
6859	Ukraine	1994	180.72510373949	0.665506375	2.11318693478072	178.611916804709
6860	Ukraine	1995	181.18813074294	0.666950167	2.11489957124014	179.0732311717
6861	Ukraine	1996	181.86407574236	0.668104744	2.11171479746788	179.752360944892
6862	Ukraine	1997	182.88765018681	0.669207846	2.11068381486479	180.776966371945
6863	Ukraine	1998	183.86121540517	0.670322301	2.11279050313571	181.748424902034
6864	Ukraine	1999	184.61309047823	0.670642905	2.11194623675869	182.501144241471
6865	Ukraine	2000	185.51559696281	0.671095001	2.11268603741514	183.402910925395
6866	Ukraine	2001	187.6925815481	0.67246782	2.11627105014657	185.576310497953
6867	Ukraine	2002	192.08888066045	0.674543221	2.112836762095	189.976043898355
6868	Ukraine	2003	197.6115571177	0.678614636	2.11158607687027	195.49997104083
6869	Ukraine	2004	203.26212619394	0.685380936	2.11237739690123	201.149748797039
6870	Ukraine	2005	208.30483974184	0.692023301	2.11252580337456	206.192313938465
6871	Ukraine	2006	212.94145378173	0.698093383	2.11376503043263	210.827688751297
6872	Ukraine	2007	217.99834326222	0.704202308	2.11341287887375	215.884930383346
6873	Ukraine	2008	222.64214381122	0.710980184	2.11368194285884	220.528461868361
6874	Ukraine	2009	226.50104780184	0.716185465	2.11454484983846	224.386502952002
6875	Ukraine	2010	228.89856539581	0.72101987	2.11278890703198	226.785776488778

TableS2 Frontier analysis of Smoking-Related Low Back Pain among WCBA

	location	year	val	SDI	frontier	eff_diff
6876	Ukraine	2011	230.16799935366	0.724891957	2.11361509453851	228.054384259121
6877	Ukraine	2012	231.66878675312	0.7288172	2.11248409650524	229.556302656615
6878	Ukraine	2013	233.50588278672	0.734033573	2.11517664891059	231.390706137809
6879	Ukraine	2014	235.54375539858	0.739883557	2.11329882046828	233.430456578112
6880	Ukraine	2015	237.65626828699	0.743790863	2.1144616217407	235.541806665249
6881	Ukraine	2016	237.39627513142	0.746857989	2.11861898803784	235.277656143382
6882	Ukraine	2017	233.53840582397	0.750593324	2.11341699377346	231.424988830197
6883	Ukraine	2018	229.13585764522	0.754451727	2.11179541657302	227.024062228647
6884	Ukraine	2019	226.10705355516	0.757980081	2.111675968756	223.995377586404
6885	Ukraine	2020	224.87348211378	0.759929872	2.11337892200602	222.760103191774
6886	Ukraine	2021	223.14427661069	0.760773913	2.11236820150488	221.031908409185
6887	United Arab Emirates	1990	32.61244125086	0.644412271	2.11252675420767	30.4999144966523
6888	United Arab Emirates	1991	33.17640235616	0.660739899	2.11160483699456	31.0647975191654
6889	United Arab Emirates	1992	33.68521801956	0.675926211	2.11804689736394	31.5671711221961
6890	United Arab Emirates	1993	33.98236248755	0.689463801	2.11509630211449	31.8672661854355
6891	United Arab Emirates	1994	34.17898428784	0.701656792	2.11390237544919	32.0650819123908
6892	United Arab Emirates	1995	34.16687655692	0.713138288	2.11240544991662	32.0544711070034
6893	United Arab Emirates	1996	33.93220690547	0.724181976	2.11359531696292	31.8186115885071
6894	United Arab Emirates	1997	33.9062098701	0.734723175	2.11197758365269	31.7942322864473
6895	United Arab Emirates	1998	34.14070206043	0.744476623	2.11154275598919	32.0291593044408
6896	United Arab Emirates	1999	34.65499859967	0.753253916	2.11336980278028	32.5416287968897
6897	United Arab Emirates	2000	35.19520221349	0.760969094	2.11291770581642	33.0822845076736
6898	United Arab Emirates	2001	35.82202565283	0.767858793	2.11141395768973	33.7106116951403
6899	United Arab Emirates	2002	36.64602220085	0.774346424	2.11220308470159	34.5338191161484
6900	United Arab Emirates	2003	37.4686723935	0.781143992	2.11324079194735	35.3554316015527

TableS2 Frontier analysis of Smoking-Related Low Back Pain among WCBA

	location	year	val	SDI	frontier	eff_diff
6901	United Arab Emirates	2004	38.29393695456	0.788525951	2.11269370945773	36.1812432451023
6902	United Arab Emirates	2005	39.10869722868	0.796428086	2.1169347795975	36.9917624490825
6903	United Arab Emirates	2006	38.4189224891	0.805537023	2.11474734564113	36.3041751434589
6904	United Arab Emirates	2007	36.51599500222	0.814870878	2.11445791178338	34.4015370904366
6905	United Arab Emirates	2008	34.88264186636	0.822793435	2.11118868084655	32.7714531855135
6906	United Arab Emirates	2009	35.17886052132	0.828304165	2.11241222434797	33.066448296972
6907	United Arab Emirates	2010	37.98974036419	0.831284254	2.11332968848961	35.8764106757004
6908	United Arab Emirates	2011	41.07092868699	0.832912804	2.11110875652325	38.9598199304668
6909	United Arab Emirates	2012	43.97181380779	0.83358611	2.11320477657203	41.858609031218
6910	United Arab Emirates	2013	46.43122877701	0.833803818	2.11284354172903	44.318385235281
6911	United Arab Emirates	2014	48.44439812158	0.833858727	2.11238426235284	46.3320138592272
6912	United Arab Emirates	2015	50.24660049793	0.834175498	2.1148759594769	48.1317245384531
6913	United Arab Emirates	2016	51.80285732291	0.83522071	2.11265508034	49.69020224257
6914	United Arab Emirates	2017	53.05968313048	0.837213553	2.11262473693368	50.9470583935463
6915	United Arab Emirates	2018	53.88731863183	0.83990014	2.11590090478861	51.7714177270414
6916	United Arab Emirates	2019	54.34032562438	0.842985326	2.1136098287539	52.2267157956261
6917	United Arab Emirates	2020	54.37866454444	0.846151976	2.1126106907503	52.2660538536897
6918	United Arab Emirates	2021	53.91632322822	0.849317734	2.11596857990638	51.8003546483136
6919	Uruguay	1990	255.35119875795	0.581921855	2.11497991820676	253.236218839743
6920	Uruguay	1991	256.37511749228	0.586156118	2.11331250795985	254.26180498432
6921	Uruguay	1992	256.94818714429	0.589822355	2.11179352280825	254.836393621482
6922	Uruguay	1993	258.37875039012	0.592295025	2.1159408960465	256.262809494074
6923	Uruguay	1994	260.06386651053	0.595188424	2.11307391830377	257.950792592226
6924	Uruguay	1995	262.51395700273	0.596825409	2.11222850883161	260.401728493898
6925	Uruguay	1996	265.86508801262	0.599775104	2.11404140693795	263.751046605682

TableS2 Frontier analysis of Smoking-Related Low Back Pain among WCBA

	location	year	val	SDI	frontier	eff_diff
6926	Uruguay	1997	269.09449973313	0.605665051	2.11481019533053	266.979689537799
6927	Uruguay	1998	272.67265019112	0.612700815	2.11498955957586	270.557660631544
6928	Uruguay	1999	275.31689528434	0.619009271	2.11499628035602	273.201899003984
6929	Uruguay	2000	277.11945202292	0.623208291	2.11373678803279	275.005715234887
6930	Uruguay	2001	279.29614994243	0.625796068	2.11701914556919	277.179130796861
6931	Uruguay	2002	282.00678928432	0.6282858	2.11517270738105	279.891616576939
6932	Uruguay	2003	284.76368372813	0.631437774	2.11584686135551	282.647836866774
6933	Uruguay	2004	286.08816933432	0.634216906	2.11151127068785	283.976658063632
6934	Uruguay	2005	285.87411061153	0.63697577	2.11216465470429	283.761945956826
6935	Uruguay	2006	283.32937155925	0.640590696	2.11184367436064	281.217527884889
6936	Uruguay	2007	278.47942436103	0.646331714	2.11115574282642	276.368268618204
6937	Uruguay	2008	272.85672378736	0.653525522	2.114475354831	270.742248432529
6938	Uruguay	2009	267.00376226165	0.660321135	2.11407160419258	264.889690657457
6939	Uruguay	2010	261.96360490333	0.666099135	2.11310094847679	259.850503954853
6940	Uruguay	2011	256.68168668354	0.667079733	2.11169211883864	254.569994564701
6941	Uruguay	2012	251.18308741755	0.665802192	2.11118346107235	249.071903956478
6942	Uruguay	2013	245.79029258058	0.668228361	2.1123735626574	243.677919017923
6943	Uruguay	2014	241.46229419136	0.67333426	2.11351358620088	239.348780605159
6944	Uruguay	2015	238.82539249697	0.680595506	2.11483287831032	236.71055961866
6945	Uruguay	2016	240.37970579298	0.689816733	2.11373143818098	238.265974354799
6946	Uruguay	2017	245.59780437243	0.699189456	2.11454354636403	243.483260826066
6947	Uruguay	2018	251.73442576513	0.70690493	2.11256193419479	249.621863830935
6948	Uruguay	2019	254.34988810475	0.712536588	2.11519866944609	252.234689435304
6949	Uruguay	2020	244.65731552585	0.716169766	2.11363407371453	242.543681452135
6950	Uruguay	2021	259.73703791588	0.719283445	2.11155018027164	257.625487735608

TableS2 Frontier analysis of Smoking-Related Low Back Pain among WCBA

	location	year	val	SDI	frontier	eff_diff
6951	Uzbekistan	1990	13.84039881973	0.500241735	2.11495051300444	11.7254483067256
6952	Uzbekistan	1991	14.13848102493	0.501544609	2.11041199175574	12.0280690331743
6953	Uzbekistan	1992	14.55148380956	0.502905194	2.11556559270135	12.4359182168586
6954	Uzbekistan	1993	15.03188011045	0.506556615	2.11124299457741	12.9206371158726
6955	Uzbekistan	1994	15.51303677726	0.511018525	2.11206017897589	13.4009765982841
6956	Uzbekistan	1995	15.98609423906	0.516986264	2.1143699394838	13.8717242995762
6957	Uzbekistan	1996	16.79617939675	0.52439291	2.11291555992842	14.6832638368216
6958	Uzbekistan	1997	17.7554988593	0.532686328	2.1146411591139	15.6408577001861
6959	Uzbekistan	1998	18.76011820261	0.541279069	2.11418231426824	16.6459358883418
6960	Uzbekistan	1999	19.86177710261	0.549739391	2.11361701003831	17.7481600925717
6961	Uzbekistan	2000	20.86266933794	0.557522311	2.11255974354622	18.7501095943938
6962	Uzbekistan	2001	21.40826406631	0.564803383	2.11534526816792	19.2929187981421
6963	Uzbekistan	2002	22.00938167567	0.571690143	2.11406439095398	19.895317284716
6964	Uzbekistan	2003	22.54520157608	0.578006538	2.11460456363432	20.4305970124457
6965	Uzbekistan	2004	23.05143542569	0.583942986	2.11419708898732	20.9372383367027
6966	Uzbekistan	2005	23.43568980957	0.589530777	2.11207252688753	21.3236172826825
6967	Uzbekistan	2006	23.79753824281	0.594498057	2.11839830187327	21.6791399409367
6968	Uzbekistan	2007	24.12671324443	0.599628055	2.1099728677353	22.0167403766947
6969	Uzbekistan	2008	24.44580801387	0.604889158	2.11607514482859	22.3297328690414
6970	Uzbekistan	2009	24.72625055151	0.610351745	2.11510989734154	22.6111406541685
6971	Uzbekistan	2010	24.83928285389	0.616097907	2.11502544329067	22.7242574105993
6972	Uzbekistan	2011	24.83489103521	0.621344619	2.11681812829392	22.7180729069161
6973	Uzbekistan	2012	24.66430645841	0.626202125	2.11408528059534	22.5502211778147
6974	Uzbekistan	2013	24.42329264325	0.630796403	2.11123026731596	22.312062375934
6975	Uzbekistan	2014	24.26756568483	0.63525705	2.11380786850567	22.1537578163243

TableS2 Frontier analysis of Smoking-Related Low Back Pain among WCBA

	location	year	val	SDI	frontier	eff_diff
6976	Uzbekistan	2015	24.26109040961	0.639805639	2.11303915473922	22.1480512548708
6977	Uzbekistan	2016	24.41432071196	0.644230597	2.11105527570276	22.3032654362572
6978	Uzbekistan	2017	24.58240622891	0.648119347	2.11073494828377	22.4716712806262
6979	Uzbekistan	2018	24.82050247119	0.651918144	2.11156811957312	22.7089343516169
6980	Uzbekistan	2019	25.09737681684	0.655836959	2.11543321054853	22.9819436062915
6981	Uzbekistan	2020	25.18393067065	0.659124204	2.11209754920131	23.0718331214487
6982	Uzbekistan	2021	25.3725564136	0.662621694	2.11306534140159	23.2594910721984
6983	Vanuatu	1990	16.28948364989	0.353100252	2.11743477714312	14.1720488727469
6984	Vanuatu	1991	16.60813307785	0.357005379	2.115621802084	14.492511275766
6985	Vanuatu	1992	16.86097588525	0.360893296	2.11135304420662	14.7496228410434
6986	Vanuatu	1993	17.10763923857	0.364444155	2.1135254374246	14.9941138011454
6987	Vanuatu	1994	17.29437590084	0.368501002	2.11417792767325	15.1801979731667
6988	Vanuatu	1995	17.41579854065	0.372479126	2.11788291872765	15.2979156219224
6989	Vanuatu	1996	17.59708024465	0.376460885	2.11185489444236	15.4852253502076
6990	Vanuatu	1997	17.93302335097	0.380667171	2.11266592991386	15.8203574210561
6991	Vanuatu	1998	18.18583618037	0.384690196	2.11166743007	16.0741687503
6992	Vanuatu	1999	18.45270354936	0.388300526	2.11201313647233	16.3406904128877
6993	Vanuatu	2000	18.68507180404	0.392158349	2.11359703064849	16.5714747733915
6994	Vanuatu	2001	18.808134369	0.395325055	2.10982871895088	16.6983056500491
6995	Vanuatu	2002	18.7486461926	0.397723782	2.11317668077521	16.6354695118248
6996	Vanuatu	2003	18.65462558478	0.400396456	2.11221160828943	16.5424139764906
6997	Vanuatu	2004	18.38303974474	0.403226097	2.11349925349974	16.2695404912403
6998	Vanuatu	2005	18.07254363842	0.406488572	2.11489880016775	15.9576448382523
6999	Vanuatu	2006	17.68860935306	0.410501729	2.11240892202119	15.5762004310388
7000	Vanuatu	2007	17.17615297393	0.414915578	2.11317926801235	15.0629737059176

TableS2 Frontier analysis of Smoking-Related Low Back Pain among WCBA

	location	year	val	SDI	frontier	eff_diff
7001	Vanuatu	2008	16.63300107466	0.419853237	2.11284861570071	14.5201524589593
7002	Vanuatu	2009	16.09393123361	0.42485465	2.11572026436275	13.9782109692472
7003	Vanuatu	2010	15.62052393206	0.429658049	2.11355879162547	13.5069651404345
7004	Vanuatu	2011	15.18237977204	0.434283044	2.11831339875788	13.0640663732821
7005	Vanuatu	2012	14.82247116285	0.438661998	2.11521193999819	12.7072592228518
7006	Vanuatu	2013	14.52991859658	0.442720176	2.1109695212345	12.4189490753455
7007	Vanuatu	2014	14.34744441737	0.446687298	2.11902695195197	12.228417465418
7008	Vanuatu	2015	14.2135151548	0.450329861	2.10999025362178	12.1035249011782
7009	Vanuatu	2016	14.15442654835	0.454072763	2.11409535971194	12.0403311886381
7010	Vanuatu	2017	14.10477052747	0.458016834	2.11272587010333	11.9920446573667
7011	Vanuatu	2018	14.0982042075	0.462080221	2.11355573373338	11.9846484737666
7012	Vanuatu	2019	14.09542247456	0.466276162	2.11503246934556	11.9803900052144
7013	Vanuatu	2020	14.10892297339	0.470038391	2.11362893486918	11.9952940385208
7014	Vanuatu	2021	14.16958146742	0.473100706	2.11318005389658	12.0564014135234
7015	Venezuela	1990	90.7281290327	0.516891218	2.11401775092385	88.6141112817761
7016	Venezuela	1991	90.50390657373	0.522272296	2.11205450421147	88.3918520695185
7017	Venezuela	1992	90.10060509481	0.529517991	2.11414263290328	87.9864624619067
7018	Venezuela	1993	89.61120641642	0.536279978	2.11353747118869	87.4976689452313
7019	Venezuela	1994	89.28592725551	0.541866058	2.11202549422534	87.1739017612847
7020	Venezuela	1995	88.91081820899	0.546348234	2.1124178896416	86.7984003193484
7021	Venezuela	1996	88.65213683317	0.548649226	2.1147716919791	86.5373651411909
7022	Venezuela	1997	87.8641355451	0.552364437	2.11535122548693	85.7487843196131
7023	Venezuela	1998	86.990775237	0.557243117	2.11299365500722	84.8777815819928
7024	Venezuela	1999	86.34629173747	0.560277999	2.11549774464	84.23079399283
7025	Venezuela	2000	85.82493289157	0.56350626	2.11162332811776	83.7133095634522

TableS2 Frontier analysis of Smoking-Related Low Back Pain among WCBA

	location	year	val	SDI	frontier	eff_diff
7026	Venezuela	2001	85.44364267008	0.565458381	2.1142504438031	83.3293922262769
7027	Venezuela	2002	84.66679282571	0.562604461	2.11180350698767	82.5549893187223
7028	Venezuela	2003	83.96933729599	0.555212224	2.11392685751211	81.8554104384779
7029	Venezuela	2004	82.935921638	0.550972256	2.11180212896493	80.8241195090351
7030	Venezuela	2005	81.70791308848	0.553941679	2.11236511771642	79.5955479707636
7031	Venezuela	2006	80.34352888685	0.56333419	2.1129661229603	78.2305627638897
7032	Venezuela	2007	79.0626820605	0.575367174	2.11339786052948	76.9492841999705
7033	Venezuela	2008	77.37708756512	0.586184887	2.11085274463515	75.2662348204848
7034	Venezuela	2009	75.9276980409	0.593288422	2.11526783515489	73.8124302057451
7035	Venezuela	2010	74.50861786513	0.599127923	2.11605296692902	72.392564898201
7036	Venezuela	2011	73.314059614	0.604823484	2.11356547836568	71.2004941356343
7037	Venezuela	2012	72.10039365117	0.610187465	2.11180923935757	69.9885844118124
7038	Venezuela	2013	70.76795041251	0.614441024	2.11250992440577	68.6554404881042
7039	Venezuela	2014	69.79969597421	0.614686806	2.11130024153383	67.6883957326762
7040	Venezuela	2015	69.27380869767	0.610675357	2.113092824095	67.160715873575
7041	Venezuela	2016	68.89017798247	0.606993213	2.11197561630051	66.7782023661695
7042	Venezuela	2017	68.82966906296	0.605160131	2.11308046497935	66.7165885979806
7043	Venezuela	2018	70.09068884252	0.604068247	2.11189805046276	67.9787907920572
7044	Venezuela	2019	72.65698171873	0.602313887	2.11388288726756	70.5430988314624
7045	Venezuela	2020	73.69845765168	0.600034781	2.11184400681158	71.5866136448684
7046	Venezuela	2021	74.32404704426	0.596513059	2.11486323671642	72.2091838075436
7047	Vietnam	1990	9.61376424022	0.407630048	2.11416720657092	7.49959703364908
7048	Vietnam	1991	9.9898395134	0.41321725	2.11320770385766	7.87663180954234
7049	Vietnam	1992	10.32780135567	0.419781657	2.11216152807203	8.21563982759797
7050	Vietnam	1993	10.72563200935	0.427072185	2.11262383257721	8.61300817677279

TableS2 Frontier analysis of Smoking-Related Low Back Pain among WCBA

	location	year	val	SDI	frontier	eff_diff
7051	Vietnam	1994	11.089446991	0.435106272	2.11303485530373	8.97641213569627
7052	Vietnam	1995	11.37101356614	0.443858785	2.11117240249738	9.25984116364262
7053	Vietnam	1996	11.79305599068	0.45310241	2.1131557672582	9.6799002234218
7054	Vietnam	1997	12.16930552876	0.462853518	2.11432310853797	10.054982420222
7055	Vietnam	1998	12.53037275007	0.472478892	2.11145732722237	10.4189154228476
7056	Vietnam	1999	12.85819494608	0.481445774	2.11749101010536	10.7407039359746
7057	Vietnam	2000	13.2821671388	0.489802045	2.11374778911616	11.1684193496838
7058	Vietnam	2001	13.38905596695	0.497630349	2.1145407621408	11.2745152048092
7059	Vietnam	2002	13.49293765705	0.505453447	2.11018498528045	11.3827526717695
7060	Vietnam	2003	13.55295998575	0.513367511	2.1130177077022	11.4399422780478
7061	Vietnam	2004	13.58587811933	0.52122939	2.11126737084847	11.4746107484815
7062	Vietnam	2005	13.56097199086	0.528956332	2.11394150736761	11.4470304834924
7063	Vietnam	2006	13.49756868598	0.536300653	2.11365429100946	11.3839143949705
7064	Vietnam	2007	13.42307056092	0.543342652	2.11451732276644	11.3085532381536
7065	Vietnam	2008	13.34674741576	0.550127119	2.11065813459101	11.236089281169
7066	Vietnam	2009	13.26170893021	0.556455944	2.11434390375049	11.1473650264595
7067	Vietnam	2010	13.16190547452	0.562768571	2.11718653408797	11.044718940432
7068	Vietnam	2011	13.03314147072	0.568945458	2.11567087659548	10.9174705941245
7069	Vietnam	2012	12.84041715509	0.575078672	2.11256607265324	10.7278510824368
7070	Vietnam	2013	12.67167546683	0.581029215	2.11283160124384	10.5588438655862
7071	Vietnam	2014	12.55333423192	0.586913815	2.1160403233759	10.4372939085441
7072	Vietnam	2015	12.52720035078	0.592849573	2.112942477776	10.414257873004
7073	Vietnam	2016	12.56296861646	0.598849485	2.11336307195226	10.4496055445077
7074	Vietnam	2017	12.62602355222	0.605019421	2.11073653025733	10.5152870219627
7075	Vietnam	2018	12.70013813622	0.611351406	2.11349762652978	10.5866405096902

TableS2 Frontier analysis of Smoking-Related Low Back Pain among WCBA

	location	year	val	SDI	frontier	eff_diff
7076	Vietnam	2019	12.79827200025	0.617733815	2.11272346606474	10.6855485341853
7077	Vietnam	2020	12.96578292255	0.62309032	2.11146957867757	10.8543133438724
7078	Vietnam	2021	13.02760651975	0.627933721	2.11509663135828	10.9125098883917
7079	Virgin Islands	1990	65.97263088006	0.655160856	2.1126812984975	63.8599495815625
7080	Virgin Islands	1991	67.48489748254	0.664018659	2.11342688938987	65.3714705931501
7081	Virgin Islands	1992	68.64312352349	0.671081871	2.11157143005747	66.5315520934325
7082	Virgin Islands	1993	69.67735983649	0.676872745	2.11476989503171	67.5625899414583
7083	Virgin Islands	1994	70.60962286468	0.681982223	2.1134473410891	68.4961755235909
7084	Virgin Islands	1995	70.96857868054	0.686730211	2.11349797399019	68.8550807065498
7085	Virgin Islands	1996	71.13318393928	0.691247699	2.11341820076618	69.0197657385138
7086	Virgin Islands	1997	71.00714985253	0.695598204	2.11545499991198	68.891694852618
7087	Virgin Islands	1998	70.77409646245	0.699768686	2.11360044504373	68.6604960174063
7088	Virgin Islands	1999	70.47238200258	0.703949342	2.11661321666425	68.3557687859157
7089	Virgin Islands	2000	70.34065628901	0.708436565	2.1156144233838	68.2250418656262
7090	Virgin Islands	2001	69.76918985207	0.713751115	2.11283537652663	67.6563544755434
7091	Virgin Islands	2002	68.82336818456	0.724398272	2.11223630084855	66.7111318837114
7092	Virgin Islands	2003	67.51142643905	0.73460985	2.11337357133321	65.3980528677168
7093	Virgin Islands	2004	66.05706441064	0.744613598	2.11250508894696	63.944559321693
7094	Virgin Islands	2005	64.65115993064	0.754267558	2.11184736755398	62.539312563086
7095	Virgin Islands	2006	63.07256041372	0.763447496	2.11384349758174	60.9587169161383
7096	Virgin Islands	2007	61.28599948022	0.772180593	2.11190839877726	59.1740910814427
7097	Virgin Islands	2008	59.71992304824	0.780342623	2.11342751991689	57.6064955283231
7098	Virgin Islands	2009	58.22486306709	0.787055146	2.11458516790531	56.1102778991847
7099	Virgin Islands	2010	57.37808458073	0.793219696	2.11423077191394	55.2638538088161
7100	Virgin Islands	2011	56.8517110542	0.798071698	2.11471332299667	54.7369977312033

TableS2 Frontier analysis of Smoking-Related Low Back Pain among WCBA

	location	year	val	SDI	frontier	eff_diff
7101	Virgin Islands	2012	56.36466082569	0.801092851	2.11261481958667	54.2520460061033
7102	Virgin Islands	2013	55.97296756648	0.803405789	2.11386440282957	53.8591031636504
7103	Virgin Islands	2014	55.62170924804	0.805641378	2.11181971478164	53.5098895332584
7104	Virgin Islands	2015	55.38485694371	0.807889245	2.11444665412484	53.2704102895852
7105	Virgin Islands	2016	55.17671421231	0.810199044	2.11450875332628	53.0622054589837
7106	Virgin Islands	2017	54.95595758221	0.812405172	2.11530166078824	52.8406559214218
7107	Virgin Islands	2018	54.90142836678	0.81482544	2.11639078015319	52.7850375866268
7108	Virgin Islands	2019	54.71887553159	0.817429008	2.11319678744737	52.6056787441426
7109	Virgin Islands	2020	54.82460140102	0.819601712	2.11522751548672	52.7093738855333
7110	Virgin Islands	2021	54.64792328351	0.821830853	2.11621414147109	52.5317091420389
7111	Western Europe	1990	256.24587236249	0.746400479	2.11445477202902	254.131417590461
7112	Western Europe	1991	256.07105701756	0.751670034	2.110991683296	253.960065334264
7113	Western Europe	1992	256.86200614057	0.757021934	2.11307310131743	254.748933039253
7114	Western Europe	1993	257.84253881958	0.762056783	2.11417426579169	255.728364553788
7115	Western Europe	1994	259.14804976602	0.766693114	2.11191451460592	257.036135251414
7116	Western Europe	1995	261.33138196146	0.770621721	2.11174027920939	259.219641682251
7117	Western Europe	1996	263.76426724451	0.774079855	2.11371677313471	261.650550471375
7118	Western Europe	1997	265.34529186051	0.777577691	2.11474888772338	263.230542972787
7119	Western Europe	1998	266.79640530925	0.780866892	2.1166389311421	264.679766378108
7120	Western Europe	1999	268.13356545009	0.784019197	2.1151176818589	266.018447768231
7121	Western Europe	2000	269.11763273181	0.787400011	2.11337332310545	267.004259408705
7122	Western Europe	2001	269.66051338123	0.790904403	2.11253066617319	267.547982715057
7123	Western Europe	2002	269.23181958174	0.794165178	2.11463508664273	267.117184495097
7124	Western Europe	2003	268.15784963093	0.796961505	2.11423283006597	266.043616800864
7125	Western Europe	2004	266.84409031827	0.79971572	2.11312020399913	264.730970114271

TableS2 Frontier analysis of Smoking-Related Low Back Pain among WCBA

	location	year	val	SDI	frontier	eff_diff
7126	Western Europe	2005	265.55727718581	0.802414038	2.113808103104	263.443469082706
7127	Western Europe	2006	263.8965557019	0.80506685	2.1177561573671	261.778799544533
7128	Western Europe	2007	261.93121337663	0.807723334	2.11668665795442	259.814526718676
7129	Western Europe	2008	259.49811609892	0.810484377	2.11489747754714	257.383218621373
7130	Western Europe	2009	257.03212711643	0.813110222	2.11058871433195	254.921538402098
7131	Western Europe	2010	254.77284522067	0.816142165	2.11526805451304	252.657577166157
7132	Western Europe	2011	251.96067399445	0.819375239	2.11323300122232	249.847440993228
7133	Western Europe	2012	248.16113214496	0.822633726	2.11523878762493	246.045893357335
7134	Western Europe	2013	243.86799666644	0.825786901	2.11517501044048	241.752821656
7135	Western Europe	2014	239.59731733247	0.828609513	2.11379030080037	237.48352703167
7136	Western Europe	2015	236.12741200667	0.831321799	2.11053307100289	234.016878935667
7137	Western Europe	2016	233.49344993489	0.834216019	2.11478338253879	231.378666552351
7138	Western Europe	2017	231.41495100793	0.837522174	2.11502766844414	229.299923339486
7139	Western Europe	2018	229.59992324837	0.84083355	2.11527814474417	227.484645103626
7140	Western Europe	2019	227.7089711951	0.844212109	2.11375438338435	225.595216811716
7141	Western Europe	2020	221.94040101759	0.846550359	2.11593525551643	219.824465762074
7142	Western Europe	2021	221.37615033618	0.848726316	2.11385289971584	219.262297436464
7143	Western sub-Saharan Africa	1990	8.69594754907	0.273700004	2.45602727954342	6.23992026952658
7144	Western sub-Saharan Africa	1991	8.70853315264	0.277410732	2.4613243879416	6.24720876469839
7145	Western sub-Saharan Africa	1992	8.69371214866	0.281024681	2.456486275056	6.237225873604
7146	Western sub-Saharan Africa	1993	8.68011819744	0.284431723	2.43318697560919	6.24693122183081
7147	Western sub-Saharan Africa	1994	8.66805438494	0.287699583	2.43469676763547	6.23335761730453
7148	Western sub-Saharan Africa	1995	8.70800119822	0.2909832	2.42752020615985	6.28048099206015
7149	Western sub-Saharan Africa	1996	8.7475627896	0.294618466	2.38227558357484	6.36528720602516
7150	Western sub-Saharan Africa	1997	8.79243670613	0.298302443	2.40327436656394	6.38916233956606

TableS2 Frontier analysis of Smoking-Related Low Back Pain among WCBA

	location	year	val	SDI	frontier	eff_diff
7151	Western sub-Saharan Africa	1998	8.83994668051	0.302025797	2.40251790953923	6.43742877097077
7152	Western sub-Saharan Africa	1999	8.87719848929	0.305809779	2.3835442546805	6.4936542346095
7153	Western sub-Saharan Africa	2000	8.8674692816	0.309780958	2.38438901808761	6.48308026351239
7154	Western sub-Saharan Africa	2001	8.85905066796	0.314028092	2.36497694761554	6.49407372034446
7155	Western sub-Saharan Africa	2002	8.83977351437	0.318880999	2.3390010886307	6.5007724257393
7156	Western sub-Saharan Africa	2003	8.80263827613	0.324232893	2.32222949817522	6.48040877795478
7157	Western sub-Saharan Africa	2004	8.75244371358	0.330228268	2.30573500701673	6.44670870656327
7158	Western sub-Saharan Africa	2005	8.70207644738	0.336779707	2.27285858458603	6.42921786279397
7159	Western sub-Saharan Africa	2006	8.6066353243	0.343248175	2.21354298888729	6.39309233541271
7160	Western sub-Saharan Africa	2007	8.46466036647	0.350040166	2.1235567657829	6.3411036006871
7161	Western sub-Saharan Africa	2008	8.28441123649	0.356716457	2.11553066358079	6.16888057290921
7162	Western sub-Saharan Africa	2009	8.10061390231	0.363491543	2.11102979793452	5.98958410437548
7163	Western sub-Saharan Africa	2010	7.92876114733	0.370608753	2.11396262928828	5.81479851804172
7164	Western sub-Saharan Africa	2011	7.74636501512	0.377609144	2.11738432268382	5.62898069243618
7165	Western sub-Saharan Africa	2012	7.53567049493	0.384753386	2.11523530112093	5.42043519380907
7166	Western sub-Saharan Africa	2013	7.31861780223	0.391890443	2.1111571876272	5.2074606146028
7167	Western sub-Saharan Africa	2014	7.12345491683	0.39916209	2.11676046320538	5.00669445362462
7168	Western sub-Saharan Africa	2015	6.96657135918	0.406238201	2.11122830552197	4.85534305365803
7169	Western sub-Saharan Africa	2016	6.83828469098	0.412927287	2.11149261250354	4.72679207847646
7170	Western sub-Saharan Africa	2017	6.71721241229	0.419615019	2.11389888313195	4.60331352915805
7171	Western sub-Saharan Africa	2018	6.60471389464	0.426437347	2.11319761561544	4.49151627902456
7172	Western sub-Saharan Africa	2019	6.51294469912	0.433383889	2.11346762282288	4.39947707629712
7173	Western sub-Saharan Africa	2020	6.42873974086	0.439822327	2.11315478402872	4.31558495683128
7174	Western sub-Saharan Africa	2021	6.3456618399	0.446022979	2.11541270181537	4.23024913808463
7175	Yemen	1990	71.31097041612	0.215664586	3.4405463258062	67.8704240903138

TableS2 Frontier analysis of Smoking-Related Low Back Pain among WCBA

	location	year	val	SDI	frontier	eff_diff
7176	Yemen	1991	71.60475243285	0.222885409	2.80460566305542	68.8001467697946
7177	Yemen	1992	71.81433247879	0.230415046	2.68816595603984	69.1261665227502
7178	Yemen	1993	71.60661745738	0.238025246	2.6398464418988	68.9667710154812
7179	Yemen	1994	71.33983557699	0.245879426	2.54447054098636	68.7953650360036
7180	Yemen	1995	70.92101668823	0.253962572	2.51178634164523	68.4092303465848
7181	Yemen	1996	70.22463088725	0.262278613	2.48538046549567	67.7392504217543
7182	Yemen	1997	69.35860154557	0.270782536	2.47225620778714	66.8863453377829
7183	Yemen	1998	68.43312244824	0.279500507	2.44282586596654	65.9902965822735
7184	Yemen	1999	67.4710244993	0.288261301	2.43098225379351	65.0400422455065
7185	Yemen	2000	66.80863029763	0.297145157	2.40521433238162	64.4034159652484
7186	Yemen	2001	65.64891001273	0.306112424	2.38670500531895	63.2622050074111
7187	Yemen	2002	64.55054317884	0.315065023	2.36132338340458	62.1892197954354
7188	Yemen	2003	63.58525967441	0.324081689	2.32475815936875	61.2605015150412
7189	Yemen	2004	62.86552059328	0.333371285	2.29010394882095	60.575416644459
7190	Yemen	2005	62.44278538007	0.343462562	2.17154402669016	60.2712413533798
7191	Yemen	2006	62.38412544879	0.353472947	2.115758341598	60.268367107192
7192	Yemen	2007	62.51336307096	0.363301218	2.11847289140131	60.3948901795587
7193	Yemen	2008	62.86547319429	0.373140288	2.11752938679341	60.7479438074966
7194	Yemen	2009	63.35572684034	0.382762252	2.11432129213159	61.2414055482084
7195	Yemen	2010	63.78883359136	0.392936641	2.10890628574853	61.6799273056115
7196	Yemen	2011	64.29579672454	0.401471801	2.11277035999286	62.1830263645471
7197	Yemen	2012	64.55158551903	0.409561349	2.11300312888797	62.438582390142
7198	Yemen	2013	64.85620626942	0.417642405	2.1111708793	62.74503539012
7199	Yemen	2014	65.08923943932	0.425189667	2.11544963792343	62.9737898013966
7200	Yemen	2015	65.45684982981	0.430197789	2.11118619816883	63.3456636316412

TableS2 Frontier analysis of Smoking-Related Low Back Pain among WCBA

	location	year	val	SDI	frontier	eff_diff
7201	Yemen	2016	66.19656467855	0.434232983	2.11287576010214	64.0836889184479
7202	Yemen	2017	66.86713275764	0.437549155	2.11303609296905	64.7540966646709
7203	Yemen	2018	67.3942466397	0.440727991	2.11437359036971	65.2798730493303
7204	Yemen	2019	67.8268703488	0.4440014	2.11437931639737	65.7124910324026
7205	Yemen	2020	67.90497377448	0.446893069	2.11532767981638	65.7896460946636
7206	Yemen	2021	68.07760518218	0.450376375	2.11239533422096	65.965209847959
7207	Zambia	1990	6.29935879375	0.304008549	2.3838976610455	3.9154611327045
7208	Zambia	1991	6.39912762636	0.30584236	2.38600207547132	4.01312555088868
7209	Zambia	1992	6.50767431487	0.307316277	2.38266846021943	4.12500585465057
7210	Zambia	1993	6.60146012105	0.308780208	2.38643888473944	4.21502123631056
7211	Zambia	1994	6.6933481366	0.309223449	2.38826026100481	4.30508787559519
7212	Zambia	1995	6.73069673028	0.309995813	2.38514136187641	4.34555536840359
7213	Zambia	1996	6.78943705976	0.311326691	2.36091791968966	4.42851914007034
7214	Zambia	1997	6.83495345759	0.31308733	2.35561278142623	4.47934067616377
7215	Zambia	1998	6.87345561684	0.314926435	2.35835951692831	4.51509609991169
7216	Zambia	1999	6.90946810412	0.317396437	2.34156943848469	4.56789866563531
7217	Zambia	2000	6.99371185746	0.320625322	2.34536124553071	4.64835061192929
7218	Zambia	2001	7.03386045025	0.324401368	2.32046353609088	4.71339691415912
7219	Zambia	2002	7.07207668621	0.328680122	2.30278875471552	4.76928793149448
7220	Zambia	2003	7.10445251383	0.333721398	2.28198360882344	4.82246890500656
7221	Zambia	2004	7.11502506611	0.339488917	2.27102138650684	4.84400367960316
7222	Zambia	2005	7.10480598973	0.346048864	2.13934882153838	4.96545716819162
7223	Zambia	2006	7.02773523267	0.353540709	2.11963275746348	4.90810247520652
7224	Zambia	2007	6.90539816591	0.362450967	2.11215473348263	4.79324343242737
7225	Zambia	2008	6.76411218677	0.371688496	2.11884965205576	4.64526253471424

TableS2 Frontier analysis of Smoking-Related Low Back Pain among WCBA

	location	year	val	SDI	frontier	eff_diff
7226	Zambia	2009	6.60349791894	0.381667118	2.11207134258545	4.49142657635455
7227	Zambia	2010	6.45735953029	0.392470989	2.1152006696674	4.3421588606226
7228	Zambia	2011	6.28867372336	0.403475629	2.11146081297984	4.17721291038016
7229	Zambia	2012	6.08803350266	0.414574501	2.11277902267463	3.97525447998538
7230	Zambia	2013	5.87837074642	0.425492302	2.114650282318	3.763720464102
7231	Zambia	2014	5.68481057429	0.436244006	2.11202042307682	3.57279015121318
7232	Zambia	2015	5.54558312901	0.446744719	2.11409764835175	3.43148548065825
7233	Zambia	2016	5.50689413472	0.457122596	2.11581586562773	3.39107826909227
7234	Zambia	2017	5.54746223527	0.467532631	2.11169720554592	3.43576502972408
7235	Zambia	2018	5.61704079225	0.477887712	2.11345823823318	3.50358255401682
7236	Zambia	2019	5.64463591702	0.487911523	2.11289802603105	3.53173789098895
7237	Zambia	2020	5.62681010767	0.497485561	2.11323005564475	3.51358005202525
7238	Zambia	2021	5.55418950537	0.505948954	2.11247306752987	3.44171643784013
7239	Zimbabwe	1990	6.55876018073	0.398559341	2.11410789131289	4.44465228941711
7240	Zimbabwe	1991	6.61241884524	0.406052877	2.11634342673648	4.49607541850352
7241	Zimbabwe	1992	6.66965806688	0.412083826	2.11627636790621	4.55338169897379
7242	Zimbabwe	1993	6.74780241631	0.4179742	2.11235649901646	4.63544591729354
7243	Zimbabwe	1994	6.81272622946	0.424051564	2.11172656559075	4.70099966386925
7244	Zimbabwe	1995	6.85373272291	0.42892904	2.11616358057631	4.73756914233369
7245	Zimbabwe	1996	6.95523828123	0.434187192	2.11370709975421	4.84153118147579
7246	Zimbabwe	1997	7.09021119326	0.438141988	2.11719739951379	4.97301379374621
7247	Zimbabwe	1998	7.23430205898	0.441093135	2.11220911167894	5.12209294730106
7248	Zimbabwe	1999	7.37513405522	0.443246604	2.11111875290701	5.26401530231299
7249	Zimbabwe	2000	7.50560995631	0.444841402	2.11281544211538	5.39279451419462
7250	Zimbabwe	2001	7.57153513308	0.446354681	2.1163021354368	5.4552329976432

TableS2 Frontier analysis of Smoking-Related Low Back Pain among WCBA

	location	year	val	SDI	frontier	eff_diff
7251	Zimbabwe	2002	7.66774928442	0.446970895	2.11112912247534	5.55662016194466
7252	Zimbabwe	2003	7.78913379758	0.446002182	2.11574167853082	5.67339211904918
7253	Zimbabwe	2004	7.87644821994	0.443696404	2.11368094895164	5.76276727098836
7254	Zimbabwe	2005	7.93650618631	0.439651657	2.11796136266356	5.81854482364644
7255	Zimbabwe	2006	7.89210604326	0.434405758	2.11202521368754	5.78008082957246
7256	Zimbabwe	2007	7.7321471261	0.42916514	2.11026141611419	5.62188570998581
7257	Zimbabwe	2008	7.52101608946	0.422448974	2.11371804523219	5.40729804422781
7258	Zimbabwe	2009	7.28577791535	0.418330613	2.11268480013725	5.17309311521275
7259	Zimbabwe	2010	7.09927137901	0.417305357	2.11226648254192	4.98700489646808
7260	Zimbabwe	2011	6.96843001701	0.419729695	2.11234821668186	4.85608180032814
7261	Zimbabwe	2012	6.81645381023	0.42574852	2.11202968078398	4.70442412944602
7262	Zimbabwe	2013	6.67016205667	0.432881888	2.11290402589368	4.55725803077632
7263	Zimbabwe	2014	6.56179774277	0.439997226	2.11448370118255	4.44731404158745
7264	Zimbabwe	2015	6.47676230253	0.446552872	2.11220157521658	4.36456072731342
7265	Zimbabwe	2016	6.48396816124	0.452473037	2.11202037300367	4.37194778823633
7266	Zimbabwe	2017	6.49578203171	0.458424984	2.11334962917178	4.38243240253822
7267	Zimbabwe	2018	6.54252105329	0.464379262	2.11182237505972	4.43069867823028
7268	Zimbabwe	2019	6.55967537716	0.468384438	2.11326278177434	4.44641259538566
7269	Zimbabwe	2020	6.53692942435	0.471573171	2.11554199610595	4.42138742824405
7270	Zimbabwe	2021	6.45204773822	0.473819486	2.11390302513726	4.33814471308274

TableS3 Frontier analysis（2021）

	location	year	val	SDI	frontier	eff_diff	trend
1	Afghanistan	2021	22.9657469654	0.337199998	2.270295904184	20.695451061216	Increase
2	Albania	2021	127.50559897743	0.706849791	2.11126240145721	125.394336575973	Increase
3	Algeria	2021	11.81888951164	0.659500924	2.11279186783493	9.70609764380507	Increase
4	American Samoa	2021	112.30714401632	0.723727533	2.11016658495132	110.196977431369	Increase
5	Andean Latin America	2021	19.67841192151	0.651602456	2.11329295101214	17.5651189704979	Increase
6	Andorra	2021	261.59580842411	0.869444113	2.11322744900885	259.482580975101	Increase
7	Angola	2021	18.63465897464	0.453721949	2.113642462248	16.521016512392	Decrease
8	Antigua and Barbuda	2021	47.3596746013	0.749886887	2.11421824411145	45.2454563571886	Increase
9	Argentina	2021	235.45087488014	0.723122973	2.11252790168767	233.338346978452	Decrease
10	Armenia	2021	40.23935325744	0.701833194	2.11509070888232	38.1242625485577	Increase
11	Australasia	2021	287.88908337378	0.845514063	2.11315463173491	285.775928742045	Decrease
12	Australia	2021	289.61811256652	0.844252814	2.11228266219289	287.505829904327	Decrease
13	Austria	2021	223.20428592982	0.853837004	2.11258661507391	221.091699314746	Increase
14	Azerbaijan	2021	25.02535220875	0.694851274	2.1119651435896	22.9133870651604	Increase
15	Bahamas	2021	33.52301665491	0.805020668	2.11420406090712	31.4088125940029	Increase
16	Bahrain	2021	37.77517945685	0.753043204	2.11324836877494	35.6619310880751	Decrease
17	Bangladesh	2021	20.94375129667	0.492420885	2.11728928700972	18.8264620096603	Increase
18	Barbados	2021	34.33007283129	0.746748764	2.11587519624065	32.2141976350494	Decrease
19	Belarus	2021	199.48074110382	0.784484711	2.11175246129773	197.368988642522	Increase
20	Belgium	2021	231.53015917972	0.853654016	2.11184458401032	229.41831459571	Decrease
21	Belize	2021	26.80074330373	0.610229002	2.11301285810435	24.6877304456257	Increase
22	Benin	2021	6.80216838226	0.373486574	2.11382601642246	4.68834236583754	Decrease
23	Bermuda	2021	94.21677592004	0.821365422	2.11585482264125	92.1009210973987	Increase
24	Bhutan	2021	41.23848087577	0.473062378	2.11205100327595	39.1264298724941	Increase
25	Bolivia	2021	17.2554596077	0.599010799	2.11231590556903	15.143143702131	Decrease
26	Bosnia and Herzegovina	2021	425.30869318157	0.723077893	2.11375972816413	423.194933453406	Increase

TableS3 Frontier analysis（2021）

	location	year	val	SDI	frontier	eff_diff	trend
27	Botswana	2021	29.30736730431	0.642721629	2.11000390228582	27.1973634020242	Decrease
28	Brazil	2021	102.43397831006	0.653043887	2.11577283072833	100.318205479332	Decrease
29	Brunei	2021	59.22347741857	0.810234367	2.11430711039229	57.1091703081777	Decrease
30	Bulgaria	2021	459.59425928615	0.768150939	2.11259791218055	457.481661373969	Increase
31	Burkina Faso	2021	6.43952816876	0.285118402	2.42830784262289	4.0112203261371	Decrease
32	Burundi	2021	12.08600276727	0.289374365	2.4437884001056	9.6422143671644	Decrease
33	Cabo Verde	2021	15.00162868309	0.533534539	2.11134293181627	12.8902857512737	Decrease
34	Cambodia	2021	24.83647435358	0.473621491	2.11184980982887	22.7246245437511	Increase
35	Cameroon	2021	5.38879850563	0.479691223	2.11422362606679	3.2745748795632	Decrease
36	Canada	2021	211.47323055982	0.87317068	2.11454446950206	209.358686090318	Decrease
37	Caribbean	2021	50.8738622025	0.642003055	2.11260786941386	48.7612543330861	Decrease
38	Central African Republic	2021	8.81061724653	0.30916769	2.38426731512647	6.42634993140353	Decrease
39	Central Asia	2021	40.65886516653	0.675163978	2.11308073558447	38.5457844309455	Increase
40	Central Europe	2021	366.5955374086	0.796244448	2.11376222366548	364.481775184934	Decrease
41	Central Latin America	2021	50.29053761521	0.6406851	2.11405434952384	48.1764832656862	Decrease
42	Central sub-Saharan Africa	2021	10.12412077232	0.472255651	2.11188927539583	8.01223149692417	Decrease
43	Chad	2021	9.01868296066	0.240436019	2.54289889601036	6.47578406464964	Decrease
44	Chile	2021	321.19909092946	0.771514716	2.1148423235885	319.084248605871	Increase
45	China	2021	16.56366537042	0.72162976	2.11257577869628	14.4510895917237	Decrease
46	Colombia	2021	67.14285637172	0.655442913	2.11223652640074	65.0306198453193	Decrease
47	Comoros	2021	11.47106616129	0.475978688	2.11134255439247	9.35972360689753	Increase
48	Congo (Brazzaville)	2021	9.18203540738	0.583075236	2.11374386870859	7.06829153867141	Increase
49	Cook Islands	2021	111.35868639155	0.779109955	2.11344664455431	109.245239746996	Increase
50	Costa Rica	2021	71.18883223967	0.700340477	2.11345555699798	69.075376682672	Decrease
51	Cote d'Ivoire	2021	10.02576679755	0.425941883	2.11170821594123	7.91405858160877	Decrease
52	Croatia	2021	395.05436634392	0.798341027	2.11130512856396	392.943061215356	Decrease

TableS3 Frontier analysis（2021）

	location	year	val	SDI	frontier	eff_diff	trend
53	Cuba	2021	87.40559984368	0.668729864	2.11194714824364	85.2936526954364	Decrease
54	Cyprus	2021	238.21164362246	0.835630545	2.11293851880029	236.09870510366	Increase
55	Czechia	2021	376.53771131949	0.828450433	2.11321634309222	374.424494976398	Decrease
56	DR Congo	2021	7.20818733363	0.383179849	2.11114761454167	5.09703971908833	Decrease
57	Democratic Republic of Sao Tome and Principe	2021	4.81086740003	0.505413747	2.11183110369	2.69903629634	Increase
58	Denmark	2021	249.49320715652	0.896424204	2.11175443407346	247.381452722447	Decrease
59	Djibouti	2021	16.18632301261	0.487958371	2.1151519295424	14.0711710830676	Increase
60	Dominica	2021	32.47036205712	0.746967185	2.11323637693643	30.3571256801836	Increase
61	Dominican Republic	2021	53.68621123845	0.619388201	2.11490608563988	51.5713051528101	Decrease
62	East Asia	2021	17.2381511016	0.725704902	2.1112972769127	15.1268538246873	Decrease
63	Eastern Europe	2021	226.91913960283	0.802851009	2.11091674059868	224.808222862231	Increase
64	Eastern sub-Saharan Africa	2021	13.03835166205	0.409720983	2.11704718729747	10.9213044747525	Decrease
65	Ecuador	2021	18.49997570619	0.661017053	2.11366723857154	16.3863084676185	Decrease
66	Egypt	2021	7.3766627756	0.606787094	2.11317402162853	5.26348875397147	Increase
67	El Salvador	2021	39.02339913795	0.563775188	2.11470738200179	36.9086917559482	Increase
68	Equatorial Guinea	2021	7.74700384095	0.657857456	2.11236608296708	5.63463775798292	Decrease
69	Eritrea	2021	2.29456044932	0.403863943	2.11047819562095	0.184082253699054	Decrease
70	Estonia	2021	236.38594369488	0.844917787	2.11327205738268	234.272671637497	Increase
71	Ethiopia	2021	4.02457678749	0.358823295	2.12245854249242	1.90211824499758	Decrease
72	Fiji	2021	71.11123945325	0.675051631	2.1136883291502	68.9975511240998	Increase
73	Finland	2021	162.0040516773	0.859831368	2.11355707107274	159.890494606227	Decrease
74	France	2021	257.35065365929	0.838364875	2.11258077182793	255.238072887462	Increase
75	Gabon	2021	11.85221801666	0.634691393	2.11398276110418	9.73823525555582	Increase
76	Gambia	2021	3.06987489781	0.40971416	2.112425875635	0.957449022175	Decrease
77	Georgia	2021	83.22832775184	0.732473604	2.11223005387847	81.1160976979615	Increase
78	Germany	2021	263.01221472328	0.902957091	2.11153592739474	260.900678795885	Decrease

TableS3 Frontier analysis（2021）

	location	year	val	SDI	frontier	eff_diff	trend
79	Ghana	2021	10.0781583161	0.56493039	2.11373999607506	7.96441832002494	Increase
80	Global	2021	56.54082767157	0.665820975	2.11288358566958	54.4279440859004	Decrease
81	Greece	2021	300.34795381744	0.791854408	2.11071292969421	298.237240887746	Increase
82	Greenland	2021	295.40233463499	0.826210336	2.11176035634724	293.290574278643	Increase
83	Grenada	2021	32.84812334592	0.668993028	2.11333018026582	30.7347931656542	Increase
84	Guam	2021	108.18081445237	0.803982203	2.11482981166061	106.065984640709	Increase
85	Guatemala	2021	27.60924515229	0.539972424	2.11431857062656	25.4949265816634	Decrease
86	Guinea	2021	7.6518692258	0.336401293	2.28772616935889	5.36414305644111	Decrease
87	Guinea-Bissau	2021	6.07989072378	0.353109621	2.11133802715544	3.96855269662456	Increase
88	Guyana	2021	24.12431113404	0.650812335	2.1150318693025	22.0092792647375	Decrease
89	Haiti	2021	22.93085647993	0.448278285	2.11452920447292	20.8163272754571	Decrease
90	High-income Asia Pacific	2021	137.39044084084	0.876767	2.11271073164964	135.27773010919	Decrease
91	High-income North America	2021	213.34604828973	0.863465474	2.11385916074767	211.232189128982	Decrease
92	Honduras	2021	32.78122389194	0.513037248	2.11269506370924	30.6685288282308	Decrease
93	Hungary	2021	390.53148065304	0.790754768	2.11499180422485	388.416488848815	Decrease
94	Iceland	2021	177.02736815513	0.87636168	2.11307579791879	174.914292357211	Decrease
95	India	2021	13.41371760274	0.575401649	2.11515340393988	11.2985641988001	Decrease
96	Indonesia	2021	18.78161101727	0.656868336	2.11362266487179	16.6679883523982	Increase
97	Iran	2021	51.70691578951	0.697207398	2.11541161047173	49.5915041790383	Increase
98	Iraq	2021	21.64823064097	0.662626231	2.11597307848866	19.5322575624813	Decrease
99	Ireland	2021	191.87545304048	0.87375385	2.11420762336062	189.761245417119	Decrease
100	Israel	2021	146.00239693378	0.809011652	2.11386894138695	143.888527992393	Decrease
101	Italy	2021	207.50625690592	0.805773534	2.11146950557582	205.394787400344	Decrease
102	Jamaica	2021	45.84650990034	0.683263064	2.11441310116206	43.7320967991779	Decrease
103	Japan	2021	175.70873014633	0.871241813	2.11555417018254	173.593175976147	Decrease
104	Jordan	2021	70.3055523294	0.725307227	2.11270926095818	68.1928430684418	Increase

TableS3 Frontier analysis（2021）

	location	year	val	SDI	frontier	eff_diff	trend
105	Kazakhstan	2021	73.53754074094	0.725144495	2.11398212014603	71.423558620794	Increase
106	Kenya	2021	6.63934798142	0.523768077	2.11549761703844	4.52385036438156	Decrease
107	Kiribati	2021	127.91438509291	0.527186583	2.11600942511456	125.798375667795	Increase
108	Kuwait	2021	45.16116956598	0.846651055	2.11440854624549	43.0467610197345	Increase
109	Kyrgyzstan	2021	53.04101700172	0.603979328	2.11376693754726	50.9272500641727	Increase
110	Laos	2021	27.7282098213	0.489136091	2.11300115346696	25.615208667833	Increase
111	Latvia	2021	216.8839904587	0.830663516	2.11353800259174	214.770452456108	Increase
112	Lebanon	2021	184.22823627689	0.744746351	2.11386168190904	182.114374594981	Increase
113	Lesotho	2021	3.92669454063	0.510393066	2.11444853529627	1.81224600533373	Decrease
114	Liberia	2021	8.03252331511	0.352442452	2.11437557371492	5.91814774139508	Decrease
115	Libya	2021	9.53732154388	0.725771399	2.11118798323839	7.42613356064161	Increase
116	Lithuania	2021	203.5066371628	0.856484049	2.11260508358886	201.394032079211	Increase
117	Low-middle SDI	2021	20.51758321601	NA	20.51758321601	0	Decrease
118	Luxembourg	2021	205.55432228888	0.884428955	2.1106409707725	203.443681318107	Increase
119	Madagascar	2021	9.02353928132	0.400246943	2.1117401803877	6.91179910093229	Decrease
120	Malawi	2021	8.37236723455	0.384553634	2.1149272915631	6.25743994298691	Decrease
121	Malaysia	2021	10.26298019317	0.742523828	2.11334380776761	8.14963638540239	Decrease
122	Maldives	2021	23.73325266898	0.650886627	2.11402367039227	21.6192289985877	Increase
123	Mali	2021	5.95865229735	0.268579941	2.49119354314138	3.46745875420861	Decrease
124	Malta	2021	244.71277432811	0.801585034	2.11409710899259	242.598677219117	Increase
125	Marshall Islands	2021	49.71760856713	0.574091128	2.11208765512974	47.6055209120003	Increase
126	Mauritania	2021	25.33641190452	0.4989451	2.11204599442712	23.2243659100929	Decrease
127	Mauritius	2021	27.94678018279	0.718260446	2.11413452726064	25.8326456555294	Decrease
128	Mexico	2021	43.74884043524	0.664575304	2.11419342782778	41.6346470074122	Decrease
129	Micronesia	2021	115.17548477511	0.587534967	2.11224882927474	113.063235945835	Increase
130	Moldova	2021	97.07859517424	0.732214875	2.11449532190986	94.9640998523302	Decrease

TableS3 Frontier analysis（2021）

	location	year	val	SDI	frontier	eff_diff	trend
131	Monaco	2021	243.25277539974	0.908262831	2.11101482362129	241.141760576119	Increase
132	Mongolia	2021	69.86632467614	0.617621565	2.11280890195846	67.7535157741815	Decrease
133	Montenegro	2021	467.74904894013	0.795800584	2.11304053871081	465.636008401419	Increase
134	Morocco	2021	9.47321222007	0.562698301	2.11779430090859	7.35541791916141	Decrease
135	Mozambique	2021	15.80232637638	0.326462614	2.32131496070022	13.4810114156798	Decrease
136	Myanmar	2021	16.64018776998	0.53390084	2.11279654848055	14.5273912214995	Decrease
137	Namibia	2021	21.62441138248	0.617564872	2.11297202350636	19.5114393589736	Decrease
138	Nauru	2021	139.14427796875	0.625177834	2.10985738086083	137.034420587889	Decrease
139	Nepal	2021	72.81641296767	0.433174635	2.11271827196719	70.7036946957028	Decrease
140	Netherlands	2021	169.81238744944	0.888464256	2.11177665625271	167.700610793187	Decrease
141	New Zealand	2021	279.17073162996	0.849442499	2.11468020606018	277.0560514239	Increase
142	Nicaragua	2021	53.26304217119	0.523958472	2.1117866633875	51.1512555078025	Increase
143	Niger	2021	4.26730955785	0.168072774	3.81168509583641	0.455624462013594	Decrease
144	Nigeria	2021	4.7589530053	0.503390833	2.11859200671063	2.64036099858937	Decrease
145	Niue	2021	87.39166325713	0.72622205	2.11442459893393	85.2772386581961	Increase
146	North Africa and Middle East	2021	45.54012415228	0.658224715	2.11471005818299	43.425414094097	Increase
147	North Korea	2021	28.09884467731	0.569854634	2.11301023354623	25.9858344437638	Decrease
148	North Macedonia	2021	397.4947543517	0.750629703	2.11591068482771	395.378843666872	Increase
149	Northern Mariana Islands	2021	92.30992352531	0.771535213	2.11628484038048	90.1936386849295	Decrease
150	Norway	2021	192.15567086426	0.91613281	2.11331613808206	190.042354726178	Increase
151	Oceania	2021	77.25314626694	0.467445126	2.11861730161859	75.1345289653214	Increase
152	Oman	2021	19.7269241096	0.773391602	2.11344105490862	17.6134830546914	Decrease
153	Pakistan	2021	20.09967467965	0.504028689	2.11489812850211	17.9847765511479	Decrease
154	Palau	2021	56.74750952449	0.754046931	2.11442039821569	54.6330891262743	Increase
155	Palestine	2021	24.68549057588	0.631011665	2.11316409978052	22.5723264760995	Decrease
156	Panama	2021	32.02788818641	0.708864828	2.11448118414269	29.9134070022673	Decrease

TableS3 Frontier analysis（2021）

	location	year	val	SDI	frontier	eff_diff	trend
157	Papua New Guinea	2021	78.49686860151	0.417797443	2.11028411329423	76.3865844882158	Increase
158	Paraguay	2021	62.90131540698	0.635718099	2.11255710765518	60.7887582993248	Increase
159	Peru	2021	21.04153787677	0.662054037	2.11281189657711	18.9287259801929	Decrease
160	Philippines	2021	33.93163199155	0.651219329	2.112925144337	31.818706847213	Decrease
161	Poland	2021	339.46501514597	0.812042809	2.11452988290488	337.350485263065	Increase
162	Portugal	2021	246.048208607	0.744151851	2.11184033842125	243.936368268579	Increase
163	Puerto Rico	2021	70.53391063466	0.825525847	2.11415663956154	68.4197539950984	Increase
164	Qatar	2021	23.90308420134	0.846860584	2.11158732467094	21.7914968766691	Decrease
165	Romania	2021	357.82340451838	0.768453864	2.11798043892164	355.705424079458	Increase
166	Russia	2021	233.57386464214	0.808536005	2.1102020247875	231.463662617352	Increase
167	Rwanda	2021	25.37041103343	0.435588706	2.11918549636118	23.2512255370688	Increase
168	Saint Kitts and Nevis	2021	19.90002299523	0.754987055	2.11397474816692	17.7860482470631	Decrease
169	Saint Lucia	2021	36.69439789473	0.672509735	2.1134820910582	34.5809158036718	Increase
170	Saint Vincent and the Grenadines	2021	28.89001171275	0.637195963	2.11162993577759	26.7783817769724	Decrease
171	Samoa	2021	74.92530368221	0.593392769	2.11159587238311	72.8137078098269	Decrease
172	San Marino	2021	221.60496878258	0.888005474	2.11221006840963	219.49275871417	Increase
173	Saudi Arabia	2021	20.14481593383	0.815143493	2.11423174262476	18.0305841912052	Increase
174	Senegal	2021	4.02953916315	0.408054193	2.11560872954355	1.91393043360645	Decrease
175	Serbia	2021	450.25729799359	0.792416294	2.11144574129432	448.145852252296	Increase
176	Seychelles	2021	31.06734677271	0.730150775	2.11366155671614	28.9536852159939	Increase
177	Sierra Leone	2021	16.26170618392	0.358665881	2.11341983438709	14.1482863495329	Decrease
178	Singapore	2021	72.3999596377	0.856097766	2.11526116476227	70.2846984729377	Decrease
179	Slovakia	2021	325.5030739532	0.81061053	2.11217922299554	323.390894730204	Increase
180	Slovenia	2021	361.22907052884	0.842430731	2.11552859650569	359.113541932334	Increase
181	Solomon Islands	2021	74.90304698437	0.429360316	2.1127907869029	72.7902561974671	Increase
182	Somalia	2021	8.35694630185	0.077688109	7.467452753336	0.889493548514	Decrease

TableS3 Frontier analysis（2021）

	location	year	val	SDI	frontier	eff_diff	trend
183	South Africa	2021	39.2124485751	0.679626598	2.11260592731536	37.0998426477846	Increase
184	South Asia	2021	16.03639714858	0.557864657	2.11340062777184	13.9229965208082	Decrease
185	South Korea	2021	64.19552045161	0.886675267	2.11147194914866	62.0840485024613	Increase
186	South Sudan	2021	8.93561741316	0.278371125	2.46296922624815	6.47264818691185	Decrease
187	Southeast Asia	2021	19.63952140856	0.649777295	2.111919460315	17.527601948245	Decrease
188	Southern Latin America	2021	259.78432573974	0.735984717	2.11792455253484	257.666401187205	Increase
189	Southern sub-Saharan Africa	2021	30.92201000752	0.642200282	2.11533762705986	28.8066723804601	Decrease
190	Spain	2021	215.37832514578	0.769283698	2.11483153044872	213.263493615331	Increase
191	Sri Lanka	2021	10.4806941935	0.701534935	2.11167827659391	8.36901591690609	Decrease
192	Sudan	2021	16.21886155769	0.541949735	2.11070532118527	14.1081562365047	Decrease
193	Suriname	2021	54.99475014207	0.633665739	2.11310998930587	52.8816401527641	Increase
194	Swaziland	2021	5.20393766042	0.585459713	2.11014771491848	3.09378994550152	Decrease
195	Sweden	2021	140.02315291237	0.886880299	2.11313338345169	137.910019528918	Decrease
196	Switzerland	2021	235.70157276356	0.933059111	2.11028814052636	233.591284623034	Increase
197	Syria	2021	44.8040498511	0.623004075	2.11372955263587	42.6903202984641	Decrease
198	Taiwan (Province of China)	2021	42.76246373246	0.874747053	2.114580960342	40.647882772118	Increase
199	Tajikistan	2021	16.44043911647	0.541511187	2.1135510756819	14.3268880407881	Decrease
200	Tanzania	2021	42.35996790571	0.446568273	2.11533393770015	40.2446339680099	Increase
201	Thailand	2021	16.55757598974	0.682547933	2.11329881014092	14.4442771795991	Decrease
202	Timor-Leste	2021	13.68138033137	0.444667619	2.11183755441948	11.5695427769505	Increase
203	Togo	2021	6.91207168856	0.408533695	2.11267031167253	4.79940137688747	Decrease
204	Tokelau	2021	78.5537634095	0.686425621	2.1155294479534	76.4382339615466	Increase
205	Tonga	2021	55.20832228164	0.626349936	2.11548140694262	53.0928408746974	Increase
206	Trinidad and Tobago	2021	55.94425002956	0.768763254	2.11320964030467	53.8310403892553	Decrease
207	Tropical Latin America	2021	101.19671446075	0.652442394	2.11267198253667	99.0840424782133	Increase
208	Tunisia	2021	26.14385211012	0.682432216	2.11536446195552	24.0284876481645	Decrease

TableS3 Frontier analysis（2021）

	location	year	val	SDI	frontier	eff_diff	trend
209	Turkiye	2021	151.53118389156	0.712692673	2.11167876294958	149.41950512861	Increase
210	Turkmenistan	2021	45.06533000192	0.682160776	2.11530393648	42.95002606544	Decrease
211	Tuvalu	2021	82.81466245684	0.576620529	2.11444642002052	80.7002160368195	Decrease
212	UK	2021	179.4357118654	0.859000182	2.1135061360849	177.322205729315	Decrease
213	USA	2021	213.53740747663	0.862448354	2.11269866942333	211.424708807207	Increase
214	Uganda	2021	10.74901983074	0.423261181	2.112829987045	8.636189843695	Decrease
215	Ukraine	2021	223.14427661069	0.760773913	2.11236820150488	221.031908409185	Increase
216	United Arab Emirates	2021	53.91632322822	0.849317734	2.11596857990638	51.8003546483136	Decrease
217	Uruguay	2021	259.73703791588	0.719283445	2.11155018027164	257.625487735608	Increase
218	Uzbekistan	2021	25.3725564136	0.662621694	2.11306534140159	23.2594910721984	Increase
219	Vanuatu	2021	14.16958146742	0.473100706	2.11318005389658	12.0564014135234	Decrease
220	Venezuela	2021	74.32404704426	0.596513059	2.11486323671642	72.2091838075436	Increase
221	Vietnam	2021	13.02760651975	0.627933721	2.11509663135828	10.9125098883917	Decrease
222	Virgin Islands	2021	54.64792328351	0.821830853	2.11621414147109	52.5317091420389	Decrease
223	Western Europe	2021	221.37615033618	0.848726316	2.11385289971584	219.262297436464	Increase
224	Western sub-Saharan Africa	2021	6.3456618399	0.446022979	2.11541270181537	4.23024913808463	Decrease
225	Yemen	2021	68.07760518218	0.450376375	2.11239533422096	65.965209847959	Increase
226	Zambia	2021	5.55418950537	0.505948954	2.11247306752987	3.44171643784013	Decrease
227	Zimbabwe	2021	6.45204773822	0.473819486	2.11390302513726	4.33814471308274	Decrease

Table S4 BAPC results

mean	sd	year
104.072558996328	0.0987643980476862	2021
102.377744648636	1.09813093122958	2022
100.83283976038	1.57687121012327	2023
99.2636704016172	2.16017971275635	2024
97.6628440247844	2.82010638523532	2025
96.0811021600697	3.55655098089615	2026
94.4804146399008	4.37319275568685	2027
92.8421004913343	5.23844698579764	2028
91.1613218601982	6.13802673538446	2029
89.4516869432886	7.07063347094591	2030
87.7744061755729	8.04814073779264	2031
86.1313024150848	9.08072189671473	2032
